# Supplementary material for: Facile access to C-glycosyl amino acids and peptides via Ni-catalyzed reductive hydroglycosylation of alkynes
Source: Nat Commun. 2021 Aug 13;12:4924. doi: 10.1038/s41467-021-25127-z (PMC8363649; doi:10.1038/s41467-021-25127-z)
Supplement: Supplementary file 1 — Supplementary Information [file 41467_2021_25127_MOESM1_ESM.pdf]

Supplementary Information

Facile Access to *C*-Glycosyl Amino Acids and Peptides via Ni-Catalyzed Reductive Hydroglycosylation of Alkynes

Liu et al.

1. General Information 3
2. Experimental Section..4
2.1 Preparation of glycosyl bromides..4
2.2 Preparation of alkyne derivatives of amino acids and peptides 25
2.3 Optimization of reaction conditions with mannose donors...............................37
2.4 Optimization of reaction conditions with glucosamine donors (GlcN) 40
2.5 Ni-Catalyzed synthesis of vinyl *C*-glycosyl amino acids and glycopeptides.....45
2.6 The scale-up reaction 68
2.7 Transformations of vinyl *C*-glycosyl amino acids 69
2.8 Radical clock experiment..78
3. NMR Spectra 81
4. Supplementary References...195

1. General Information

All commercially available chemicals were used as received without further purification, unless otherwise stated. The catalytic reactions were carried out in Schlenk flasks under N_2 atmosphere using pre-dried glassware. Thin layer chromatography (TLC) was performed on TLC silica gel 60 F254 (Merck). Preparative layer chromatography was performed on PLC silica gel 60 F254 (Merck, 0.5 mm). The TLC plates were visualized with UV light and/or by charring with EtOH/H_2SO_4 (8%, v/v) or staining with a basic solution of $KMnO_4$. NMR spectra were measured on Bruker AM 400, Bruker AM 500, Agilent 500, or 600 MHz NMR spectrometer using TMS as an internal standard. Chemical shifts were given relative to TMS (0.00 ppm), $CDCl_3$ (7.26 ppm for ^{1}H NMR, 77.16 ppm for ^{13}C NMR), D_2O (4.79 ppm for ^{1}H NMR), or CD_3OD (3.31 ppm for ^{1}H NMR, 49.03 ppm for ^{13}C NMR). The following abbreviations were used to describe peak splitting patterns when appropriate: br = broad, s = singlet, d = doublet, t = triplet, q = quartet, dd = doublet of doublet, m = multiplet. Coupling constants (*J*) were reported in hertz (Hz). High-resolution mass spectra were recorded with IonSpec 4.7 Tesla FTMS or APEXIII 7.0 Tesla FTMS. Mass spectroscopy data of the products were collected on an HRMS-TOF instrument using ESI ionization. Optical rotations were measured on an Anton Paar MCP5500 S2 polarimeter.

2. Experimental Section

2.1 Preparation of glycosyl bromides

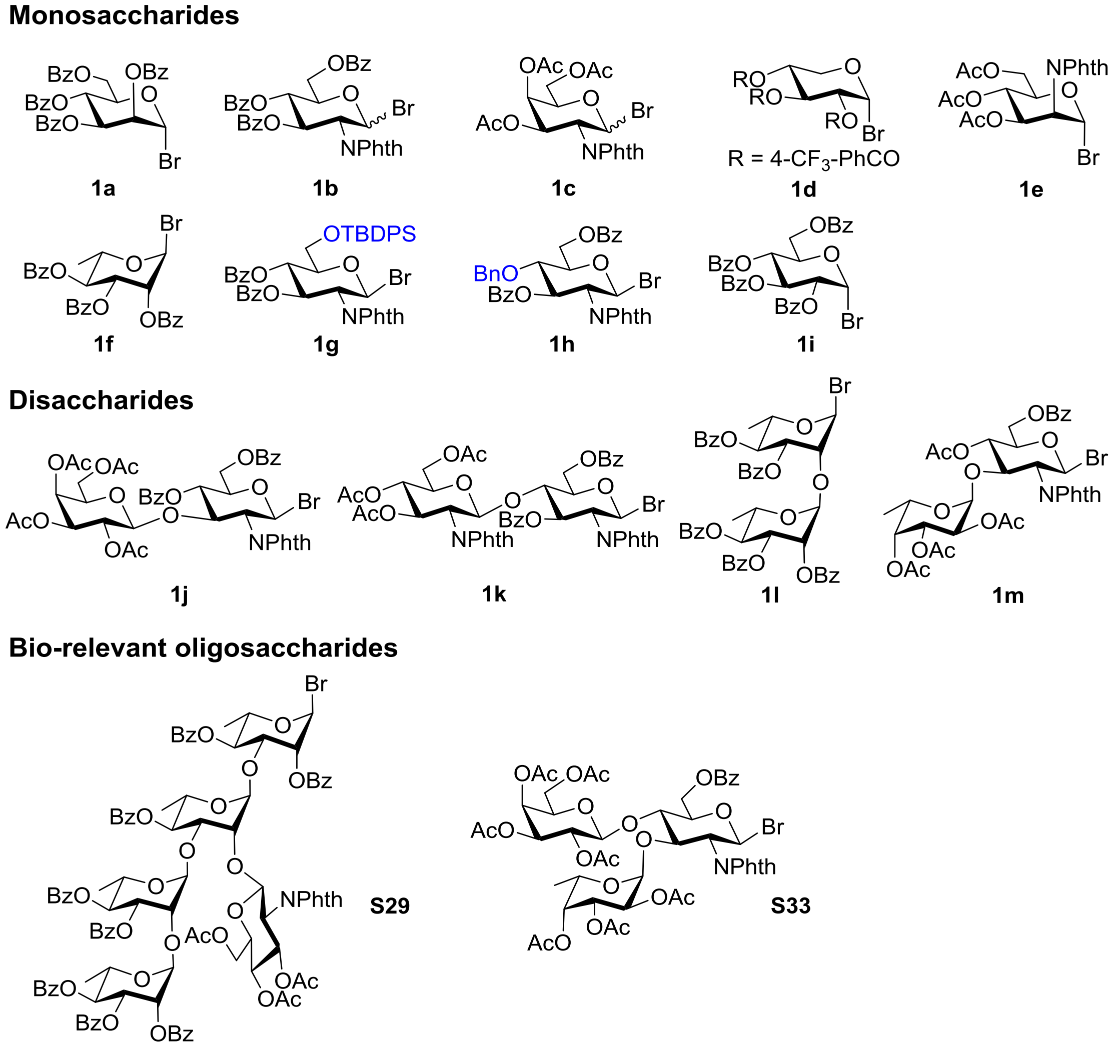

Supplementary Figure 1. Glycosyl bromides used in this study.

Method A. The glycosyl bromides were prepared from the corresponding glycosyl acetate or benzoate via treatment with 33% HBr in HOAc[1]. (pls check if the cited ref. 1 correct)

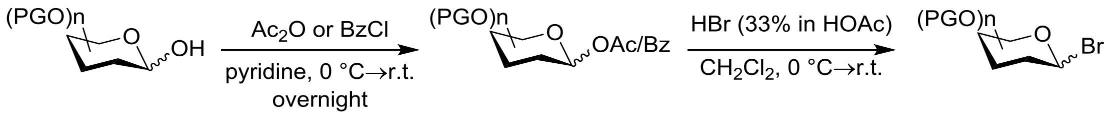

Supplementary Figure 2. General Method A for preparation of glycosyl bromides.

Unless otherwise stated, the fully protected glycosyl acetate/benzoate was dissolved in CH_2Cl_2 (0.6 M, x mL) and cooled to 0 ℃, 33% HBr in HOAc (0.5x mL) was added dropwise under 0 ℃. The ice bath was removed and the mixture was stirred at room

temperature for 2–7 h, until the disappearance of the starting material as monitored by TLC. The mixture was diluted with CH_2Cl_2 and washed with ice-water (3 times), sat. aq. $NaHCO_3$ (3 times) or 0.5 M aq. $KHCO_3$ (3 times), and finally brine (3 times). The organic layer was dried over Na_2SO_4, filtered, and concentrated to give the glycosyl bromide. In most cases, the bromide product was pure and was used directly for the subsequent coupling reaction or stored at -20 ℃.

Compounds **1a**, **1f** and **1i** are known compounds and were prepared according to the literature methods[1]. (pls check if the cited ref. 1 correct)

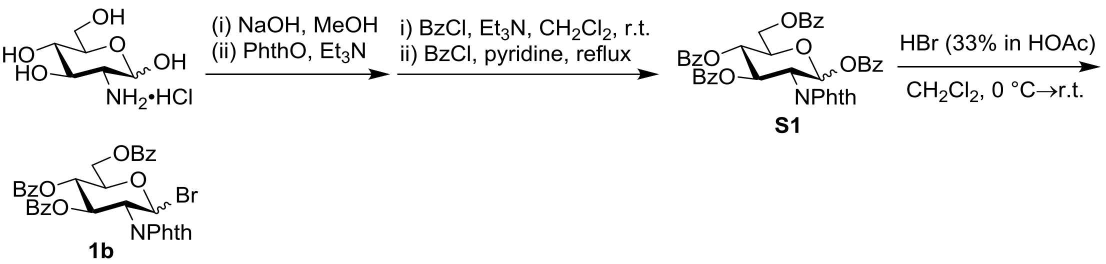

Supplementary Figure 3. Preparation of GlcN bromide **1b**.

Compound **1b** is a known compound and was prepared employing a modified literature procedure[2,3].

To a solution of glucosamine hydrochloride (4.3 g, 20 mmol) in MeOH (100 mL) were added sodium hydroxide (0.88 g, 22 mmol) and a trace amount of H_2O. After stirring rigorously at room temperature for 0.5 h, phthalic anhydride (1.63 g, 11 mmol) was added and the stirring was kept for ~0.5 h until a clear solution was resulted. To the solution was added Et_3N (3.0 mL, 22 mmol). The mixture was stirred for 15 min and white syrup appeared. The mixture was treated with a second portion of phthalic anhydride (1.63 g, 11 mmol) and stirred overnight at room temperature. The mixture was cooled to 0 ℃, filtered, and washed with cold MeOH. The filtrates were dried under reduced pressure to yield a white solid (5.0 g, 58%).

The above solid was suspended in dry CH_2Cl_2 (40 mL), to which Et_3N (16 mL, 116 mmol) was added. BzCl (10 mL, 87 mmol) was then added slowly at 0 ℃. After stirring at room temperature overnight, the mixture was diluted with CH_2Cl_2 (100 mL). The mixture was washed with 1 N aq. HCl (3×30 mL), sat. aq. $NaHCO_3$ (3×30 mL), and brine (3×30 mL) successively. The organic layer was dried over $MgSO_4$ and concentrated. The crude product was suspended in pyridine (20 mL), to which another portion of BzCl (1.3 mL, 11.6 mmol) was added. After refluxing for 4 h, the reaction mixture was concentrated, and the residue was redissolved in CH_2Cl_2 (200 mL). The mixture was washed with 1 N aq. HCl (3×30 mL), saturated $NaHCO_3$ (3×30 mL), and brine (3×30 mL) successively. The organic layer was dried over $MgSO_4$ and concentrated. The crude product was purified by column chromatography (petroleum ether/ethyl acetate = 2/1) to provide 1,3,4,6-tetra-*O*-benzoyl-2-deoxy-2-phthalimido-D-glucopyranose **S1** (6.9 g, 82%) as a white solid: ^1H NMR (500 MHz, $CDCl_3$) δ 8.06 (dd, J = 8.3, 1.3 Hz, 2H), 7.97 (dd, J = 8.4, 1.2 Hz, 2H), 7.91 (dd, J = 8.4, 1.2 Hz, 2H), 7.78 (dd, J = 8.4, 1.3 Hz, 4H), 7.66 (dd, J = 5.4, 3.0 Hz, 2H), 7.59–7.45 (m, 3H), 7.48–

7.39 (m, 3H), 7.41–7.36 (m, 2H), 7.36–7.31 (m, 2H), 7.28 (ddt, J = 8.6, 7.3, 1.4 Hz, 2H), 6.94 (d, J = 8.8 Hz, 1H), 6.48 (dd, J = 10.7, 9.2 Hz, 1H), 5.84 (t, J = 9.6 Hz, 1H), 4.95 (dd, J = 10.7, 8.8 Hz, 1H), 4.69–4.63 (m, 1H), 4.55–4.47 (m, 2H).

Compound **S1** (3.63 g, 5 mmol) was dissolved in dry CH_2Cl_2 (7.5 mL), and the mixture was cooled to 0 ℃, to which 33% HBr in HOAc (3.25 mL) was added dropwise. After warmed to room temperature, the mixture was stirred until the disappearance of starting material (TLC, 5–7 h). The mixture was diluted with CH_2Cl_2 (100 mL) and washed with ice-water (3×30 mL), 0.5 M aq. $KHCO_3$ (3×20 mL), and brine (3×30 mL) successively. The organic layer was dried over Na_2SO_4, filtered, and concentrated under reduced pressure to give **1b** as a white solid, which was pure enough and was used directly. It was found that **1b** was stable for at least one year when stored at -20 ℃.

3,4,6-Tri-*O*-acetyl-2-deoxy-2-phthalimido-D-galactopyranosyl bromide (**1c, β/α = 2:1**)

Compound **1c** was prepared as a pale yellow solid from known 1,3,4,6-tetra-*O*-acetyl-2-deoxy-2-phthalimido-α/β-D-galactopyranose[4] (955 mg, 2 mmol) in quantitative yield, employing Method A.

1cβ: ^{1}H NMR (500 MHz, $CDCl_3$) δ 7.88 (m, 2H), 7.77 (m, 2H), 6.40 (d, J = 9.5 Hz, 1H), 5.77 (dd, J = 11.1, 3.4 Hz, 1H), 5.54 (d, J = 3.4 Hz, 1H), 4.86–4.82 (m, 1H), 4.23–4.18 (m, 1H), 2.24 (s, 3H), 2.08 (s, 3H), 1.86 (s, 3H). **1cα**: ^{1}H NMR (500 MHz, $CDCl_3$) δ 7.88 (m, 2H), 7.77 (m, 2H), 6.69 (d, J = 3.6 Hz, 1H), 6.53 (dd, J = 12.0, 3.1 Hz, 1H), 5.72 (dd, J = 3.2, 1.3 Hz, 1H), 4.90–4.78 (m, 1H), 4.60 (t, J = 6.7 Hz, 1H), 4.27–4.13 (m, 2H), 2.18 (s, 3H), 2.08 (s, 3H), 1.91 (s, 3H).

2,3,4-Tri-*O*-(4-trifluoromethylbenzoyl)-α-D-xylopyranosyl bromide (**1d**)

Compound **1d** was prepared as a white solid from 1,3,4,6-tetra-*O*-(4-trifluoromethylbenzoyl)-α/β-D-xylopyranose (838 mg, 1 mmol) in quantitative yield, employing Method A.

1d: ^{1}H NMR (500 MHz, $CDCl_3$) δ 8.11 (t, J = 8.4 Hz, 4H), 8.04 (d, J = 8.2 Hz, 2H), 7.69 (d, J = 8.2 Hz, 4H), 7.62 (d, J = 8.3 Hz, 2H), 6.80 (d, J = 4.0 Hz, 1H), 6.23 (t, J = 9.8 Hz, 1H), 5.55 (dt, J = 10.2, 5.2 Hz, 1H), 5.35 (dd, J = 9.9, 4.0 Hz, 1H), 4.37 (dd, J = 11.4, 5.9 Hz, 1H), 4.18 (t, J = 11.2 Hz, 1H); ^{13}C NMR (125 MHz, $CDCl_3$) δ 164.56, 164.36, 164.17, 130.58, 130.44, 130.30, 125.88-125.75 (m), 87.25, 71.65, 70.91, 69.15, 62.86; ^{19}F NMR (376 MHz, $CDCl_3$) δ -63.34, -63.38, -63.40.

3,4,6-Tri-*O*-acetyl-2-deoxy-2-phthalimido-α-D-mannopyranosyl bromide (**1e**)

Compound **1e** was prepared as a white solid, which should be stored at -20 ℃, from known 1,3,4,6-tetra-*O*-acetyl-2-deoxy-2-phthalimido-α/β-D-mannopyranose[4] (2.0 g, 4.2 mmol) in quantitative yield, employing Method A.

1e: ^{1}H NMR (500 MHz, $CDCl_3$) δ 7.88 (dd, J = 5.5, 3.1 Hz, 2H), 7.78 (dd, J = 5.5, 3.0 Hz, 2H), 6.82 (d, J = 3.5 Hz, 1H), 5.68 (dd, J = 6.7, 5.3 Hz, 1H), 5.58 (dd, J = 9.3, 6.7 Hz, 1H), 5.23 (dd, J = 5.3, 3.6 Hz, 1H), 4.46–4.41 (m, 1H), 4.36–4.29 (m, 2H), 2.17 (s, 3H), 2.12 (s, 3H), 1.95 (s, 3H); ^{13}C NMR (125 MHz, $CDCl_3$) δ 170.85, 169.88, 169.74,

167.52, 134.79, 131.27, 124.00, 84.45, 72.66, 68.66, 67.96, 62.22, 55.85, 20.92, 20.89, 20.77.

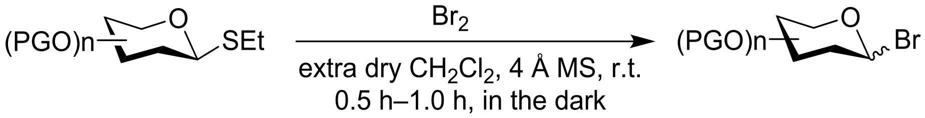

Supplementary Figure 4. General Method B for preparation of glycosyl bromides.

Method B. The glycosyl bromides were prepared from the corresponding ethyl thioglycosides via treatment with stoichiometric liquid bromine[5]. A mixture of the ethyl thioglycoside (1.0 equiv.) and activated molecular sieves (4 Å, 100 mg/mL CH_2Cl_2) in CH_2Cl_2 (0.1 M) was stirred under argon for 0.5 h. A freshly prepared solution of Br_2 (0.5–1.2 equiv.) in CH_2Cl_2 (1/165, v/v) was added. The reaction flask was wrapped with aluminum foil and was stirred for 30–60 min at room temperature in the dark. After dilution with CH_2Cl_2 and filtration, the mixture was washed with sat. aq. $Na_2S_2O_3$, sat. aq. $NaHCO_3$, and brine (2 times) successively. The organic phase was dried over Na_2SO_4, concentrated, and azeotroped with toluene for three times to afford the glycosyl bromide. Generally, the product was pure enough as determined by ^{1}H NMR analysis and was used directly for the subsequent coupling reaction. **Note**: i) glycosyl bromides are generally not stable on TLC plates; ii) some glycosyl bromides have similar mobility on TLC plates with the thioglycosides, so the progress of the reactions was monitored by ^{1}H NMR.

Ethyl 6-*O*-*tert*-butyldiphenylsilyl-3,4-di-*O*-benzoyl-2-deoxy-2-phthalimido-1-thio-β-D-glucopyranosyl bromide (1g)

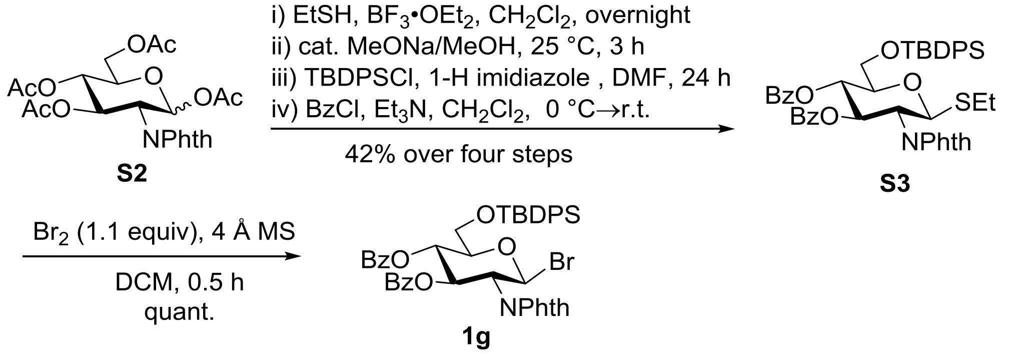

Supplementary Figure 5. Preparation of GlcN bromide **1g**.

To a stirred solution of the commercially available 1,3,4,6-tri-*O*-acetyl-2-deoxy-2-phthalimido-D-glucopyranoside (**S2**) (5.0 g, 10.5 mmol) in extra dry CH_2Cl_2 (30 mL), was added EtSH (1.16 mL, 40 mmol). After which, BF_3 OEt_2 (5.08 ml, 40 mmol) was added slowly at 0 °C, and the mixture was stirred overnight. The reaction mixture was diluted with CH_2Cl_2 (100 mL), washed with water (3 ×50 mL), aq. $NaHCO_3$ (3 ×50 mL),

and brine (3×30 mL) successively. The organic phase was dried over $MgSO_4$. After concentration, the crude product was used directly in the next step.

To a stirred solution of the crude product in mixed solvents (CH_2Cl_2/MeOH, 1:10, 22 mL) was added MeONa (120 mg, 2.2 mmol). The mixture was stirred for 3 h. After completion, Amberlite® resin IR-120 (H^+ form) was added to neutralize the solution. The resin was filtered off, and the filtrate was concentrated and dried over oil pump to give the crude product as a solid.

To a stirred solution of one half of the obtained crude product in dry DMF (10 mL) were added imidazole (680 mg, 10 mmol) and TBDPSCl (1.57 mL, 12 mmol). After stirring at rt for 24 h, the solution was diluted with ethyl acetate (100 mL), washed with water (3×30 mL), and brine (3×30 mL). After concentration, the crude product was redissolved in dry CH_2Cl_2 (15 mL), and Et_3N (4.2 mL, 30 mmol) was added. BzCl (1.7 mL, 15 mmol) was added dropwise at 0 ℃ and the resulting mixture was stirred overnight at room temperature. The reaction mixture was diluted with CH_2Cl_2 (50 mL), washed with water (3×30 mL) and brine (3×30 mL), and was then concentrated to dryness. The residue was purified by column chromatography (petroleum ether/ethyl acetate = 5/1, v/v) to provide ethyl 6-*O*-*tert*-butyldiphenylsilyl-3,4-di-*O*-benzoyl-2-deoxy-2-phthalimido-1-thio-β-D-glucopyranoside **S3** (1.65 g, 42%) as a white solid: ^{1}H NMR (500 MHz, $CDCl_3$) δ 7.87 (dd, *J* = 13.8, 7.4 Hz, 3H), 7.77 (d, *J* = 7.4 Hz, 2H), 7.75–7.68 (m, 4H), 7.69–7.64 (m, 1H), 7.59 (d, *J* = 6.9 Hz, 2H), 7.51 (t, *J* = 7.4 Hz, 1H), 7.42 (t, *J* = 7.4 Hz, 1H), 7.38–7.32 (m, 6H), 7.28–7.25 (m, 2H), 7.20 (t, *J* = 7.5 Hz, 2H), 6.26 (t, *J* = 9.8 Hz, 1H), 5.69 (t, *J* = 9.7 Hz, 1H), 5.64 (d, *J* = 10.5 Hz, 1H), 4.65 (t, *J* = 10.4 Hz, 1H), 3.99 (dt, *J* = 10.1, 3.5 Hz, 1H), 3.86 (d, *J* = 3.5 Hz, 1H), 2.88–2.76 (m, 1H), 2.76–2.63 (m, 1H), 1.28 (t, *J* = 7.4 Hz, 3H), 1.04 (s, 9H); ^{13}C NMR (125 MHz, $CDCl_3$) δ 165.94, 165.13, 135.82, 135.67, 133.31, 133.19, 133.10, 129.95, 129.91, 129.76, 129.71, 129.37, 128.88, 128.46, 128.41, 127.76, 127.72, 123.85, 123.76, 80.92, 79.37, 72.55, 69.74, 63.05, 54.24, 26.75, 24.05, 19.30, 15.24; HRMS (ESI) calcd. for $C_{46}H_{45}NO_8Na$ $(M + Na)^+$ m/z 822.2527, found 822.2526.

Compound **S3** (0.5 mmol) and activated molecular sieves (4Å, 800 mg) were suspended in extra dry CH_2Cl_2 (8 mL), and the mixture was stirred under argon for 0.5 h. To another round bottom flask wrapped with aluminum foil was charged with activated molecular sieves (4Å, 200 mg) and CH_2Cl_2 (2.1 mL), to which Br_2 (32 μL, 0.25 mmol) was injected under stirring. After stirring for ~20 mins in the dark, the mixture was transferred into the first bottle via syringe. The resulting mixture was stirred for 30 mins in the dark. After dilution with CH_2Cl_2 (20 mL), the molecular sieves were filtered off and washed with CH_2Cl_2. The organic phase was washed with sat. aq. $Na_2S_2O_3$, sat. aq. $NaHCO_3$, and brine successively. After dried over Na_2SO_4 and concentrated under vaccum, the crude product was azeotroped with toluene for three times to give **1g**, which was pure enough, as determined by ^{1}H NMR analysis, and was used directly. **1g**: ^{1}H NMR (500 MHz, $CDCl_3$) δ 7.91–7.82 (m, 4H), 7.79–7.70 (m, 6H), 7.60 (d, *J* = 6.8 Hz, 2H), 7.52 (t, *J* = 7.4 Hz, 1H), 7.43 (t, *J* = 7.4 Hz, 1H), 7.36–7.31 (m, 5H), 7.31 (d, *J* = 7.5 Hz, 1H), 7.28 (d, *J* = 7.7 Hz, 2H), 7.22 (t, *J* = 7.5 Hz, 2H), 6.54 (d, *J* = 9.5 Hz, 1H), 6.18 (dd, *J* = 10.4, 9.3 Hz, 1H), 5.80 (t, *J* = 9.7 Hz, 1H), 4.86

(dd, J = 10.4, 9.5 Hz, 1H), 4.02 (ddd, J = 10.1, 4.5, 2.2 Hz, 1H), 3.88 (qd, J = 11.9, 3.4 Hz, 2H), 1.06 (s, 9H); ^{13}C NMR (125 MHz, $CDCl_3$) δ 165.79, 164.94, 135.87, 135.75, 134.52, 133.47, 133.05, 133.03, 129.97, 129.94, 129.84, 129.78, 129.16, 128.59, 128.53, 128.47, 127.82, 127.75, 123.95, 80.04, 77.88, 71.62, 69.00, 62.70, 58.83, 26.81, 19.37.

4-*O*-Benzyl-3,6-di-*O*-benzoyl-2-deoxy-2-phthalimido-1-thio-β-D-glucopyranosyl bromide (1h)

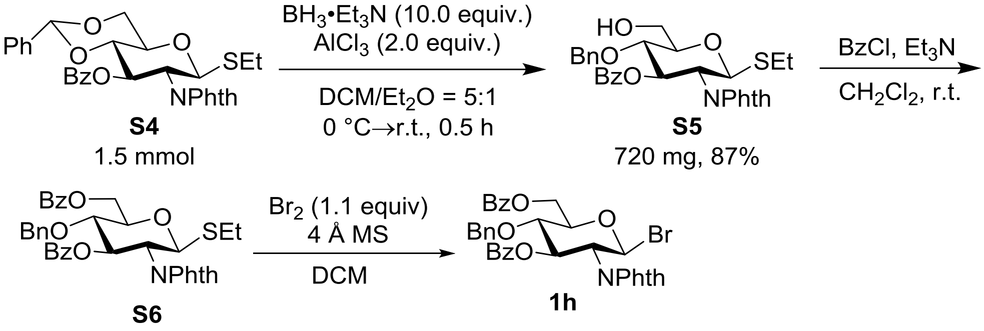

Supplementary Figure 6. Preparation of GlcN bromide **1h**.

To a solution of **S4**[6] (0.82 g, 1.5 mmol) and BH_3 Et_3N (2.19 mL, 15 mmol) in CH_2Cl_2 (25 mL) and Et_2O (5 mL) was added $AlCl_3$ (0.4 g, 3.0 mmol) at 0 ˚C. After stirring for 30 min at room temperature, sat. aq. $NaHCO_3$ was added slowly. The mixture was filtered through a celite pad and the aqueous layer was extracted with CH_2Cl_2. The combined organic layer was washed with water and brine, and was dried over Na_2SO_4. After filtration and removal of the solvent, the residue was purified by silica gel column chromatography (petroleum/ethyl acetate = 4/1 to 2/1) to afford ethyl 4-*O*-benzyl-3-*O*-benzoyl-2-deoxy-2-phthalimido-1-thio-β-D-glucopyranoside **S5** (0.72 g, 87%) as a white foam.

To a stirred solution of **S5** in dry CH_2Cl_2 (4 mL) was added Et_3N (1.0 mL, 6.0 equiv.). BzCl (150 μL, 3.0 equiv.) was added dropwise at 0 ℃. After stirring at room temperature for 4 h, the mixture was diluted with CH_2Cl_2 (20 mL), and was washed with water and brine. The organic layer was dried over Mg_2SO_4. After filtration and removal of the solvent, the residue was purified by silica gel column chromatography to afford ethyl 4-*O*-benzyl-3,6-di-*O*-benzoyl-2-deoxy-2-phthalimido-1-thio-β-D-glucopyranoside **S6** (754 mg, 89%) as a white solid: ^{1}H NMR (500 MHz, $CDCl_3$) δ 8.09 (d, J = 7.2 Hz, 2H), 7.88 (d, J = 7.8 Hz, 3H), 7.73–7.63 (m, 3H), 7.61 (t, J = 7.4 Hz, 1H), 7.50 (q, J = 7.5 Hz, 3H), 7.36 (t, J = 7.7 Hz, 2H), 7.16–7.03 (m, 5H), 6.21 (dd, J = 10.4, 8.7 Hz, 1H), 5.61 (d, J = 10.5 Hz, 1H), 4.68 (dd, J = 12.0, 2.1 Hz, 1H), 4.61–4.43 (m, 4H), 4.03 (ddd, J = 9.8, 4.7, 2.1 Hz, 1H), 3.99–3.91 (m, 1H), 2.78–2.54 (m, 1H), 1.20 (t, J = 7.4 Hz, 3H); ^{13}C NMR (125 MHz, $CDCl_3$) δ 166.31, 165.69, 136.93, 134.39, 134.19, 133.46, 133.31, 131.32, 129.99, 129.89, 129.85, 129.22, 128.58, 128.50, 128.27, 128.13, 123.77, 81.33, 77.41, 76.70, 74.92, 74.50, 63.66, 54.31, 24.65, 15.17; HRMS (ESI) calcd. for $C_{37}H_{33}NO_8SNa$ $(M + Na)^+$ m/z 674.1819, found 674.1825.

Compound **S6** (335 mg, 0.51 mmol) and activated molecular sieves (4 Å, 800 mg) were suspended in extra dry CH_2Cl_2 (8 mL), the mixture was stirred under argon for 0.5 h. Another round bottom flask wrapped with aluminum foil was charged with activated molecular sieves (4 Å, 300 mg) and CH_2Cl_2 (3.0 mL), to which Br_2 (32 μL, 0.25 mmol) was injected under stirring. After stirring for ~20 mins in the dark, the mixture was transferred into the first bottle via syringe. The resulting mixture was stirred for 30 mins in the dark. After dilution with CH_2Cl_2 (20 mL), the molecular sieves were filtered off and washed with CH_2Cl_2. The organic phase was washed with sat. aq. $Na_2S_2O_3$, sat. aq. $NaHCO_3$, and brine successively, and was then dried over Na_2SO_4. After concentration, the crude product was azeotroped with toluene for three times to give crude **1h**. ^{1}H NMR analysis showed a mixture containing **1h** (75%) together with the corresponding glycal (25%). The glycosyl bromide **1h** was not stable and was used directly in the next step. **1h**: ^{1}H NMR (500 MHz, $CDCl_3$) δ 8.11 (d, J = 7.3 Hz, 2H), 7.95–7.78 (m, 4H), 7.70 (s, 2H), 7.63 (t, J = 7.3 Hz, 1H), 7.55–7.51 (m, 3H), 7.40–7.33 (m, 3H), 7.13–7.03 (m, 4H), 6.53 (d, J = 9.4 Hz, 1H), 6.14 (dd, J = 10.5, 7.8 Hz, 1H), 4.79–4.72 (m, 1H), 4.68 (d, J = 10.8 Hz, 1H), 4.64–4.59 (m, 1H), 4.58–4.51 (m, 2H), 4.07–4.03 (m, 2H); ^{13}C NMR (125 MHz, $CDCl_3$) δ 166.24, 165.53, 134.51, 133.63, 133.44, 130.01, 129.87, 128.64, 128.55, 128.41, 128.30, 123.94, 78.31, 77.94, 75.88, 75.04, 73.53, 63.02, 58.75.

4,6-Di-*O*-benzoyl-2-deoxy-2-phthalimido-3-*O*-(2,3,4,6-tetra-*O*-acetyl-β-D-galactopyranosyl)-β-D-glucopyranosyl bromide (1j)

Compound **1j** was prepared employing a modified literature procedure[7,8].

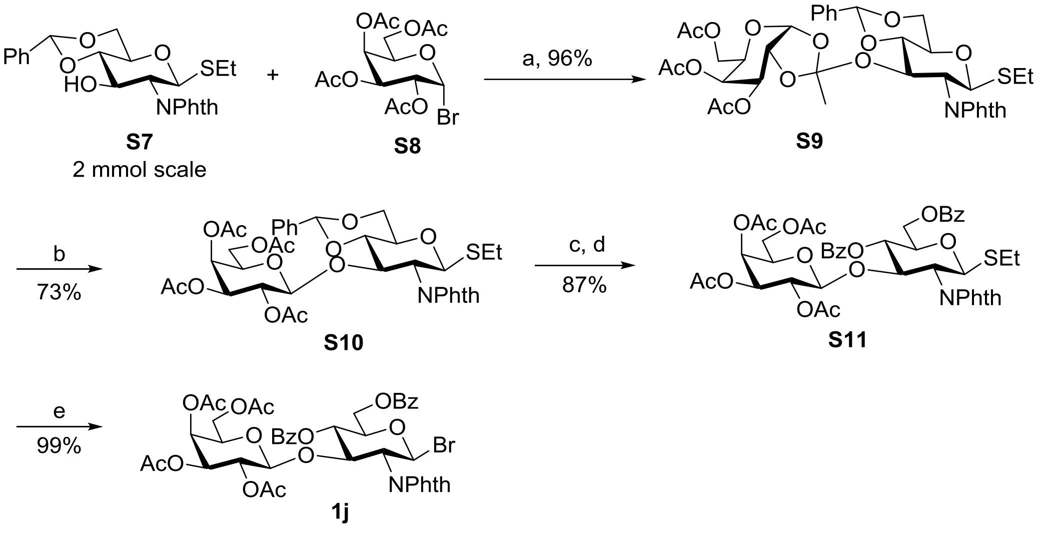

Supplementary Figure 7. Preparation of disaccharide bromide **1j**. Reagents and conditions: a) DTBMP (1.0 equiv.), AgOTf (1.65 equiv.), CH_2Cl_2, 4 Å MS, 10 ℃, 1 h, then 25 ℃, 7.5 h, 96%; b) TMSOTf (5 mol%), CH_2Cl_2, 4Å MS, 18 h, 73%; c) aq. HOAc (HOAc/H_2O = 3:2), 60 ℃, 0.5 h; d) BzCl (6.0 equiv.), DMAP (cat.), pyridine, 4 h, 60 ℃, 87% for 2 steps; e) Br_2 (1.0 equiv.), CH_2Cl_2, 4 Å MS, 25 ℃, in the dark, 0.5 h; this step was repeated twice to give **1j** quantitatively.

To a mixture of **S7**[7,8] (882 mg, 2.0 mmol) and **S8** (1.233 g, 3.0 mmol) in dry CH_2Cl_2 (25 mL) were added activated molecular sieves (4 Å, 2.5 g) and DTBMP (2,6-di-*tert*-butyl-4-methylpyridine) (0.42 g, 2.0 mmol). The mixture was stirred at -30 ℃ for 30 mins, to which a solution of silver triflate (848 mg, 3.3 mmol) in dry toluene (8 mL) was added under argon. The mixture was stirred at 10 ℃ for 1 h, after which the mixture was warmed to room temperature and stirred for 7.5 h. TLC (petroleum ether/ethyl acetate = 2:1, R_f = 0.3) showed the complete consumption of the acceptor. The reaction was quenched with Et_3N (2.0 mL). After dilution with CH_2Cl_2 and filtration, the filtrate was successively washed with water and brine, and was dried over Na_2SO_4. After concentration, the residue was purified by column chromatography (petroleum ether/ethyl acetate = 2:1) to give **S9** (1.49 g, 96%) as a white foam: ^{1}H NMR (500 MHz, $CDCl_3$) δ 7.95–7.87 (m, 2H), 7.53–7.45 (m, 2H), 7.79–7.72 (m, 2H), 7.53–7.45 (m, 3H), 7.37–7.32 (m, 5H), 5.59 (d, *J* = 4.7 Hz, 1H), 5.57 (s, 1H), 5.39 (d, *J* = 10.7 Hz, 1H), 5.21 (t, *J* = 2.9 Hz, 1H), 4.72–4.65 (m, 2H), 4.41 (dd, *J* = 10.4, 4.5 Hz, 1H), 4.30 (dd, *J* = 10.7, 9.6 Hz, 1H), 4.09 (td, *J* = 6.6, 2.5 Hz, 1H), 4.02 (dd, *J* = 11.3, 6.7 Hz, 1H), 3.96 (dd, *J* = 11.3, 6.5 Hz, 1H), 3.81 (t, *J* = 9.9 Hz, 1H), 3.75–3.67 (m, 3H), 2.77–2.61 (m, 2H), 2.02 (s, 3H), 1.99 (s, 3H), 1.86 (s, 3H), 1.53 (s, 3H), 1.20 (t, *J* = 7.4 Hz, 3H); ^{13}C NMR (125 MHz, $CDCl_3$) δ 170.48, 169.79, 169.64, 168.66, 167.54, 137.25, 134.32, 134.20, 132.17, 131.68, 129.06, 128.30, 126.12, 123.73, 123.41, 121.79, 101.50, 97.55, 81.97, 80.56, 73.77, 71.28, 70.91, 70.88, 68.97, 68.73, 65.66, 61.19, 55.05, 24.84, 24.26, 20.81, 20.77, 20.60, 14.99.

A suspension of **S9** (1.49 g, 1.93 mmol) and molecular sieves (4 Å, 2.0 g) in extra dry CH_2Cl_2 (20 mL) was stirred at room temperature for 30 min, to which TMSOTf (~19 µL, 5 mol%) was added. The mixture was stirred for 18 h and was then diluted with CH_2Cl_2 (30 mL). After filtration and concentration, the crude product was purified by column chromatography (petroleum ether/ethyl acetate = 2:1 or petroleum ether/dichloromethane/ethyl acetate = 2:1:0.5) to give ethyl 4,6-*O*-benzylidene-2-deoxy-2-phthalimido-3-*O*-(2,3,4,6-tetra-*O*-acetyl-β-D-galactopyranosyl)-1-thio-β-D-glucopyranoside **S10** (1.07 g, 73%) as a white foam: ^{1}H NMR (500 MHz, $CDCl_3$) δ 7.92–7.81 (m, 2H), 7.75 (q, *J* = 3.5 Hz, 2H), 7.49–7.42 (m, 2H), 7.34 (dd, *J* = 5.1, 2.0 Hz, 3H), 5.55 (s, 1H), 5.26 (d, *J* = 10.7 Hz, 1H), 5.17 (dd, *J* = 3.5, 1.1 Hz, 1H), 4.97 (dd, *J* = 10.4, 8.0 Hz, 1H), 4.75 (dd, *J* = 9.2, 8.1 Hz, 1H), 4.71 (dd, *J* = 9.6, 2.7 Hz, 1H), 4.52 (d, *J* = 8.0 Hz, 1H), 4.41–4.30 (m, 2H), 4.01 (dd, *J* = 11.0, 8.1 Hz, 1H), 3.81 (d, *J* = 5.3 Hz, 1H), 3.79 (d, *J* = 3.0 Hz, 1H), 3.80–3.75 (m, 1H), 3.67 (td, *J* = 9.7, 4.8 Hz, 1H), 3.47 (ddd, *J* = 8.2, 5.5, 1.2 Hz, 1H), 2.73–2.56 (m, 2H), 2.03 (s, 3H), 1.88 (s, 3H), 1.81 (s, 3H), 1.53 (s, 3H), 1.13 (t, *J* = 7.4 Hz, 3H); ^{13}C NMR (125 MHz, $CDCl_3$) δ 170.22, 170.01, 170.00, 168.86, 168.35, 167.01, 137.00, 134.55, 134.19, 131.68, 131.52, 129.27, 128.34, 126.02, 123.97, 123.22, 101.40, 100.39, 81.69, 80.88, 77.36, 76.41, 70.95, 70.62, 70.28, 69.15, 68.63, 66.61, 60.78, 54.16, 23.86, 20.60, 20.55, 20.43, 20.10, 14.80.

A solution of **S10** (414.3 mg, 0.55 mmol) in acetic acid/water (7.5 mL, 3:2) was stirred at 60 ℃ for 0.5 h. After warming to room temperature, the solvents were

removed *in vacuo*. The residue diol was azeotroped with toluene for three times and was used directly in the next step.

To a solution of the diol in pyridine (1.0 mL) containing DMAP (10 mol%) was slowly added benzyl chloride (380 μL, 3.3 mmol) at room temperature. The mixture was heated to 65 ℃ and stirred for 3 h. After removal of solvent, the residue was purified by column chromatography (petroleum ether/ethyl acetate = 1:1) to afford ethyl 4,6-di-*O*-benzoyl-2-deoxy-2-phthalimido-3-*O*-(2,3,4,6-tetra-*O*-acetyl-β-D-galactopyranosyl)-1-thio-β-D-glucopyranoside **S11** (426 mg, 87%) as a white foam: ^{1}H NMR (500 MHz, $CDCl_3$) δ 8.03 (d, *J* = 8.3 Hz, 2H), 7.99 (d, *J* = 8.2 Hz, 2H), 7.87 (d, *J* = 17.5 Hz, 2H), 7.79 (dd, *J* = 5.3, 2.5 Hz, 2H), 7.53 (dt, *J* = 15.0, 7.4 Hz, 2H), 7.39 (dt, *J* = 15.8, 7.6 Hz, 4H), 5.42 (t, *J* = 9.5 Hz, 1H), 5.29 (d, *J* = 10.5 Hz, 1H), 5.00 (s, 1H), 4. 93-4.86 (dt, *J* = 18.0, 9.7 Hz, 2H), 4.60 (d, *J* = 12.0 Hz, 1H), 4.54 (d, *J* = 10.4 Hz, 1H), 4.47 (t, *J* = 10.4 Hz, 1H), 4.41 (dd, *J* = 12.2, 5.4 Hz, 2H), 4.20 (d, *J* = 7.8 Hz, 1H), 4.17–4.13 (m, 1H), 3.55–3.41 (m, 2H), 3.32 (ddd, *J* = 10.1, 7.1, 1.4 Hz, 1H), 2.74–2.64 (m, 1H), 2.63–2.54 (m, 1H), 1.95 (s, 3H), 1.85 (s, 3H), 1.82 (s, 3H), 1.80 (s, 3H), 1.15 (t, *J* = 7.4 Hz, 3H); ^{13}C NMR (125 MHz, $CDCl_3$) δ 170.17, 170.08, 170.05, 169.13, 168.77, 167.17, 166.23, 164.64, 134.85, 134.71, 133.36, 133.12, 131.50, 131.29, 129.87, 129.84, 129.78, 129.69, 128.37, 123.83, 100.30, 81.32, 77.36, 76.21, 76.03, 70.94, 70.33, 69.02, 66.30, 63.66, 60.23, 54.72, 24.10, 20.68, 20.48, 20.44, 20.41, 14.97.

Compound **S11** (134 mg, 0.15 mmol) was dissolved in extra dry CH_2Cl_2 (3.0 mL), and activated molecular sieves (4 Å, 300 mg) was added. The mixture was stirred under argon for 0.5 h. Another round bottom flask wrapped with aluminum foil was charged with activated molecular sieves (4 Å, 150 mg) and CH_2Cl_2 (1.3 mL), to which Br_2 (7.7 μL, 0.15 mmol) was injected under stirring. After stirring for ~20 mins in the dark, the mixture was transferred into the first bottle via syringe. The resulting mixture was stirred for 30 mins in the dark. After dilution with CH_2Cl_2 (20 mL), the molecular sieves were filtered off and washed with CH_2Cl_2. The organic phase was washed with sat. aq. $Na_2S_2O_3$, sat. aq. $NaHCO_3$, and brine successively, and was then dried over Na_2SO_4. After concentration, the crude product was azeotroped with toluene for three times to give crude **1j**. ^{1}H NMR analysis showed that ~20% of the starting material was still left in the crude product. Thus, this procedure was repeated once again. ^{1}H NMR analysis showed completion of the reaction, and **1j** was pure enough and was used directly. **1j**: ^{1}H NMR (500 MHz, $CDCl_3$) δ 8.05 (t, *J* = 6.6 Hz, 4H), 7.92 (dd, *J* = 5.4, 3.0 Hz, 2H), 7.84 (dd, *J* = 5.5, 3.0 Hz, 2H), 7.60–7.53 (m, 2H), 7.43 (q, *J* = 8.0 Hz, 4H), 6.27 (d, *J* = 9.6 Hz, 1H), 5.54 (t, *J* = 9.6 Hz, 1H), 5.02 (d, *J* = 3.5 Hz, 1H), 4.93–4.86 (m, 2H), 4.73 (dd, *J* = 10.4, 9.6 Hz, 1H), 4.65 (dd, *J* = 12.3, 2.7 Hz, 1H), 4.57 (dd, *J* = 10.4, 3.5 Hz, 1H), 4.45 (dd, *J* = 12.4, 5.2 Hz, 1H), 4.26 (ddd, *J* = 9.9, 5.1, 2.7 Hz, 1H), 4.20 (d, *J* = 7.8 Hz, 1H), 3.55–3.46 (m, 2H), 3.38–3.30 (m, 1H), 1.98 (s, 3H), 1.90 (s, 3H), 1.83 (s, 3H), 1.82 (s, 3H); ^{13}C NMR (125 MHz, $CDCl_3$) δ 170.11, 170.00, 169.08, 166.16, 164.46, 134.99, 133.47, 133.22, 131.15, 129.86, 129.57, 129.47, 128.40, 128.39, 123.99, 100.23, 78.00, 77.36, 77.06, 75.14, 70.83, 70.40, 69.65, 68.90, 66.23, 63.08, 60.17, 58.99, 20.64, 20.45, 20.42, 20.36.

3,6-Di-*O*-benzoyl-2-deoxy-2-phthalimido-4-*O*-(3,4,6-tri-*O*-acetyl-2-deoxy-2-phthalimido-β-D-glucopyranosyl)-β-D-glucopyranosyl bromide (1k)

Compound **1k** was prepared employing a modified literature procedure[9].

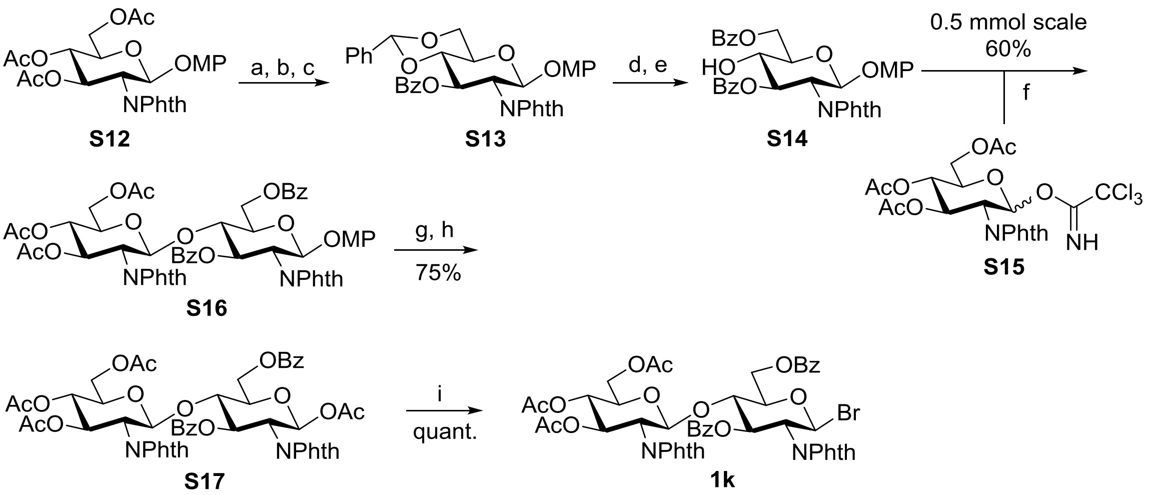

Supplementary Figure 8. Preparation of disaccharide bromide **1k**. Reagents and conditions: a) MeONa, MeOH/CH_2Cl_2 (1:1), 25 °C, 4 h; b) PhCH(OMe)$_2$, *p*-TSA (10 mol%), DMF, 50 °C, 5 h; c) BzCl (5.0 equiv.), pyridine, 40 °C, 4 h; d) aq. HOAc (HOAc/H_2O = 4:1, v/v), 80 °C, 1.5 h, 67% for 4 steps; e) BzCl (1.1 equiv.), pyridine/CH_2Cl_2 (1:3), 0 °C→25 °C, 12 h; then another portion of BzCl (1.1 equiv.) was added, 25 °C, 5 h, 75%; f) TMSOTf (20 mol%), CH_2Cl_2, 4 Å MS, -40 °C→0 °C, 6 h, 60%; g) CAN (5.0 equiv.), CH_3CN/H_2O (4:1), 25 °C, 1h; h) AcCl (5.0 equiv.), pyridine, 0 °C→25 °C, 0.5 h, 75% for 2 steps; i) HBr in HOAc, CH_2Cl_2, 0 °C→25 °C, 3 h, quant.

To a stirred solution of **S12** (2.7 g, 5 mmol) in MeOH/CH_2Cl_2 (1:1, v/v, 20 mL) was added MeONa (108 mg, 40 mol%). After stirring at room temperature for 4 h, the mixture was neutralized with Amberlite® resign IR-120 (H^+ form). The mixture was filtrated, concentrated, and dried over oil pump. The crude product was redissolved in DMF (10 mL). Benzaldehyde dimethyl acetal (2.3 mL, 15 mmol) was added and the reaction was kept rotating over vacuo evaporator at 50 °C for 5 h. After cooled to room temperature, the mixture was neutralized with Et_3N and concentrated. The residue was azeotroped with toluene for one time, and the crude *p*-methoxyphenyl 4,6-*O*-benzylidene-2-deoxy-2-phthalimido-β-D-glucopyranoside was dissolved in pyridine (10 mL). DMAP (10 mol%) was added, and benzyl chloride (1.7 mL, 15 mmol) was then added slowly at 0 °C. The mixture was heated to 50 °C and stirred for 2 h. After completion of the reaction, the solvent was removed *in vacuo*. The residue was dissolved in CH_2Cl_2 (50 mL), washed with 1 N aq. HCl (2×10 mL) and brine (2×10 mL). The organic phase was dried over $MgSO_4$ and concentrated to afford crude **S13**.

The crude **S13** was dissolved in acetic acid/water (4:1, v/v, 50 mL) and stirred at 80 °C for 1.5 h. The mixture was cooled to room temperature and neutralized carefully with sat. aq. $NaHCO_3$ at 0 °C. The mixture was extracted with ethyl acetate, dried over $MgSO_4$, and concentrated. The crude product was purified by column chromatography (petroleum ether/ethyl acetate = 2:1 then CH_2Cl_2/MeOH = 10:1) to afford *p*-

methoxyphenyl 3-*O*-benzoyl-2-deoxy-2-phthalimido-β-D-glucopyranoside (1.95 g, 67% over 4 steps).

To a stirred solution of *p*-methoxyphenyl 3-*O*-benzoyl-2-deoxy-2-phthalimido-β-D-glucopyranoside (1.04 g, 2.0 mmol) in pyridine/DCM (1:3, v/v, 28 mL) was added BzCl (253 μL, 1.0 equiv.) in CH_2Cl_2 (0.5 mL) dropwise under 0 ℃. After 12 h, about 30% of the starting material was converted, and another portion of BzCl (253 μL, 1.0 equiv.) in CH_2Cl_2 (0.5 mL) was added. After 12 h, the mixture was concentrated and purified by column chromatography to give *p*-methoxyphenyl 3,6-di-*O*-benzoyl-2-deoxy-2-phthalimido-β-D-glucopyranoside **S14** (940 mg, 75%): ^{1}H NMR (500 MHz, $CDCl_3$) δ 8.09 (d, *J* = 7.1 Hz, 2H), 7.90 (d, *J* = 7.2 Hz, 2H), 7.80 (dd, *J* = 5.3, 3.2 Hz, 2H), 7.65 (dd, *J* = 5.5, 3.0 Hz, 2H), 7.60 (t, *J* = 7.4 Hz, 1H), 7.53–7.49 (m, 1H), 7.47 (t, *J* = 7.8 Hz, 1H), 7.35 (t, *J* = 7.8 Hz, 2H), 6.89 (d, *J* = 9.1 Hz, 2H), 6.66 (d, *J* = 9.1 Hz, 2H), 6.01 (dd, *J* = 10.7, 8.7 Hz, 1H), 5.97 (d, *J* = 8.5 Hz, 1H), 4.78–4.71 (m, 3H), 4.09 (ddd, *J* = 9.8, 5.4, 2.9 Hz, 1H), 3.96 (td, *J* = 9.3, 3.9 Hz, 1H), 3.69 (s, 3H), 3.59 (d, *J* = 4.9 Hz, 1H); ^{13}C NMR (125 MHz, $CDCl_3$) δ 167.26, 166.89, 155.72, 150.70, 134.43, 133.74, 133.41, 131.45, 130.08, 130.03, 129.84, 128.79, 128.61, 128.56, 123.79, 118.97, 114.51, 97.63, 74.62, 74.50, 70.72, 63.83, 55.68, 54.51.

To a stirred solution of 3,4,6-tri-*O*-acetyl-2-deoxy-2-phthalimido-β-D-glucopyranoside (1.5 mmol) in dry CH_2Cl_2 (30 mL) were added CCl_3CN (1.5 mL, 15 mmol) and K_2CO_3 (4.5 mmol). The mixture was stirred for 3 h, and was then filtrated. After concentration and azeotroped with toluene, the crude 3,4,6-tri-*O*-acetyl-2-deoxy-2-phthalimido-β-D-glucopyranosyl trichloroacetimidate **S15** was obtained and was used directly.

A suspension of **S14** (312 mg, 0.5 mmol), **S15**, and activated molecular sieves (4 Å, 1 g) in extra dry CH_2Cl_2 (10 mL) was stirred at -40 ℃ for 0.5 h. TMSOTf (18 μL, 20 mol%) was added, and the resulting mixture was warmed to 0 ℃ slowly and stirred for 6 h. After quenching with triethylamine, the reaction mixture was concentrated. The residue was purified by silica gel column chromatography (petroleum ether/ethyl acetate = 2:1) to give *p*-methoxyphenyl 3,6-di-*O*-benzoyl-2-deoxy-2-phthalimido-4-*O*-(3,4,6-tri-*O*-acetyl-2-deoxy-2-phthalimido-β-D-glucopyranosyl)-β-D-glucopyranoside **S16** (311 mg, 60%) as a white foam: ^{1}H NMR (500 MHz, $CDCl_3$) δ 7.96 (d, *J* = 7.7 Hz, 2H), 7.86 (d, *J* = 7.7 Hz, 2H), 7.75–7.63(br, 5H), 7.61–7.51 (m, 5H), 7.47–7.42 (m, 4H), 6.76 (d, *J* = 8.6 Hz, 2H), 6.59 (d, *J* = 8.7 Hz, 2H), 6.18 (dd, *J* = 10.4, 8.6 Hz, 1H), 5.87 (d, *J* = 8.5 Hz, 1H), 5.66 (d, *J* = 8.3 Hz, 1H), 5.58 (t, *J* = 9.9 Hz, 1H), 4.99 (t, *J* = 9.6 Hz, 1H), 4.59 (dd, *J* = 10.7, 8.3 Hz, 2H), 4.26 (q, *J* = 8.5 Hz, 2H), 4.07 (dd, *J* = 11.8, 4.9 Hz, 1H), 4.04–4.01 (m, 1H), 3.82 (dd, *J* = 12.2, 3.8 Hz, 1H), 3.65 (s, 1H), 3.55 (dd, *J* = 12.3, 2.2 Hz, 1H), 3.28 (dt, *J* = 10.2, 2.9 Hz, 1H), 2.00 (s, 3H), 1.88 (s, 3H), 1.77 (s, 3H); ^{13}C NMR (125 MHz, $CDCl_3$) δ 170.50, 170.02, 169.17, 165.30, 164.99, 155.62, 150.37, 134.24, 134.19, 133.61, 133.08, 131.35, 130.85, 129.70, 129.66, 129.48, 128.89, 128.70, 128.29, 123.65, 123.57, 118.99, 114.31, 97.56, 97.50, 76.36, 72.53, 72.13, 71.77, 70.39, 67.97, 62.45, 60.94, 55.48, 54.91, 54.88, 20.69, 20.46, 20.29.

To a stirred solution of **S16** (311.6 mg, 0.3 mmol) in CH_3CN/H_2O (4:1, v/v, 3 mL), ammonium cerium (IV) nitrate (1.5 mmol) was added. After stirring at room temperature for 1 h, the mixture was diluted with EtOAc (100 mL) and washed with water (3×30 mL) and brine (3×30 mL). The organic layer was dried over Na_2SO_4 and concentrated *in vacuo* to give the corresponding lactol.

To a stirred solution of the above crude product in pyridine (3.0 mL) was added Ac_2O (106 μL, 5.0 equiv.) in CH_2Cl_2 (0.9 mL) dropwise. The reaction was stirred at room temperature for 0.5 h. After evaporation of the solvent, the crude product was purified by column chromatography (petroleum ether/ethyl acetate = 2:1) to afford 3,6-di-*O*-benzoyl-2-deoxy-2-phthalimido-4-*O*-(3,4,6-tri-*O*-acetyl-2-deoxy-2-phthalimido-β-D-glucopyranosyl)-β-D-glucopyranosyl acetate **S17** (977 mg, 75% over two steps): ^{1}H NMR (500 MHz, $CDCl_3$) δ 7.94 (d, J = 7.4 Hz, 2H), 7.87 (d, J = 7.6 Hz, 2H), 7.82–7.62 (m, 4H), 7.63–7.55 (m, 4H), 7.47 (t, J = 7.7 Hz, 3H), 7.44 (t, J = 7.7 Hz, 3H), 6.56 (d, J = 8.9 Hz, 1H), 6.20 (dd, J = 10.4, 8.6 Hz, 1H), 5.66 (d, J = 8.4 Hz, 1H), 5.56 (dd, J = 10.7, 9.2 Hz, 1H), 4.98 (t, J = 9.6 Hz, 1H), 4.66–4.57 (m, 1H), 4.49 (dd, J = 10.4, 8.9 Hz, 1H), 4.32–4.27 (m, 2H), 4.25 (dd, J = 10.7, 8.5 Hz, 1H), 4.10–4.01 (m, 2H), 3.83 (dd, J = 12.3, 3.9 Hz, 1H), 3.53 (dd, J = 12.2, 2.2 Hz, 1H), 3.25 (ddd, J = 10.2, 3.9, 2.2 Hz, 1H), 2.02 (s, 3H), 1.91 (s, 3H), 1.87 (s, 3H), 1.76 (s, 3H); ^{13}C NMR (125 MHz, $CDCl_3$) δ 170.59, 170.07, 169.21, 168.53, 167.56, 165.32, 164.97, 134.37, 134.26, 133.74, 133.18, 129.79, 129.70, 129.48, 128.87, 128.79, 128.38, 123.65, 97.55, 89.67, 75.69, 73.04, 72.07, 71.81, 70.40, 68.02, 62.22, 61.00, 54.91, 53.96, 20.76, 20.52, 20.35.

To a stirred solution of **S17** (977 mg, 0.2 mmol) in dry CH_2Cl_2 (4.0 mL), 33% HBr in HOAc (0.25 mL) was added dropwise at 0 ℃. After stirring for 2 h, the mixture was diluted with CH_2Cl_2 (50 mL), and washed with ice-water (3×20 mL), sat. aq. $NaHCO_3$ (3×10 mL), and brine (3×20 mL) successively. The organic layer was dried over Na_2SO_4, filtered, and concentrated to give **1k** as a white solid, which was pure enough and was used directly. **1k**: ^{1}H NMR (500 MHz, $CDCl_3$) δ 7.91 (d, J = 7.5 Hz, 2H), 7.83 (d, J = 7.5 Hz, 2H), 7.78–7.54 (m, 10H), 7.45 (t, J = 7.8 Hz, 2H), 7.41 (t, J = 7.6 Hz, 2H), 6.43 (d, J = 9.5 Hz, 1H), 6.08 (dd, J = 10.2, 8.6 Hz, 1H), 5.63 (d, J = 8.4 Hz, 1H), 5.53 (dd, J = 10.6, 9.2 Hz, 1H), 4.95 (t, J = 9.6 Hz, 1H), 4.63 (t, J = 8.8 Hz, 2H), 4.60 (d, J = 8.7 Hz, 1H), 4.30 (t, J = 9.3 Hz, 1H), 4.22 (dd, J = 10.7, 8.4 Hz, 1H), 4.03 (dd, J = 12.2, 3.5 Hz, 1H), 4.01–3.95 (m, 1H), 3.79 (dd, J = 12.3, 3.7 Hz, 1H), 3.50 (dd, J = 12.3, 2.2 Hz, 1H), 3.21 (ddd, J = 10.1, 3.7, 2.2 Hz, 1H), 1.98 (s, 3H), 1.84 (s, 3H), 1.73 (s, 3H); ^{13}C NMR (125 MHz, $CDCl_3$) δ 170.53, 170.05, 169.20, 165.28, 164.88, 134.45, 133.83, 133.26, 131.24, 130.83, 129.80, 129.70, 129.31, 128.83, 128.73, 128.39, 123.81, 123.67, 97.66, 77.57, 77.36, 77.26, 75.54, 72.16, 71.83, 70.38, 67.92, 62.21, 60.91, 58.60, 54.91, 20.76, 20.51, 20.34.

3,4-Di-*O*-benzoyl-2-*O*-(2,3,4-tri-*O*-benzoyl-α-L-rhamnopyranosyl)-α-L-rhamnopyranosyl bromide (1l)

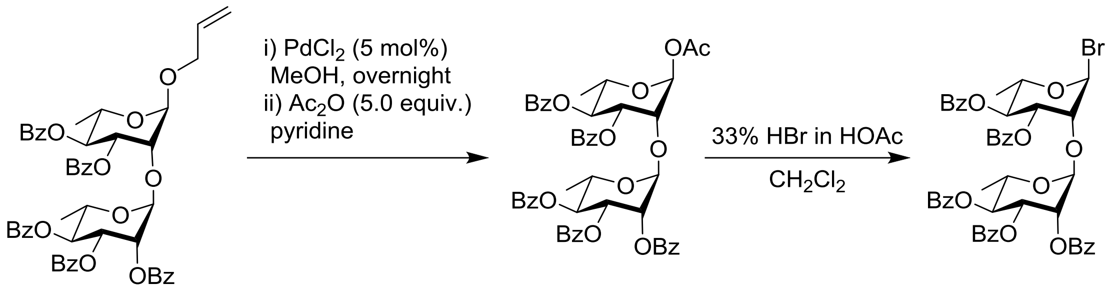

Supplementary Figure 9. Preparation of disaccharide bromide **1l**.

To a solution of **S18**[10] (130.6 mg, 0.15 mmol) in MeOH (1.5 mL) was added $PdCl_2$ (1.4 mg, 5 mol%). After stirring at room temperature for 12 h, the mixture was diluted with CH_2Cl_2, filtered, and concentrated to dryness. The crude product was dissolved in pyridine (2.0 mL), and Ac_2O (71 μL, 5.0 equiv.) was added under 0 ℃. After stirring at room temperature for 1.0 h, the solvent was removed, and the product was purified by column chromatography to afford 3,4-di-*O*-benzoyl-2-*O*-(2,3,4-tri-*O*-benzoyl-α-L-rhamnopyranosyl)-α-L-rhamnopyranosyl acetate **S19** (116.5 mg, 89% yield over two steps): ^{1}H NMR (500 MHz, $CDCl_3$) δ 8.04–7.96 (m, 8H), 7.84 (d, *J* = 7.2 Hz, 2H), 7.58 (t, *J* = 7.4 Hz, 1H), 7.55–7.49 (m, 2H), 7.47–7.37 (m, 8H), 7.33 (t, *J* = 7.6 Hz, 3H), 7.29–7.23 (m, 2H), 6.31 (d, *J* = 2.0 Hz, 1H), 5.95 (dd, *J* = 10.1, 3.5 Hz, 1H), 5.88 (dd, *J* = 3.5, 1.7 Hz, 1H), 5.81 (dd, *J* = 10.2, 3.2 Hz, 1H), 5.75–5.71 (m, 1H), 5.69 (t, *J* = 8.9 Hz, 1H), 5.19 (d, *J* = 1.7 Hz, 1H), 4.40–4.32 (m, 2H), 4.24 (dq, *J* = 9.5, 6.2 Hz, 1H), 2.25 (s, 3H), 1.43 (d, *J* = 6.2 Hz, 3H), 1.39 (d, *J* = 6.2 Hz, 3H); ^{13}C NMR (125 MHz, $CDCl_3$) δ 168.99, 165.97, 165.44, 165.36, 165.19, 133.52, 133.49, 133.35, 133.16, 130.11, 129.98, 129.95, 129.90, 129.87, 129.84, 129.42, 129.38, 129.27, 128.79, 128.65, 128.64, 128.57, 128.49, 128.37, 99.52, 92.40, 74.92, 71.72, 71.40, 70.74, 70.56, 69.74, 69.67, 67.97, 21.24, 17.87, 17.61.

To a stirred solution of **S19** (116.3 mg, 0.133 mmol) in dry CH_2Cl_2 (2.0 mL) was added 33% HBr in HOAc (0.5 mL) dropwise at 0 ℃. After stirring for 1 h, the mixture was diluted with CH_2Cl_2 (20 mL) and washed with ice-water (2×10 mL), sat. aq. $NaHCO_3$ (2×10 mL), and brine (2×10 mL) successively. The organic layer was dried over Na_2SO_4, filtered, and concentrated to give **1l** as a white solid, which was pure enough and was used directly. **1l**: ^{1}H NMR (500 MHz, $CDCl_3$) δ 8.06–7.98 (m, 8H), 7.84 (d, *J* = 8.3 Hz, 2H), 7.57 (t, *J* = 7.5 Hz, 1H), 7.53 (q, *J* = 6.5 Hz, 2H), 7.46–7.38 (m, 8H), 7.34 (t, *J* = 7.7 Hz, 2H), 7.26 (t, *J* = 7.5 Hz, 2H), 6.62 (d, *J* = 1.5 Hz, 1H), 6.17 (dd, *J* = 10.2, 3.3 Hz, 1H), 5.93 (dd, *J* = 10.1, 3.4 Hz, 1H), 5.82–5.74 (m, 2H), 5.70 (t, *J* = 10.0 Hz, 1H), 5.16 (d, *J* = 1.7 Hz, 1H), 4.60 (dd, *J* = 3.1, 1.5 Hz, 1H), 4.43–4.34 (m, 1H), 4.33 (dt, *J* = 12.5, 6.2 Hz, 1H), 1.47 (d, *J* = 6.3 Hz, 3H), 1.40 (d, *J* = 6.2 Hz, 3H); ^{13}C NMR (125 MHz, $CDCl_3$) δ 165.95, 165.82, 165.47, 165.35, 165.18, 133.58, 133.54, 133.51, 133.48, 133.21, 130.13, 129.99, 129.97, 129.95, 129.83, 129.33, 129.30, 129.25, 128.83, 128.67, 128.60, 128.55, 128.39, 99.90, 86.14, 80.30, 77.42, 71.87,

71.63, 71.23, 70.47, 69.78, 69.69, 68.17, 17.80, 17.44; HRMS (ESI) calcd. for $C_{32}H_{35}N_2O_{13}Na$ $(M + Na)^+$ *m/z* 915.1623, found 915.1626.

4-*O*-Acetyl-3-*O*-(2,3,4-tri-*O*-acetyl-α-L-fucopyranosyl)-6-*O*-benzoyl-2-deoxy-2-phthalimido-β-D-glucopyranosyl bromide (1m)

Compound **1m** was prepared employing a literature procedure[11].

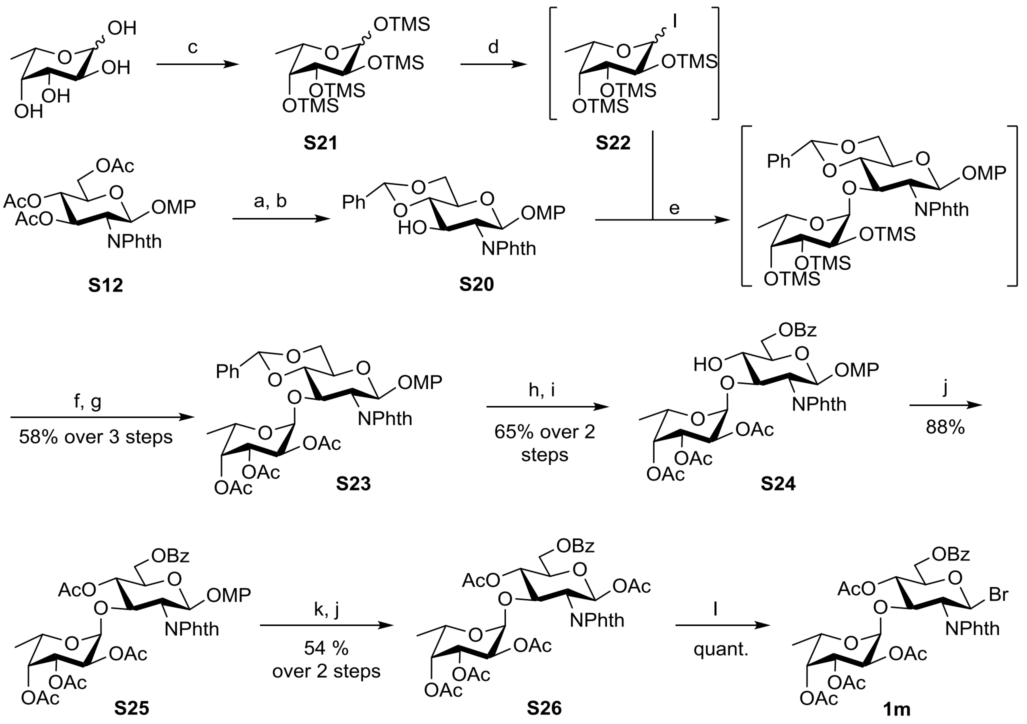

Supplementary Figure 10. Preparation of disaccharide bromide **1m**. Reagents and conditions: a) MeONa, MeOH/CH_2Cl_2 (10:1, v/v), 25 ℃, 4 h; b) $PhCH(OMe)_2$, *p*-TSA (10 mol%), DMF, 50 ℃, 5 h; c) TMSCl (5.2 equiv.), Et_3N (6.0 equiv.), DMF, 0 ℃→25 ℃, 5 h; d) TMSI (1.0 equiv.), 4 Å MS, CH_2Cl_2, 25 ℃, 1 h; e) 2,6-di-*tert*-butylpyridine (5.0 equiv.), 25 ℃, 36 h; f) MeOH/HOAc (6:1), 25 ℃, 1 h; g) Ac_2O (50 equiv.), pyridine, 25 ℃, overnight, 58% over 3 steps; h) HOAc/H_2O (3:2), 100 ℃, 1.5 h, 80%; i) BzCl (6.0 equiv.), pyridine, 25 ℃, 1 h, 82%; j) Ac_2O (10 equiv.), pyridine, 25 ℃, overnight, 88%; k) CAN (3.0 equiv.), CH_3CN/H_2O (4:1), 25 ℃; 1 h; then Ac_2O (15.0 equiv.), pyridine, 25 ℃, overnight, 57% over 2 steps; l) 33% HBr in HOAc, CH_2Cl_2, 0 ℃→25 ℃, 1 h, quantitative.

To a stirred solution of L-fucose (6.7 g, 40 mmol) and Et_3N (33 mL, 0.24 mol, 6.0 equiv.) in dry DMF (200 mL) was added TMSCl (26.36 mL, 0.208 mol, 5.2 equiv.) slowly at 0 ℃. The reaction mixture was stirred for 10 mins, after which the mixture was warmed to room temperature slowly and kept stirring for 5 h. After dilution with *n*-hexane (400 mL), the mixture was washed with water (4×400 mL) and brine (3×100 mL), and then dried over Na_2SO_4. After removal of solvent, pertrimethylsilyl fucose **S21** was obtained as a colorless oil (16.3 g, 90 %).

To a stirred solution of **S21** (13.58 g, 30 mmol) and 4Å MS (10 g) in dry CH_2Cl_2 (200 mL) was added TMSI (4.1 mL, 30 mmol, 1.0 equiv.) at room temperature. The

mixture was stirred for 60 min in the dark by wrapped in aluminum foil to give the corresponding iodide **S22**.

p-Methoxyphenyl 4,6-*O*-benzyliden-2-deoxy-2-phthalimido-β-D-glucopyranoside **S20** (5 g, 10 mmol) was added into the above reaction mixture. 2,6-Di-*tert*-butylpyridine (11.4 mL, 50 mmol) was dissolved in dry CH_2Cl_2 (100 mL) and transferred into the above mixture. The mixture was stirred at room temperature for 36 h, the molecular sieves were filtered off, the filtrate was concentrated and dried over oil pump. The crude product was dissolved in MeOH/HOAc (6:1, v/v, 48 mL), and the mixture was stirred at room temperature for 1 h. After evaporation of the solvent, the crude product was dried over oil pump and was dissolved in pyridine (100 mL). Acetic anhydride (47 mL, 0.5 mol) was added, and the mixture was stirred at room temperature overnight. After removal of solvent, the residue was dissolved in AcOEt (100 mL), and was then washed with 1 N aq. HCl (2×20 mL) and brine (3×30 mL). The organic layer was dried over $MgSO_4$ and concentrated. The crude product was purified by column chromatography to yield *p*-methoxyphenyl 4,6-*O*-benzylidene-3-*O*-(2,3,4-tri-*O*-acetyl-α-L-fucopyranosyl)-2-deoxy-2-phthalimido-β-D-glucopyranoside **S23** (4.5 g, 58 % over 3 steps): ^{1}H NMR (500 MHz, $CDCl_3$) δ 7.85 (dd, *J* = 5.6, 3.1 Hz, 2H), 7.76 (dd, *J* = 5.5, 3.0 Hz, 2H), 7.49 (dd, *J* = 7.7, 1.8 Hz, 2H), 7.41–7.32 (m, 3H), 6.77 (d, *J* = 9.2 Hz, 2H), 6.71 (d, *J* = 9.2 Hz, 2H), 5.62 (d, *J* = 8.5 Hz, 1H), 5.59 (s, 1H), 5.26 (dd, *J* = 10.9, 3.3 Hz, 1H), 5.03 (dd, *J* = 3.4, 1.2 Hz, 1H), 4.94–4.86 (m, 2H), 4.78 (dd, *J* = 11.0, 3.9 Hz, 1H), 4.56 (dd, *J* = 10.3, 8.5 Hz, 1H), 4.42 (dd, *J* = 10.4, 4.4 Hz, 1H), 4.33 (q, *J* = 6.6 Hz, 1H), 3.87 (t, *J* = 10.0 Hz, 1H), 3.83 (t, *J* = 9.0 Hz, 1H), 3.80–3.78 (m, 1H), 3.71 (s, 3H), 2.01 (s, 3H), 1.96 (s, 3H), 1.92 (s, 3H); ^{13}C NMR (125 MHz, $CDCl_3$) δ 170.66, 170.65, 170.07, 155.77, 150.68, 137.01, 134.62, 129.61, 128.52, 126.61, 123.80, 118.68, 114.63, 102.52, 98.42, 95.99, 80.35, 72.13, 71.29, 68.85, 68.04, 67.95, 66.72, 64.74, 56.40, 55.73, 20.81, 20.79, 20.69, 15.19; HRMS (ESI) calcd. for $C_{40}H_{41}NO_{15}Na$ $(M + Na)^+$ m/z 798.2368, found 798.2367.

A solution of **S23** (4.5 g, 5.8 mmol) in HOAc/H_2O (3:2, v/v, 60 mL) was stirred at 100 ℃ for 1.5 h. The reaction mixture was cooled to room temperature and neutralized carefully with sat. aq. $NaHCO_3$ under 0 ℃. The mixture was extracted with ethyl acetate, washed with brine, and dried over $MgSO_4$. After concentration, the crude product was purified by a short silica gel column chromatography (firstly with petroleum ether/ethyl acetate = 2:1 to remove the generated benzaldehyde, then with CH_2Cl_2/MeOH = 10:1) to afford the crude diol (3.2 g, 80%), which and was used directly in the next step.

The above crude diol (2.43 g, 3.53 mmol) was dissolved in pyridine (15 mL) and BzCl (2.0 mL, 17.6 mmol, 5.0 equiv.) was added. The mixture was stirred at room temperature for 1 h. After completion, as monitored by TLC (petroleum ether/ethyl acetate = 1:1), the solvent was removed *in vacuo*. The residue was dissolved in AcOEt (150 mL) and was then washed with 1 N sat. aq. HCl (2×30 mL) and brine (2×30 mL) successively. The organic layer was dried over $MgSO_4$ and concentrated. The crude product was purified by column chromatography to give *p*-methoxyphenyl 3-*O*-(2,3,4-tri-*O*-acetyl-α-L-fucopyranosyl)-6-*O*-benzoyl-2-deoxy-2-phthalimido-β-D-glucopyranoside **S24** (2.3 g, 82%): ^{1}H NMR (500 MHz, $CDCl_3$) δ 8.07 (d, *J* = 7.7 Hz,

2H), 7.87 (s, 1H), 7.73 (dd, *J* = 4.9, 3.0 Hz, 2H), 7.59 (t, *J* = 7.4 Hz, 1H), 7.46 (t, *J* = 7.6 Hz, 2H), 6.80 (d, *J* = 9.0 Hz, 2H), 6.60 (d, *J* = 8.9 Hz, 2H), 5.63 (d, *J* = 7.7 Hz, 1H), 5.31 (d, *J* = 1.9 Hz, 1H), 5.22 (ddd, *J* = 9.0, 3.5, 1.6 Hz, 1H), 5.08 (m, 2H), 4.74 (dd, *J* = 11.9, 2.2 Hz, 1H), 4.66 (dd, *J* = 11.9, 6.1 Hz, 1H), 4.57–4.46 (m, 3H), 3.90 (ddd, *J* = 8.8, 6.3, 2.1 Hz, 1H), 3.69 (t, *J* = 8.0 Hz, 1H), 3.66 (s, 3H), 2.10 (s, 3H), 1.89 (s, 3H), 1.30 (s, 3H), 1.13 (d, *J* = 6.5 Hz, 3H); ^{13}C NMR (125 MHz, $CDCl_3$) δ 170.49, 170.05, 169.80, 166.71, 155.56, 150.71, 134.44, 133.33, 131.66, 129.91, 129.86, 128.52, 123.80, 118.72, 114.41, 97.60, 97.49, 80.70, 80.67, 74.04, 70.94, 70.89, 67.63, 67.55, 66.18, 63.92, 55.61, 54.78, 20.69, 20.65, 19.62, 15.86; HRMS (ESI) calcd. for $C_{40}H_{41}NO_{16}Na$ $(M + Na)^+$ m/z 814.2318, found 814.2320.

To a stirred solution of **S24** (396 mg, 0.5 mmol) in pyridine (3 mL) was added Ac_2O (0.49 mL, 10 equiv.). After stirring at room temperature overnight, methanol (0.7 mL) was added, and the mixture was stirred for another one hour. After removal of the solvent, the crude product was purified by column chromatography (petroleum ether/ethyl acetate = 1:1) to yield *p*-methoxyphenyl 4-*O*-acetyl-3-*O*-(2,3,4-tri-*O*-acetyl-α-L-fucopyranosyl)-6-*O*-benzoyl-2-deoxy-2-phthalimido-β-D-glucopyranoside **S25** (366 mg, 88%): ^{1}H NMR (500 MHz, $CDCl_3$) δ 8.05 (d, *J* = 7.1 Hz, 2H), 7.78–7.70 (m, 2H), 7.59 (t, *J* = 8.0 Hz, 1H), 7.46 (t, *J* = 7.7 Hz, 2H), 6.81 (d, *J* = 9.1 Hz, 2H), 6.58 (d, *J* = 9.1 Hz, 2H), 5.73 (d, *J* = 8.0 Hz, 1H), 5.22 (dd, *J* = 3.4, 1.4 Hz, 1H), 5.15 (dd, *J* = 9.9, 8.3 Hz, 1H), 5.13 (dd, *J* = 11.0, 3.3 Hz, 1H), 5.05 (d, *J* = 3.4 Hz, 1H), 5.00 (dd, *J* = 11.0, 3.4 Hz, 1H), 4.65–4.48 (m, 3H), 4.36 (dd, *J* = 12.1, 7.3 Hz, 1H), 4.27 (q, *J* = 7.7, 7.0 Hz, 1H), 3.94 (ddd, *J* = 10.1, 7.3, 2.8 Hz, 1H), 3.66 (s, 3H), 2.10 (s, 3H), 2.09 (s, 3H), 1.82 (s, 3H), 1.48 (s, 3H), 1.06 (d, *J* = 6.5 Hz, 3H); ^{13}C NMR (125 MHz, $CDCl_3$) δ 170.52, 170.46, 170.11, 169.29, 166.21, 155.71, 150.63, 134.30, 133.33, 129.94, 129.91, 128.55, 118.96, 114.45, 97.54, 97.44, 72.59, 72.04, 71.43, 68.56, 67.20, 66.20, 63.40, 55.65, 55.13, 21.22, 20.74, 20.60, 20.11, 15.42; HRMS (ESI) calcd. for $C_{42}H_{43}NO_{17}Na$ $(M + Na)^+$ m/z 856.2423, found 856.2422.

To a stirred solution of **S25** (366 mg, 0.44 mmol) in CH_3CN/H_2O (4:1, v/v, 5 mL) was added diammonium cerium (IV) nitrate (723 mg, 1.32 mmol). After stirring at room temperature for 1 h, the mixture was diluted with EtOAc (50 mL) and washed with water (3×15 mL) and brine (3×15 mL) successively. The organic layer was dried over Na_2SO_4 and concentrated *in vacuo*. The crude product was dissolved in pyridine (4 mL), and Ac_2O (0.6 mL, 15 equiv.) was added. After stirring at room temperature overnight, methanol (1.0 mL) was added, and the mixture was stirred for another one hour. After removal of solvent, the crude product was purified by column chromatography (petroleum ether/ethyl acetate = 1:1) to give 4-*O*-acetyl-3-*O*-(2,3,4-tri-*O*-acetyl-α-L-fucopyranosyl)-6-*O*-benzoyl-2-deoxy-2-phthalimido-β-D-glucopyranosyl acetate **S26** (193 mg, 57% over 2 steps): ^{1}H NMR (500 MHz, $CDCl_3$) δ 8.09 (d, *J* = 7.2 Hz, 2H), 7.89 (dd, *J* = 5.1, 3.0 Hz, 2H), 7.76 (dd, *J* = 5.4, 3.0 Hz, 2H), 7.58 (t, *J* = 7.4 Hz, 1H), 7.47 (t, *J* = 7.7 Hz, 2H), 6.45 (d, *J* = 8.8 Hz, 1H), 5.20 (t, *J* = 9.4 Hz, 1H), 5.20 (d, *J* = 2.8 Hz, 1H), 5.09 (dd, *J* = 11.1, 3.3 Hz, 1H), 5.04 (d, *J* = 3.4 Hz, 1H), 4.99 (dd, *J* = 11.0, 3.4 Hz, 1H), 4.65 (dd, *J* = 10.6, 8.8 Hz, 1H), 4.51 (dd, *J* =

10.7, 9.0 Hz, 1H), 4.50 (dd, *J* = 11.7, 2.8 Hz, 1H), 4.32 (dd, *J* = 12.3, 5.4 Hz, 1H), 4.24 (q, *J* = 6.3 Hz, 1H), 3.99 (ddd, *J* = 9.9, 5.4, 2.7 Hz, 1H), 2.09 (s, 4H), 2.08 (s, 3H), 1.98 (s, 3H), 1.81 (s, 3H), 1.53 (s, 3H), 1.05 (d, *J* = 6.5 Hz, 3H); ^{13}C NMR (125 MHz, $CDCl_3$) δ 170.48, 170.39, 169.83, 169.30, 168.83, 167.67, 166.34, 134.38, 133.25, 131.59, 129.93, 129.82, 128.51, 124.01, 97.47, 89.78, 76.55, 73.14, 71.33, 71.28, 68.42, 67.09, 66.17, 62.85, 54.11, 21.15, 20.90, 20.68, 20.54, 20.14, 15.35; HRMS (ESI) calcd. for $C_{37}H_{39}NO_{17}Na$ $(M + Na)^+$ m/z 792.2110, found 792.2115.

To a stirred solutio of **S26** (123 mg, 0.16 mmol) in CH_2Cl_2 (1.0 mL) was added 33% HBr in HOAc (0.2 mL) dropwise under 0 ℃. The mixture was removed from the ice bath and stirred for 1 h at room temperature. The mixture was diluted with CH_2Cl_2 (50 mL), and was then washed with ice-water (3×20 mL), sat. aq. $NaHCO_3$ (3×10 mL), and brine (2×20 mL) successively. The organic layer was dried over Na_2SO_4, filtered, and concentrated to give **1m** as a pale yellow solid, which was in high purity and was used directly in the coupling reaction. **1m**: ^{1}H NMR (500 MHz, $CDCl_3$) δ 8.10 (br, 2H), 7.90 (s, 2H), 7.77 (dd, *J* = 5.8, 2.9 Hz, 2H), 7.59 (t, *J* = 7.0 Hz, 1H), 7.48 (t, *J* = 7.4 Hz, 2H), 6.35 (d, *J* = 9.5 Hz, 1H), 5.24 (t, *J* = 9.4 Hz, 1H), 5.20 (d, *J* = 2.9 Hz, 1H), 5.08 (dd, *J* = 11.0, 3.2 Hz, 1H), 5.03 (d, *J* = 3.4 Hz, 1H), 4.98 (dd, *J* = 11.1, 3.3 Hz, 1H), 4.67 (t, *J* = 9.9 Hz, 1H), 4.54 (dd, *J* = 12.9, 3.1 Hz, 1H), 4.50 (t, *J* = 9.3 Hz, 1H), 4.34 (dd, *J* = 12.6, 5.6 Hz, 1H), 4.23 (q, *J* = 6.7 Hz, 1H), 3.95 (ddd, *J* = 9.0, 5.6, 2.3 Hz, 1H), 2.08 (s, 6H), 1.81 (s, 3H), 1.54 (s, 3H), 1.05 (d, *J* = 6.4 Hz, 3H); ^{13}C NMR (125 MHz, $CDCl_3$) δ 170.47, 170.44, 169.77, 169.24, 166.35, 134.47, 133.39, 131.65, 129.99, 129.73, 128.57, 124.13, 97.51, 77.81, 77.41, 76.91, 71.34, 71.17, 68.47, 67.05, 66.27, 63.00, 58.68, 21.14, 20.71, 20.56, 20.18, 15.40.

2,4-Di-*O*-benzoyl-3-*O*-(4-*O*-benzoyl-3-*O*-(3,4-di-*O*-benzoyl-2-*O*-(2,3,4-tri-*O*-benzoyl-α-L-rhamnopyranosyl)-α-L-rhamnopyranosyl)-2-*O*-(3,4,6-tri-*O*-acetyl-2-deoxy-2-phthalimido-β-D-glucopyranosyl)-α-L-rhamnopyranosyl)-α-L-rhamnopyranosyl bromide (S29)

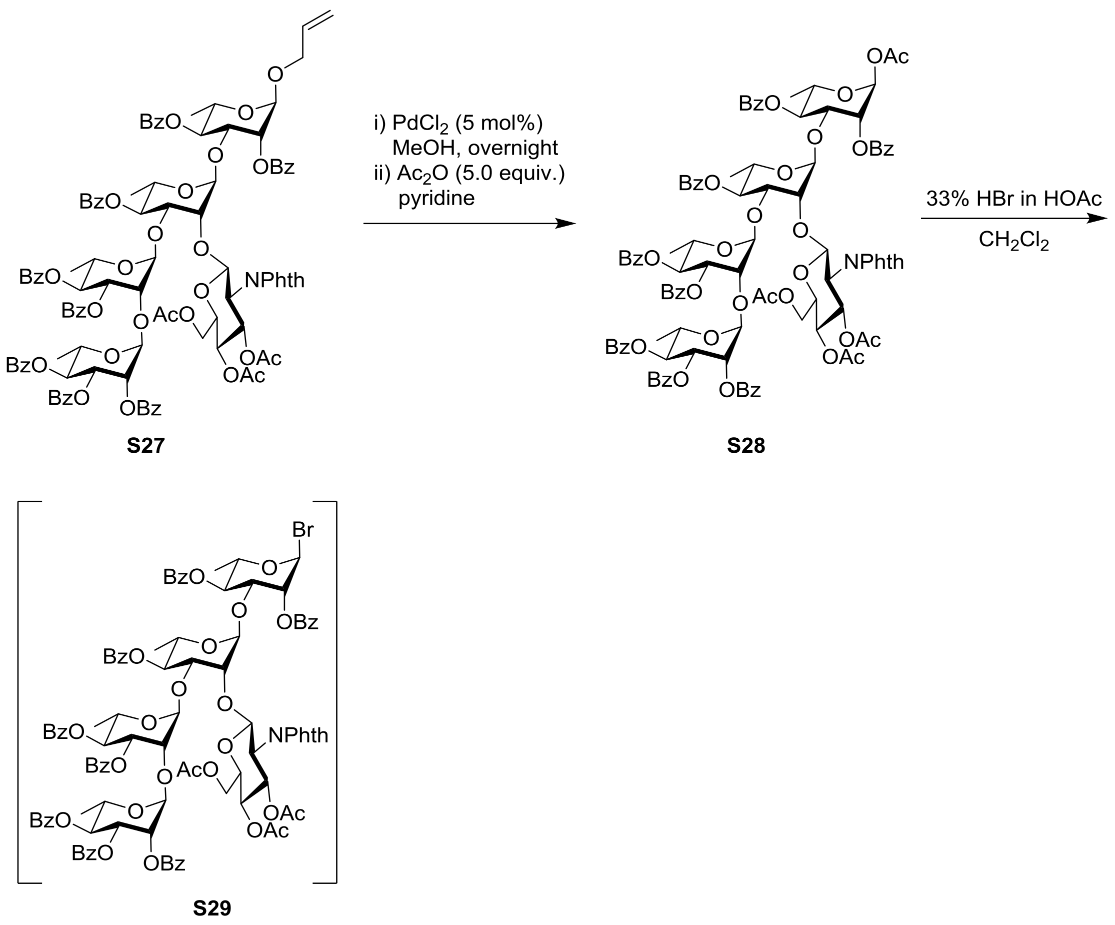

Supplementary Figure 11. Preparation of pentasaccharide bromide **S29**.

Allyl 2,4-di-*O*-benzoyl-3-*O*-(4-*O*-benzoyl-3-*O*-(3,4-di-*O*-benzoyl-2-*O*-(2,3,4-tri-*O*-benzoyl-α-L-rhamnopyranosyl)-α-L-rhamnopyranosyl)-2-*O*-(3,4,6-tri-*O*-acetyl-2-deoxy-2-phthalimido-β-D-glucopyranosyl)-α-L-rhamnopyranosyl)-α-L-rhamnopyranoside **S27** was prepared employing a literature procedure[10].

S27: ^{1}H NMR (500 MHz, $CDCl_3$) δ 8.14 (d, *J* = 7.2 Hz, 2H), 8.08–8.01 (m, 4H), 7.98 (d, *J* = 7.3 Hz, 2H), 7.94 (ddd, *J* = 8.4, 3.9, 1.3 Hz, 4H), 7.85–7.80 (m, 2H), 7.80 (d, *J* = 7.3 Hz, 2H), 7.61 (td, *J* = 17.7, 16.5, 7.5 Hz, 5H), 7.54 (d, *J* = 7.7 Hz, 2H), 7.52–7.48 (m, 5H), 7.45 (t, *J* = 7.7 Hz, 2H), 7.42–7.34 (m, 8H), 7.27 (d, *J* = 8.2 Hz, 2H), 7.24 (d, *J* = 8.0 Hz, 2H), 7.01 (t, *J* = 7.8 Hz, 2H), 6.84 (t, *J* = 7.5 Hz, 1H), 6.02–5.91 (m, 2H), 5.81 (dd, *J* = 10.0, 3.4 Hz, 1H), 5.77 (dd, *J* = 3.5, 1.7 Hz, 1H), 5.74 (dd, *J* = 10.3, 3.1 Hz, 1H), 5.65 (d, *J* = 8.4 Hz, 1H), 5.49 (d, *J* = 9.7 Hz, 1H), 5.45 (d, *J* = 10.0 Hz, 1H), 5.41 (t, *J* = 9.9 Hz, 1H), 5.39–5.33 (m, H), 5.27 (dq, *J* = 10.5, 1.4 Hz, 1H), 5.04 (d, *J* = 2.0 Hz, 1H), 5.02 (d, *J* = 1.7 Hz, H), 4.85 (d, *J* = 1.7 Hz, 1H), 4.79 (d, *J* = 1.7 Hz, 1H), 4.67 (t, *J* = 9.8 Hz, 1H), 4.39 (dd, *J* = 9.8, 3.4 Hz, 1H), 4.29 (dd, *J* = 10.7, 8.3 Hz, 1H), 4.23 (ddt, *J* = 10.1, 7.4, 2.1 Hz, 2H), 4.10–3.97 (m, 5H), 3.89 (t, *J* = 2.3 Hz, 1H), 3.83 (dq, *J* = 9.8, 6.1 Hz, 1H), 3.58 (ddd, *J* = 9.7, 6.3, 3.3 Hz, 2H), 3.53 (d, *J* = 10.1 Hz, 1H), 2.06 (s, 3H), 2.03 (s, 3H), 1.83 (s, 3H), 1.28 (d, *J* = 6.1 Hz, 3H), 1.26 (d, *J* = 6.3 Hz, 3H), 0.82 (d, *J* = 6.3 Hz, 3H), 0.69 (d, *J* = 6.2 Hz, 3H).

To a stirred solutio of **S27** (44.5 mg, 0.0235 mmol) in MeOH (0.5 mL) was added $PdCl_2$ (1.2 mg, 29 mol%). After stirring at room temperature for 12 h, the mixture was diluted with CH_2Cl_2, filtered, and concentrated to dryness. The crude product was dissolved in pyridine (2.0 mL), and Ac_2O (0.2 mL) was added at 0 ℃. The reaction mixture was stirred at room temperature for 2 h. After quenching with MeOH (0.2 mL) under 0 ℃ and stirring for another 2.0 h, the solvent was removed. The crude product was purified by preparative TLC (petroleum ether/CH_2Cl_2/ethyl acetate = 2:1:1) to afford 2,4-di-*O*-benzoyl-3-*O*-(4-*O*-benzoyl-3-*O*-(3,4-di-*O*-benzoyl-2-*O*-(2,3,4-tri-*O*-benzoyl-α-L-rhamnopyranosyl)-α-L-rhamnopyranosyl)-2-*O*-(3,4,6-tri-*O*-acetyl-2-deoxy-2-phthalimido-β-D-glucopyranosyl)-α-L-rhamnopyranosyl)-α-L-rhamnopyranosyl acetate **S28** (23.1 mg, 52% over two steps): ^{1}H NMR (500 MHz, $CDCl_3$) δ 8.14 (d, J = 7.3 Hz, 2H), 8.08–8.04 (m, 4H), 7.99 (d, J = 7.3 Hz, 2H), 7.95 (dd, J = 7.2, 4.4 Hz, 4H), 7.86–7.82 (m, 2H), 7.80 (d, J = 7.3 Hz, 2H), 7.69–7.58 (m, 5H), 7.58–7.48 (m, 7H), 7.47–7.43 (m, 2H), 7.43–7.34 (m, 8H), 7.27 (m, 4H), 7.02 (t, J = 7.8 Hz, 2H), 6.86 (t, J = 7.5 Hz, 1H), δ 6.25 (d, J = 1.9 Hz, 1H), 5.99 (dd, J = 10.8, 9.0 Hz, 1H), 5.81 (dd, J = 10.1, 3.4 Hz, 1H), 5.78–5.73 (m, 2H), 5.66 (d, J = 8.4 Hz, 1H), 5.53 (t, J = 9.9 Hz, 1H), 5.47 (t, J = 10.0 Hz, 1H), 5.42 (t, J = 9.9 Hz, 1H), 5.37 (dd, J = 3.5, 2.1 Hz, 1H), 5.11–5.02 (m, 2H), 4.85 (d, J = 1.7 Hz, 1H), 4.80 (d, J = 1.6 Hz, 1H), 4.69 (t, J = 9.8 Hz, 1H), 4.40 (dd, J = 9.9, 3.4 Hz, 1H), 4.28 (dd, J = 10.8, 8.4 Hz, 1H), 4.22 (dd, J = 10.0, 2.5 Hz, 1H), 4.10 (dd, J = 9.9, 6.2 Hz, 1H), 4.07 (dd, J = 3.2, 1.6 Hz, 1H), 4.03–3.96 (m, 2H), 3.92 (t, J = 2.3 Hz, 1H), 3.85 (dq, J = 12.4, 6.1 Hz, 1H), 3.60 (ddt, J = 9.9, 7.7, 3.6 Hz, 3H), 2.21 (s, 3H), 2.05 (s, 3H), 2.04 (s, 3H), 1.83 (s, 3H), 1.32–1.22 (m, 6H), 0.83 (d, J = 6.3 Hz, 3H), 0.70 (d, J = 6.2 Hz, 3H); ^{13}C NMR (125 MHz, $CDCl_3$) δ 170.80, 169.96, 169.83, 168.47, 167.75, 165.90, 165.87, 165.71, 165.54, 165.38, 165.15, 165.07, 164.25, 134.17, 133.81, 133.79, 133.58, 133.52, 133.18, 133.16, 133.05, 133.02, 131.51, 130.08, 130.04, 129.99, 129.97, 129.93, 129.80, 129.65, 129.57, 129.54, 129.53, 129.47, 129.42, 129.41, 129.35, 128.99, 128.91, 128.85, 128.71, 128.51, 128.48, 128.45, 128.37, 128.34, 124.04, 101.37, 99.12, 98.91, 98.23, 90.48, 76.52, 76.08, 75.51, 73.80, 72.57, 72.52, 71.88, 71.72, 71.42, 71.04, 70.52, 70.28, 69.53, 69.22, 68.90, 68.41, 68.17, 67.42, 61.77, 54.66, 21.12, 20.92, 20.87, 20.68, 17.78, 17.65, 17.49, 17.28; HRMS (ESI) calcd. for $C_{102}H_{95}NO_{35}Na$ $(M + Na)^+$ m/z 1916.5577, found 1916.5582.

To a stirred solution of **S28** (23.1 mg, 0.0122 mmol) in dry CH_2Cl_2 (1.0 mL) was added 33% HBr in HOAc (0.2 mL) dropwise at 0 ℃. The mixture was stirred at room temperature and was monitored by TLC. After completion (1 h), the mixture was diluted with CH_2Cl_2 (20 mL) and was washed with ice-water (2×5 mL), $NaHCO_3$ (2×5 mL), and brine (2×5 mL), successively. The organic layer was dried over Na_2SO_4 and filtered. The filtrate was concentrated under reduced pressure to give **S29** as a white solid, which was used directly for the following coupling reaction.

3-*O*-(2,3,4-Tri-*O*-acetyl-α-L-fucopyranosyl)-4-*O*-(2,3,4,6-tetra-*O*-acetyl-β-D-galactopyranosyl)-6-*O*-benzoyl-2-deoxy-2-phthalimido-β-D-glucopyranosyl bromide (S33)

Compound **S33** was prepared employing a literature procedure[11].

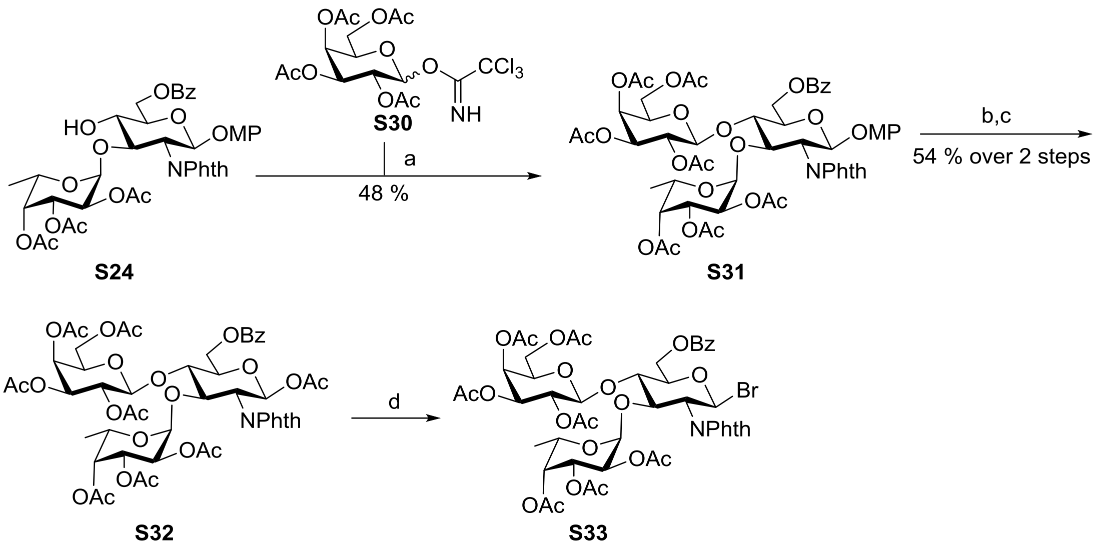

Supplementary Figure 12. Preparation of Lewis[x] trisaccharide bromide **S33**. Reagents and conditions: a) TMSOTf (20 mol%), CH_2Cl_2/Et_2O (1:5), 4 Å MS, -40 °C→25 °C, 8 h, 48 %; b) CAN (3.0 equiv.), CH_3CN/H_2O (4:1), 25 °C; 1.5 h; c) Ac_2O (15.0 equiv.), pyridine, 25 °C, overnight; d) 33% HBr in HOAc, CH_2Cl_2, 0 °C→25 °C, 2 h, quantitative.

To a stirred solution of 2,3,4,6-tetra-*O*-acetyl-D-galactopyranose (1.67 g, 5 mmol) in CH_2Cl_2 (20 mL) and Cl_3CCN (6 mL, 60 mmol) was added K_2CO_3 (3.5 g, 25 mmol). The mixture was stirred at room temperature for 6 h. After filtration, the mixture was concentrated and azeotroped with toluene (2×20 mL) to give trichloroacetimidate 2,3,4,6-tetra-*O*-acetyl-D-galactopyranoside **S30**, which was used directly in the next step.

Compound **S24** (529 mg, 0.67 mmol, 1.0 equiv.), **S30** (3.88 mmol, 5.8 equiv.), and 4 Å MS (1 g) were suspended in dry CH_2Cl_2 (3 mL) and Et_2O (15 mL). The mixture was stirred under -40 °C for 0.5 h, after which TMSOTf (19 μL, 20 mol %) was injected. After stirring at -40 °C for 0.5 h, the reaction was allowed to warm to 0 °C slowly and stirred for 4 h at 0 °C. The reaction was quenched with Et_3N (0.5 mL). After filtration, the mixture was concentrated and purified by column chromatography (petroleum ether/ethyl acetate/CH_2Cl_2 = 2:1:0.5 to 2:1:1.5) to give recovered **S30** (180 mg, 34%) and *p*-methoxyphenyl 3-*O*-(2,3,4-tri-*O*-acetyl-α-L-fucopyranosyl)-4-*O*-(2,3,4,6-tetra-*O*-acetyl-β-D-galactopyranosyl)-6-*O*-benzoyl-2-deoxy-2-phthalimido-β-D-glucopyranoside **S31** (360 mg, 48%): ^{1}H NMR (500 MHz, $CDCl_3$) δ 8.07 (d, *J* = 7.9 Hz, 2H), 7.85 (dd, *J* = 5.4, 3.1 Hz, 2H), 7.77 (dd, *J* = 5.5, 3.1 Hz, 2H), 7.64 (t, *J* = 7.4 Hz, 1H), 7.53 (t, *J* = 7.7 Hz, 2H), 6.72 (d, *J* = 9.1 Hz, 2H), 6.57 (d, *J* = 9.0 Hz, 2H), 5.53 (d, *J* = 8.6 Hz, 1H), 5.40 (d, *J* = 3.5 Hz, 1H), 5.39 (d, *J* = 3.5 Hz, 1H), 5.17 (dd, *J* = 11.0, 3.4 Hz, 1H), 5.15 (dd, *J* = 10.4, 8.0 Hz, 1H), 5.03-4.95 (m, 3H), 4.95 (dd, *J* = 10.3, 3.5 Hz, 1H), 4.85 (dd, *J* = 10.5, 9.0 Hz, 1H), 4.83 (dd, *J* = 10.9, 4.1 Hz, 1H), 4.66 (d, *J* = 8.2 Hz, 1H), 4.58 (dd, *J* = 11.6, 6.7 Hz, 1H), 4.54 (dd, *J* = 10.8, 8.8 Hz, 1H), 4.39 (dd, *J* = 11.8, 5.6 Hz, 1H), 4.31 (dd, *J* = 11.5, 7.5 Hz, 1H), 4.11 (t, *J* = 9.4 Hz, 1H),

3.93 (ddd, J = 10.0, 5.7, 1.9 Hz, 1H), 3.83 (t, J = 7.0 Hz, 1H), 3.66 (m, 3H), 2.19 (s, 3H), 2.14 (s, 3H), 2.11 (s, 3H), 2.09 (s, 3H), 2.07 (s, 3H), 1.97 (s, 3H), 1.93 (s, 3H), 1.23 (d, J = 6.5 Hz, 3H); ^{13}C NMR (125 MHz, $CDCl_3$) δ 170.84, 170.60, 170.59, 170.09, 169.89, 169.22, 165.79, 155.69, 150.70, 134.62, 133.69, 129.84, 129.53, 128.84, 123.86, 118.82, 114.45, 100.85, 97.86, 95.39, 75.82, 73.53, 71.47, 71.33, 71.30, 71.19, 68.99, 68.26, 67.87, 66.80, 64.44, 62.42, 60.82, 56.35, 55.63, 20.94, 20.94, 20.85, 20.81, 20.78, 20.68, 20.65, 16.01; HRMS (ESI) calcd. for $C_{54}H_{59}NO_{25}Na$ $(M + Na)^+$ m/z 1144.3268, found 1144.3254.

To a stirred solution of **S31** (360 mg, 0.321 mmol) in CH_3CN/H_2O (4:1, v/v, 3.2 mL) was added diammonium cerium (IV) nitrate (528 mg, 3.0 equiv.). After stirring at room temperature for 1.5 h, the mixture was diluted with EtOAc (50 mL) and washed with water (3×15 mL) and brine (3×15 mL) successively. The organic layer was dried over Na_2SO_4 and was concentrated. A short silica-gel column chromatography was used to remove the impurity with high mobility and insoluble impurities (petroleum ether/ethyl acetate = 2:1 to 1:1) to provide the crude product (273 mg, 83%). The crude product was dissolved in pyridine (3 mL), and Ac_2O (0.38 mL, 15.0 equiv.) was added. The mixture was stirred at room temperature overnight, after which methanol (0.5 mL) was added. The mixture was stirred for another one hour. After removal of solvent, the mixture was purified by column chromatography (petroleum ether/ethyl acetate = 1:1) to give 3-*O*-(2,3,4-tri-*O*-acetyl-α-L-fucopyranosyl)-4-*O*-(2,3,4,6-tetra-*O*-acetyl-β-D-galactopyranosyl)-6-*O*-benzoyl-2-deoxy-2-phthalimido-β-D-glucopyranosyl acetate **S32** (185 mg, 54% over 2 steps): ^{1}H NMR (500 MHz, $CDCl_3$) δ 8.09 (d, J = 7.3 Hz, 2H), 7.86 (dd, J = 5.5, 3.1 Hz, 2H), 7.79 (dd, J = 5.5, 3.1 Hz, 2H), 7.69–7.61 (m, 1H), 7.56 (t, J = 7.6 Hz, 2H), 6.25 (d, J = 8.8 Hz, 1H), 5.37 (d, J = 3.5 Hz, 2H), 5.19–5.09 (m, 2H), 4.98 (q, J = 7.0 Hz, 1H), 4.95–4.89 (m, 3H), 4.87 (dd, J = 10.3, 3.5 Hz, 1H), 4.81 (dd, J = 10.9, 4.1 Hz, 1H), 4.67 (d, J = 8.1 Hz, 1H), 4.56 (dd, J = 11.3, 6.2 Hz, 1H), 4.44 (dd, J = 10.4, 8.9 Hz, 1H), 4.37 (dd, J = 12.3, 3.6 Hz, 1H), 4.28 (dd, J = 11.6, 7.8 Hz, H), 4.16 (t, J = 9.4 Hz, 1H), 3.95 (ddd, J = 10.2, 3.9, 2.1 Hz, 1H), 3.71 (t, J = 7.0 Hz, 1H), 2.18 (s, 3H), 2.13 (s, 3H), 2.09 (s, 3H), 2.08 (s, 3H), 2.07 (s, 3H), 1.96 (s, 3H), 1.93 (s, 3H), 1.91 (s, 3H), 1.23 (d, J = 6.5 Hz, 3H); ^{13}C NMR (125 MHz, $CDCl_3$) δ 170.78, 170.71, 170.53, 170.43, 170.05, 169.84, 169.06, 168.85, 165.70, 134.71, 133.77, 131.23, 129.75, 129.40, 128.93, 123.94, 100.50, 95.31, 90.11, 77.16, 74.73, 73.86, 71.40, 71.29, 71.01, 68.86, 68.12, 67.88, 66.70, 64.45, 61.95, 60.69, 55.36, 20.89, 20.87, 20.81, 20.79, 20.79, 20.74, 20.65, 20.62, 15.97; HRMS (ESI) calcd. for $C_{49}H_{55}NO_{25}Na$ $(M + Na)^+$ m/z 1080.2955, found 1080.2944.

To a stirred solution of **S32** (185 mg, 0.175 mmol) in dry CH_2Cl_2 (1.0 mL) was added 33% HBr in HOAc (0.2 mL) dropwise under 0 ℃. The mixture was warmed to room temperature and was stirred for 1.5 h. After dilution with CH_2Cl_2 (50 mL), the mixture was washed with ice-water (3×20 mL), sat. aq. $NaHCO_3$ (3×10 mL), and brine (2×20 mL), successively. The organic layer was dried over Na_2SO_4, filtered, and concentrated to give **S33**, which was pure enough and was used directly in the coupling reaction. **S33**: ^{1}H NMR (500 MHz, $CDCl_3$) δ 8.10 (d, J = 7.9 Hz, 2H), 7.91–7.85 (m,

2H), 7.83–7.77 (m, 2H), 7.66 (t, *J* = 7.4 Hz, 1H), 7.56 (t, *J* = 7.1 Hz, 2H), 6.19 (d, *J* = 10.1 Hz, 1H), 5.37 (d, *J* = 3.5 Hz, 2H), 5.14 (dd, *J* = 7.7, 2.9 Hz, 1H), 5.12 (dd, *J* = 11.34, 7.38 Hz, 1H), 4.98 (d, *J* = 2.7 Hz, 1H), 4.96 (s, 1H), 4.91 (d, *J* = 2.9 Hz, 1H), 4.88 (d, *J* = 10.3, 3.4 Hz, 1H), 4.83 (dd, *J* = 10.9, 4.1 Hz, 1H), 4.76 (t, *J* = 9.6 Hz, 1H), 4.67 (d, *J* = 8.2 Hz, 1H), 4.62 (t, *J* = 9.8 Hz, 1H), 4.55 (dd, *J* = 11.1, 6.1 Hz, 1H), 4.38 (dd, *J* = 12.5, 3.8 Hz, 1H), 4.29 (dd, *J* = 11.2, 7.4 Hz, 1H), 4.20 (t, *J* = 9.5 Hz, 1H), 3.89 (dd, *J* = 10.0, 2.1 Hz, 1H), 3.73 (t, *J* = 7.1 Hz, 1H), 2.18 (s, 3H), 2.13 (s, 3H), 2.10 (s, 3H), 2.09 (s, 3H), 2.07 (s, 3H), 1.96 (s, 3H), 1.92 (s, 3H), 1.22 (d, *J* = 6.5 Hz, 3H); ^{13}C NMR (125 MHz, $CDCl_3$) δ 170.74, 170.72, 170.50, 170.41, 169.99, 169.78, 169.05, 165.63, 134.77, 133.80, 129.75, 129.25, 128.91, 124.00, 100.54, 95.41, 78.47, 78.19, 74.64, 71.77, 71.35, 71.32, 71.11, 68.85, 68.09, 67.66, 66.66, 64.50, 61.91, 60.69, 59.69, 59.50, 20.90, 20.87, 20.78, 20.77, 20.70, 20.60, 20.58, 15.94.

2.2 Preparation of alkyne derivatives of amino acids and peptides

Amino acids

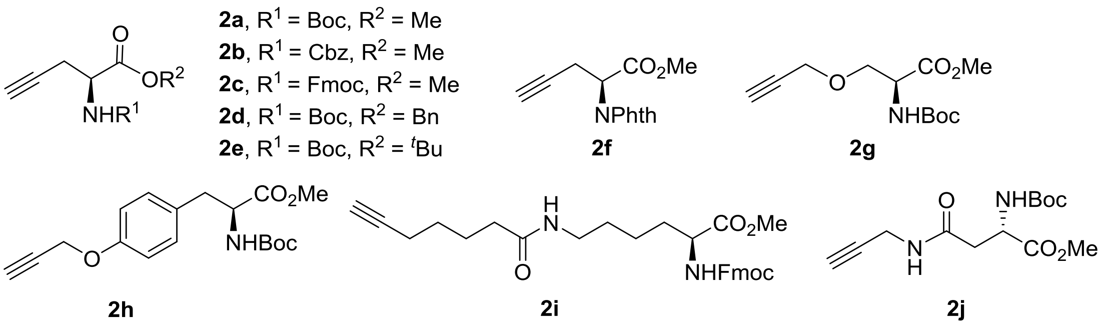

Peptides

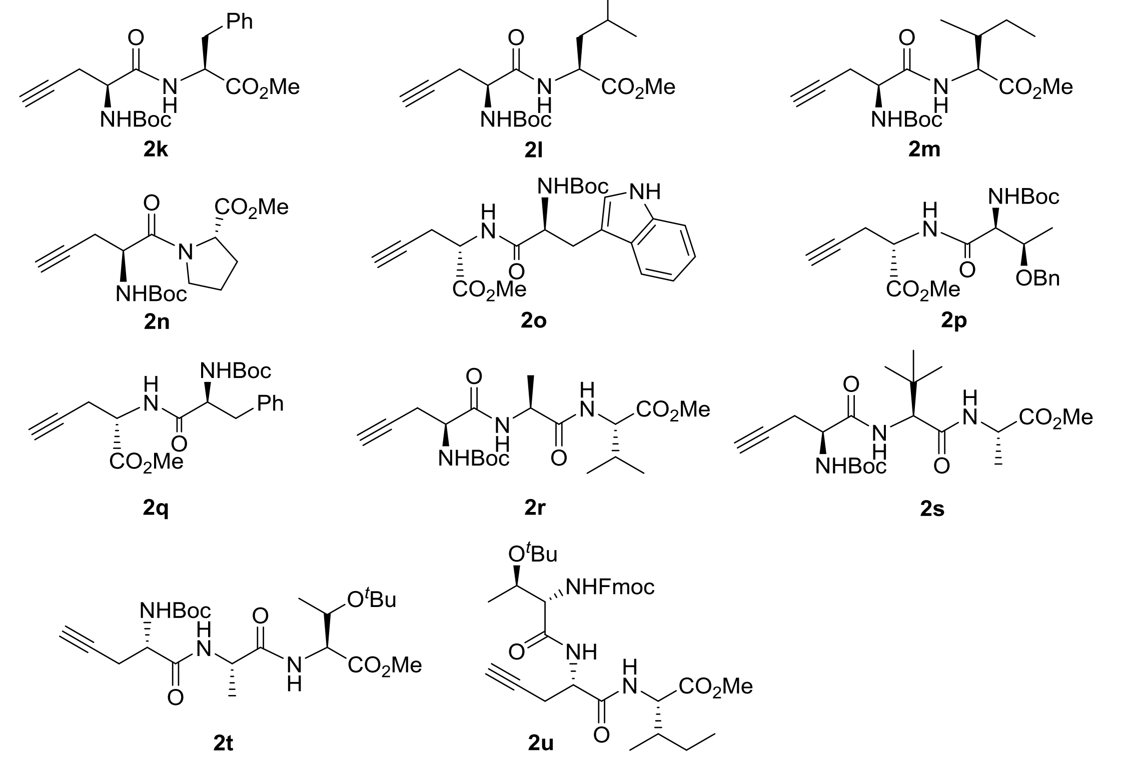

Internal acetylenic amino acid

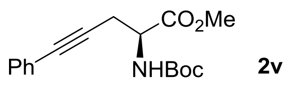

Supplementary Figure 13. Acetylenic amino acid and peptide derivatives used in this study.

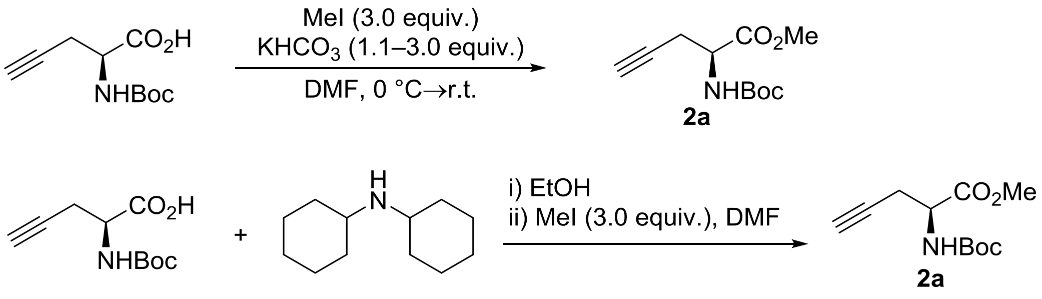

Supplementary Figure 14. General procedures for methylation of amino acids.

Procedure A: To a stirred solution of Boc-L-propargylglycine (2.1 g, 10 mmol) in anhydrous DMF (15 mL) was added $KHCO_3$ (1.1 equiv.) at 0 °C. After stirring for 0.5 h at 0 °C, MeI (3.0 equiv.) was injected, and the mixture was stirred at room temperature overnight. After dilution with ethyl acetate (100 mL), the mixture was washed with water (3×30 mL), sat. aq. $NaHCO_3$ (3×20 mL), and brine, successively. The organic layer was dried over $MgSO_4$ and concentrated to afford **2a** (2.0 g, 88%), which was pure enough and used without further purification.

Procedure B: To a stirred solution of Boc-L-propargylglycine (2.1 g, 10 mmol) in anhydrous EtOH (30 mL) was added dicyclohexylamine (1.0 equiv.). After stirring at room temperature for 0.5 h, the solvent was removed *in vacuo*, and the crude product was dried over oil pump. The obtained white solid was suspended in dry DMF (50 mL), and MeI (3.2 equiv.) was added. The reaction mixture was stirred at room temperature for 30 h. After dilution with ethyl acetate (200 mL), the mixture was washed with water (3×50 mL), sat. aq. $NaHCO_3$ (3×50 mL), 1M aq. HCl (3×30 mL), and brine, successively. The organic layer was dried over $MgSO_4$ and concentrated to afford product **2a** (1.9 g, 83%), which was pure enough and was used without further purification.

***N-Tert*-butoxycarbonyl-L-propargylglycine methyl ester (2a)**[12]

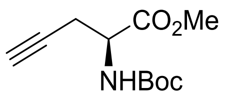

^{1}H NMR (500 MHz, $CDCl_3$) δ 5.36 (d, J = 8.6 Hz, 1H), 4.48 (dt, J = 9.3, 4.9 Hz, 1H), 3.78 (s, 3H), 2.83–2.61 (m, 2H), 2.05 (t, J = 2.7 Hz, 1H), 1.46 (s, 9H).

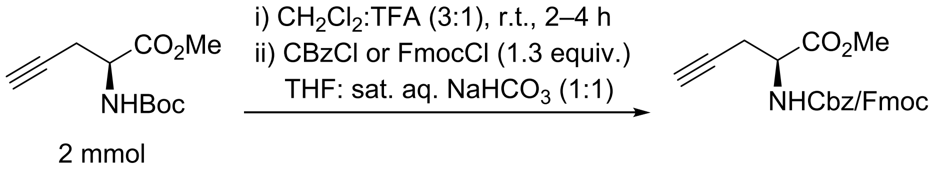

Supplementary Figure 15. General procedure for Cbz/Fmoc protection of amino acids.

To a stirred solution of methyl Boc-L-propargylglycinate (454.5 mg, 2 mmol) in dry CH_2Cl_2 (4.5 mL) was added TFA (1.5 mL) slowly at 0 °C. The reaction mixture was stirred for 2–4 h (monitored by TLC) at room temperature to remove the Boc protecting

group. After completion, the reaction solution was concentrated and dried over oil pump. To a stirred solution of the crude product in THF (10 mL) was added sat. aq. $NaHCO_3$ (10 mL). After stirring for 0.5 h, CbzCl or FmocCl (1.3 equiv.) was added. After stirring for 4 h, the mixture was diluted with ethyl acetate. The organic phase was washed with water, 1 N aq. HCl, and brine, successively. After dried over anhydrous $MgSO_4$ and concentrated, the pure product was obtained by column chromatography.

***N*-Benzyloxycarbonyl-L-propargylglycine methyl ester** (**2b**)[13]

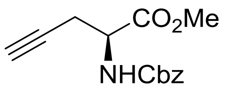

381 mg, 73%. ^{1}H NMR (500 MHz, $CDCl_3$) δ 7.38–7.31 (m, 5H), 5.62 (d, J = 8.4 Hz, 1H), 5.13 (s, 2H), 4.55 (dt, J = 8.9, 4.8 Hz, 1H), 3.79 (s, 3H), 2.78 (dt, J = 5.2, 2.6 Hz, 2H), 2.04 (t, J = 2.6 Hz, 1H).

***N*-(9-Fluorenylmethoxycarbonyl)-L-propargylglycine methyl ester** (**2c**)[14]

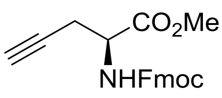

572 mg, 82%. ^{1}H NMR (500 MHz, $CDCl_3$) δ 7.77 (d, J = 7.5 Hz, 2H), 7.62 (d, J = 6.8 Hz, 3H), 7.41 (t, J = 7.4 Hz, 3H), 7.33 (t, J = 7.5 Hz, 4H), 5.66 (d, J = 8.3 Hz, 1H), 4.56 (dt, J = 9.0, 4.8 Hz, 1H), 4.41 (d, J = 7.2 Hz, 2H), 4.25 (t, J = 7.3 Hz, 1H), 3.81 (s, 3H), 2.80 (dd, J = 4.8, 2.6 Hz, 2H), 2.08 (t, J = 2.6 Hz, 1H).

***N*-*Tert*-butoxycarbonyl-L-propargylglycine benzyl ester (2d)**[15]

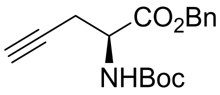

Prepared by a similar procedure as **2a** [Boc-L-propargylglycine (0.8 g, 3.75 mmol), BnBr (0.9 equiv.), $KHCO_3$ (3.0 equiv.), DMF (5 mL), 1 h; 1.12 g, 97%]. ^{1}H NMR (500 MHz, $CDCl_3$) δ 7.38–7.31 (m, 5H), 5.36 (d, J = 8.5 Hz, 1H), 5.21 (q, J = 12.3 Hz, 2H), 4.52 (dt, J = 9.1, 4.8 Hz, 1H), 2.79 (ddd, J = 17.0, 4.6, 2.6 Hz, 1H), 2.72 (ddd, J = 17.0, 5.2, 2.7 Hz, 1H), 2.01 (t, J = 2.6 Hz, 1H), 1.45 (s, 9H).

***N*-*Tert*-butoxycarbonyl-L-propargylglycine *tert*-butyl ester** (**2e**)[16]

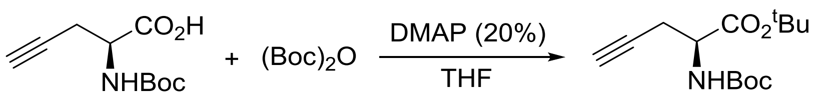

2e

Supplementary Figure 16. Preparation of acetylenic amino acid **2e**.

To a stirred solution of Boc-L-propargylglycine (530 mg, 2.5 mmol) and $(Boc)_2O$ (1.4 equiv.) in THF (25 mL) was added DMAP (0.2 equiv.) slowly. The reaction mixture was stirred for 5 h at room temperature. After concentration, the pure product **2e** was obtained by column chromatography (280 mg, 42%): ^{1}H NMR (500 MHz, $CDCl_3$) δ 5.34 (d, J = 7.8 Hz, 1H), 4.34 (dt, J = 8.7, 4.6 Hz, 1H), 2.70 (dd, J = 7.6, 3.7 Hz, 2H), 2.02 (t, J = 2.6 Hz, 1H), 1.49 (s, 9H), 1.45 (s, 9H).

***N*,*N*-Phthaloyl-L-propargylglycine methyl ester (2f)**[17]

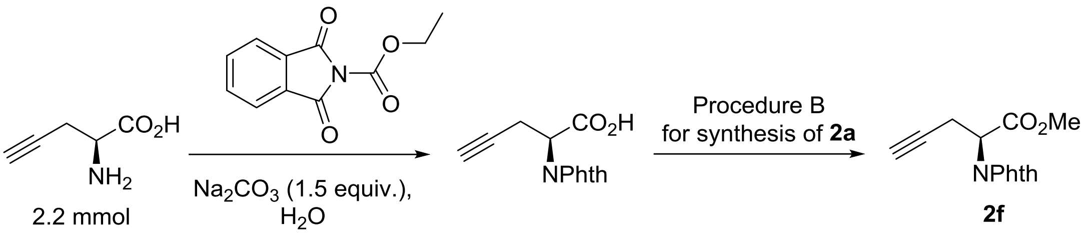

Supplementary Figure 17. Preparation of acetylenic amino acid **2f**.

To a stirred suspension of L-propargylglycine (250 mg, 2.2 mmol) in distilled water (3 ml) was added Na_2CO_3 (1.5 equiv.). After stirring for 10 mins, *N*-ethoxycarbonylphthalimide (1.5 equiv.) was added, and the reaction was continued until the solution became clear. The reaction mixture was cooled to 0 ℃ and aq. HCl (1 N, 15 mL) was added to acidify the solution. After extraction with ethyl acetate, the organic phase was washed with water and brine, and was then concentrated. The crude product was subjected directly to the Procedure B (as for the preparation of **2a**) to provide **2f** (384 mg, 68% yield over two steps): ^{1}H NMR (500 MHz, $CDCl_3$) δ 7.93–7.87 (m, 2H), 7.82–7.71 (m, 2H), 5.09 (dd, J = 11.2, 4.9 Hz, 1H), 3.76 (s, 3H), 3.31–3.19 (m, 1H), 3.17–3.02 (m, 1H), 1.91 (s, 1H).

***N*-(*Tert*-butoxycarbonyl)-*O*-(prop-2-yn-1-yl)-L-serine methyl ester (2g)**[18,19]

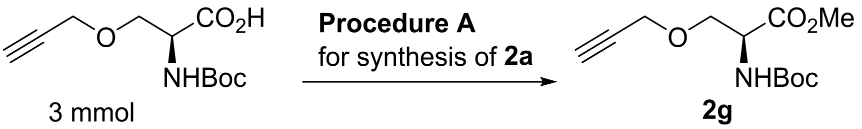

Supplementary Figure 18. Preparation of acetylenic amino acid **2g**.

Compound **2g** was prepared from *N*-(*tert*-butoxycarbonyl)-*O*-(prop-2-yn-1-yl)-L-serine (0.72 g, 3 mmol) using Procedure A (as for the preparation of **2a**) (640 mg, 83%): ^{1}H NMR (500 MHz, $CDCl_3$) δ 5.38 (d, J = 8.8 Hz, 1H), 4.53–4.37 (m, 1H), 4.15 (dd, J = 2.4, 0.6 Hz, 2H), 3.96 (dd, J = 9.3, 3.2 Hz, 1H), 3.81–3.72 (m, 4H), 2.46 (td, J = 2.4, 0.6 Hz, 1H), 1.46 (s, 9H).

***N*-(*Tert*-butoxycarbonyl)-*O*-(prop-2-yn-1-yl)-L-tyrosine methyl ester (2h)**[20]

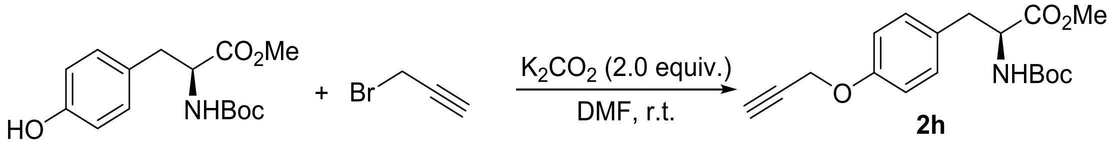

Supplementary Figure 19. Preparation of acetylenic amino acid **2h**.

A mixture of Boc-L-Tyr-OMe (1.47 g, 5 mmol), propargyl bromide (1.1 mL, 1.3 mmol), and K_2CO_3 (2.0 equiv.) in DMF (10 mL) was stirred at room temperature overnight. The mixture was diluted with ethyl acetate. The organic phase was washed with 0.5 M HCl, water, and brine. After dried over $MgSO_4$, the solution was concentrated *in vacuo*

to give **2h** as a viscous oil (1.25 g, 75%): ^{1}H NMR (500 MHz, $CDCl_3$) δ 7.05 (d, J = 8.5 Hz, 2H), 6.90 (d, J = 8.6 Hz, 2H), 4.96 (d, J = 8.3 Hz, 1H), 4.67 (d, J = 2.4 Hz, 2H), 4.54 (q, J = 6.7 Hz, 1H), 3.71 (s, 1H), 3.03 (qd, J = 14.0, 5.9 Hz, 2H), 2.51 (t, J = 2.4 Hz, 1H), 1.42 (s, 9H).

N^2-(((9H-Fluoren-9-yl) methoxy) carbonyl)-N^6-(hept-6-ynoyl)-L-lysine methyl ester (2i)

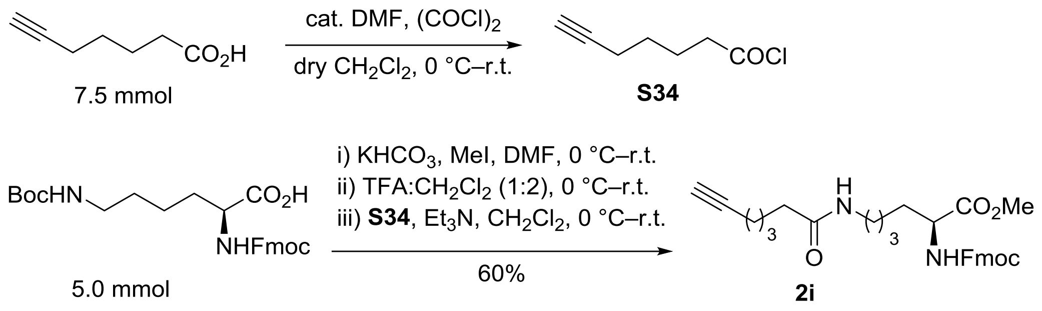

Supplementary Figure 20. Preparation of acetylenic amino acid **2i**.

To a stirred solution of 6-heptynoic acid (946 mg, 7.5 mmol) in extra dry CH_2Cl_2 (15 mL) was added catalytic amount of DMF (0.05–0.1 mL), followed by addition of $(COCl)_2$ (1.27 mL, 2.0 equiv.) dropwise under 0 ℃ (**Caution**: The mixture bubbled vigorously!). The mixture was stirred vigorously for 0.5 h under 0 ℃ and 0.5 h at room temperature. The solvent and residue oxalyl chloride was removed *in vacuo* to give the crude acyl chloride **S34**, which was used directly in the next step.

To a stirred solution of *N*-Fmoc-*N*'-Boc-L-Lys (2.3 g, 5.0 mmol) in dry DMF (10 mL) was added $KHCO_3$ (1.5 g, 3.0 equiv.) at 0 ℃. After vigorously stirring for 20 mins, MeI (0.65 mL, 2.0 equiv.) was added. The mixture was stirred for 7 h at room temperature. After dilution with ethyl acetate (50 mL), the mixture was washed with water and brine and was then dried over anhydrous $MgSO_4$. After concentration, the crude product was dissolved in dry CH_2Cl_2 (10 mL). TFA (5 mL) was added slowly under 0 ℃, and the reaction mixture was stirred for 2 h at room temperature. The solvent was removed *in vacuo* and the residue was dried over oil pump. To a stirred solution of the above residue in dry CH_2Cl_2 (10 mL) was added Et_3N (2.1 mL, 3.0 equiv.) at 0 ℃. The crude acyl chloride **S34** was dissolved in extra dry CH_2Cl_2 (2 mL) and was injected to this reaction mixture. After stirring for 5 h at room temperature, the reaction mixture was diluted with CH_2Cl_2 (50 mL) and washed with 1 M aq. HCl (2×20 mL), sat. aq. $NaHCO_3$ (2×20 mL), and brine, successively. After concentration, the residue was purified by column chromatography (petroleum ether/ethyl acetate = 2:1 to 1:1) to yield **2i** (1.5 g, 60%): ^{1}H NMR (500 MHz, $CDCl_3$) δ 7.76 (d, J = 7.5 Hz, 2H), 7.60 (t, J = 6.4 Hz, 2H), 7.40 (t, J = 7.4 Hz, 2H), 7.31 (t, J = 7.4 Hz, 2H), 5.67 (t, J = 5.7 Hz, 1H), 5.51 (d, J = 8.2 Hz, 1H), 4.46–4.33 (m, 3H), 4.22 (t, J = 7.1 Hz, 1H), 3.75 (s, 3H), 3.24 (q, J = 6.7 Hz, 2H), 2.23–2.11 (m, 4H), 1.94 (s, 1H), 1.89–1.81 (m, 1H), 1.77–1.65 (m, 3H), 1.57–1.48 (m, 4H), 1.43–1.31 (m, 2H); ^{13}C NMR (125 MHz, $CDCl_3$) δ 173.00, 172.89, 156.19, 143.96, 143.78, 141.39, 127.84, 127.17, 125.16, 120.11, 84.19, 68.75,

67.13, 53.68, 52.57, 47.25, 39.05, 36.18, 32.29, 29.07, 28.02, 24.88, 22.53, 18.26; HRMS (ESI) calcd. for $C_{29}H_{34}N_2O_5Na$ (M + Na)$^+$ m/z 513.2360, found 513.2362.

Methyl *N*^{2}-(*tert*-butoxycarbonyl)-*N*^{4}-(prop-2-yn-1-yl)-L-asparaginate (2j)[21]

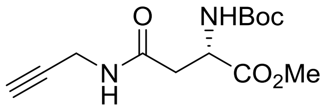

^{1}H NMR (500 MHz, $CDCl_3$) δ 5.93 (s, 1H), 5.70 (d, *J* = 7.5 Hz, 1H), 4.57–4.48 (m, 1H), 4.07–4.01 (m, 2H), 3.76 (d, *J* = 4.2 Hz, 3H), 2.92 (dd, *J* = 15.6, 4.2 Hz, 1H), 2.73 (dd, *J* = 15.8, 4.4 Hz, 1H), 2.34–2.17 (m, 1H), 1.44 (d, *J* = 4.7 Hz, 9H).

General Procedure for the preparation of peptides

General Procedure I: preparation of dipeptides

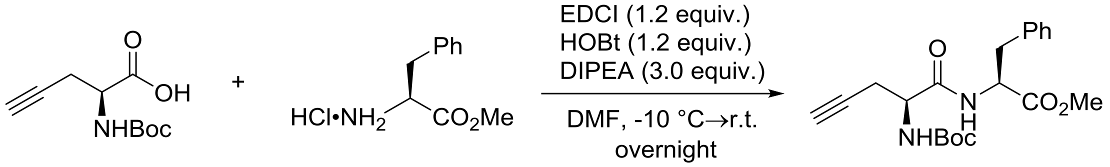

2k

Supplementary Figure 21. General Procedure I for the synthesis of dipeptides (**2k** as an example).

To a stirred suspension of Boc-L-propargylglycine (1.06 g, 5.0 mmol, 1.0 equiv.), methyl L-phenylalaninate hydrochloride (6 mmol, 1.2 equiv.), and HOBt (1.2 equiv.) in dry DMF (15 mL) was added DIPEA (2.61 mL, 3.0 eq) under -10 ℃. After which, EDCI (1.2 equiv.) was added. The reaction mixture was stirred under -10 ℃ for 0.5 h. Then the mixture was warmed to room temperature and stirred overnight. After dilution with ethyl acetate, the organic phase was washed with water, 1M aq. HCl, and brine thrice. The organic phase was dried over anhydrous $MgSO_4$, and then concentrated to provide the crude product. The pure product was obtained by column chromatography.

***N*-(*Tert*-butoxycarbonyl)-L-*C*-propargylglycyl-L-phenylalanine methyl ester (2k)**

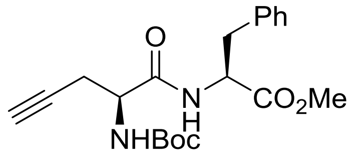

1.47 g, 79%. ^{1}H NMR (500 MHz, $CDCl_3$) δ 7.30–7.23 (m, 3H), 7.14–7.10 (m, 2H), 6.74 (d, *J* = 7.5 Hz, 1H), 5.20 (s, 1H), 4.86 (dt, *J* = 7.6, 5.7 Hz, 1H), 4.27 (s, 1H), 3.71 (s, 3H), 3.21–3.06 (m, 2H), 2.79 (d, *J* = 17.0 Hz, 1H), 2.56 (ddd, *J* = 16.9, 6.7, 2.7 Hz, 1H), 2.02 (t, *J* = 2.7 Hz, 1H), 1.44 (s, 9H); ^{13}C NMR (125 MHz, $CDCl_3$) δ 171.48, 169.90, 155.29, 135.74, 129.37, 128.59, 127.16, 80.50, 79.41, 71.79, 53.48, 52.81, 52.37, 37.89, 28.29, 22.31; HRMS (ESI) calcd. for $C_{20}H_{26}N_2O_5Na$ (M + Na)$^+$ m/z 397.1734, found 397.1737.

***N*-(*Tert*-butoxycarbonyl)-L-*C*-propargylglycyl-L-leucine methlyl ester (2l)**

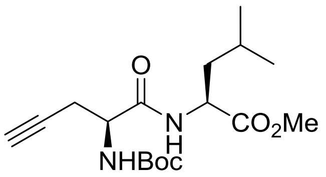

1.3 g, 78%. ^{1}H NMR (500 MHz, $CDCl_3$) δ 6.70 (s, 1H), 5.25 (s, 1H), 4.63 (td, J = 8.7, 4.7 Hz, 1H), 4.29 (s, 1H), 3.73 (s, 1H), 2.82 (ddd, J = 16.9, 5.4, 2.7 Hz, 1H), 2.60 (ddd, J = 16.9, 6.5, 2.4 Hz, 1H), 2.08 (t, J = 2.6 Hz, 1H), 1.71–1.61 (m, 1H), 1.61–1.52 (m, 1H), 1.47 (s, 1H), 0.93 (dd, J = 6.0, 5.0 Hz, 1H); ^{13}C NMR (125 MHz, $CDCl_3$) δ 173.05, 170.03, 80.77, 79.53, 71.77, 52.95, 52.46, 51.01, 41.79, 28.38, 24.86, 22.94, 22.23, 22.04; HRMS (ESI) calcd. for $C_{17}H_{28}N_2O_5Na$ $(M + Na)^+$ m/z 363.1890, found 363.1892.

***N*-(*Tert*-butoxycarbonyl)-L-*C*-propargylglycyl-L-isoleucine methyl ester (2m)**

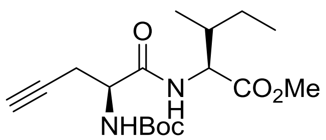

1.41 g, 83%. ^{1}H NMR (500 MHz, $CDCl_3$) δ 6.89 (d, J = 8.6 Hz, 1H), 5.32 (d, J = 7.2 Hz, 1H), 4.59 (dd, J = 8.6, 4.9 Hz, 1H), 4.31 (d, J = 7.2 Hz, 1H), 3.74 (s, 3H), 2.83 (ddd, J = 16.9, 5.4, 2.6 Hz, 1H), 2.60 (ddd, J = 16.9, 6.7, 2.7 Hz, 1H), 2.10 (t, J = 2.6 Hz, 1H), 1.91 (dqd, J = 9.4, 4.8, 2.5 Hz, 1H), 1.47 (s, 9H), 1.47–1.39 (m, 1H), 1.18 (ddq, J = 14.2, 9.0, 7.3 Hz, 1H), 0.92 (dd, J = 8.5, 7.0 Hz, 6H); ^{13}C NMR (125 MHz, $CDCl_3$) δ 171.97, 170.04, 155.43, 80.63, 79.54, 71.75, 56.72, 52.90, 52.19, 38.02, 28.33, 25.14, 22.14, 15.47, 11.62, 11.62; HRMS (ESI) calcd. for $C_{17}H_{28}N_2O_5Na$ $(M + Na)^+$ m/z 363.1890, found 363.1894.

***N*-(*Tert*-butoxycarbonyl)-L-*C*-propargylglycyl-L-proline methyl ester (2n)**

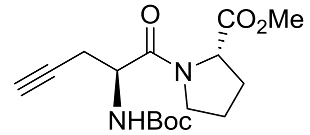

1.29 g, 80%, occurring as a mixture of rotamers. ^{1}H NMR (500 MHz, $CDCl_3$) δ 5.33 (dd, J = 24.2, 9.0 Hz, 1H), 4.85–4.62 (m, 1H), 4.58–4.51 (m, 1H), 3.79–3.71 (m, 5H), 2.70–2065 (m, 1H), 2.61–2.54 (m, 1H), 2.26–2.20 (m, 1H), 2.12–1.97 (m, 4H), 1.44 & 1.42 (s, 9H). The major rotamer: ^{13}C NMR (125 MHz, $CDCl_3$) δ 172.22, 169.49, 155.27, 80.07, 78.99, 71.00, 59.01, 52.35, 50.59, 47.21, 29.08, 28.41, 24.95, 22.99; HRMS (ESI) calcd. for $C_{16}H_{24}N_2O_5Na$ $(M + Na)^+$ m/z 347.1577, found 347.1579.

***O*-Benzyl-*N*-(*tert*-butoxycarbonyl)-L-threonyl-L-*C*-propargylglycine methyl ester (2p)**

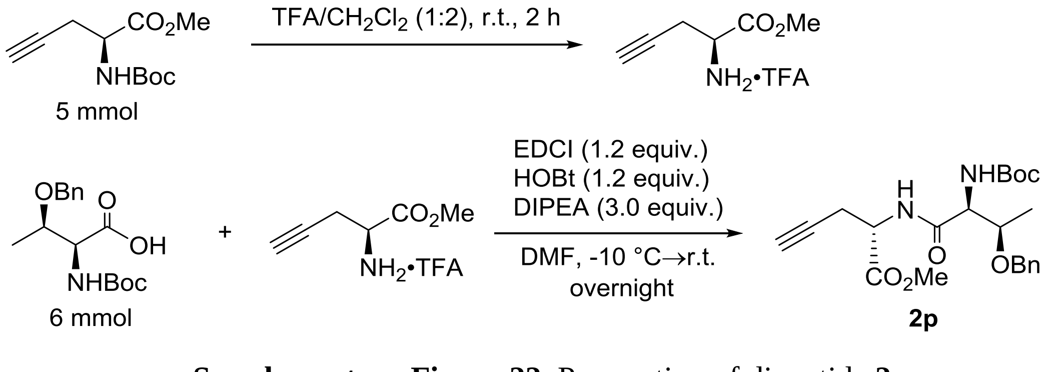

Supplementary Figure 22. Preparation of dipeptide **2p**.

To a stirred solution of methyl L-Boc-*C*-propargylglycinate (650 mg, 2.86 mmol) in dry CH_2Cl_2 (6 mL) was added TFA (2 mL) slowly at 0 ℃. The mixture was stirred at room temperature to remove the Boc group. After completion (monitored by TLC, 2–4 h), the reaction solution was concentrated and dried *in vacuo*.

To a stirred solution of the above crude product, *O*-benzyl-*N*-(*tert*-butoxycarbonyl)-L-threonine (1.06 g, 1.2 equiv.), and HOBt (1.2 equiv.) in dry DMF (10 mL) was added DIPEA (1.5 mL, 3.0 equiv.) under -10 ℃. After which, EDCI (1.2 equiv.) was added. The reaction mixture was stirred under -10 ℃ for 0.5 h. Then the mixture was warmed to room temperature and stirred overnight. After dilution with ethyl acetate, the organic phase was washed with water, and 1M aq. HCl, and brine thrice. The organic phase was dried over anhydrous $MgSO_4$, and concentrated to provide the crude product, which was purified by column chromatography to yield **2p** (890 mg, 75%): ^{1}H NMR (500 MHz, $CDCl_3$) *δ* 7.48 (d, *J* = 7.7 Hz, 1H), 7.38–7.31 (m, 4H), 7.30–7.27 (m, 1H), 5.52 (d, *J* = 6.5 Hz, 1H), 4.73 (dt, *J* = 9.1, 4.8 Hz, 1H), 4.62 (q, *J* = 11.4 Hz, 2H), 4.36 (d, *J* = 4.6 Hz, 1H), 4.18 (d, *J* = 3.7 Hz, 1H), 3.76 (s, 3H), 2.81–2.62 (m, 2H), 1.79 (s, 1H), 1.47 (s, 9H), 1.24 (d, *J* = 6.3 Hz, 3H); ^{13}C NMR (125 MHz, $CDCl_3$) *δ* 170.49, 170.08, 155.89, 138.08, 128.47, 127.95, 127.83, 80.29, 78.21, 74.98, 71.88, 71.69, 57.60, 52.82, 50.93, 28.44, 22.44, 15.28.

***N*-(*Tert*-butoxycarbonyl)-L-tryptophanyl-L-*C*-propargylglycine methyl ester (2o)**

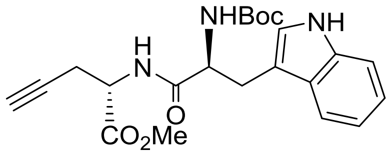

340 mg, 82%. ^{1}H NMR (500 MHz, $CDCl_3$) δ 8.16 (s, 1H), 7.64 (t, *J* = 5.4 Hz, 1H), 7.36 (t, *J* = 5.7 Hz, 1H), 7.23–7.04 (m, 3H), 6.60 (s, 1H), 5.18 (s, 1H), 4.59 (s, 1H), 4.50 (s, 1H), 3.70 (s, 3H), 3.43–3.15 (m, 2H), 2.67 (s, 3H), 1.87 (s, 1H), 1.44 (s, 9H); ^{13}C NMR (125 MHz, $CDCl_3$) δ 170.31, 136.32, 123.33, 122.36, 119.87, 118.93, 111.27, 110.55, 78.24, 71.68, 55.28, 52.87, 50.86, 28.43, 22.43; HRMS (ESI) calcd. for $C_{22}H_{27}N_3O_5Na$ $(M + Na)^+$ m/z 436.1843, found 436.1841.

***N*-(*Tert*-butoxycarbonyl)-L-phenylalanyl-L-*C*-propargylglycine methyl ester (2q)**

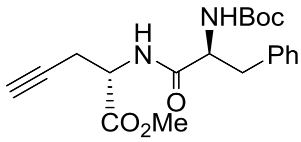

1.48 g, 79% (5 mmol scale). ^{1}H NMR (500 MHz, $CDCl_3$) δ 7.31 (t, *J* = 7.4 Hz, 2H), 7.2 6 (m, 1H), 7.21 (d, *J* = 7.6 Hz, 3H), 6.69 (d, *J* = 7.6 Hz, 1H), 4.97 (s, 1H), 4.67 (s, 1H), 4.42 (s, 1H), 3.76 (s, 3H), 3.10 (d, *J* = 6.6 Hz, 2H), 2.75 (dt, *J* = 4.8, 2.4 Hz, 2H), 1.97 (t, *J* = 2.6 Hz, 1H), 1.42 (s, 9H); ^{13}C NMR (125 MHz, $CDCl_3$) δ 171.17, 170.39, 155.41, 129.44, 128.85, 127.10, 80.45, 78.27, 71.82, 55.67, 52.96, 50.89, 38.35, 28.38, 22.46; HRMS (ESI) calcd. for $C_{20}H_{26}N_2O_5Na$ $(M + Na)^+$ m/z 397.1734, found 397.1736.

General Procedure II: preparation of tripeptides

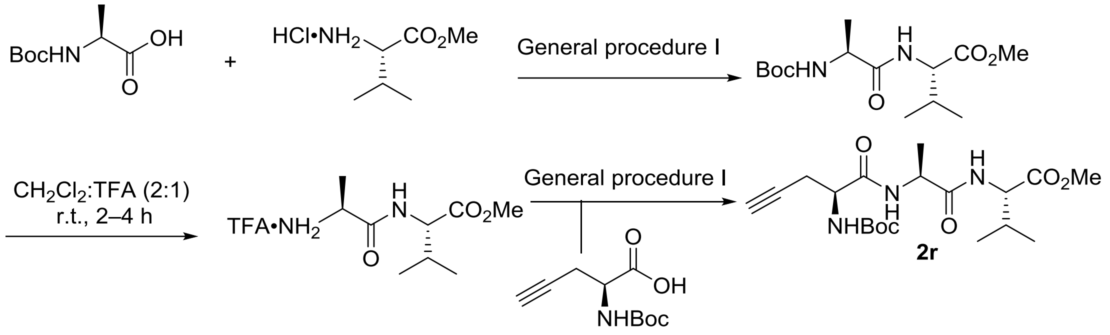

Supplementary Figure 23. General Procedure II for preparation of tripeptides (**2r** as an example).

To a suspension of Boc-L-alanine (1.13 g, 6.0 mmol, 1.0 eq), methyl L-valinate hydrochloride (6 mmol, 1.0 equiv.), and HOBt (1.2 eq) in dry DMF (15 mL) was added DIPEA (3.13 mL, 3.0 eq) under -10 ℃. After which, EDCI (1.2 eq) was added. The reaction mixture was stirred under -10 ℃ for 0.5 h. Then the mixture was warmed to room temperature and stirred overnight. After dilution with ethyl acetate, the organic phase was washed with water, and 1 N aq. HCl, and brine thrice. The organic phase was dried over anhydrous $MgSO_4$ and concentrated to provide the crude dipeptide. The crude product was dissolved in dry CH_2Cl_2 (15 mL) and cooled to 0 ℃. TFA (5 mL) was added slowly and the mixture was stirred for 2–4 h (monitored by TLC) at room temperature to remove thr Boc group. After completion, the reaction solution was concentrated and dried *in vacuo*.

To a stirred solution of the above crude product, Boc-*L*-propargylglycine (1.06 g, 5 mmol), and HOBt (1.2 eq) in dry DMF (20 mL) was added DIPEA (3.13 mL, 3.0 eq) under -10 ℃. After which, EDCI (1.2 eq) was added. The reaction mixture was stirred under -10 ℃ for 0.5 h. Then the mixture was warmed to room temperature and stirred overnight. After dilution with ethyl acetate, the organic phase was washed with water, 1N aq. HCl, and brine thrice. The organic phase was dried over anhydrous $MgSO_4$ and concentrated. The pure product was obtained by column chromatography.

***N*-(*Tert*-butoxycarbonyl)-L-*C*-propargylglycyl-L-alanyl-L-valine methyl ester (2r)**

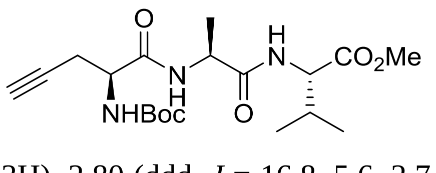

1.83 g, 91%. ^{1}H NMR (500 MHz, $CDCl_3$) δ 7.03 (d, J = 7.4 Hz, 1H), 6.88 (d, J = 8.7 Hz, 1H), 5.41 (d, J = 8.1 Hz, 1H), 4.61 (p, J = 7.0 Hz, 1H), 4.52 (dd, J = 8.8, 5.0 Hz, 1H), 4.33 (d, J = 7.0 Hz, 1H), 3.75 (s, 3H), 2.80 (ddd, J = 16.8, 5.6, 2.7 Hz, 1H), 2.60 (ddd, J = 16.9, 6.8, 2.6 Hz, 1H), 2.17 (pd, J = 6.9, 5.0 Hz, 1H), 2.09 (t, J = 2.6 Hz, 1H), 1.46 (s, 9H), 1.40 (d, J = 7.0 Hz, 3H), 0.93 (d, J = 6.9 Hz, 3H), 0.90 (d, J = 6.9 Hz, 3H); ^{13}C NMR (125 MHz, $CDCl_3$) δ 172.25, 171.75, 170.19, 155.41, 80.72, 79.30, 72.08, 57.40, 53.01, 52.32, 49.29, 31.28, 28.39, 22.62, 19.10, 18.35, 17.91; HRMS (ESI) calcd. for $C_{19}H_{31}N_3O_6Na$ $(M + Na)^+$ m/z 420.2105, found 420.2108.

***N*-(*Tert*-butoxycarbonyl)-L-*C*-propargylglycyl-L-tertleucyl-L-alanine methyl ester (2s)**

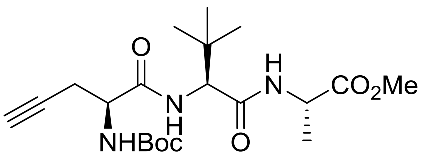

1.82 g, 88%. ^1H NMR (500 MHz, $CDCl_3$) δ 7.25 (d, J = 9.2 Hz, 1H), 7.15 (d, J = 7.3 Hz, 1H), 5.59 (d, J = 8.4 Hz, 1H), 4.57 (p, J = 7.2 Hz, 1H), 4.46 (d, J = 9.3 Hz, 1H), 4.41 (d, J = 7.2 Hz, 1H), 3.74 (s, 3H), 2.79 (ddd, J = 16.9, 5.7, 2.6 Hz, 1H), 2.65–2.55 (m, 1H), 2.09 (d, J = 2.6 Hz, 1H), 1.46 (s, 9H), 1.39 (d, J = 7.2 Hz, 3H), 1.02 (s, 9H); ^{13}C NMR (125 MHz, $CDCl_3$) δ 173.29, 170.34, 169.76, 155.45, 80.44, 79.52, 77.16, 71.85, 60.52, 53.18, 52.42, 47.98, 35.05, 28.34, 26.61, 22.49, 18.02; HRMS (ESI) calcd. for $C_{20}H_{33}N_3O_6Na$ $(M + Na)^+$ m/z 434.2262, found 434.2265.

***N*-(*Tert*-butoxycarbonyl)-L-*C*-propargylglycyl-L-alanyl-*O*-(*tert*-butyl)-L-threonine methyl ester (2t)**

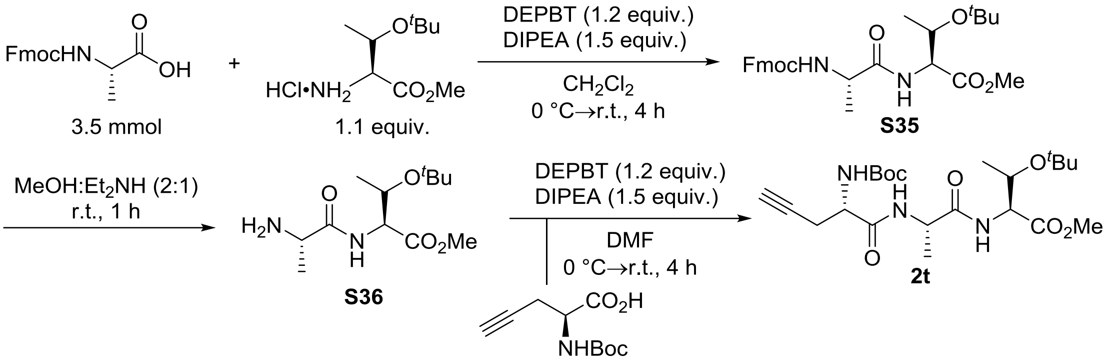

Supplementary Figure 24. Preparation of tripeptide **2t**.

To a stirred solution of *N*-Fmoc-L-alanine (1.09 g, 3.5 mmol), methyl *O*-(*tert*-butyl)-L-threoninate hydrochloride (1.1 equiv.), and 3-(diethoxyphosphoryloxy)-1,2,3-benzotrizin-4(3H)-one (DEPBT) (1.2 equiv.) in dry CH_2Cl_2 (8 mL) was added DIPEA (1.5 eq) under 0 ℃. The mixture was stirred under 0 ℃ for 0.5 h, then warmed to room temperature slowly and stirred for 4 h. After dilution with CH_2Cl_2, the organic phase was washed with water and brine thrice. The organic phase was dried over anhydrous Na_2SO_4 and concentrated. The crude product was dissolved in MeOH (22 mL), and Et_2NH (11 mL) was added. The mixture was stirred for 1 h at room temperature to remove the Fmoc. After completion, the solvent and residue Et_2NH were removed *in vacuo*. To a stirred solution of the crude product, Boc-L-propargylglycine (1.2 equiv.) and 3-(diethoxyphosphoryloxy)-1,2,3-benzotrizin-4(3H)-one (DEPBT) (1.2 equiv.) in dry DMF (15 mL) was added DIPEA (1.5 eq) at 0 ℃. The mixture was stirred under 0 ℃ for 0.5 h, then warmed to room temperature slowly and stirred for 4 h. After dilution with ethyl acetate, the organic phase was washed with water and brine thrice. The organic phase was dried over anhydrous Na_2SO_4 and concentrated, and the residue was purified by column chromatography to yield **2t** (546 mg, 34%): ^1H NMR (500 MHz, $CDCl_3$) δ 7.00 (d, J = 7.3 Hz, 1H), 6.49 (d, J = 9.2 Hz, 1H), 5.32 (d, J = 8.1 Hz, 1H), 4.58 (p, J = 7.0 Hz, 1H), 4.48 (dd, J = 9.2, 1.8 Hz, 1H), 4.35–4.29 (m, 1H), 4.24 (qd, J = 6.3, 1.8 Hz, 1H), 3.71 (s, 3H), 2.82 (d, J = 17.5 Hz, 1H), 2.59 (ddd, J = 16.9, 6.8, 2.6

Hz, 1H), 2.10 (t, J = 2.6 Hz, 1H), 1.46–1.45 (m, 12H)1.16 (d, J = 6.3 Hz, 3H), 1.12 (s, 9H); ^{13}C NMR (125 MHz, $CDCl_3$) δ 172.31, 171.05, 169.76, 155.36, 80.65, 79.43, 74.37, 72.03, 67.36, 58.00, 52.95, 52.40, 49.27, 28.45, 28.40, 22.74, 21.11, 19.09; HRMS (ESI) calcd. for $C_{22}H_{37}N_3O_7Na$ (M + Na)$^+$ m/z 478.2524, found 478.2526.

Methyl *N*-(9-fluorenylmethoxycarbonyl)-*O*-(*tert*-butyl)-L-threonyl-L-*C*-propargylglycyl-L-alloisoleucinate (2u)

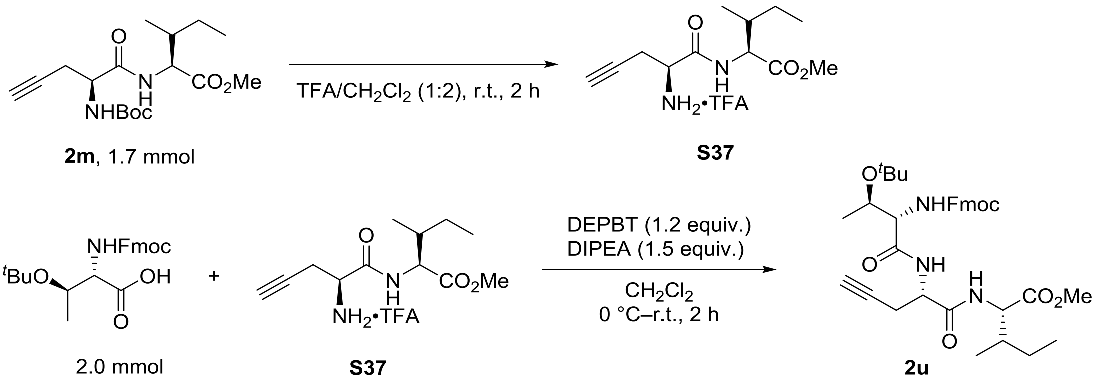

Supplementary Figure 25. Preparation of tripeptide **2u**.

To a stirred solution of methyl (tert-butoxycarbonyl)-L-propargylglycyl-L-alloisoleucinate (**2m**) (578 mg, 1.7 mmol) in dry CH_2Cl_2 (6 mL) was added TFA (3 mL) slowly at 0 ℃. The mixture was stirred for 2–4 h at room temperature to remove the Boc group (monitored by TLC). After completion, the reaction solution was concentrated and dried over oil pump to give the crude **S37**.

To a stirred solution of the above crude **S37**, *N*-(9-fluorenylmethoxycarbonyl)-*O*-(tert-butyl)-L-threonine (0.79 g, 2.0 mmol) and 3-(diethoxyphosphoryloxy)-1,2,3-benzotrizin-4(3H)-one (DEPBT) (1.2 equiv.) in dry CH_2Cl_2 (8 mL) was added DIPEA (1.5 eq) under 0 ℃. The mixture was stirred under 0 ℃ for 0.5 h, then warmed to room temperature slowly and stirred for 2 h. After dilution with CH_2Cl_2, the organic phase was washed with water and brine thrice. The organic phase was dried over anhydrous Na_2SO_4 and concentrated, and the residue was purified by column chromatography to yield **2u** (154 mg, 25%): ^{1}H NMR (500 MHz, $CDCl_3$) δ 7.76 (d, J = 7.5 Hz, 2H), 7.61 (dd, J = 10.2, 7.9 Hz, 3H), 7.40 (t, J = 7.3 Hz, 2H), 7.32 (t, J = 7.4 Hz, 2H), 6.88 (d, J = 8.8 Hz, 1H), 6.00 (d, J = 5.4 Hz, 1H), 4.73 (dt, J = 8.8, 5.5 Hz, 1H), 4.62 (dd, J = 8.7, 5.1 Hz, 1H), 4.40 (d, J = 7.2 Hz, 2H), 4.28 (t, J = 4.7 Hz, 1H), 4.25–4.17 (m, 2H), 3.72 (s, 3H), 2.96 (ddd, J = 16.9, 5.1, 2.6 Hz, 1H), 2.59 (ddd, J = 17.0, 6.0, 2.6 Hz, 1H), 2.07 (t, J = 2.6 Hz, 1H), 1.97–1.83 (m, 1H), 1.45 (tdt, J = 10.7, 7.9, 3.1 Hz, 1H), 1.32 (s, 9H), 1.28–1.17 (m, 1H), 1.12 (d, J = 6.4 Hz, 3H), 0.91 (t, J = 7.3 Hz, 6H); ^{13}C NMR (125 MHz, $CDCl_3$) δ 171.83, 169.53, 169.41, 156.06, 143.96, 143.74, 141.43, 141.40, 127.85, 127.17, 127.15, 125.24, 125.19, 120.13, 120.10, 79.41, 76.02, 71.99, 67.15, 66.78, 58.87, 56.86, 52.19, 51.71, 47.26, 37.88, 28.27, 25.16, 22.24, 17.08, 15.55, 11.60; HRMS (ESI) calcd. for $C_{35}H_{45}N_3O_7Na$ (M + Na)$^+$ m/z 642.3150, found 642.3152.

Methyl (*S*)-2-((*tert*-butoxycarbonyl)amino)-5-phenylpent-4-ynoate (2v)[22]

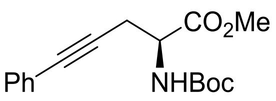

^{1}H NMR (500 MHz, $CDCl_3$) δ 7.41–7.34 (m, 2H), 7.35–7.20 (m, 3H), 5.39 (d, *J* = 8.7 Hz, 1H), 4.56 (dt, *J* = 9.6, 5.1 Hz, 1H), 3.80 (s, 3H), 3.00–2.80 (m, 2H), 1.46 (s, 9H).

2.3 Optimization of Reaction Conditions with Mannose Donors

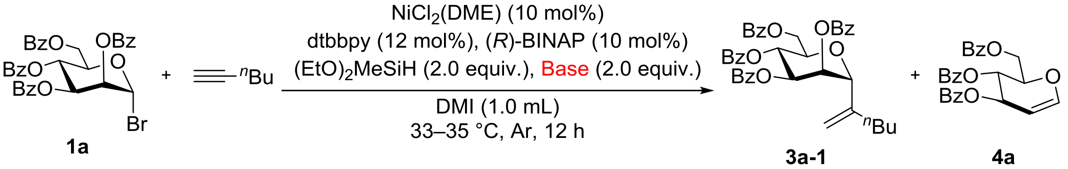

Entry	Base	Yield (%)	
		**3a-1**	**4a**
1	$NaHCO_3$	0	
2	NaH_2PO_4	0	
3	Li_2CO_3	<10	
4	Na_2CO_3	60	
5	K_2CO_3	12	
6	Cs_2CO_3	57	
7	KF	56	15

Supplementary Figure 26. Optimization of the base. The yield was determined by ^{1}H NMR using CH_2Br_2 as an internal standard. dtbbpy = 4,4'-di-*tert*-butyl-2,2'-bipyridine, DMI = 1,3-dimethyl-2-imidazolidinone.

2-(2,3,4,6-Tetra-*O*-benzoyl-α-D-mannopyranosyl)-1-hexene (**3a-1**)

^{1}H NMR (500 MHz, $CDCl_3$) *δ* 8.09 (t, *J* = 8.0 Hz, 4H), 7.96 (d, *J* = 7.2 Hz, 2H), 7.87 (d, *J* = 7.2 Hz, 2H), 7.59 (q, *J* = 7.6 Hz, 2H), 7.51 (t, *J* = 7.4 Hz, 1H), 7.47–7.40 (m, 5H), 7.36 (t, *J* = 7.8 Hz, 2H), 7.29 (t, *J* = 7.8 Hz, 2H), 6.23 (dd, *J* = 3.1, 1.9 Hz, 1H), 6.08 (t, *J* = 9.9 Hz, 1H), 5.78 (dd, *J* = 9.9, 3.1 Hz, 1H), 5.58 (d, *J* = 2.0 Hz, 1H), 5.41 (q, *J* = 1.4 Hz, 1H), 4.73 (d, *J* = 1.9 Hz, 1H), 4.65 (dd, *J* = 12.0, 2.5 Hz, 1H), 4.52 (dd, *J* = 12.0, 5.9 Hz, 1H), 4.16 (ddd, *J* = 9.9, 5.9, 2.5 Hz, 1H), 2.29 (ddd, *J* = 15.3, 9.8, 5.4 Hz, 1H), 2.19 (ddd, *J* = 15.5, 9.8, 6.2 Hz, 1H), 1.59–1.52 (m, 1H), 1.48 (dddd, *J* = 13.1, 9.6, 6.5, 3.1 Hz, 1H), 1.31 (tt, *J* = 14.2, 7.4 Hz, 2H), 0.90 (t, *J* = 7.3 Hz, 3H); ^{13}C NMR (125 MHz, $CDCl_3$) *δ* 166.32, 166.14, 165.80, 165.58, 143.98, 133.58, 133.48, 133.39, 133.22, 129.97, 129.90, 129.84, 129.68, 129.08, 128.65, 128.56, 128.51, 128.47, 115.10, 77.19, 71.32, 71.24, 70.34, 67.62, 63.69, 33.14, 29.74, 22.58, 14.07; HRMS (ESI) calcd. for $C_{40}H_{38}O_9Na$ $(M + Na)^+$ m/z 685.2408, found 385.2409.

2,3,4,6-Tetra-*O*-benzoyl-D-glucal 4a[23]

^{1}H NMR (500 MHz, $CDCl_3$) *δ* 8.07–7.97 (m, 6H), 7.59–7.51 (m, 3H), 7.46–7.36 (m, 6H), 6.61 (dd, *J* = 6.2, 1.2 Hz, 1H), 5.85–5.75 (m, 1H), 5.71 (ddd, *J* = 5.4, 3.7, 1.1 Hz, 1H), 5.12 (dd, *J* = 6.2, 3.5 Hz, 1H), 4.73–4.63 (m, 3H).

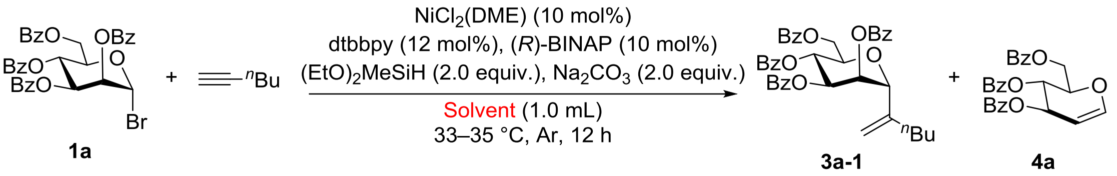

Entry	Solvent	Yield (%)		
		3a-1	4a	1a
1	DMF	<20		
2	DMAc	61	<5	
3	DME	63		30
4	Dioxane	<20		
5	THF	53	6%	20

Supplementary Figure 27. Optimization of the solvent. The yield was determined by ^{1}H NMR using CH_2Br_2 as an internal standard. dtbbpy = 4,4'-di-*tert*-butyl-2,2'-bipyridine, DME = 1,2-dimethoxyethane, DMAc = *N*,*N*-dimethylacetamide.

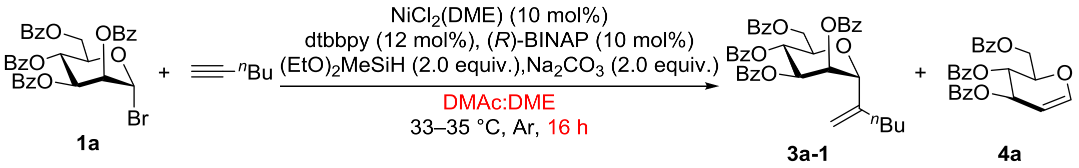

Entry	DMAc:DME	Yield (%)		
		3a-1	4a	1a
1	10:1	66	<10	0
2	5:1	63	<10	0
3	2:1	66	<10	0
4	1:2	65	<10	0
5	1:5	>66	<10	17
6	1:10	76	<5	13

Supplementary Figure 28. Further optimization of the solvent. The yield was determined by ^{1}H NMR using CH_2Br_2 as an internal standard. dtbbpy = 4,4'-di-*tert*-butyl-2,2'-bipyridine, DME = 1,2-dimethoxyethane, DMAc = *N*,*N*-dimethylacetamide.

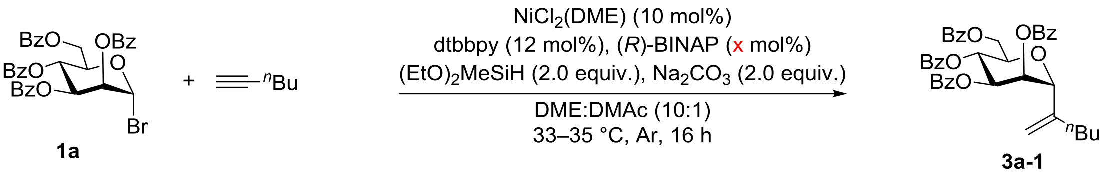

Entry	x	Yield (%) 3a-1	Yield (%) 1a
1	0	42	31
2	5	67	13
3	10	72	13
4	15	43	38
5	20	56	26
6[a]	15	75	7

Supplementary Figure 29. Optimization of the equivalents of (*R*)-BINAP. The yield was determined by ^{1}H NMR using CH_2Br_2 as an internal standard. [a]22 h. dtbbpy = 4,4'-di-*tert*-butyl-2,2'-bipyridine, DME = 1,2-dimethoxyethane, DMAc = *N*,*N*-dimethylacetamide.

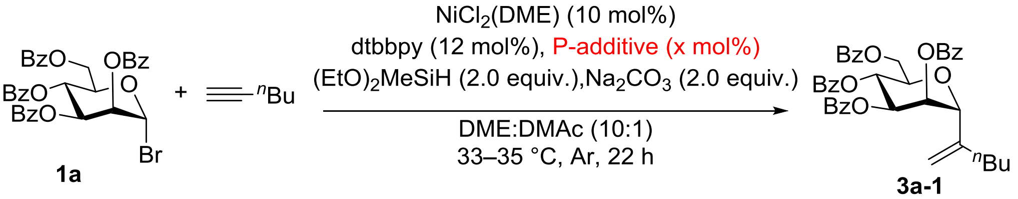

Entry	P-additive (x mol%)	Yield (%) 3a-1	Yield (%) 1a
1[a]	(*R*)-BINAP (10)	0	92
2	(*rac*)-BINAP (10)	67	23
3	(*S*)-BINAP (10)	65	15
4	PPh_3 (20)	77 (80)[b]	0
5	dppe (10)	53	20
6	$P(p\text{-}MeOC_6H_4)_3$ (20)	60	30
7	P(2-furanyl)$_3$ (20)	65	15
8	$P(o\text{-}MeC_6H_4)_3$ (20)	38	14
9	$P(p\text{-}CF_3C_6H_4)_3$ (20)	--	

Supplementary Figure 30. Optimization of the P-based additive. The yield was determined by ^{1}H NMR using CH_2Br_2 as an internal standard. [a]Without dtbbpy. [b]Isolated yield. dtbbpy = 4,4'-di-*tert*-butyl-2,2'-bipyridine, DME = 1,2-dimethoxyethane, DMAc = *N*,*N*-dimethylacetamide.

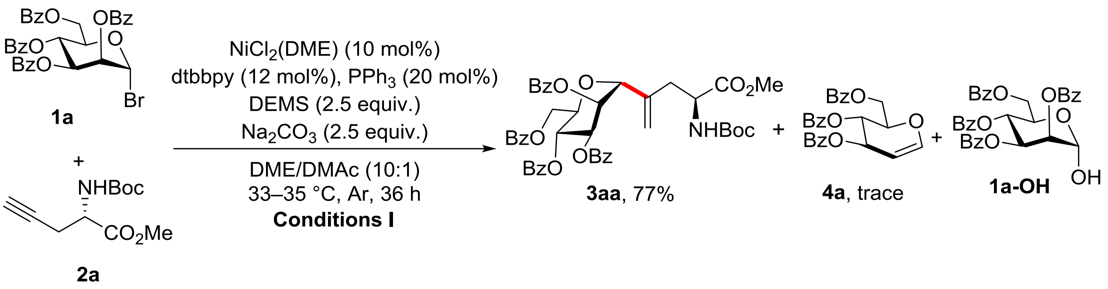

Entry	deviation from the **Conditions I**	Yield (%)			
		**3aa**	**4a**	**1a-OH**	**1a**
1	none	87 (77)[a]	<5		
2	air	trace		57	38
3	w/o PPh_3	81			
4	w/o dtbbpy		<5	48	46
5	w/o Na_2CO_3			63	31
6	$Ni(PPh_3)_2Cl_2$, w/o PPh_3	90 (82)[b]			
7	$Ni(PPh_3)_2Cl_2$, w/o PPh_3, w/o dtbbpy				92
8	**cat. 1**, w/o dtbbpy	90 (82)[b]	<5		
9	**cat. 1**, w/o dtbbpy, w/o PPh_3	87	5		

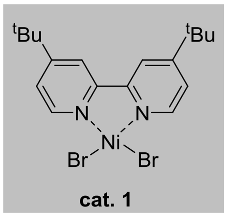

cat. 1

Supplementary Figure 31. Optimized Conditions I and control experiments for the synthesis of **3aa**. The yield was determined by ^{1}H NMR using CH_2Br_2 as an internal standard. [a]Isolated yield. [b]Average of two isolated yields for entry 6 and entry 8.

2,3,4,6-Tetra-*O*-benzoyl-α/β-D-mannopyranose (1a-OH)

^{1}H NMR (500 MHz, CD_3Cl) δ 8.12 (d, J = 7.3 Hz, 2H), 8.02 (d, J = 7.4 Hz, 2H), 7.96 (d, J = 7.4 Hz, 2H), 7.85 (d, J = 7.4 Hz, 2H), 7.56 (t, J = 7.0 Hz, 2H), 7.49 (t, J = 7.4 Hz, 1H), 7.41 (q, J = 7.8 Hz, 3H), 7.35 (q, J = 7.6 Hz, 4H), 7.26 (t, J = 7.3 Hz, 2H), 6.19 (t, J = 10.1 Hz, 1H), 6.02 (dd, J = 10.2, 3.2 Hz, 1H), 5.75 (dd, J = 3.2, 1.9 Hz, 1H), 5.54 (dd, J = 3.9, 1.9 Hz, 1H), 4.77 (dd, J = 12.2, 2.7 Hz, 1H), 4.68 (dt, J = 10.1, 3.1 Hz, 1H), 4.44 (dd, J = 12.2, 3.5 Hz, 1H), 4.30 (d, J = 4.1 Hz, 1H).

2.4 Optimization of Reaction Conditions with Glucosamine Donors (GlcN)

We found that the judicious choice of protecting groups (i.e., *N*-Phth and *O*-Bz/Ac) on the GlcN substrates was critical for the present coupling reaction.

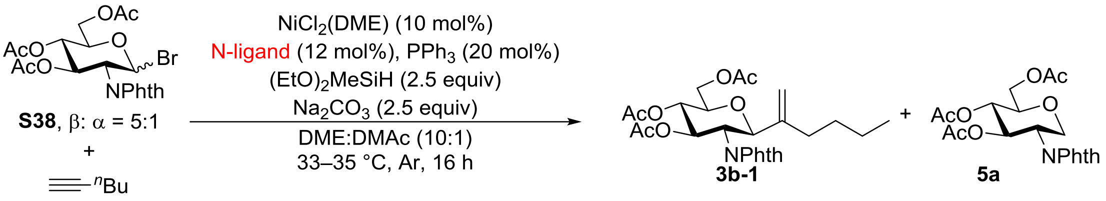

Entry	N-ligand	Yield (%)	
		3b-1	5a
1	L_1	10	40
2	L_2	5	33
3	L_3	trace	>20
4	L_4	trace	>20
5	L_5	--	>20
6	L_6	--	>20
7	L_7	--	>20
8	L_8	--	>20
9	L_9	--	>20

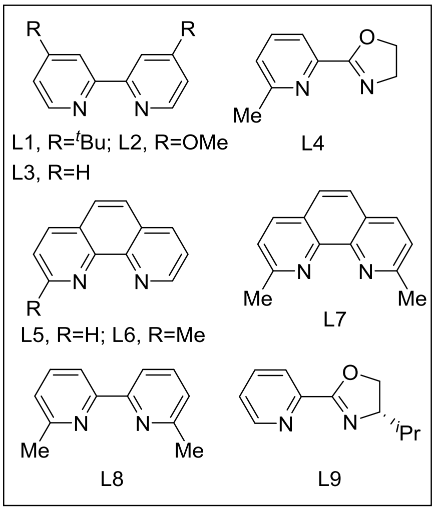

Supplementary Figure 32. Optimization of the *N*-ligand. The yield was determined by ^{1}H NMR using CH_2Br_2 as an internal standard. DME = 1,2-dimethoxyethane, DMAc = *N*,*N*-dimethylacetamide.

2-(3,4,6-Tri-*O*-acetyl-2-deoxy-2-phthalimido-β-D-glucopyranosyl)-1-hexene (3b-1)
Colorless syrup. ^{1}H NMR (500 MHz, $CDCl_3$) *δ* 7.86–7.79 (m, 2H), 7.76–7.70 (m, 2H), 5.87 (dd, *J* = 10.4, 9.1 Hz, 1H), 5.18 (t, *J* = 9.6 Hz, 1H), 4.94 (s, 1H), 4.84 (d, *J* = 10.7 Hz, 2H), 4.45 (t, *J* = 10.4 Hz, 1H), 4.27 (dd, *J* = 12.2, 4.5 Hz, 1H), 4.18 (dd, *J* = 12.2, 2.2 Hz, 1H), 3.87 (ddd, *J* = 10.1, 4.5, 2.4 Hz, 1H), 2.18–2.08 (m, 4H), 2.04 (s, 3H), 2.00 (dd, *J* = 10.1, 5.3 Hz, 1H), 1.86 (s, 3H), 1.41 (ddt, *J* = 16.5, 12.1, 6.0 Hz, 1H), 1.30 (qt, *J* = 12.6, 5.6 Hz, 3H), 0.87 (t, *J* = 7.2 Hz, 3H); ^{13}C NMR (125 MHz, $CDCl_3$) *δ* 170.93, 170.34, 169.79, 167.99, 167.43, 144.79, 134.49, 134.30, 131.75, 131.14, 123.69, 115.02, 79.59, 75.72, 71.89, 69.41, 62.58, 52.93, 29.98, 29.75, 22.56, 20.95, 20.84, 20.66, 14.07; HRMS (ESI) calcd. for $C_{26}H_{31}NO_9Na$ $(M + Na)^+$ m/z 524.1891, found 524.1895.

3,4,6-Tri-*O*-acetyl-1,5-anhydro-2-deoxy-2-phthalimido-D-glucitol (5a)[24]
^{1}H NMR (500 MHz, $CDCl_3$) *δ* 7.85 (dd, *J* = 5.5, 3.0 Hz, 2H), 7.74 (dd, *J* = 5.5, 3.1 Hz, 2H), 5.84 (dd, *J* = 10.4, 9.1 Hz, 1H), 5.13 (dd, *J* = 10.1, 9.1 Hz, 1H), 4.56 (ddd, *J* = 11.5, 10.5, 5.4 Hz, 1H), 4.35 (t, *J* = 11.3 Hz, 1H), 4.27 (dd, *J* = 12.3, 4.8 Hz, 1H), 4.18 (dd, *J* = 12.3, 2.2 Hz, 1H), 3.95 (dd, *J* = 11.2, 5.5 Hz, 1H), 3.79 (ddd, *J* = 10.1, 4.8, 2.2 Hz, 1H), 2.13 (s, 3H), 2.04 (s, 3H), 1.87 (s, 3H).

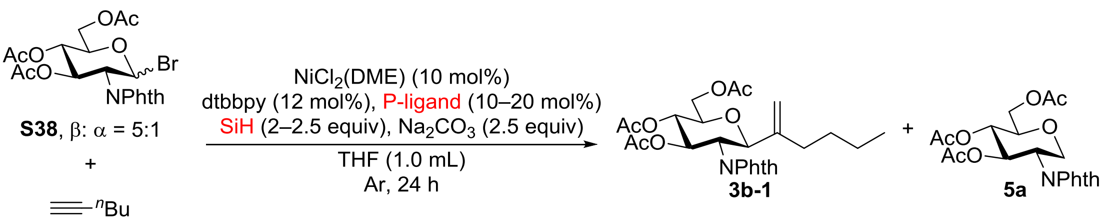

Entry	P-additives	Yield (%)	
		3b-1	5a
1	--	42	22
2	L_1	37	34
3	L_2	52	18
4	L_3	43	37
5	L_4	65	8
6	L_5	58	17

Using $(EtO)_2MeSiH$ (2.0 equiv), 33–35 °C, Ar, 24 h. 20 mol% for L1, L2, L3; 10 mol% for L4, L5

Entry	P-additives	Yield (%)	
		3b-1	5a
1	--	28	trace
2	L_1	53	trace
3	L_2	50	trace
4	L_3	52	trace
5	L_4	68 (70)[a]	trace
6	L_5	70 (70)[a]	trace

Using PMHS (2.5 equiv), r.t. (18–23 °C), Ar, 24 h. 20 mol% for L1, L2, L3; 10 mol% for L4, L5

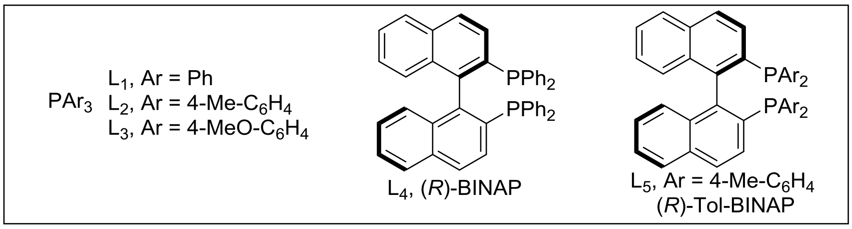

Supplementary Figure 33. Optimization of the P-based additive and silanes. The yield was determined by ^{1}H NMR using CH_2Br_2 as an internal standard. [a]Average of two isolated yields for entry 5 and entry 6. dtbbpy = 4,4’-di-*tert*-butyl-2,2’-bipyridine, PMHS = poly(methylhydrosiloxane).

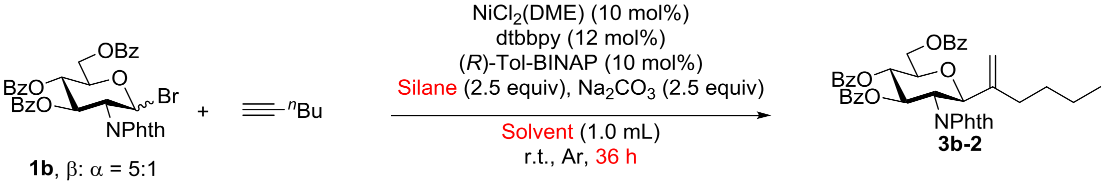

Entry	Solvent	Silane	Yield of **3b-2** (%)
1	DME:DMAc (10:1)	$(EtO)_2MeSiH$	>91
2	DME:DMAc (10:1)	PMHS	>91
3	THF	$(EtO)_2MeSiH$	>91
4	THF	PMHS	>91

Supplementary Figure 34. Control experiments using **1b** as glycosyl donor. The yield was determined by ^{1}H NMR using CH_2Br_2 as an internal standard. dtbbpy = 4,4'-di-*tert*-butyl-2,2'-bipyridine, PMHS = poly(methylhydrosiloxane).

2-(3,4,6-Tri-*O*-benzoyl-2-deoxy-2-phthalimido-β-D-glucopyranosyl)-1-hexene (**3b-2**)

^{1}H NMR (500 MHz, $CDCl_3$) δ 8.05 (d, *J* = 7.2 Hz, 2H), 7.91 (d, *J* = 7.2 Hz, 2H), 7.86 (d, *J* = 7.8 Hz, 1H), 7.76 (d, *J* = 7.2 Hz, 2H), 7.68 (dt, *J* = 6.6, 3.1 Hz, 2H), 7.64 (d, *J* = 7.2 Hz, 1H), 7.55 (t, *J* = 7.4 Hz, 1H), 7.47 (t, *J* = 7.4 Hz, 1H), 7.41 (q, *J* = 8.0 Hz, 3H), 7.32 (t, *J* = 7.8 Hz, 2H), 7.25 (t, *J* = 7.8 Hz, 2H), 6.36 (dd, *J* = 10.4, 9.3 Hz, 1H), 5.73 (t, *J* = 9.6 Hz, 1H), 5.07 (d, *J* = 10.4 Hz, 1H), 5.01 (s, 1H), 4.86 (d, *J* = 1.6 Hz, 1H), 4.70 (t, *J* = 10.5 Hz, 1H), 4.66 (dd, *J* = 12.2, 3.1 Hz, 1H), 4.47 (dd, *J* = 12.1, 4.5 Hz, 1H), 4.25 (ddd, *J* = 10.1, 4.5, 3.1 Hz, 1H), 2.20 (ddd, *J* = 15.7, 10.0, 5.4 Hz, 1H), 2.04 (ddd, *J* = 15.9, 10.0, 5.7 Hz, 1H), 1.43 (m, 1H), 1.40–1.32 (m, 1H), 1.27 (m, 2H), 0.83 (t, *J* = 7.3 Hz, 3H); ^{13}C NMR (125 MHz, $CDCl_3$) δ 168.18, 167.42, 166.28, 165.85, 165.38, 144.88, 134.35, 134.16, 133.44, 133.29, 133.13, 131.74, 131.13, 129.93, 129.86, 129.84, 129.05, 128.84, 128.46, 128.45, 128.37, 123.66, 123.63, 115.07, 79.79, 75.91, 72.31, 70.46, 63.36, 53.25, 30.05, 29.91, 22.57, 14.02; HRMS (ESI) calcd. for $C_{41}H_{37}NO_9Na$ $(M + Na)^+$ m/z 710.2361, found 710.2364.

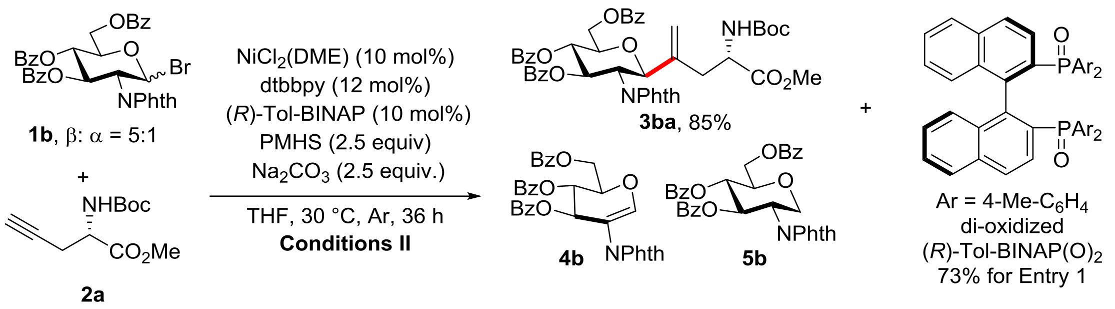

Entry	deviation from the **Conditions II**	Yield (%)			
		**3ba**	**4b**	**5b**	**1b**
1	none	90 (85)[a]	<5	<5	trace
2	air	trace	48		26
3	O_2	trace	44		36
4	w/o (*R*)-Tol-BINAP	73	<5	16	trace
5	w/o dtbbpy	trace	20		66
6	$Ni(COD)_2$, w/o (*R*)-Tol-BINAP, 16 h	60	5	12	19
7	$Ni(COD)_2$, w/o (*R*)-Tol-BINAP, 24 h	73	5	18	trace

Supplementary Figure 35. Optimized Conditions II and control experiments for the synthesis of **3ba**. The yield was determined by ^{1}H NMR using CH_2Br_2 as an internal standard. [a]Isolated yield.

3,4,6-Tri-*O*-benzoyl-2-phthalimido-glucal (**4b**)

^{1}H NMR (500 MHz, $CDCl_3$) δ 8.15 (d, J = 8.3 Hz, 2H), 8.02 (d, J = 8.4 Hz, 2H), 7.90 (d, J = 8.3 Hz, 2H), 7.87–7.82 (m, 2H), 7.74–7.68 (m, 2H), 7.60 (t, J = 7.4 Hz, 1H), 7.55 (t, J = 7.4 Hz, 1H), 7.48 (t, J = 6.4 Hz, 3H), 7.41 (t, J = 7.7 Hz, 2H), 7.35 (t, J = 7.7 Hz, 2H), 6.95 (s, 1H), 6.00–5.95 (m, 1H), 5.86 (dt, J = 3.5, 1.9 Hz, 1H), 5.04–4.98 (m, 1H), 4.97–4.90 (m, 1H), 4.80 (dd, J = 11.3, 5.3 Hz, 1H); ^{13}C NMR (125 MHz, $CDCl_3$) δ 167.82, 166.14, 165.72, 165.17, 148.75, 134.40, 133.76, 133.52, 133.38, 131.87, 130.23, 129.91, 129.87, 129.45, 129.13, 129.01, 128.70, 128.60, 128.55, 123.85, 110.12, 105.52, 74.32, 67.67, 66.27, 61.70; HRMS (ESI) calcd. for $C_{35}H_{25}NO_9Na$ (M + Na)$^+$ m/z 626.1422, found 626.1424.

3,4,6-Tri-*O*-benzoyl-1,5-anhydro-2-deoxy-2-phthalimido-D-glucitol (**5b**)

^{1}H NMR (500 MHz, $CDCl_3$) δ 8.08 (dd, J = 8.1, 1.4 Hz, 2H), 7.92 (dd, J = 8.1, 1.5 Hz, 2H), 7.80 (s, 2H), 7.79–7.73 (m, 2H), 7.69 (dd, J = 5.5, 3.0 Hz, H), 7.56 (t, J = 7.4 Hz, 1H), 7.51–7.39 (m, 4H), 7.34 (t, J = 7.8 Hz, 2H), 7.29–7.24 (m, 2H), 6.31 (dd, J = 10.5, 9.2 Hz, 1H), 5.69 (t, J = 9.6 Hz, 1H), 4.81 (td, J = 10.9, 5.4 Hz, 1H), 4.65 (dd, J = 12.2, 2.7 Hz, 1H), 4.55 (t, J = 11.4 Hz, 1H), 4.46 (dd, J = 12.2, 5.0 Hz, 1H), 4.17 (ddd, J = 10.1, 4.9, 2.7 Hz, 1H), 4.08 (dd, J = 11.3, 5.4 Hz, 1H); ^{13}C NMR (125 MHz, $CDCl_3$) δ 166.39, 165.96, 165.38, 134.41, 133.51, 133.34, 133.24, 131.53, 129.97, 129.96, 129.88, 129.80, 129.03, 128.83, 128.52, 128.41, 123.74, 76.96, 72.23, 70.26, 65.88, 63.34, 50.71; HRMS (ESI) calcd. for $C_{35}H_{27}NO_9Na$ (M + Na)$^+$ m/z 628.1578, found 628.1581.

[1,1'-binaphthalene]-2,2'-diylbis(di-p-tolylphosphine oxide) (di-oxidized (*R*)-Tol-BINAP(O)$_2$)[25]

^{1}H NMR (500 MHz, $CDCl_3$) δ 7.84–7.74 (m, 4H), 7.48 (dd, *J* = 12.0, 8.0 Hz, 4H), 7.43 (dd, *J* = 11.6, 8.6 Hz, 2H), 7.37 (ddd, *J* = 8.1, 5.9, 2.0 Hz, 2H), 7.27 (dd, *J* =11.3, 8.1, 4H), 7.03 (d, *J* = 6.4 Hz, 4H), 6.98 (d, *J* = 6.1 Hz, 4H), 6.89-6.85 (m, 4H), 2.32 (s, 6H), 2.27 (s, 6H); ^{31}P NMR (162 MHz, $CDCl_3$) δ 29.20.

2.5 Ni-Catalyzed synthesis of vinyl *C*-glycosyl amino acids and glycopeptides

General Procedure A (unless otherwise stated)

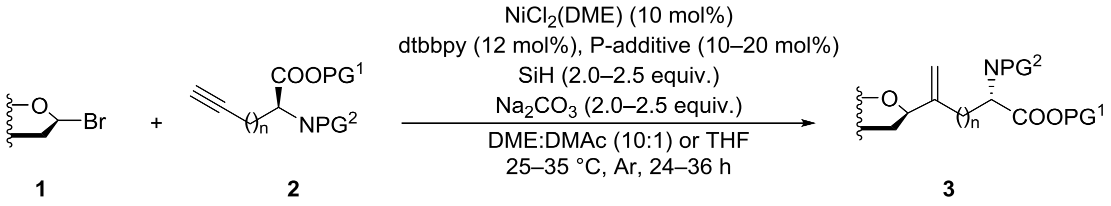

To an oven-dried 10 mL Schlenk tube (Titan, TF891910) containing a Teflon coated magnetic stirring bar were added glycosyl bromide **1** (0.1 mmol), $NiCl_2$(DME) (10 mol%), dtbbpy (12 mol%), PPh_3 (20 mol%), and Na_2CO_3 (2.5 equiv). The tube was sealed with a rubber cap then with parafilm and evacuated then refilled with Ar for at least five cycles. The acetylenic amino acid and peptide derivative 2 was dissolved in solvent (DME/DMAc = 1:1, 1.0 mL) and injected into the reaction tube (this substrate was added directly with glycosyl bromide if it was solid). When stirring, $(EtO)_2$MeSiH (40 µL, 2.5 equiv.) was injected via microliter syringe. Otherwise noted, the tube was moved to an oil bath preheated to 33–35 ℃ and kept stirring for 36 h. The reaction mixture was diluted with CH_2Cl_2 (20 mL) and filtered. After concentration, the residue was purified by column chromatography on silica gel or preparative TLC to afford the desired product **3**.

General Procedure B (unless otherwise stated)

To an oven-dried 10 mL Schlenk tube (Titan, TF891910) containing a Teflon coated magnetic stirring bar were added glycosyl bromide **1** (0.1 mmol), $NiCl_2$(DME) (10 mol%), dtbbpy (12 mol%), (*R*)-Tol-BINAP (10 mol%), and Na_2CO_3 (2.5 equiv). The tube was sealed with a rubber cap then with parafilm and evacuated then refilled with Ar for at least five cycles. The acetylenic amino acid and peptide derivative **2** was dissolved in THF (1.0 mL) and injected into the reaction tube (this substrate was added directly with glycosyl bromide if it was solid). When stirring, PMHS (32 µL, 2.5 equiv.) was injected via microliter syringe. Otherwise noted, the tube was kept stirring under 30 ℃ for 36 h. The reaction mixture was diluted with CH_2Cl_2 (20 mL) and filtered. After concentration, the residue was purified by flash column chromatography on silica gel or preparative TLC to afford the desired product **3**.

***N*-(*Tert*-butoxycarbonyl)-3-(1-(2,3,4,6-tetra-*O*-benzoyl-α-D-mannopyranosyl)eth-1-en-1-yl)-L-alanine methyl ester** (**3aa**)

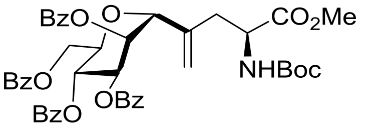

3aa

General Procedure A. White foam, 62.5 mg, 77%. $[\alpha]_D^{25}$ –57.5 (*c* 1.0, $CHCl_3$); ^1H NMR (500 MHz, $CDCl_3$) δ 8.09 (d, *J* = 7.4 Hz, 2H), 8.05 (d, *J* = 7.3 Hz, 2H), 7.96 (d, *J* = 7.3 Hz, 2H), 7.86 (d, *J* = 7.3 Hz, 2H), 7.59 (q, *J* = 7.6 Hz, 2H), 7.51 (t, *J* = 7.4 Hz, 2H), 7.44 (dq, *J* = 15.6, 7.8 Hz, 5H), 7.36 (t, *J* = 7.8 Hz, 2H), 7.30 (t, *J* = 7.8 Hz, 2H), 6.21 (t, *J* = 2.6 Hz, 1H), 6.07 (t, *J* = 9.6 Hz, 1H), **5.74 (s, 1H, vinyl H)**, 5.72 (dd, *J* = 9.8, 3.1 Hz, 1H), **5.51 (s, 1H, vinyl H)**, 5.11 (d, *J* = 8.2 Hz, 1H), **4.75 (s, 1H, anomeric H)**, 4.66 (dd, *J* = 12.1, 2.6 Hz, 1H), 4.55 (dd, *J* = 12.3, 5.5 Hz, 2H), 4.14 (ddd, *J* = 9.7, 5.5, 2.7 Hz, 1H), 3.69 (s, 3H), 2.79-2.65 (m, 2H), 1.42 (s, 9H); ^{13}C NMR (125 MHz, $CDCl_3$) δ 172.94, 166.29, 166.16, 165.67, 165.51, 139.37, 133.62, 133.54, 133.47, 133.25, 129.99, 129.92, 129.87, 129.85, 129.59, 129.07, 129.02, 128.68, 128.61, 128.59, 128.52, 119.38, 80.25, 77.16, 71.65, 71.08, 69.78, 67.41, 63.45, 52.51, 52.40, 36.20, 28.39; HRMS (ESI) calcd. for $C_{45}H_{35}NO_{13}Na$ $(M + Na)^+$ m/z 830.2783, found 830.2786.

***N*-Benzyloxycarbonyl-3-(1-(2,3,4,6-tetra-*O*-benzoyl-α-D-mannopyranosyl)eth-1-en-1-yl)-L-alanine methyl ester** (**3ab**)

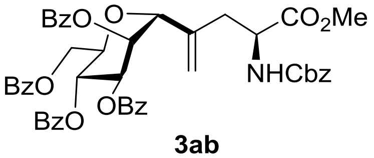

3ab

General Procedure A. White foam, 65.7 mg, 78%. $[\alpha]_D^{25}$ –52.1 (*c* 0.7, $CHCl_3$); ^1H NMR (500 MHz, $CDCl_3$) δ 8.08 (d, *J* = 7.4 Hz, 2H), 8.05 (dd, *J* = 8.2, 1.1 Hz, 2H), 7.96 (dd, *J* = 8.3, 1.2 Hz, 2H), 7.87 (dd, *J* = 8.3, 1.1 Hz, 2H), 7.59 (t, *J* = 7.5 Hz, 1H), 7.54 (t, *J* = 7.2 Hz, 1H), 7.51 (t, *J* = 6.9 Hz, 1H), 7.46 (t, *J* = 7.5 Hz, 1H), 7.44–7.39 (m, 4H), 7.36 (t, *J* = 7.8 Hz, 2H), 7.34–7.26 (m, 7H), 6.18 (s, 1H), 6.06 (t, *J* = 9.5 Hz, 1H), 5.71 (d, *J* = 6.1 Hz, 2H), 5.48 (s, 1H), 5.42 (d, *J* = 8.2 Hz, 1H), 5.08 (s, 2H), 4.78 (d, *J* = 2.1 Hz, 1H), 4.68–4.54 (m, 3H), 4.14 (ddd, *J* = 11.3, 5.1, 2.4 Hz, 1H), 3.69 (s, 3H), 2.79 (dd, *J* = 14.6, 7.1 Hz, 1H), 2.71 (dd, *J* = 14.6, 6.5 Hz, 1H); ^{13}C NMR (125 MHz, $CDCl_3$) δ 172.52, 166.27, 166.10, 165.66, 165.51, 155.82, 139.21, 136.26, 133.63, 133.54, 133.54, 133.48, 133.25, 129.98, 129.91, 129.85, 129.82, 129.53, 129.03, 128.99, 128.67, 128.61, 128.58, 128.27, 128.18, 119.47, 76.62, 71.73, 70.95, 69.71, 67.42, 67.19, 63.36, 52.87, 52.59, 36.24, 29.38; HRMS (ESI) calcd. for $C_{48}H_{43}NO_{13}Na$ $(M + Na)^+$ m/z 864.2627, found 864.2628.

***N*-(9-Fluorenylmethoxycarbonyl)-3-(1-(2,3,4,6-tetra-*O*-benzoyl-α-D-mannopyranosyl)eth-1-en-1-yl)-L-alanine methyl ester** (**3ac**)

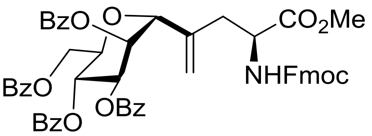

3ac

General Procedure A. White foam, 64.7 mg, 70%. $[\alpha]_D^{25}$ –50.7 (*c* 1.0, $CHCl_3$); ^1H NMR (500 MHz, $CDCl_3$) δ 8.08 (d, *J* = 7.6 Hz, 2H), 8.04 (d, *J* = 7.5 Hz, 2H), 7.96 (d, *J* = 7.2 Hz, 2H), 7.88 (d, *J* = 8.2 Hz, 2H), 7.74 (d, *J* = 7.5 Hz, 2H), 7.61–7.55 (m, 3H), 7.55–7.48 (m, 2H), 7.46 (t, *J* = 7.5 Hz, 1H), 7.42–7.33 (m, 8H), 7.32–7.26 (m, 4H), 6.20 (t, *J* = 2.8 Hz, 1H), 6.07 (t, *J* = 9.5 Hz, 1H), 5.78–5.69 (m, 2H), 5.52–5.47 (m, 2H), 4.81 (s, 1H), 4.70–4.62

(m, 2H), 4.59 (dd, J = 12.3, 5.8 Hz, 1H), 4.40–4.31 (m, 2H), 4.23–4.14(m, 2H), 3.70 (s, 3H), 2.80 (dd, J = 14.7, 7.2 Hz, 1H), 2.73 (dd, J = 14.6, 6.3 Hz, 1H); ^{13}C NMR (125 MHz, $CDCl_3$) δ 172.54, 166.29, 166.11, 165.69, 165.52, 155.85, 143.92, 143.85, 141.38, 139.27, 133.63, 133.54, 133.49, 133.26, 129.97, 129.90, 129.86, 129.82, 129.51, 129.02, 128.98, 128.67, 128.59, 128.58, 128.53, 127.80, 127.19, 125.23, 120.05, 119.48, 76.65, 71.79, 70.94, 69.73, 67.46, 67.25, 63.39, 52.94, 52.63, 47.22, 36.26; HRMS (ESI) calcd. for $C_{55}H_{47}NO_{13}Na$ $(M + Na)^+$ m/z 952.2940, found 952.2941.

***N*-(*Tert*-butoxycarbonyl)-3-(1-(2,3,4,6-tetra-*O*-benzoyl-α-D-mannopyranosyl)eth-1-en-1-yl)-L-alanine benzyl ester** (**3ad**)

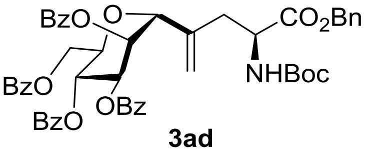

3ad

General Procedure A. White foam, 70.9 mg, 80%. $[\alpha]_D^{25}$ –69.7 (c 1.0, $CHCl_3$); ^{1}H NMR (500 MHz, $CDCl_3$) δ 8.10–8.04 (m, 4H), 7.95 (d, J = 7.3 Hz, 2H), 7.86 (d, J = 7.2 Hz, 2H), 7.58 (dt, J = 15.4, 7.5 Hz, 2H), 7.50 (t, J = 7.4 Hz, 1H), 7.46–7.39 (m, 5H), 7.38–7.33 (m, 3H), 7.32–7.27 (m, J = 8.1, 7.7 Hz, 6H), 6.19 (t, J = 2.6 Hz, 1H), 6.08 (t, J = 9.7 Hz, 1H), 5.70 (dd, J = 9.8, 3.1 Hz, 1H), 5.65 (s, 1H), 5.43 (s, 1H), 5.17–5.11 (m, J = 6.7 Hz, 3H), 4.76 (s, 1H), 4.64 (dd, J = 12.2, 2.7 Hz, 1H), 4.52 (dd, J = 12.1, 5.3 Hz, 1H), 4.11 (ddd, J = 9.6, 5.3, 2.6 Hz, 1H), 2.76 (dd, J = 14.5, 7.0 Hz, 1H), 2.69 (dd, J = 14.5, 6.6 Hz, 1H), 1.42 (s, 9H); ^{13}C NMR (125 MHz, $CDCl_3$) δ 172.20, 166.22, 166.09, 165.56, 165.46, 155.22, 139.07, 135.25, 133.58, 133.51, 133.42, 133.17, 129.96, 129.92, 129.87, 129.83, 129.82, 129.56, 129.02, 128.99, 128.65, 128.54, 128.47, 119.42, 80.19, 76.91, 71.57, 71.07, 69.70, 67.37, 67.30, 63.37, 52.40, 36.14, 28.36; HRMS (ESI) calcd. for $C_{51}H_{49}NO_{13}Na$ $(M + Na)^+$ m/z 906.3096, found 906.3098.

***N*-(*Tert*-butoxycarbonyl)-3-(1-(2,3,4,6-tetra-*O*-benzoyl-α-D-mannopyranosyl)eth-1-en-1-yl)-L-alanine *tert*-butyl ester** (**3ae**)

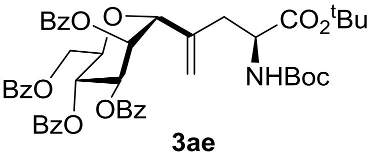

3ae

General Procedure A. White foam, 70 mg, 82%. $[\alpha]_D^{25}$ –58.8 (c 1.0, $CHCl_3$); ^{1}H NMR (500 MHz, $CDCl_3$) δ 8.10 (d, J = 7.3 Hz, 2H), 8.06 (d, J = 7.5 Hz, 2H), 7.95 (d, J = 7.7 Hz, 2H), 7.86 (d, J = 8.1 Hz, 2H), 7.58 (q, J = 7.7 Hz, 2H), 7.50 (t, J = 7.4 Hz, 1H), 7.42 (h, J = 8.8, 8.0 Hz, 5H), 7.35 (t, J = 7.7 Hz, 2H), 7.29 (t, J = 7.7 Hz, 2H), 6.24 (t, J = 2.5 Hz, 1H), 6.11 (t, J = 9.8 Hz, 1H), 5.77–5.70 (m, 2H), 5.51 (s, 1H), 5.12 (d, J = 8.2 Hz, 1H), 4.81 (s, 1H), 4.69 (dd, J = 12.1, 2.5 Hz, 1H), 4.55 (dd, J = 11.9, 4.7 Hz, 1H), 4.43 (q, J = 7.2 Hz, 1H), 4.14 (ddd, J = 10.0, 5.2, 2.6 Hz, 1H), 2.78 (dd, J = 14.6, 6.3 Hz, 1H), 2.67 (dd, J = 14.4, 7.0 Hz, 1H), 1.43 (s, 18H); ^{13}C NMR (125 MHz, $CDCl_3$) δ 171.42, 166.27, 166.18, 165.55, 165.47, 155.19, 139.51, 133.56, 133.47, 133.42, 133.17, 129.93, 129.89, 129.87, 129.84, 129.60, 129.07, 129.02, 128.64, 128.55, 128.48, 119.32, 82.62, 79.90, 76.71, 71.46, 71.20, 69.84, 67.34, 63.47, 52.68, 36.47, 28.38, 27.94; HRMS (ESI) calcd. for $C_{48}H_{39}NO_{13}Na$ $(M + Na)^+$ m/z 872.3253, found 871.3258.

***N,N*-Phthaloyl-3-(1-(2,3,4,6-tetra-*O*-benzoyl-α-D-mannopyranosyl)eth-1-en-1-yl)-L-alanine methyl ester** (**3af**)

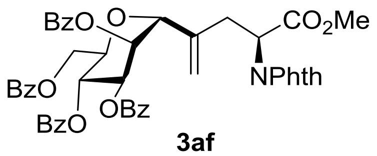

3af

General Procedure A. White foam, 61.2 mg, 73%. $[\alpha]_D^{25}$ –82.0 (*c* 1.0, $CHCl_3$); ^{1}H NMR (500 MHz, $CDCl_3$) δ 8.07 (d, *J* = 7.0 Hz, 2H), 8.02 (d, *J* = 6.9 Hz, 2H), 7.98 (d, *J* = 7.0 Hz, 2H), 7.87 (d, *J* = 7.0 Hz, 2H), 7.80 (dt, *J* = 5.5, 2.8 Hz, 2H), 7.68 (dt, *J* = 5.6, 2.8 Hz, 2H), 7.59 (t, *J* = 7.4 Hz, 1H), 7.57–7.49 (m, 2H), 7.46 (t, *J* = 7.4 Hz, 2H), 7.43–7.35 (m, 5H), 7.30 (t, *J* = 7.1 Hz, 2H), 6.14 (q, *J* = 2.7 Hz, 1H), 6.06 (t, *J* = 9.0 Hz, 1H), 5.71 (d, *J* = 9.5 Hz, 1H), 5.66 (s, 1H), 5.47 (s, 1H), 5.24 (ddd, *J* = 10.4, 4.9, 2.3 Hz, 1H), 4.74 (s, 1H), 4.63 (dt, *J* = 12.2, 2.6 Hz, 1H), 4.57 (ddd, *J* = 12.2, 5.4, 2.4 Hz, 1H), 4.22 (ddt, *J* = 8.4, 5.3, 2.6 Hz, 1H), 3.70 (s, 3H), 3.39–3.22 (m, 2H); ^{13}C NMR (125 MHz, $CDCl_3$) δ 169.28, 167.76, 166.29, 166.03, 165.63, 165.46, 139.43, 134.29, 133.60, 133.50, 133.45, 133.10, 131.79, 129.96, 129.94, 129.90, 129.86, 129.52, 129.04, 128.99, 128.63, 128.58, 128.52, 128.50, 123.72, 118.24, 77.32, 71.60, 70.93, 69.72, 67.32, 63.29, 53.10, 50.86, 32.57; HRMS (ESI) calcd. for $C_{48}H_{39}NO_{13}Na$ $(M + Na)^+$ m/z 860.2314, found 860.2316.

***N*-(*Tert*-butoxycarbonyl)-*O*-(2-(2,3,4,6-tetra-*O*-benzoyl-α-D-mannopyranosyl) prop-2-en-1-yl)-L-serine methyl ester (3ag)**

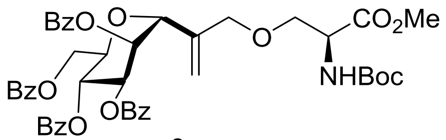

3ag

General Procedure A. White foam, 59.8 mg, 71%. $[\alpha]_D^{25}$ –37.0 (*c* 1.2, $CHCl_3$); ^{1}H NMR (500 MHz, $CDCl_3$) δ 8.08 (t, *J* = 8.8 Hz, 4H), 7.96 (d, *J* = 7.9 Hz, 2H), 7.87 (d, *J* = 7.9 Hz, 2H), 7.59 (q, *J* = 7.3, 6.8 Hz, 2H), 7.51 (t, *J* = 7.1 Hz, 1H), 7.47–7.4 (m, 5H), 7.36 (t, *J* = 7.0 Hz, 2H), 7.29 (t, *J* = 7.0 Hz, 2H), 6.17 (s, 1H), 6.09 (t, *J* = 10.5 Hz, 1H), 5.74–5.68 (m, 2H), 5.64–5.58 (m, 2H), 4.79 (s, 1H), 4.64 (d, *J* = 11.9 Hz, 1H), 4.55 (ddd, *J* = 12.0, 5.6, 1.9 Hz, 1H), 4.46 (d, *J* = 7.5 Hz, 1H), 4.21 (ddt, *J* = 10.1, 5.0, 2.1 Hz, 1H), 4.16 (d, *J* = 12.8 Hz, 1H), 4.10 (d, *J* = 12.6 Hz, 1H), 3.87 (d, *J* = 9.0 Hz, 1H), 3.71 (s, 3H), 1.43 (s, 9H); ^{13}C NMR (125 MHz, $CDCl_3$) δ 171.07, 166.24, 166.15, 165.65, 165.55, 155.71, 139.66, 133.62, 133.54, 133.46, 133.29, 129.98, 129.91, 129.87, 129.82, 129.57, 129.03, 129.01, 128.67, 128.59, 128.49, 117.48, 80.05, 75.87, 71.87, 71.46, 71.17, 70.04, 67.44, 63.47, 54.07, 52.59, 28.42; HRMS (ESI) calcd. for $C_{46}H_{47}NO_{14}Na$ $(M + Na)^+$ m/z 860.2889, found 860.2893.

***N*-(*Tert*-butoxycarbonyl)-*O*-(2-(2,3,4,6-tetra-*O*-benzoyl-α-D-mannopyranosyl) prop-2-en-1-yl)-L-tyrosine methyl ester (3ah)**

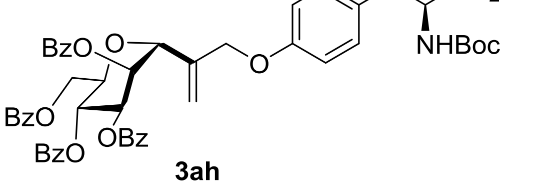

3ah

General Procedure A. White foam, 81.5 mg, 89%. $[\alpha]_D^{25}$ –32.9 (*c* 1.3, $CHCl_3$); ^{1}H NMR (500 MHz, $CDCl_3$) δ 8.07 (td, *J* = 8.2, 1.4 Hz, 4H), 7.97 (dd, *J* = 8.4, 1.2 Hz, 2H), 7.88 (dd, *J* = 8.3, 1.2 Hz, 2H), 7.62–7.55 (m, 2H), 7.51 (t, *J* = 7.4 Hz, 1H), 7.47–7.41 (m, 3H), 7.40–7.35 (m, 4H), 7.30 (t, *J* = 7.8 Hz, 2H), 6.99 (d, *J* = 8.5 Hz, 2H), 6.84 (d, *J* = 8.6 Hz, 2H), 6.26 (dd, *J* = 3.1, 2.1 Hz, 1H), 6.09 (t, *J* = 9.6 Hz, 1H), 5.88 (d, *J* = 10.4 Hz, 2H), 5.84 (dd, *J* = 9.7, 3.1 Hz, 1H), 4.99 (d, *J* = 8.3 Hz, 1H), 4.96 (s, 1H), 4.68–

4.56 (m, 4H), 4.58–4.51 (m, 1H), 4.28 (ddd, *J* = 9.0, 5.8, 2.8 Hz, 1H), 3.70 (s, 3H), 3.02 (qd, *J* = 14.0, 5.9 Hz, 2H), 1.42 (s, 9H); ^{13}C NMR (125 MHz, $CDCl_3$) *δ* 172.49, 166.24, 166.10, 165.64, 165.53, 157.53, 155.21, 139.16, 133.63, 133.55, 133.46, 133.26, 130.44, 129.96, 129.89, 129.84, 129.80, 129.75, 129.51, 128.98, 128.97, 128.66, 128.58, 128.57, 128.49, 118.43, 114.91, 80.02, 75.87, 71.71, 71.10, 69.83, 68.14, 67.53, 63.46, 54.61, 52.30, 37.55, 28.41; HRMS (ESI) calcd. for $C_{52}H_{51}NO_{14}Na$ $(M + Na)^+$ m/z 936.3202, found 936.3203.

***N*²-(9-Fluorenylmethoxycarbonyl)-*N*⁶-(6-(2,3,4,6-tetra-*O*-benzoyl-α-D-mannopyranosyl)hept-6-enoyl)-L-lysine methyl ester** (**3ai**)

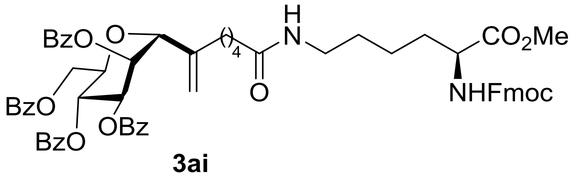

3ai

General Procedure A (at 25 ℃). White foam, 75 mg, 70%. $[\alpha]_D^{25}$ –39.5 (*c* 1.5, $CHCl_3$); ^{1}H NMR (500 MHz, $CDCl_3$) *δ* 8.07 (ddd, *J* = 13.1, 8.3, 1.2 Hz, 4H), 7.96 (dd, *J* = 8.3, 1.2 Hz, 2H), 7.86 (dd, *J* = 8.3, 1.2 Hz, 2H), 7.75 (d, *J* = 7.5 Hz, 2H), 7.62–7.54 (m, 4H), 7.50 (t, *J* = 7.4 Hz, 1H), 7.47–7.33 (m, 9H), 7.29 (q, *J* = 7.4 Hz, 4H), 6.20 (dd, *J* = 3.2, 1.9 Hz, 1H), 6.09 (t, *J* = 9.8 Hz, 1H), 5.73 (dd, *J* = 9.9, 3.1 Hz, 1H), 5.69 (t, *J* = 5.7 Hz, 1H), 5.57 (s, 1H), 5.47 (d, *J* = 8.1 Hz, 1H), 5.38 (s, 1H), 4.66 (dd, *J* = 12.1, 2.6 Hz, 1H), 4.51 (dd, *J* = 12.0, 5.5 Hz, 1H), 4.44–4.31 (m, 3H), 4.21 (t, *J* = 7.0 Hz, 1H), 4.15 (ddd, *J* = 9.7, 5.6, 2.7 Hz, 1H), 3.74 (s, 3H), 3.22 (q, *J* = 6.9 Hz, 2H), 2.32-2.26 (m, *J* = 14.6, 9.2, 5.3 Hz, 1H), 2.24–2.11 (m, 3H), 1.89–1.79 (m, 1H), 1.73–1.45 (m, 7H), 1.44–1.30 (m, 2H); ^{13}C NMR (125 MHz, $CDCl_3$) *δ* 173.00, 166.32, 166.17, 165.81, 165.57, 156.18, 143.97, 143.80, 143.31, 141.39, 133.60, 133.54, 133.44, 133.28, 129.93, 129.89, 129.86, 129.84, 129.82, 129.60, 129.05, 129.02, 128.67, 128.58, 128.49, 127.83, 127.18, 125.20, 125.17, 120.10, 115.51, 77.01, 71.29, 71.25, 70.29, 67.53, 67.12, 63.54, 53.71, 52.55, 47.26, 39.06, 36.42, 33.04, 32.24, 29.16, 27.07, 25.40, 22.54; HRMS (ESI) calcd. for $C_{63}H_{62}N_2O_{14}Na$ $(M + Na)^+$ m/z 1093.4093, found 1093.4095.

***N*²-(*Tert*-butoxycarbonyl)-*N*⁴-(2-(2,3,4,6-tetra-*O*-benzoyl-α-D-mannopyranosyl) prop-2-en-1-yl)-L-asparagine methyl ester** (**3aj**)

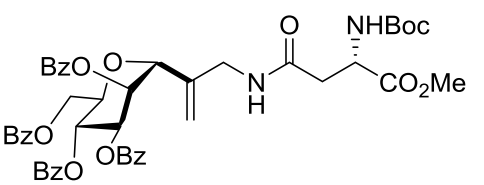

3aj

General Procedure A (at 25 ℃). Colorless oil, 50 mg, 58%. $[\alpha]_D^{25}$ –33.3 (*c* 1.0, $CHCl_3$); ^{1}H NMR (500 MHz, $CDCl_3$) *δ* 8.10 (d, *J* = 7.3 Hz, 2H), 8.04 (d, *J* = 7.2 Hz, 2H), 7.98 (d, *J* = 7.3 Hz, 2H), 7.87 (d, *J* = 7.3 Hz, 2H), 7.60–7.57 (m, 3H), 7.52 (t, *J* = 7.4 Hz, 1H), 7.48-7..36 (m, 7H), 7.30 (t, *J* = 7.8 Hz, 2H), 6.26 (t, *J* = 6.2 Hz, 1H), 6.16 (t, *J* = 2.7 Hz, 1H), 6.10 (t, *J* = 9.6 Hz, 1H), 5.82 (d, *J* = 8.5 Hz, 1H), 5.72 (dd, *J* = 9.9, 3.4 Hz, 1H), 5.70 (s, 1H), 5.57 (s, 1H), 4.77 (s, 1H), 4.66 (dd, *J* = 12.1, 2.8 Hz, 1H), 4.59 (dd, *J* = 12.1, 5.3 Hz, 1H), 4.57–4.52 (m, 1H), 4.30–4.19 (m, 2H), 3.85 (dd, *J* = 16.4, 5.5 Hz, 1H), 3.74 (s, 3H), 2.96 (dd, *J* = 15.7, 3.5 Hz, 1H), 2.75 (dd, *J* = 15.7, 4.5 Hz, 1H), 1.44 (s, 9H); ^{13}C NMR (125 MHz, $CDCl_3$) *δ* 172.04, 170.24, 166.36, 166.19, 165.77, 165.54, 155.81, 139.96, 133.68, 133.63, 133.54, 133.31, 129.97, 129.94, 129.89, 129.87, 129.79, 129.41, 128.99,

128.92, 128.69, 128.62, 128.55, 116.63, 80.16, 76.25, 71.71, 71.12, 69.80, 67.29, 63.28, 52.81, 50.53, 41.75, 38.05, 28.44; HRMS (ESI) calcd. for $C_{47}H_{48}N_2O_{14}Na$ (M + Na)$^+$ m/z 887.2998, found 887.3000.

***N*-(*Tert*-butoxycarbonyl)-3-(1-(2,3,4,6-tetra-*O*-benzoyl-α-D-mannopyranosyl)eth-1-en-1-yl)-L-alanyl-L-phenylalanine methyl ester** (**3ak**)

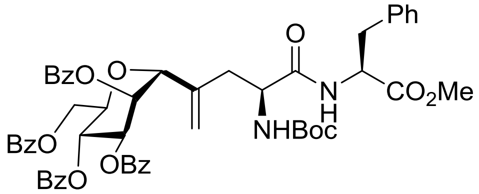

3ak

General Procedure A. White foam, 61.3 mg, 64%. $[\alpha]_D^{25}$ –39.9 (*c* 0.9, $CHCl_3$); ^{1}H NMR (500 MHz, $CDCl_3$) δ 8.09 (d, *J* = 7.2 Hz, 2H), 8.04 (d, *J* = 7.3 Hz, 2H), 7.98 (d, *J* = 7.2 Hz, 2H), 7.87 (d, *J* = 8.4 Hz, 2H), 7.58 (q, *J* = 7.1, 6.3 Hz, 2H), 7.51 (t, *J* = 7.4 Hz, 1H), 7.48–7.35 (m, 7H), 7.30 (t, *J* = 7.8 Hz, 2H), 7.25 (m, 2H), 7.20 (t, *J* = 7.3 Hz, 1H), 7.12 (d, *J* = 6.9 Hz, 2H), 6.82 (d, *J* = 7.9 Hz, 1H), 6.18 (s, 1H), 6.10 (t, *J* = 9.5 Hz, 1H), 5.70 (dd, *J* = 9.6, 3.1 Hz, 1H), 5.65 (s, 1H), 5.43 (s, 1H), 5.08 (d, *J* = 8.2 Hz, 1H), 4.87–4.80 (m, 2H), 4.80 (s, 1H), 4.68 (dd, *J* = 12.1, 2.9 Hz, 1H), 4.54 (dd, *J* = 12.1, 5.2 Hz, 1H), 4.41 (s, 1H), 4.23 (p, *J* = 3.4 Hz, 1H), 3.65 (s, 3H), 3.14 (dd, *J* = 13.9, 5.8 Hz, 1H), 3.07 (dd, *J* = 13.8, 6.5 Hz, 1H), 2.83–2.73 (m, 1H), 2.53 (dd, *J* = 15.3, 8.4 Hz, 1H), 1.40 (s, 9H); ^{13}C NMR (125 MHz, $CDCl_3$) δ 171.58, 171.06, 166.29, 166.07, 165.75, 165.51, 155.53, 139.21, 135.94, 133.59, 133.54, 133.46, 133.19, 129.98, 129.94, 129.87, 129.86, 129.50, 129.35, 129.06, 129.02, 128.67, 128.64, 128.58, 128.50, 127.20, 118.51, 80.30, 77.26, 71.56, 70.94, 69.83, 67.41, 63.27, 53.50, 53.19, 52.39, 38.04, 36.23, 28.34; HRMS (ESI) calcd. for $C_{54}H_{54}N_2O_{14}Na$ (M + Na)$^+$ m/z 977.3467, found 977.3471.

***N*-(*Tert*-butoxycarbonyl)-3-(1-(2,3,4,6-tetra-*O*-benzoyl-α-D-mannopyranosyl)eth-1-en-1-yl)-L-alanyl-L-leucine methyl ester** (**3al**)

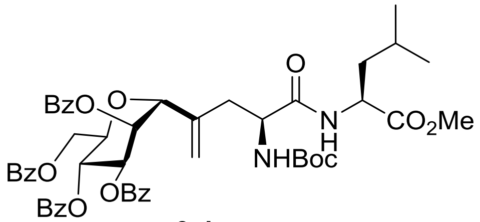

3al

General Procedure A. White foam, 57.7 mg, 63%. $[\alpha]_D^{25}$ –74.7 (*c* 1.0, $CHCl_3$); ^{1}H NMR (400 MHz, $CDCl_3$) δ 8.10 (d, *J* = 7.7 Hz, 2H), 8.05 (d, *J* = 7.7 Hz, 2H), 7.98 (d, *J* = 7.5 Hz, 2H), 7.87 (d, *J* = 7.6 Hz, 2H), 7.58 (q, *J* = 7.4 Hz, 2H), 7.51 (t, *J* = 7.4 Hz, 1H), 7.48–7.34 (m, 7H), 7.30 (t, *J* = 7.7 Hz, 2H), 6.82 (d, *J* = 8.3 Hz, 1H), 6.21 (s, 1H), 6.11 (t, *J* = 9.6 Hz, 1H), 5.72 (dd, *J* = 9.6, 3.1 Hz, 1H), 5.67 (s, 1H), 5.47 (s, 1H), 5.14 (d, *J* = 8.4 Hz, 1H), 4.85 (s, 1H), 4.71 (dd, *J* = 12.4, 2.9 Hz, 1H), 4.63–4.55(m, 2H), 4.48–4.39 (m, 1H), 4.34–4.24 (m, 1H), 3.67 (s, 3H), 2.85 (dd, *J* = 15.1, 5.8 Hz, 1H), 2.56 (dd, *J* = 15.1, 8.4 Hz, 1H), 1.72–1.55 (m, 3H), 1.40 (s, 9H), 0.91 (d, *J* = 6.0 Hz, 6H); ^{13}C NMR (125 MHz, $CDCl_3$) δ 173.01, 171.26, 166.34, 166.09, 165.89, 165.54, 155.70, 139.13, 133.60, 133.51, 133.46, 133.20, 129.99, 129.95, 129.91, 129.89, 129.87, 129.49, 129.06, 129.03, 128.66, 128.57, 128.50, 118.46, 80.30, 71.53, 70.97, 70.03, 67.44, 63.35, 53.02, 52.37, 50.91, 41.28, 36.28, 28.32, 24.83, 22.98, 21.84; HRMS (ESI) calcd. for $C_{31}H_{56}N_2O_{14}Na$ (M + Na)$^+$ m/z 943.3624, found 943.3625.

N-($Tert$-butoxycarbonyl)-3-(1-(2,3,4,6-tetra-O-benzoyl-α-D-mannopyranosyl)eth-1-en-1-yl)-L-alanyl-L-isoleucine methyl ester (**3am**)

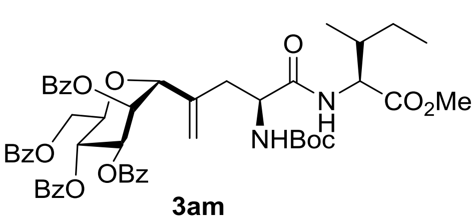

3am

General Procedure A. White foam, 57 mg, 62%. $[\alpha]_D^{25}$ –93.1 (c 1.0, $CHCl_3$); ^1H NMR (500 MHz, $CDCl_3$) δ 8.10 (d, J = 7.4 Hz, 2H), 8.05 (d, J = 7.4 Hz, 2H), 7.98 (d, J = 7.2 Hz, 2H), 7.87 (d, J = 7.2 Hz, 2H), 7.62–7.54 (m, 2H), 7.41 (m, 4H), 7.30 (t, J = 7.8 Hz, 2H), 6.93 (d, J = 8.7 Hz, 2H), 6.20 (s, 1H), 6.11 (t, J = 9.5 Hz, 1H), 5.73 (dd, J = 9.7, 3.1 Hz, 1H), 5.66 (s, 1H), 5.46 (s, 1H), 5.11 (d, J = 8.3 Hz, 1H), 4.84 (s, 1H), 4.69 (dd, J = 12.2, 2.8 Hz, 1H), 4.61–4.53 (m, 2H), 4.49–4.39 (m, 1H), 4.29 (s, 1H), 3.67 (s, 3H), 2.84 (dd, J = 15.3, 5.9 Hz, 1H), 2.56 (dd, J = 15.2, 8.2 Hz, 1H), 1.91 (tdt, J = 9.0, 6.8, 3.4 Hz, 1H), 1.41 (s, 10H), 1.20 (tdt, J = 16.5, 9.2, 4.2 Hz, 1H), 0.88 (m, 6H); ^{13}C NMR (125 MHz, $CDCl_3$) δ 171.97, 171.20, 166.29, 166.04, 165.84, 165.53, 155.69, 139.25, 133.57, 133.43, 133.17, 129.97, 129.93, 129.87, 129.85, 129.50, 129.05, 129.03, 128.65, 128.55, 128.48, 118.21, 80.29, 77.30, 77.16, 71.56, 70.91, 70.00, 67.42, 63.37, 56.70, 53.08, 52.14, 37.79, 35.94, 28.32, 25.03, 15.59, 11.65; HRMS (ESI) calcd. for $C_{51}H_{56}N_2O_{14}Na$ $(M + Na)^+$ m/z 943.3624, found 943.3627.

N-($Tert$-butoxycarbonyl)-3-(1-(2,3,4,6-tetra-O-benzoyl-α-D-mannopyranosyl)eth-1-en-1-yl)-L-alanyl-L-proline methyl ester (**3an**)

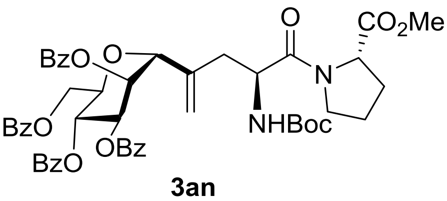

3an

General Procedure A. White foam, 57.4 mg, 63%. $[\alpha]_D^{25}$ –64.4 (c 1.0, $CHCl_3$); ^1H NMR (400 MHz, $CDCl_3$) δ 8.13 (d, J = 7.3 Hz, 2H), 8.04 (d, J = 7.3 Hz, 2H), 7.97 (d, J = 7.3 Hz, 2H), 7.87 (d, J = 7.2 Hz, 2H), 7.58 (q, J = 6.6, 5.8 Hz, 2H), 7.51 (t, J = 7.4 Hz, 1H), 7.48–7.42 (m, 3H), 7.40–7.34 (m, 4H), 7.30 (t, J = 7.8 Hz, 2H), 6.23 (t, J = 2.5 Hz, 1H), 6.16 (t, J = 9.8 Hz, 1H), 5.78 (s, 1H), 5.72 (dd, J = 9.8, 3.1 Hz, 1H), 5.55 (s, 1H), 5.29 (d, J = 9.1 Hz, 1H), 4.85 (s, 1H), 4.76 (dd, J = 12.3, 2.5 Hz, 1H), 4.74–4.66 (m, 1H), 4.54–4.47 (m, 2H), 4.34–4.27 (m, 1H), 3.82 (dt, J = 10.6, 6.7 Hz, 1H), 3.78–3.72 (m, 1H), 3.66 (s, 3H), 2.74 (dd, J = 14.5, 5.1 Hz, 1H), 2.59 (dd, J = 14.7, 8.7 Hz, 1H), 2.23-2.18 (m, 1H), 2.10–1.93 (m, 3H), 1.40 (s, 9H); ^{13}C NMR (125 MHz, $CDCl_3$) δ 172.32, 170.63, 166.26, 166.18, 165.63, 165.48, 155.46, 138.74, 133.56, 133.41, 133.16, 130.04, 129.98, 129.91, 129.90, 129.86, 129.66, 129.15, 129.09, 128.60, 128.55, 128.50, 120.00, 79.90, 77.67, 71.33, 71.26, 69.97, 67.36, 63.29, 59.11, 52.33, 51.07, 47.26, 36.56, 29.16, 28.40, 25.08; HRMS (ESI) calcd. for $C_{50}H_{52}N_2O_{14}Na$ $(M + Na)^+$ m/z 927.3311, found 927.3315.

N-($Tert$-butoxycarbonyl)-L-tryptophanyl-3-(1-(2,3,4,6-tetra-O-benzoyl-α-D-mannopyranosyl)eth-1-en-1-yl)-L-alanine methyl ester (**3ao**)

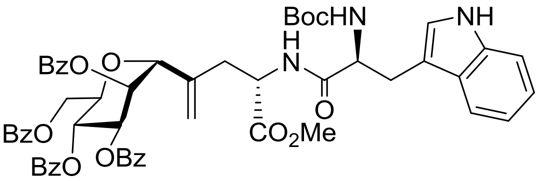

3ao

White foam, 66.6 mg, 67%. $[\alpha]_D^{25}$ –65.1 (*c* 1.1, $CHCl_3$); ^1H NMR (500 MHz, $CDCl_3$) δ 8.84–8.77 (m, 1H), 8.08 (t, *J* = 7.3 Hz, 4H), 7.95 (d, *J* = 7.6 Hz, 2H), 7.89 (d, *J* = 7.5 Hz, 2H), 7.76 (d, *J* = 7.9 Hz, 1H), 7.59 (dt, *J* = 16.2, 7.4 Hz, 2H), 7.48 (dt, *J* = 15.9, 7.4 Hz, 2H), 7.44–7.38 (m, 5H), 7.35 (t, *J* = 7.8 Hz, 2H), 7.31 (t, *J* = 7.7 Hz, 2H), 7.19 (t, *J* = 7.5 Hz, 1H), 7.13 (t, *J* = 7.2 Hz, 2H), 6.21 (d, *J* = 7.8 Hz, 1H), 6.12 (t, *J* = 2.5 Hz, 1H), 6.08 (t, *J* = 9.7 Hz, 1H), 5.62 (dd, *J* = 9.8, 3.1 Hz, 1H), 5.34 (s, 1H), 5.06 (s, 1H), 4.70–4.62 (m, 2H), 4.61 (s, 1H), 4.53 (s, 1H), 4.50 (dd, *J* = 12.1, 5.3 Hz, 1H), 4.03 (ddd, *J* = 8.8, 5.2, 2.6 Hz, 1H), 3.61 (s, 3H), 3.42 (d, *J* = 14.4 Hz, 1H), 3.10 (dd, *J* = 14.5, 8.2 Hz, 1H), 2.70–2.64 (m, 1H), 2.46 (dd, *J* = 14.6, 6.3 Hz, 1H), 1.45 (s, 9H); ^{13}C NMR (125 MHz, $CDCl_3$) δ 172.01, 171.54, 166.32, 166.17, 166.08, 165.49, 155.60, 138.51, 136.49, 133.75, 133.65, 133.57, 133.34, 130.05, 129.86, 129.80, 129.70, 129.30, 128.94, 128.86, 128.72, 128.61, 128.59, 128.55, 127.33, 124.07, 122.35, 119.83, 119.70, 119.03, 111.56, 110.28, 80.07, 76.48, 71.46, 70.96, 70.00, 67.27, 63.31, 54.83, 52.50, 50.57, 35.41, 28.84, 28.43; HRMS (ESI) calcd. for $C_{56}H_{55}N_3O_{14}Na$ (M + Na)$^+$ m/z 1016.3576, found 1016.3579.

***O*-Benzyl-*N*-(*tert*-butoxycarbonyl)-L-threonyl-3-(1-(2,3,4,6-tetra-*O*-benzoyl-α-D-mannopyranosyl)eth-1-en-1-yl)-L-alanine methyl ester** (**3ap**)

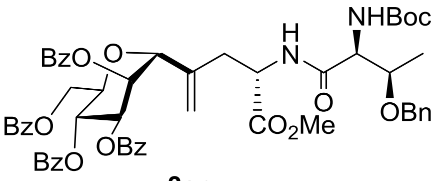

3ap

General Procedure A. White foam, 80 mg, 80%. $[\alpha]_D^{25}$ –50.5 (*c* 1.4, $CHCl_3$); ^1H NMR (500 MHz, $CDCl_3$) *δ* 8.09–8.04 (m, 4H), 7.95 (d, *J* = 7.3 Hz, 2H), 7.87 (d, *J* = 7.3 Hz, 2H), 7.63–7.54 (m, 2H), 7.50 (t, *J* = 7.4 Hz, 1H), 7.48–7.39 (m, 5H), 7.38–7.34 (m, 2H), 7.29 (m, 7H), 7.25–7.21 (m, 1H), 6.10 (t, *J* = 2.7 Hz, 1H), 6.04 (t, *J* = 9.6 Hz, 1H), 5.67 (dd, *J* = 9.7, 3.1 Hz, 1H), 5.54 (d, *J* = 7.1 Hz, 1H), 5.52 (s, 1H), 5.33 (s, 1H), 4.77 (q, *J* = 7.1 Hz, 1H), 4.71 (d, *J* = 2.1 Hz, 1H), 4.68–4.59 (m, 2H), 4.53 (m, 2H), 4.33 (dd, *J* = 6.9, 3.2 Hz, 1H), 4.19–4.05 (m, 2H), 3.71 (dd, *J* = 8.6, 4.8 Hz, 1H), 3.66 (s, 3H), 2.70 (dd, *J* = 14.7, 7.4 Hz, 1H), 2.64 (dd, *J* = 14.7, 7.0 Hz, 1H), 1.45 (s, 9H), 1.19 (d, *J* = 6.4 Hz, 3H); ^{13}C NMR (125 MHz, $CDCl_3$) δ 171.92, 169.96, 166.22, 165.99, 165.50, 165.45, 155.77, 139.00, 137.95, 133.57, 133.49, 133.41, 133.22, 129.93, 129.84, 129.79, 129.74, 129.53, 129.08, 128.96, 128.62, 128.54, 128.51, 128.46, 128.28, 127.93, 127.88, 80.13, 76.52, 74.99, 71.59, 71.52, 70.84, 69.65, 67.36, 63.39, 57.35, 52.43, 51.32, 35.78, 28.38, 14.82; HRMS (ESI) calcd. for $C_{56}H_{58}N_2O_{15}Na$ (M + Na)$^+$ m/z 1021.3729, found 1021.3733.

***N*-(*Tert*-butoxycarbonyl)-L-phenylalanyl--3-(1-(2,3,4,6-tetra-*O*-benzoyl-α-D-mannopyranosyl)eth-1-en-1-yl)-L-alanine methyl ester** (**3aq**)

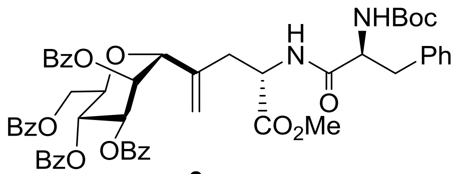

3aq

White foam, 65.9 mg, 69%. $[\alpha]_D^{25}$ –51.8 (*c* 1.1, $CHCl_3$); ^1H NMR (500 MHz, $CDCl_3$) δ 8.09 (d, *J* = 7.3 Hz, 2H), 8.04 (d, *J* = 7.4 Hz, 2H), 7.96 (d, *J* = 7.3 Hz, 2H), 7.87 (d, *J* = 7.3 Hz, 2H), 7.58 (t, *J* = 7.4 Hz, 2H), 7.51 (t, *J* = 7.4 Hz, 1H), 7.50–7.35 (m, 7H), 7.30 (t, *J* = 7.8 Hz, 3H), 7.24 (d, *J* = 7.1 Hz, 2H), 7.20 (t, *J* = 5.9 Hz, 3H), 6.58 (d, *J* = 7.6 Hz, 1H), 6.13 (t, *J* = 2.7 Hz, 1H), 6.08 (t, *J* = 9.6 Hz, 1H), 5.70 (dd, *J* = 9.7, 3.1 Hz, 1H), 5.66 (d, *J* = 1.9 Hz, 1H), 5.42 (s, 1H), 5.19–5.09 (m, 1H), 4.78 (q, *J* = 7.0 Hz, 1H), 4.71 (s, 1H), 4.67 (dd, *J* = 12.0, 2.7 Hz, 1H), 4.56 (dd, *J* = 12.1, 5.3 Hz, 1H), 4.44–4.32 (m, 1H), 4.17–4.09 (m, 1H), 3.66 (s, 3H), 3.12 (dd, *J* = 13.9, 6.3 Hz, 1H), 3.01 (d, *J* = 10.1 Hz, 1H), 2.77-2.66 (m, 2H), 1.37 (s, 9H); ^{13}C NMR (125 MHz, $CDCl_3$) δ 171.85, 171.26, 166.28, 166.10, 165.72, 165.48, 155.52, 139.03, 136.77, 133.63, 133.58, 133.49, 133.30, 129.96, 129.89, 129.84, 129.74, 129.45, 129.43, 128.96, 128.92, 128.69, 128.66, 128.61, 128.58, 128.52, 126.99, 119.51, 80.24, 76.58, 71.65, 70.91, 69.80, 67.27, 63.32, 55.93, 52.61, 51.22, 38.34, 35.83, 28.31; HRMS (ESI) calcd. for $C_{54}H_{54}N_2O_{14}Na$ $(M + Na)^+$ m/z 977.3467, found 977.3470.

***N*-(*Tert*-butoxycarbonyl)-3-(1-(2,3,4,6-tetra-*O*-benzoyl-α-D-mannopyranosyl)eth-1-en-1-yl)-L-alanyl-L-alanyl-L-valine methyl ester** (**3ar**)

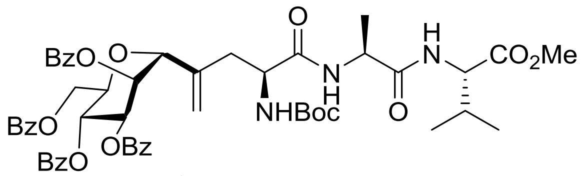

3ar

General Procedure A. White foam, 54.5 mg, 56%. $[\alpha]_D^{25}$ –58.3 (*c* 1.0, $CHCl_3$); ^1H NMR (500 MHz, $CDCl_3$) *δ* 8.10 (d, *J* = 7.3 Hz, 2H), 8.03 (d, *J* = 7.4 Hz, 2H), 7.98 (d, *J* = 7.4 Hz, 2H), 7.87 (d, *J* = 7.3 Hz, 2H), 7.58 (q, *J* = 7.3 Hz, 2H), 7.51 (t, *J* = 7.4 Hz, 1H), 7.41 (ddt, *J* = 29.9, 15.5, 7.6 Hz, 7H), 7.30 (t, *J* = 7.8 Hz, 2H), 7.05 (s, 1H), 6.83 (d, *J* = 8.6 Hz, 1H), 6.21 (s, 1H), 6.11 (t, *J* = 9.5 Hz, 1H), 5.71 (dd, *J* = 9.6, 3.0 Hz, 1H), 5.68 (s, 1H), 5.48 (s, 1H), 5.24 (d, *J* = 7.5 Hz, 1H), 4.83 (s, 1H), 4.73 (dd, *J* = 12.2, 2.9 Hz, 1H), 4.57 (dd, *J* = 12.1, 5.0 Hz, 1H), 4.54–4.48 (m, 2H), 4.44 (br, 1H), 4.30 (ddd, *J* = 8.6, 4.8, 2.9 Hz, 1H), 3.70 (s, 3H), 2.84 (dd, *J* = 15.2, 5.0 Hz, 1H), 2.58 (dd, *J* = 15.2, 9.0 Hz, 1H), 2.13 (dq, *J* = 13.7, 6.8 Hz, 1H), 1.40 (s, 12H), 0.90 (d, *J* = 6.9 Hz, 3H), 0.87 (d, *J* = 6.9 Hz, 3H); ^{13}C NMR (125 MHz, $CDCl_3$) *δ* 172.30, 171.83, 171.60, 166.34, 166.09, 165.82, 165.51, 155.75, 139.18, 133.61, 133.59, 133.48, 133.23, 129.96, 129.93, 129.88, 129.85, 129.42, 129.05, 128.98, 128.65, 128.57, 128.51, 118.60, 80.47, 71.58, 70.97, 69.83, 67.41, 63.20, 57.27, 53.27, 52.24, 49.33, 31.31, 28.41, 28.30, 19.03, 17.87, 17.76; HRMS (ESI) calcd. for $C_{53}H_{59}N_3O_{15}Na$ $(M + Na)^+$ m/z 1000.3838, found 1000.3841.

***N*-(*Tert*-butoxycarbonyl)-3-(1-(2,3,4,6-tetra-*O*-benzoyl-α-D-mannopyranosyl)eth-1-en-1-yl)-L-alanyl-L-tertleucyl-L-alanine methyl ester** (**3as**)

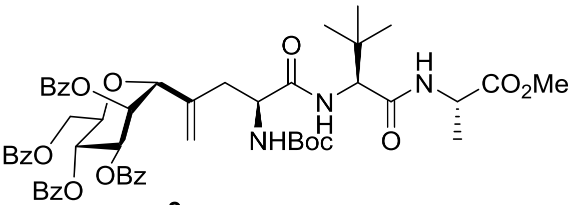

3as

General Procedure A. White foam, 61.8 mg, 62%. [α]$_D^{25}$ –54.7 (*c* 1.0, $CHCl_3$); ^{1}H NMR (500 MHz, $CDCl_3$) *δ* 8.10 (d, *J* = 7.2 Hz, 2H), 8.05 (d, *J* = 7.4 Hz, 2H), 7.98 (d, *J* = 7.2 Hz, 2H), 7.86 (d, *J* = 7.2 Hz, 2H), 7.58 (q, *J* = 7.6 Hz, 2H), 7.51 (t, *J* = 7.4 Hz, 1H), 7.48–7.33 (m, 7H), 7.29 (t, *J* = 7.8 Hz, 2H), 7.12 (d, *J* = 8.7 Hz, 1H), 6.59 (d, *J* = 7.3 Hz, 1H), 6.21 (s, 1H), 6.11 (t, *J* = 9.5 Hz, 1H), 5.72 (dd, *J* = 9.7, 3.0 Hz, H), 5.66 (s, 1H), 5.46 (d, *J* = 1.6 Hz, 1H), 5.26 (d, *J* = 8.1 Hz, H), 4.83 (s, 1H), 4.71 (dd, *J* = 12.2, 2.9 Hz, 1H), 4.61–4.53 (m, 2H), 4.47 (q, *J* = 7.6 Hz, 1H), 4.32–4.25 (m, 2H), 3.69 (s, 3H), 2.82 (dd, *J* = 15.3, 5.8 Hz, 1H), 2.61 (dd, *J* = 15.2, 8.6 Hz, 1H), 1.40 (s, 9H), 1.37 (d, *J* = 7.2 Hz, 3H), 1.02 (s, 9H); ^{13}C NMR (125 MHz, $CDCl_3$) *δ* 173.30, 171.36, 169.68, 166.28, 166.09, 165.76, 165.50, 155.72, 139.39, 133.56, 133.53, 133.43, 133.16, 129.96, 129.93, 129.88, 129.85, 129.51, 129.06, 129.01, 128.63, 128.56, 128.48, 118.31, 80.28, 71.55, 71.00, 69.87, 67.36, 63.27, 61.00, 53.41, 52.45, 47.98, 34.71, 28.44, 28.33, 26.68, 18.28; HRMS (ESI) calcd. for $C_{54}H_{61}N_3O_{15}Na$ (M + Na)$^+$ m/z 1014.3998, found 1014.4005.

***N*-(*Tert*-butoxycarbonyl)-3-(1-(2,3,4,6-tetra-*O*-benzoyl-α-D-mannopyranosyl)eth-1-en-1-yl)-L-alanyl-L-alanyl-*O*-(tert-butyl)-L-threonine methyl ester (3at)**

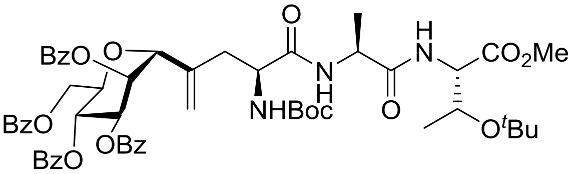

3at

General Procedure A. White foam, 54.0 mg, 52%. [α]$_D^{25}$ –52.2 (*c* 1.0, $CHCl_3$); ^{1}H NMR (500 MHz, $CDCl_3$) *δ* 8.11 (d, *J* = 7.8 Hz, 2H), 8.03 (d, *J* = 7.6 Hz, 2H), 7.98 (d, *J* = 8.0 Hz, 2H), 7.87 (d, *J* = 8.0 Hz, 2H), 7.58 (t, *J* = 7.2 Hz, 2H), 7.51 (t, *J* = 6.9 Hz, 2H), 7.47-7.35 (m, 7H), 7.29 (dd, *J* = 12.7, 5.7 Hz, 2H), 7.09 (d, *J* = 7.3 Hz, 1H), 6.55 (d, *J* = 9.4 Hz, 1H), 6.21 (s, 1H), 6.10 (t, *J* = 9.4 Hz, 1H), 5.73 (d, *J* = 9.5 Hz, 1H), 5.69 (s, 1H), 5.49 (s, 1H), 5.18 (d, *J* = 8.9 Hz, 1H), 4.82 (s, 1H), 4.73 (d, *J* = 12.2 Hz, 1H), 4.61–4.51 (m, 2H), 4.51–4.42 (m, 2H), 4.28 (ddt, *J* = 9.9, 5.0, 2.3 Hz, 1H), 4.21 (q, *J* = 6.0 Hz, 1H), 3.68 (s, 3H), 2.82 (d, *J* = 13.9 Hz, 1H), 2.60 (dd, *J* = 14.8, 8.9 Hz, 1H), 1.43 (d, *J* = 5.9 Hz, 3H), 1.40 (s, 9H), 1.15 (d, *J* = 4.8 Hz, 3H), 1.09 (s, 9H); ^{13}C NMR (125 MHz, $CDCl_3$) *δ* 172.38, 171.11, 171.09, 166.32, 166.07, 165.68, 165.49, 155.62, 139.36, 133.55, 133.49, 133.41, 133.18, 129.95, 129.92, 129.89, 129.85, 129.51, 129.07, 129.03, 128.60, 128.56, 128.54, 128.47, 118.70, 80.28, 74.23, 71.54, 71.06, 69.80, 67.40, 63.20, 57.89, 53.34, 52.28, 49.14, 36.56, 28.39, 28.31, 20.96, 18.59; HRMS (ESI) calcd. for $C_{56}H_{65}N_3O_{16}Na$ (M + Na)$^+$ m/z 1058.4257, found 1058.4260.

***N*-(*Tert*-butoxycarbonyl)-3-(1-(3,4,6-tri-*O*-benzoyl-2-deoxy-2-phthalimido-β-D-glucopyranosyl)eth-1-en-1-yl)-L-alanine methyl ester (3ba)**

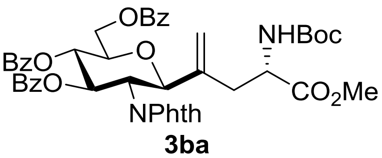

3ba

General Procedure B. White foam, 70.8 mg, 85%. [α]$_D^{25}$ 28.1 (*c* 1.0, $CHCl_3$); ^{1}H NMR (500 MHz, $CDCl_3$)

δ 8.07 (d, *J* = 7.3 Hz, 2H), 7.90 (d, *J* = 7.4 Hz, 2H), 7.86 (d, *J* = 7.6 Hz, 1H), 7.74 (d, *J* = 7.4 Hz, 2H), 7.72–7.67 (m, 2H), 7.67–7.62 (m, 1H), 7.55 (t, *J* = 7.3 Hz, 1H), 7.47 (t, *J* = 7.8 Hz, 1H), 7.41 (q, *J* = 8.1 Hz, 3H), 7.32 (t, *J* = 7.6 Hz, 2H), 7.27–7.22 (m, 2H), 6.40 (dd, *J* = 10.4, 9.3 Hz, 1H), 5.69 (t, *J* = 9.7 Hz, 1H), 5.52 (d, *J* = 8.3 Hz, 1H), **5.10 (s, 1H, vinyl H)**, **5.06 (d, *J* = 10.6 Hz, 1H, anomeric H)**, **4.94 (s, 1H, vinyl H)**, 4.69–4.61 (m, 2H), 4.49 (ddt, *J* = 12.4, 8.5, 4.4 Hz, 2H), 4.27 (ddd, *J* = 10.3, 5.0, 3.0 Hz, 1H), 3.65 (s, 1H), 2.73 (dd, *J* = 15.5, 3.9 Hz, 1H), 2.53 (dd, *J* = 15.5, 8.6 Hz, 1H), 1.30 (s, 9H); ^{13}C NMR (125 MHz, $CDCl_3$) *δ* 172.51, 167.99, 167.63, 166.24, 165.70, 165.31, 155.51, 139.29, 134.51, 134.24, 133.50, 133.36, 133.19, 131.63, 130.98, 129.95, 129.92, 129.89, 129.81, 129.72, 128.87, 128.68, 128.46, 128.37, 123.75, 119.43, 79.63, 79.49, 76.11, 71.80, 70.20, 63.18, 52.90, 52.57, 52.17, 32.68, 28.28; HRMS (ESI) calcd. for $C_{46}H_{44}N_2O_{13}Na$ $(M + Na)^+$ m/z 855.2736, found 855.741.

***N*-(*Tert*-butoxycarbonyl)-3-(1-(3,4,6-tri-*O*-benzoyl-2-deoxy-2-phthalimido-β-D-glucopyranosyl)eth-1-en-1-yl)-L-alanyl-L-leucine methyl ester** (**3bl**)

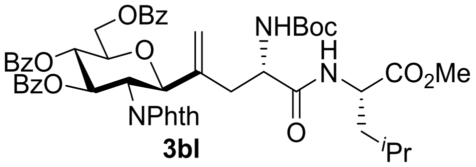

General Procedure B (at 15–17 °C). White foam, 69.8 mg, 73%. $[\alpha]_D^{25}$ 20.8 (*c* 1.1, $CHCl_3$); ^{1}H NMR (500 MHz, $CDCl_3$) *δ* 8.04 (d, *J* = 7.7 Hz, 2H), 7.93 (d, *J* = 8.2 Hz, 2H), 7.86 (d, *J* = 7.3 Hz, 1H), 7.75 (d, *J* = 8.2 Hz, 2H), 7.72–7.67 (m, 2H), 7.66–7.62 (m, 1H), 7.54 (t, *J* = 7.4 Hz, 1H), 7.49 (t, *J* = 7.6 Hz, 1H), 7.41 (t, *J* = 7.7 Hz, 3H), 7.34 (t, *J* = 7.7 Hz, 2H), 7.29–7.20 (m, 2H), 6.84 (d, *J* = 8.2 Hz, 1H), 6.41 (t, *J* = 9.8 Hz, 1H), 5.68 (t, *J* = 9.7 Hz, 1H), 5.52 (d, *J* = 7.7 Hz, 1H), 5.13 (d, *J* = 10.5 Hz, 1H), 5.09 (s, 1H), 4.99 (s, 1H), 4.71 (t, *J* = 10.4 Hz, 1H), 4.64 (dd, *J* = 12.2, 2.9 Hz, 1H), 4.58 (dd, *J* = 12.5, 5.9 Hz, 1H), 4.54 (dd, *J* = 8.2, 4.9 Hz, 1H), 4.46–4.31 (m, 1H), 3.70 (s, 3H), 2.66 (dd, *J* = 14.9, 5.8 Hz, 1H), 2.65–2.56 (m, 1H), 1.59–1.49 (m, 2H), 1.46–1.36 (m, 10H), 0.96–0.80 (m, 6H); ^{13}C NMR (125 MHz, $CDCl_3$) *δ* 172.93, 171.06, 168.04, 167.59, 166.32, 165.73, 165.37, 155.58, 139.05, 134.49, 134.25, 133.55, 133.37, 133.27, 131.60, 131.01, 129.96, 129.91, 129.81, 129.58, 128.84, 128.68, 128.52, 128.50, 128.39, 123.78, 123.73, 119.44, 79.98, 79.31, 76.47, 71.92, 70.37, 63.70, 53.39, 52.78, 52.28, 50.76, 41.67, 34.49, 28.34, 24.69, 22.76, 22.00; HRMS (ESI) calcd. for $C_{52}H_{55}N_3O_{14}Na$ $(M + Na)^+$ m/z 968.3576, found 968.3581.

***N*-(*Tert*-butoxycarbonyl)-3-(1-(3,4,6-tri-*O*-benzoyl-2-deoxy-2-phthalimido-β-D-glucopyranosyl)eth-1-en-1-yl)-L-alanyl-L-alanyl-*O*-(tert-butyl)-L-threonine methyl ester** (**3bt**)

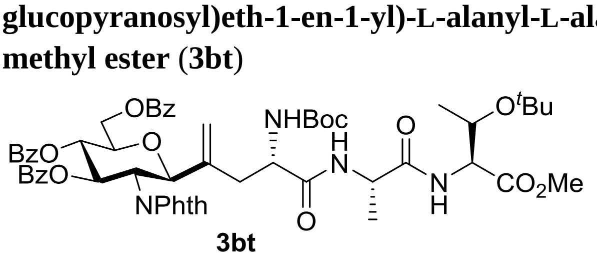

General Procedure B (at 15–17 °C). White foam, 60.6 mg, 57%. $[\alpha]_D^{25}$ 20.0 (*c* 1.0, $CHCl_3$); ^{1}H NMR (500 MHz, $CDCl_3$) *δ* 8.03 (d, *J* = 7.4 Hz, 2H), 7.92 (d, *J* = 7.3 Hz, 2H), 7.86 (d, *J* = 7.2 Hz, 1H), 7.74 (d, *J* = 7.2 Hz, 2H), 7.71-7.67 (m, 2H), 7.68–7.61 (m, 1H), 7.53 (t, *J* = 7.4 Hz, 1H), 7.48 (t, *J* = 7.4 Hz, 1H), 7.41 (t, *J* = 7.7 Hz, 3H), 7.34 (t, *J* = 7.8 Hz, 2H), 7.25 (t, *J* = 7.8 Hz, 2H), 7.13 (d, *J* = 7.2 Hz, 1H), 6.51 (d, *J* = 8.6 Hz, 1H), 6.41 (t, *J* = 9.8

Hz, 2H), 5.69 (t, *J* = 9.7 Hz, 1H), 5.61 (d, *J* = 7.4 Hz, 1H), 5.11 (d, *J* = 10.9 Hz, 1H), 5.10 (s, 1H), 5.00 (s, 1H), 4.70 (t, *J* = 10.4 Hz, 1H), 4.62 (d, *J* = 4.3 Hz, 2H), 4.52–4.43 (m, 2H), 4.42–4.34 (m, 2H), 4.22 (q, *J* = 5.8 Hz, 1H), 3.72 (s, 3H), 2.70–2.61 (m, *J* = 5.9 Hz, 2H), 1.34 (s, 9H), 1.32 (d, *J* = 7.4 Hz, 3H), 1.16 (d, *J* = 6.3 Hz, 3H), 1.11 (s, 9H); ^{13}C NMR (125 MHz, $CDCl_3$) δ 172.29, 171.15, 171.00, 168.01, 167.56, 166.32, 165.71, 165.33, 155.51, 139.03, 134.48, 134.24, 133.52, 133.35, 133.25, 131.61, 131.01, 129.96, 129.89, 129.81, 129.61, 128.87, 128.71, 128.53, 128.48, 128.38, 123.78, 123.73, 119.57, 79.96, 79.29, 76.44, 74.26, 71.90, 70.29, 67.41, 63.55, 57.91, 53.48, 52.76, 52.32, 49.11, 34.22, 28.41, 28.31, 20.95, 18.72; HRMS (ESI) calcd. for $C_{57}H_{64}N_4O_{16}Na$ $(M + Na)^+$ m/z 1083.4210, found 1083.4213.

***N*-(9-Fluorenylmethoxycarbonyl)-*O*-(*tert*-butyl)-L-threonyl-3-(1-(3,4,6-tri-O-benzoyl-2-deoxy-2-phthalimido-β-D-glucopyranosyl)eth-1-en-1-yl)-L-alanyl-L-isoleucine methyl ester** (**3bu**)

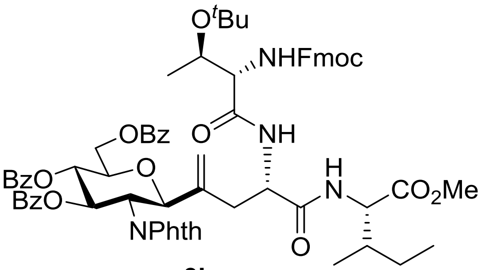

3bu

General Procedure B (at 25 °C). White foam, 95.5 mg, 78%. $[\alpha]_D^{25}$ 15.6 (*c* 1.0, $CHCl_3$); ^{1}H NMR (500 MHz, $CDCl_3$) δ 8.02 (d, *J* = 7.3 Hz, 2H), 7.92 (d, *J* = 7.3 Hz, 2H), 7.84 (d, *J* = 7.3 Hz, 1H), 7.76 (d, *J* = 7.6 Hz, 4H), 7.70 (d, *J* = 7.2 Hz, 1H), 7.63-7055 (m, 4H), 7.49 (dt, *J* = 14.6, 7.4 Hz, 2H), 7.41–7.38 (m, 5H), 7.34–7.28 (m, 4H), 7.25 (t, *J* = 7.8 Hz, 2H), 6.96 (d, *J* = 8.3 Hz, 1H), 6.45 (t, *J* = 9.8 Hz, 1H), 5.88 (d, *J* = 5.4 Hz, 1H), 5.69 (t, *J* = 9.7 Hz, 1H), 5.14 (d, *J* = 10.5 Hz, 1H), 5.06 (s, 1H), 4.95 (s, 1H), 4.83 (td, *J* = 8.5, 5.2 Hz, 1H), 4.69 (t, *J* = 10.4 Hz, 1H), 4.63–4.54 (m, 2H), 4.51 (dd, *J* = 8.4, 5.3 Hz, 1H), 4.43 (dd, *J* = 10.6, 7.3 Hz, 1H), 4.38–4.29 (m, 2H), 4.21 (t, *J* = 7.2 Hz, 1H), 4.18–4.09 (m, 2H), 3.71 (s, 3H), 2.84 (dd, *J* = 15.7, 5.2 Hz, 1H), 2.48 (dd, *J* = 15.6, 8.9 Hz, 1H), 1.90–1.78 (m, 1H), 1.51–1.33 (m, 1H), 1.29 (s, 3H), 1.24–1.14 (m, 1H), 1.02 (d, *J* = 6.2 Hz, 3H), 0.90-0.87 (m, 6H); ^{13}C NMR (125 MHz, $CDCl_3$) δ 171.72, 170.78, 169.10, 167.98, 167.71, 166.34, 165.71, 165.40, 155.86, 143.96, 143.78, 141.37, 141.36, 139.49, 134.44, 134.34, 133.50, 133.31, 133.11, 131.50, 130.90, 129.93, 129.89, 129.81, 129.67, 128.86, 128.73, 128.46, 128.42, 128.35, 127.80, 127.12, 125.24, 125.17, 123.86, 123.74, 120.07, 120.04, 117.51, 78.98, 76.37, 75.66, 71.93, 70.39, 66.96, 66.57, 63.70, 58.62, 56.83, 52.86, 52.04, 51.44, 47.23, 37.63, 35.27, 28.19, 25.34, 17.00, 15.51, 11.58; HRMS (ESI) calcd. for $C_{70}H_{72}N_4O_{16}Na$ $(M + Na)^+$ m/z 1247.4836, found 1247.4858

***O*-Benzyl-*N*-(*tert*-butoxycarbonyl)-L-threonyl-3-(1-(3,4,6-tri-*O*-benzoyl-2-deoxy-2-phthalimido-β-D-glucopyranosyl)eth-1-en-1-yl)-L-alanine methyl ester** (**3bp**)

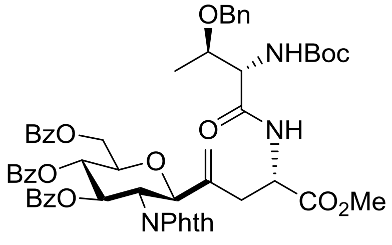

3bp

General Procedure B (at 25 °C). White foam, 64.5 mg, 63%. $[\alpha]_D^{25}$ 22.9 (*c* 1.2, $CHCl_3$); ^1H NMR (500 MHz, $CDCl_3$) δ 8.04 (d, *J* = 7.2 Hz, 2H), 7.90 (d, *J* = 7.2 Hz, 2H), 7.86 (d, *J* = 7.3 Hz, 1H), 7.75 (t, *J* = 8.0 Hz, 3H), 7.69 (t, *J* = 7.2 Hz, 1H), 7.64 (t, *J* = 7.3 Hz, 1H), 7.54 (t, *J* = 7.4 3Hz, 1H), 7.47 (t, *J* = 6.9 Hz, 1H), 7.41 (t, *J* = 7.8 Hz, 3H), 7.35–7.28 (m, 6H), 7.25 (t, *J* = 7.8 Hz, 3H), 6.97 (d, *J* = 8.0 Hz, 1H), 6.38 (dd, *J* = 10.3, 9.3 Hz, 1H), 5.67 (t, *J* = 9.7 Hz, 1H), 5.53 (d, *J* = 6.7 Hz, 1H), 4.98 (d, *J* = 10.5 Hz, H), 4.87 (s, 1H), 4.77–4.70 (m, 1H), 4.68 (s, 1H), 4.63 (dd, *J* = 12.3, 2.8 Hz, 1H), 4.60–4.51 (m, 3H), 4.44 (dd, *J* = 12.2, 4.5 Hz, 1H), 4.27 (dd, *J* = 6.9, 3.3 Hz, 1H), 4.16 (dd, *J* = 9.8, 4.4 Hz, 1H), 4.05 (dq, *J* = 9.3, 5.7 Hz, 1H), 3.60 (s, 3H), 2.78 (dd, *J* = 15.9, 3.9 Hz, 1H), 2.36 (dd, *J* = 15.8, 10.2 Hz, 1H), 1.43 (s, 9H), 1.18 (d, *J* = 6.4 Hz, 3H); ^{13}C NMR (125 MHz, $CDCl_3$) δ 172.07, 169.81, 168.03, 167.69, 166.23, 165.74, 165.32, 155.55, 138.97, 138.27, 134.50, 134.41, 133.47, 133.35, 133.14, 131.57, 130.94, 129.93, 129.84, 129.78, 128.98, 128.76, 128.47, 128.44, 128.38, 127.87, 127.80, 123.90, 123.77, 117.95, 79.79, 79.11, 75.97, 75.13, 71.84, 71.43, 70.16, 63.10, 56.89, 52.97, 52.34, 50.75, 32.96, 28.44, 14.82; HRMS (ESI) calcd. for $C_{57}H_{57}N_3O_{15}Na$ $(M + Na)^+$ m/z 1046.3862, found 1046.3867.

***N*-(*Tert*-butoxycarbonyl)-3-(1-(3,4,6-tri-*O*-acetyl-2-deoxy-2-phthalimido-β-D-galactopyranosyl)eth-1-en-1-yl)-L-alanine methyl ester** (**3ca**)

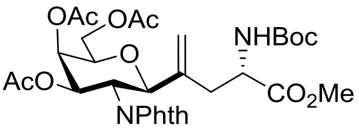

3ca

General Procedure B (at 25–30 °C; using PPh_3 (20 mol%) or (*R*)-Tol-BINAP (10 mol%) resulted in similar yield). White foam, 51.6 mg, 79%. $[\alpha]_D^{25}$ 0.5 (*c* 1.0, $CHCl_3$); ^1H NMR (400 MHz, $CDCl_3$) δ 7.87–7.80 (m, 2H), 7.76–7.74 (m, 2H), 5.96 (dd, *J* = 11.0, 3.3 Hz, 1H), 5.55 (d, *J* = 3.2 Hz, 1H), 5.44 (d, *J* = 8.3 Hz, 1H), 5.02 (s, 1H), 4.89 (s, 1H), 4.77 (d, *J* = 10.5 Hz, 1H), 4.59 (t, *J* = 10.7 Hz, 1H), 4.47 (td, *J* = 8.5, 3.7 Hz, 1H), 4.27–4.10 (m, 3H), 3.72 (s, 3H), 2.71 (dd, *J* = 15.9, 3.8 Hz, 1H), 2.50 (dd, *J* = 15.9, 8.8 Hz, 1H), 2.23 (s, 3H), 2.07 (s, 3H), 1.86 (s, 3H), 1.38 (s, 9H); ^{13}C NMR (125 MHz, $CDCl_3$) δ 172.75, 170.49, 170.31, 169.82, 167.99, 167.92, 155.61, 139.55, 134.58, 134.35, 131.55, 131.06, 123.72, 118.98, 80.02, 79.63, 74.30, 68.51, 67.15, 61.52, 52.25, 52.21, 48.88, 32.46, 28.37, 20.84, 20.78, 20.65; HRMS (ESI) calcd. for $C_{31}H_{38}N_2O_{13}Na$ $(M + Na)^+$ m/z 669.2266, found 669.2267.

***N*-(*Tert*-butoxycarbonyl)-3-(1-(2,3,4-tri-*O*-(4-trifluoromethyl)benzoyl-β/α-D-xylopyranosyl)eth-1-en-1-yl)-L-alanine methyl ester** (**3da**)

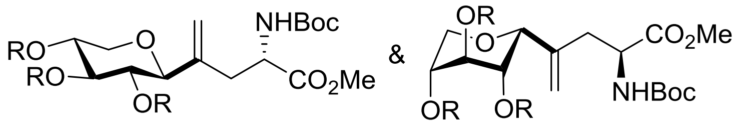

β anomer R = 4-CF_3-PhCO α anomer

3da

General Procedure B (at under 25–30 ℃, using NiBr$_2$(DME) (10 mol%) as catalyst). $^4C_1$(β)/$^1C_4$(α) = 3:1, 57 mg, 65%. **β anomer:** $[\alpha]_D^{25}$ -2.5 (*c* 0.9, $CHCl_3$); ^{1}H NMR (500 MHz, $CDCl_3$) δ 8.05 (t, *J* = 9.1 Hz, 4H), 8.00 (d, *J* = 8.2 Hz, 2H), 7.67 (t, *J* = 9.2 Hz, 4H), 7.60 (d, *J* = 8.2 Hz, 2H), 5.94 (t, *J* = 9.6 Hz, 1H), 5.47 (td, *J* = 9.7, 4.3 Hz, 2H), 5.36 (d, *J* = 8.0 Hz, 1H), 5.14 (s, 1H), 4.98 (s, 1H), 4.57–4.47 (m, 1H), 4.44 (dd, *J* = 11.3, 5.7 Hz, 1H), 4.13 (d, *J* = 9.7 Hz, 1H), 3.77 (s, 3H), 3.62 (t, *J* = 10.9 Hz, 1H), 2.77 (dd, *J* = 16.6, 3.7 Hz, 1H), 2.54 (dd, *J* = 16.5, 9.9 Hz, 1H), 1.42 (s, 9H); ^{13}C NMR (125 MHz, $CDCl_3$) δ 173.10, 164.81, 164.68, 164.34, 155.68, 138.95, 135.27 (d, *J* = 33.18 Hz), 135.22 (d, *J* = 33.70 Hz), 135.18 (d, *J* = 33.73 Hz), 132.08, 131.98, 130.33, 130.31, 130.24, 125.73 (q, *J* = 4.1 Hz), 123.55 (q, *J* = 272.87 Hz), 123.51 (q, *J* = 273.15 Hz), 123.48 (q, *J* = 272.83 Hz), 118.23, 83.64, 79.89, 74.20, 70.72, 70.43, 66.97, 52.49, 51.81, 32.39, 28.45; HRMS (ESI) calcd. for $C_{40}H_{36}F_9NO_{11}Na$ (M + Na)$^+$ m/z 900.2037, found 900.2041. **α anomer**: ^{1}H NMR (600 MHz, $CDCl_3$) δ 8.29 (d, *J* = 8.0 Hz, 2H), 8.10 (d, *J* = 8.1 Hz, H), 8.01 (d, *J* = 8.1 Hz, 2H), 7.80 (d, *J* = 8.2 Hz, 2H), 7.59 (d, *J* = 8.2 Hz, 2H), 7.47 (d, *J* = 8.1 Hz, 2H), 5.69 (td, *J* = 3.0, 1.5 Hz, 1H, H3), 5.41 (d, *J* = 1.9 Hz, 1H, H2), 5.40 (s, 1H, vinyl-H), 5.14 (dd, *J* = 3.1, 1.7 Hz, 1H, H4), 5.13 (d, *J* = 8.4 Hz, 1H, NH), 5.10 (s, 1H, vinyl-H), 4.56 (s, 1H, H1), 4.54–4.43 (m, 1H, α-H), 4.41 (d, *J* = 13.6 Hz, 1H, H5), 4.21 (dd, *J* = 13.6, 2.0 Hz, 1H, H5), 3.68 (s, 3H, OMe), 2.62 (dd, *J* = 14.8, 3.9 Hz, 1H, β-H), 2.50 (dd, *J* = 14.6, 10.1 Hz, 1H, β-H), 1.40 (s, 9H, Me); ^{13}C NMR (150 MHz, $CDCl_3$) δ 172.87, 164.50, 164.43, 163.33, 155.52, 139.39, 135.57 (q, *J* = 32.7 Hz), 135.28 (q, *J* = 33.15 Hz), 135.19 (q, *J* = 33.32 Hz), 130.63, 130.53, 130.38, 125.98 (q, *J* = 3.6 Hz), 125.57 (q, *J* = 3.6 Hz), 125.41 (q, *J* = 3.6 Hz), 123.58 (q, *J* = 272.76 Hz), 123.46 (q, *J* = 272.77 Hz), 123.44 (q, *J* = 272.72 Hz), 116.09, 80.17, 75.70 (C1), 67.66 (C2), 67.60 (C4), 67.12 (C3), 66.87 (C5), 52.53, 52.07, 35.81, 28.36.

***N*-(*Tert*-butoxycarbonyl)-3-(1-(3,4,6-tri-*O*-acetyl-2-deoxy-2-phthalimido-α-D-mannopyranosyl)eth-1-en-1-yl)-L-alanine methyl ester** (**3ea**)

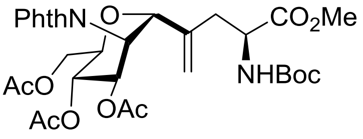

3ea

General Procedure B (at 25 ℃, using PPh$_3$ (20 mol%) as additive). White foam, 46.7 mg, 72%. $[\alpha]_D^{25}$ 12.6 (*c* 0.8, $CHCl_3$); ^{1}H NMR (500 MHz, $CDCl_3$) δ 7.80 (dd, *J* = 5.5, 3.1 Hz, 2H), 7.71 (dd, *J* = 5.5, 3.1 Hz, 2H), 5.43 (d, *J* = 10.5 Hz, 1H), 5.31 (t, *J* = 3.4 Hz, 1H), 5.28–5.21 (m, 2H), 5.06 (s, 1H), 4.97 (dd, *J* = 3.8, 1.7 Hz, 1H), 4.87–4.70 (m, 2H), 4.40 (dd, *J* = 11.7, 5.3 Hz, 1H), 4.38–4.29 (m, 1H), 4.24–4.15 (m, 1H), 3.67 (s, 3H), 2.56–2.45 (m, 2H), 2.23 (s, 3H), 2.13 (s, 3H), 2.07 (s, 3H), 1.40 (s, 9H); ^{13}C NMR (125 MHz, $CDCl_3$) δ 172.86, 170.73, 169.88, 169.75, 167.90, 155.46, 139.58, 134.39, 131.48, 123.57, 119.42, 79.84, 74.09, 69.36, 69.04, 68.46, 60.59, 53.30, 52.28, 49.00, 33.01, 28.40, 21.17, 21.00, 20.91; HRMS (ESI) calcd. for $C_{31}H_{38}N_2O_{13}Na$ (M + Na)$^+$ *m/z* 669.2266, found 669.2257.

***N*-(*Tert*-butoxycarbonyl)-3-(1-(2,3,4-tri-*O*-benzoyl-α-L-rhamnopyranosyl)eth-1-en-1-yl)-L-alanine methyl ester** (**3fa**)

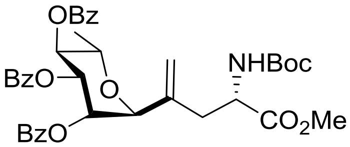

3fa

General Procedure B (at 25 °C, using PPh_3 (20 mol%) as additive). White foam, 67 mg, 93%. $[\alpha]_D^{25}$ 114.8 (*c* 1.0, $CHCl_3$); ^{1}H NMR (500 MHz, $CDCl_3$) δ 8.07 (d, J = 7.2 Hz, 2H), 7.98 (dd, J = 8.4, 1.2 Hz, 2H), 7.86 (d, J = 7.2 Hz, 2H), 7.59 (t, J = 1 Hz, 1H), 7.52 (t, J = 7.4 Hz, 1H), 7.48–7.43 (m, 3H), 7.39 (t, J = 7.8 Hz, 2H), 7.29 (t, J = 7.8 Hz, 2H), 6.16 (s, 1H, H2), 5.69–5.66 (m, 2H, H3, H4), 5.64 (s, 1H, vinyl-H), 5.43 (s, 1H, vinyl-H), 5.16 (d, J = 8.2 Hz, 1H, NH), 4.74 (s, 1H, H1), 4.58 (td, J = 8.9, 4.1 Hz, 1H, α-H), 3.87 (m, 1H, H5), 3.76 (s, 3H), 2.85 (dd, J = 14.7, 4.3 Hz, 1H), 2.43 (dd, J = 14.7, 9.7 Hz, 1H), 1.42 (m, 12H); ^{13}C NMR (125 MHz, $CDCl_3$) δ 172.85, 166.10, 165.77, 165.72, 155.40, 140.23, 133.46, 133.44, 133.34, 130.00, 129.80, 129.79, 129.66, 129.38, 129.18, 128.59, 128.54, 128.45, 118.48, 80.12, 75.92, 72.09, 70.98, 69.75, 69.71, 52.53, 52.08, 36.52, 28.35, 17.83; HRMS (ESI) calcd. for $C_{38}H_{41}NO_{11}Na$ $(M + Na)^+$ m/z 710.2572, found 710.2574.

***N*-(*Tert*-butoxycarbonyl)-3-(1-(6-*O*-*tert*-butyldiphenylsilyl-3,4-di-*O*-benzoyl-2-deoxy-2-phthalimido-β-D-glucopyranosyl)eth-1-en-1-yl)-L-alanine methyl ester** (**3ga**)

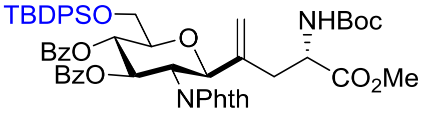

3ga

General Procedure B (at 30 °C). White foam, 67 mg, 93%. $[\alpha]_D^{25}$ 13.9 (*c* 1.0, $CHCl_3$); ^{1}H NMR (500 MHz, $CDCl_3$) δ 7.88 (dd, J = 13.0, 7.4 Hz, 3H), 7.79 (d, J = 7.4 Hz, 2H), 7.70 (q, J = 7.2, 6.5 Hz, 4H), 7.64 (t, J = 7.4 Hz, 1H), 7.56 (d, J = 7.1 Hz, 2H), 7.50 (t, J = 7.4 Hz, 1H), 7.43–7.32 (m, 6H), 7.26 (d, J = 9.6 Hz, 3H), 7.15 (t, J = 7.4 Hz, 2H), 6.37 (t, J = 9.8 Hz, 1H), 5.75 (t, J = 9.6 Hz, 1H), 5.08 (d, J = 7.2 Hz, 2H), 4.97 (t, J = 5.3 Hz, 2H), 4.65 (t, J = 10.4 Hz, 1H), 4.54 (td, J = 9.7, 9.2, 3.5 Hz, 1H), 3.96 (ddd, J = 10.0, 4.1, 2.3 Hz, 1H), 3.92–3.79 (m, 2H), 3.65 (s, 3H), 2.88–2.75 (m, 1H), 2.53 (dd, J = 16.9, 10.5 Hz, 1H), 1.34 (s, 9H), 1.04 (s, 9H); ^{13}C NMR (125 MHz, $CDCl_3$) δ 172.91, 168.03, 167.99, 165.83, 165.08, 155.57, 139.81, 135.67, 135.55, 134.45, 134.11, 133.24, 133.22, 133.01, 132.99, 131.72, 131.02, 129.87, 129.85, 129.74, 129.64, 129.37, 128.89, 128.41, 128.33, 127.76, 127.64, 123.71, 116.80, 79.73, 79.59, 78.81, 72.28, 69.77, 62.78, 53.04, 52.30, 51.43, 32.35, 28.34, 26.69, 19.24; HRMS (ESI) calcd. for $C_{55}H_{58}N_2O_{12}SiNa$ $(M + Na)^+$ m/z 989.3651, found 986.3658.

***N*-(*Tert*-butoxycarbonyl)-3-(1-(4-*O*-benzyl-3,6-di-*O*-benzoyl-2-deoxy-2-phthalimido-β-D-glucopyranosyl)eth-1-en-1-yl)-L-alanine methyl ester** (**3ha**)

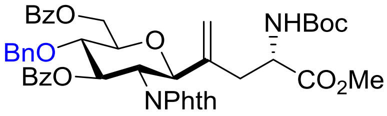

3ha

General Procedure B (at 30 °C). White foam, 47.7 mg, 58%. $[\alpha]_D^{25}$ 59.5 (*c* 0.5, $CHCl_3$); ^{1}H NMR (500 MHz, $CDCl_3$) δ 8.11 (d, J = 7.4 Hz, 2H), 7.86 (t, J = 8.3 Hz, 3H), 7.68 (dd, J = 13.8, 6.7 Hz, 2H), 7.64–7.59 (m, 2H), 7.50 (t, J = 7.8 Hz, 3H), 7.35 (t, J = 7.8 Hz, 2H), 7.10 (q, J = 5.9, 5.3 Hz, 5H),

6.29 (dd, J = 10.4, 8.6 Hz, 1H), 5.61 (d, J = 8.0 Hz, 1H), 5.06 (s, 1H), 4.97 (d, J = 10.5 Hz, 1H), 4.88 (s, 1H), 4.66 (s, 2H), 4.56 (q, J = 10.8 Hz, 2H), 4.49 (t, J = 10.5 Hz, 1H), 4.43 (td, J = 8.1, 3.8 Hz, 1H), 4.02 (dt, J = 10.2, 3.0 Hz, 1H), 3.93 (t, J = 9.4 Hz, 1H), 3.61 (s, 3H), 2.67 (dd, J = 15.3, 4.0 Hz, 1H), 2.52 (dd, J = 15.4, 8.2 Hz, 1H), 1.26 (s, 9H); ^{13}C NMR (125 MHz, $CDCl_3$) δ 172.51, 167.90, 166.35, 165.63, 155.57, 139.54, 136.94, 134.48, 134.16, 133.45, 133.34, 131.71, 131.06, 130.01, 129.99, 129.82, 129.26, 128.64, 128.57, 128.51, 128.33, 128.14, 123.80, 123.70, 119.44, 79.62, 79.22, 76.66, 75.00, 74.25, 63.25, 53.09, 52.81, 52.13, 32.43, 28.30; HRMS (ESI) calcd. for $C_{46}H_{46}N_2O_{12}Na$ $(M + Na)^+$ m/z 841.2943, found 841.2946.

***N*-(*Tert*-butoxycarbonyl)-3-(1-(2,3,4,6-*tetra*-*O*-benzoyl-β/α-D-glucopyranosyl)eth-1-en-1-yl)-L-alanine methyl ester** (**3ia**)

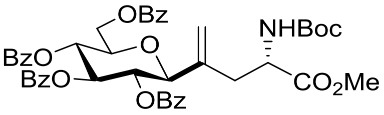

3ia, β

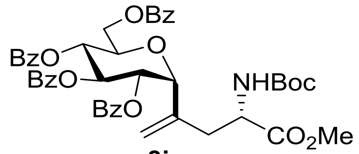

3ia, α

General Procedure B. **β-Anomer** (white foam, 20 mg, 25%): $[\alpha]_D^{25}$ 16.8 (c 0.5, $CHCl_3$); ^{1}H NMR (500 MHz, $CDCl_3$) δ 8.04 (dd, J = 7.8, 1.6 Hz, 2H), 7.93 (d, J = 7.7 Hz, 2H), 7.91 (d, J = 7.9 Hz, 2H), 7.83 (d, J = 7.3 Hz, 2H), 7.60–7.47 (m, 2H), 7.46–7.32 (m, 7H), 7.27 (t, J = 7.7 Hz, 2H), 5.97 (t, J = 9.6 Hz, 1H), 5.73–5.58 (m, 2H), 5.47 (t, J = 9.7 Hz, 1H), **5.12 (s, 1H, vinyl H)**, **4.93 (s, 1H, vinyl H)**, 4.65 (dd, J = 12.3, 3.0 Hz, 1H), 4.47 (ddt, J = 12.2, 8.0, 4.0 Hz, 2H), **4.23 (d, *J* = 9.8 Hz, 1H, anomeric H)**, 4.15 (dt, J = 8.7, 3.8 Hz, 1H), 3.69 (s, 2H), 2.76 (dd, J = 16.8, 3.4 Hz, 1H), 2.60 (dd, J = 16.6, 10.0 Hz, 1H), 1.37 (s, 9H); ^{13}C NMR (125 MHz, $CDCl_3$) δ 173.13, 166.30, 165.99, 165.96, 165.38, 155.83, 138.88, 133.65, 133.62, 133.42, 133.28, 129.98, 129.95, 129.83, 129.73, 129.03, 128.96, 128.87, 128.57, 128.54, 128.46, 118.21, 83.26, 79.66, 76.22, 73.84, 70.39, 69.63, 63.25, 52.32, 51.93, 31.83, 28.41. **α-Anomer** (white foam, 22 mg, 27%): $[\alpha]_D^{25}$ 6.7 (c 0.5, $CHCl_3$); ^{1}H NMR (500 MHz, $CDCl_3$) δ 8.05 (d, J = 6.8 Hz, 2H), 7.98 (d, J = 6.8 Hz, 2H), 7.96 (d, J = 8.3 Hz, 2H), 7.93 (d, J = 6.9 Hz, 2H), 7.58–7.52 (m, 2H), 7.50 (t, J = 7.5 Hz, 2H), 7.43 (t, J = 7.8 Hz, 2H), 7.37 (dt, J = 9.1, 7.7 Hz, 4H), 7.31 (t, J = 7.8 Hz, 2H), 6.11 (t, J = 7.9 Hz, 1H), **5.86 (s, 1H, vinyl H)**, 5.59 (t, J = 7.7 Hz, 1H), **5.38 (s, 1H, vinyl H)**, 5.13 (d, J = 8.1 Hz, 1H), **5.01 (d, *J* = 4.7 Hz, 1H, anomeric H)**, 4.65 (dd, J = 12.1, 6.6 Hz, 1H), 4.58 (dd, J = 12.1, 3.5 Hz, 1H), 4.46 (q, J = 7.1 Hz, 1H), 4.29 (td, J = 7.2, 3.5 Hz, 1H), 3.53 (s, 3H), 2.64 (dd, J = 14.8, 7.6 Hz, 1H), 2.56 (dd, J = 15.0, 6.4 Hz, 1H), 1.36 (s, 9H); ^{13}C NMR (125 MHz, $CDCl_3$) δ 172.98, 166.30, 165.76, 165.42, 165.41, 155.32, 138.10, 133.60, 133.59, 133.30, 130.02, 129.93, 129.88, 129.71, 129.12, 129.00, 128.64, 128.63, 128.58, 128.52, 119.36, 80.07, 72.54, 71.48, 70.52, 69.92, 69.33, 62.97, 52.36, 52.25, 36.40, 28.33; HRMS (ESI) calcd. for $C_{45}H_{45}NO_{13}Na$ $(M + Na)^+$ m/z 830.2783, found 830.2780.

***N*-(*Tert*-butoxycarbonyl)-3-(1-(4,6-di-*O*-benzoyl-2-deoxy-2-phthalimido-3-*O*-(2,3,4,6-tetra-*O*-acetyl-β-D-galactopyranosyl)-β-D-glucopyranosyl)eth-1-en-1-yl)-L-alanine methyl ester** (**3ja**)

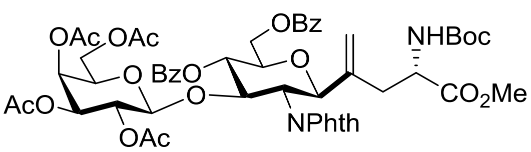

3ja

General Procedure B (at 25 ℃). White foam, 61.4 mg, 58%. $[\alpha]_D^{25}$ -23.1 (*c* 1.0, $CHCl_3$); ^1H NMR (500 MHz, $CDCl_3$) δ 8.06 (t, *J* = 7.1 Hz, 4H), 7.90 (d, *J* = 6.6 Hz, 1H), 7.91–7.78 (m, 3H), 7.57 (t, *J* = 7.4 Hz, 1H), 7.54 (t, *J* = 7.5 Hz, 1H), 7.46–7.40 (m, 4H), 5.51 (d, *J* = 8.4 Hz, 1H), 5.44 (t, *J* = 9.5 Hz, 1H), 5.04–4.97 (m, 2H), 4.95 (s, 1H), 4.88 (dd, *J* = 10.4, 7.8 Hz, 1H), 4.85 (s, 1H), 4.66 (td, *J* = 9.5, 8.9, 3.9 Hz, 2H), 4.56 (dd, *J* = 10.4, 3.5 Hz, 1H), 4.49–4.41 (m, 3H), 4.22 (d, *J* = 7.9 Hz, 1H), 4.18–4.13 (m, 1H), 3.64 (s, 3H), 3.56–3.42 (m, 2H), 3.33 (dd, *J* = 9.8, 6.8 Hz, 1H), 2.68 (dd, *J* = 15.7, 3.6 Hz, 1H), 2.47 (dd, *J* = 15.8, 8.9 Hz, 1H), 1.99 (s, 3H), 1.86 (s, 3H), 1.82 (s, 3H), 1.81 (s, 3H), 1.31 (s, 9H); ^{13}C NMR (101 MHz, $CDCl_3$) δ 172.51, 170.23, 170.15, 170.11, 169.08, 168.79, 167.69, 166.32, 164.75, 155.53, 139.74, 134.85, 133.41, 133.16, 131.26, 131.20, 129.97, 129.91, 129.88, 129.72, 128.43, 128.41, 123.86, 123.77, 118.85, 100.22, 79.94, 79.62, 76.05, 75.73, 70.94, 70.32, 69.12, 66.32, 63.43, 60.21, 53.92, 52.43, 52.16, 32.27, 28.29, 20.72, 20.52, 20.47, 20.41; HRMS (ESI) calcd. for $C_{53}H_{58}N_2O_{21}Na$ $(M + Na)^+$ *m/z* 1081.3424, found 1081.3427.

***N*-(*Tert*-butoxycarbonyl)-3-(1-(3,6-di-*O*-benzoyl-2-deoxy-2-phthalimido-4-*O*-(3,4,6-tri-*O*-acetyl-2-deoxy-2-phthalimido-β-D-glucopyranosyl)-β-D-glucopyranosyl)eth-1-en-1-yl)-L-alanine methyl ester** (**3ka**)

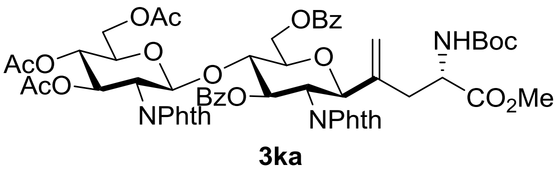

3ka

General Procedure B (at 25 ℃). White foam, 61.8 mg, 58% (66% yield based on recovered starting material). $[\alpha]_D^{25}$ 78.8 (*c* 1.0, $CHCl_3$); ^1H NMR (500 MHz, $CDCl_3$) δ 7.94 (d, *J* = 7.8 Hz, 2H), 7.89–7.79 (m, 3H), 7.70 (tt, *J* = 7.8, 3.6 Hz, 1H), 7.64–7.37 (m, 12H), 6.23 (dd, *J* = 10.2, 8.5 Hz, 1H), 5.70 (d, *J* = 8.4 Hz, 1H), 5.55 (dd, *J* = 10.7, 9.2 Hz, 1H), 5.23 (d, *J* = 8.3 Hz, 1H), 5.00 (t, *J* = 9.6 Hz, 1H), 4.99 (s, 1H), 4.92 (d, *J* = 10.5 Hz, 1H), 4.84 (s, 1H), 4.61 (d, *J* = 11.5 Hz, 1H), 4.39 (t, *J* = 10.4 Hz, 1H), 4.26 (dd, *J* = 10.7, 8.4 Hz, 1H), 4.26 (m, 1H), 4.20 (t, *J* = 9.2 Hz, 1H), 4.06 (dd, *J* = 12.1, 3.5 Hz, 1H), 3.93 (d, *J* = 9.9 Hz, 1H), 3.85 (dd, *J* = 12.3, 3.4 Hz, 1H), 3.51 (dd, *J* = 12.6, 2.2 Hz, 1H), 3.47 (s, 3H), 3.19 (dt, *J* = 10.0, 2.9 Hz, 1H), 2.53 (dd, *J* = 15.4, 4.0 Hz, 1H), 2.35 (dd, *J* = 15.4, 8.9 Hz, 1H), 2.00 (s, 3H), 1.87 (s, 3H), 1.76 (s, 3H), 1.24 (s, 9H); ^{13}C NMR (125 MHz, $CDCl_3$) δ 172.44, 170.66, 170.16, 169.26, 167.90, 167.76, 165.48, 165.01, 155.35, 139.12, 134.48, 134.28, 134.09, 133.73, 133.14, 131.73, 130.97, 129.83, 129.72, 129.56, 129.05, 128.85, 128.41, 123.74, 123.61, 118.93, 97.78, 79.56, 79.02, 76.40, 76.20, 73.12, 71.83, 70.53, 67.95, 62.59, 60.90, 55.06, 53.04, 52.26, 52.02, 32.44, 28.24, 20.82, 20.57, 20.40; HRMS (ESI) calcd. for $C_{59}H_{59}N_3O_{21}Na$ $(M + Na)^+$ *m/z* 1168.3533, found 1168.3532.

***N*-(*Tert*-butoxycarbonyl)-3-(1-(3,4-di-*O*-benzoyl-(2,3,4-tri-*O*-benzoyl-α-L-rhamnopyranosyl)-α-L-rhamnopyranosyl)eth-1-en-1-yl)-L-alanine methyl ester (3la)**

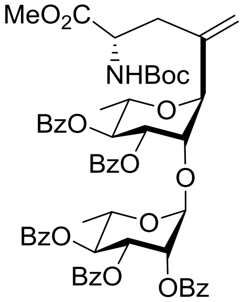

3la

The reaction conditions were modified basing on **General procdure A**: 0.1 mmol scale, alkyne (3.0 equiv.), $NiBr_2$(DME) (20 mol%) instead of $NiCl_2$(DME) as catalyst, dtbbpy (20 mol%) and PPh_3 (30 mol%) as additive, THF (1.5 mL), 25 ℃, 36 h. White foam, 70.8 mg, 68%. $[\alpha]_D^{25}$ 141.8 (*c* 1.0, $CHCl_3$); ^{1}H NMR (500 MHz, $CDCl_3$) δ 8.04–8.01 (m, 4H), 8.00–7.96 (m, 4H), 7.83 (dd, *J* = 8.3, 1.2 Hz, 2H), 7.55 (t, *J* = 7.5 Hz, 1H), 7.51 (q, *J* = 7.7 Hz, 2H), 7.45–7.35 (m, 8H), 7.32 (t, *J* = 7.6 Hz, 2H), 7.25 (t, *J* = 5.9 Hz, 2H), 5.98 (dd, *J* = 10.1, 3.4 Hz, 1H), 5.85 (dd, *J* = 3.6, 1.8 Hz, 1H), 5.72 (q, *J* = 8.8 Hz, 1H), 5.66 (t, *J* = 10.0 Hz, 2H), 5.48 (s, 1H), 5.41 (s, 1H), 5.21 (d, *J* = 8.3 Hz, 1H), 5.16 (d, *J* = 1.7 Hz, 1H), 4.89 (s, 1H), 4.75 (t, *J* = 2.7 Hz, 1H), 4.62 (td, *J* = 9.1, 3.7 Hz, 1H), 4.40 (dq, *J* = 12.7, 6.3 Hz, 1H), 3.85–3.69 (m, 5H), 2.88 (dd, *J* = 14.4, 3.9 Hz, 1H), 2.36 (dd, *J* = 14.1, 9.7 Hz, 1H), 1.45 (d, *J* = 6.3 Hz, 3H), 1.39 (d, *J* = 6.6 Hz, 3H), 1.38 (s, 9H); ^{13}C NMR (125 MHz, $CDCl_3$) δ 172.73, 166.44, 166.03, 165.44, 165.29, 165.09, 155.34, 141.04, 133.42, 133.37, 133.36, 133.24, 133.03, 130.06, 129.98, 129.90, 129.84, 129.81, 129.65, 129.52, 129.50, 129.38, 128.90, 128.62, 128.54, 128.49, 128.46, 128.30, 118.45, 99.36, 79.91, 76.46, 75.16, 72.23, 72.00, 71.89, 70.70, 69.89, 69.83, 67.79, 52.54, 52.16, 37.78, 28.31, 17.92, 17.70; HRMS (ESI) calcd. for $C_{58}H_{59}NO_{17}Na$ (M + Na)$^+$ m/z 1064.3675, found 1064.3679.

***N*-(*Tert*-butoxycarbonyl)-3-(1-(4-*O*-acetyl-3-*O*-(2,3,4-tri-*O*-acetyl-α-L-fucopyranosyl)-6-*O*-benzoyl-2-deoxy-2-phthalimido-β-D-glucopyranosyl)eth-1-en-1-yl)-L-alanine methyl ester** (**3ma**)

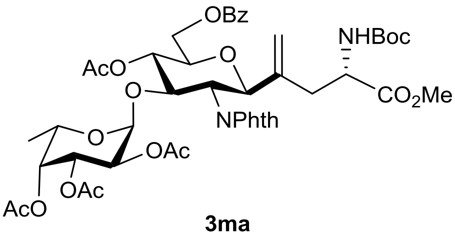

3ma

General Procedure B (at 30 ℃). White foam, 56.7 mg, 60%. $[\alpha]_D^{25}$ –15.6 (*c* 1.0, $CHCl_3$); ^{1}H NMR (500 MHz, $CDCl_3$) δ 8.09 (d, *J* = 7.8 Hz, 2H), 7.88 (d, *J* = 7.0 Hz, 1H), 7.77 (d, *J* = 7.1 Hz, 1H), 7.71 (p, *J* = 7.2 Hz, 2H), 7.56 (t, *J* = 7.4 Hz, 1H), 7.45 (t, *J* = 7.6 Hz, 2H), 5.53 (d, *J* = 8.3 Hz, 1H), 5.16 (d, *J* = 3.2 Hz, 1H), 5.13 (t, *J* = 9.4 Hz, 1H), 5.05 (dd, *J* = 11.2, 3.4 Hz, 1H), 5.04 (d, *J* = 3.4 Hz, 1H), 4.96 (s, 1H), 4.95 (dd, *J* = 10.6, 3.3 Hz, 1H), 4.84 (s, 1H), 4.72 (d, *J* = 10.6 Hz, 1H), 4.67 (t, *J* = 9.6 Hz, 1H), 4.54 (dd, *J* = 12.3, 2.8 Hz, 1H), 4.41 (q, *J* = 10.5, 9.0 Hz, 2H), 4.31 (dd, *J* = 12.2, 5.6 Hz, 1H), 4.21 (q, *J* = 6.4 Hz, 1H), 3.88–3.83 (m, 1H), 3.61 (s, 3H), 2.63 (dd, *J* = 15.5, 3.7 Hz, 1H), 2.46 (dd, *J* = 15.6, 8.6 Hz, 1H), 2.08 (s, 3H), 2.06 (s, 3H), 1.78 (s, 3H), 1.58 (s, 3H), 1.27 (s, 9H), 1.04 (d, *J* = 6.5 Hz, 3H); ^{13}C NMR (125 MHz, $CDCl_3$) δ 172.48, 170.49, 170.47, 170.01, 169.28, 168.40, 167.62, 166.36, 155.55, 139.61, 134.40, 134.17, 133.25, 131.63, 131.57, 129.99, 129.89, 129.82, 128.53, 124.45, 123.26, 119.15, 97.20, 79.59, 79.40, 76.25, 71.84, 71.36, 68.46, 67.10, 66.00, 63.23, 53.34, 52.62, 52.13, 32.41, 28.29, 21.19, 20.69, 20.54, 20.28, 15.42; HRMS (ESI) calcd. for $C_{48}H_{53}N_2O_{19}Na$ (M + Na)$^+$ m/z 961.3237, found 961.3225.

***N*-(*Tert*-butoxycarbonyl)-3-(*E*-(1-(2,3,4,6-tetra-*O*-benzoyl-α-D-mannopyranosyl)-2-phenyl)eth-1-en-1-yl)-L-alanine methyl ester** (**3av1**)

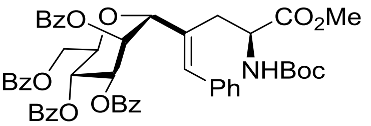

3av1

General Procedure B. White foam, 34.4 mg, 39%. $[\alpha]_D^{25}$ –55.3 (*c* 0.5, $CHCl_3$); ^1H NMR (500 MHz, $CDCl_3$ δ 8.09 (d, *J* = 4.2 Hz, 2H), 8.08 (d, *J* = 4.1 Hz, 2H), 7.99 (d, *J* = 6.8 Hz, 2H), 7.91 (d, *J* = 6.8 Hz, 2H), 7.59 (dt, *J* = 14.9, 7.5 Hz, 2H), 7.52 (t, *J* = 7.4 Hz, 1H), 7.48 (t, *J* = 7.4 Hz, 1H), 7.45–7.40 (m, 5H), 7.38 (t, *J* = 7.0 Hz, 5H), 7.35–7.28 (m, 3H), **7.21 (s, 1H, vinyl H)**, 6.34 (s, 1H), 6.10 (t, *J* = 9.1 Hz, 1H), 5.85 (dd, *J* = 9.4, 2.9 Hz, 1H), 5.13–5.04 (m, 2H), 4.71 (dd, *J* = 12.0, 3.0 Hz, 1H), 4.64 (dd, *J* = 12.1, 5.8 Hz, 1H), 4.59 (q, *J* = 7.7 Hz, 1H), 4.30–4.20 (m, 1H), 3.65 (s, 3H), 3.09 (dd, *J* = 14.3, 6.4 Hz, 1H), 2.79 (dd, *J* = 14.2, 8.0 Hz, 1H), 1.36 (s, 9H); ^{13}C NMR (125 MHz, $CDCl_3$) δ 172.84, 166.33, 166.12, 165.69, 165.56, 155.28, 135.99, 133.64, 133.55, 133.51, 133.23, 132.53, 130.03, 129.97, 129.90, 129.88, 129.63, 129.09, 129.01, 128.71, 128.68, 128.61, 128.57, 127.81, 80.06, 71.88, 70.95, 69.75, 67.65, 63.42, 52.48, 30.66, 28.35; HRMS (ESI) calcd. for $C_{51}H_{49}NO_{13}Na$ $(M + Na)^+$ m/z 906.3096, found 906.3093.

***N*-(*Tert*-butoxycarbonyl)-3-(*E*-(2-(2,3,4,6-tetra-*O*-benzoyl-α-D-mannopyranosyl)-2-phenyl)eth-1-en-1-yl)-L-alanine methyl ester** (**3av2**)

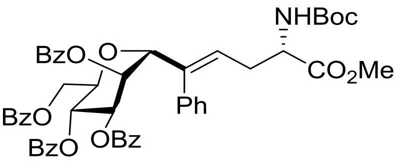

3av2

General Procedure B. Colorless oil, 8.6 mg, 13%. $[\alpha]_D^{25}$ –49.6 (*c* 0.3, $CHCl_3$); ^1H NMR (500 MHz, $CDCl_3$) δ 8.04 (d, *J* = 9.7 Hz, 2H), 7.97 (t, *J* = 7.7 Hz, 4H), 7.86 (d, *J* = 7.0 Hz, 2H), 7.61–7.54 (m, 2H), 7.54–7.49 (m, 1H), 7.48–7.35 (m, 8H), 7.31 (dt, *J* = 13.5, 7.6 Hz, 4H), 7.23 (t, *J* = 6.1 Hz, 2H), **6.20 (t, *J* = 7.4 Hz, 1H, vinyl H)**, 6.03 (t, *J* = 9.2 Hz, 1H), 5.95 (s, 1H), 5.80 (dd, *J* = 9.4, 3.0 Hz, 1H), 5.23 (d, *J* = 8.0 Hz, 1H), 5.13 (s, 1H), 4.60–4.40 (m, 3H), 4.40–4.35 (m, 1H), 3.75 (s, 3H), 2.67–2.55 (m, 1H), 2.49 (dt, *J* = 14.5, 7.3 Hz, 1H), 1.42 (s, 9H); ^{13}C NMR (125 MHz, $CDCl_3$) δ 166.38, 165.66, 165.61, 136.56, 133.58, 133.50, 133.38, 133.15, 130.01, 129.96, 129.90, 129.69, 129.22, 129.18, 128.80, 128.68, 128.62, 128.59, 128.49, 128.46, 127.97, 126.57, 79.92, 78.05, 70.54, 69.86, 67.88, 63.51, 53.45, 52.61, 32.80, 28.45; HRMS (ESI) calcd. for $C_{51}H_{49}NO_{13}Na$ $(M + Na)^+$ m/z 906.3096, found 906.3093.

***N*-(*Tert*-butoxycarbonyl)-3-(*E*-(1-(3,4,6-tri-*O*-benzoyl-2-deoxy-2-phthalimido-β-D-glucopyranosyl)-2-phenyl)eth-1-en-1-yl)-L-alanine methyl ester** (**3bv1**)

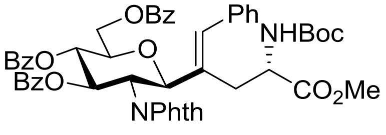

3bv1

General Procedure B. White foam, 38 mg, 42%. $[\alpha]_D^{25}$ –25.0 (*c* 0.5, $CHCl_3$); ^1H NMR (500 MHz, $CDCl_3$) δ 8.11 (d, *J* = 7.5 Hz, 2H), 7.92 (d, *J* = 6.9 Hz, 2H), 7.83 (d, *J* = 7.3 Hz, 1H), 7.77 (d, *J* = 7.8 Hz, 2H), 7.69 (d, *J* = 7.2 Hz, 1H), 7.64 (t, *J* = 7.2 Hz, 1H), 7.60 (d, *J* = 7.3 Hz, 1H), 7.54 (t, *J* = 7.7 Hz, 1H), 7.48 (t, *J* = 7.4 Hz, 1H), 7.46–7.39 (m, 3H), 7.33 (t, *J* = 7.8 Hz, 2H), 7.26 (t, *J* = 7.8 Hz, 2H), 7.19 (t, *J* = 7.2 Hz, 2H), 7.14 (t, *J* = 7.4 Hz, 1H), 6.96 (d, *J* = 7.4 Hz, 2H), **6.65 (s, 1H, vinyl H)**, 6.48 (t, *J* = 9.8 Hz, 1H), 5.77 (t, *J* = 9.7 Hz, 1H), 5.16 (d, *J* = 10.5 Hz, 1H), 5.13 (d, *J* = 8.8 Hz, 1H), 4.82 (t, *J* = 10.4 Hz, 1H), 4.69 (d, *J* = 12.1 Hz, 1H), 4.58 (dd, *J* = 12.1, 4.7 Hz, 2H), 4.32 (dt, *J* = 8.1, 3.6 Hz, 1H), 3.52 (s, 3H), 2.89 (dd, *J* = 14.3, 8.9 Hz, 1H), 2.78 (dd, *J* = 14.3, 4.2

Hz, 1H), 1.34 (s, 9H); ^{13}C NMR (125 MHz, $CDCl_3$) δ 172.77, 168.26, 167.64, 166.39, 165.81, 165.34, 155.15, 135.89, 134.51, 134.29, 133.48, 133.36, 133.11, 132.30, 131.62, 131.03, 130.08, 129.98, 129.89, 129.81, 129.05, 128.83, 128.64, 128.53, 128.48, 128.46, 128.39, 128.35, 128.30, 127.49, 123.80, 123.65, 80.68, 79.52, 76.19, 72.07, 70.21, 63.34, 53.38, 53.01, 52.21, 29.56, 28.40; HRMS (ESI) calcd. for $C_{52}H_{48}N_2O_{13}Na$ $(M + Na)^+$ m/z 931.3049, found 931.3044.

***N*-(*Tert*-butoxycarbonyl)-3-(*E*-(2-(3,4,6-tri-*O*-benzoyl-2-deoxy-2-phthalimido-β-D-glucopyranosyl)-2-phenyl)eth-1-en-1-yl)-L-alanine methyl ester** (**3bv2**)

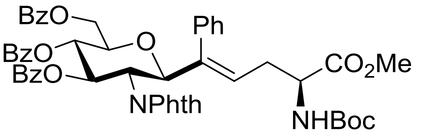

3bv2

General Procedure B. Colorless oil, 4.7 mg, 5%. $[\alpha]_D^{25}$ 57.2 (*c* 0.2, $CHCl_3$); ^{1}H NMR (500 MHz, $CDCl_3$ δ 8.06 (d, *J* = 8.1 Hz, 2H), 7.89 (dd, *J* = 8.4, 1.3 Hz, 2H), 7.75 (d, *J* = 7.3 Hz, 1H), 7.71–7.53 (m, 6H), 7.50–7.41 (m, 3H), 7.38 (t, *J* = 7.4 Hz, 1H), 7.32 (dd, *J* = 8.3, 7.4 Hz, 2H), 7.21 (dd, *J* = 8.3, 7.4 Hz, 2H), 7.10–7.04 (m, 3H), 6.97 (dd, *J* = 7.4, 2.0 Hz, 2H), 6.24 (dd, *J* = 10.4, 9.3 Hz, 1H), **5.84 (t, *J* = 7.4 Hz, 1H, vinyl H)**, 5.62 (t, *J* = 9.7 Hz, 1H), 5.36 (d, *J* = 10.3 Hz, 1H), 4.96 (d, *J* = 8.2 Hz, 1H), 4.72 (dd, *J* = 12.1, 3.0 Hz, 1H), 4.46 (dd, *J* = 11.7, 3.9 Hz, 1H), 4.43 (t, *J* = 9.7 Hz, 1H), 4.27 (ddd, *J* = 10.1, 4.3, 3.0 Hz, 1H), 4.21 (q, *J* = 6.3 Hz, 1H), 3.53 (s, 3H), 2.50–2.40 (m, 1H), 2.40–2.20 (m, 1H), 1.40 (s, 9H); ^{13}C NMR (125 MHz, $CDCl_3$) δ 168.18, 166.32, 165.95, 165.35, 135.71, 134.18, 134.02, 133.46, 133.27, 133.20, 130.00, 129.98, 129.94, 129.87, 129.40, 129.11, 128.81, 128.55, 128.49, 128.34, 128.13, 127.51, 123.53, 123.46, 79.42, 78.94, 75.93, 72.50, 70.16, 63.11, 54.26, 52.83, 52.19, 31.72, 28.45; HRMS (ESI) calcd. for $C_{52}H_{48}N_2O_{13}Na$ $(M + Na)^+$ m/z 931.3049, found 931.3046.

***N*-(*Tert*-butoxycarbonyl)-3-(1-(2,4-di-*O*-benzoyl-3-*O*-(4-*O*-benzoyl-3-*O*-(3,4-di-*O*-benzoyl-2-*O*-(2,3,4-tri-*O*-benzoyl-α-L-rhamnopyranosyl)-α-L-rhamnopyranosyl)-2-*O*-(3,4,6-tri-*O*-acetyl-2-deoxy-2-phthalimido-β-D-glucopyranosyl)-α-L-rhamnopyranosyl)-α-L-rhamnopyranosyl)eth-1-en-1-yl)-L-alanine methyl ester** (**14**)

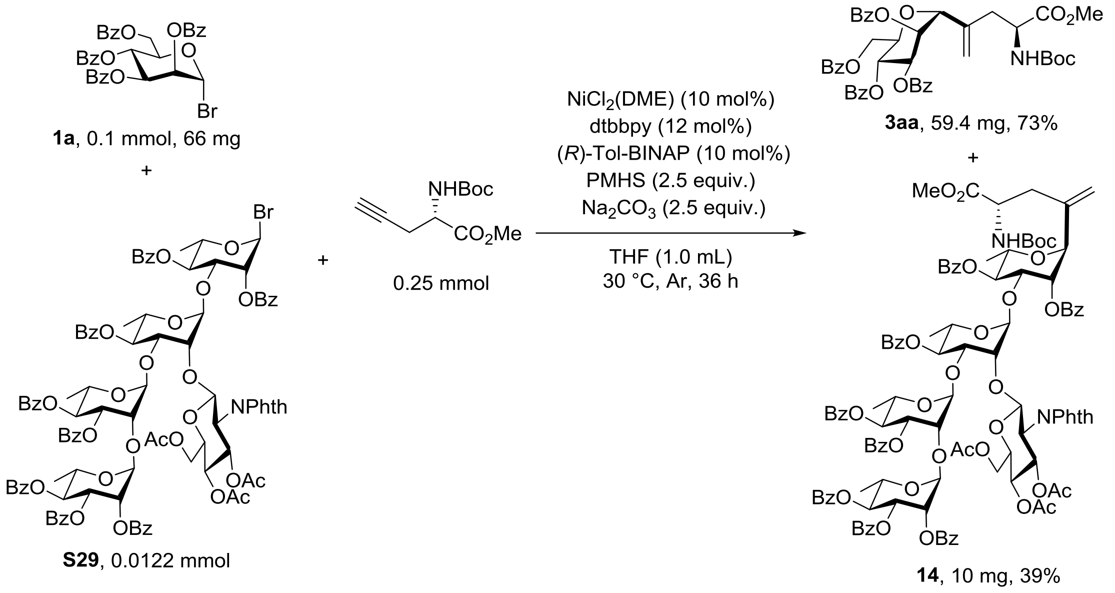

Supplementary Figure 36. Preparation of pentasaccharide amino acid **14**.

The amount of **S29** was small (about 0.0122 mmol, Supplementary Figure 11), therefore accurately weighing other reagents was difficult. As a compromise, we conducted the synthesis with **1a** as the reference substrate. Thus, to an oven-dried 10 mL Schlenk tube (Titan, TF891910) containing a Teflon coated magnetic stirring bar was added glycosyl bromide **1a** (66 mg, 0.1 mmol), $NiCl_2$(DME) (10 mol%), dtbbpy (12 mol%), (*R*)-Tol-BINAP (10 mol%), and Na_2CO_3 (2.5 equiv). The tube was sealed with a rubber cap and wrapped with parafilm, and was then evacuated then refilled with Ar for at least five cycles. Alkyne **2a** (56 mg, 0.25 mmol) was dissolved in THF (1.0 mL) and injected into the reaction tube. When stirring, silane PMHS (32 μL, 2.5 equiv.) was injected via microliter syringe. The tube was kept stirring under 30 ℃ for 1.5 h. Then **S29** (which was used directly after preparation, about 0.0122 mmol, see Supplementary Figure 11) was dissolved in THF (0.3 mL) and was injected into the reaction tube via microliter syringe; the residue glycosyl bromide was dissolved in another portion of THF (0.2 mL) and added. The tube was evacuated then refilled with Ar for three cycles again, then the mixture was stirred at 30 ℃ for 36 h. The reaction mixture was diluted with CH_2Cl_2 (20 mL) and filtered. The filtrate was concentrated *in vacuo*. The residue was purified by preparative TLC (petroleum ether/ dichloromethane/ethyl acetate = 2:1:1, R_f = 0.3) to afford the desired product **3aa** (59.4 mg, 73%) and **14** (10 mg, 39% over two steps).

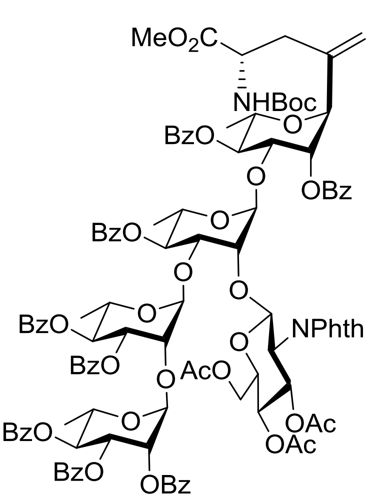

14

$[\alpha]_D^{25}$ 83.9 (*c* 0.3, $CHCl_3$); ^1H NMR (600 MHz, $CDCl_3$) δ 8.13 (d, *J* = 7.7 Hz, 2H), 8.06 (d, *J* = 7.9 Hz, 2H), 8.02 (d, *J* = 7.8 Hz, 2H), 8.00 (d, *J* = 7.6 Hz, 2H), 7.96 (t, *J* = 8.2 Hz, 4H), 7.85 (s, 2H), 7.80 (d, *J* = 7.7 Hz, 2H), 7.67–7.54 (m, 5H), 7.53–7.45 (m, 7H), 7.43–7.35(m, 5H), 7.30–7.23 (m, 5H), 7.06 (t, *J* = 7.7 Hz, 1H), 6.85 (t, *J* = 7.5 Hz, 1H), 6.01 (dd, *J* = 10.7, 9.0 Hz, 1H), 5.83 (dd, *J* = 10.1, 3.0 Hz, 2H), 5.80–5.72 (m, 3H), 5.50–5.45 (m, 2H), 5.43 (t, *J* = 10.0 Hz, 1H), 5.28 (s, 1H), 5.27 (s, 1H), 5.15 (d, *J* = 6.7 Hz, 1H), 5.08–5.01 (m, 2H), 4.89 (s, 1H), 4.87 (s, 1H), 4.73 (t, *J* = 9.8 Hz, 1H), 4.65 (s, 1H), 4.51 (m, 1H), 4.36 (d, *J* = 8.4 Hz, 1H), 4.32–4.26 (m, 2H), 4.17 (s, 1H), 4.09 (d, *J* = 9.0 Hz, 1H), 4.02 (s, 1H), 3.94–3.88 (m, 2H), 3.83–3.73 (m, 1H), 3.79–3.74 (m, 1H), 3.72 (s, 1H), 3.62 (dq, *J* = 11.8, 6.1 Hz, 1H), 2.75 (d, *J* = 15.3 Hz, 1H), 2.39 (dd, *J* = 15.1, 9.6 Hz, 1H), 2.04 (s, 3H), 1.99 (s, 3H), 1.84 (s, 3H), 1.38 (s, 9H), 1.34 (d, *J* = 6.4 Hz, 3H), 1.28 (d, *J* = 6.1 Hz, 3H), 0.84 (d, *J* = 6.2 Hz, 3H), 0.71 (d, *J* = 6.2 Hz, 3H); ^{13}C NMR (150 MHz, $CDCl_3$) δ 172.86, 170.78, 169.97, 169.86, 167.78, 166.15, 165.91, 165.71, 165.59, 165.38, 165.15, 165.08, 164.29, 155.43, 140.74, 134.15, 133.67, 133.58, 133.55, 133.21, 133.17, 133.10, 133.01, 131.57, 130.10, 130.01, 130.00, 129.99, 129.93, 129.82, 129.71, 129.63, 129.57, 129.52, 129.45, 129.44, 129.08, 128.80, 128.72, 128.54, 128.48, 128.42, 128.39, 124.05, 117.60, 100.51, 99.17, 99.08, 98.23, 80.05, 76.52, 75.40, 75.11, 73.86, 73.18, 72.70, 71.92, 71.57, 71.18, 70.54, 70.36, 70.31, 69.98, 69.54, 69.37, 68.37, 68.16, 67.47, 61.82, 54.69, 53.93, 53.58, 52.53, 52.23, 35.92, 28.38, 20.90, 20.85, 20.71, 17.67, 17.58, 17.50, 17.41; HRMS (ESI) calcd. for $C_{111}H_{110}N_2O_{37}Na$ $(M + Na)^+$ m/z 2085.6680, found 2085.6686.

***N*-(*Tert*-butoxycarbonyl)-3-(1-(3-*O*-(2,3,4-tri-*O*-acetyl-α-L-fucopyranosyl)-4-*O*-(2,3,4,6-tetra-*O*-acetyl-β-D-galactopyranosyl)-6-*O*-benzoyl-2-deoxy-2-phthalimido-β-D-glucopyranosyl)eth-1-en-1-yl)-L-alanine methyl ester (15)**

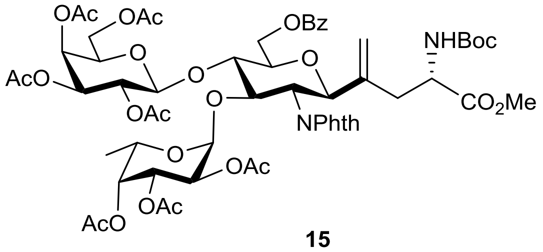

15

General Procedure B (at 30 °C). White foam, 71 mg, 58%. $[\alpha]_D^{25}$ –48.8 (*c* 1.0, $CHCl_3$); ^1H NMR (600 MHz, $CDCl_3$) δ 8.11 (d, *J* = 7.8 Hz, 2H), 7.84 (dd, *J* = 5.7, 2.8 Hz, 1H), 7.79 (dd, *J* = 5.4, 3.3 Hz, 1H), 7.78–7.75 (m, 2H), 7.64 (t, *J* = 7.2 Hz, 1H), 7.55 (t, *J* = 7.5 Hz, 2H), 5.41–5.32 (m, 3H, NH, H3’, H4’, H4’’), 5.15 (t, *J* = 7.81 Hz, 1H, H2’’), 5.14 (dd, *J* = 9.7, 4.3 Hz, 1H, H2’), 5.01 (q, *J* = 6.7 Hz, 1H, H5’), 4.95 (d, *J* = 4.0 Hz, 1H, H1’), 4.94 (dd, *J* = 12.18, 2.9 Hz, 1H), 4.88 (t, *J* = 9.46 Hz, 1H, H3), **4.85 (s, 1H, vinyl-H)**, 4.78 (dd, *J* = 10.9, 4.1 Hz, 1H, H3’’), **4.76 (s, 1H, vinyl-H)**, 4.69 (d, *J* = 8.2 Hz, 1H, H1’’), 4.60 (d, *J* = 10.5 Hz, 1H, H1), 4.55 (dd, *J* = 11.4, 5.9 Hz, 1H, H6’), 4.44 (dd, *J* = 12.2, 3.9 Hz, 1H, H6), 4.38 (dt, *J* = 8.0, 3.9 Hz, 1H, α-H), 4.34 (t, *J* = 10.4 Hz, 1H, H2), 4.30 (dd, *J* = 11.4, 8.2 Hz, 1H, H6’’), 4.07 (t, *J* = 9.4 Hz, 1H, H4), 3.84–3.78 (m, 1H, H5), 3.76–3.73

(m, 1H, H5’’), 3.56 (s, 3H, OMe), 2.61 (dd, *J* = 16.0, 3.6 Hz, 1H, β-H), 2.40 (dd, *J* = 15.9, 9.3 Hz, 1H, β-H), 2.18 (s, 3H), 2.15 (s, 3H), 2.07 (s, 3H), 2.07 (s, 3H), 2.01 (s, 3H), 1.97 (s, 3H), 1.92 (s, 3H), 1.30 (s, 9H), 1.24 (d, *J* = 6.6 Hz, 3H); ^{13}C NMR (150 MHz, $CDCl_3$) δ carbonyl (172.50, 170.69, 170.54, 170.42, 170.36, 169.98, 169.70, 169.09, 168.63, 168.07, 165.81, 155.39), arene and alkene (139.57, 134.51, 134.45, 133.58, 131.47, 131.07, 129.74, 128.79, 123.79, 123.53, 118.43), 100.63 (C1’’), 95.08 (C1’), 80.01 (C1), 79.49 (C-Boc), 77.28 (C5), 75.38 (C4), 71.96 (C3), 71.38 (C4’ or C4’’), 71.14 (C5’’), 71.08 (C4’ or C4’’), 68.88 (C2’ or C2’’), 68.01 (C3’’), 67.95 (C2’ or C2’’), 66.59 (C3’), 64.30 (C5’), 62.16 (C6), 60.44 (C6’’), 54.51(C2), 52.16 (α-C), 52.00 (OMe), 31.80 (β-C), 28.20 (Me-Boc), Me of acetyl (20.82, 20.71, 20.70, 20.65, 20.55, 20.53), 15.88 (C6’); HRMS (ESI) calcd. for $C_{58}H_{70}N_2O_{27}Na$ $(M + Na)^+$ m/z 1249.4058, found 1249.4046.

2.6 The Scale-up Reaction

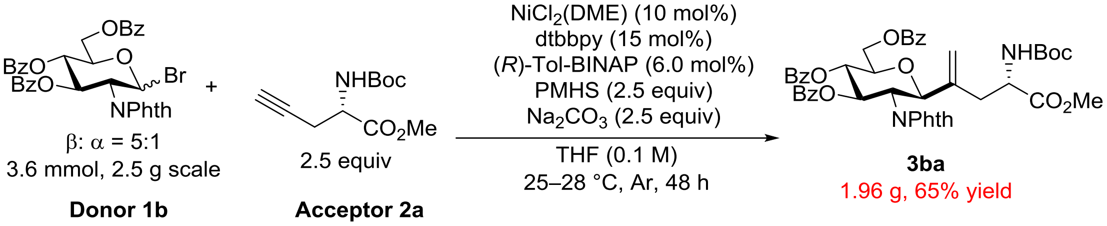

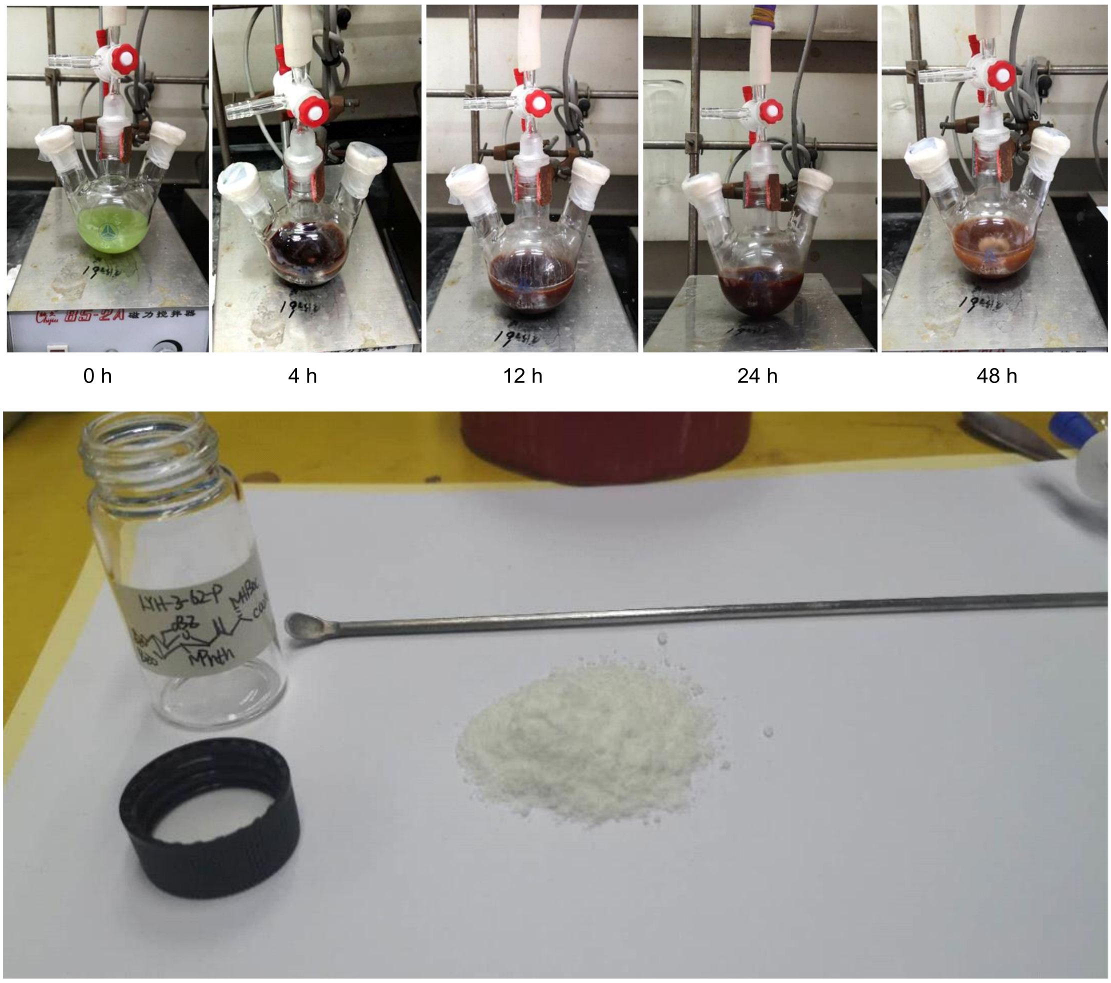

Supplementary Figure 37. A gram scale preparation of **3ba**.

To a 250 mL three neck round-bottom flask containing a Teflon coated magnetic stirring bar was added glycosyl bromide **1b** (2.465 g, 3.6 mmol), $NiCl_2$(DME) (79 mg, 10 mol%), dtbbpy (145 mg, 15 mol%), (*R*)-Tol-BINAP (146 mg, 6 mol%), and Na_2CO_3 (954 mg, 2.5 equiv). The flask was equipped with a balloon filled with Ar and sealed with two rubber caps then with parafilm and evacuated then refilled with Ar for at least five cycles. Compound **2a** (2.04 g, 2.5 equiv.) was dissolved in THF (36 mL) and injected into the flask. The flask was evacuated and refilled with Ar for another three cycles. When stirring, silane PMHS (1.116 mL, 2.5 equiv.) was injected via microliter syringe. After which a light green solution was obtained and 10 minutes later the reaction mixture began to turn red slowly. 30 minutes later the reaction mixture became

dark red. The mixture was kept stirring vigorously under indicated temperature (25–28 ℃) for 48 h until the completion of the reaction as determined by TLC. The mixture was then diluted with CH_2Cl_2 (50 mL) and filtered. The filtrate was concentrated *in vacuo*. The residue was purified by column chromatography on silica gel (petroleum ether/ethyl acetate = 2:1 or petroleum ether/dichloromethane/ethyl acetate = 2:1:0.5) to afford the desired product **3ba** (1.96 g, 65%) as a white solid.

2.7 Transformations of vinyl *C*-glycosyl amino acids

Protecting group transformation

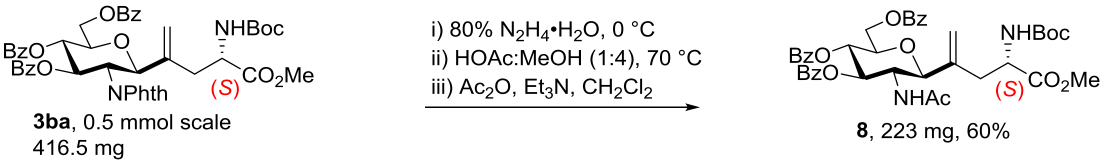

To a stirred solution of **3ba** (416.5 mg, 0.5 mmol) in MeOH (5 mL) was added 80% hydrazine hydrate (160 μL, about 5.0 equiv.) via microliter syringe at 0 ℃. The mixture was stirred at 0 ℃ for 9 h to open the Phth ring. After the disappearance of **3ba** as determined by TLC (petroleum ether/ethyl acetate = 1:1), the mixture was diluted and azeotroped with toluene (3×30 mL) to remove the residue hydrazine. The residue was dissolved in MeOH/HOAc (10 mL, 4:1, v/v), and the solution was heated under 70 ℃ for 1.5 h to remove the residue Phth. The mixture was concentrated *in vacuo* and dried over oil pump. The residue was dissolved in dry CH_2Cl_2 (7 mL), to which Et_3N (0.8 mL) and Ac_2O (0.5 mL) were added. The mixture was stirred at room temperature for 6 h, after which MeOH (0.5 mL) was added and stirred for 0.5 h. The solvent was removed *in vacuo*. The residue was purified by column chromatography (petroleum ether/ethyl acetate = 1:1) to provide **8** (223 mg, 60% yield).

***N*-(*Tert*-butoxycarbonyl)-3-(1-(3,4,6-tri-*O*-benzoyl-2-deoxy-2-acetylamido-β-D-glucopyranosyl)eth-1-en-1-yl)-L-alanine methyl ester (8)**

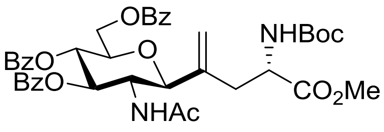

8

^{1}H NMR (500 MHz, $CDCl_3$) δ 8.02 (d, J = 7.7 Hz, 2H), 7.91 (d, J = 7.7 Hz, 2H), 7.87 (d, J = 8.0 Hz, 2H), 7.53–7.45 (m, 3H), 7.40–7.29 (m, 6H), 6.11 (d, J = 10.8 Hz, 1H), 5.85 (d, J = 7.4 Hz, 1H), 5.66 (t, J = 9.7 Hz, 1H), 5.62–5.49 (m, 1H), 5.10 (s, 1H), 5.01 (s, 1), 4.63 (dd, J = 12.3, 2.8 Hz, 1), 4.51–4.42 (m, 3H), 4.17–4.07 (m, 1H), 4.00 (d, J = 9.7 Hz, 1H), 3.68 (s, 3H), 2.74 (d, J = 15.5 Hz, 1H), 2.60 (dd, J = 16.1, 10.7 Hz, 1H), 1.81 (s, 3H), 1.39 (s, 9H); ^{13}C NMR (125 MHz, $CDCl_3$) δ 173.14, 170.95, 167.42, 166.31, 165.24, 156.03, 139.70, 133.83, 133.57, 133.20, 129.94, 129.86, 129.74, 129.66, 128.88, 128.64, 128.56, 128.46, 117.23, 84.64, 79.45, 76.06, 74.64, 69.49, 63.32, 52.25, 51.93, 51.78, 32.05, 28.41, 23.14; HRMS (ESI) calcd. for $C_{40}H_{44}N_2O_{12}Na$ $(M + Na)^+$ m/z 767.2786, found 767.2792.

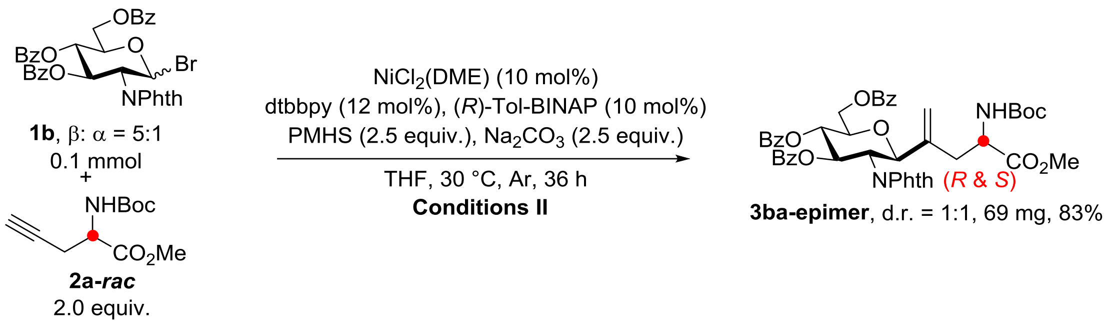

The **3ba-epimer** was prepared according to **General Procedure B** and was used for analysing the dr value of **3ba**. To an oven-dried 10 mL Schlenk tube (Titan, TF891910) containing a Teflon coated magnetic stirring bar were added glycosyl bromide **1b** (0.1 mmol), $NiCl_2$(DME) (10 mol%), dtbbpy (12 mol%), (*R*)-Tol-BINAP (10 mol%), and Na_2CO_3 (2.5 equiv). The tube was sealed with a rubber cap then with parafilm and evacuated then refilled with Ar for at least five cycles. *N*-Boc-Pra-OMe (**2a-*rac***, prepared by mixing the D- and L-enantiomer in 1:1 ratio, 2.0 equiv) was dissolved in THF (1.0 mL) and injected into the reaction tube. When stirring, PMHS (32 μL, 2.5 equiv.) was injected via microliter syringe. The tube was kept stirring under 30 ℃ for 36 h. The reaction mixture was diluted with CH_2Cl_2 (20 mL) and filtered. After concentration, the residue was purified by flash column chromatography to afford the desired product (69 mg, 83%) as a syrup. The two diastereomers could not be seperated by flash column chromatography and were obtained as a mixtue in a 1:1 dr ratio.

_N_-(_Tert_-butoxycarbonyl)-3-(1-(3,4,6-tri-_O_-benzoyl-2-deoxy-2-phthalimido-β-D-glucopyranosyl) eth-1-en-1-yl)- D/L-alanine methyl ester (**3ba-epimer**)

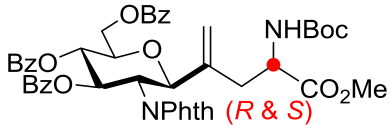

3ba-epimer, d.r. = 1:1

***S*-Diastereomer:** ^{1}H NMR (500 MHz, $CDCl_3$) δ 8.07 (d, *J* = 7.3 Hz, 2H), 7.90 (d, *J* = 7.4 Hz, 2H), 7.86 (d, *J* = 7.6 Hz, 1H), 7.74 (d, *J* = 7.4 Hz, 2H), 7.72–7.67 (m, 2H), 7.67–7.62 (m, 1H), 7.55 (t, *J* = 7.3 Hz, 1H), 7.47 (t, *J* = 7.8 Hz, 1H), 7.41 (q, *J* = 8.1 Hz, 3H), 7.32 (t, *J* = 7.6 Hz, 2H), 7.27–7.22 (m, 2H), 6.40 (dd, *J* = 10.4, 9.3 Hz, 1H), 5.69 (t, *J* = 9.7 Hz, 1H), 5.52 (d, *J* = 8.3 Hz, 1H), 5.10 (s, 1H), 5.06 (d, *J* = 10.6 Hz, 1H), 4.94 (s, 1H), 4.69–4.61 (m, 2H), 4.49 (ddt, *J* = 12.4, 8.5, 4.4 Hz, 2H), 4.27 (ddd, *J* = 10.3, 5.0, 3.0 Hz, 1H), 3.65 (s, 1H), 2.73 (dd, *J* = 15.5, 3.9 Hz, 1H), 2.53 (dd, *J* = 15.5, 8.6 Hz, 1H), 1.30 (s, 9H); ^{13}C NMR (125 MHz, $CDCl_3$) δ 172.51, 167.99, 167.63, 166.24, 165.70, 165.31, 155.51, 139.29, 134.51, 134.24, 133.50, 133.36, 133.19, 131.63, 130.98, 129.95, 129.92, 129.89, 129.81, 129.72, 128.87, 128.68, 128.46, 128.37, 123.75, 119.43, 79.63, 79.49, 76.11, 71.80, 70.20, 63.18, 52.90, 52.57, 52.17, 32.68, 28.28. ***R*-Diastereomer:** ^{1}H NMR (500 MHz, $CDCl_3$) δ 8.11 (d, *J* = 7.0 Hz, 2H), 7.91 (d, *J* = 7.0 Hz, 2H), 7.88 (d, *J* = 7.3 Hz, 1H), 7.77–7.62 (m, 5H), 7.57 (q, *J* = 6.3, 5.4 Hz, 1H), 7.51–7.44 (m, 3H), 7.41 (t, *J* = 7.5 Hz, 1H), 7.33 (t, *J* = 7.9 Hz, 2H), 7.25 (t, *J* = 7.4 Hz, 2H), 6.38 (t, *J* = 9.8 Hz, 1H), 5.72 (t, *J* = 9.7 Hz, 1H), 5.68 (d, *J* = 7.2 Hz, 1H), 5.19 (s, 1H), 5.11 (s, 1H), 5.09 (d, *J* = 10.5 Hz, 1H), 4.62 (t, *J* = 10.4 Hz, 1H), 4.60 (s, 2H), 4.33–4.25 (m, 2H), 3.71 (s, 3H), 2.57 (d, *J* = 7.3 Hz, 2H), 1.31 (s, 9H); ^{13}C NMR (125

MHz, $CDCl_3$) δ 172.76, 168.09, 167.36, 166.29, 165.75, 165.24, 155.60, 139.57, 134.53, 134.32, 133.51, 133.38, 133.22, 131.59, 130.98, 130.04, 129.96, 129.93, 129.81, 129.65, 128.87, 128.64, 128.55,128.46, 128.37, 123.77, 119.96, 79.71, 79.05, 75.92, 71.78, 69.79, 62.68,54.26, 52.89, 52.33, 31.91, 28.28

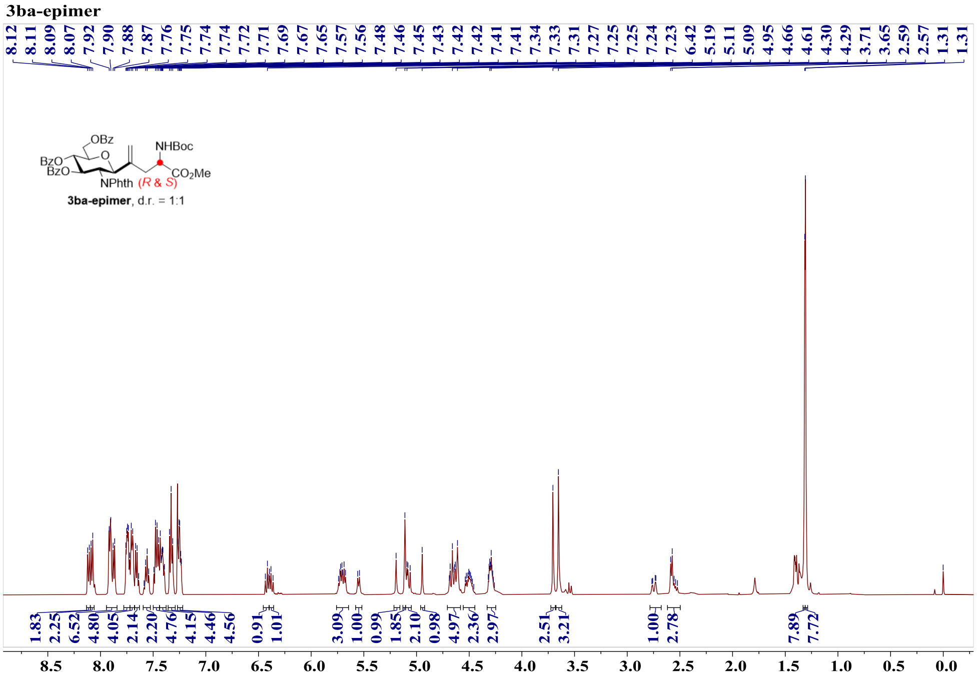

Supplementary Figure 38. ^{1}H NMR spectrum of the 1:1 mixture of **3ba** epimers.

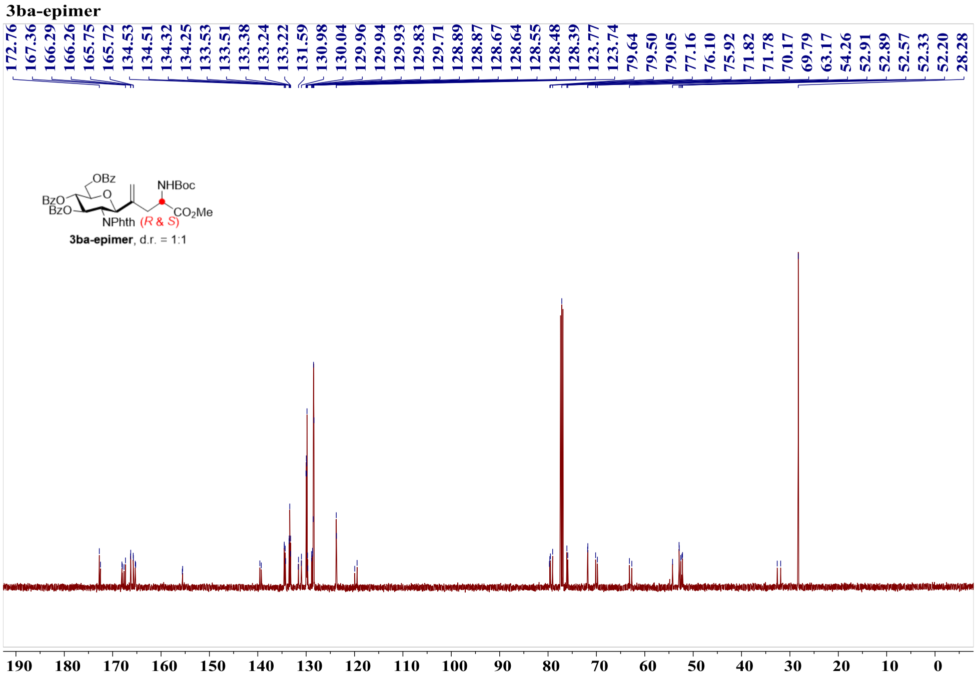

Supplementary Figure 39. ^{13}C NMR spectrum of the 1:1 mixture of **3ba** epimers.

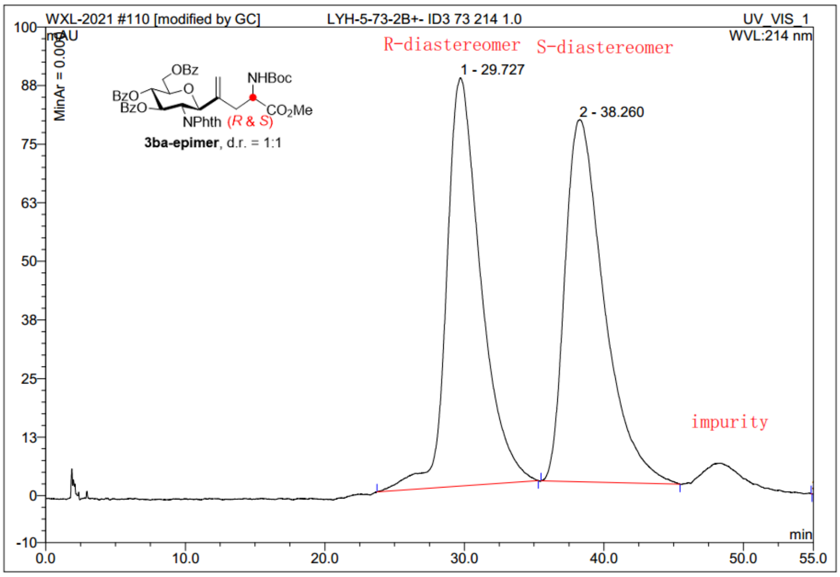

No.	Ret.Time min	Peak Name	Height mAU	Area mAU*min	Rel.Area %	Amount	Type
1	29.73	n.a.	87.140	248.165	49.71	n.a.	BMB*
2	38.26	n.a.	77.281	251.066	50.29	n.a.	BMB*
3	54.89	n.a.	0.033	0.001	0.00	n.a.	BMB
Total:			164.454	499.232	100.00	0.000	

Supplementary Figure 40. HPLC chromatogram of the 1:1 mixture of **3ba** epimers (Chiralpak® ID3, *n*-hexane/*i*-PrOH 70:30, 1.0 mL/min, detection at 214 nm)

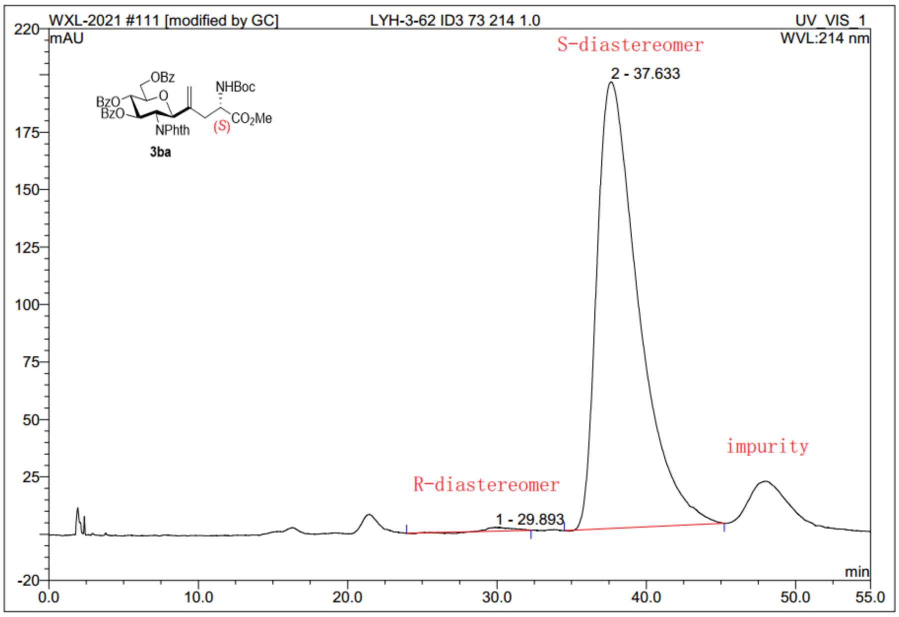

No.	Ret.Time min	Peak Name	Height mAU	Area mAU*min	Rel.Area %	Amount	Type
1	29.89	n.a.	1.636	2.287	0.36	n.a.	BMB*
2	37.63	n.a.	194.434	627.281	99.64	n.a.	BMB*
Total:			196.070	629.567	100.00	0.000	

Supplementary Figure 41. HPLC chromatogram of **3ba** prepared by the present method (Chiralpak® ID3, *n*-hexane/*i*-PrOH 70:30, 1.0 mL/min, detection at 214 nm).

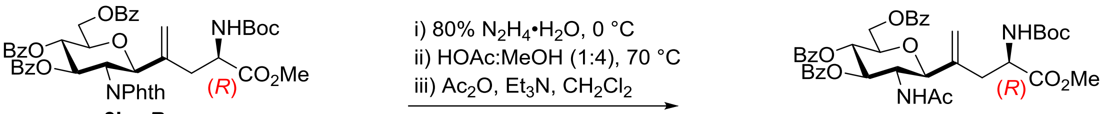

3ba-*R* **8-*R***

In addition, the synthesized **3ba-*R*** was subjected to the deprotection procudure mentioned above, and the resulting **8-*R*** was obtained as a standard to check the retention of chirality in compund **8**.

***N*-(*Tert*-butoxycarbonyl)-3-(1-(3,4,6-tri-*O*-benzoyl-2-deoxy-2-acetylamido-β-D-glucopyranosyl)eth-1-en-1-yl)-*D*-alanine methyl ester (8-*R*)**

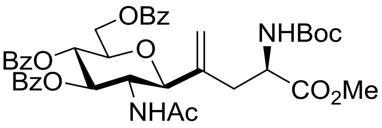

8-*R*

White powder, 18 mg, 55%. $[\alpha]_D^{25}$ –20.6 (*c* 1.0, $CHCl_3$); ^1H NMR (500 MHz, $CDCl_3$) δ 8.07 (d, *J* = 7.0 Hz, 2H), 7.90 (d, *J* = 8.1 Hz, 2H), 7.86 (d, *J* = 7.8 Hz, 2H), 7.55 (t, *J* = 7.5 Hz, 1H), 7.52–7.40 (m, 4H), 7.34 (t, *J* = 7.7 Hz, 2H), 7.31 (t, *J* = 7.7 Hz, 2H), 5.92 (d, *J* = 9.2 Hz, 1H), 5.70 (d, *J* = 6.7 Hz, 1H), 5.66 (d, *J* = 9.6 Hz, 1H), 5.63 (d, *J* = 9.8 Hz, 1H), 5.22 (s, 2H), 4.56 (d, *J* = 3.3 Hz, 2H), 4.36 (q, *J* = 9.8 Hz, 1H), 4.31–4.24 (m, 1H), 4.11 (dt, *J* = 8.4, 3.2 Hz, 1H), 4.05 (d, *J* = 10.2 Hz, 1H), 3.71 (s, 3H), 2.72 (dd, *J* = 15.1, 4.5 Hz, 1H), 2.53 (dd, *J* = 14.9, 10.1 Hz, 1H), 1.80 (s, 3H), 1.33 (s, 9H); ^{13}C NMR (125 MHz, $CDCl_3$) δ 172.93, 170.30, 167.22, 166.35, 165.18, 155.76, 140.14, 133.76, 133.55, 133.28, 130.02, 129.97, 129.81, 129.60, 128.93, 128.71, 128.63, 128.58, 128.55, 119.44, 83.09, 79.79, 75.92, 74.52, 69.09, 62.69, 54.43, 52.41, 52.38, 32.61, 28.35, 23.27.

The **8-epimer** was prepared via mixing compound **8** and compound **8-*R*** in 53.3:47.7 ratio (as determined by chiral HPLC). The retention of chirality in compound **8** was proven by chiral HPLC analysis (Supplementary Figures 42 and 43).

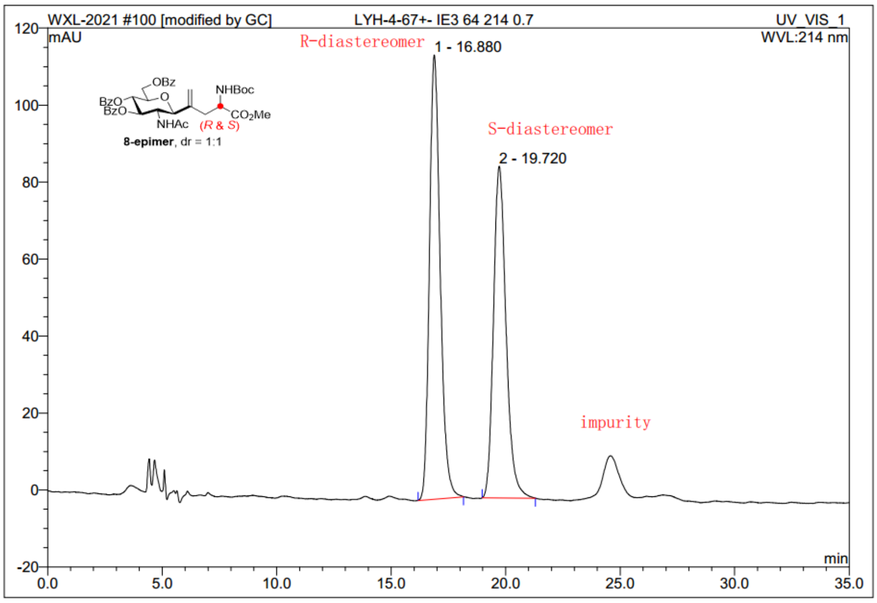

No.	Ret.Time min	Peak Name	Height mAU	Area mAU*min	Rel.Area %	Amount	Type
1	16.88	n.a.	115.431	62.648	53.32	n.a.	BMB*
2	19.72	n.a.	86.206	54.854	46.68	n.a.	BMB*
Total:			201.637	117.502	100.00	0.000	

Supplementary Figure 42. HPLC chromatogram of the mixture of **8-*R*** and **8**(*S*) prepared by the present method (Chiralpak® ID3, *n*-hexane/*i*-PrOH 70:30, 1.0 mL/min, detection at 214 nm).

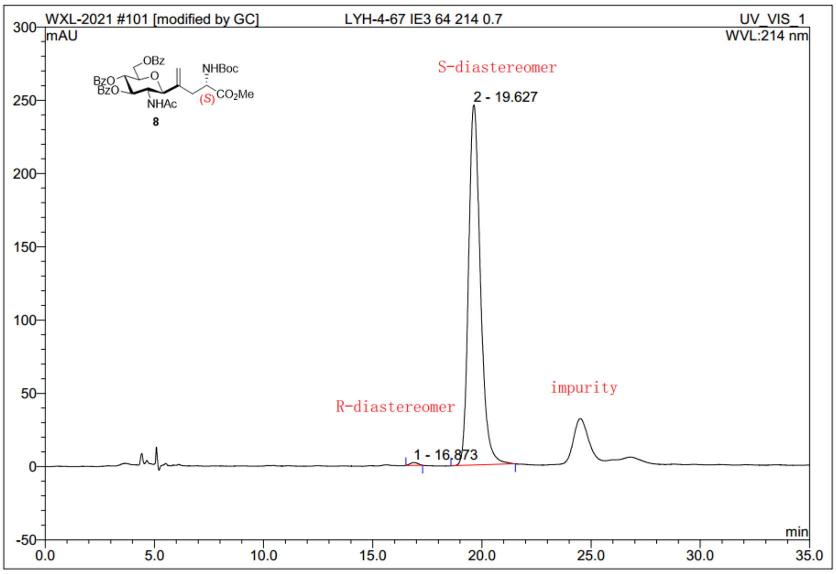

No.	Ret.Time min	Peak Name	Height mAU	Area mAU*min	Rel.Area %	Amount	Type
1	16.87	n.a.	1.885	0.785	0.50	n.a.	BMB*
2	19.63	n.a.	245.899	154.842	99.50	n.a.	BMB*
Total:			247.784	155.627	100.00	0.000	

Supplementary Figure 43. HPLC chromatogram of **8** prepared by the present method (Chiralpak® ID3, *n*-hexane/*i*-PrOH 70:30, 1.0 mL/min, detection at 214 nm).

Saccharide chain elongation

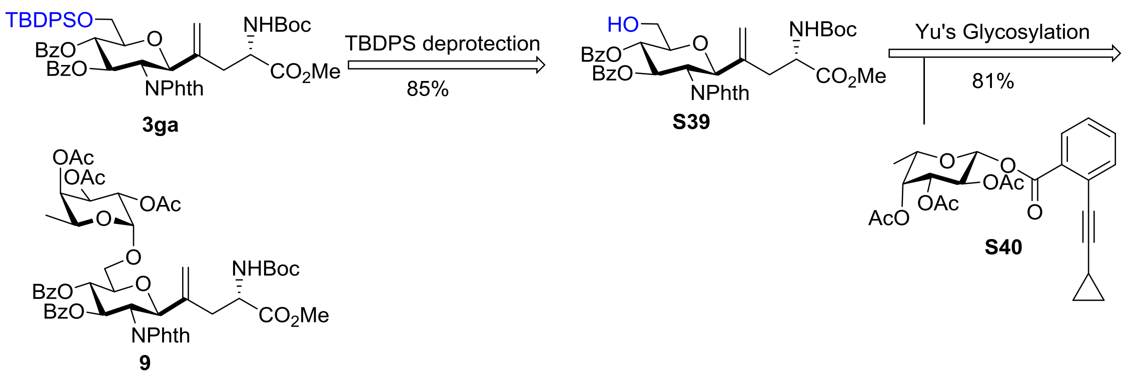

Supplementary Figure 44. Preparation of vinyl *C*-disaccharide amino acid **9**.

To a stirred solution of **3ga** (596.7 g, 0.62 mmol) in pyridine (20 mL) was added HF-pyridine complex (2 mL) at 0 ℃. The mixture was gradually warmed up to room temperature and stirred for 2 h. The reaction mixture was diluted with EtOAc, washed with sat. aq. $NaHCO_3$ and brine. After dried over Na_2SO_4 and concentrated, the residue was purified by silica gel flash column chromatography (petroleum ether/ethyl acetate = 2:1) to afford **S39** (382 mg, 85%).

***N*-(*Tert*-butoxycarbonyl)-3-(1-(3,4-di-*O*-benzoyl-2-deoxy-2-phthalimido-β-D-glucopyranosyl)eth-1-en-1-yl)-L-alanine methyl ester (S39)**

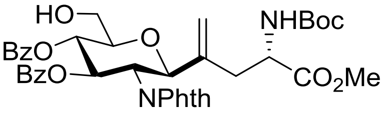

S39

^{1}H NMR (500 MHz, $CDCl_3$) δ 7.94 (d, J = 7.2 Hz, 2H), 7.86 (d, J = 7.3 Hz, 1H), 7.75 (d, J = 7.8 Hz, 1H), 7.50 (t, J = 7.4 Hz, 3H), 7.42 (t, J = 7.4 Hz, 1H), 7.36 (t, J = 7.8 Hz, 2H), 7.26 (t, J = 7.8 Hz, 2H), 6.38 (t, J = 9.8 Hz, 1H), 5.57 (t, J = 9.6 Hz, 1H), 5.42 (d, J = 9.3 Hz, 1H), 5.11 (s, 1H), 5.05 (d, J = 10.5 Hz, 1H), 4.90 (s, 1H), 4.78 (td, J = 9.6, 3.9 Hz, 1H), 4.65 (t, J = 10.4 Hz, 1H), 3.98–3.91 (m, 1H), 3.78 (d, J = 8.7 Hz, 4H), 3.73–3.67 (m, 1H), 2.83 (dd, J = 14.7, 3.9 Hz, 1H), 2.32 (dd, J = 14.5, 9.7 Hz, 1H), 1.43 (s, 9H); ^{13}C NMR (125 MHz, $CDCl_3$) δ 172.86, 167.99, 167.52, 165.80, 165.72, 155.58, 139.05, 134.50, 134.28, 133.58, 133.36, 131.69, 131.05, 130.02, 129.85, 128.98, 128.85, 128.53, 128.42, 123.76, 121.55, 80.45, 79.81, 79.05, 71.93, 70.20, 61.86, 53.06, 52.78, 52.47, 34.36, 28.44; HRMS (ESI) calcd. for $C_{39}H_{40}N_2O_{12}Na$ $(M + Na)^+$ m/z 751.2473, found 751.2476.

Compound **S39** (36.4 mg, 0.05 mmol), 2,3,4-tri-*O*-acetyl-β-L-fucopyranosyl 2-(cyclopropylethynyl)benzoate **S40** (22.9 mg, 1.0 equiv.), and 4Å MS (50 mg) were suspended in extra dry CH_2Cl_2 (1.0 mL). The mixture was stirred at room temperature for 0.5 h, then cooled to 0 ℃. $Au(PPh_3)NTf_2$ (3.6 mg, 10 mol%) was added in one portion. The reaction mixture was stirred for 0.5 h at room temperature, and was quenched with Et_3N (10 μL). The mixture was diluted with CH_2Cl_2 (20 mL) and filtrated. After concentration, the residue was purified by preparative TLC (petroleum ether/dichloromethane/ethyl acetate = 2:1:1) to afford **9** (40 mg, 81%) as a white foam.

N-(*Tert*-butoxycarbonyl)-3-(1-(3,4-di-*O*-benzoyl-6-*O*-(2,3,4-tri-*O*-acetyl-α-L-fucopyranosyl)-2-deoxy-2-phthalimido-β-D-glucopyranosyl)eth-1-en-1-yl)-L-alanine methyl ester (9)

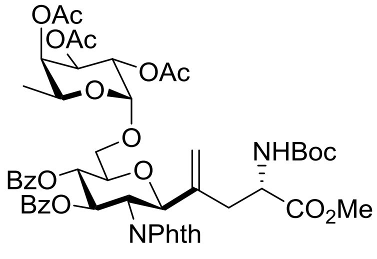

9

$[\alpha]_D^{25}$ 2.5 (*c* 1.0, $CHCl_3$); ^1H NMR (500 MHz, $CDCl_3$) δ 7.91 (d, *J* = 7.9 Hz, 2H), 7.86 (d, *J* = 7.2 Hz, 1H), 7.69 (dt, *J* = 19.2, 7.5 Hz, 5H), 7.49 (t, *J* = 7.4 Hz, 1H), 7.42 (t, *J* = 7.4 Hz, 1H), 7.35 (t, *J* = 7.6 Hz, 2H), 7.30–7.23 (m, 2H), 6.30 (t, *J* = 9.8 Hz, 1H), 5.62 (t, *J* = 9.7 Hz, 1H), 5.46 (d, *J* = 8.7 Hz, 1H), 5.27–5.19 (m, 2H), 5.17–5.13 (m, 1H), 5.11 (s, 1H), 5.00 (d, *J* = 10.8 Hz, 1H), 4.85 (d, *J* = 7.9 Hz, 1H), 4.62–4.52 (m, 2H), 4.10 (d, *J* = 10.0 Hz, 1H), 4.04–3.96 (m, 2H), 3.96–3.88 (m, 1H), 3.74 (s, 3H), 2.85 (dd, *J* = 16.1, 5.5 Hz, 1H), 2.45 (dd, *J* = 16.1, 8.6 Hz, 1H), 2.24 (s, 3H), 2.15 (s, 3H), 1.99 (s, 3H), 1.40 (s, 9H), 1.11 (d, *J* = 6.4 Hz, 3H); ^{13}C NMR (125 MHz, $CDCl_3$) δ 172.89, 170.87, 170.32, 169.63, 167.98, 167.66, 165.75, 164.92, 155.56, 139.55, 134.51, 134.24, 133.36, 133.32, 131.63, 130.98, 129.90, 129.76, 129.19, 128.79, 128.39, 123.78, 123.72, 118.56, 100.75, 79.95, 79.79, 78.24, 72.17, 71.52, 70.74, 69.44, 69.38, 69.05, 66.87, 52.85, 52.46, 51.91, 32.07, 28.39, 21.04, 20.81, 20.79, 16.08; HRMS (ESI) calcd. for $C_{51}H_{56}N_2O_{19}Na$ $(M + Na)^+$ m/z 1023.3369, found 1023.3375.

Peptide chain elongation

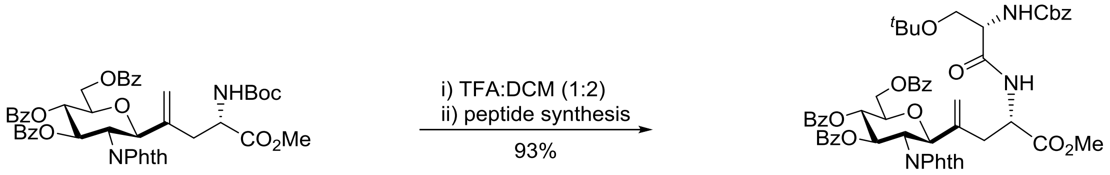

Supplementary Figure 45. Preparation of vinyl *C*-glycosyl dipeptide **10**.

To a stirred solution of **3ba** (83.3 mg, 0.1 mmol) in dry CH_2Cl_2 (1 mL) was added TFA (0.5 mL) at 0 ℃. The mixture was stirred for 1.5 h at room temperature to remove the Boc group. The solution was then concentrated and dried *in vacuo*. The residue was dissolved in dry DMF (1 mL), and cooled to -10 ℃. *N*-((Benzyloxy)carbonyl)-*O*-(*tert*-butyl)-L-serine (45 mg, 1.5 equiv.) and HOBt (1.5 equiv.) were dissolved in dry DMF (1 mL), and this solution was transfered into the above solution slowly under -10 ℃. DIPEA (70 μL, 4.0 equiv.) was injected followed by addition of EDCI (1.5 equiv.). The reaction mixture was stirred under -10 ℃ for 0.5 h, and was then warmed up to room temperature and stirred for 6 h. After removal of solvent, the residue was purified by column chromatography (petroleum ether/ethyl acetate = 1:1) to provide **10** (93.3 mg, 92%) as a white solid.

***O*-*Tert*-butyl-*N*-(benzyloxycarbonyl-)-L-serinyl-3-(1-(3,4,6-tri-*O*-benzoyl-2-deoxy-2-phthalimido-β-D-glucopyranosyl)eth-1-en-1-yl)-L-alanine methyl ester (10)**

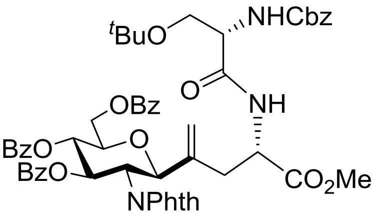

10

$[\alpha]_D^{25}$ 28.5 (*c* 0.9, $CHCl_3$); ^{1}H NMR (500 MHz, $CDCl_3$) *δ* 8.06 (d, *J* = 7.3 Hz, 2H), 7.91 (d, *J* = 7.4 Hz, 2H), 7.85 (d, *J* = 7.4 Hz, 1H), 7.76 (d, *J* = 7.5 Hz, 2H), 7.70 (d, *J* = 7.0 Hz, 1H), 7.64 (t, *J* = 7.3 Hz, 1H), 7.59–7.51 (m, 2H), 7.46 (t, *J* = 7.4 Hz, 1H), 7.45–7.38 (m, 3H), 7.37–7.28 (m, 7H), 7.28–7.22 (m, 2H), 7.09 (d, *J* = 8.2 Hz, 1H), 6.42 (t, *J* = 9.8 Hz, 1H), 5.72 (t, *J* = 9.7 Hz, 1H), 5.17–5.00 (m, 4H), 4.91 (s, 1H), 4.78 (td, *J* = 9.4, 3.9 Hz, 1H), 4.69 (dd, *J* = 12.1, 2.9 Hz, 1H), 4.65 (t, *J* = 10.4 Hz, 1H), 4.46 (dd, *J* = 12.2, 4.5 Hz, 1H), 4.29–4.13 (m, 2H), 3.76 (dd, *J* = 8.9, 3.9 Hz, 1H), 3.61 (s, 3H), 3.37 (t, *J* = 8.1 Hz, 1H), 2.84 (dd, *J* = 15.8, 3.9 Hz, 1H), 2.46 (dd, *J* = 15.7, 10.1 Hz, 1H), 1.15 (s, 9H); ^{13}C NMR (125 MHz, $CDCl_3$) *δ* 171.91, 170.18, 168.01, 167.71, 166.21, 165.69, 165.26, 155.89, 139.10, 136.37, 134.46, 134.36, 133.43, 133.31, 133.11, 131.53, 129.87, 129.86, 129.77, 129.69, 128.89, 128.55, 128.41, 128.33, 128.18, 128.14, 123.76, 123.72, 118.13, 79.24, 75.98, 74.08, 71.83, 70.13, 66.91, 63.06, 61.76, 54.32, 52.96, 52.32, 50.84, 33.14, 27.35; HRMS (ESI) calcd. for $C_{56}H_{55}N_3O_{15}Na$ $(M + Na)^+$ m/z 1032.3525, found 1032.3527.

Deprotection of ester groups

***N*-(*Tert*-butoxycarbonyl)-3-(1-(α-D-mannopyranosyl)eth-1-en-1-yl)-L-alanine** (**11**)

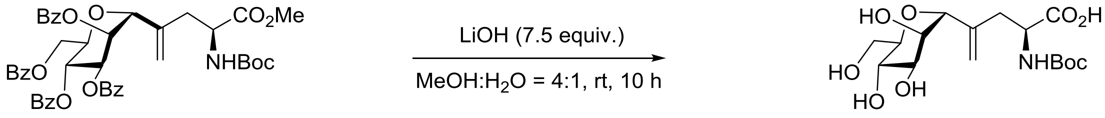

3aa **11**, quant.

To a Schlenk flasks (10 mL) equipped with a stirr bar were added **3aa** (41.0 mg, 0.05 mmol), LiOH (9 mg, 7.5 equiv.), MeOH (4.0 mL) and deionized water (1.0 mL). The mixture was stirred at room temperature for 10 h, after which, HOAc (24 µL) was added and the mixture was stirred for 20 mins. After dilution with MeOH (5.0 mL) and concentration, the mixture was purified by column chromatography (CH_2Cl_2/MeOH using a gradient ratio of 10/1, 5/1, 2/1, 1/1) to provide **11** as a white solid (19 mg, 100%): $[\alpha]_D^{25}$ 31.8 (*c* 0.9, MeOH); ^{1}H NMR (500 MHz, D_2O) δ 5.22 (d, *J* = 9.9 Hz, 1H), 5.12 (s, 1H), 4.40 (d, *J* = 12.1 Hz, 1H), 4.29 (t, *J* = 2.8 Hz, 1H), 4.02–3.91 (m, 1H), 3.77 (ddd, *J* = 10.9, 9.4, 2.7 Hz, 1H), 3.68 (dd, *J* = 12.3, 6.7 Hz, 1H), 3.59 (t, *J* = 8.9 Hz, 1H), 3.34 (t, *J* = 6.9 Hz, 1H), 2.49–2.33 (m, 2H), 1.35 (s, 9H); ^{13}C NMR (126 MHz, D_2O) δ 179.18, 157.29, 140.17, 116.58, 80.90, 78.07, 74.96, 70.66, 68.90, 67.49, 60.95, 54.90, 35.57, 27.51; HRMS (ESI) calcd. for $C_{16}H_{27}NO_9Na$ $(M + Na)^+$ m/z 400.1587, found 400.1579.

***N*-(*Tert*-butoxycarbonyl)-3-(1-(2-deoxy-2-acetylamido-β-D-glucopyranosyl)eth-1-en-1-yl)-L-alanine** (**12**)

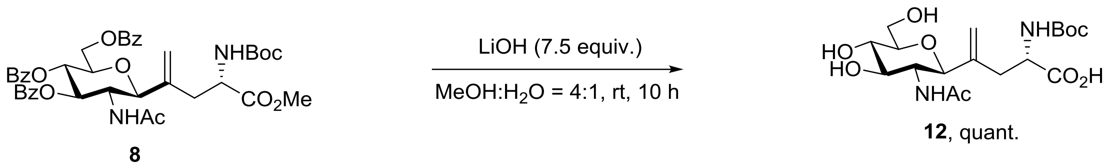

To a Schlenk flasks (10 mL) equipped with a stirr bar were added **8** (37.5 mg, 0.05 mmol), LiOH (9 mg, 7.5 equiv.), MeOH (4.0 mL) and deionized water (1.0 mL). The mixture was stirred at room temperature for 10 h, after which, HOAc (24 μL) was added and the mixture was stirred for 20 mins. After dilution with MeOH (5.0 mL) and concentration, the mixture was purified by column chromatography (CH_2Cl_2/MeOH using a gradient ratio of 5/1, 2/1, 1/1, 1/2, pure MeOH) to provide **12** as a white solid (22 mg, 100%): $[\alpha]_D^{25}$ 10.3 (*c* 1.0, MeOH); ^{1}H NMR (500 MHz, CD_3OD) δ 5.06 (s, 1H), 4.99 (s, 1H), 4.30 (dd, *J* = 10.1, 3.6 Hz, 1H), 3.88 (t, *J* = 10.0 Hz, 1H), 3.85 (dd, *J* = 12.7, 2.5 Hz, 1H), 3.77 (d, *J* = 10.2 Hz, 1H), 3.70 (dd, *J* = 12.1, 6.0 Hz, 2H), 3.44 (t, *J* = 9.2 Hz, 2H), 3.35 (t, *J* = 10.9 Hz, 1H), 3.26 (ddd, *J* = 9.2, 6.3, 2.1 Hz, 1H), 2.75 (dd, *J* = 15.5, 3.5 Hz, 1H), 2.34 (dd, *J* = 15.4, 10.1 Hz, 1H), 1.91 (s, 3H), 1.42 (s, 9H); ^{13}C NMR (125 MHz, CD_3OD) δ 173.53, 157.99, 143.51, 117.88, 84.87, 82.03, 80.02, 77.36, 72.27, 63.21, 55.68, 54.70, 49.03, 35.60, 28.87, 22.95; HRMS (ESI) calcd. for $C_{18}H_{30}N_2O_9Na$ $(M + Na)^+$ m/z 441.1844, found 441.1841.

N-(Tert-butoxycarbonyl)-3-(1-(α-D-mannopyranosyl)eth-1-en-1-yl)-L-alanyl-L-alanyl-O-(tert-butyl)-L-threonine (13)

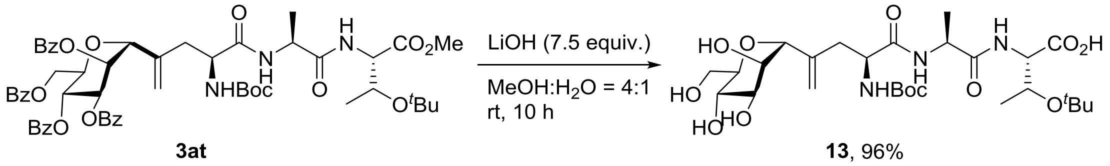

To a Schlenk flasks (10 mL) equipped with a stirr bar were added **3at** (52.0 mg, 0.05 mmol), LiOH (9 mg, 7.5 equiv.), MeOH (4.0 mL) and deionized water (1.0 mL). The mixture was stirred at room temperature for 10 h, after which, HOAc (24 μL) was added and the mixture was stirred for 20 mins. After dilution with MeOH (5.0 mL) and concentration, the mixture was purified by column chromatography (CH_2Cl_2/MeOH using a gradient ratio of 10/1, 5/1, 2/1, 1/1, 1/2) to provide **13** as a white solid (29 mg, 96%): $[\alpha]_D^{25}$ -0.7 (*c* 1.0, MeOH); ^{1}H NMR (500 MHz, CD_3OD) δ 5.21 (s, 1H), 5.17 (s, 1H), 4.49 (q, *J* = 7.0 Hz, 1H), 4.45 (d, *J* = 3.5 Hz, 1H), 4.33 (t, *J* = 7.5 Hz, 1H), 4.28–4.22 (m, 2H), 4.18 (t, *J* = 3.6 Hz, 1H), 3.83 (dd, *J* = 11.9, 6.9 Hz, 1H), 3.77 (dd, *J* = 12.0, 2.9 Hz, 1H), 3.73 (dd, *J* = 7.9, 3.2 Hz, 1H), 3.66 (t, *J* = 7.7 Hz, 1H), 3.42 (td, *J* = 7.3, 2.9 Hz, 1H), 2.53 (d, *J* = 7.4 Hz, 2H), 1.43 (s, 9H), 1.39 (d, *J* = 7.0 Hz, 3H), 1.19 (s, 9H), 1.14 (d, *J* = 6.1 Hz, 3H); ^{13}C NMR (12 MHz, CD_3OD) δ 174.38, 174.09, 157.73, 143.19, 117.52, 80.72, 78.46, 78.02, 75.03, 72.72, 70.16, 69.76, 69.40, 62.57, 60.88, 55.01, 50.48, 37.33, 28.94, 28.74, 21.55, 18.37; HRMS (ESI) calcd. for $C_{27}H_{47}N_3O_{12}Na$ $(M + Na)^+$ m/z 628.3052, found 628.3054.

2.8 Radical clock experiment

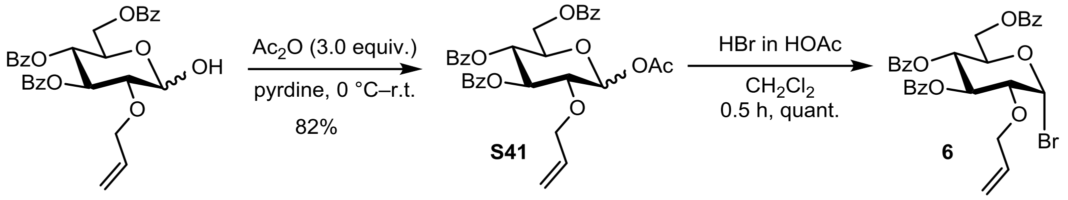

Supplementary Figure 46. Preparation of glycosyl bromide substrate **6**.

To a solution of 2-*O*-allyl-3,4,6-tri-*O*-benzoyl-α,β-D-glucopyranose[26] (533 mg, 1.0 mmol) in pyridine (4 mL) was added Ac_2O (282 μL, 3.0 equiv.) at 0 ℃. The mixture was stirred at room temperature for 3 h. After completion, MeOH (1.0 mL) was added and the mixture was kept stirring for 1 h. After concentration, the crude product was purifed by column chromatography (petroleum ether/ethyl acetate = 4:1) to provide 2-*O*-allyl-3,4,6-tri-*O*-benzoyl-α,β-D-glucopyranosyl acetate **S41** (470 mg, 82%) as a white solid: ^{1}H NMR (500 MHz, $CDCl_3$) δ 8.01 (d, *J* = 8.4 Hz, 2H), 7.96 (d, *J* = 8.2 Hz, 2H), 7.90 (dd, *J* = 14.5, 8.2 Hz, 2H), 7.57–7.47 (m, 3H), 7.43–7.29 (m, 6H), 6.48 (d, *J* = 3.6 Hz, 0.6H), 5.89 (t, *J* = 9.8 Hz, 1H), 5.83 (d, *J* = 7.9 Hz, 0.4H), 5.76–5.65 (m, 1.4H), 5.59 (dt, *J* = 19.5, 9.8 Hz, 1H), 5.18 (ddq, *J* = 18.9, 17.4, 1.6 Hz, 1H), 5.12 (dt, *J* = 10.3, 1.3 Hz, 0.6H), 5.06 (dq, *J* = 10.3, 1.3 Hz, 0.4H), 4.57 (ddt, *J* = 10.3, 7.5, 3.0 Hz, 1H), 4.47–4.34 (m, 1H), 4.22–4.16 (m, 1H), 4.15–4.05 (m, 1.4H), 4.02 (ddt, *J* = 13.0, 6.0, 1.3 Hz, 0.6H), 3.88 (dd, *J* = 10.0, 3.6 Hz, 0.6H), 3.76 (dd, *J* = 9.3, 8.0 Hz, 0.4H), 2.26 (s, 1.8H), 2.17 (s, 1.2H); ^{13}C NMR (125 MHz,$CDCl_3$) δ 169.32, 166.23, 165.93, 165.66, 165.44, 134.14, 133.92, 133.56, 133.39, 133.32, 133.23, 133.19, 129.99, 129.98, 129.91, 129.88, 129.85, 129.53, 129.41, 128.95, 128.89, 128.54, 128.53, 128.51, 128.49, 128.45, 118.54, 117.87, 93.95, 89.69, 77.97, 75.97, 74.31, 73.73, 72.91, 72.48, 71.83, 70.28, 69.29, 69.18, 63.04, 62.89, 21.23, 21.16; HRMS (ESI) calcd. for $C_{32}H_{30}O_{10}Na$ $(M + Na)^+$ m/z 597.1731, found 597.1737.

To a stirred solution of **S41** (173 mg, 0.3 mmol) in CH_2Cl_2 (2.0 mL) was added 33% HBr in HOAc (0.5 mL) dropwise under 0 ℃. The mixture was kept stirring for 0.5 h at 0 ℃, and was then diluted with CH_2Cl_2 (50 mL). The mixture was washed with ice-water (3×20 mL), sat. aq. $NaHCO_3$ (3×10 mL), and brine (2×20 mL) successively. The organic layer was dried over Na_2SO_4, filtered, and concentrated to give **6** as a white foam, which was in high purity and used directly in the coupling reaction. **6**: ^{1}H NMR (500 MHz, $CDCl_3$) δ 8.04 (dd, *J* = 8.4, 1.4 Hz, 2H), 7.94 (ddd, *J* = 8.0, 7.5, 1.4 Hz, 4H), 7.56 (ddt, *J* = 8.8, 7.2, 1.3 Hz, 1H), 7.53–7.47 (m, 2H), 7.43 (dd, *J* = 8.2, 7.5 Hz, 2H), 7.37 (dd, *J* = 16.2, 8.5 Hz, 4H), 6.61 (d, *J* = 3.8 Hz, 1H), 5.99 (t, *J* = 9.6 Hz, 1H), 5.78 (ddt, *J* = 17.3, 10.3, 5.8 Hz, 1H), 5.65 (t, *J* = 10.0 Hz, 1H), 5.25 (dq, *J* = 17.3, 1.5 Hz, 1H), 5.16 (dq, *J* = 10.3, 1.2 Hz, 1H), 4.68–4.60 (m, 2H), 4.49–4.43 (m, 1H), 4.14 (ddt, *J* = 13.0, 5.9, 1.3 Hz, 1H), 4.07 (ddt, *J* = 13.0, 5.8, 1.4 Hz, 1H), 3.78 (dd, *J* = 9.6, 3.8 Hz, 1H); ^{13}C NMR (125 MHz, $CDCl_3$) δ 166.14, 165.61, 165.41, 133.74, 133.67, 133.32, 130.05, 129.92, 129.84, 129.66, 129.46, 128.75, 128.57, 128.54, 128.50, 118.84, 89.35, 77.06, 72.68, 72.31, 72.21, 68.28, 62.26.

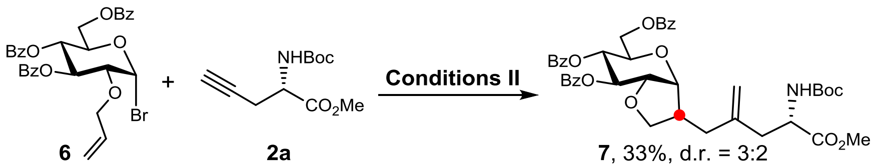

Supplementary Figure 47. The reaction of bromide **6** with alkyne **2a**.

To an oven-dried 10 mL Schlenk tube (Titan, TF891910) containing a Teflon coated magnetic stirring bar were added glycosyl bromide **6** (89.3 mg, 0.15 mmol), $NiCl_2$(DME) (10 mol%), dtbbpy (12 mol%), (*R*)-Tol-BINAP (10 mol%), and Na_2CO_3 (2.5 equiv). The tube was sealed with a rubber cap then with parafilm and evacuated then refilled with Ar for at least five cycles. Compound **2a** was dissolved in THF (1.5 mL) and injected into the reaction tube. When stirring, PMHS (48 µL, 2.5 equiv.) was injected via microliter syringe. The tube was kept stirring at 30 ℃ for 36 h. The mixture was diluted with CH_2Cl_2 (20 mL) and filtered. After concentration, the residue was purified by preparative TLC (toluene/ethyl acetate/CH_2Cl_2 = 5:0.5:1) to afford the **7** (36.8 mg, d.r. = 3:2) as a syrup: ^{1}H NMR (500 MHz, $CDCl_3$) δ 8.02 (d, *J* = 6.9 Hz, 2H), 8.01–7.90 (m, 4H), 7.58–7.47 (m, 3H), 7.45–7.32 (m, 6H), 5.75 (dd, *J* = 8.6, 6.2 Hz, 0.4 H), 5.68 (dd, *J* = 7.2, 4.6 Hz, 0.6 H), 5.46–5.38 (m, 1H), 5.10 (d, *J* = 8.1 Hz, 0.4 H), 4.95 (d, *J* = 8.3 Hz, 0.6 H), 4.91 (s, 0.4 H), 4.89 (s, 0.6 H), 4.87 (dd, *J* = 11.6, 4.3 Hz, 0.4 H), 4.84 (s, 0.4 H), 4.76 (s, 0.6 H), 4.70 (dd, *J* = 12.1, 6.9 Hz, 0.4 H), 4.56 (t, *J* = 4.2 Hz, 1H), 4.50 (dd, *J* = 12.4, 3.7 Hz, 0.6 H), 4.46 (dd, *J* = 11.4, 4.1 Hz, 0.6 H), 4.46 (m, 0.4 H), 4.43–4.33 (m, 1.6 H), 4.31–4.27 (m, 1 H), 4.24 (t, *J* = 5.8 Hz, 0.4 H), 4.03 (t, *J* = 7.8 Hz, 0.6 H), 3.77 (dd, *J* = 10.8, 7.9 Hz, 0.6 H), 3.54 (dd, *J* = 9.0, 5.5 Hz, 0.4 H), 2.69–2.42 (m, 2H), 2.40–2.30 (m, 1H), 2.30–2.20 (m, 1H), 2.19–2.09 (m, 1H), 1.42 (s, 3.6 H), 1.41 (s, 5.4 H); ^{13}C NMR (125 MHz, $CDCl_3$) δ 172.99, 166.31, 166.29, 165.77, 165.74, 165.43, 154.11, 142.33, 141.83, 133.53, 133.45, 133.35, 133.31, 133.29, 129.99, 129.95, 129.91, 129.87, 129.84, 129.79, 129.76, 129.74, 129.46, 129.38, 129.24, 129.08, 128.57, 128.54, 128.52, 128.51, 128.46, 115.39, 114.82, 80.33, 80.08, 79.04, 78.82, 77.16, 73.63, 73.07, 72.60, 72.08, 71.81, 71.57, 68.60, 67.78, 63.06, 62.48, 52.40, 52.35, 52.06, 51.99, 43.28, 42.01, 39.44, 38.97, 37.25, 30.33, 28.40; HRMS (ESI) calcd. for $C_{41}H_{45}NO_{12}Na$ $(M + Na)^+$ m/z 766.2834, found 766.2835.

3. NMR Spectra

Supplementary Figure 48. ^{1}H NMR spectrum of compound **1c** (500 MHz, $CDCl_3$, 25 °C)

1c

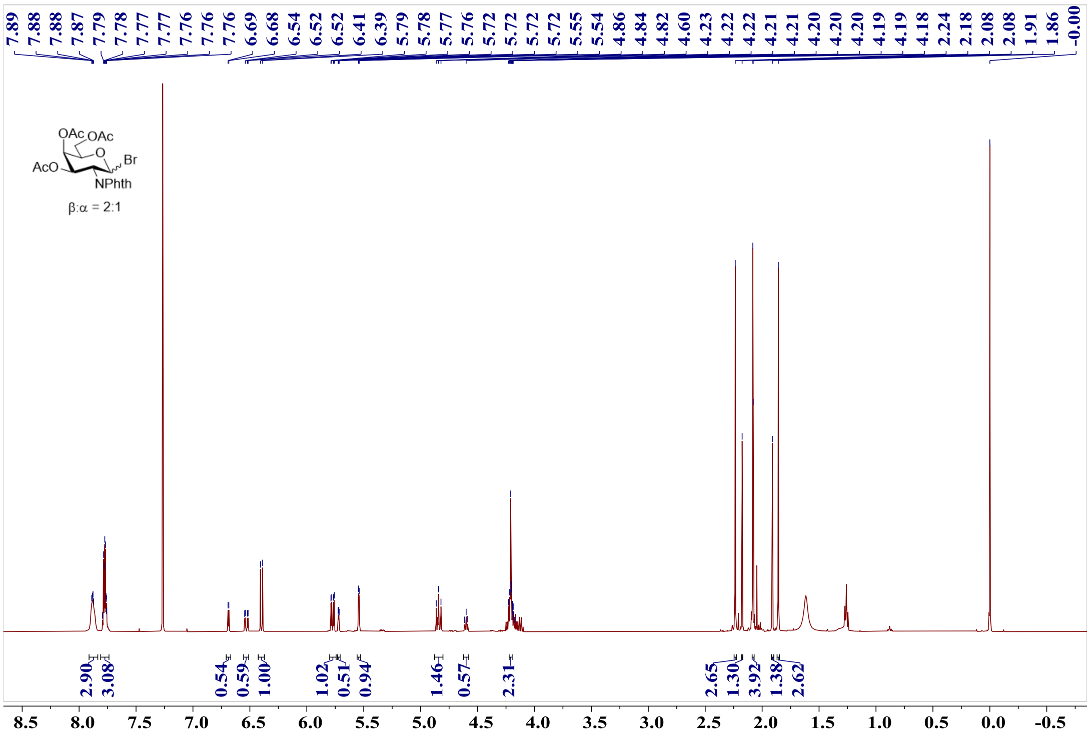

Supplementary Figure 49. ^{1}H NMR spectrum of compound **1d** (500 MHz, $CDCl_3$, 25 °C)

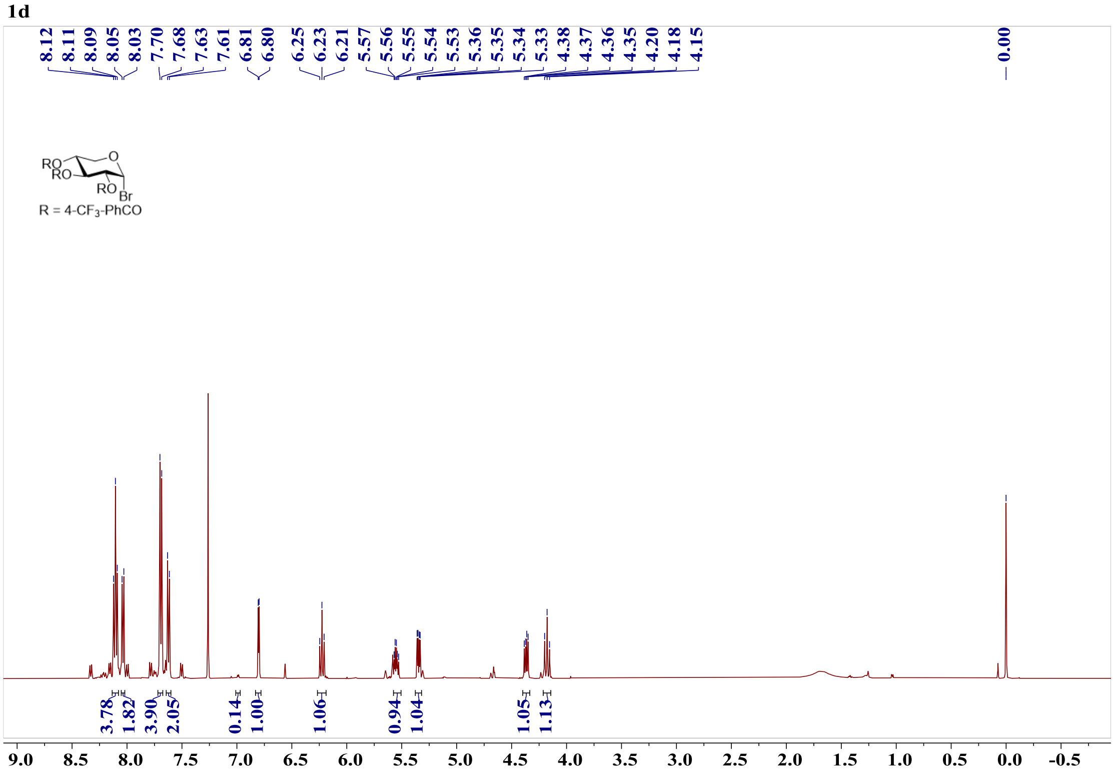

Supplementary Figure 50. ^{13}C NMR spectrum of compound **1d** (125 MHz, $CDCl_3$, 25 °C)

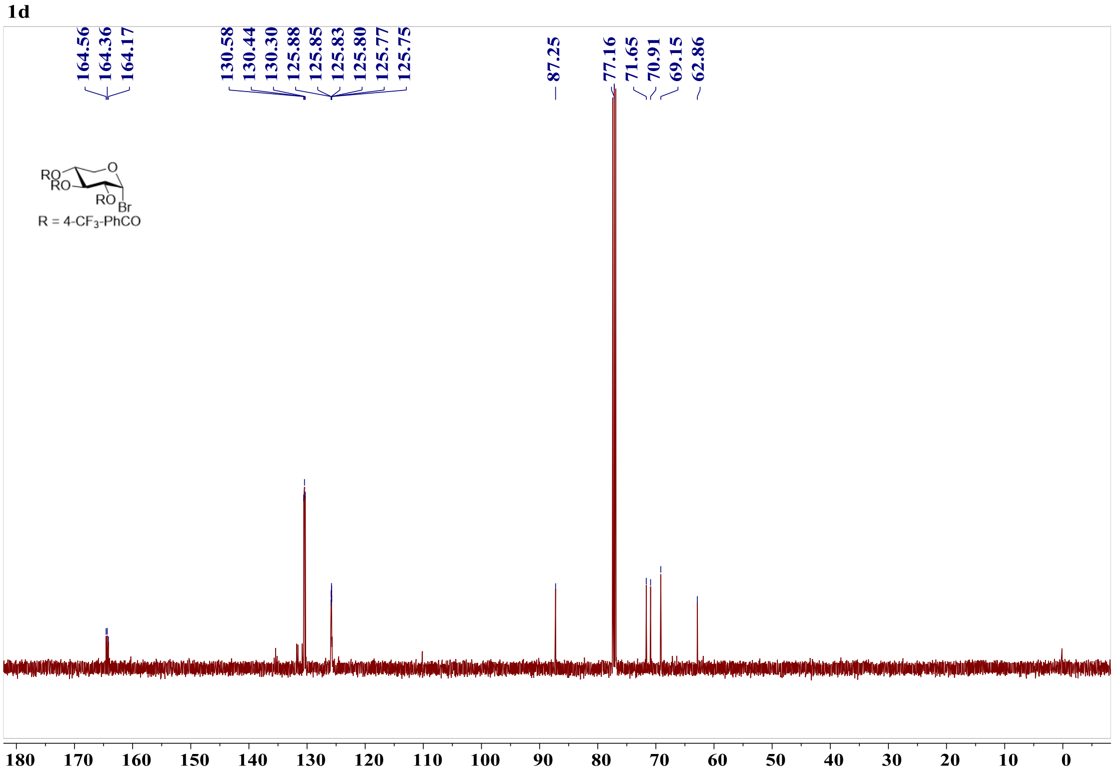

Supplementary Figure 51. ^{1}H NMR spectrum of compound **1e** (500 MHz, $CDCl_3$, 25 °C)

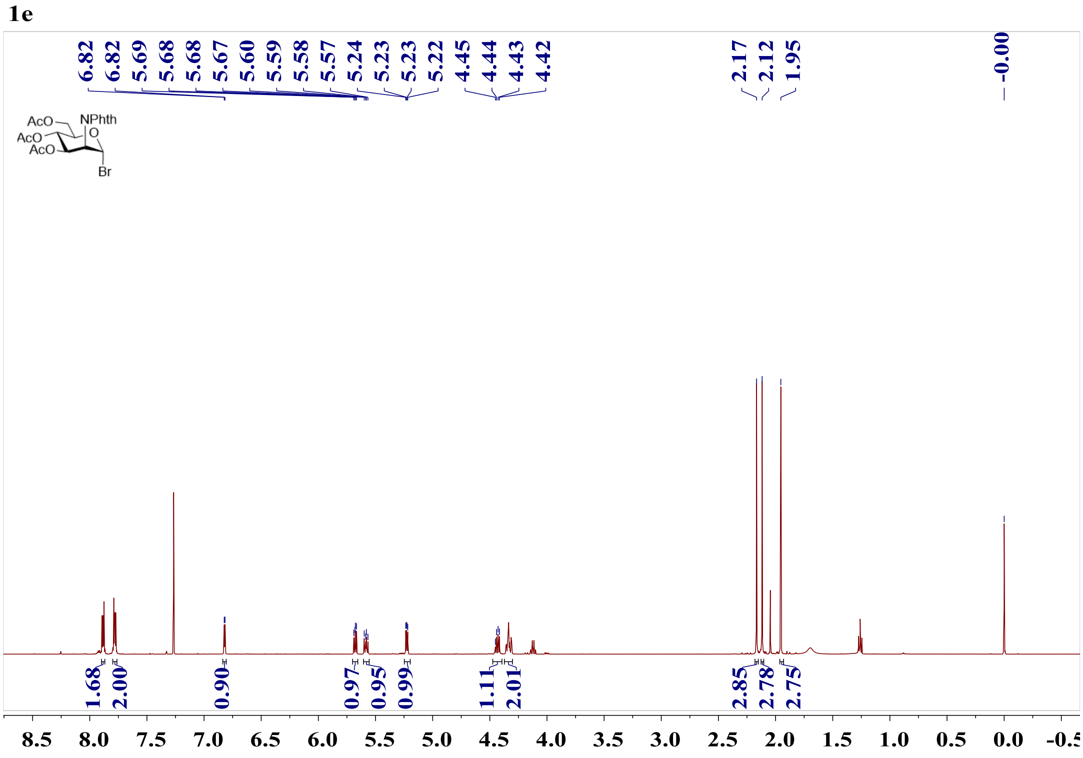

Supplementary Figure 52. ^{13}C NMR spectrum of compound **1e** (125 MHz, $CDCl_3$, 25 °C)

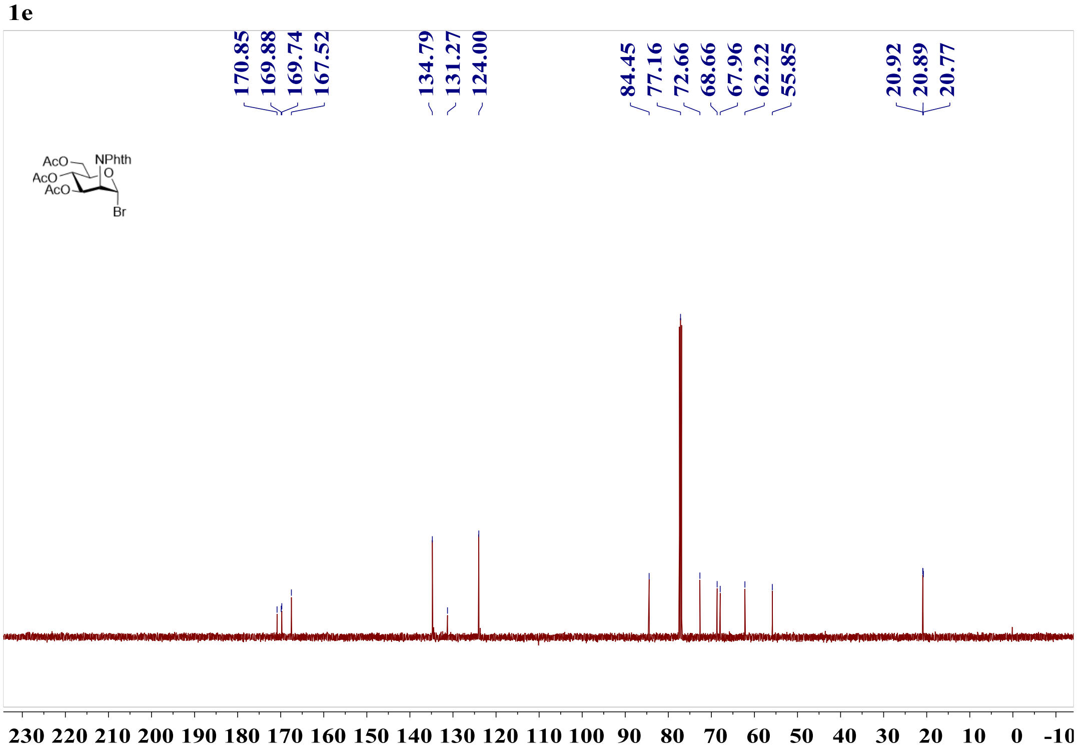

Supplementary Figure 53. ^{1}H NMR spectrum of compound **S3** (500 MHz, $CDCl_3$, 25 ^{o}C)

S3

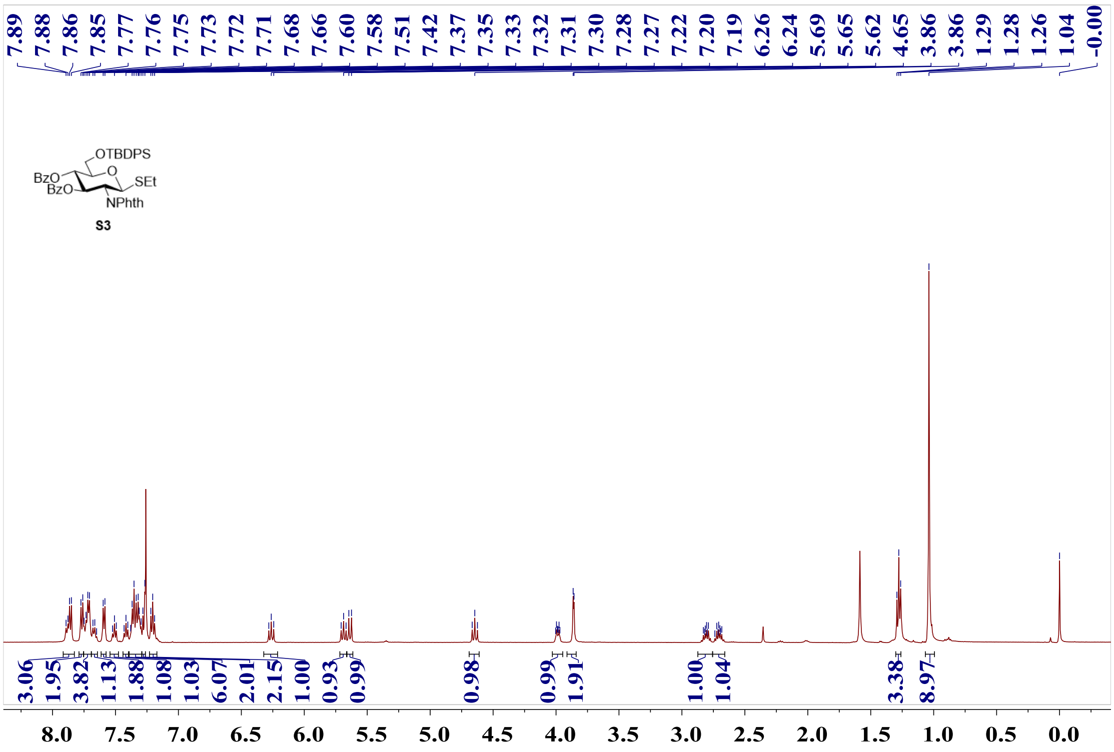

Supplementary Figure 54. ^{13}C NMR spectrum of compound **S3** (125 MHz, $CDCl_3$, 25 ^{o}C)

S3

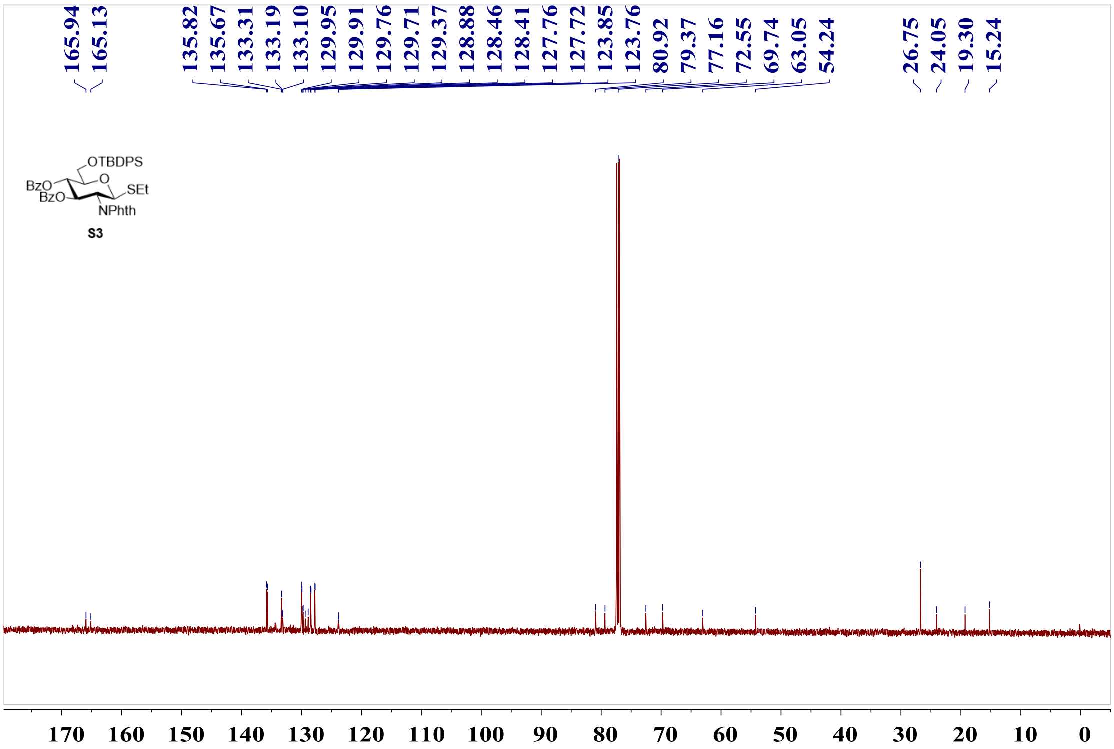

Supplementary Figure 55. ^{1}H NMR spectrum of compound **1g** (500 MHz, $CDCl_3$, 25 °C)

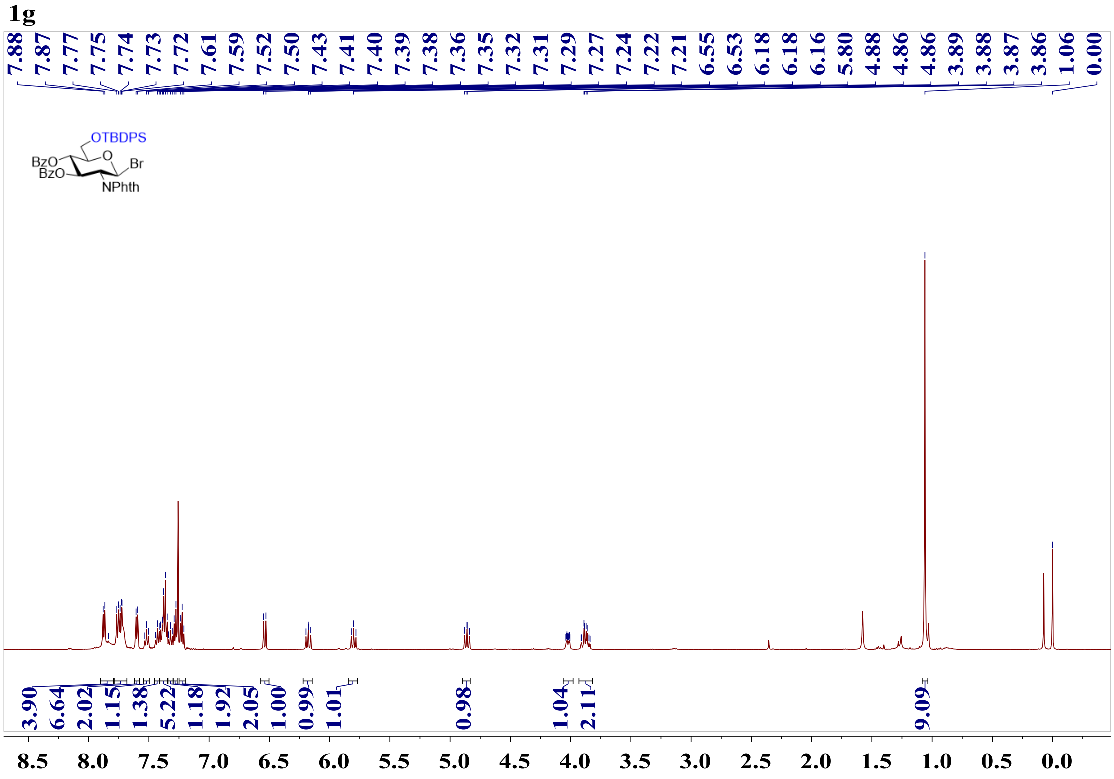

Supplementary Figure 56. ^{13}C NMR spectrum of compound **1g** (125 MHz, $CDCl_3$, 25 °C)

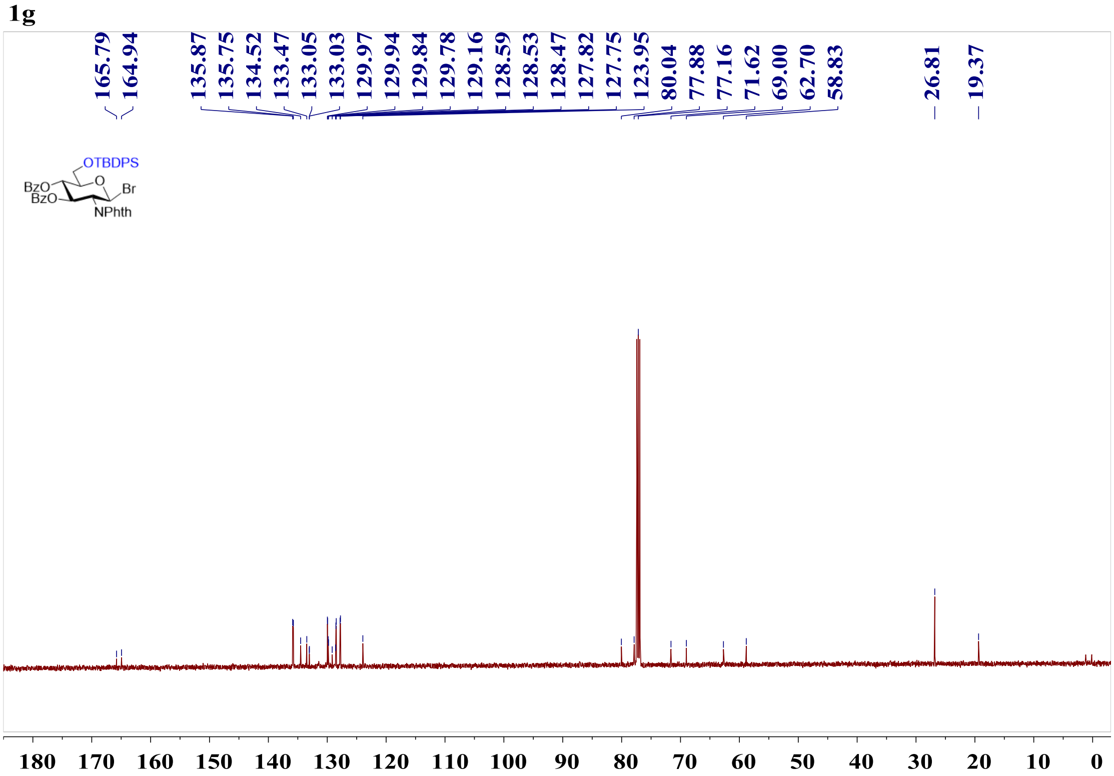

Supplementary Figure 57. ^{1}H NMR spectrum of compound **S6** (500 MHz, $CDCl_3$, 25 °C)

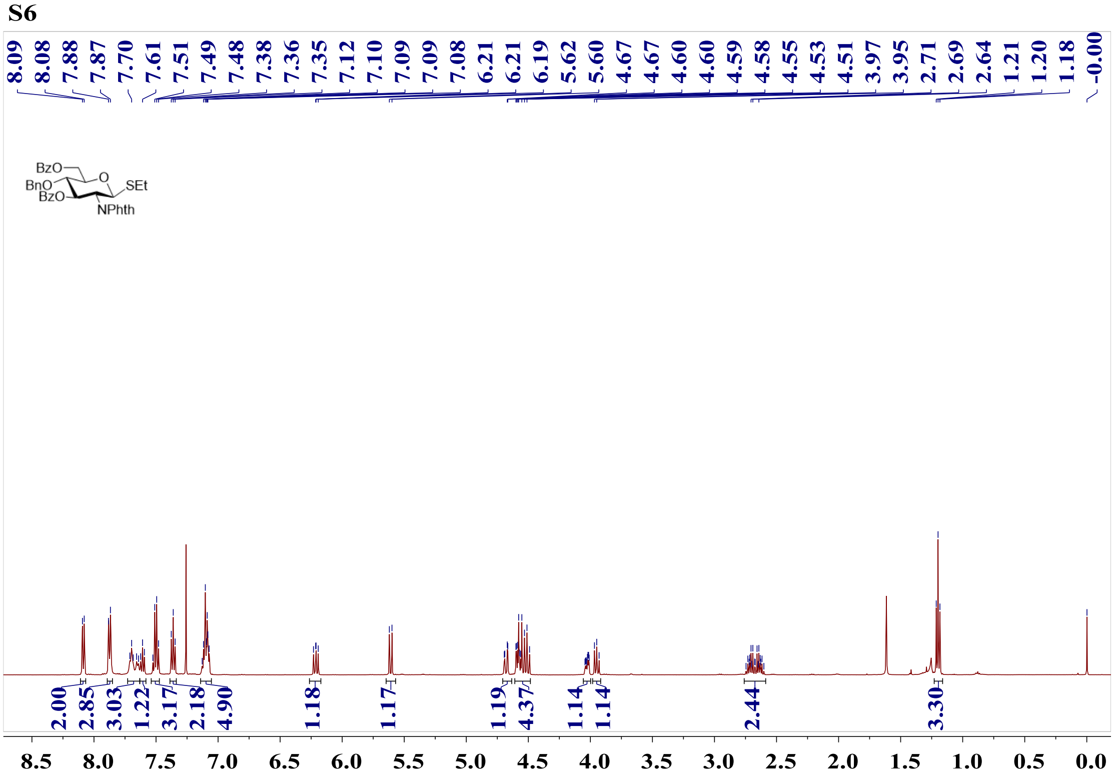

Supplementary Figure 58. ^{13}C NMR spectrum of compound **S6** (125 MHz, $CDCl_3$, 25 °C)

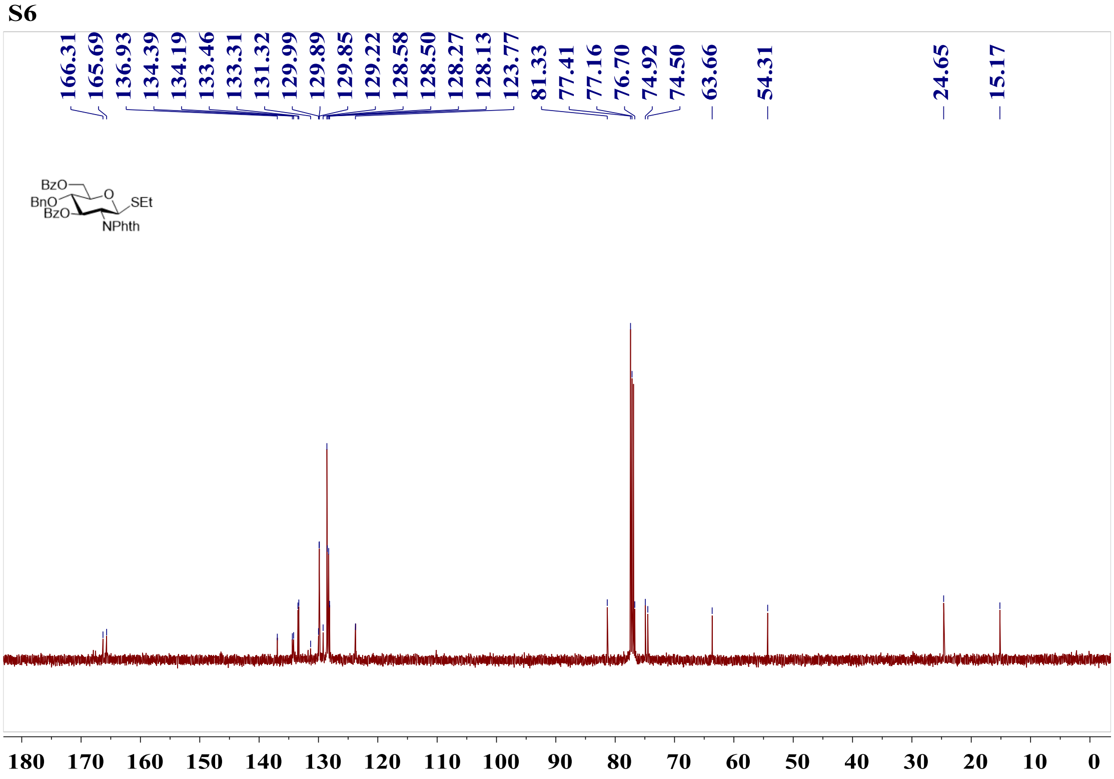

Supplementary Figure 59. ^{1}H NMR spectrum of compound **1h** (500 MHz, $CDCl_3$, 25 °C)

1h

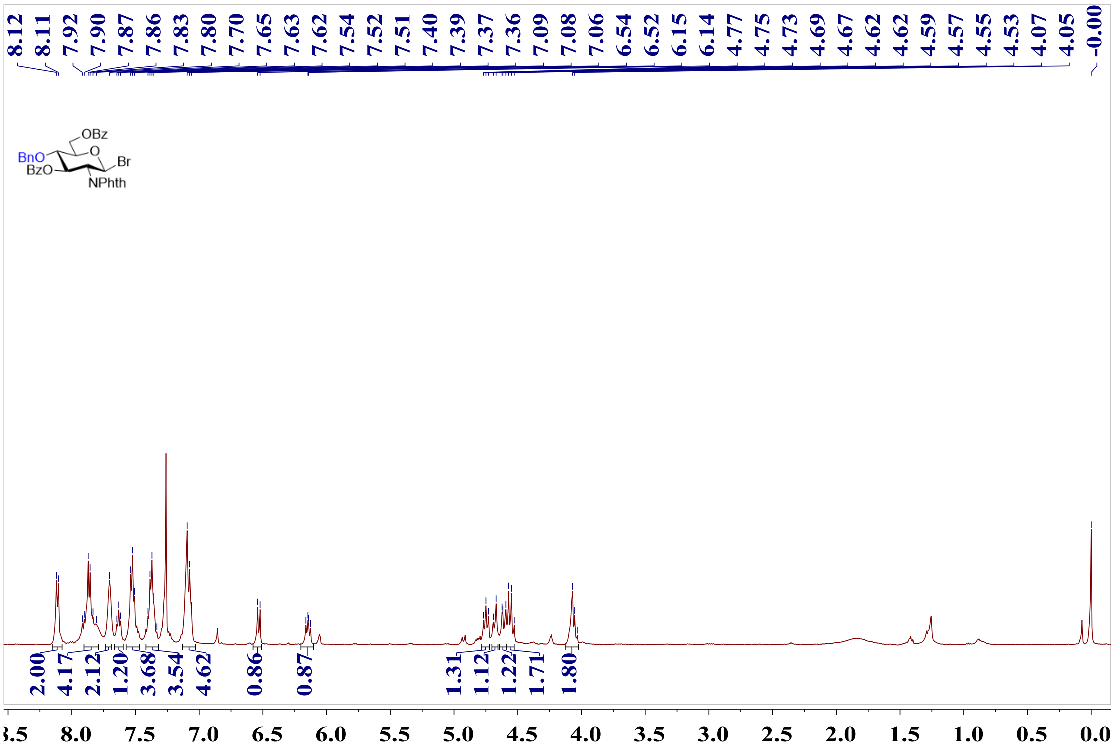

Supplementary Figure 60. ^{13}C NMR spectrum of compound **1h** (125 MHz, $CDCl_3$, 25 °C)

1h

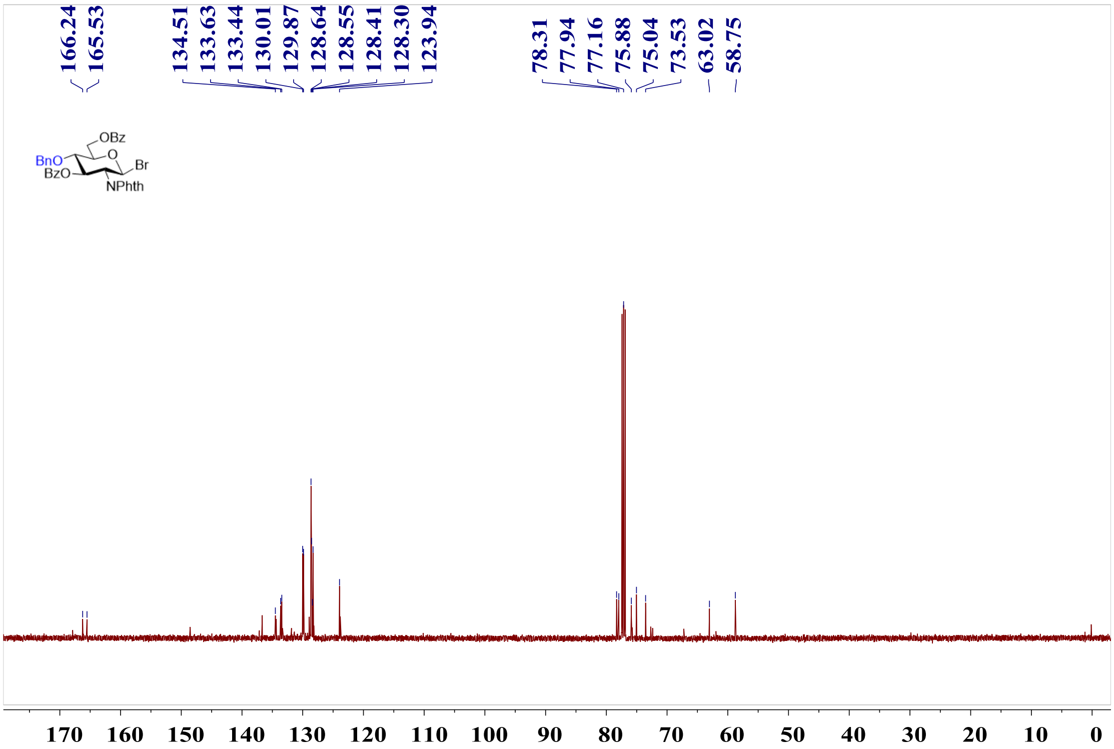

Supplementary Figure 61. ^{1}H NMR spectrum of compound **S9** (500 MHz, $CDCl_3$, 25 ^{o}C)

S9

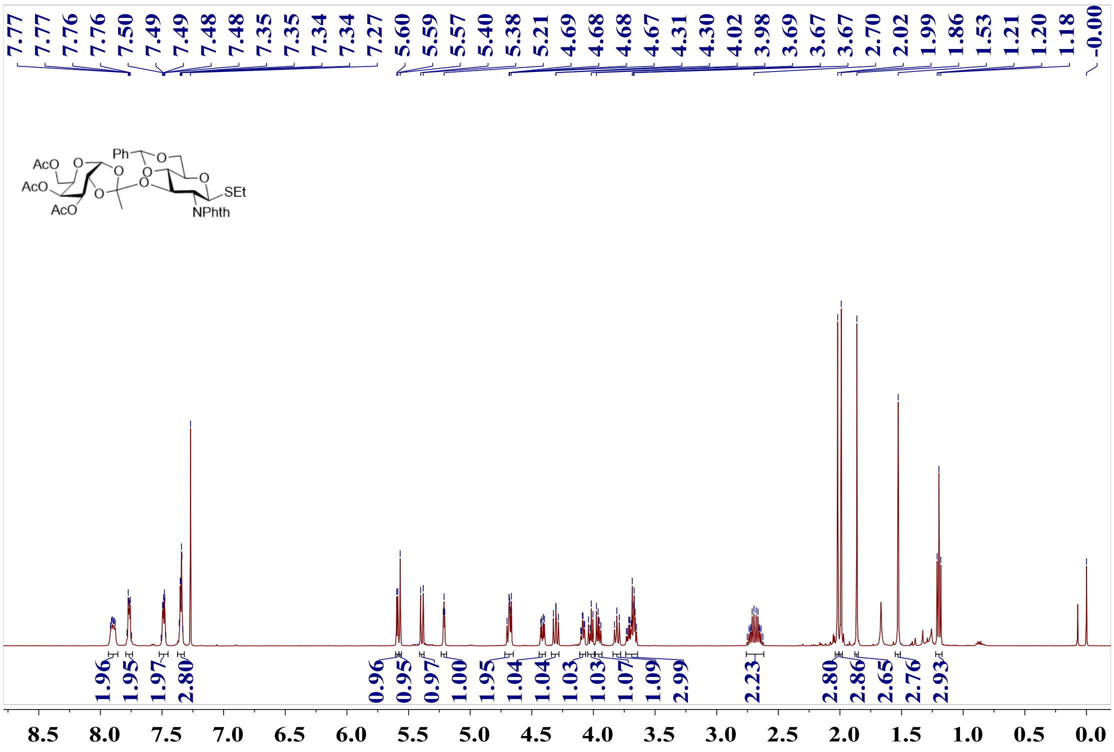

Supplementary Figure 62. ^{13}C NMR spectrum of compound **S9** (125 MHz, $CDCl_3$, 25 ^{o}C)

S9

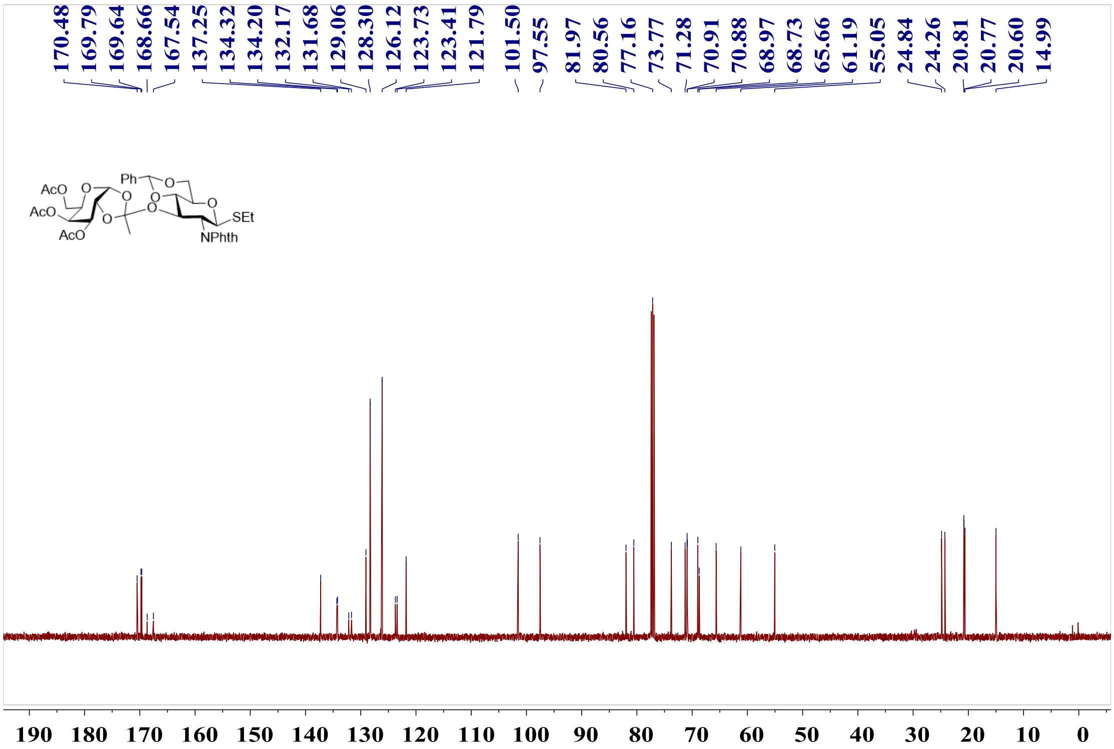

Supplementary Figure 63. ^{1}H NMR spectrum of compound **S11** (500 MHz, $CDCl_3$, 25 ^{o}C)

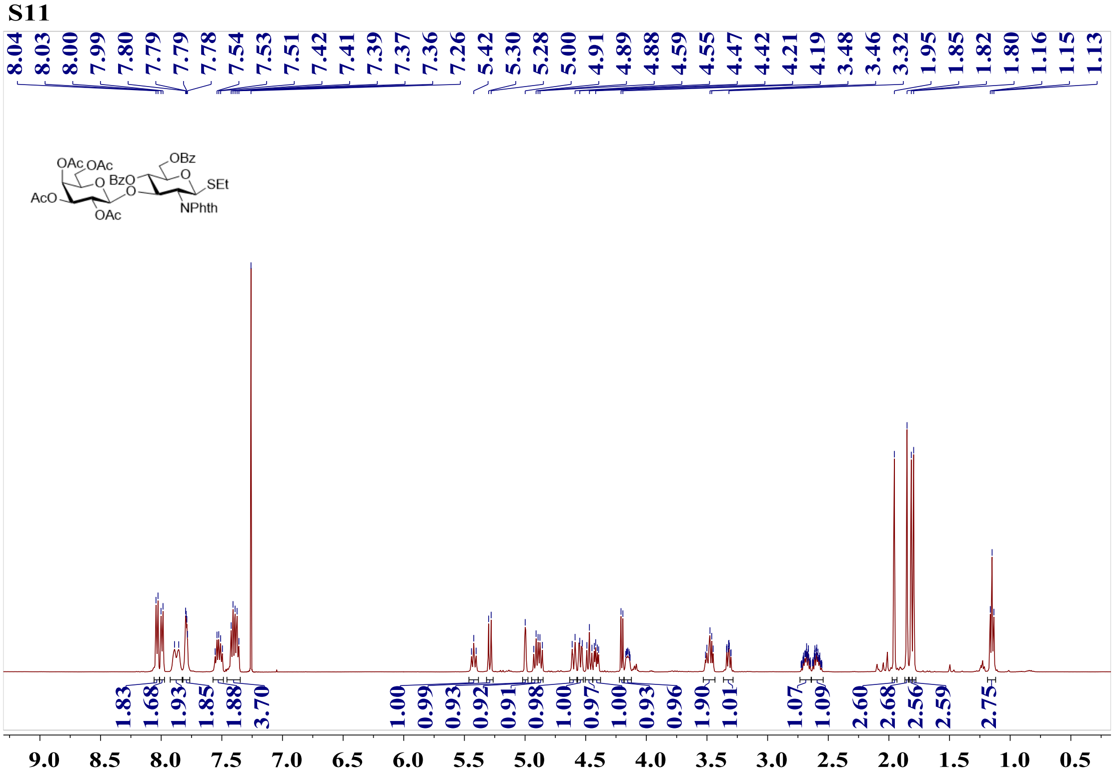

Supplementary Figure 64. ^{13}C NMR spectrum of compound **S11** (125 MHz, $CDCl_3$, 25 ^{o}C)

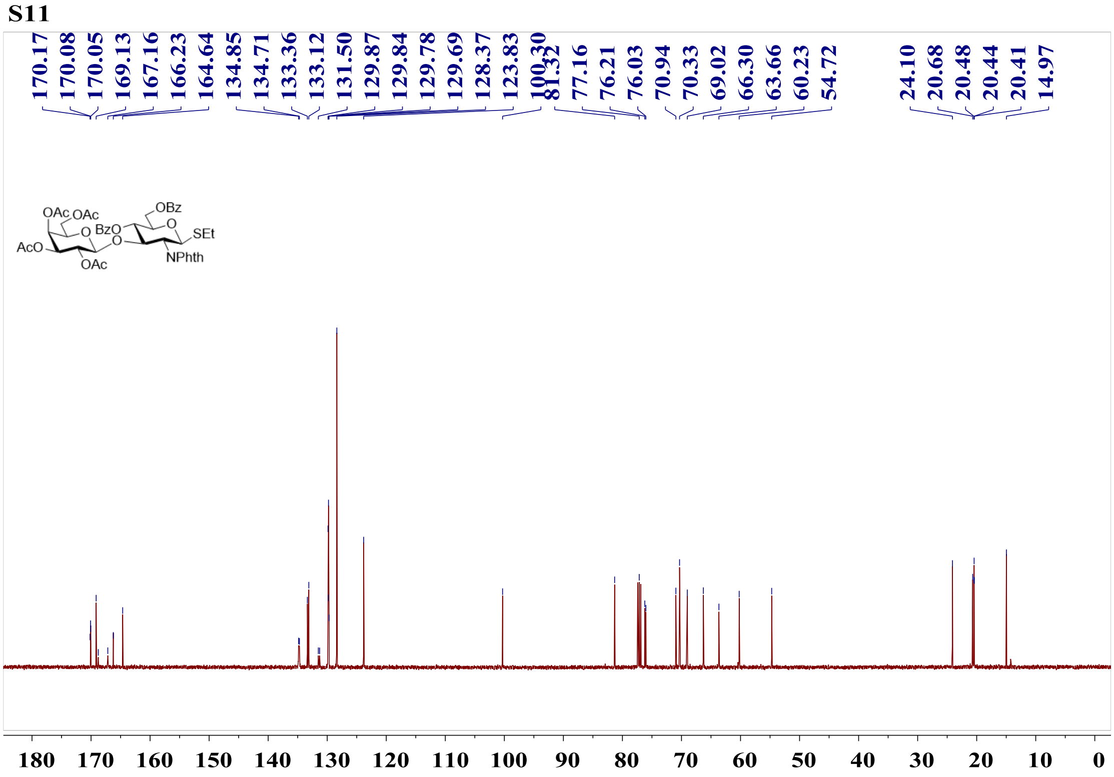

Supplementary Figure 65. ^{1}H NMR spectrum of compound **1j** (500 MHz, $CDCl_3$, 25 ^{o}C)

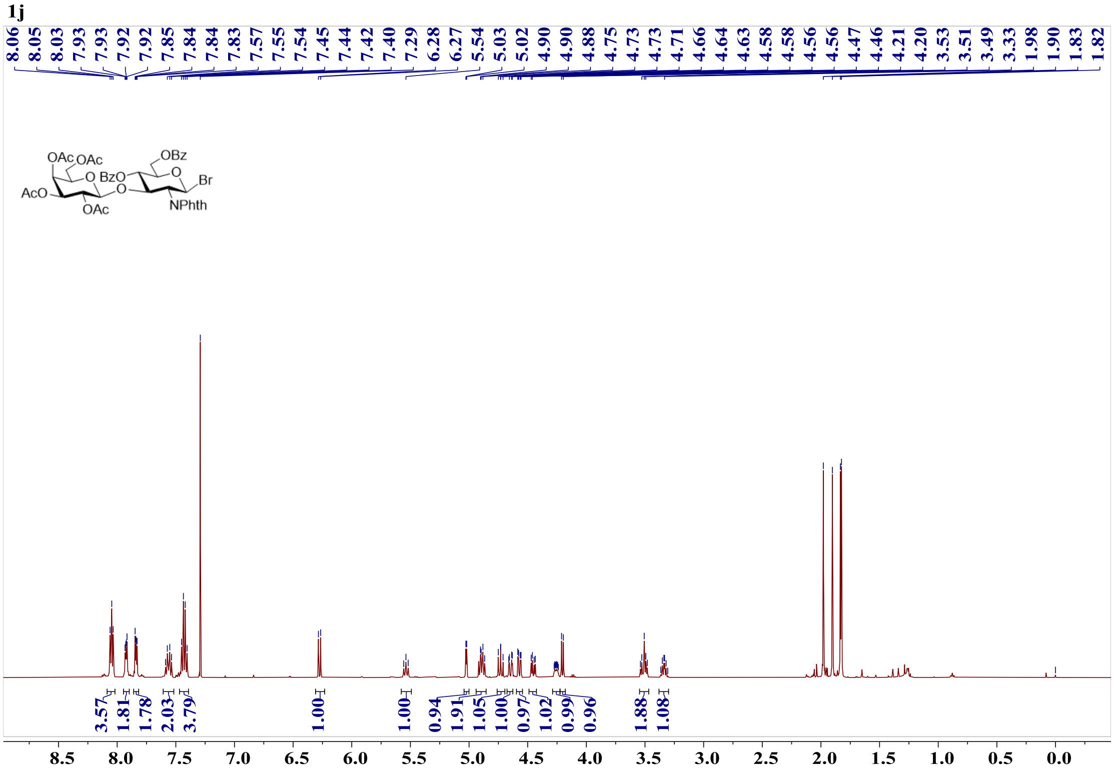

Supplementary Figure 66. ^{13}C NMR spectrum of compound **1j** (125 MHz, $CDCl_3$, 25 ^{o}C)

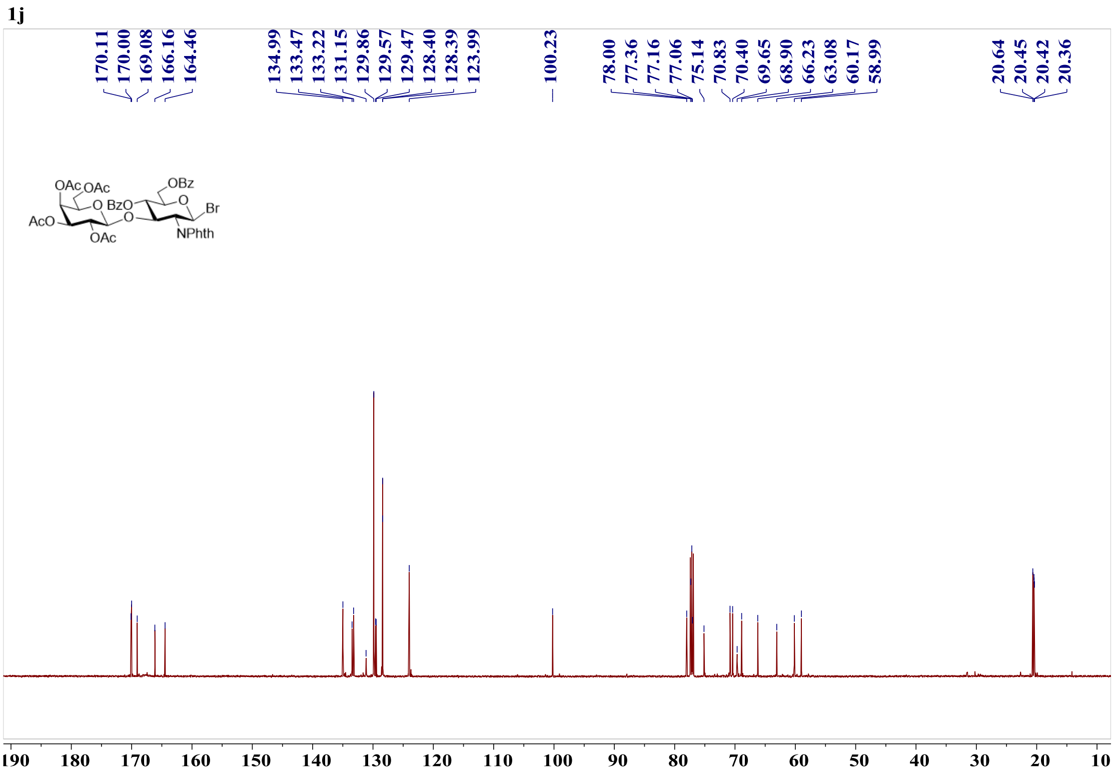

Supplementary Figure 67. ^{1}H NMR spectrum of compound **S14** (500 MHz, $CDCl_3$, 25 °C)

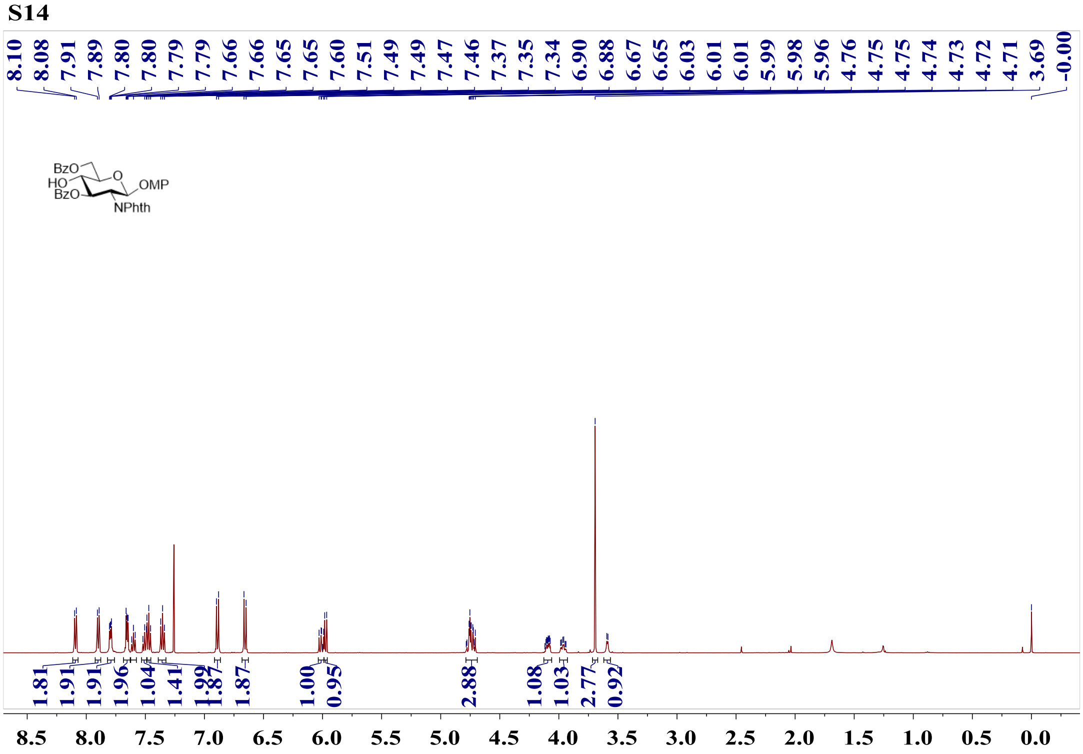

Supplementary Figure 68. ^{13}C NMR spectrum of compound **S14** (125 MHz, $CDCl_3$, 25 °C)

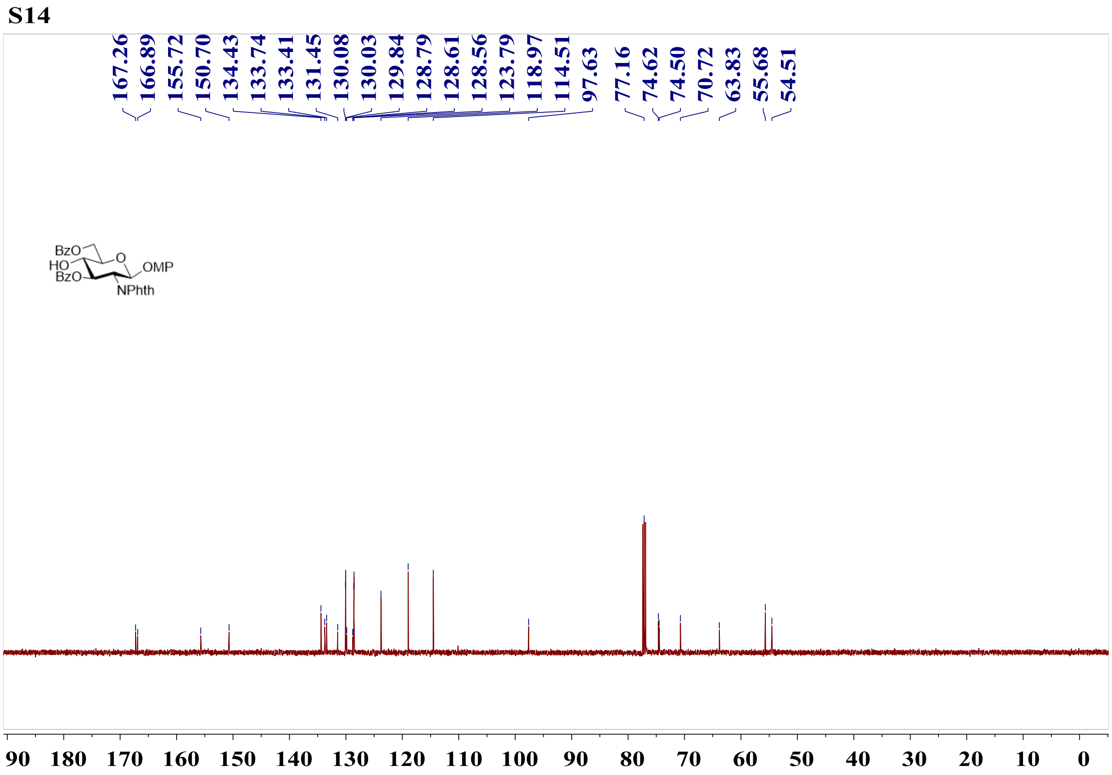

Supplementary Figure 69. ^{1}H NMR spectrum of compound **S16** (500 MHz, $CDCl_3$, 25 °C)

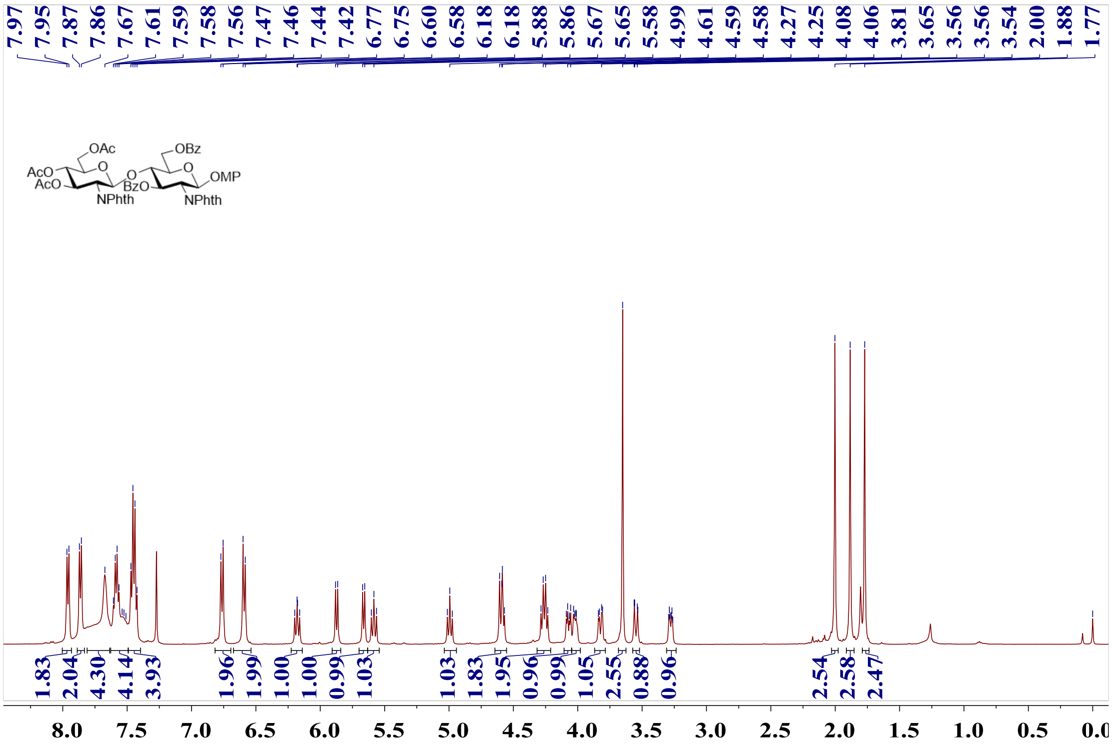

Supplementary Figure 70. ^{13}C NMR spectrum of compound **S16** (125 MHz, $CDCl_3$, 25 °C)

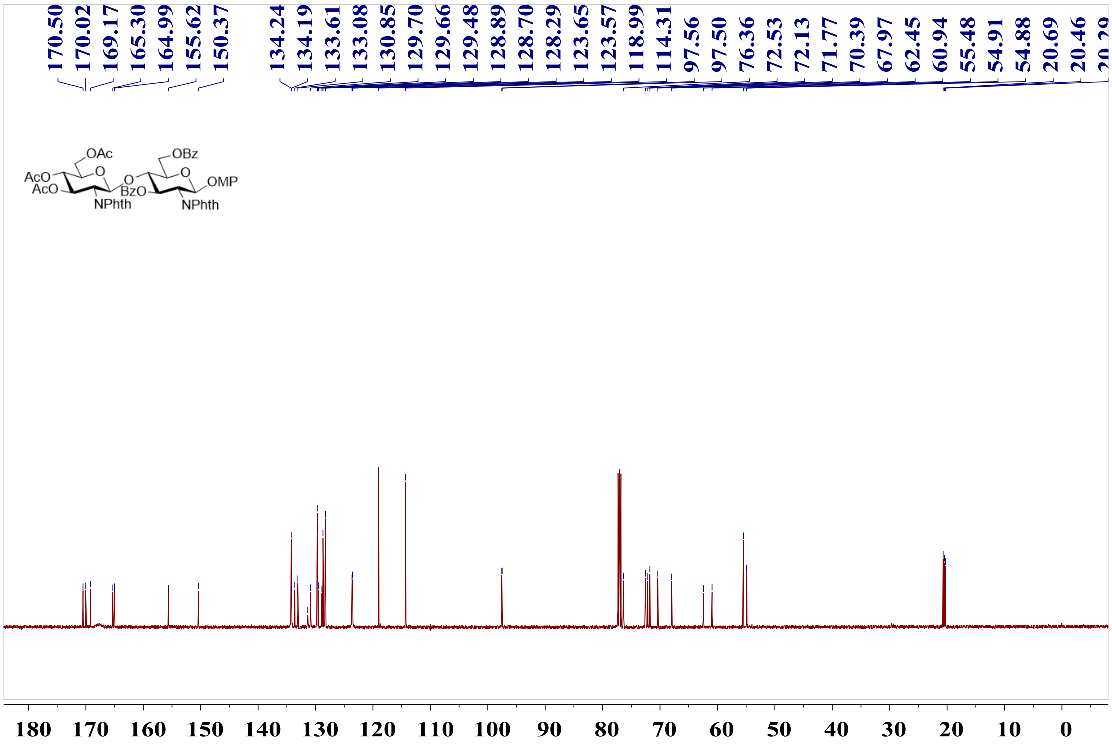

Supplementary Figure 71. ^{1}H NMR spectrum of compound **S17** (500 MHz, $CDCl_3$, 25 °C)

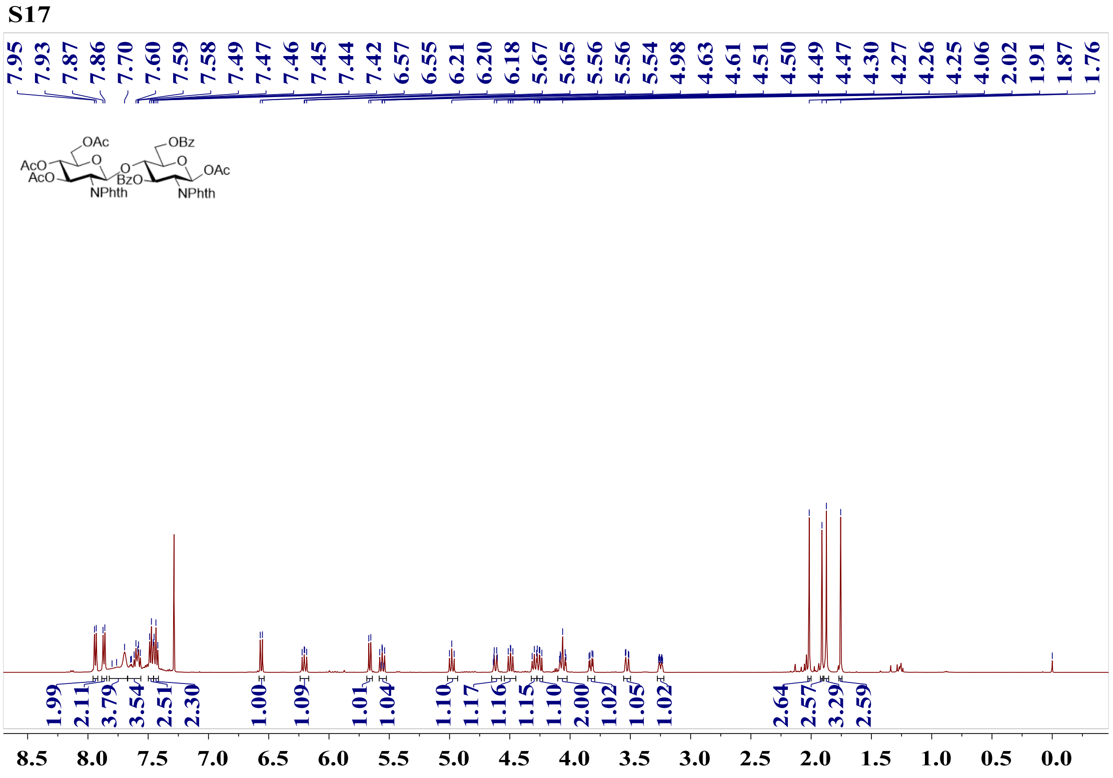

Supplementary Figure 72. ^{13}C NMR spectrum of compound **S17** (125 MHz, $CDCl_3$, 25 °C)

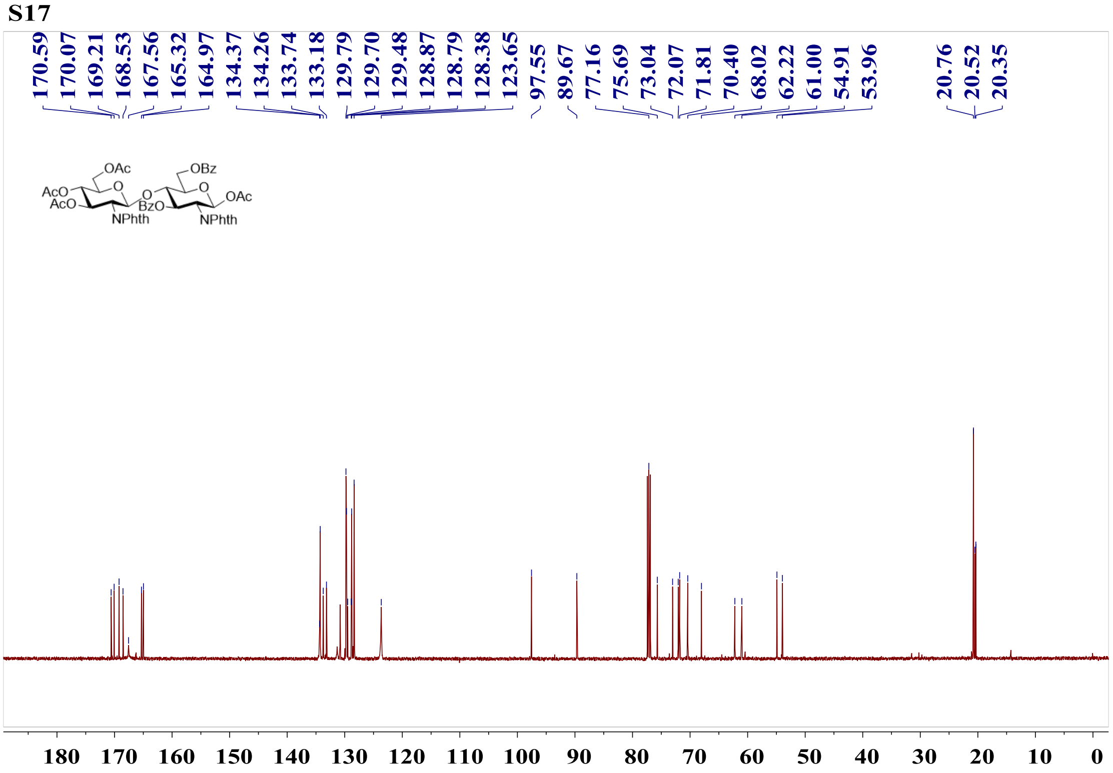

Supplementary Figure 73. ^{1}H NMR spectrum of compound **1k** (500 MHz, $CDCl_3$, 25 °C)

1k

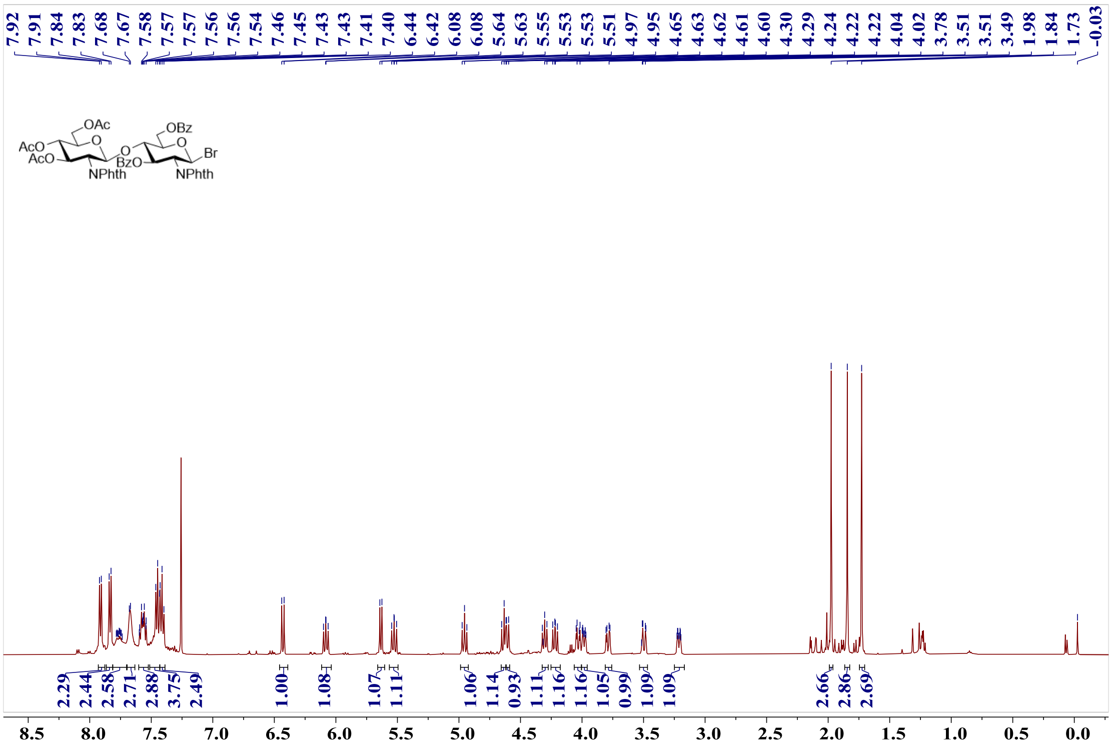

Supplementary Figure 74. ^{13}C NMR spectrum of compound **1k** (125 MHz, $CDCl_3$, 25 °C)

1k

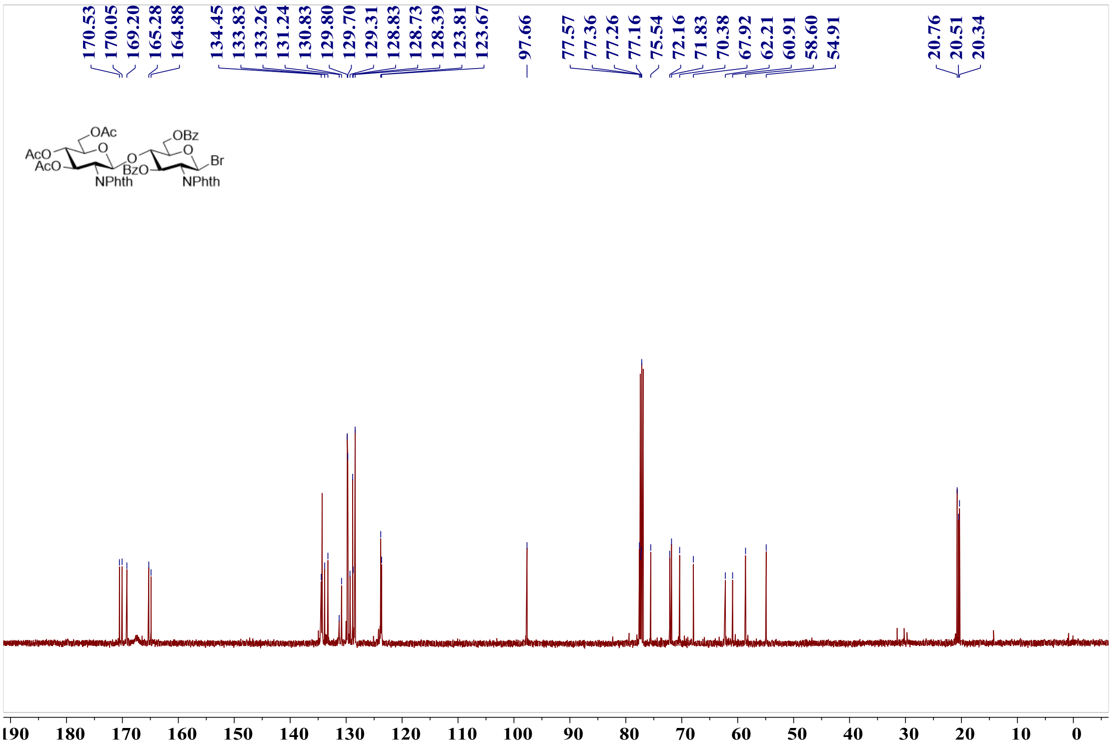

Supplementary Figure 75. ^{1}H NMR spectrum of compound **S19** (500 MHz, $CDCl_3$, 25 °C)

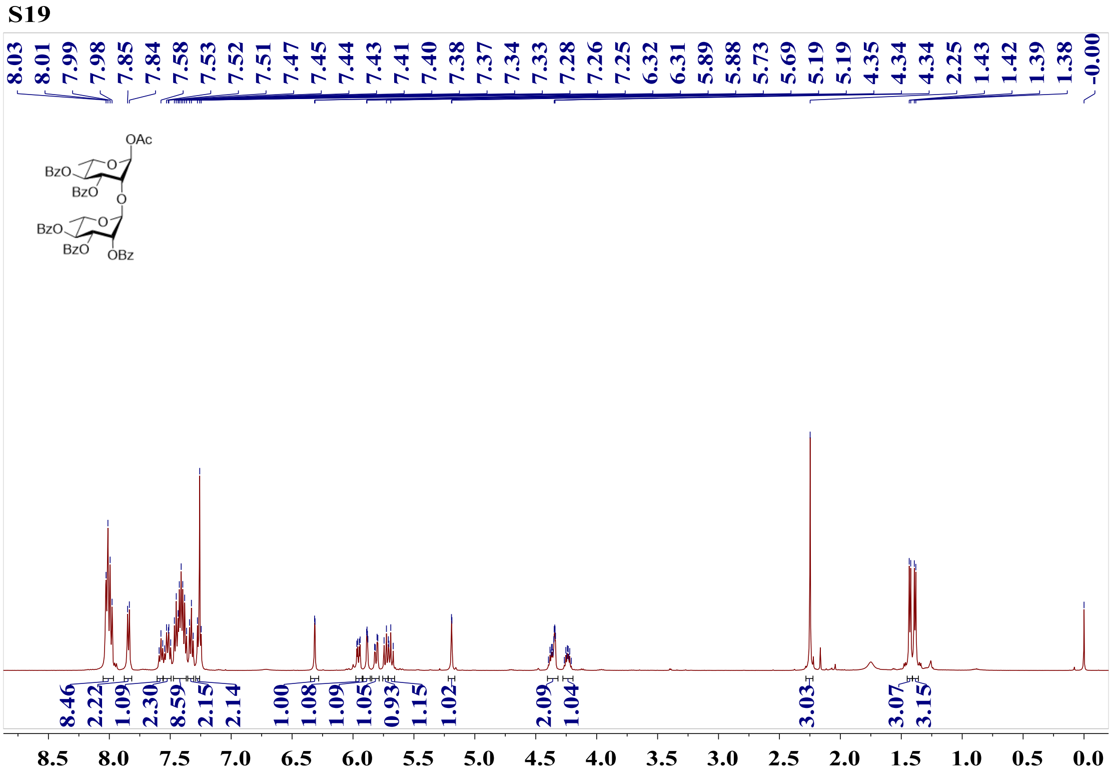

Supplementary Figure 76. ^{13}C NMR spectrum of compound **S19** (125 MHz, $CDCl_3$, 25 °C)

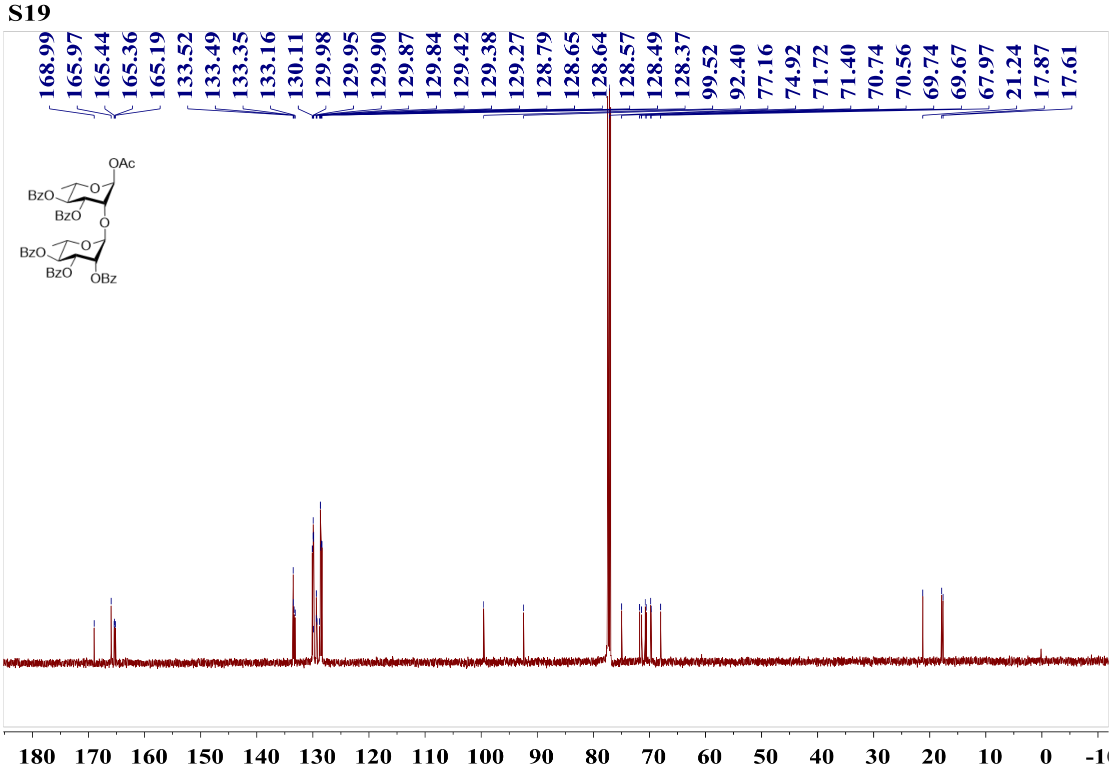

Supplementary Figure 77. ^{1}H NMR spectrum of compound **1l** (500 MHz, $CDCl_3$, 25 ^{o}C)

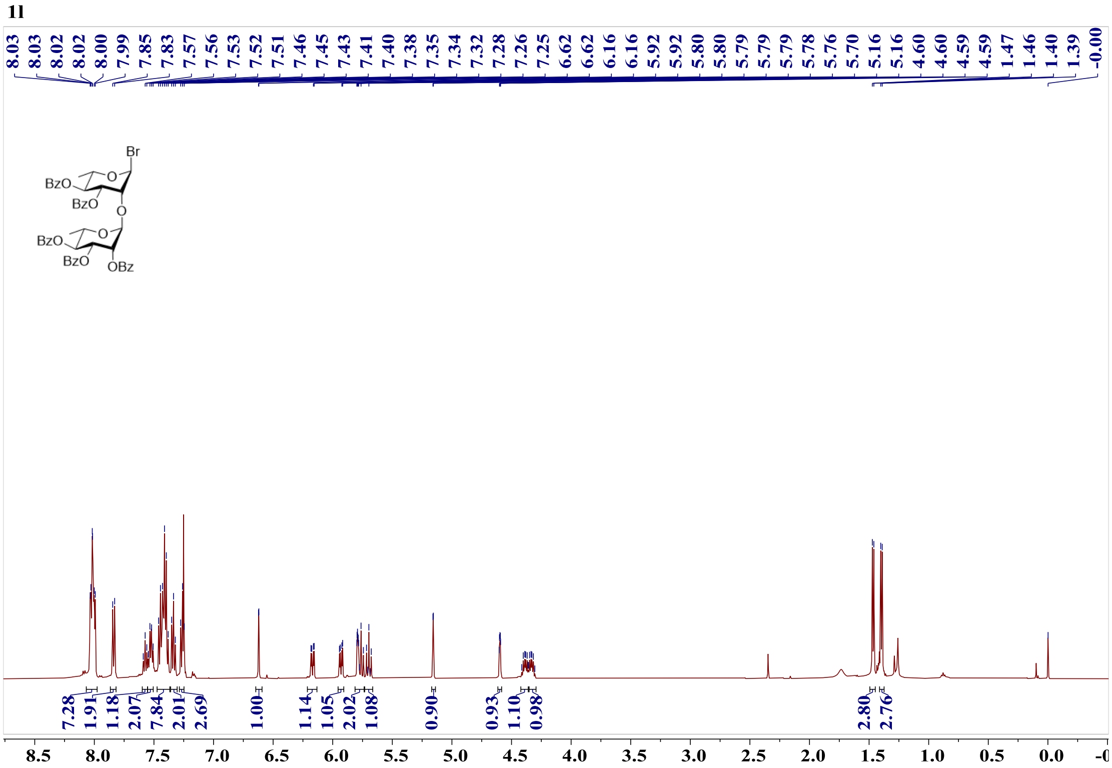

Supplementary Figure 78. ^{13}C NMR spectrum of compound **1l** (125 MHz, $CDCl_3$, 25 ^{o}C)

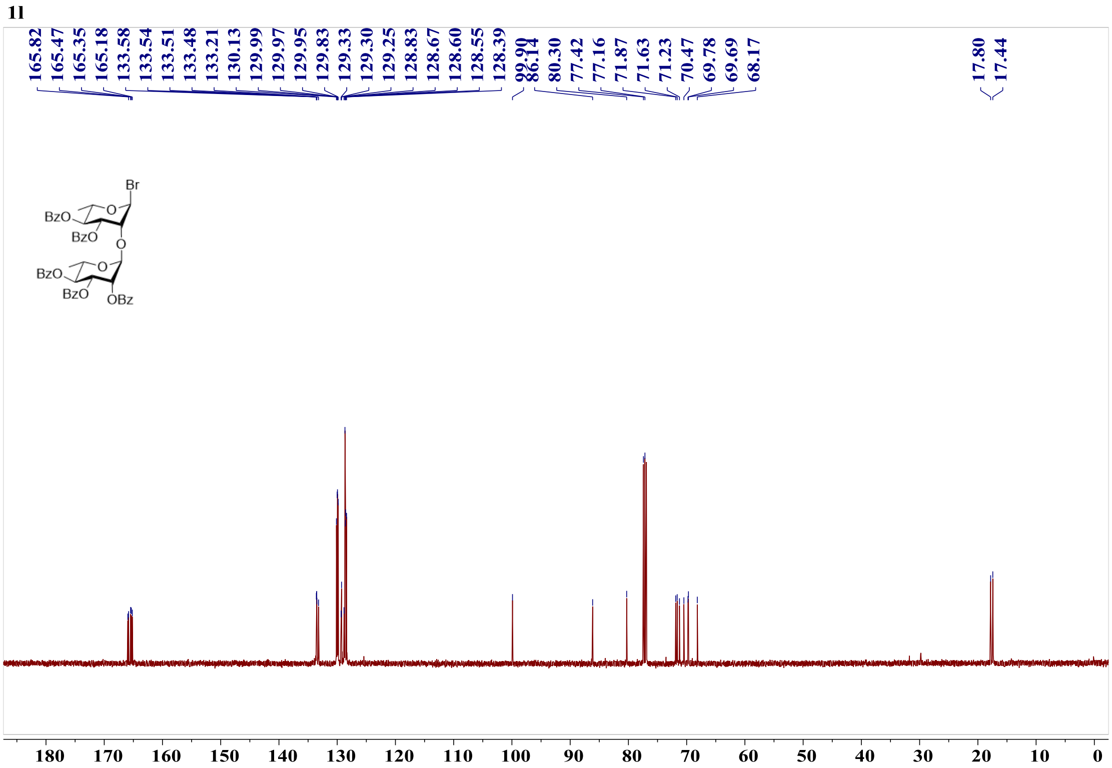

Supplementary Figure 79. ^{1}H NMR spectrum of compound **S23** (500 MHz, $CDCl_3$, 25 ^{o}C)

S23

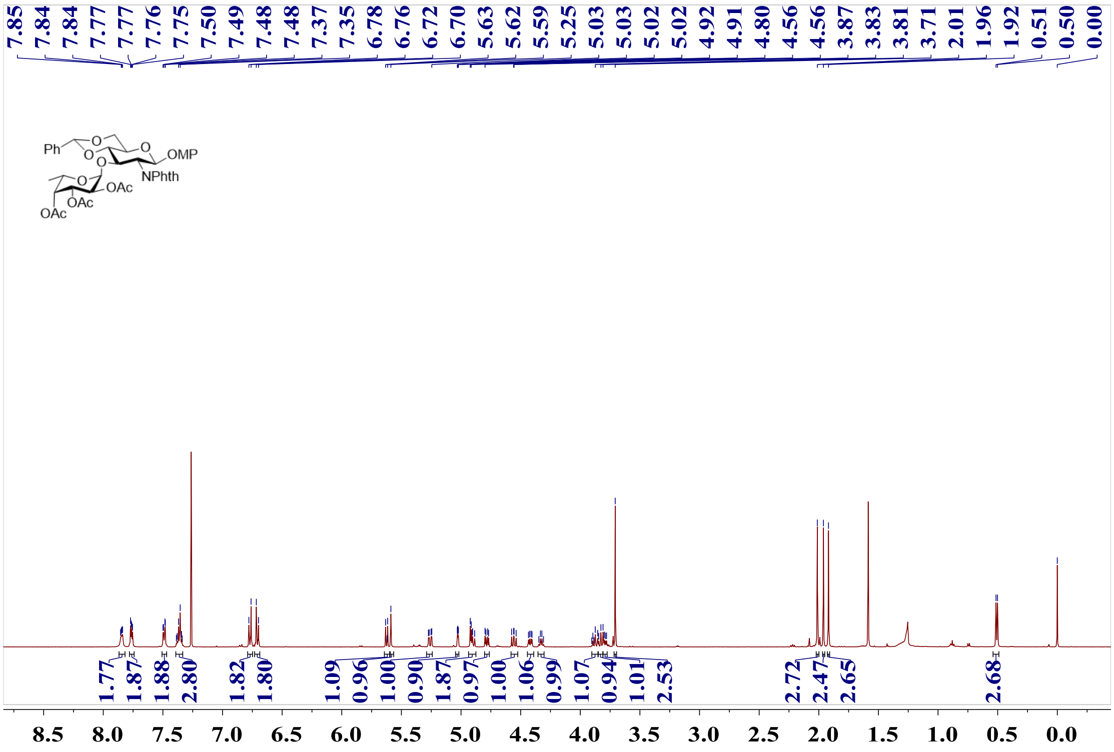

Supplementary Figure 80. ^{13}C NMR spectrum of compound **S23** (125 MHz, $CDCl_3$, 25 ^{o}C)

S23

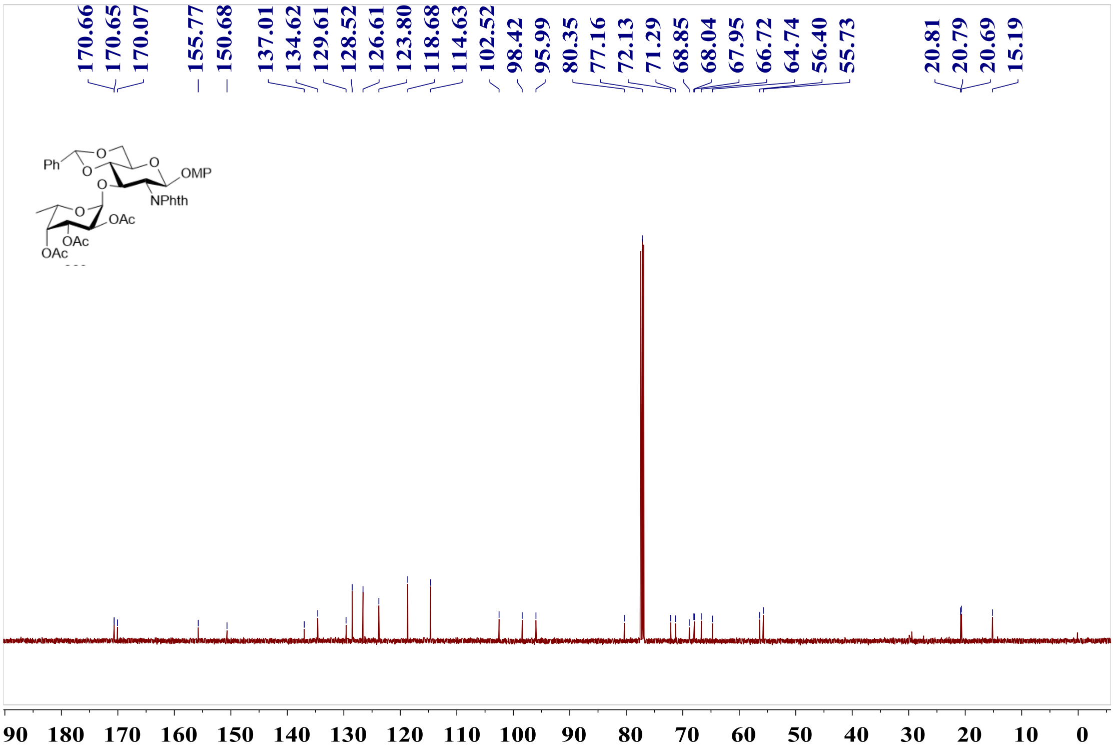

Supplementary Figure 81. ^{1}H NMR spectrum of compound **S24** (500 MHz, $CDCl_3$, 25 °C)

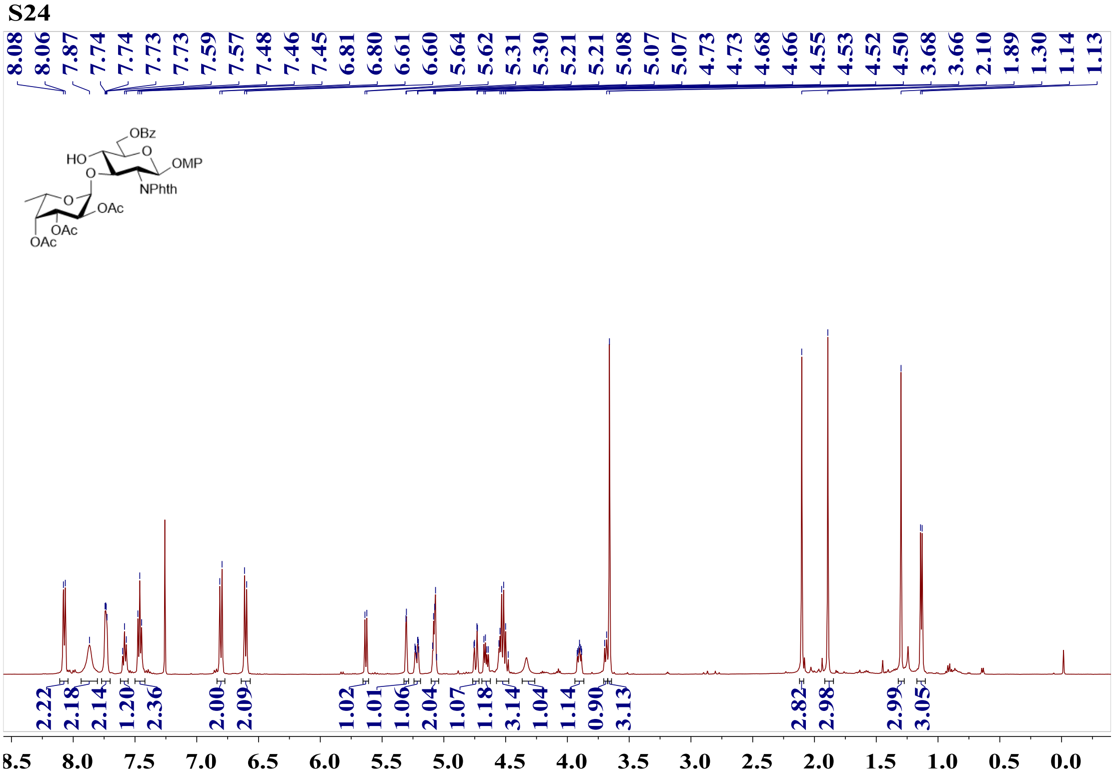

Supplementary Figure 82. ^{13}C NMR spectrum of compound **S24** (125 MHz, $CDCl_3$, 25 °C)

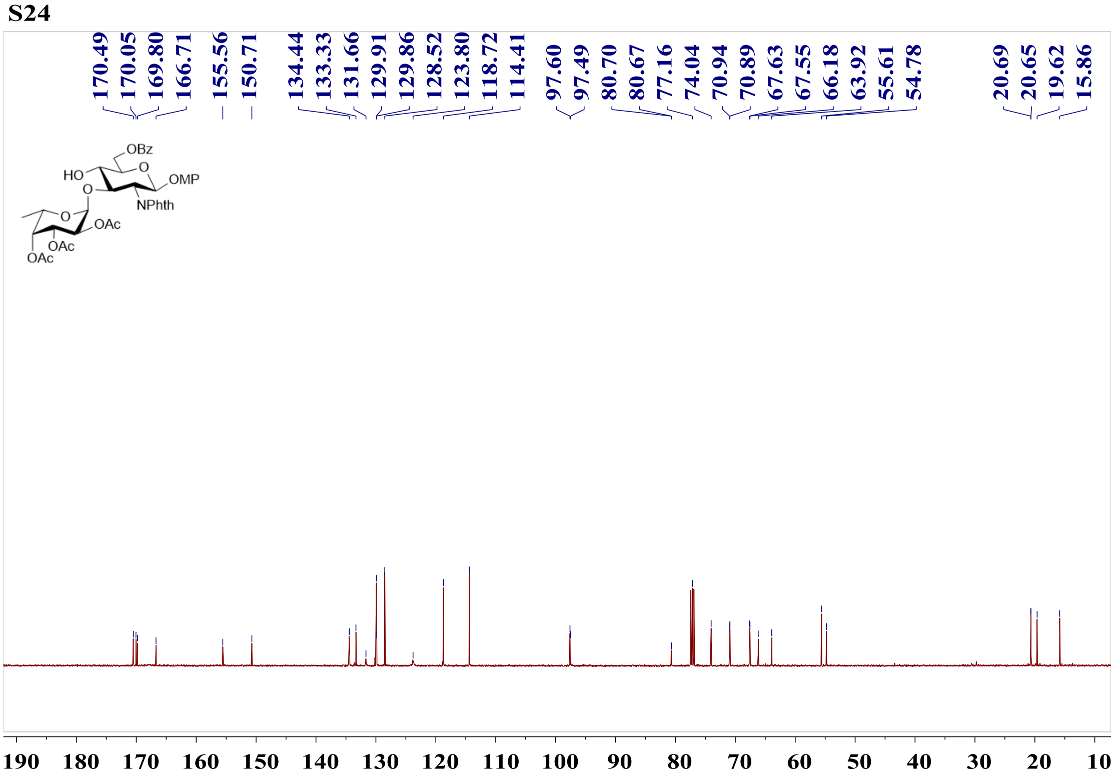

Supplementary Figure 83. ^{1}H NMR spectrum of compound **S25** (500 MHz, $CDCl_3$, 25 ^{o}C)

S25

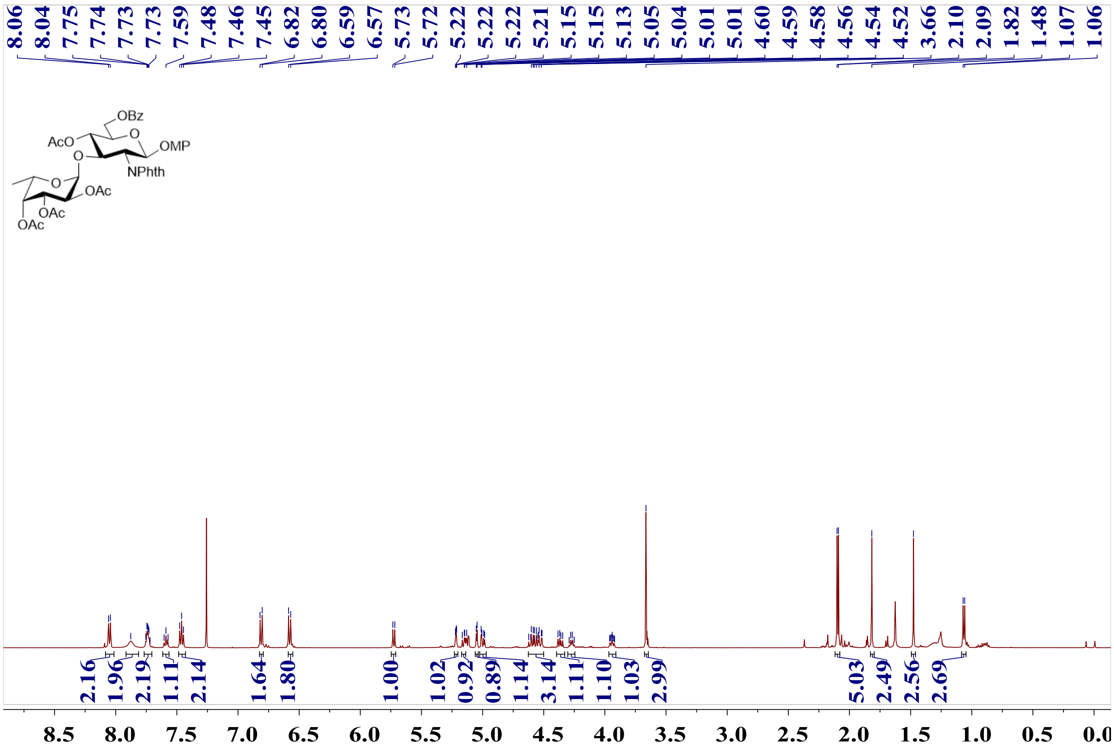

Supplementary Figure 84. ^{13}C NMR spectrum of compound **S25** (125 MHz, $CDCl_3$, 25 ^{o}C)

S25

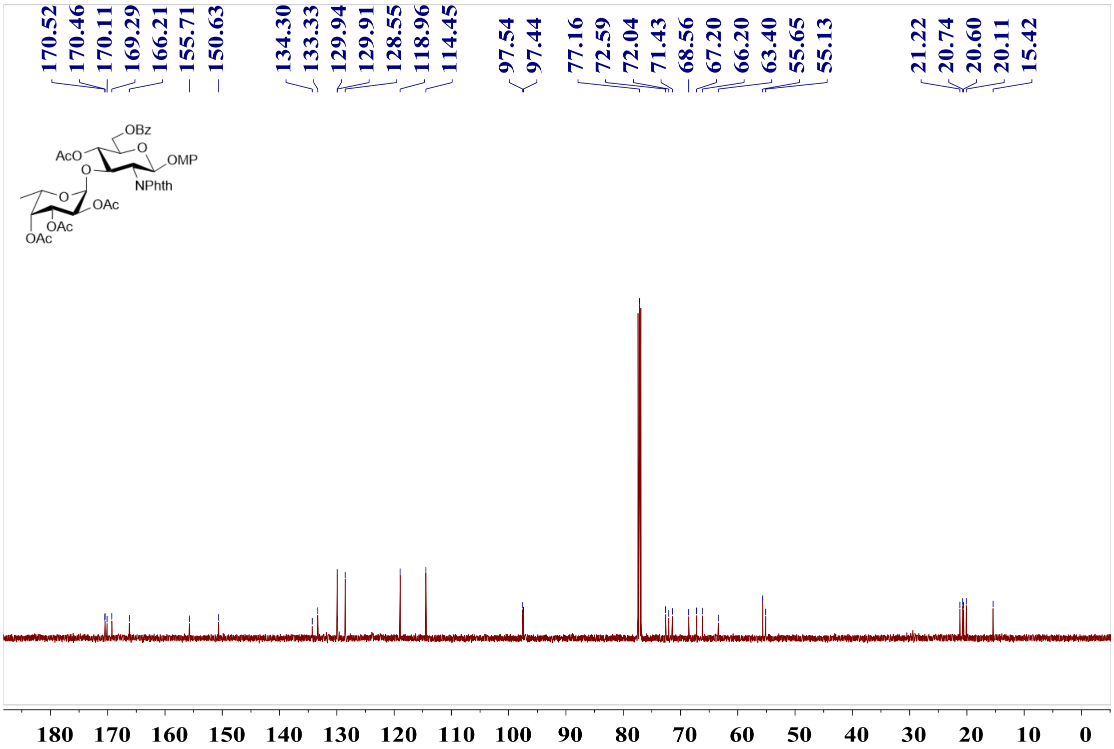

Supplementary Figure 85. ^{1}H NMR spectrum of compound **S26** (500 MHz, $CDCl_3$, 25 °C)

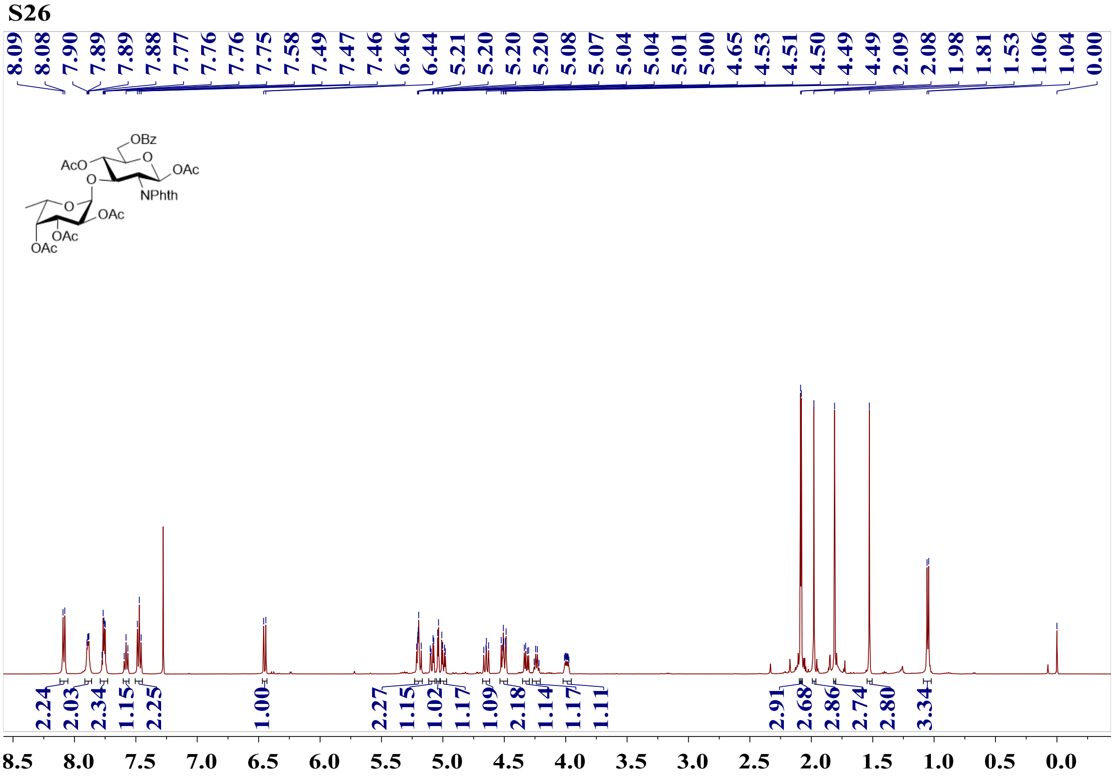

Supplementary Figure 86. ^{13}C NMR spectrum of compound **S26** (125 MHz, $CDCl_3$, 25 °C)

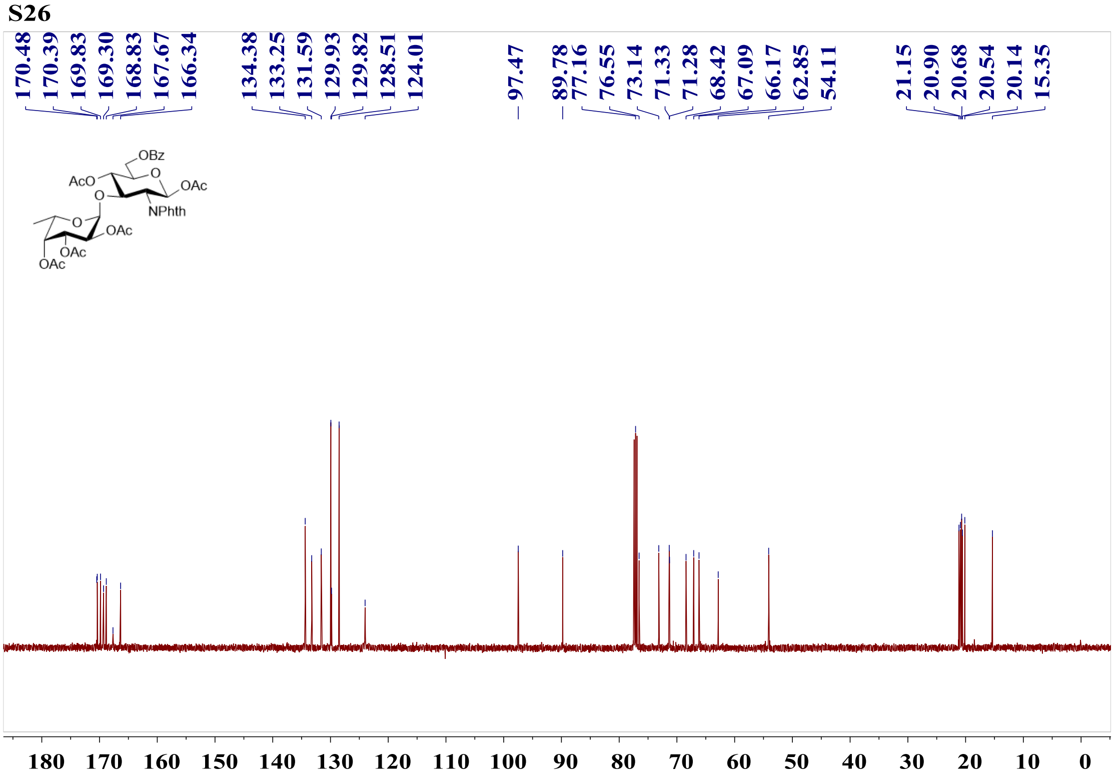

Supplementary Figure 87. ^{1}H NMR spectrum of compound **1m** (500 MHz, $CDCl_3$, 25 °C)

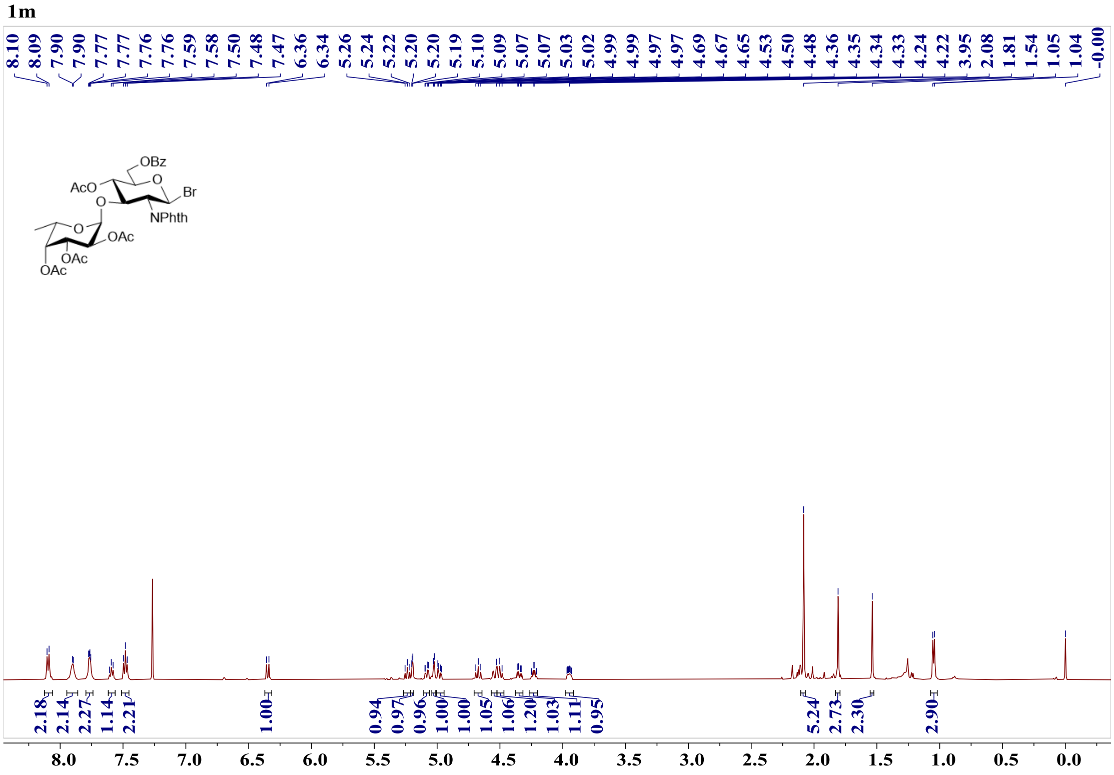

Supplementary Figure 88. ^{13}C NMR spectrum of compound **1m** (125 MHz, $CDCl_3$, 25 °C)

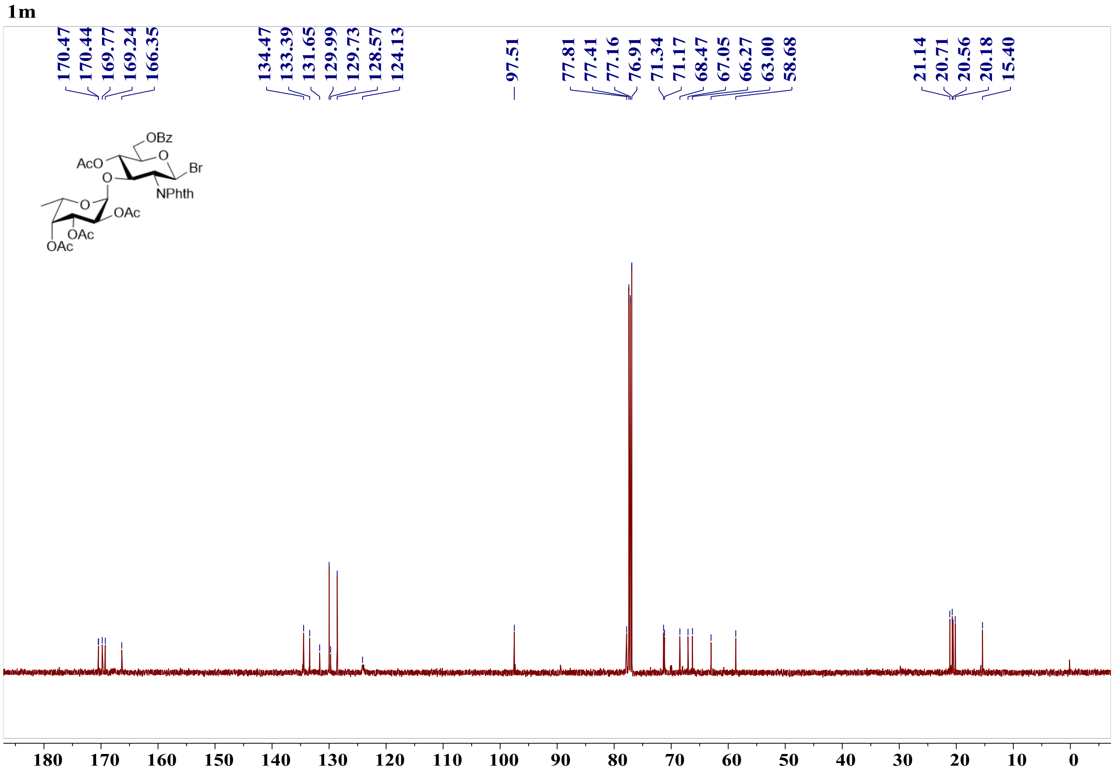

Supplementary Figure 89. ^{1}H NMR spectrum of compound **S27** (500 MHz, $CDCl_3$, 25 ^{o}C)

S27

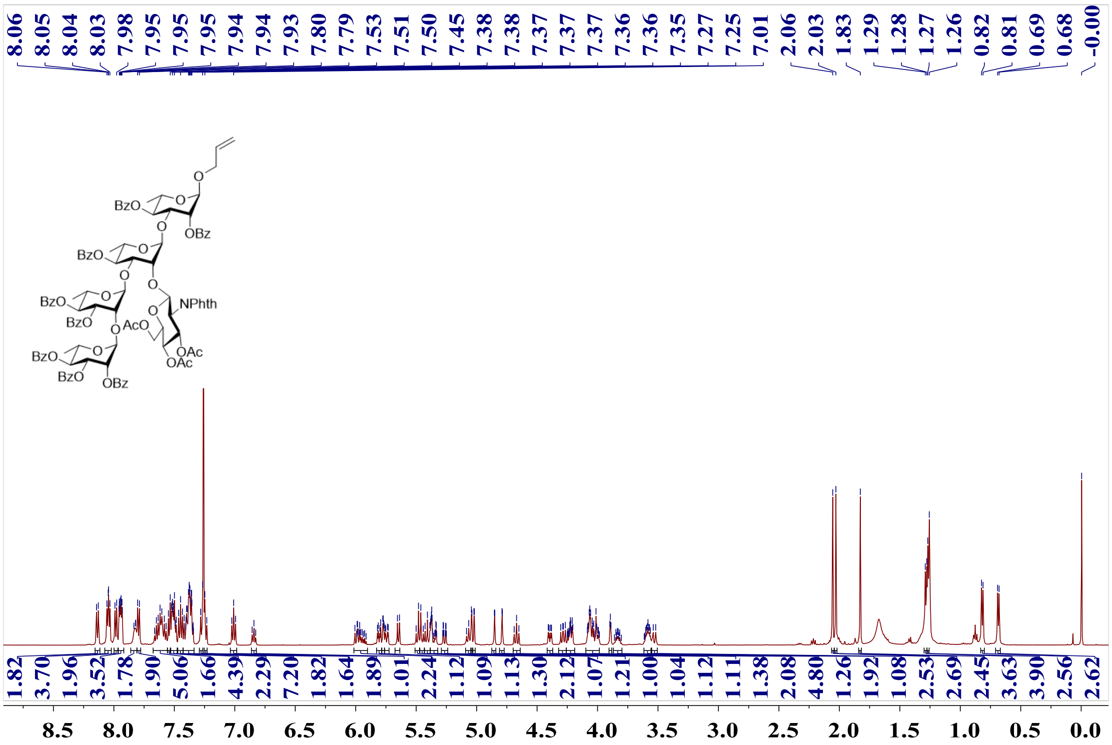

Supplementary Figure 90. ^{1}H NMR spectrum of compound **S28** (500 MHz, $CDCl_3$, 25 °C)

S28

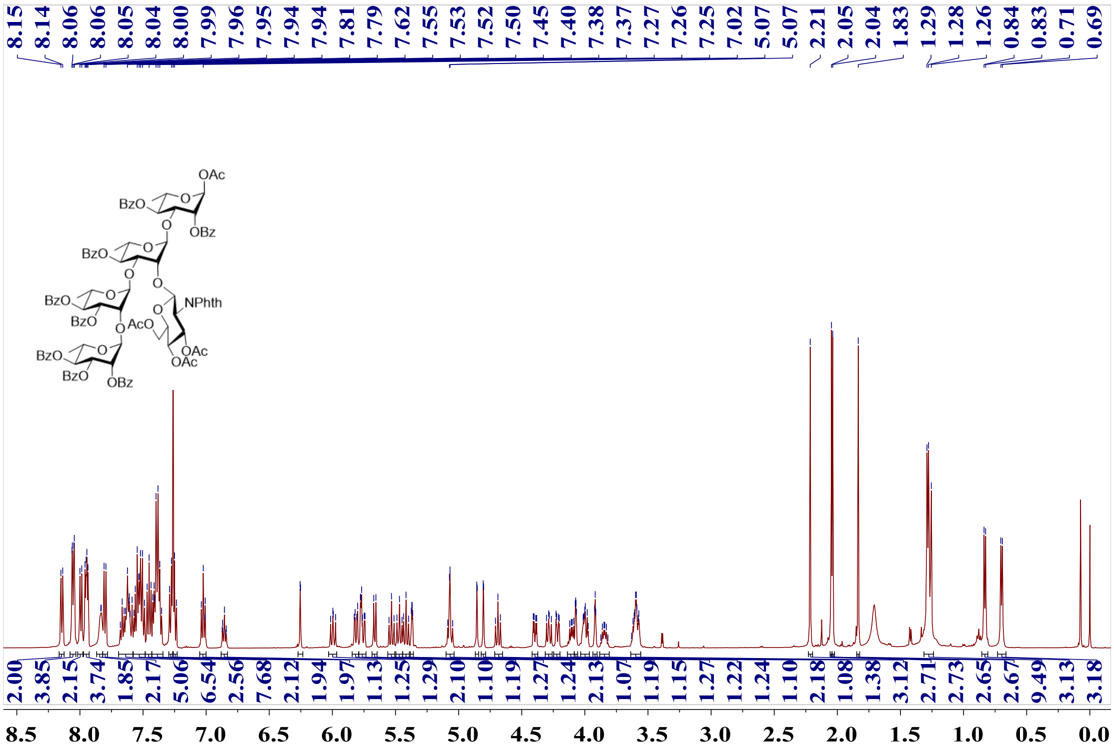

Supplementary Figure 91. ^{13}C NMR spectrum of compound **S28** (125 MHz, $CDCl_3$, 25 °C)

S28

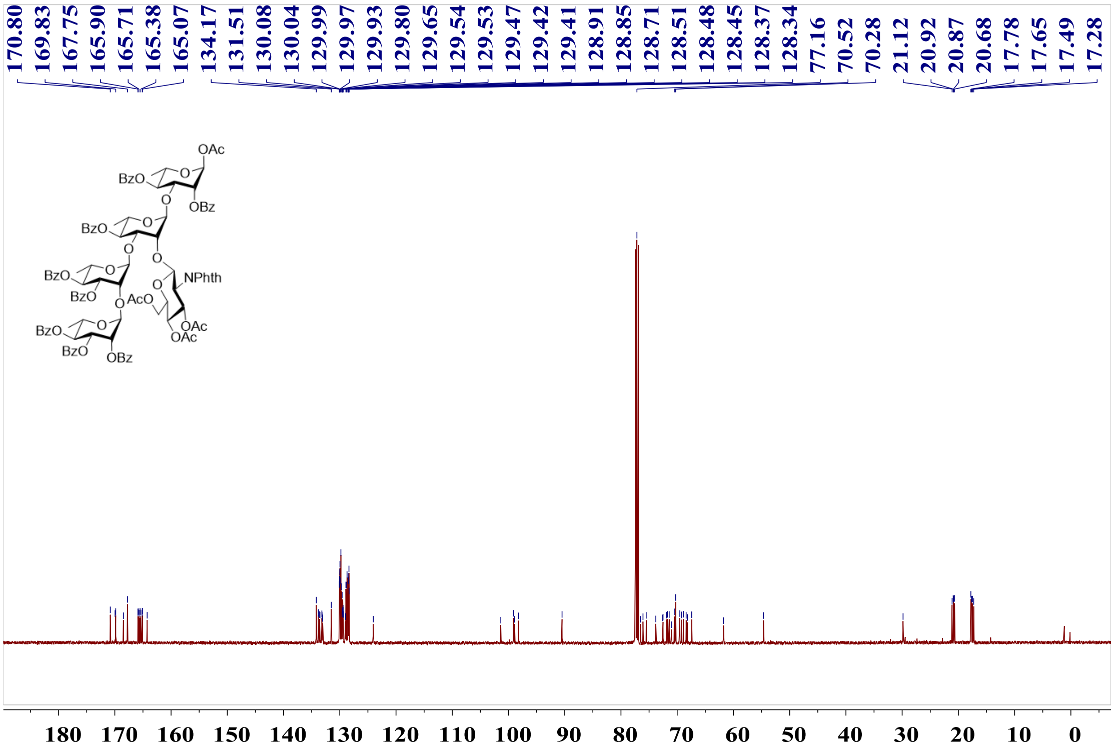

Supplementary Figure 92. ^{1}H NMR spectrum of compound **S31** (500 MHz, $CDCl_3$, 25 °C)

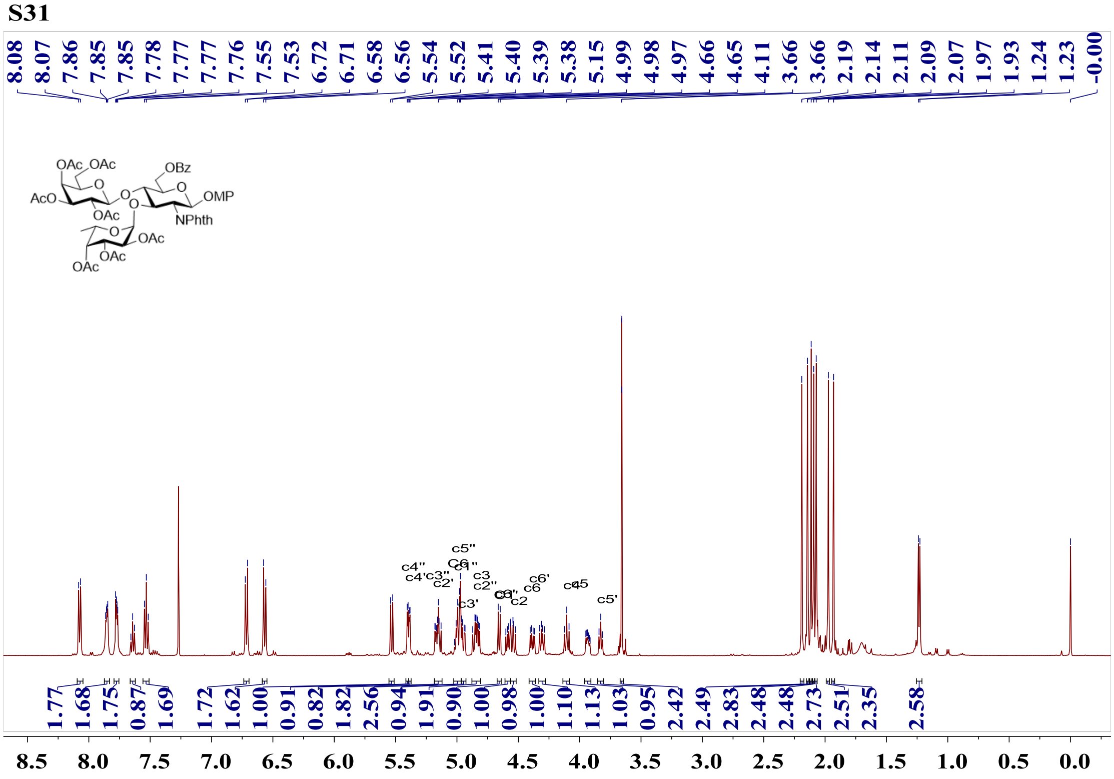

Supplementary Figure 93. ^{13}C NMR spectrum of compound **S31** (125 MHz, $CDCl_3$, 25 °C)

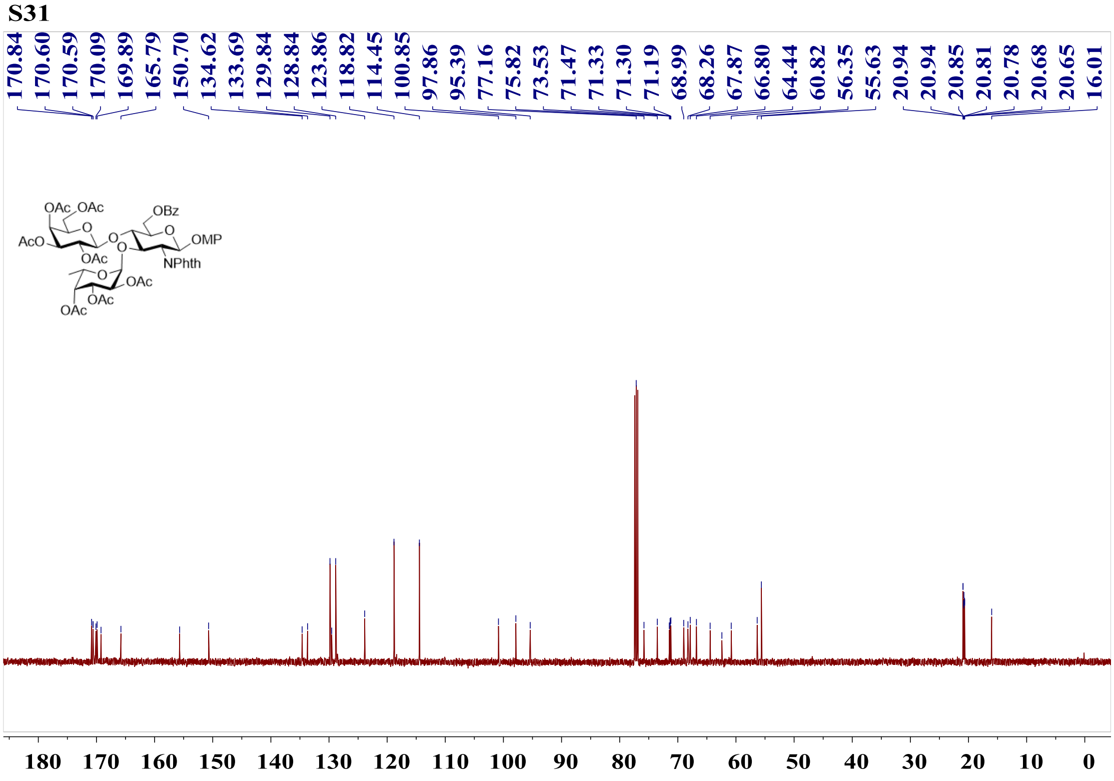

Supplementary Figure 94. ^{1}H NMR spectrum of compound **S32** (500 MHz, $CDCl_3$, 25 ^{o}C)

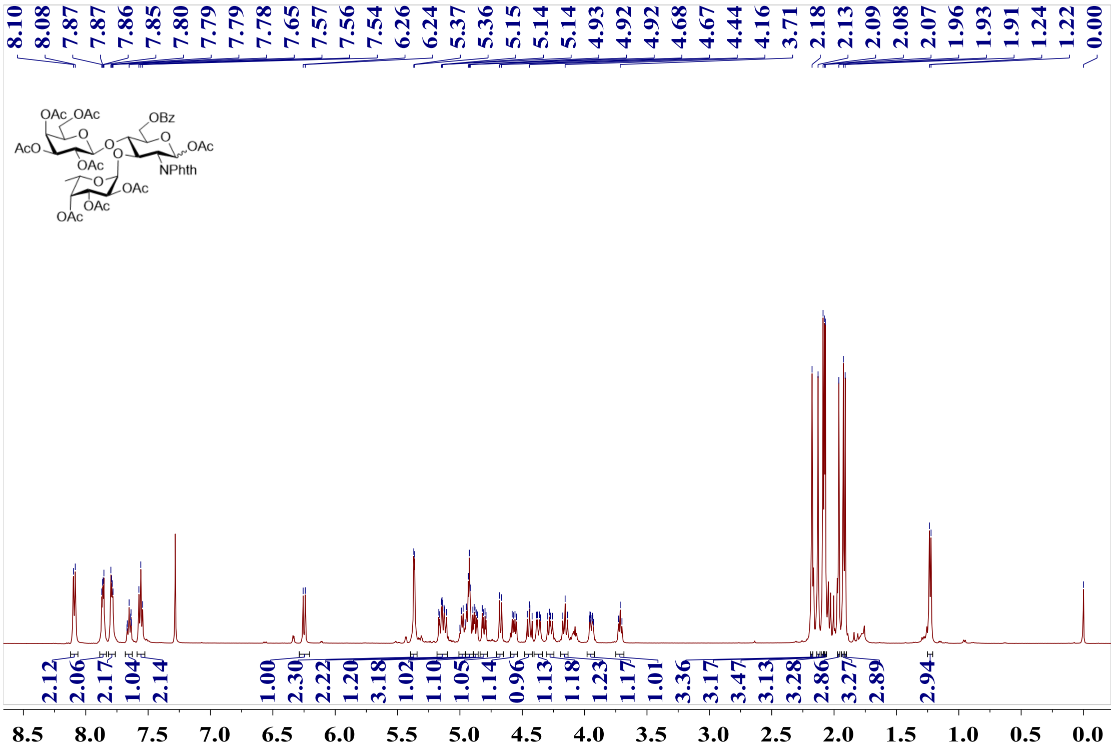

Supplementary Figure 95. ^{13}C NMR spectrum of compound **S32** (125 MHz, $CDCl_3$, 25 ^{o}C)

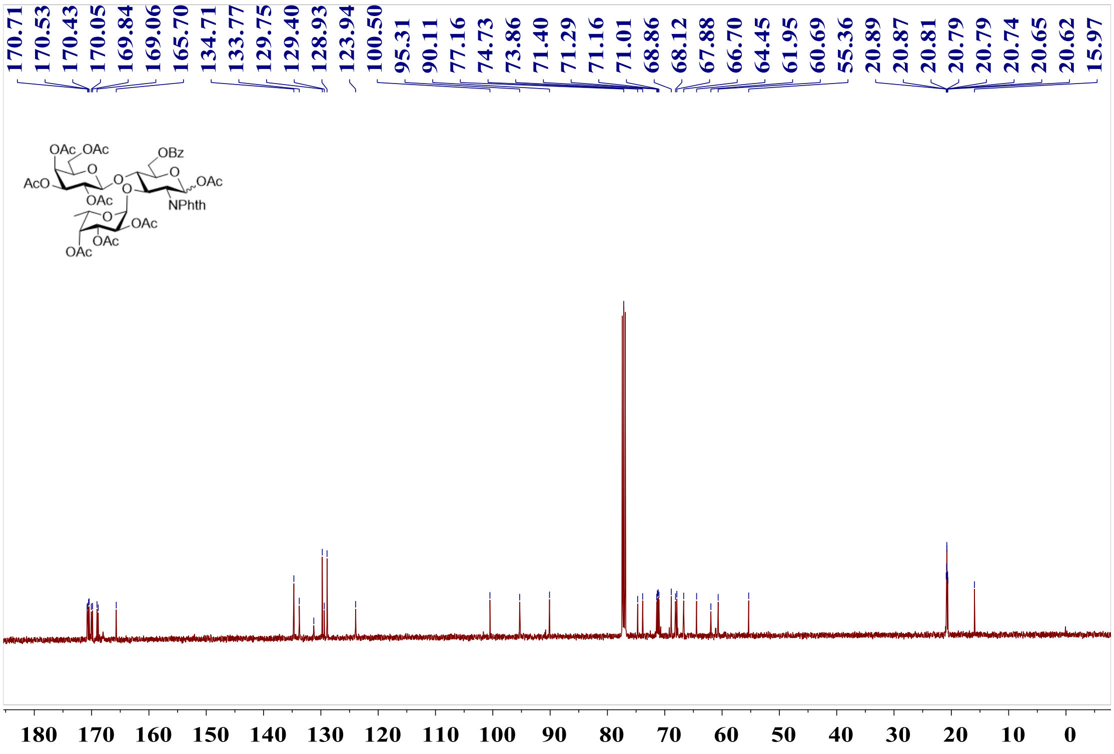

Supplementary Figure 96. ^{1}H NMR spectrum of compound **S33** (500 MHz, $CDCl_3$, 25 ^{o}C)

S33

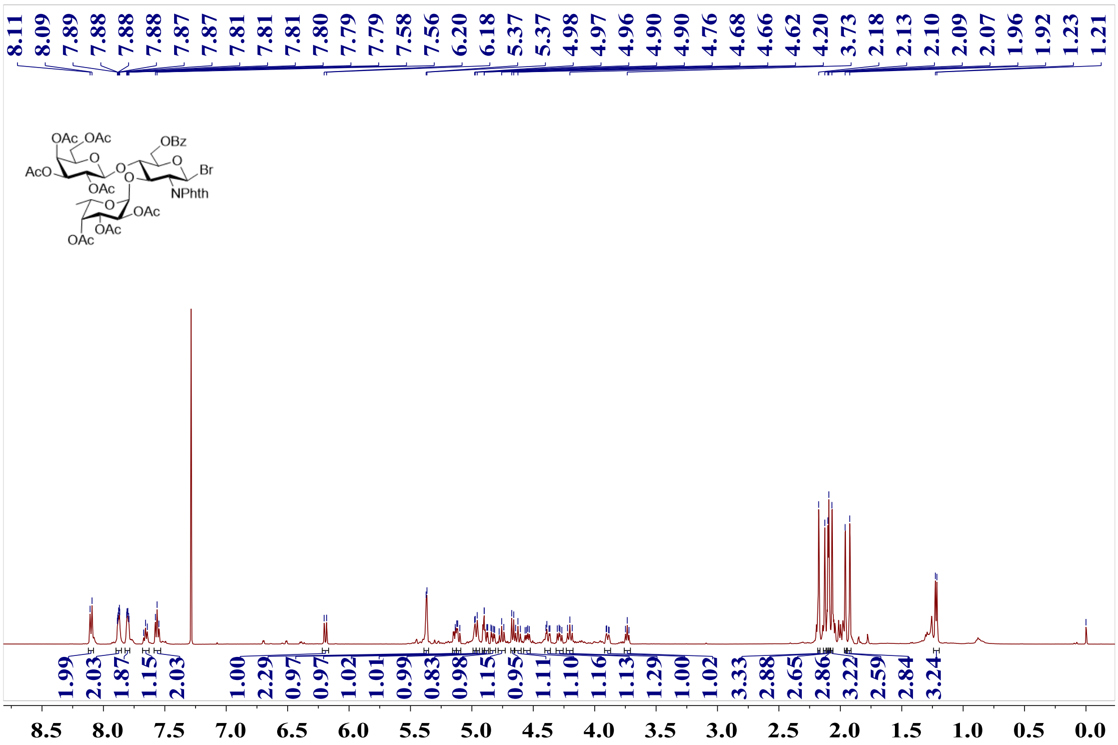

Supplementary Figure 97. ^{13}C NMR spectrum of compound **S33** (500 MHz, $CDCl_3$, 25 ^{o}C)

S33

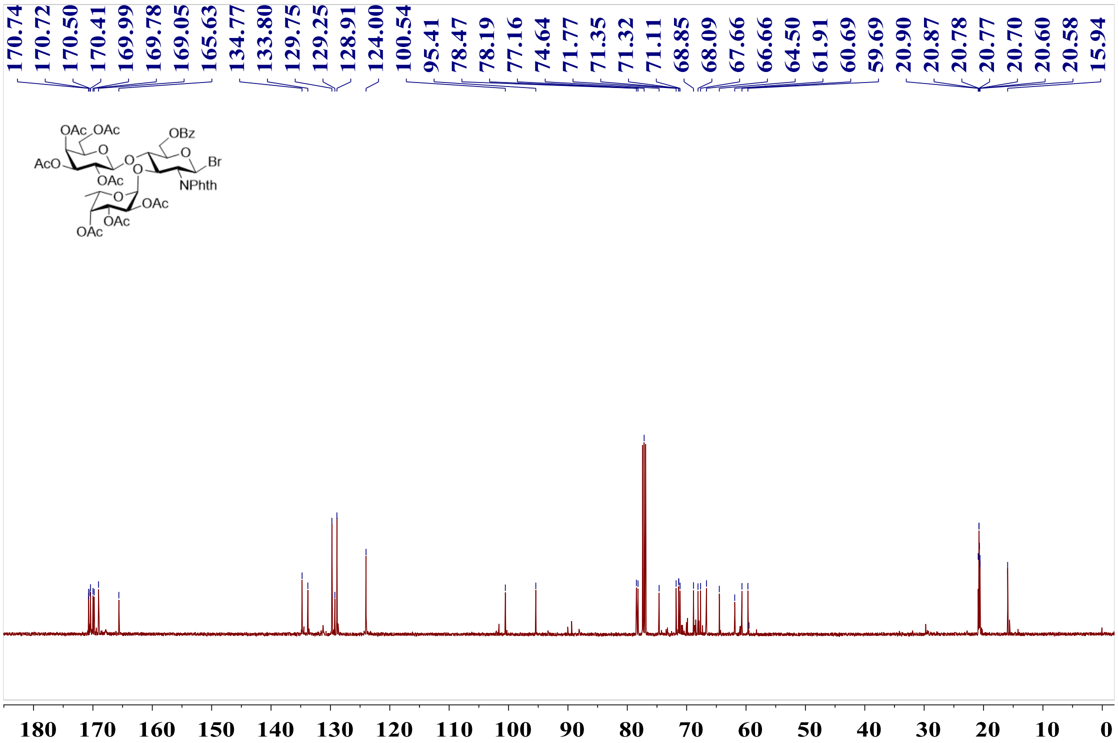

Supplementary Figure 98. ^{1}H NMR spectrum of compound **2a** (500 MHz, $CDCl_3$, 25 °C)

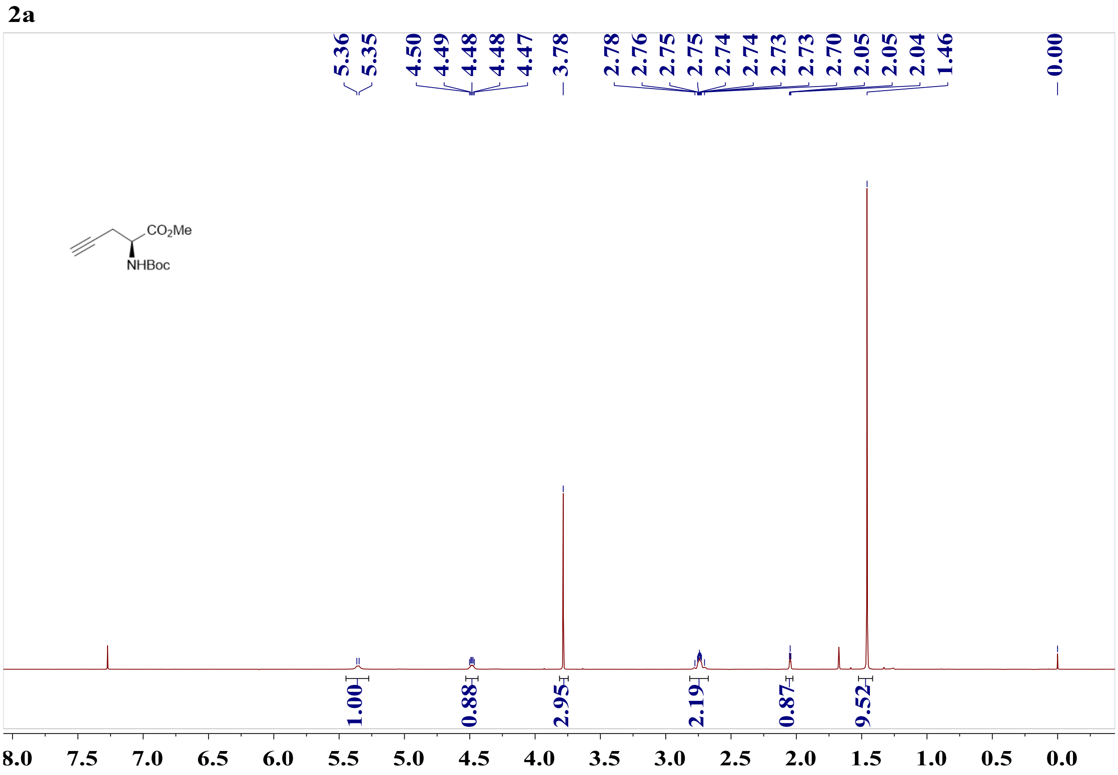

Supplementary Figure 99. ^{1}H NMR spectrum of compound **2b** (500 MHz, $CDCl_3$, 25 °C)

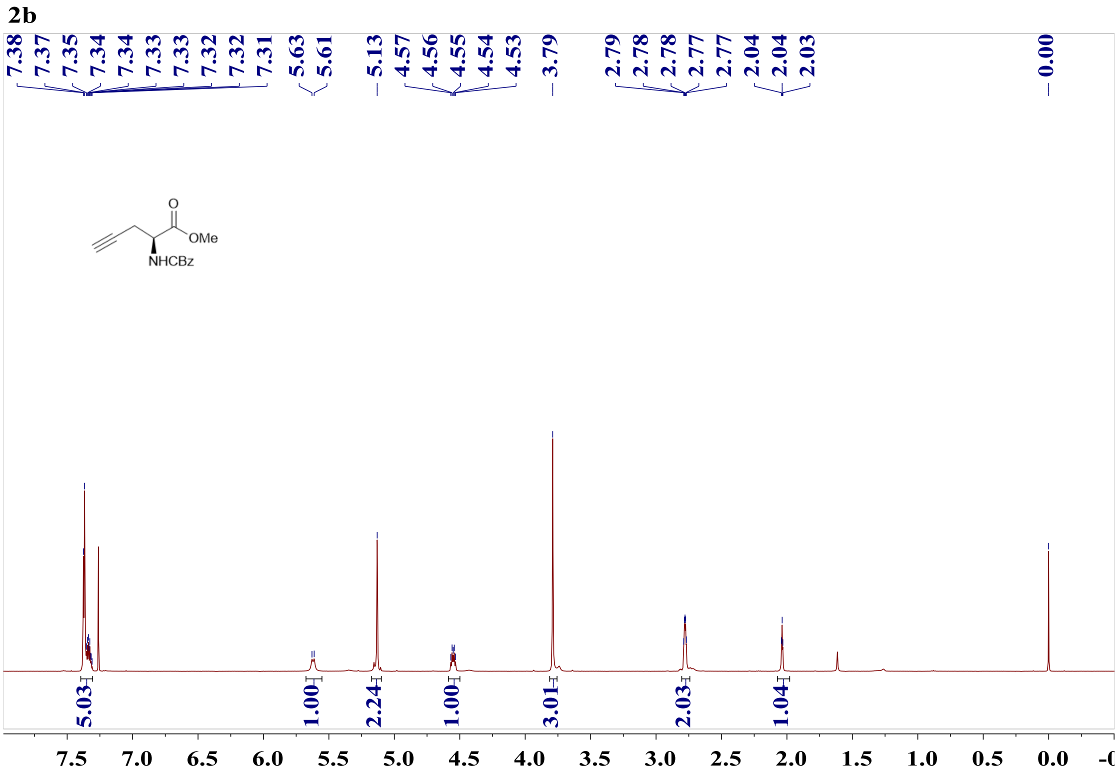

Supplementary Figure 100. ^{1}H NMR spectrum of compound **2c** (500 MHz, $CDCl_3$, 25 °C)

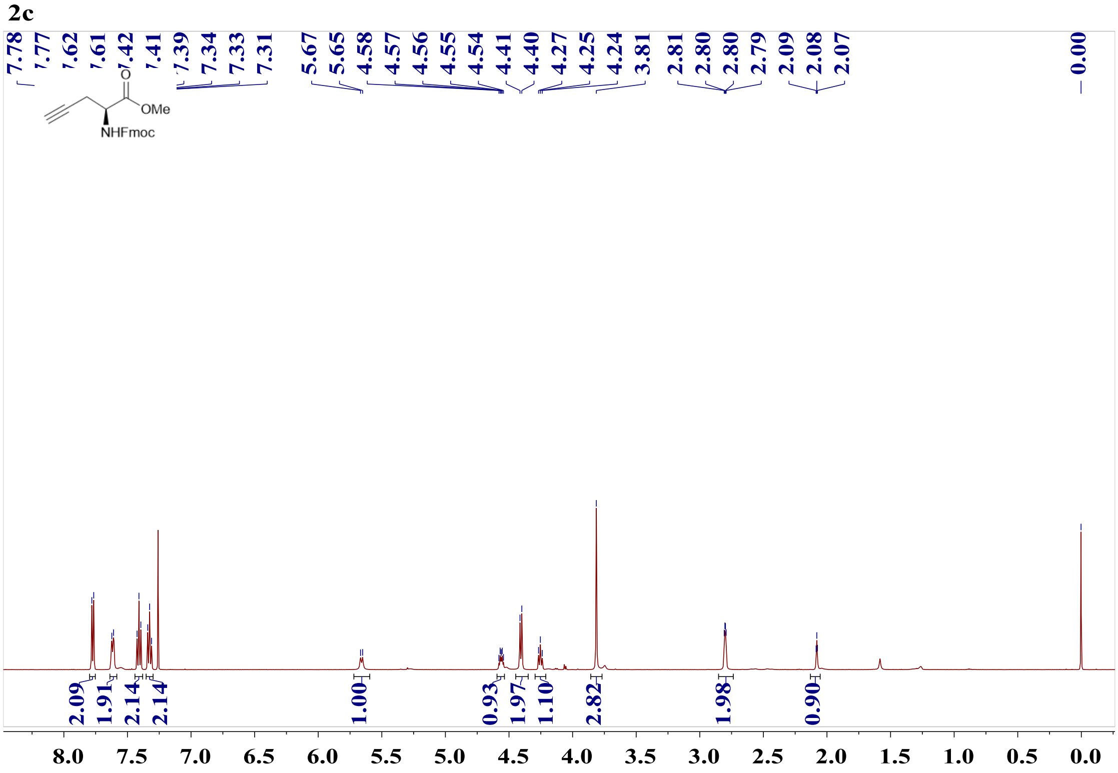

Supplementary Figure 101. ^{1}H NMR spectrum of compound **2d** (500 MHz, $CDCl_3$, 25 °C)

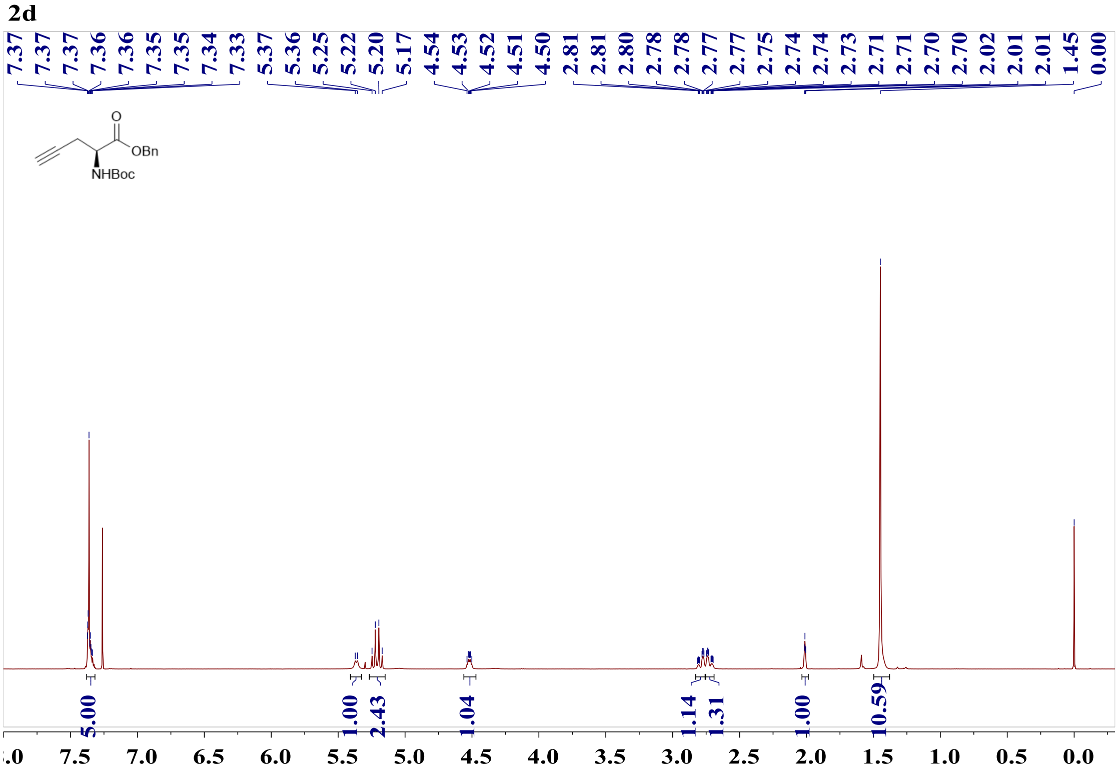

Supplementary Figure 102. ^{1}H NMR spectrum of compound **2e** (500 MHz, $CDCl_3$, 25 °C)

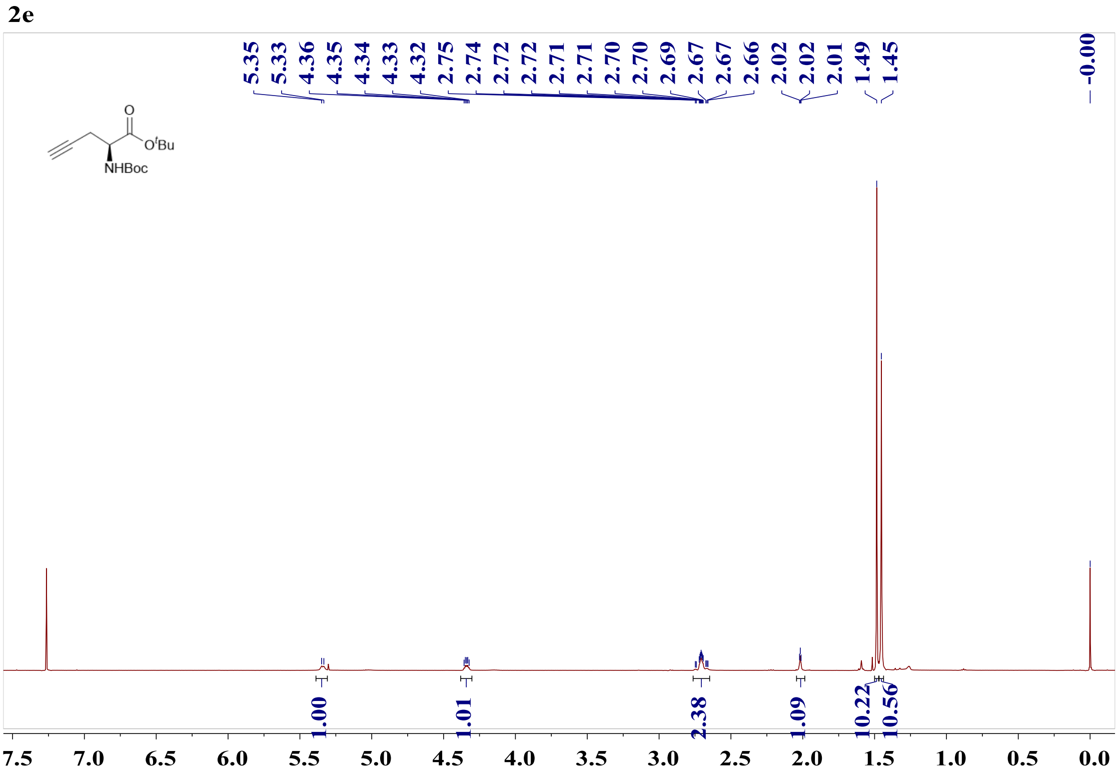

Supplementary Figure 103. ^{1}H NMR spectrum of compound **2f** (500 MHz, $CDCl_3$, 25 °C)

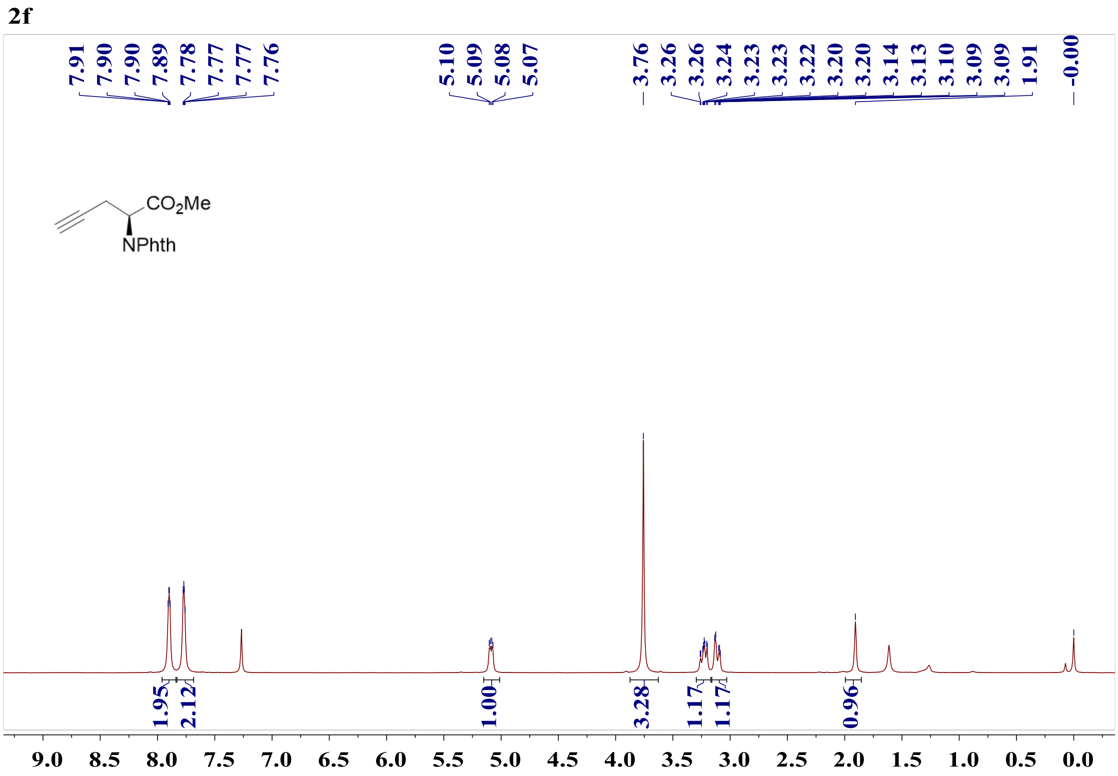

Supplementary Figure 104. ^{1}H NMR spectrum of compound **2g** (500 MHz, $CDCl_3$, 25 °C)

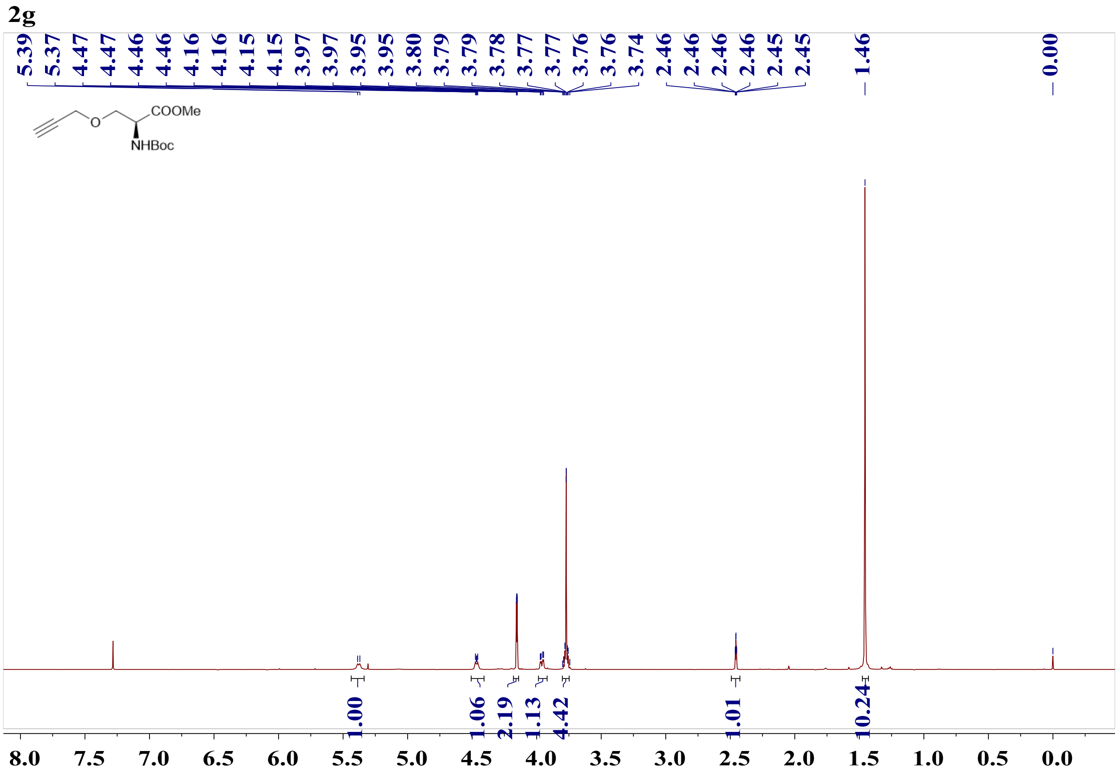

Supplementary Figure 105. ^{1}H NMR spectrum of compound **2h** (500 MHz, $CDCl_3$, 25 °C)

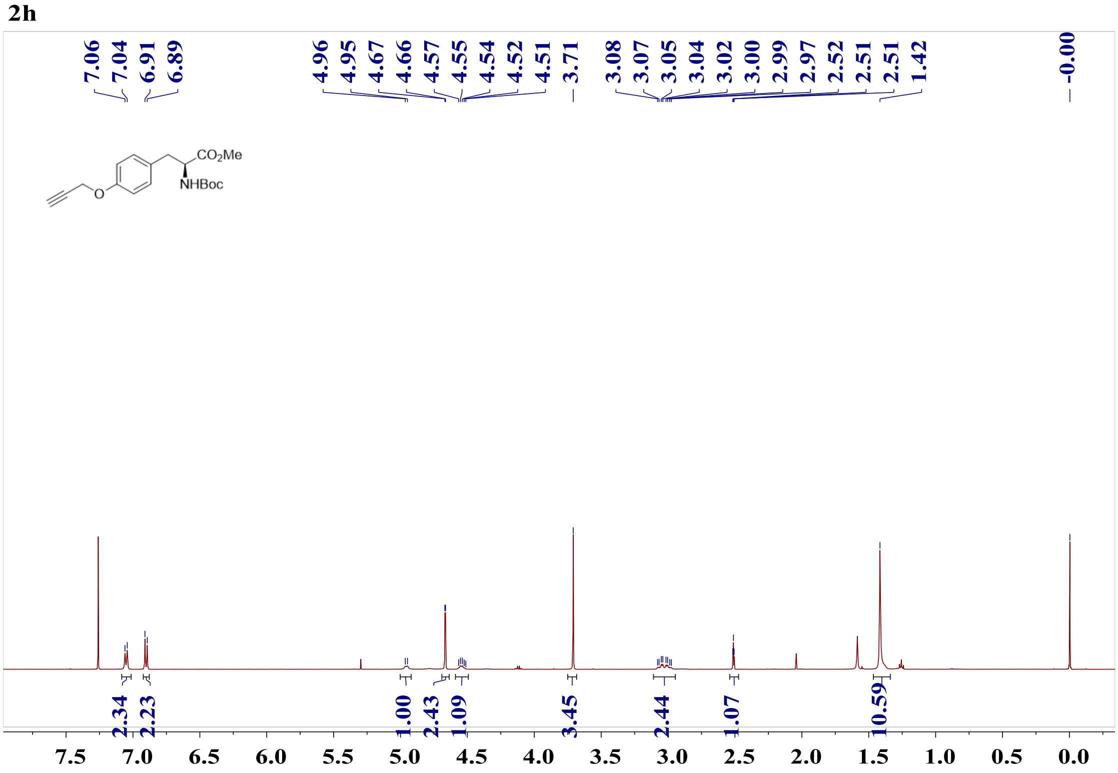

Supplementary Figure 106. ^{1}H NMR spectrum of compound **2i** (500 MHz, $CDCl_3$, 25 °C)

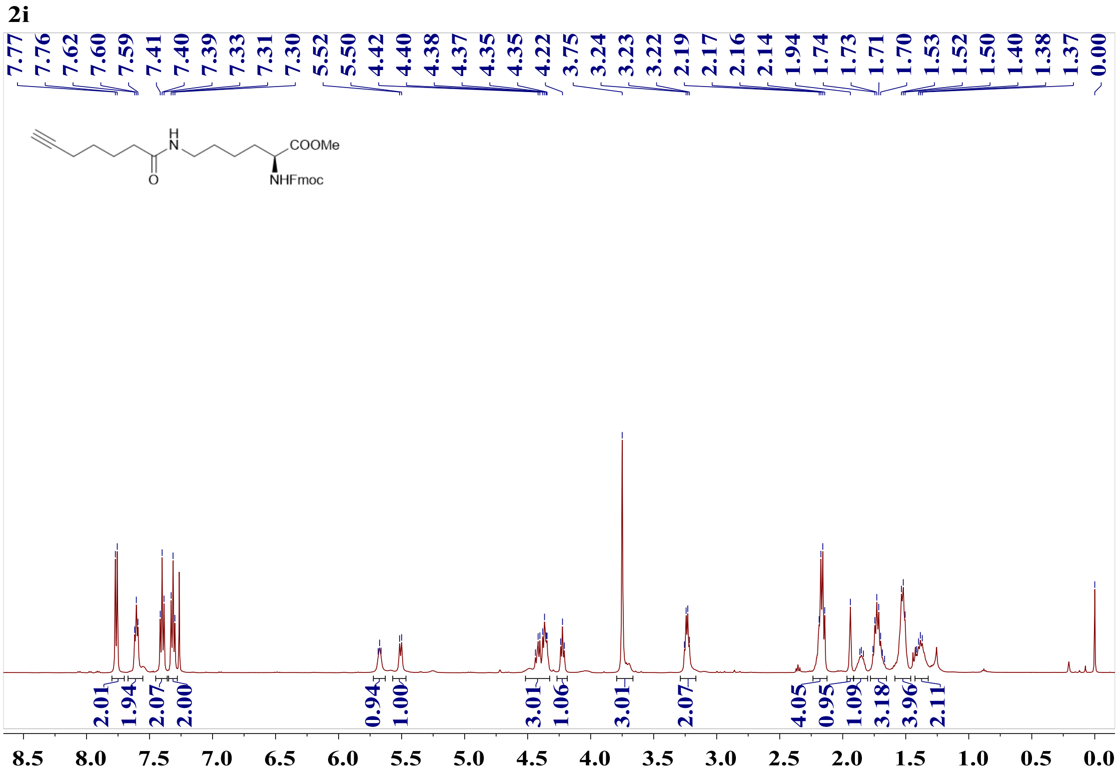

Supplementary Figure 107. ^{13}C NMR spectrum of compound **2i** (125 MHz, $CDCl_3$, 25 °C)

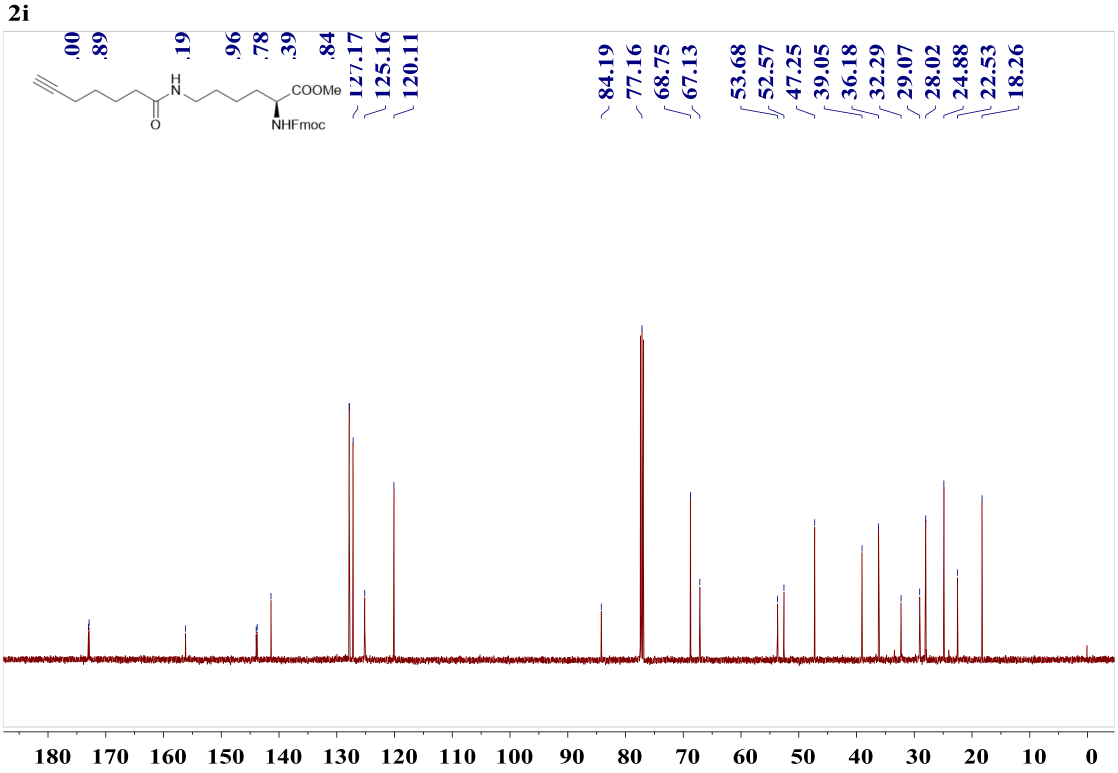

Supplementary Figure 108. ^{1}H NMR spectrum of compound **2j** (500 MHz, $CDCl_3$, 25 ^{o}C)

2j

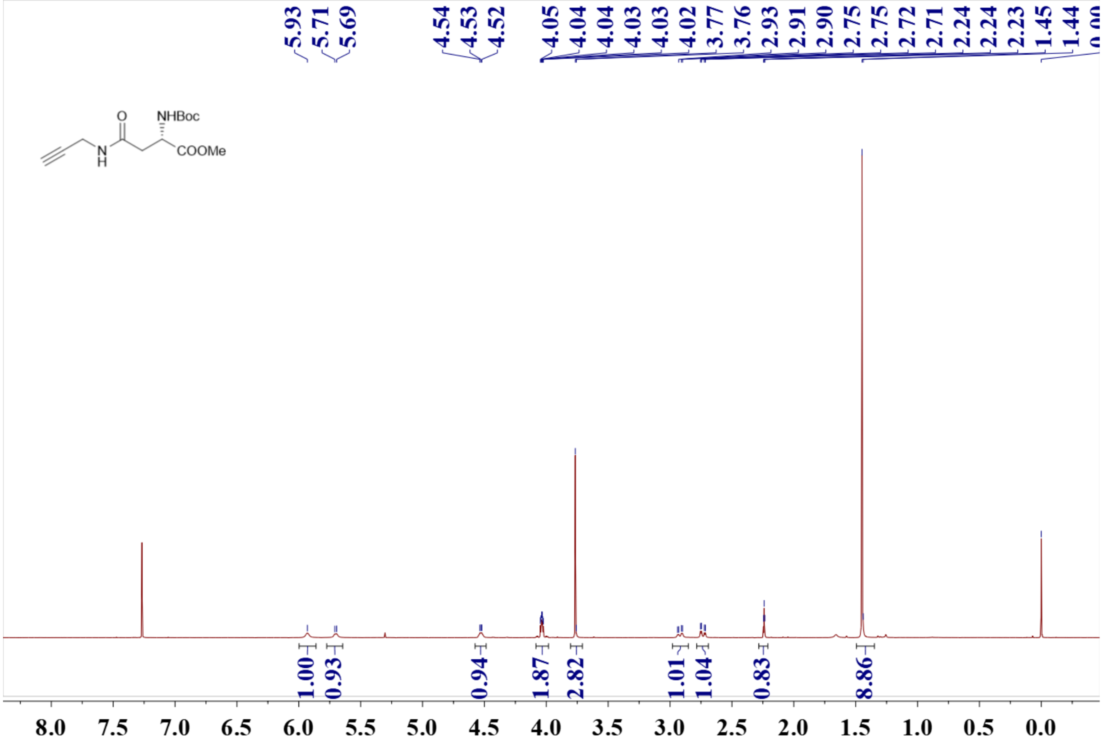

Supplementary Figure 109. ^{1}H NMR spectrum of compound **2k** (500 MHz, $CDCl_3$, 25 °C)

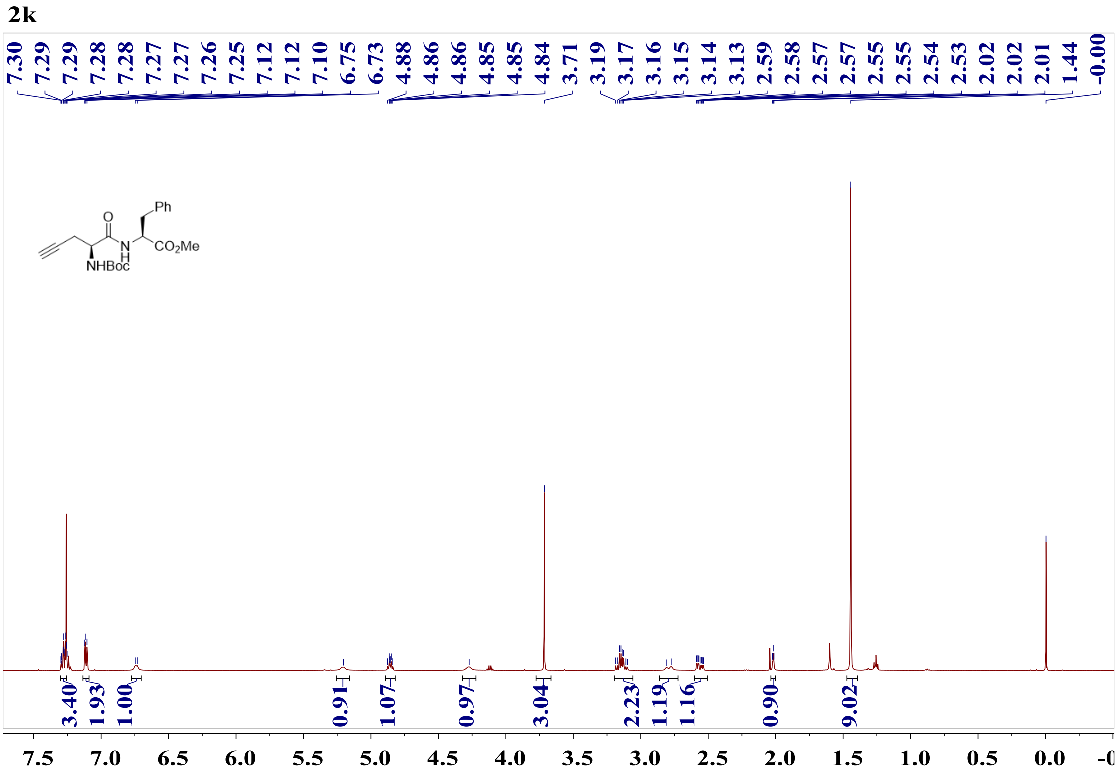

Supplementary Figure 110. ^{13}C NMR spectrum of compound **2k** (125 MHz, $CDCl_3$, 25 °C)

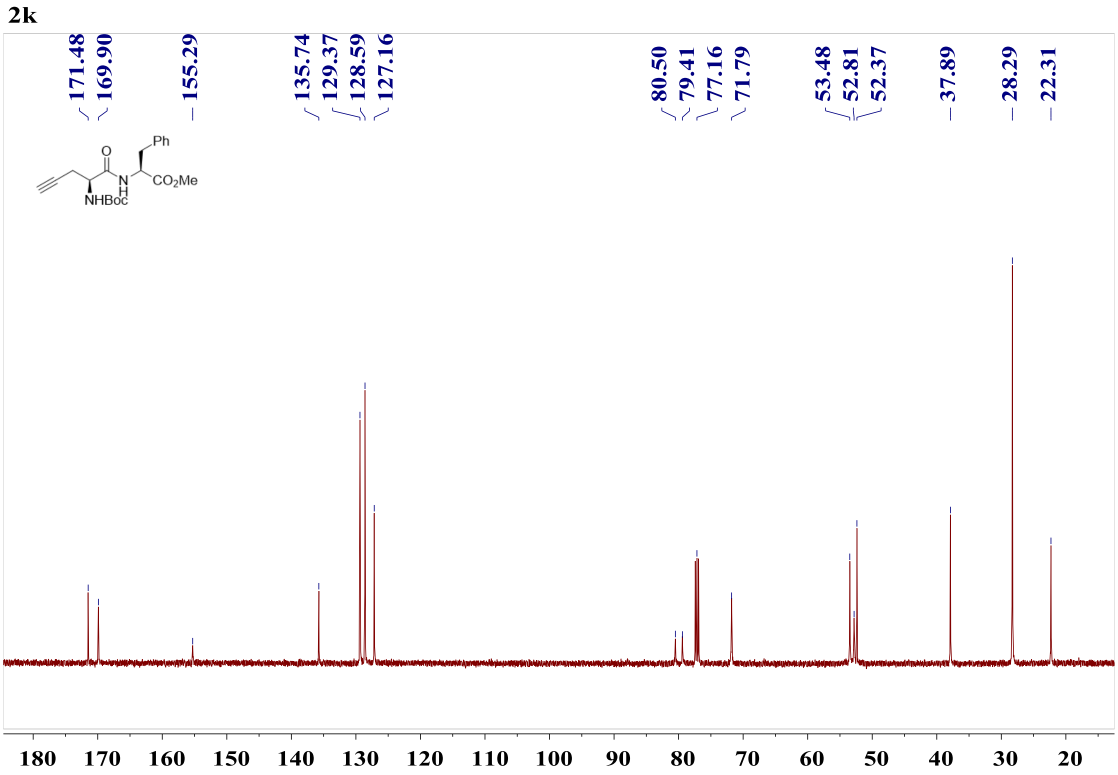

Supplementary Figure 111. ^{1}H NMR spectrum of compound **2l** (500 MHz, $CDCl_3$, 25 °C)

2l

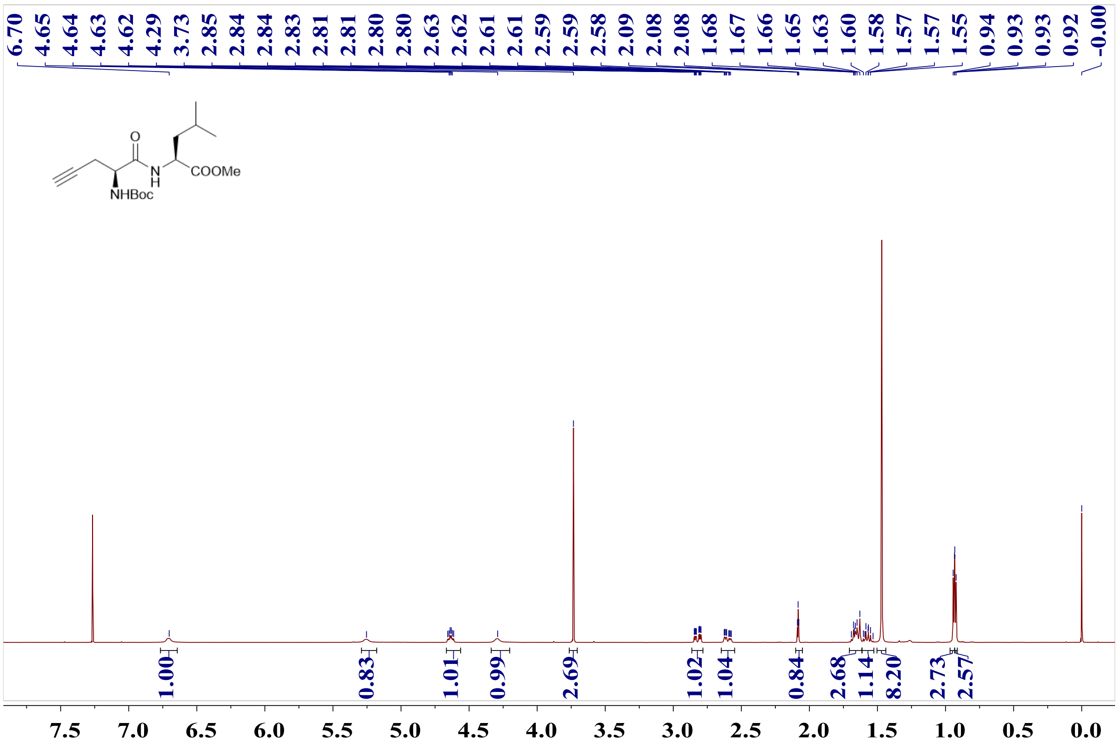

Supplementary Figure 112. ^{13}C NMR spectrum of compound **2l** (125 MHz, $CDCl_3$, 25 °C)

2l

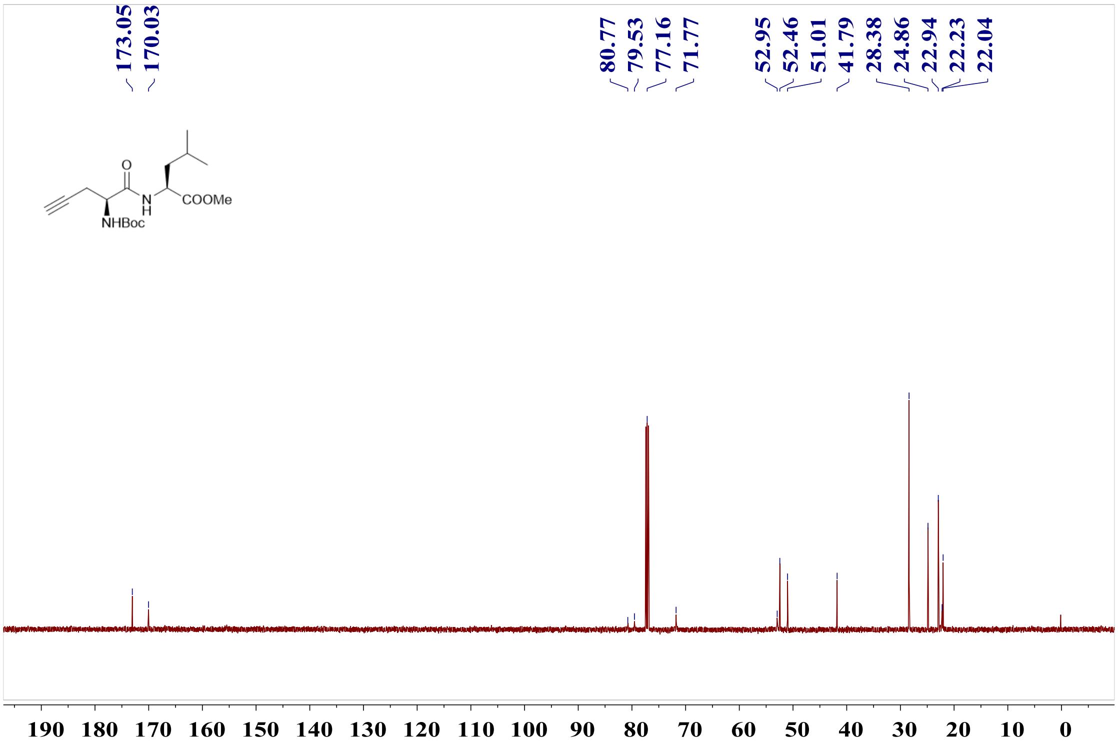

Supplementary Figure 113. ^{1}H NMR spectrum of compound **2m** (500 MHz, $CDCl_3$, 25 °C)

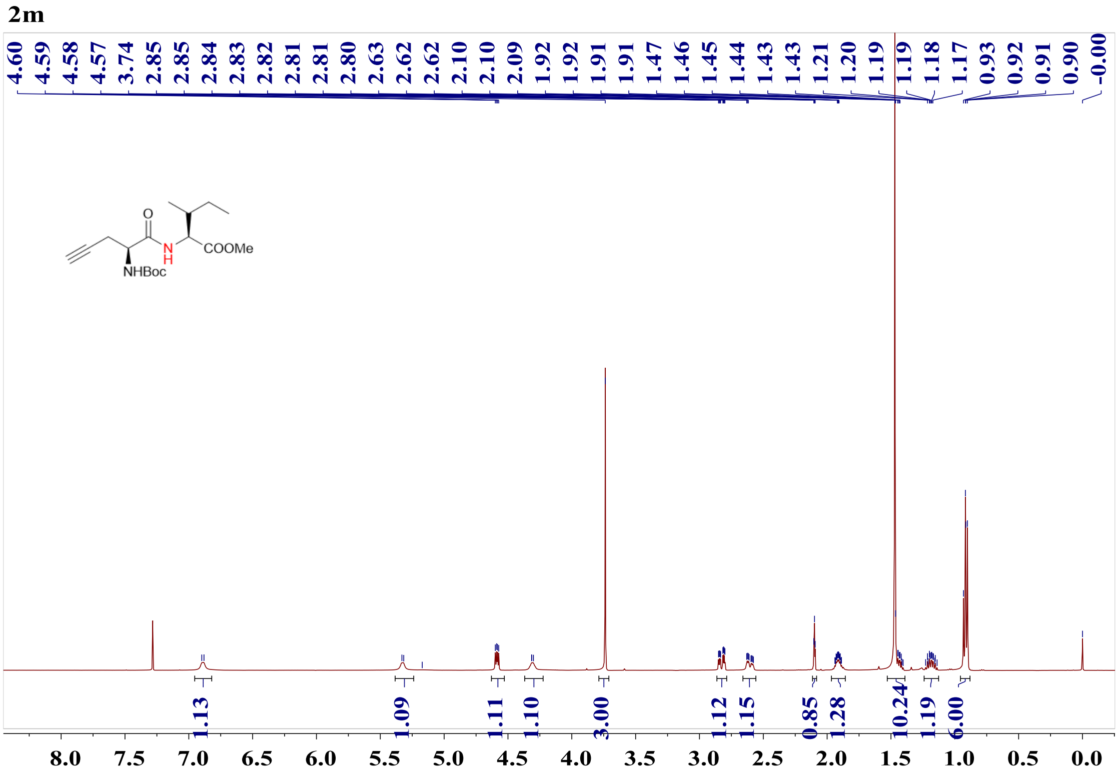

Supplementary Figure 114. ^{13}C NMR spectrum of compound **2m** (125 MHz, $CDCl_3$, 25 °C)

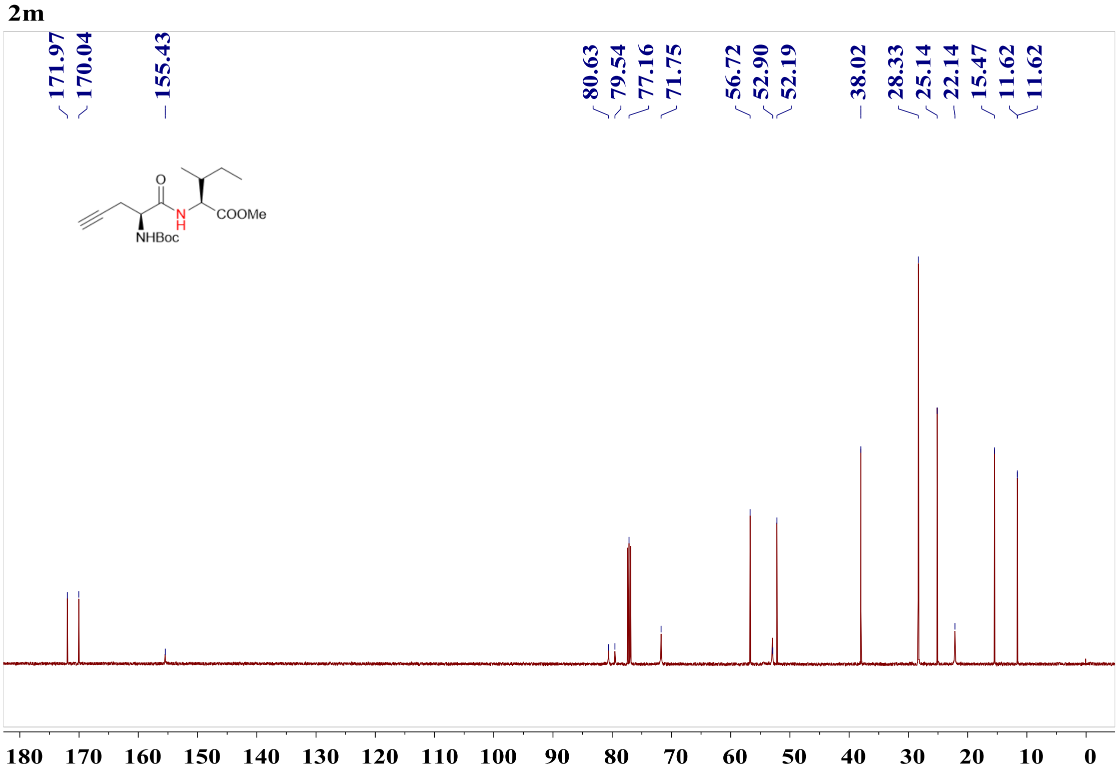

Supplementary Figure 115. ^{1}H NMR spectrum of compound **2n** (500 MHz, $CDCl_3$, 25 °C)

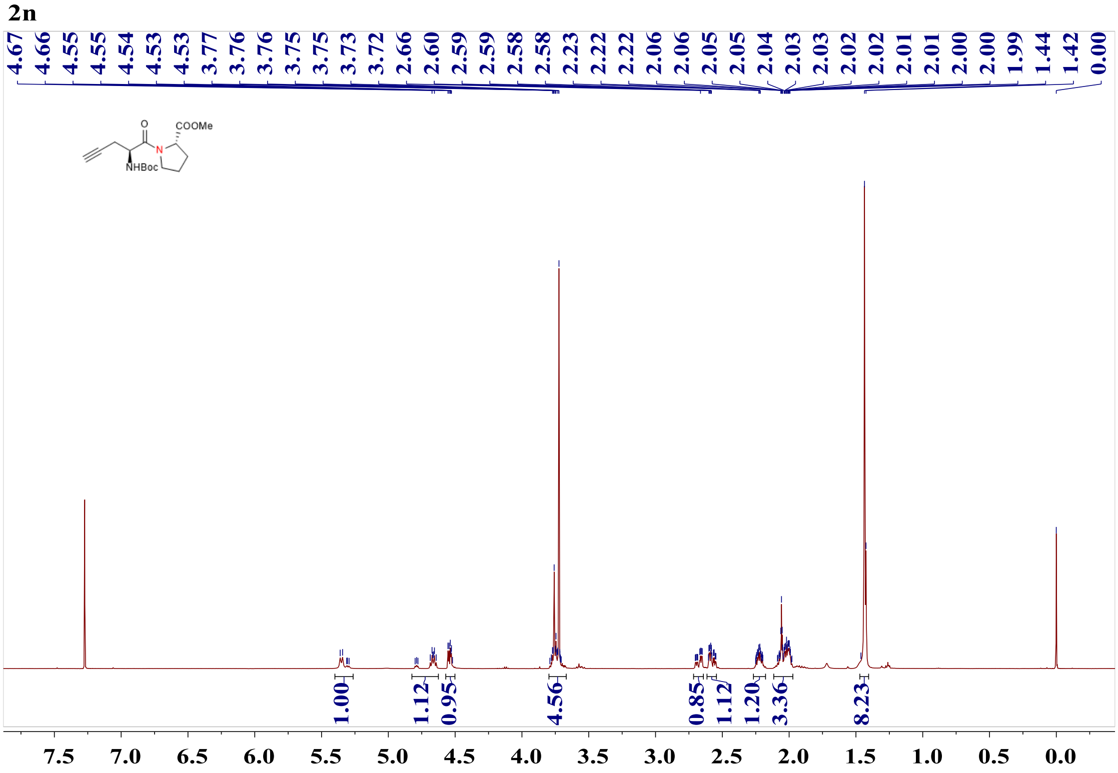

Supplementary Figure 116. ^{13}C NMR spectrum of compound **2n** (125 MHz, $CDCl_3$, 25 °C)

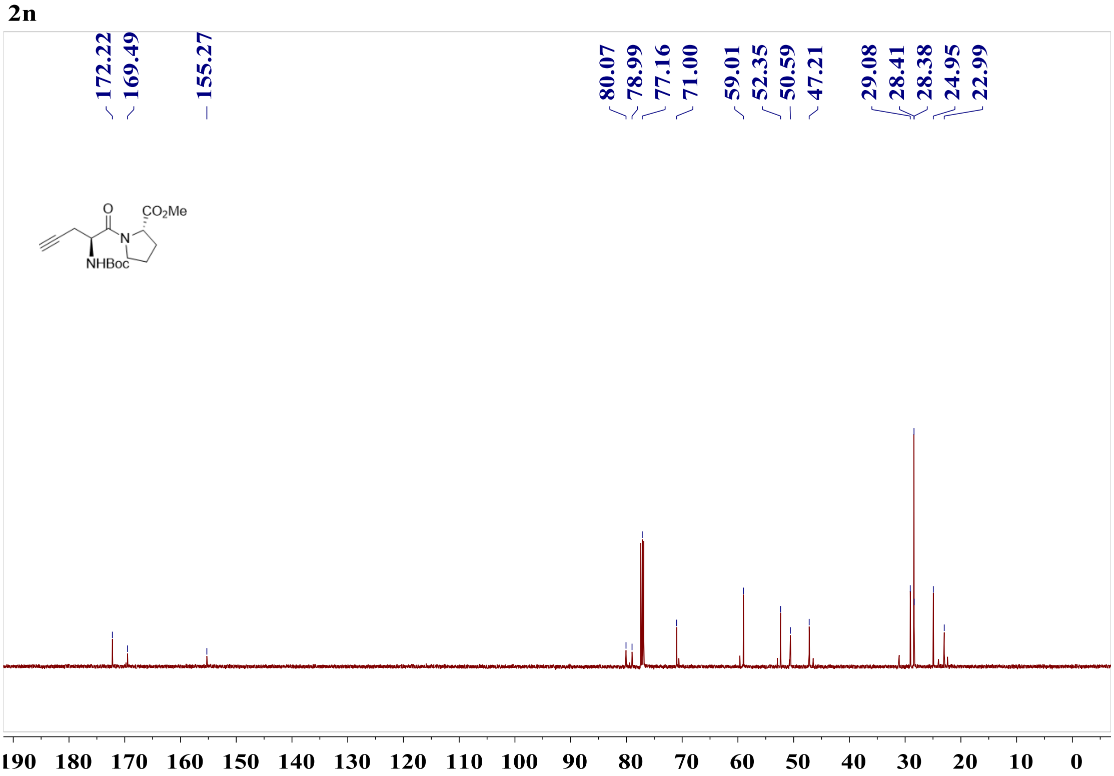

Supplementary Figure 117. ^{1}H NMR spectrum of compound **2o** (500 MHz, $CDCl_3$, 25 °C)

2o

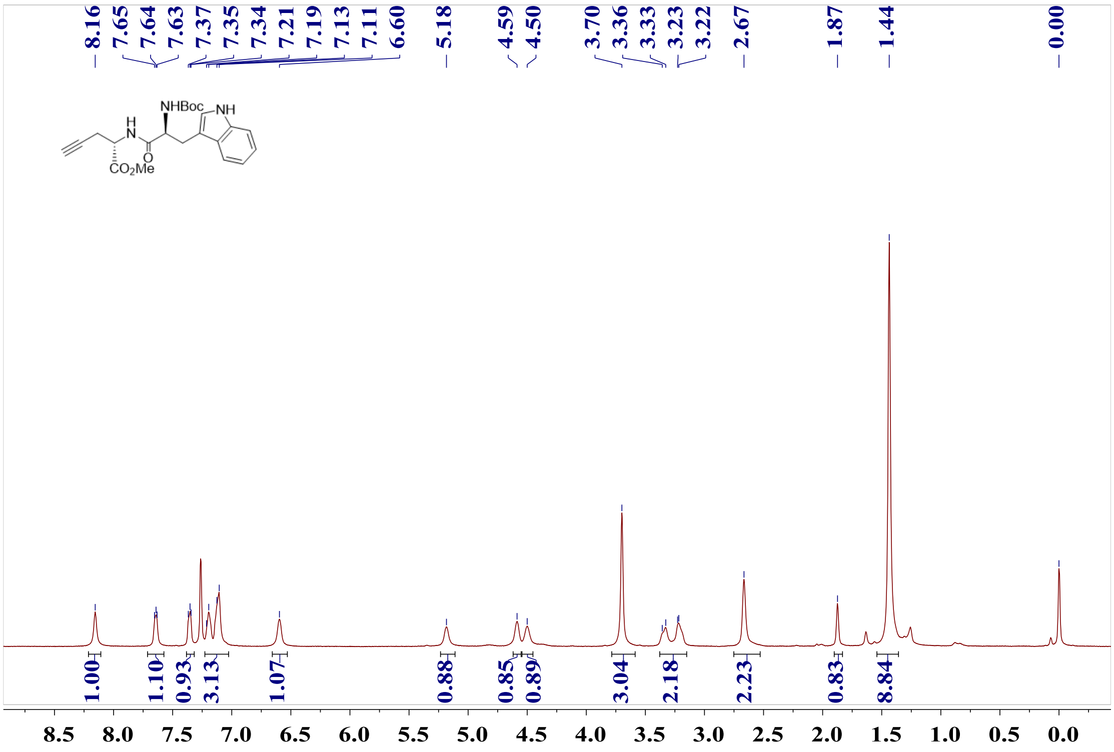

Supplementary Figure 118. ^{13}C NMR spectrum of compound **2o** (125 MHz, $CDCl_3$, 25 °C)

2o

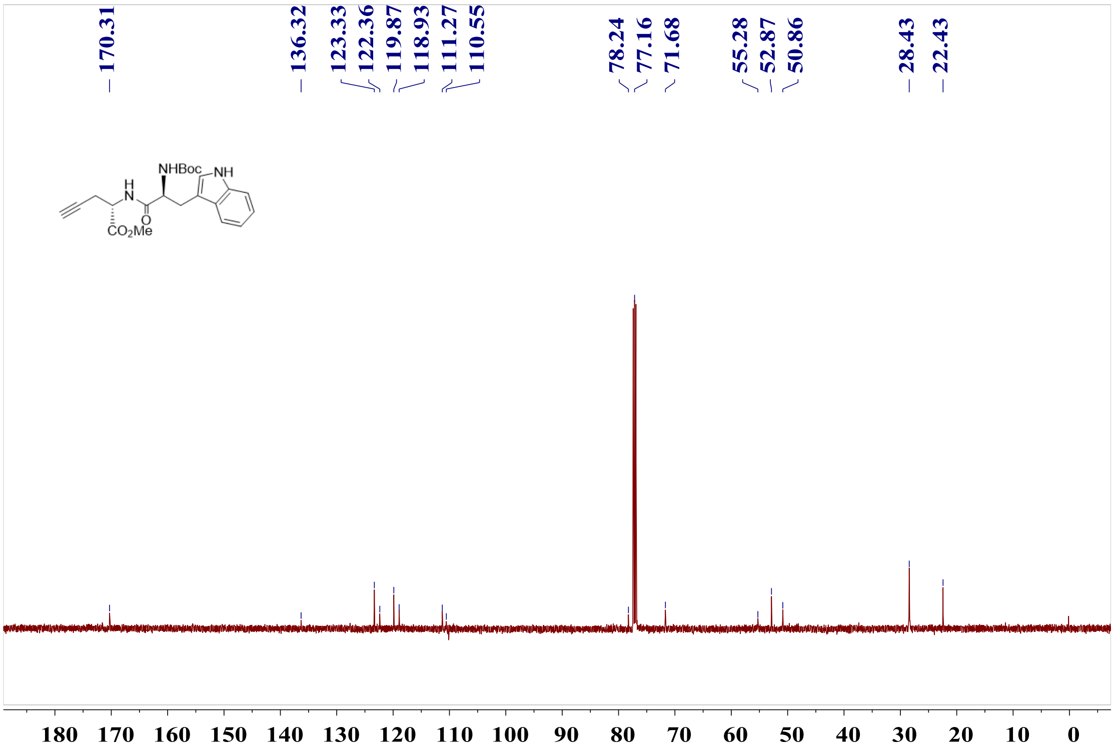

Supplementary Figure 119. ^{1}H NMR spectrum of compound **2p** (500 MHz, $CDCl_3$, 25 °C)

2p

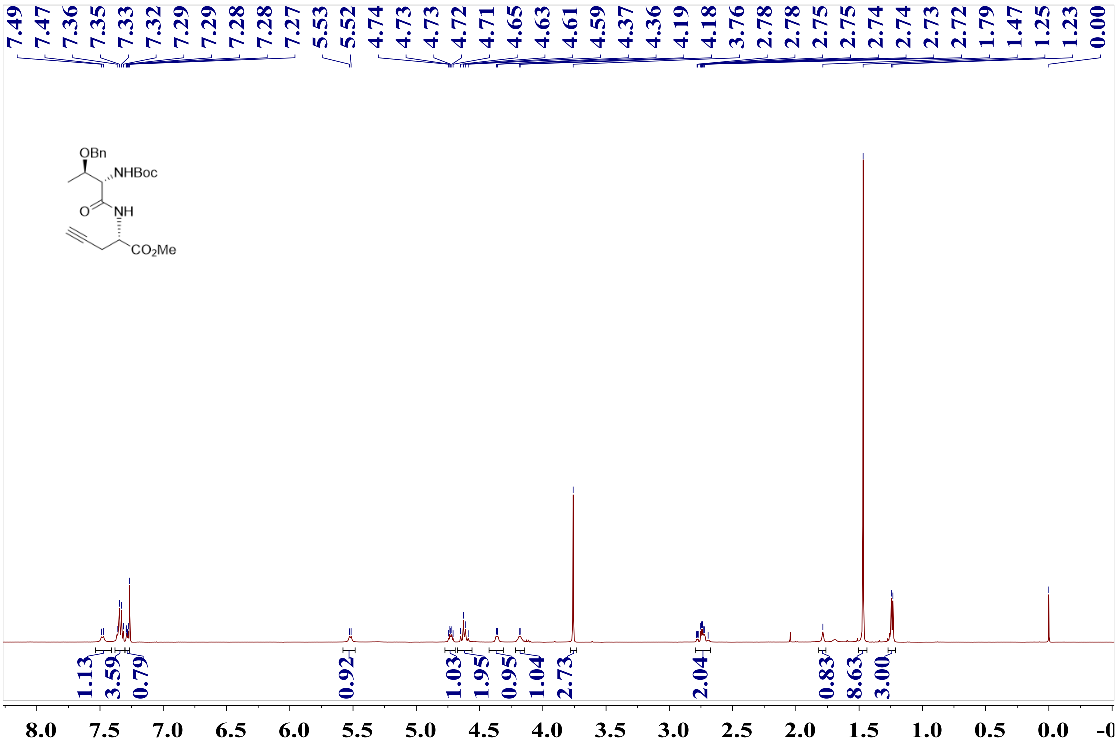

Supplementary Figure 120. ^{13}C NMR spectrum of compound **2p** (125 MHz, $CDCl_3$, 25 °C)

2p

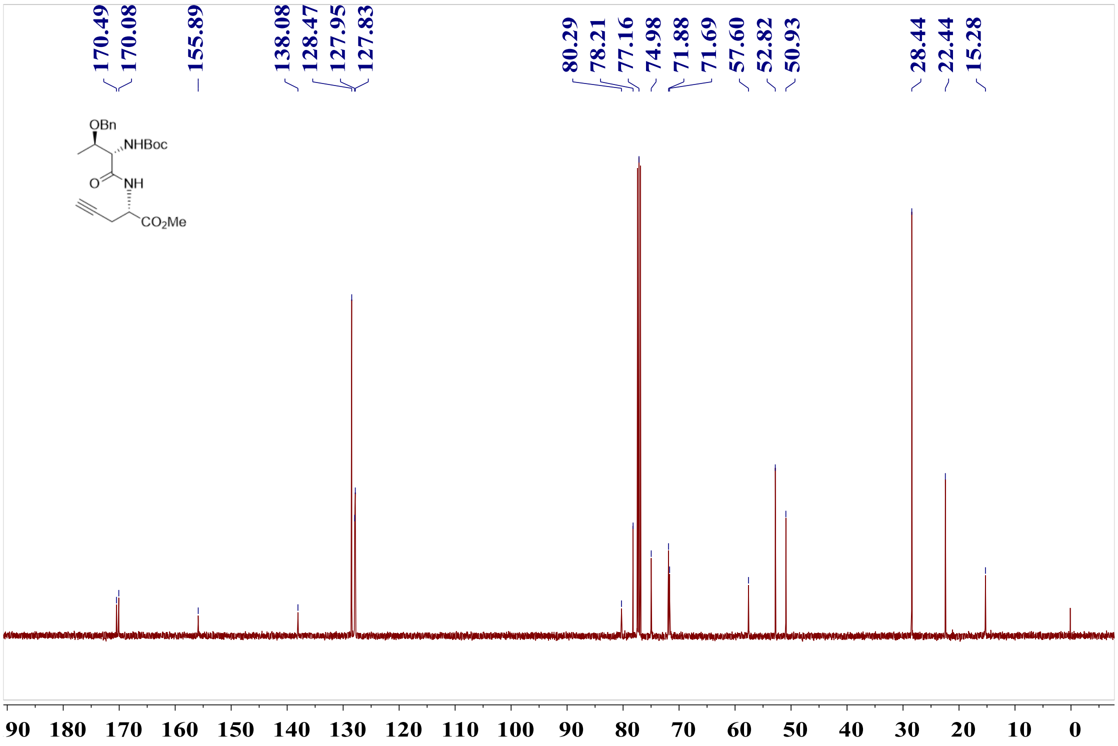

Supplementary Figure 121. ^{1}H NMR spectrum of compound **2q** (500 MHz, $CDCl_3$, 25 °C)

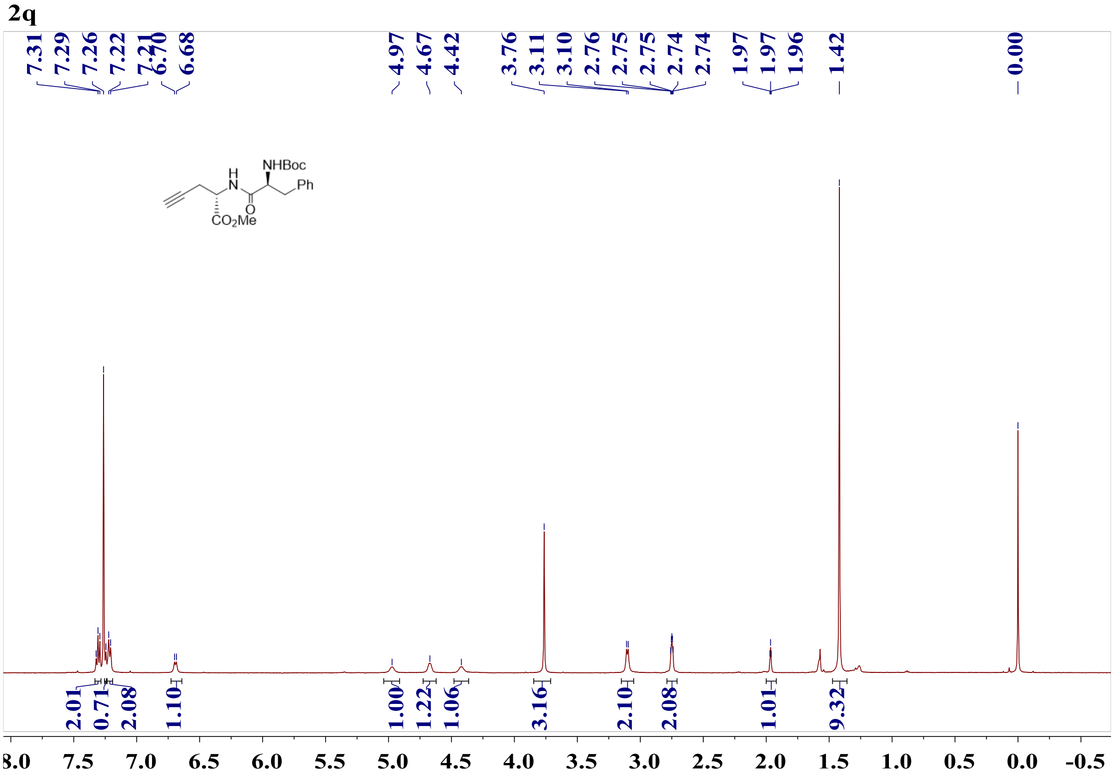

Supplementary Figure 122. ^{13}C NMR spectrum of compound **2q** (125 MHz, $CDCl_3$, 25 °C)

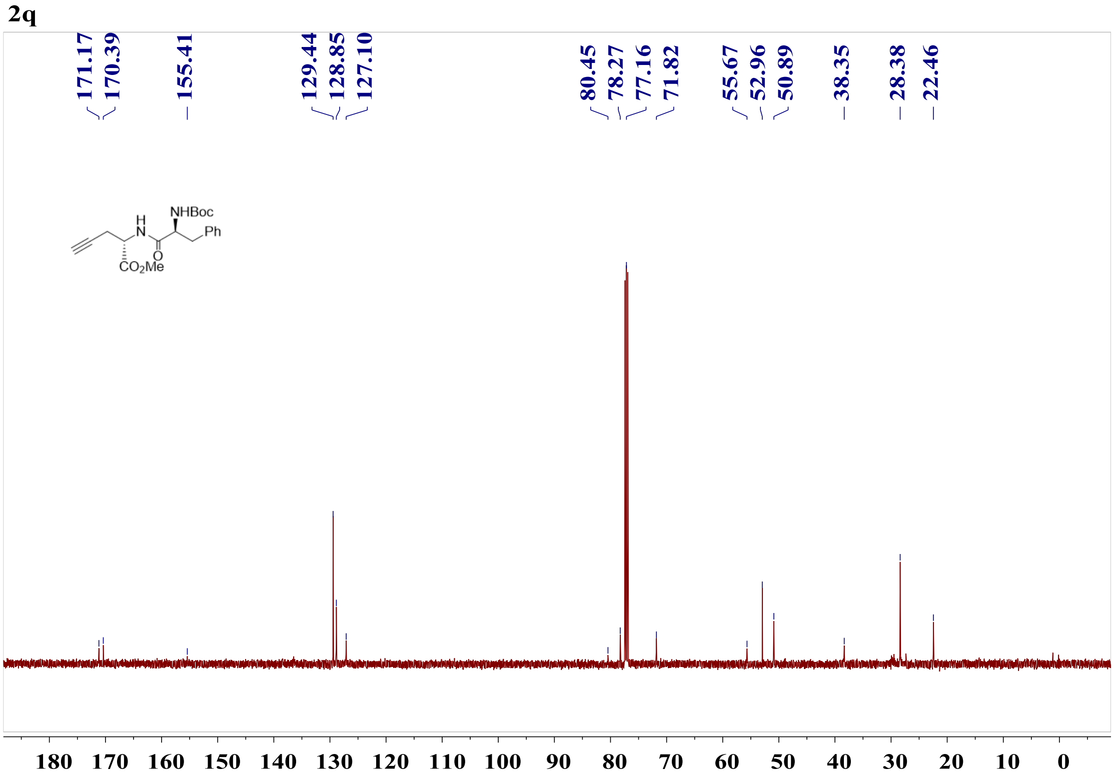

Supplementary Figure 123. ^{1}H NMR spectrum of compound **2r** (500 MHz, $CDCl_3$, 25 °C)

2r

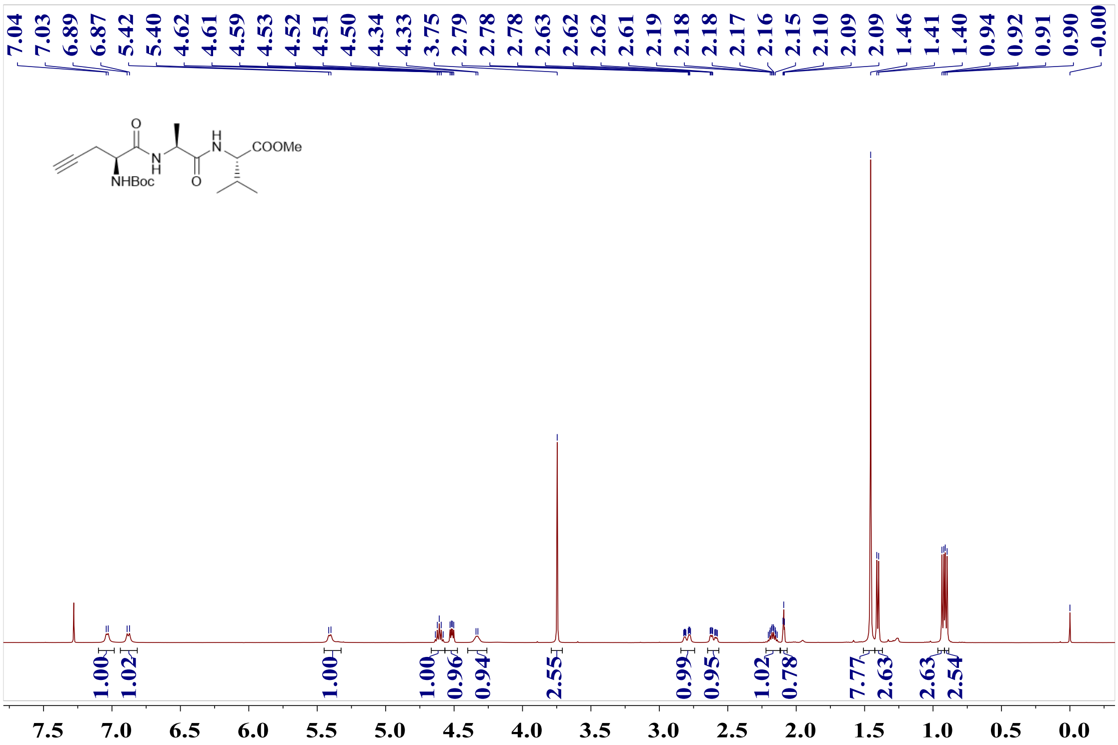

Supplementary Figure 124. ^{13}C NMR spectrum of compound **2r** (125 MHz, $CDCl_3$, 25 °C)

2r

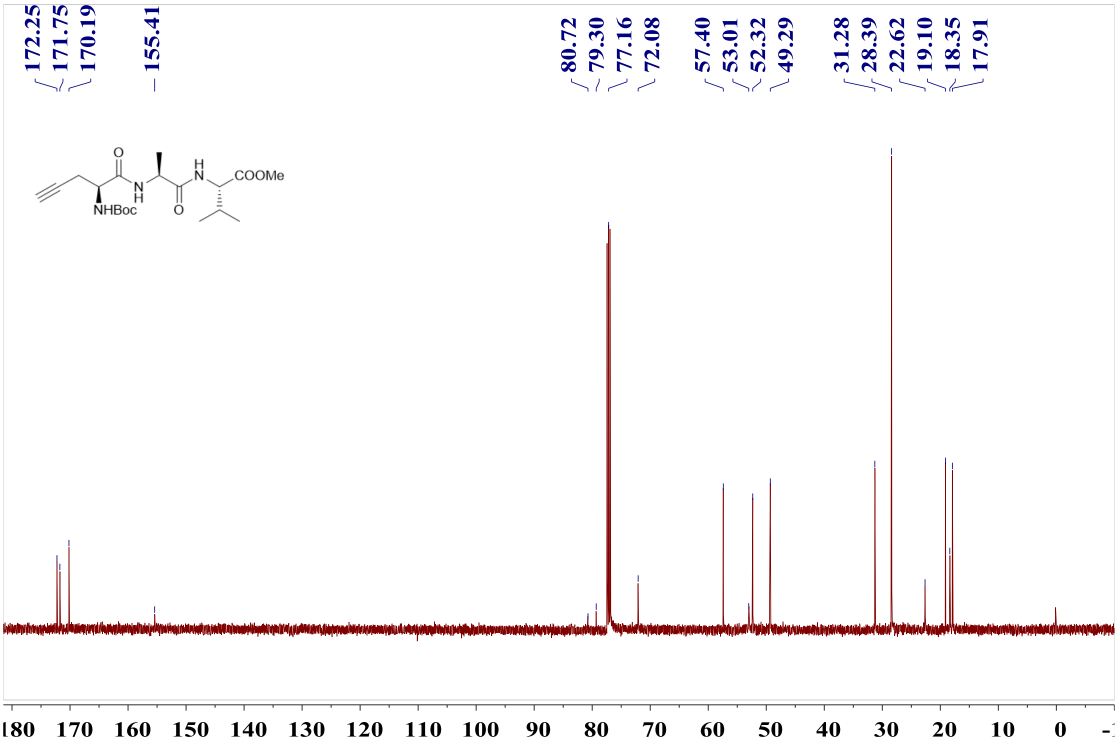

Supplementary Figure 125. ^{1}H NMR spectrum of compound **2s** (500 MHz, $CDCl_3$, 25 ^{o}C)

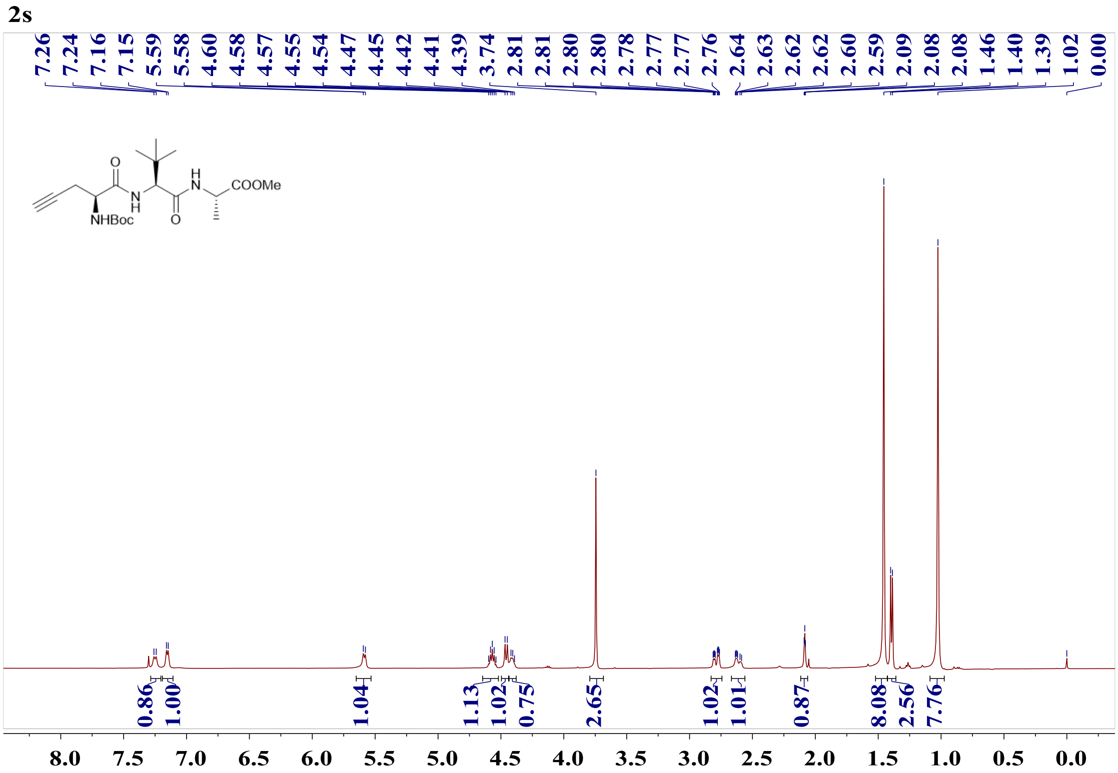

Supplementary Figure 126. ^{13}C NMR spectrum of compound **2s** (125 MHz, $CDCl_3$, 25 ^{o}C)

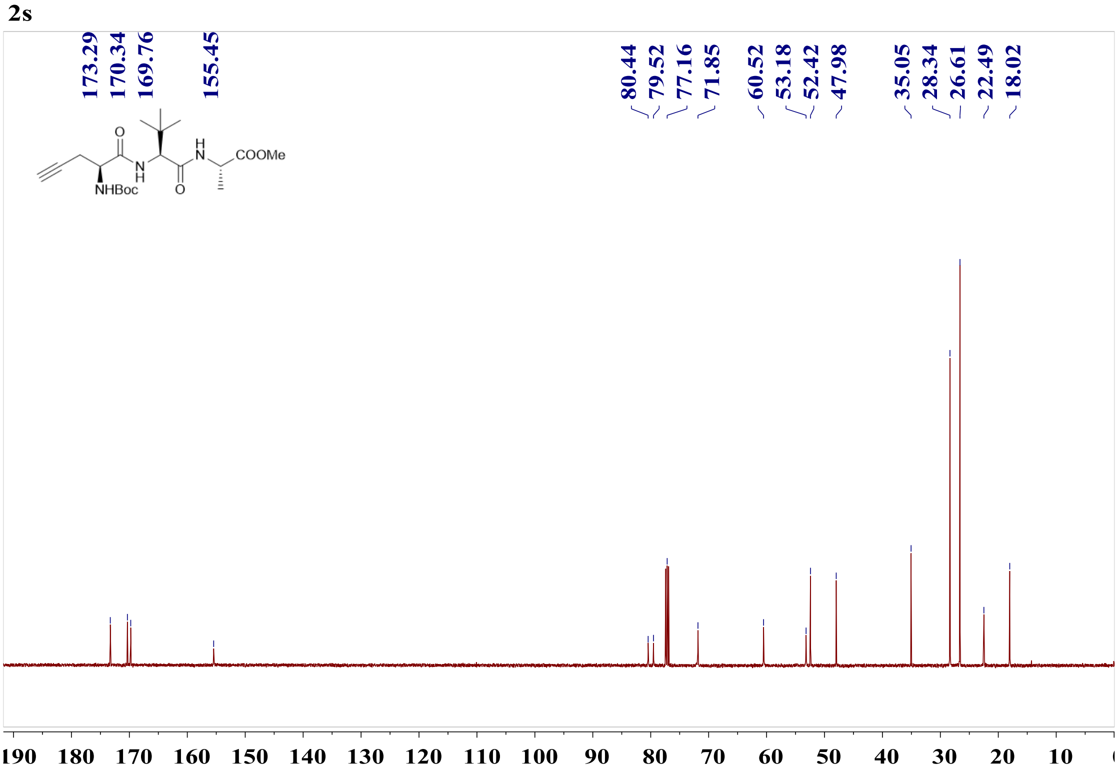

Supplementary Figure 127. ^{1}H NMR spectrum of compound **2t** (500 MHz, $CDCl_3$, 25 ^{o}C)

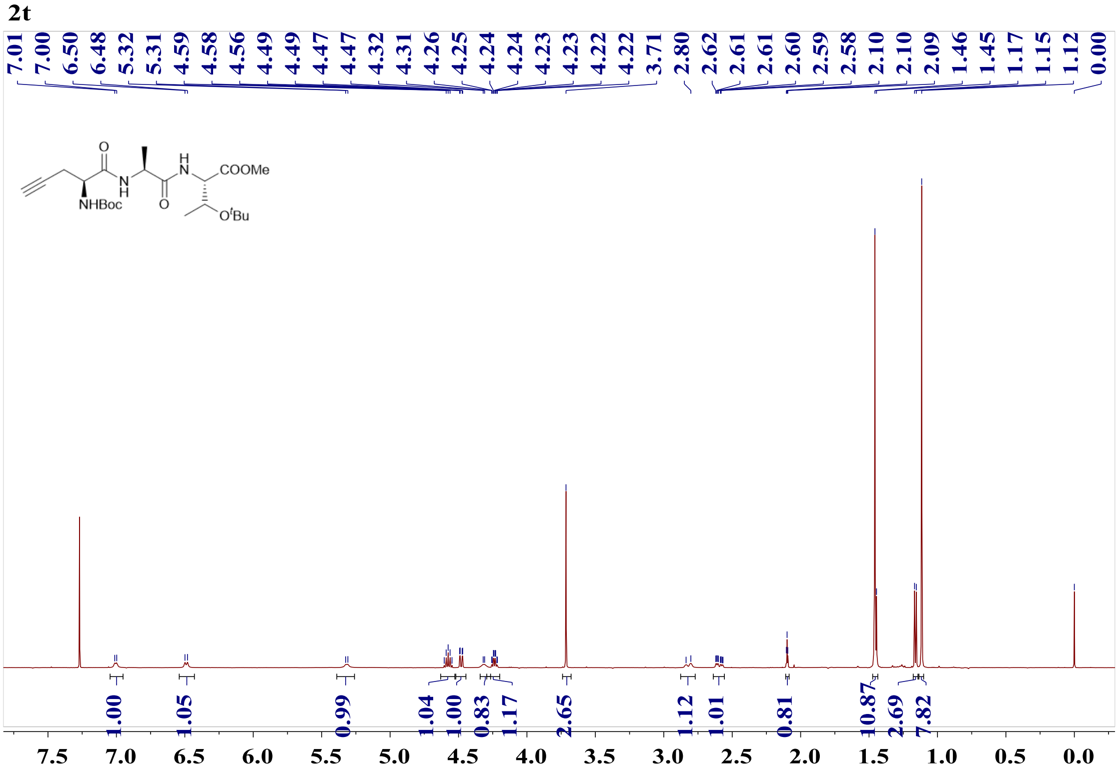

Supplementary Figure 128. ^{13}C NMR spectrum of compound **2t** (125 MHz, $CDCl_3$, 25 ^{o}C)

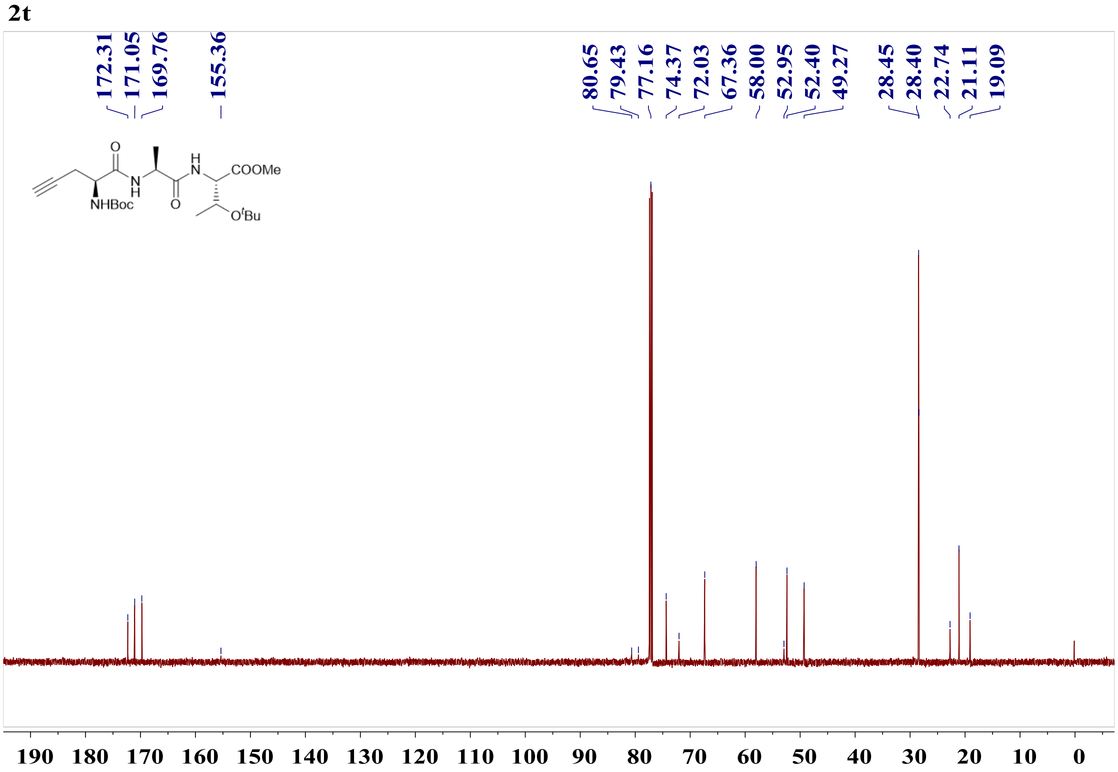

Supplementary Figure 129. ^{1}H NMR spectrum of compound **2u** (500 MHz, $CDCl_3$, 25 ^{o}C)

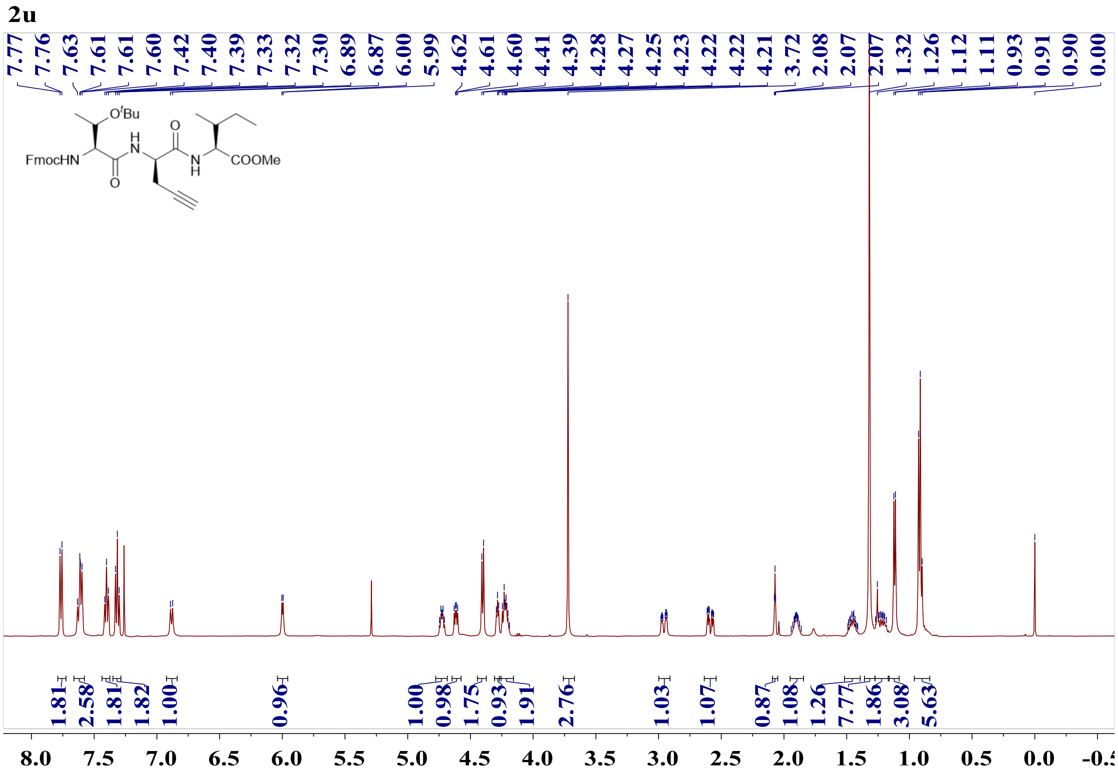

Supplementary Figure 130. ^{13}C NMR spectrum of compound **2u** (125 MHz, $CDCl_3$, 25 ^{o}C)

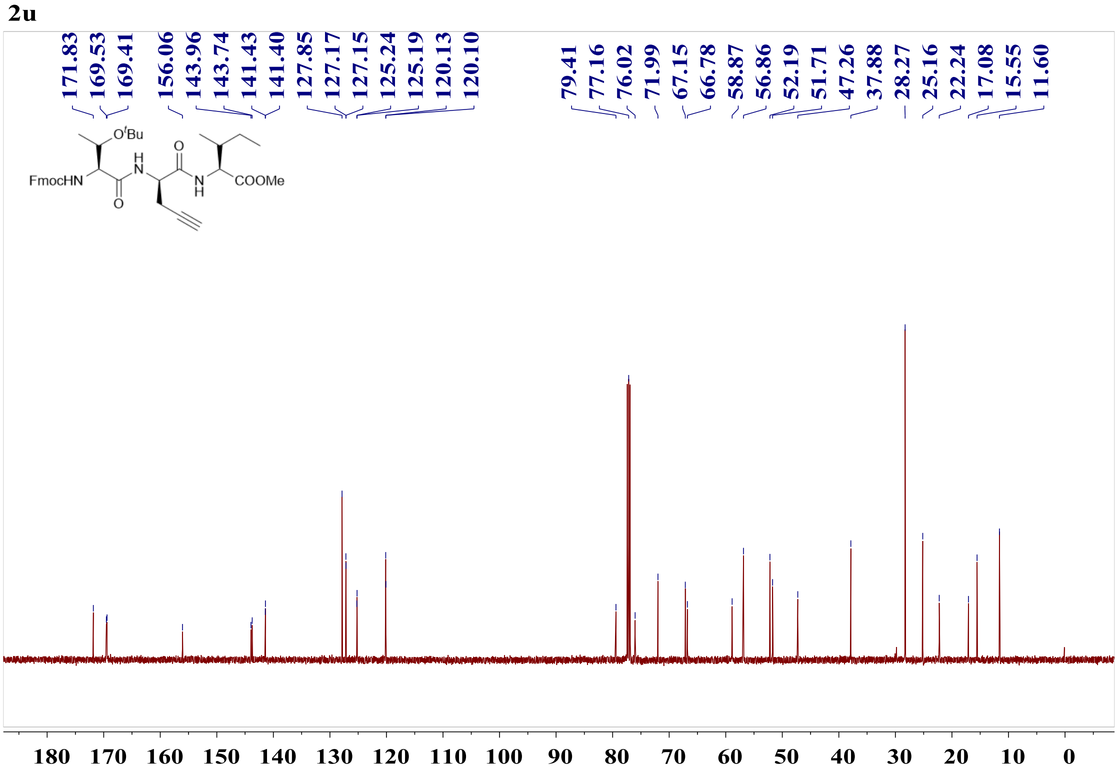

Supplementary Figure 131. ^{1}H NMR spectrum of compound **2v** (500 MHz, $CDCl_3$, 25 ^{o}C)

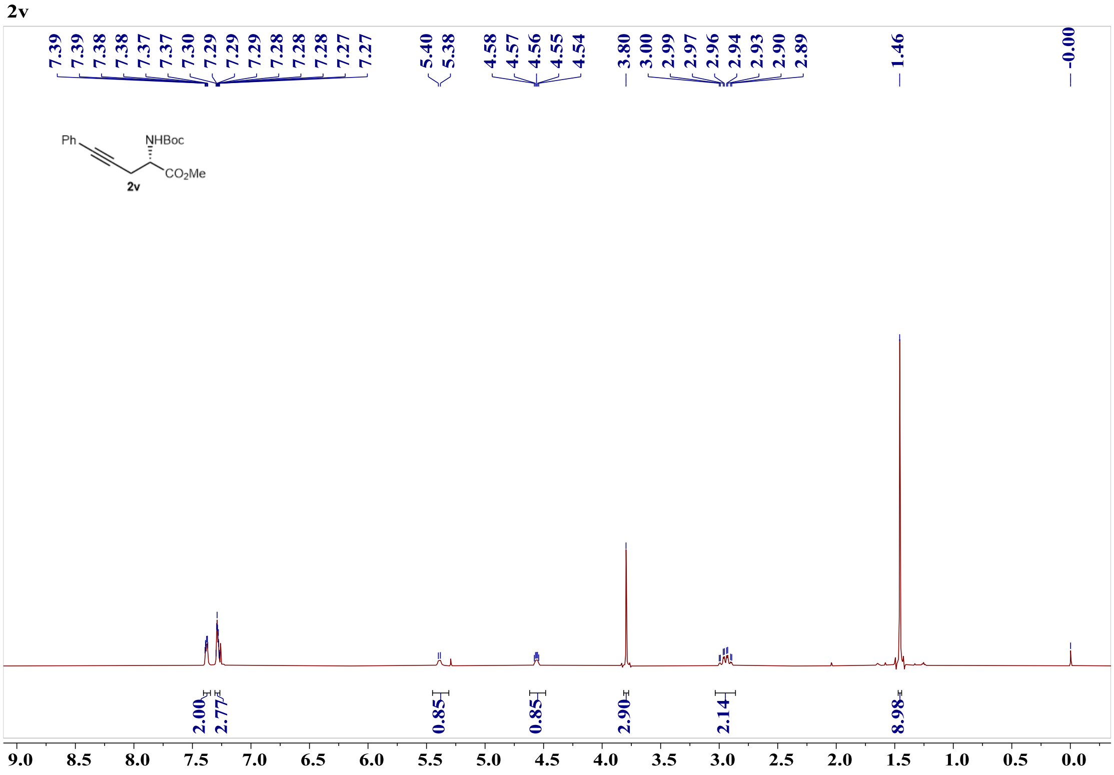

Supplementary Figure 132. ^{1}H NMR spectrum of compound **3a-1** (500 MHz, $CDCl_3$, 25 °C)

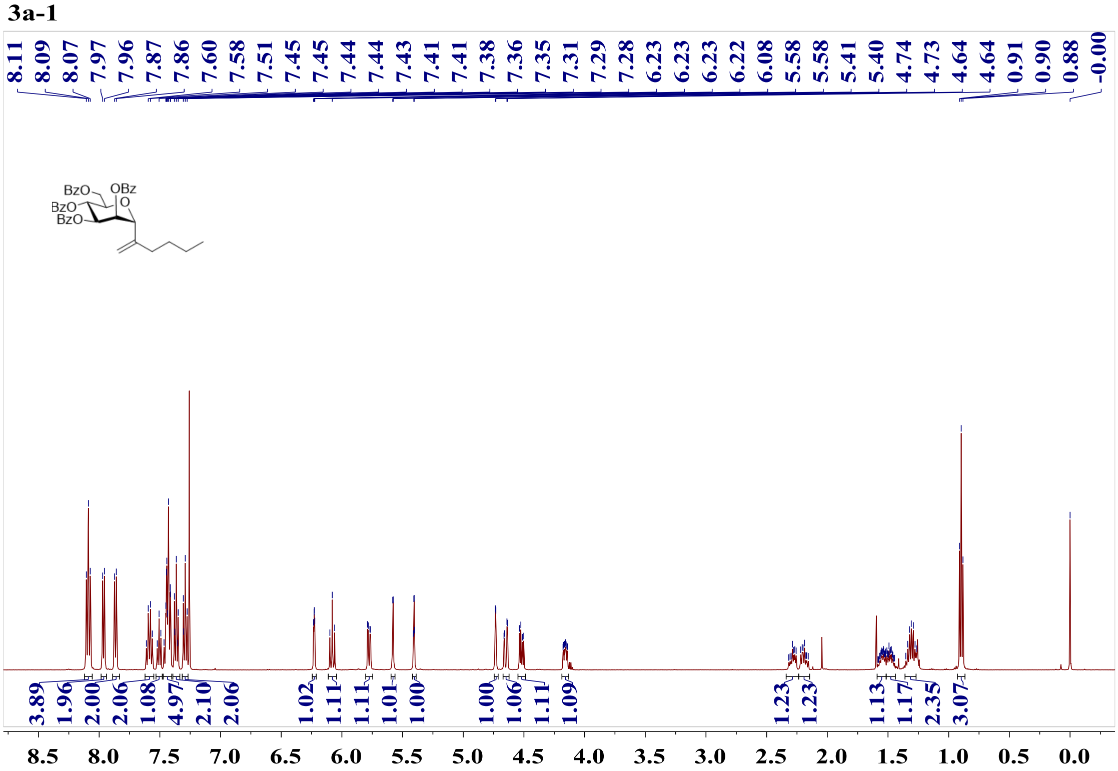

Supplementary Figure 133. ^{13}C NMR spectrum of compound **3a-1** (125 MHz, $CDCl_3$, 25 °C)

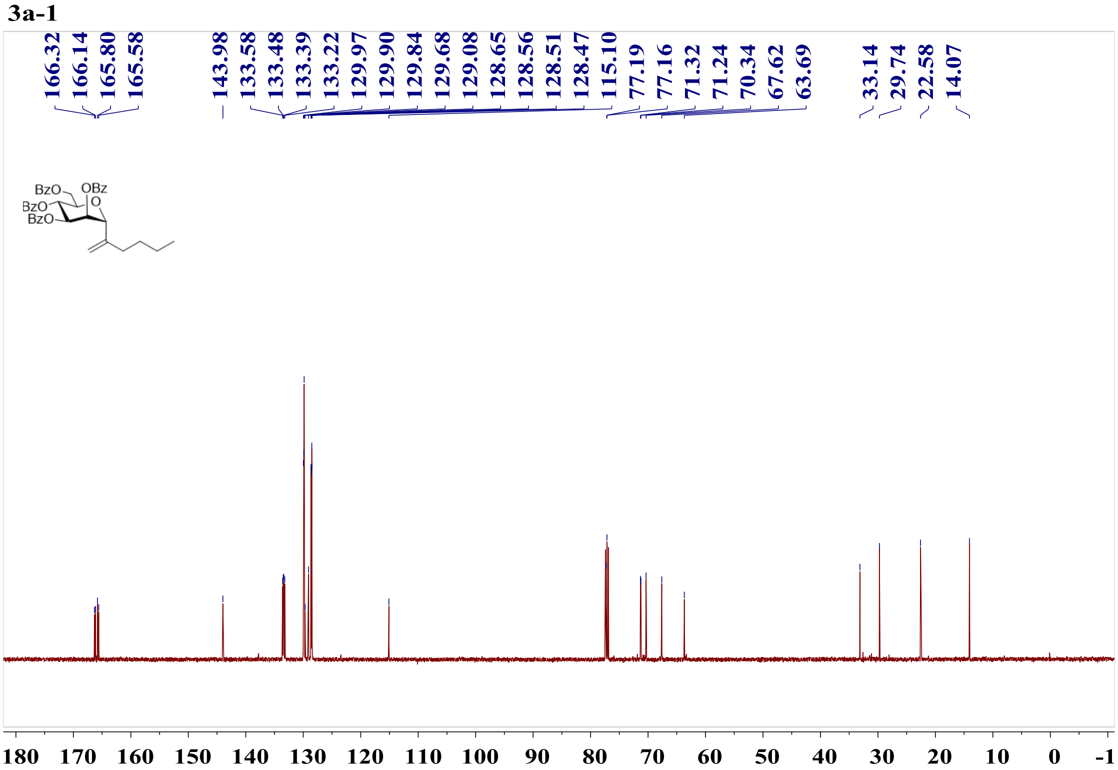

Supplementary Figure 134. ^{1}H NMR spectrum of compound **4a** (500 MHz, $CDCl_3$, 25 °C)

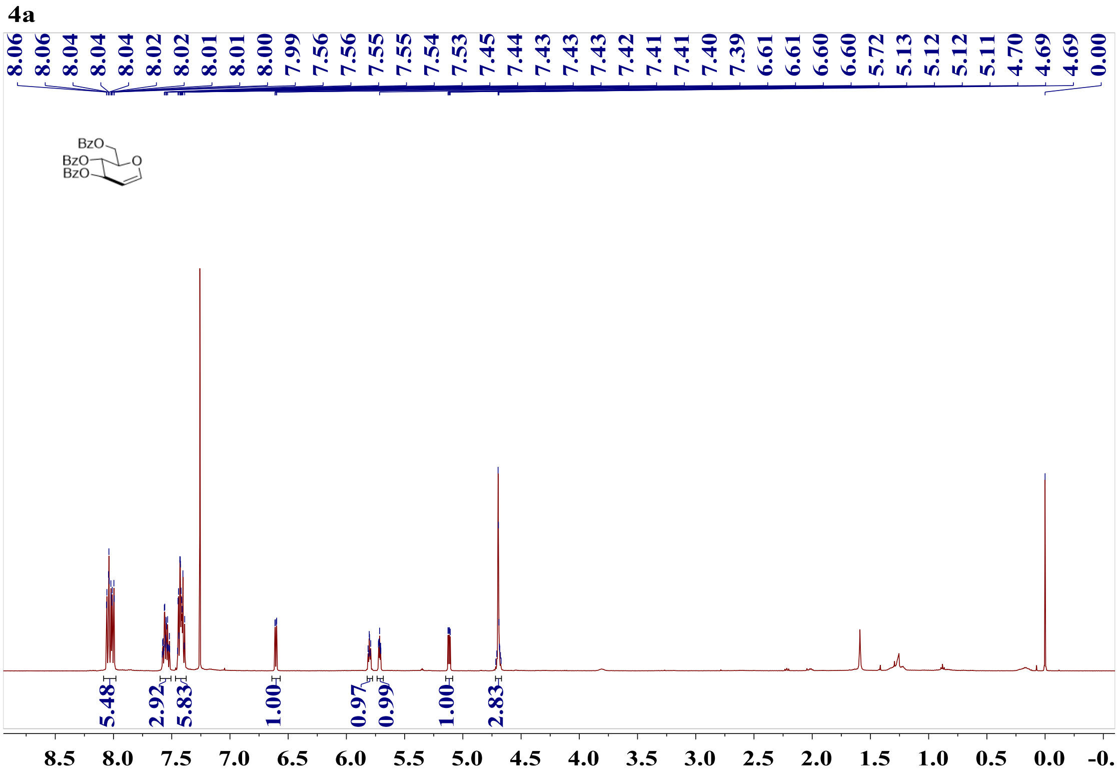

Supplementary Figure 135. ^{1}H NMR spectrum of compound **3b-1** (500 MHz, $CDCl_3$, 25 °C)

3b-1

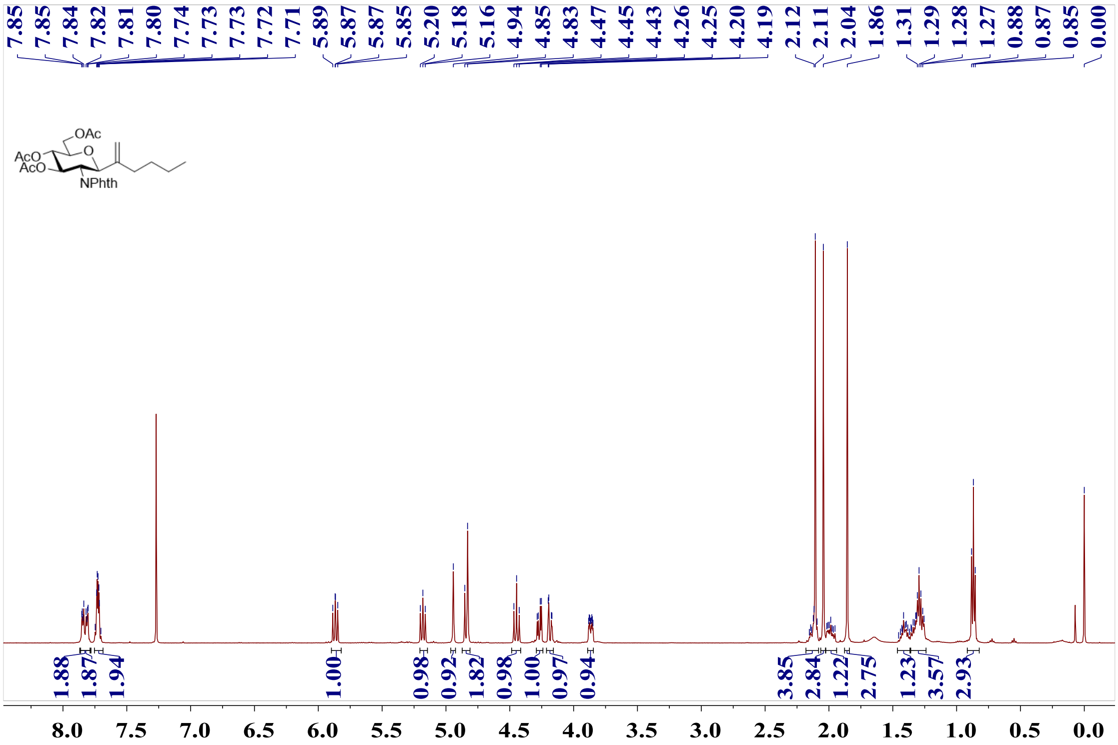

Supplementary Figure 136. ^{13}C NMR spectrum of compound **3b-1** (125 MHz, $CDCl_3$, 25 °C)

3b-1

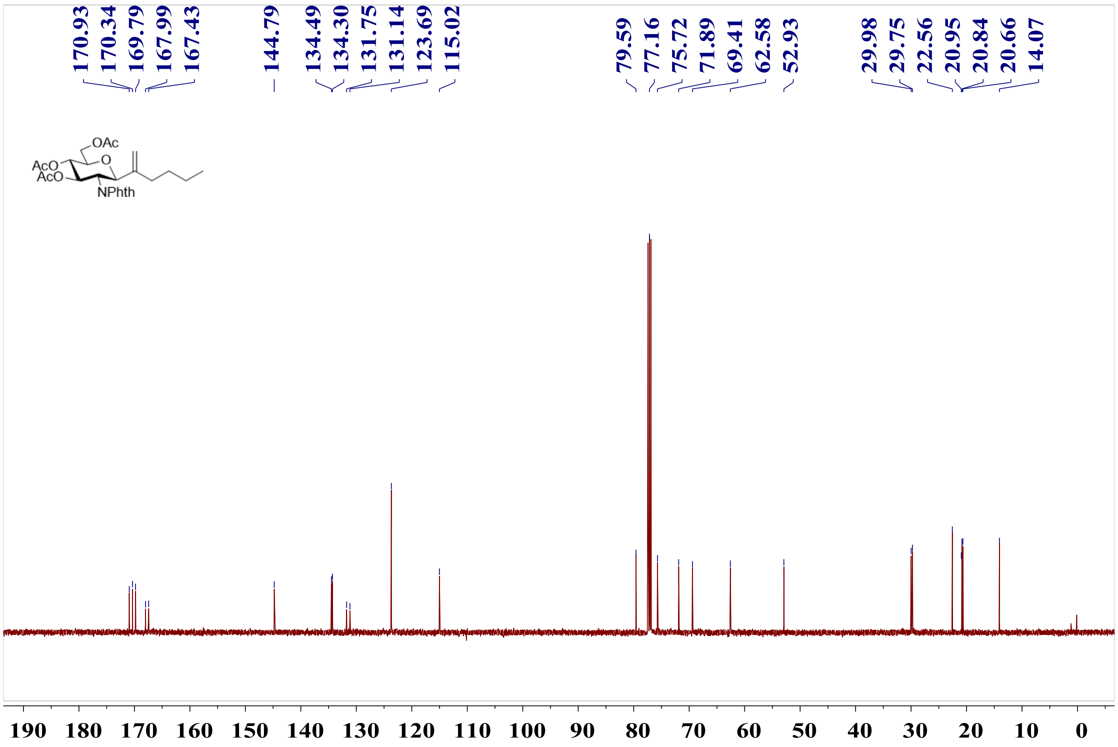

Supplementary Figure 137. ^{1}H NMR spectrum of compound **5a** (500 MHz, $CDCl_3$, 25 °C)

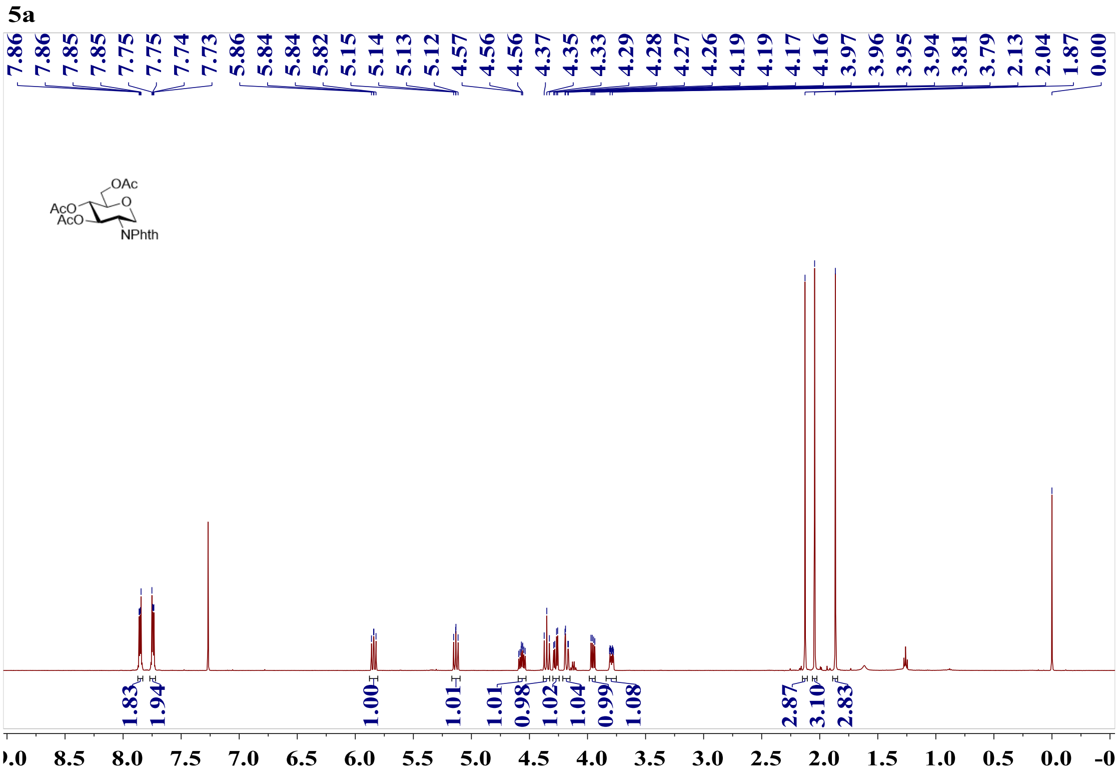

Supplementary Figure 138. ^{1}H NMR spectrum of compound **3b-2** (500 MHz, $CDCl_3$, 25 °C)

3b-2

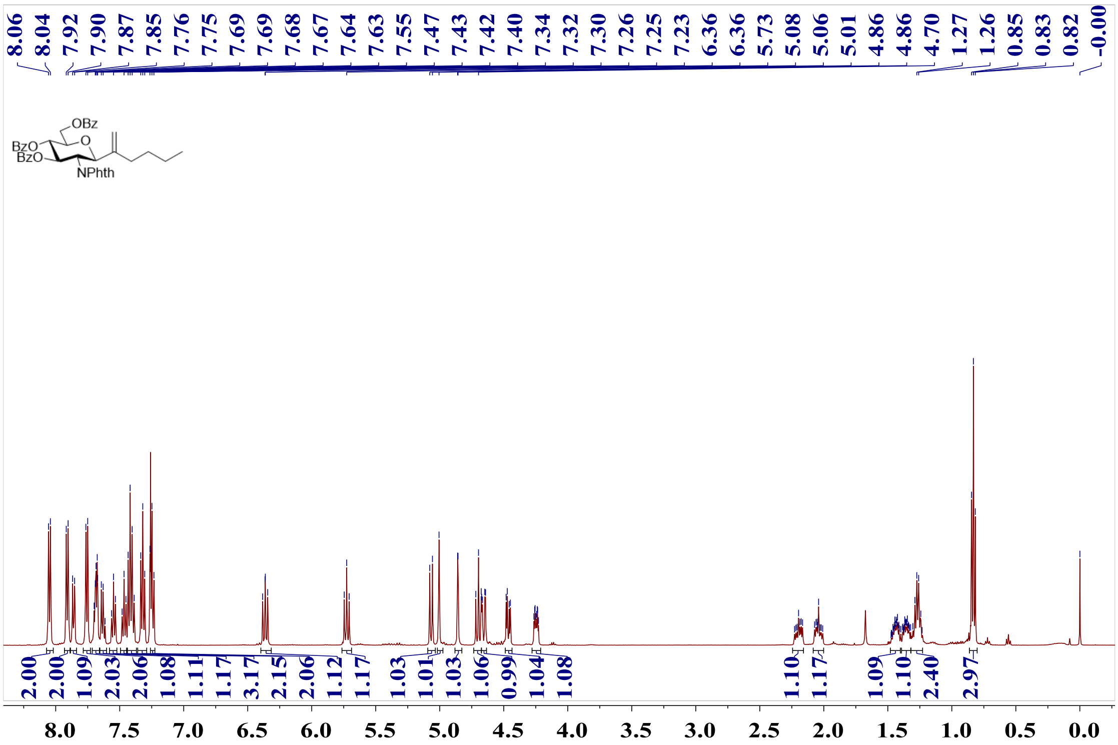

Supplementary Figure 139. ^{13}C NMR spectrum of compound **3b-2** (125 MHz, $CDCl_3$, 25 °C)

3b-2

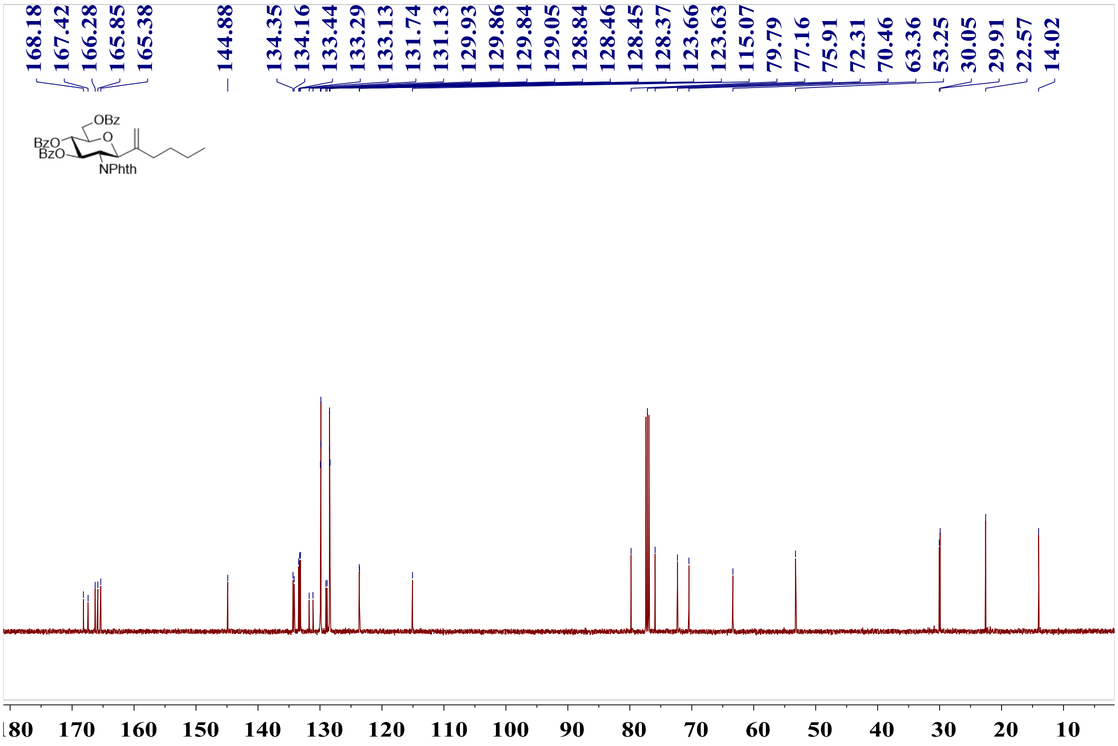

Supplementary Figure 140. ^{1}H NMR spectrum of compound **4b** (500 MHz, $CDCl_3$, 25 °C)

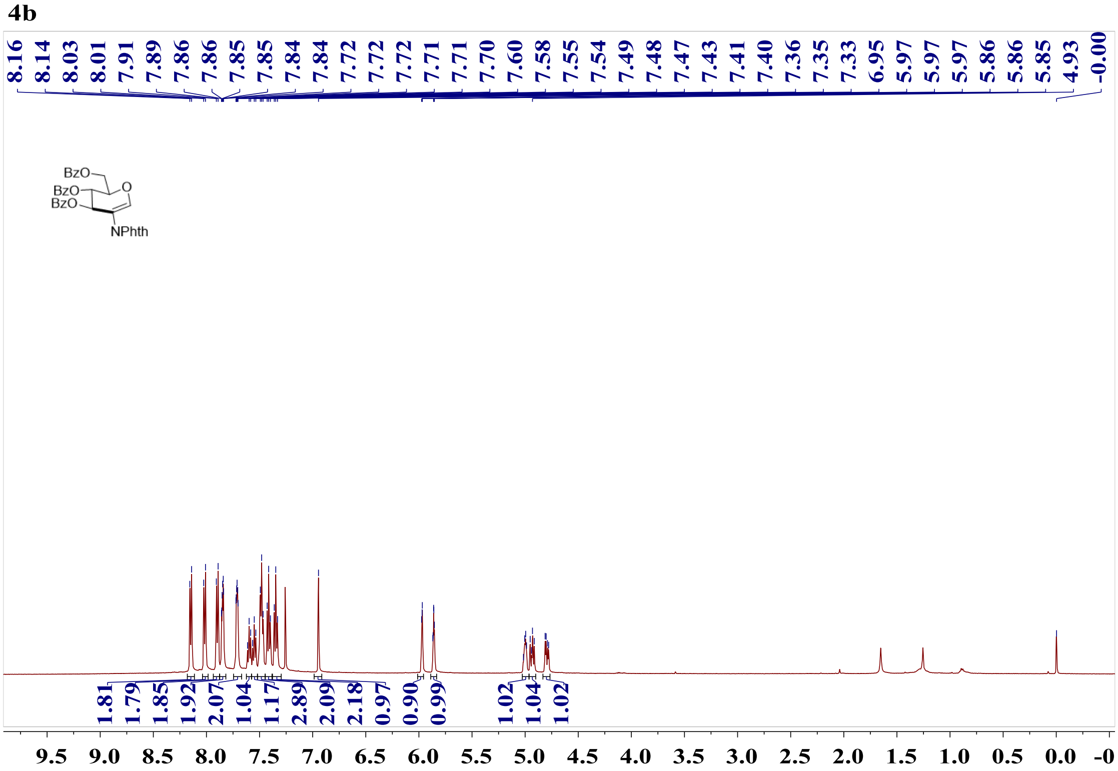

Supplementary Figure 141. ^{13}C NMR spectrum of compound **4b** (125 MHz, $CDCl_3$, 25 °C)

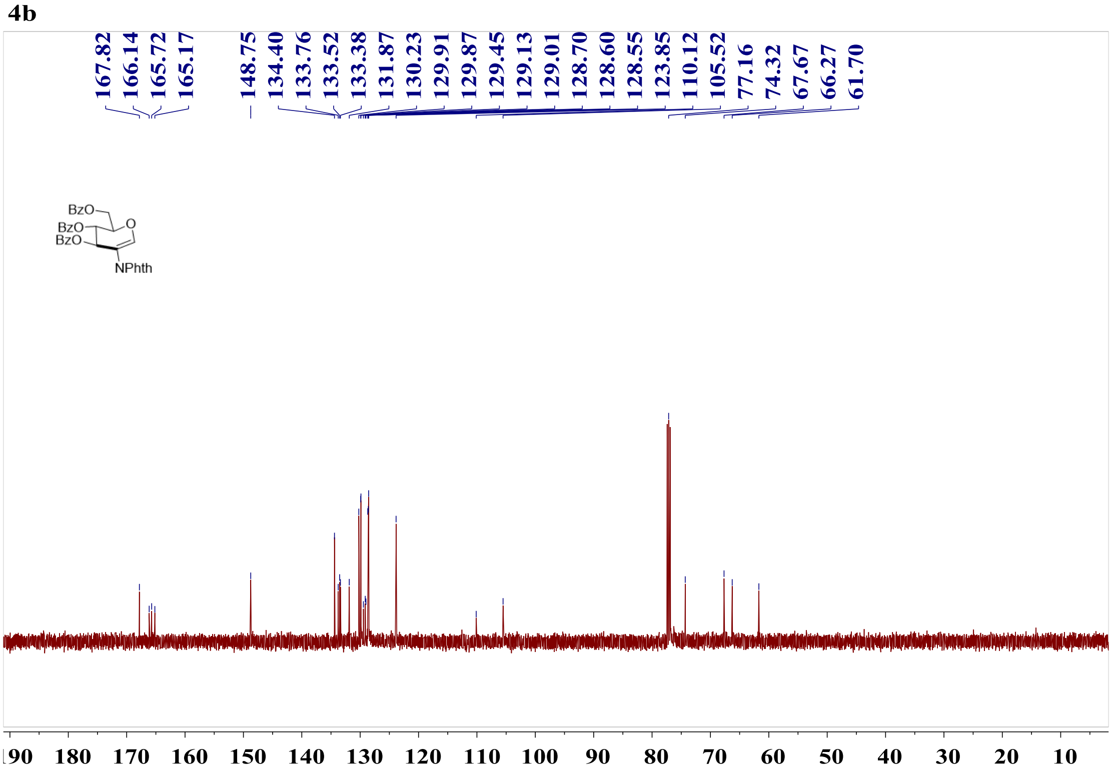

Supplementary Figure 142. ^{1}H NMR spectrum of compound **5b** (500 MHz, $CDCl_3$, 25 °C)

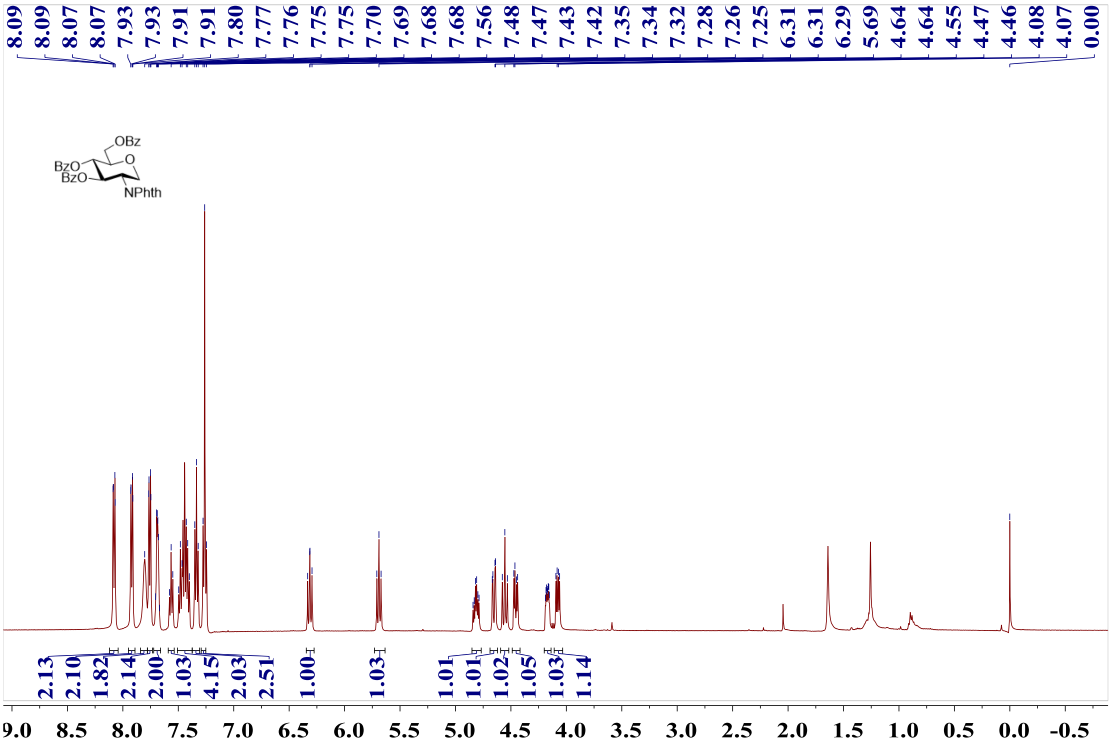

Supplementary Figure 143. ^{13}C NMR spectrum of compound **5b** (125 MHz, $CDCl_3$, 25 °C)

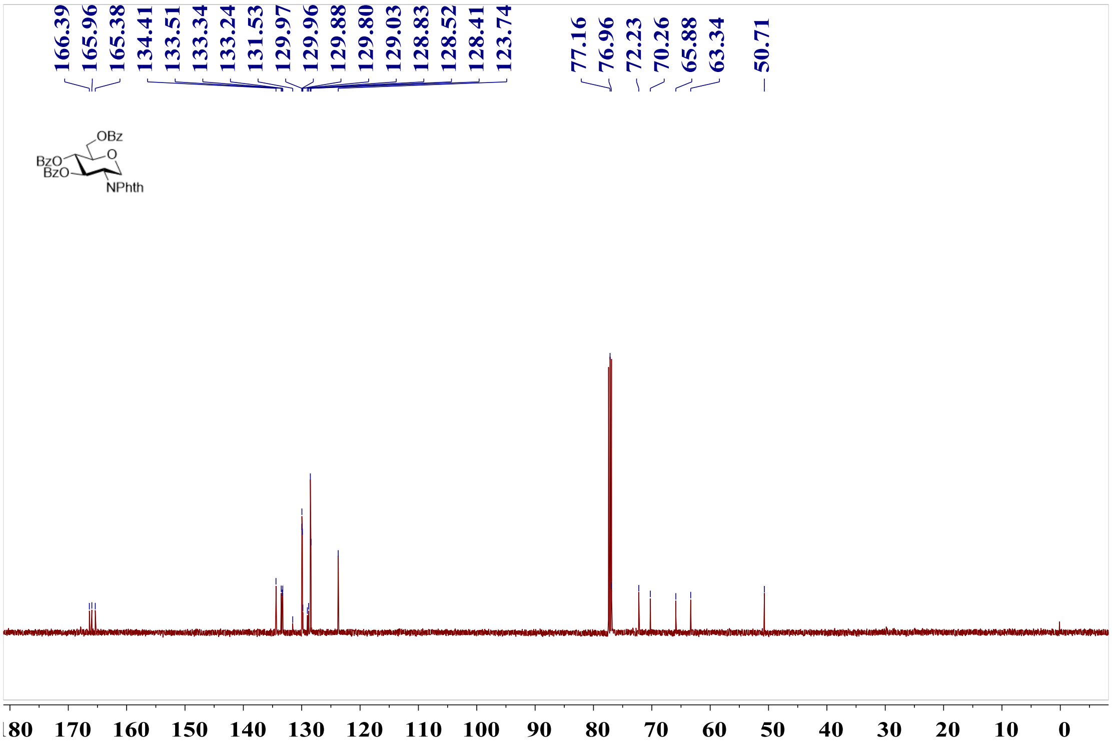

Supplementary Figure 144. ^{1}H NMR spectrum of (R)-Tol-BINAP(O)$_2$ (500 MHz, $CDCl_3$, 25 °C)

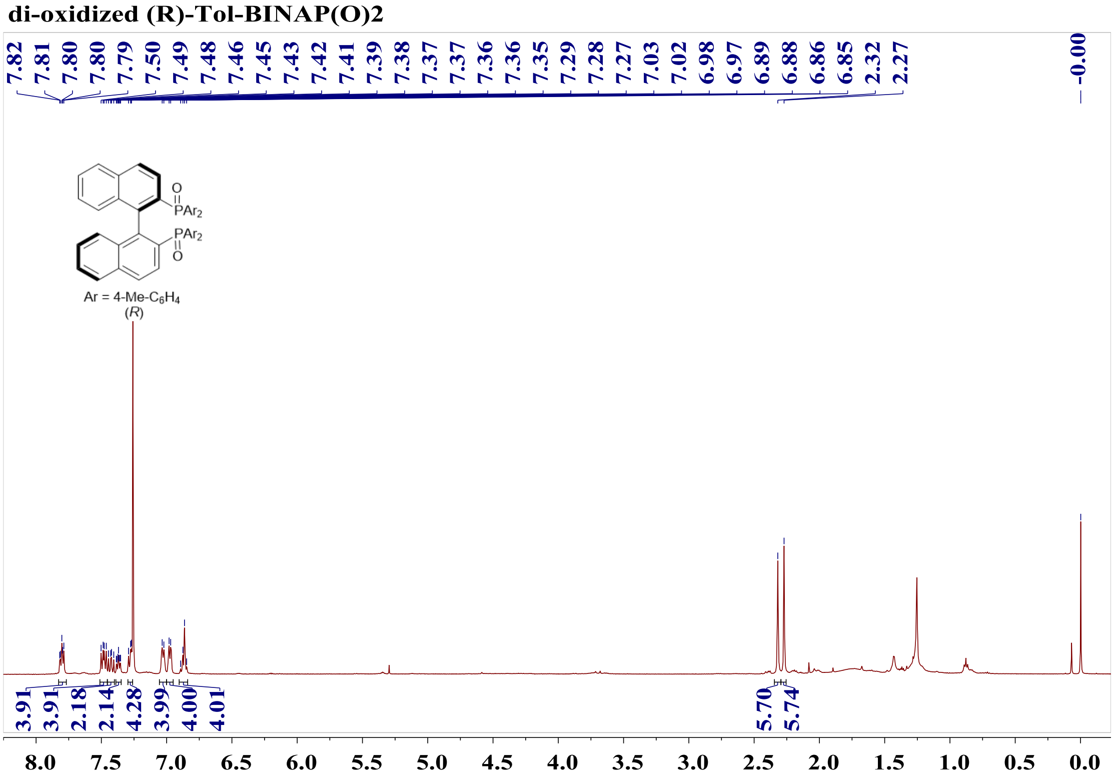

Supplementary Figure 145. ^{31}P NMR spectrum of (R)-Tol-BINAP(O)$_2$ (162 MHz, $CDCl_3$, 25 °C)

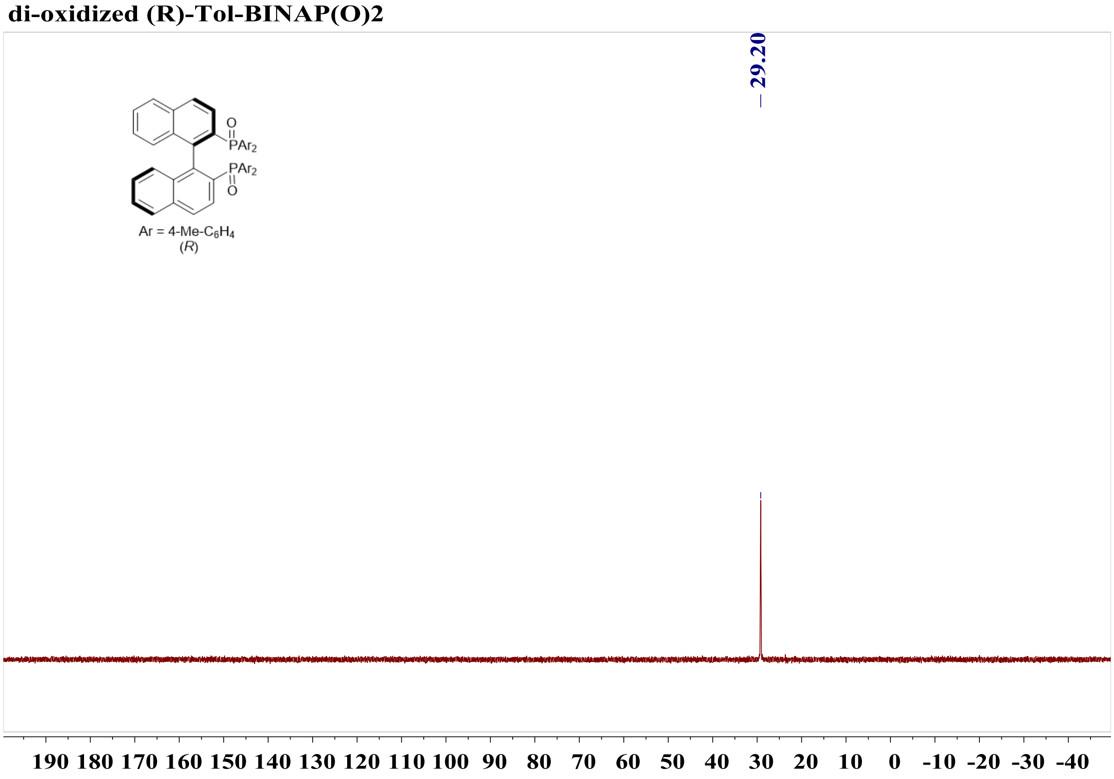

Supplementary Figure 146. ^{1}H NMR spectrum of compound **3aa** (500 MHz, $CDCl_3$, 25 °C)

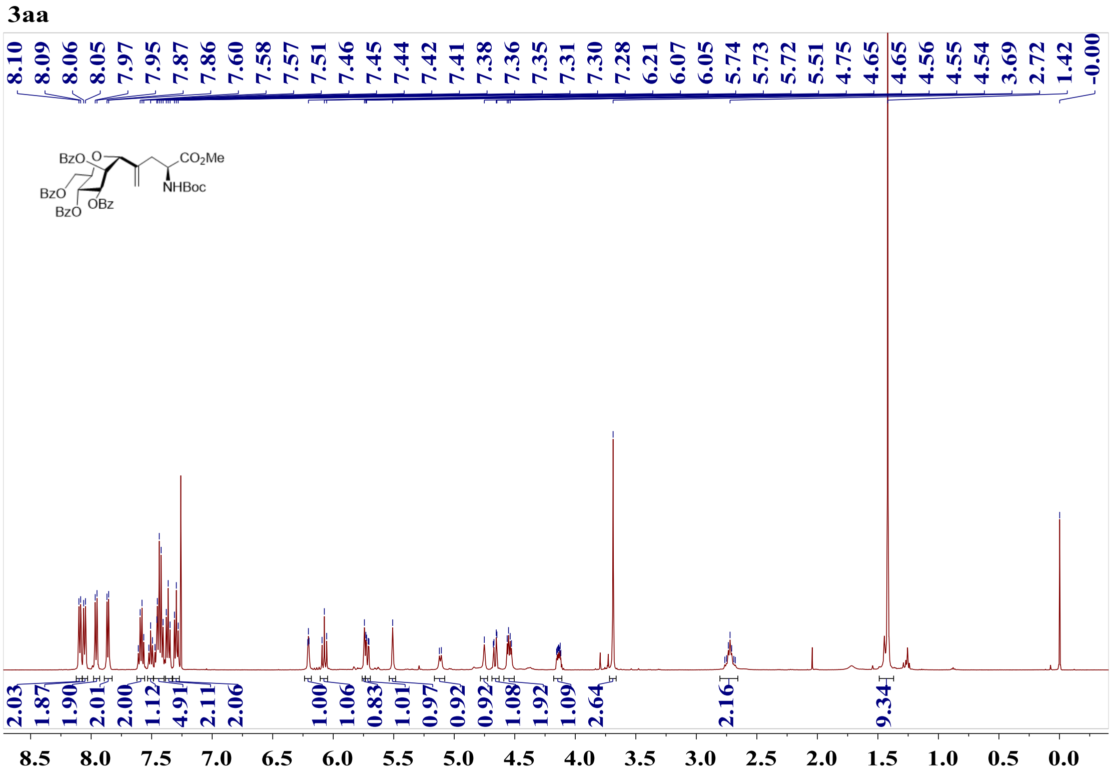

Supplementary Figure 147. ^{13}C NMR spectrum of compound **3aa** (125 MHz, $CDCl_3$, 25 °C)

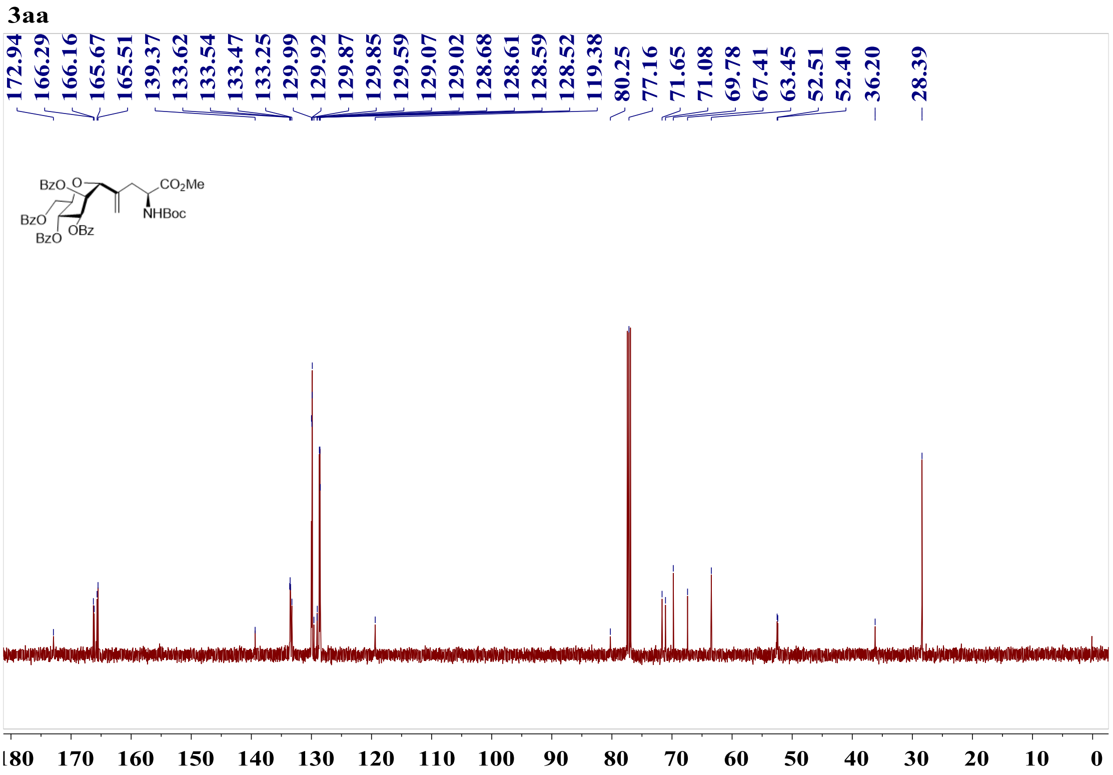

Supplementary Figure 148. ^{1}H NMR spectrum of compound **3ab** (500 MHz, $CDCl_3$, 25 °C)

3ab

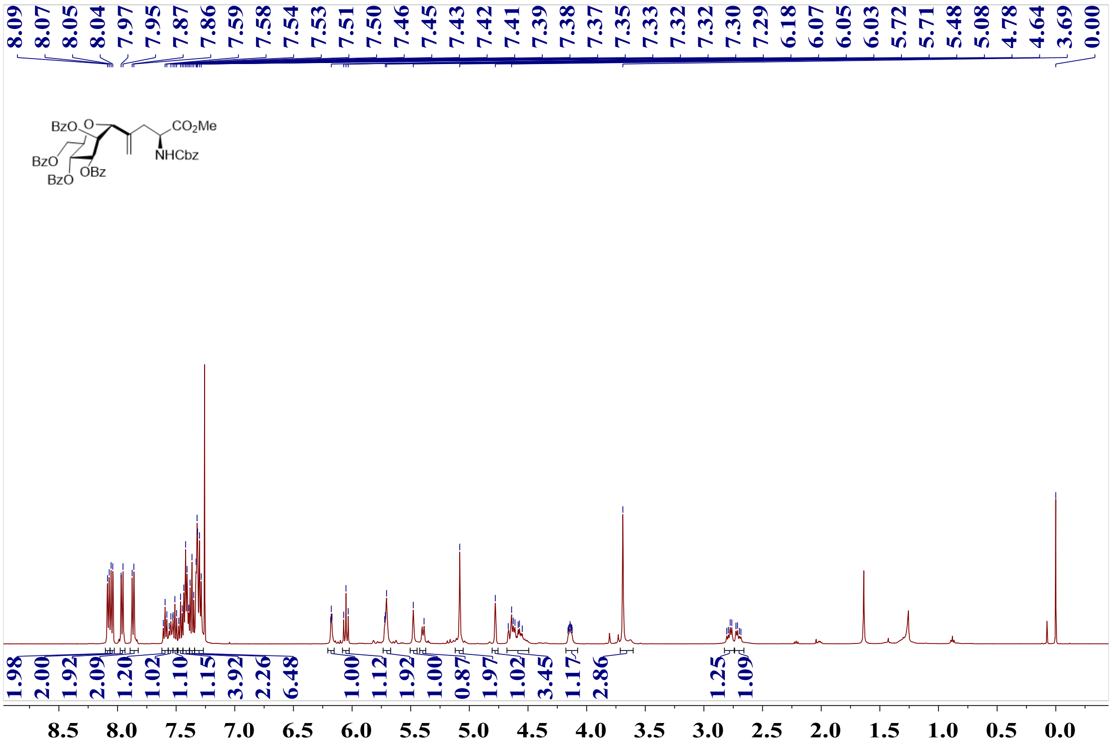

Supplementary Figure 149. ^{13}C NMR spectrum of compound **3ab** (125 MHz, $CDCl_3$, 25 °C)

3ab

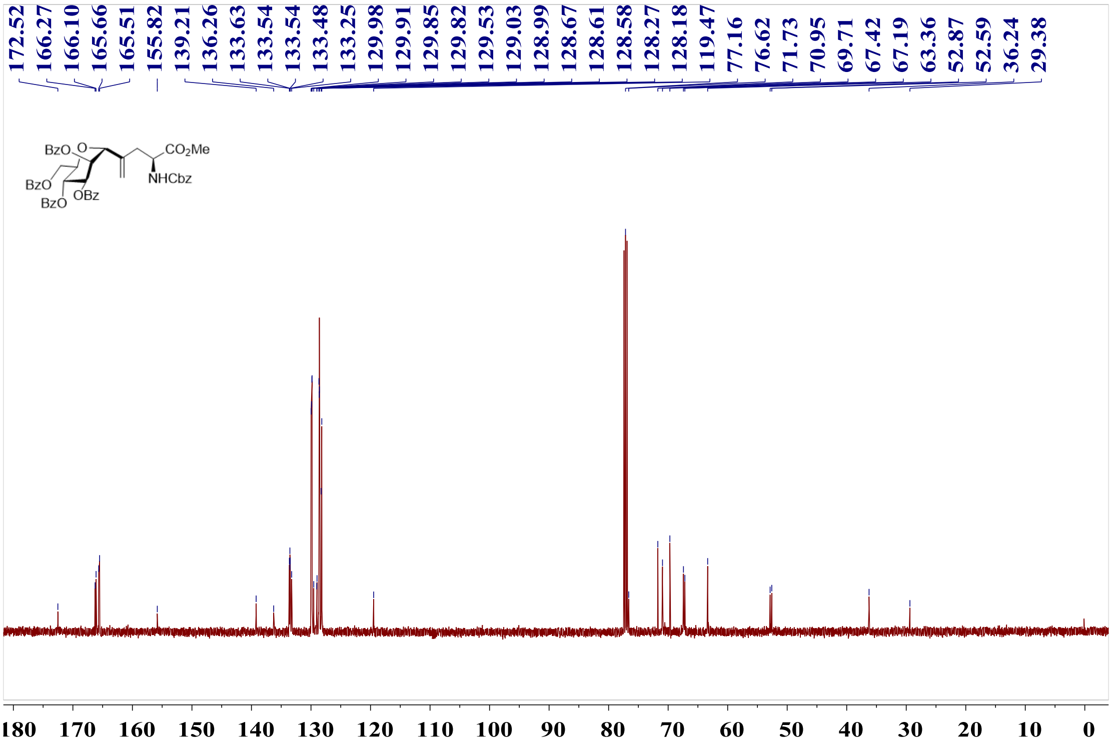

Supplementary Figure 150. ^{1}H NMR spectrum of compound **3ac** (500 MHz, $CDCl_3$, 25 °C)

3ac

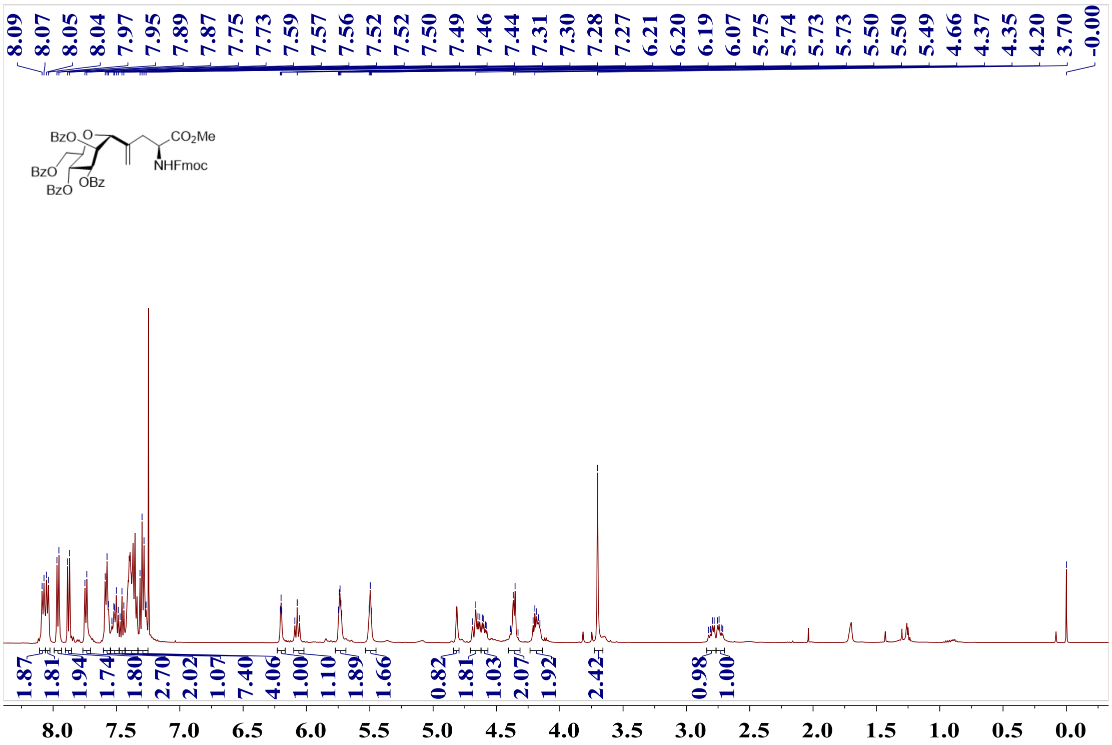

Supplementary Figure 151. ^{13}C NMR spectrum of compound **3ac** (125 MHz, $CDCl_3$, 25 °C)

3ac

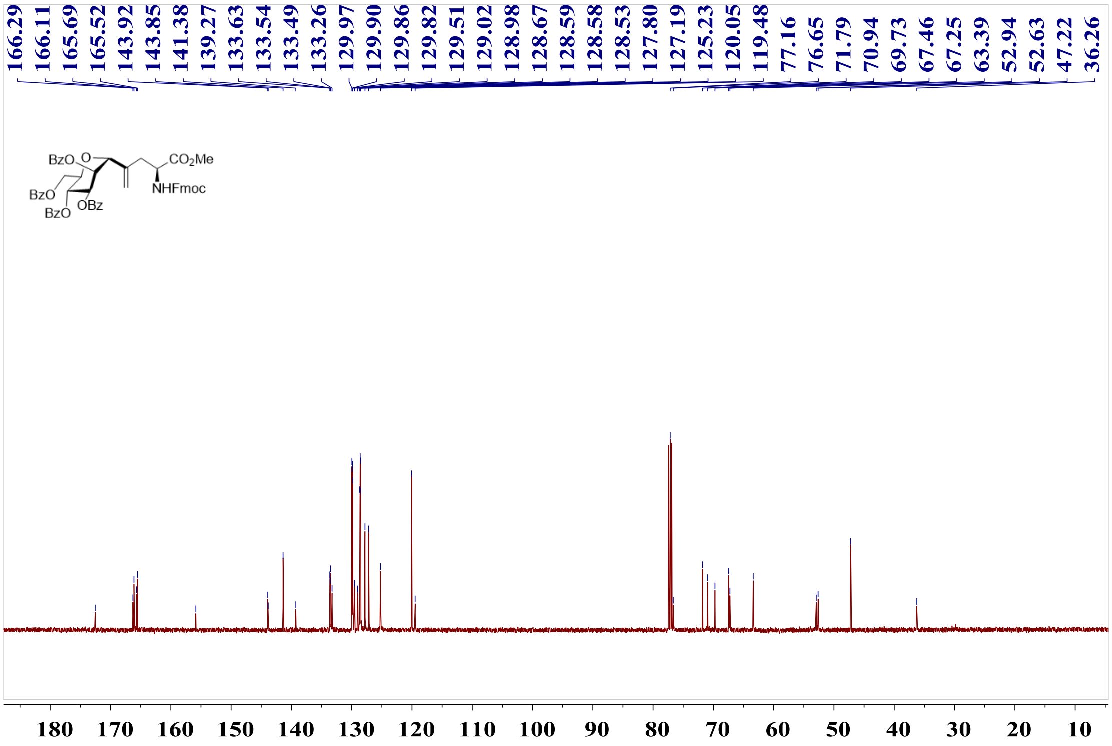

Supplementary Figure 152. ^{1}H NMR spectrum of compound **3ad** (500 MHz, $CDCl_3$, 25 °C)

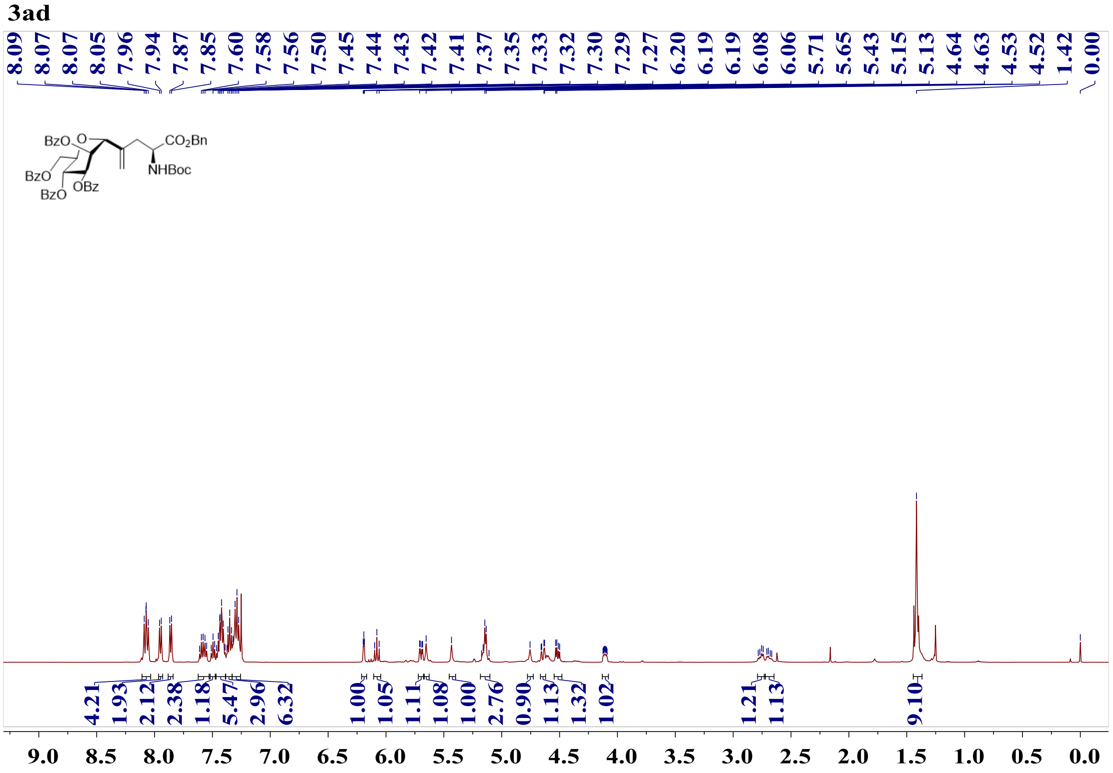

Supplementary Figure 153. ^{13}C NMR spectrum of compound **3ad** (125 MHz, $CDCl_3$, 25 °C)

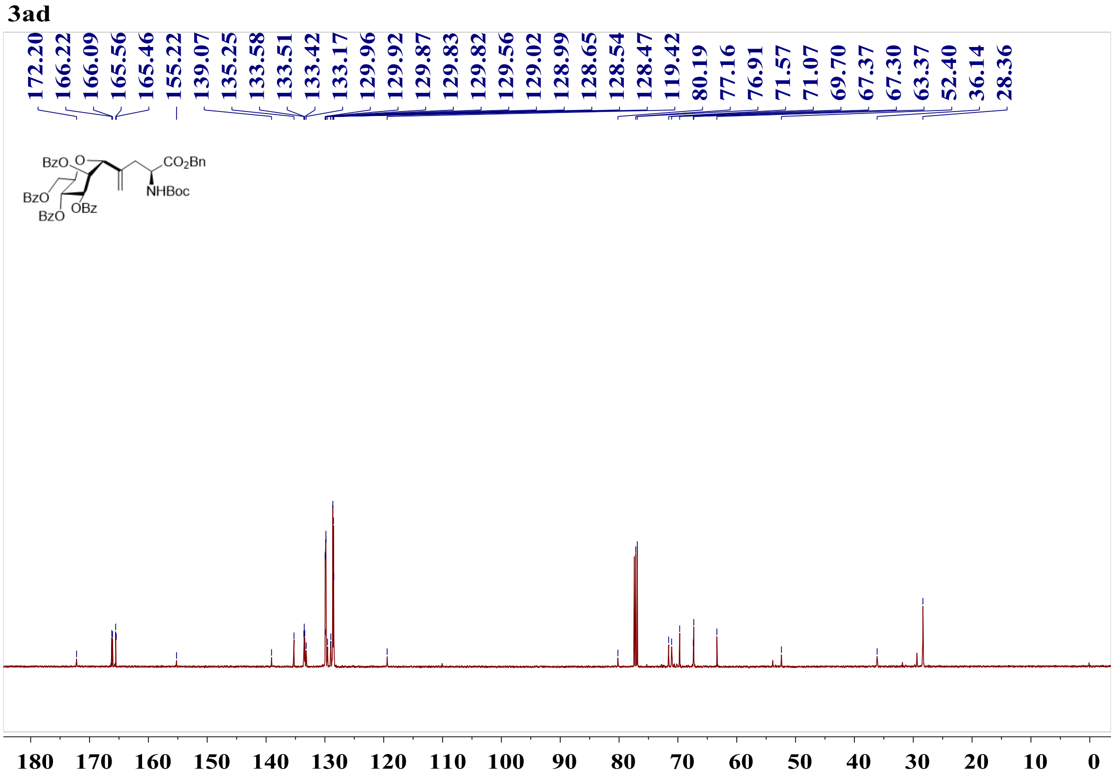

Supplementary Figure 154. ^{1}H NMR spectrum of compound **3ae** (500 MHz, $CDCl_3$, 25 ^{o}C)

3ae

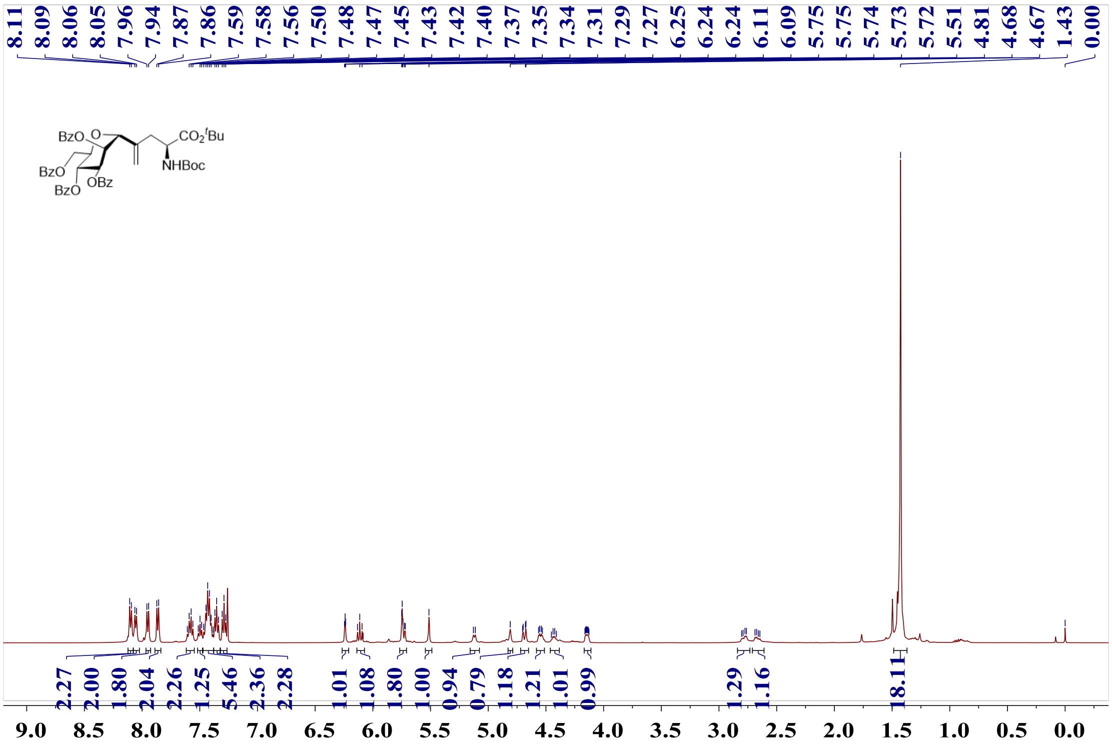

Supplementary Figure 155. ^{13}C NMR spectrum of compound **3ae** (125 MHz, $CDCl_3$, 25 ^{o}C)

3ae

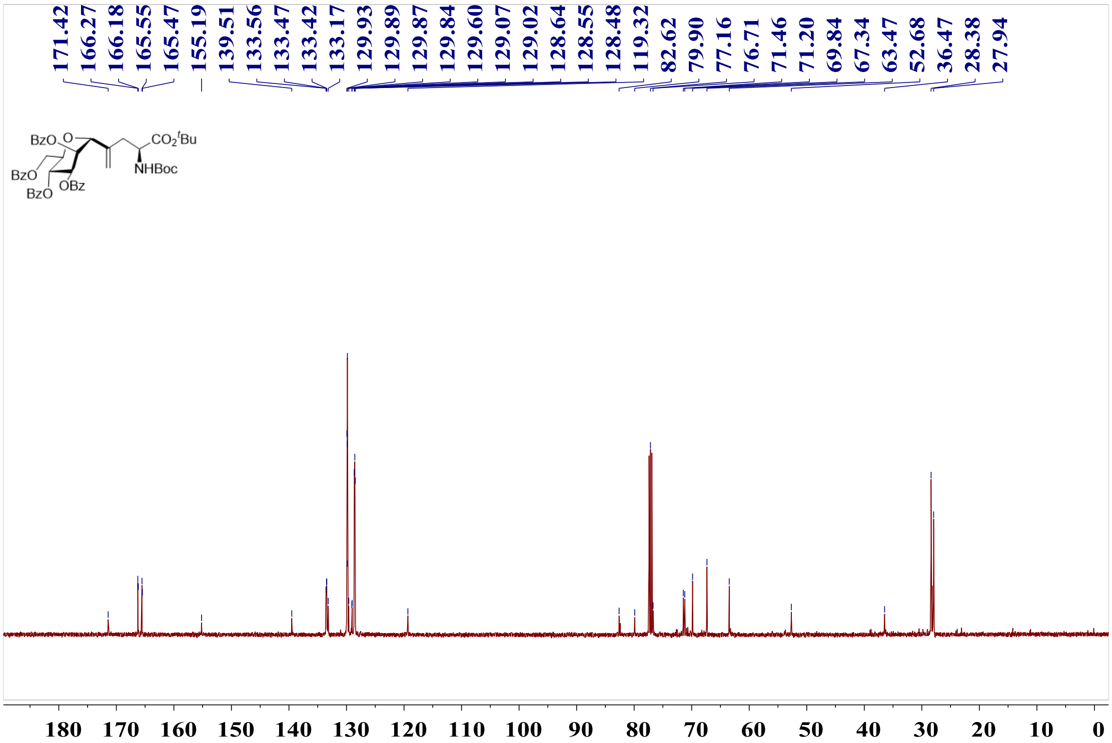

Supplementary Figure 156. ^{1}H NMR spectrum of compound **3af** (500 MHz, $CDCl_3$, 25 ^{o}C)

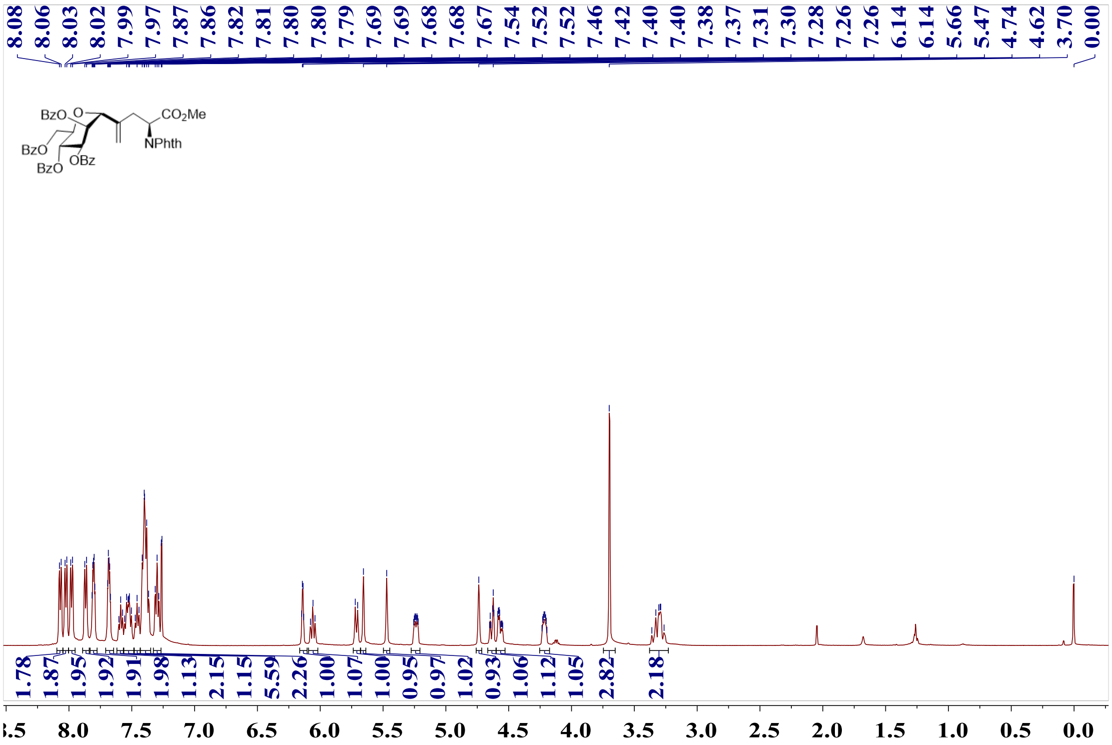

Supplementary Figure 157. ^{13}C NMR spectrum of compound **3af** (125 MHz, $CDCl_3$, 25 ^{o}C)

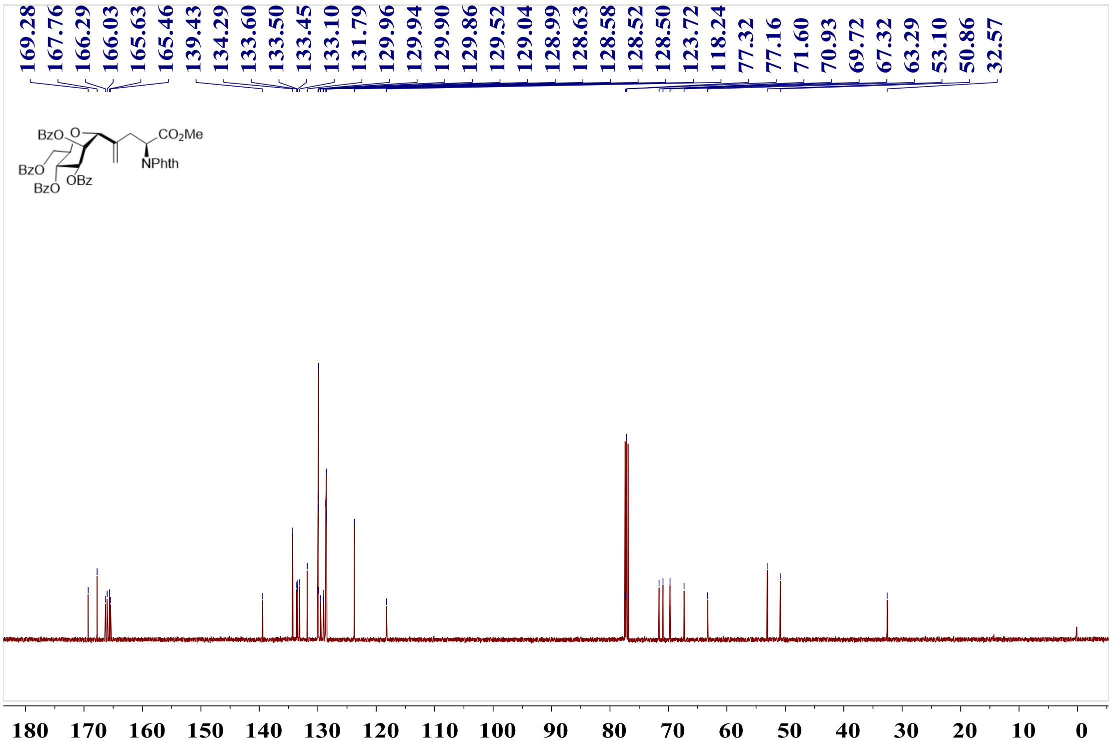

Supplementary Figure 158. ^{1}H NMR spectrum of compound **3ag** (500 MHz, $CDCl_3$, 25 ^{o}C)

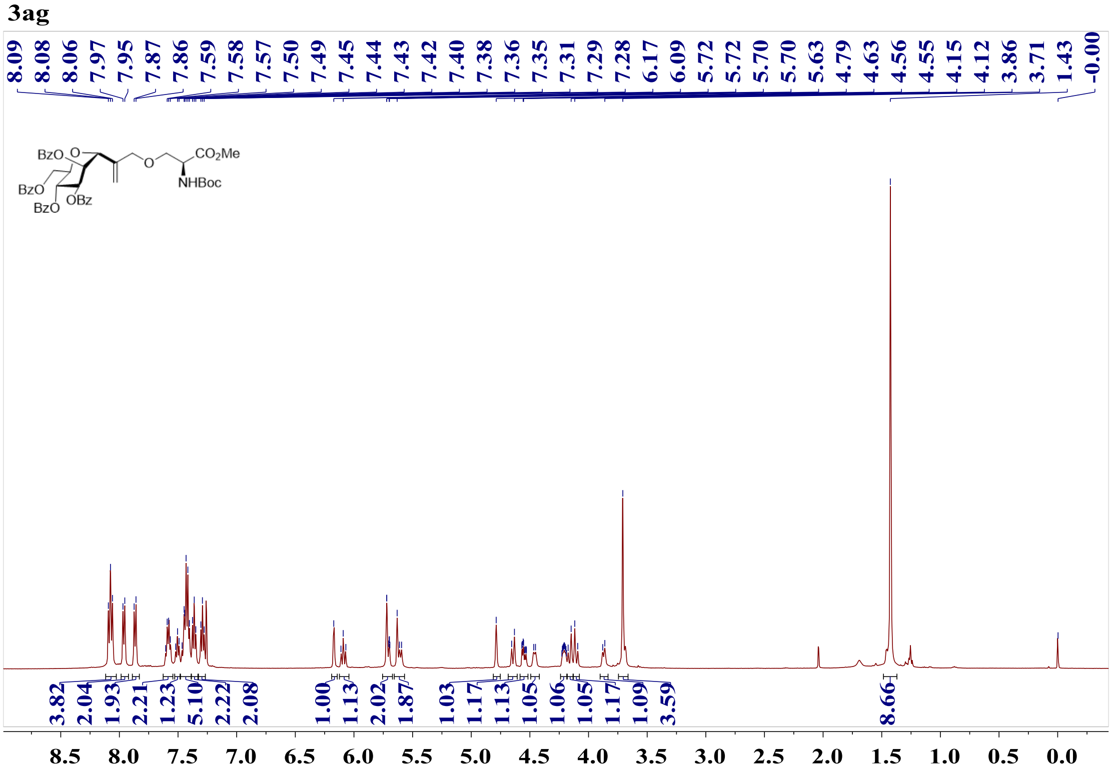

Supplementary Figure 159. ^{13}C NMR spectrum of compound **3ag** (125 MHz, $CDCl_3$, 25 ^{o}C)

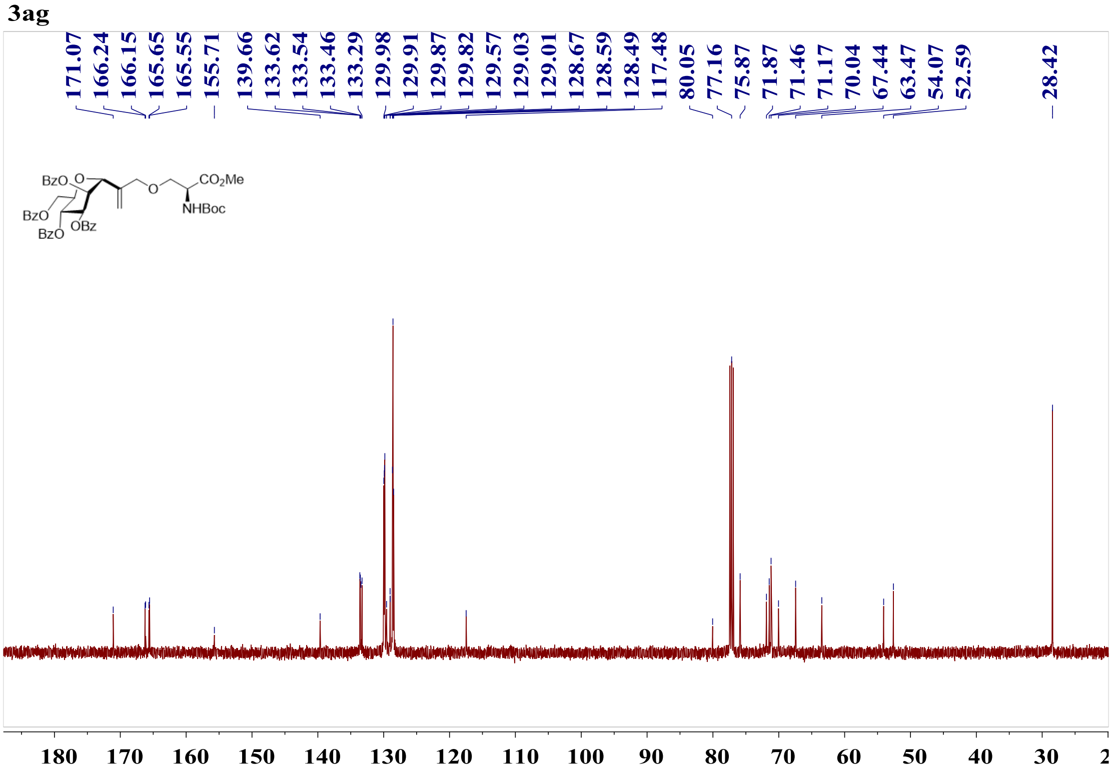

Supplementary Figure 160. ^{1}H NMR spectrum of compound **3ah** (500 MHz, $CDCl_3$, 25 °C)

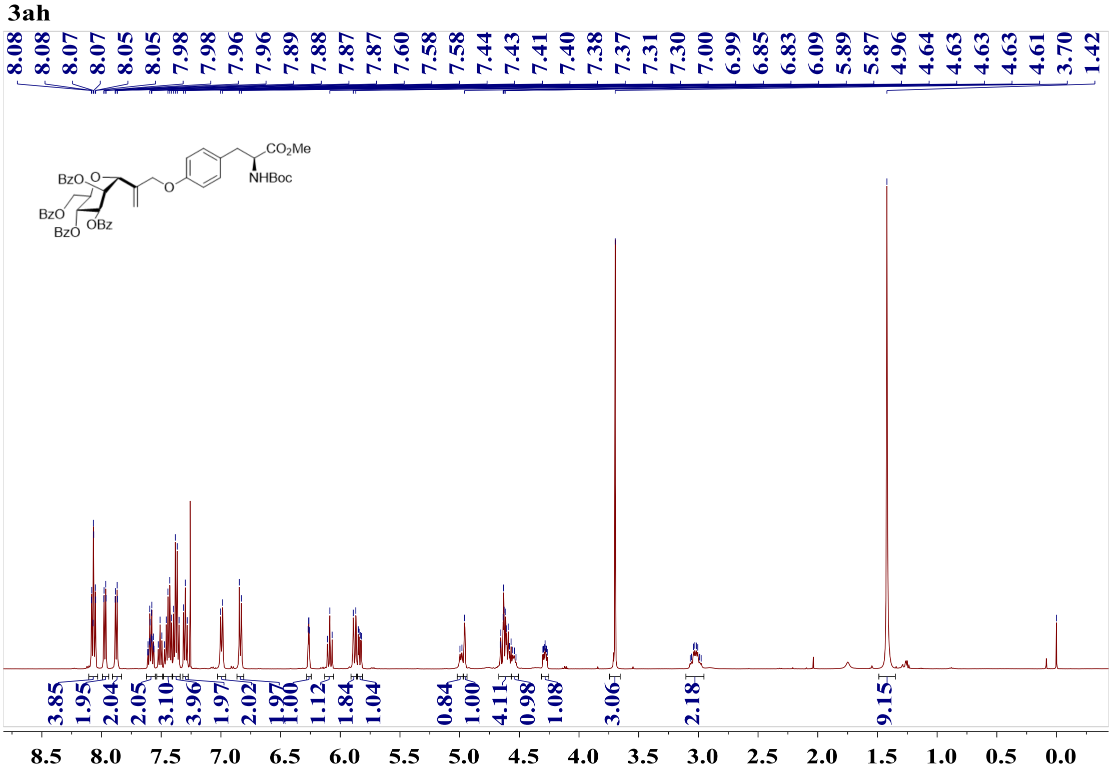

Supplementary Figure 161. ^{13}C NMR spectrum of compound **3ah** (125 MHz, $CDCl_3$, 25 °C)

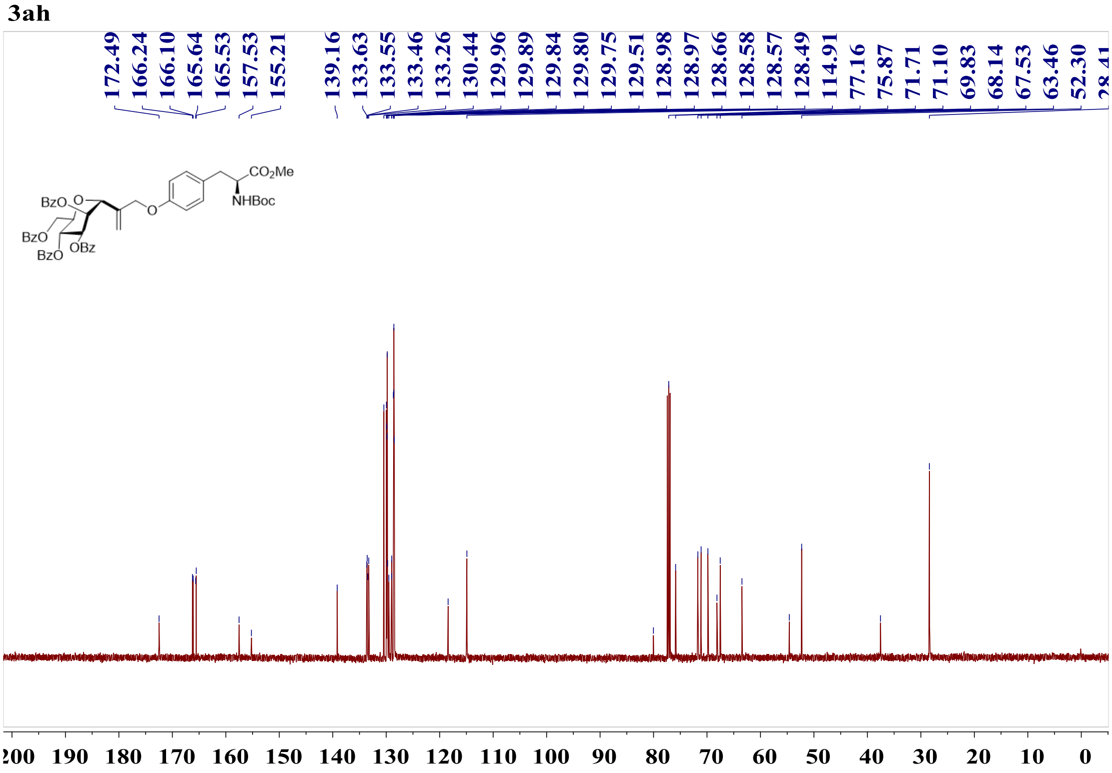

Supplementary Figure 162. ^{1}H NMR spectrum of compound **3ai** (500 MHz, $CDCl_3$, 25 °C)

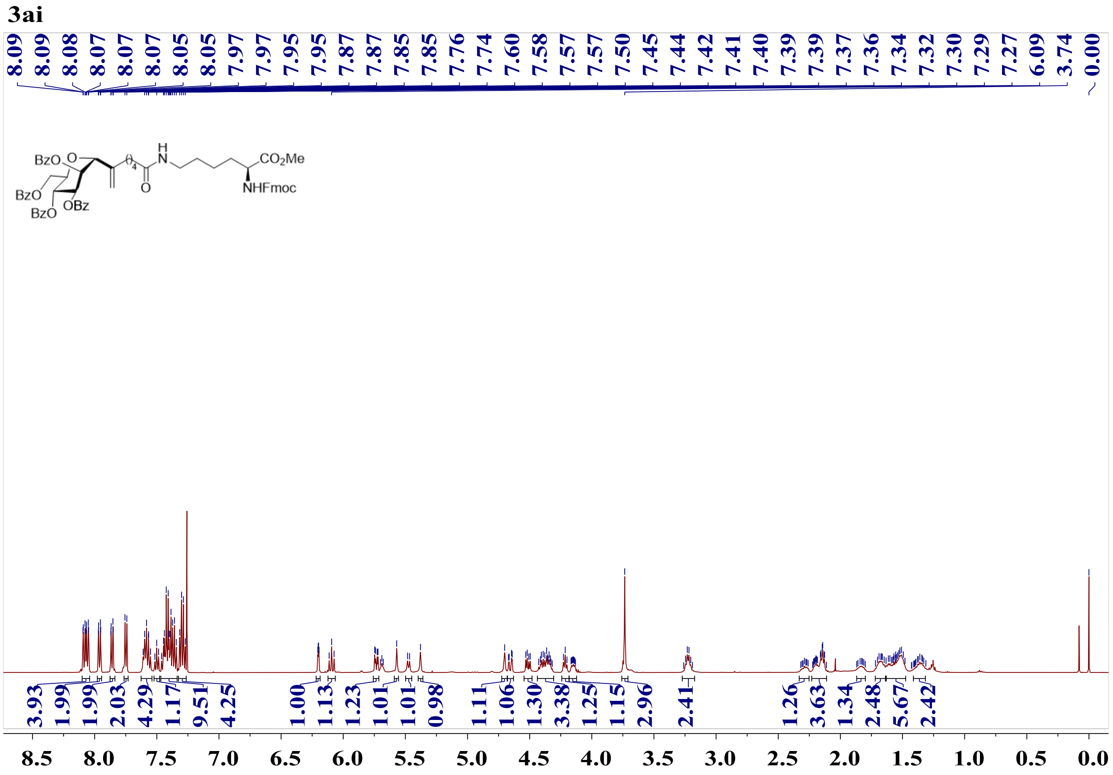

Supplementary Figure 163. ^{13}C NMR spectrum of compound **3ai** (125 MHz, $CDCl_3$, 25 °C)

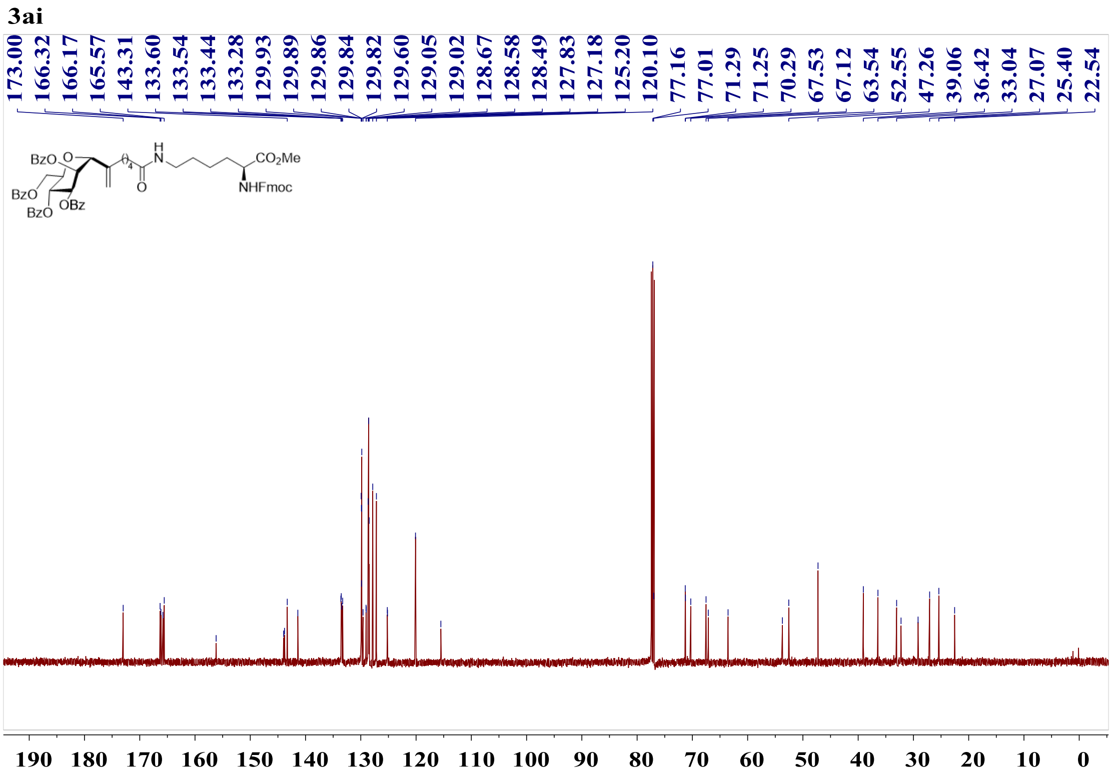

Supplementary Figure 164. ^{1}H NMR spectrum of compound **3aj** (500 MHz, $CDCl_3$, 25 °C)

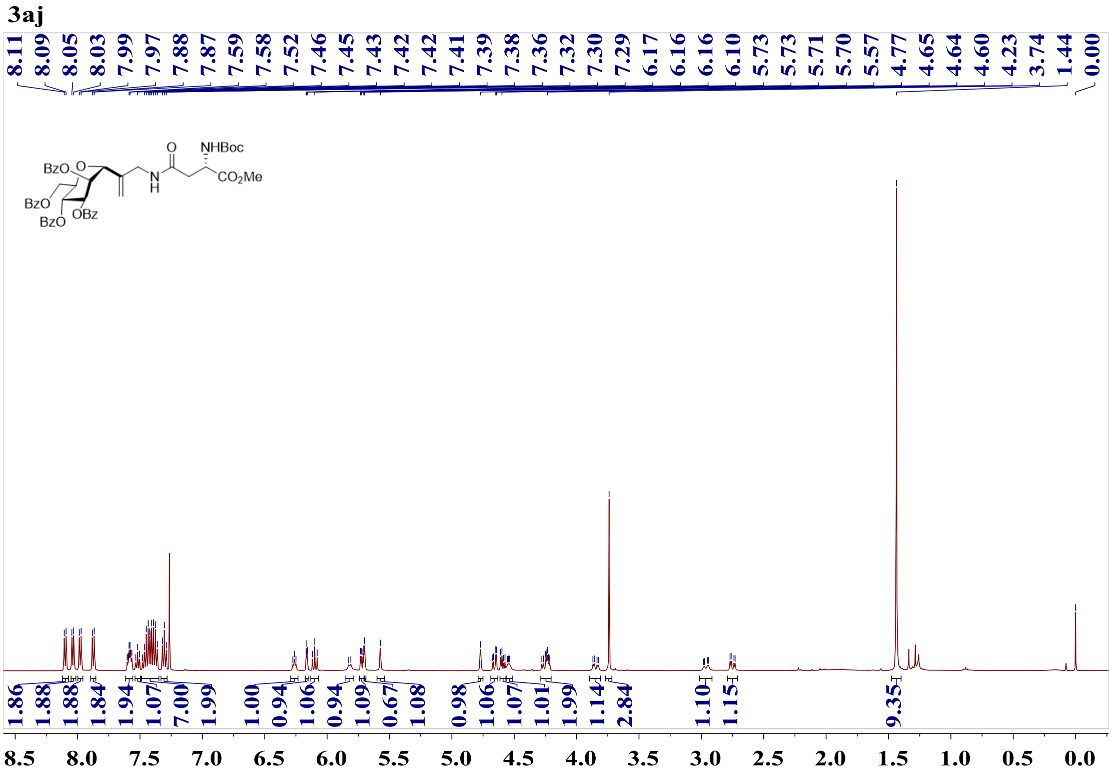

Supplementary Figure 165. ^{13}C NMR spectrum of compound **3aj** (125 MHz, $CDCl_3$, 25 °C)

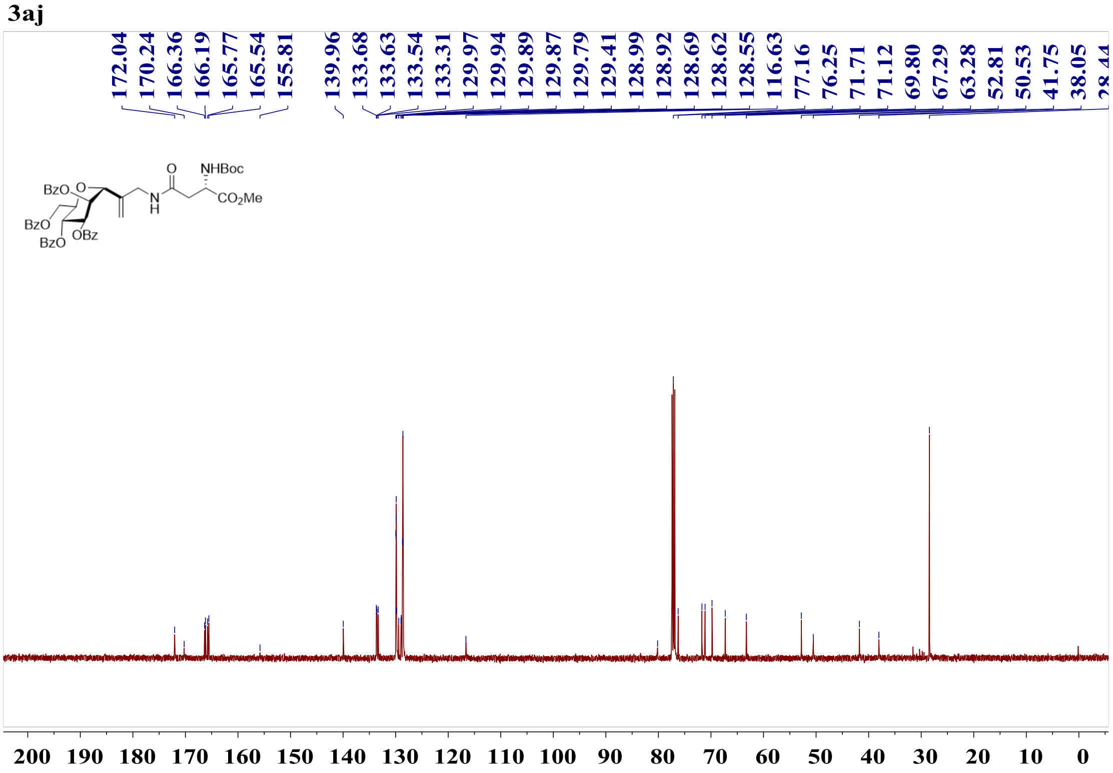

Supplementary Figure 166. ^{1}H NMR spectrum of compound **3ak** (500 MHz, $CDCl_3$, 25 °C)

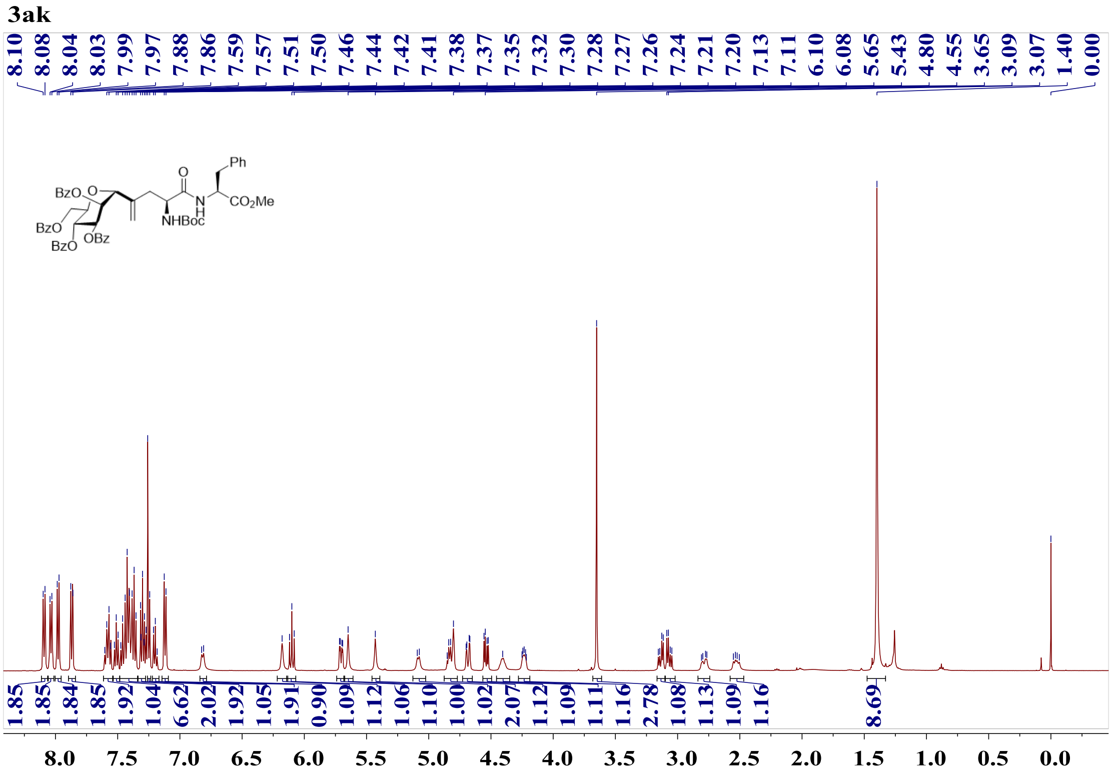

Supplementary Figure 167. ^{13}C NMR spectrum of compound **3ak** (500 MHz, $CDCl_3$, 25 °C)

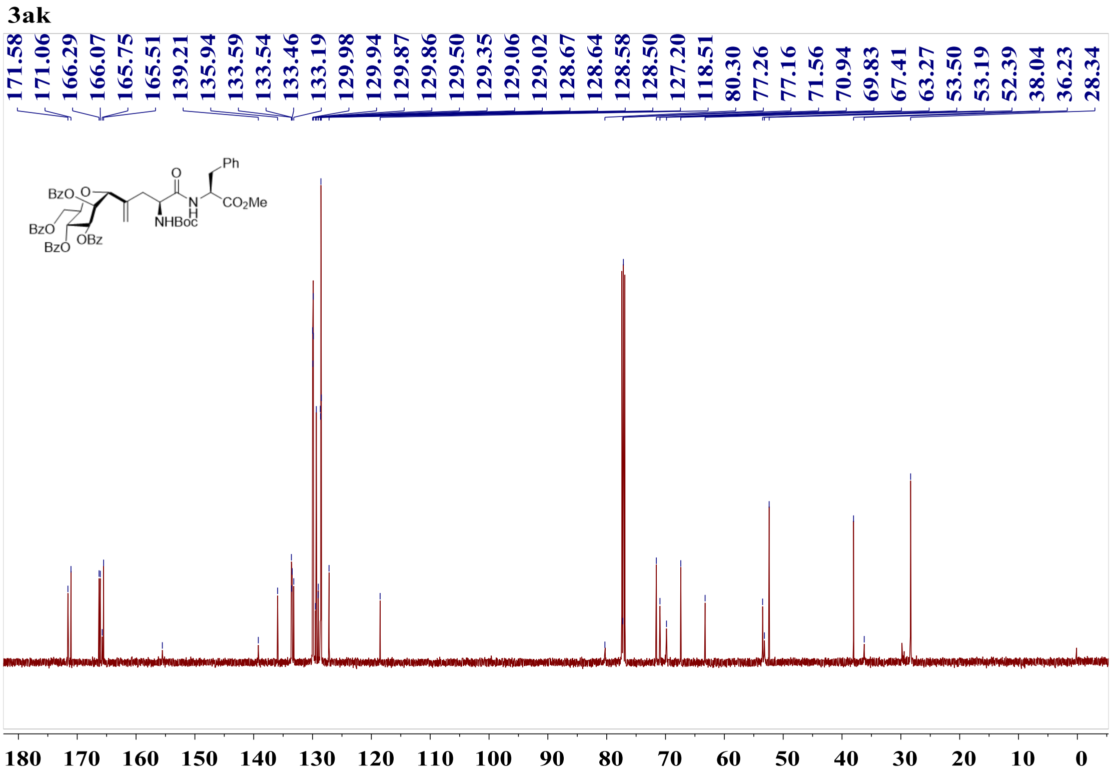

Supplementary Figure 168. ^{1}H NMR spectrum of compound **3al** (500 MHz, $CDCl_3$, 25 °C)

3al

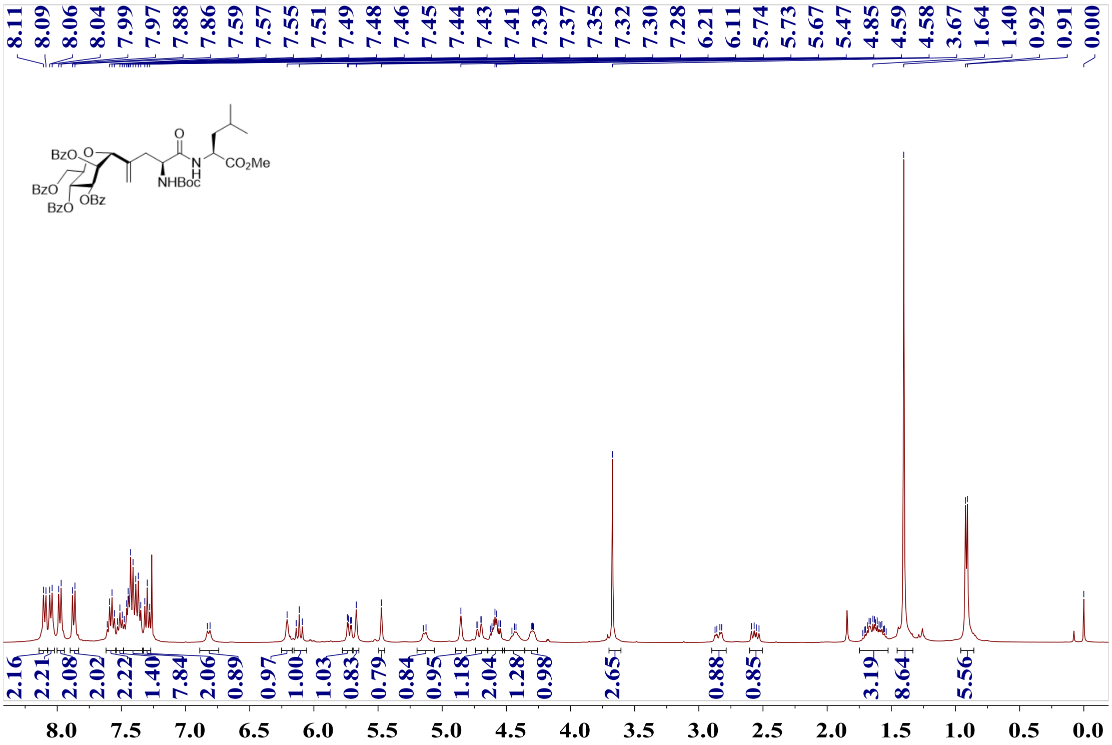

Supplementary Figure 169. ^{13}C NMR spectrum of compound **3al** (125 MHz, $CDCl_3$, 25 °C)

3al

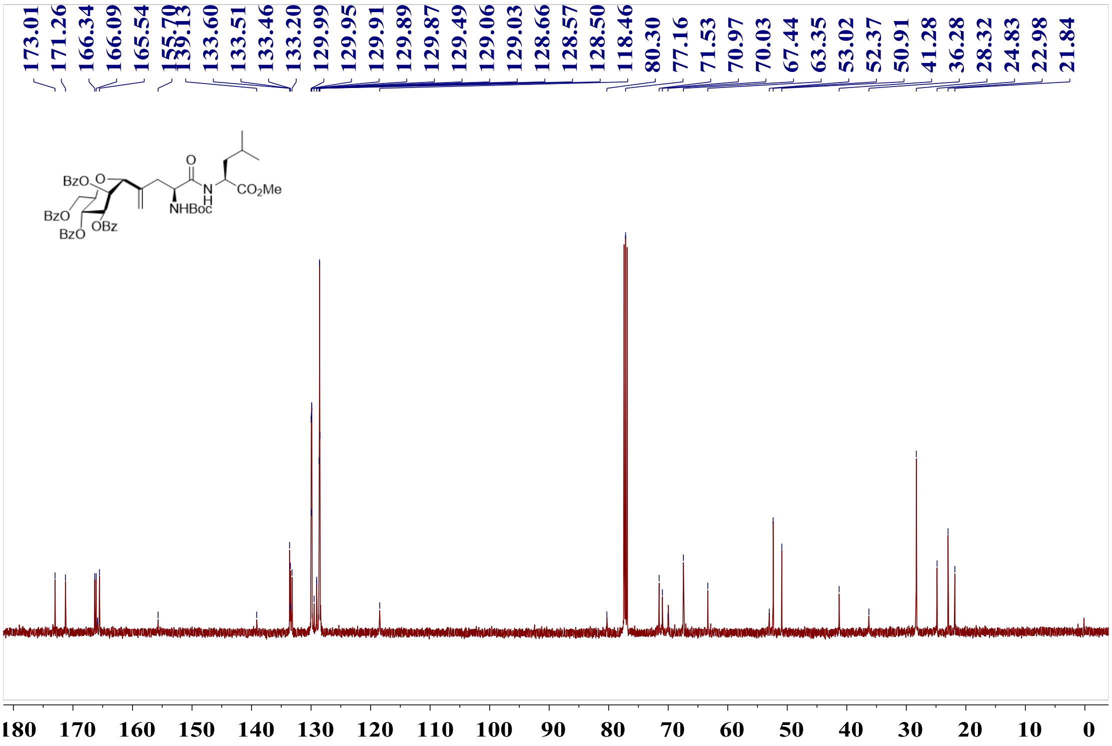

Supplementary Figure 170. ^{1}H NMR spectrum of compound **3am** (500 MHz, $CDCl_3$, 25 °C)

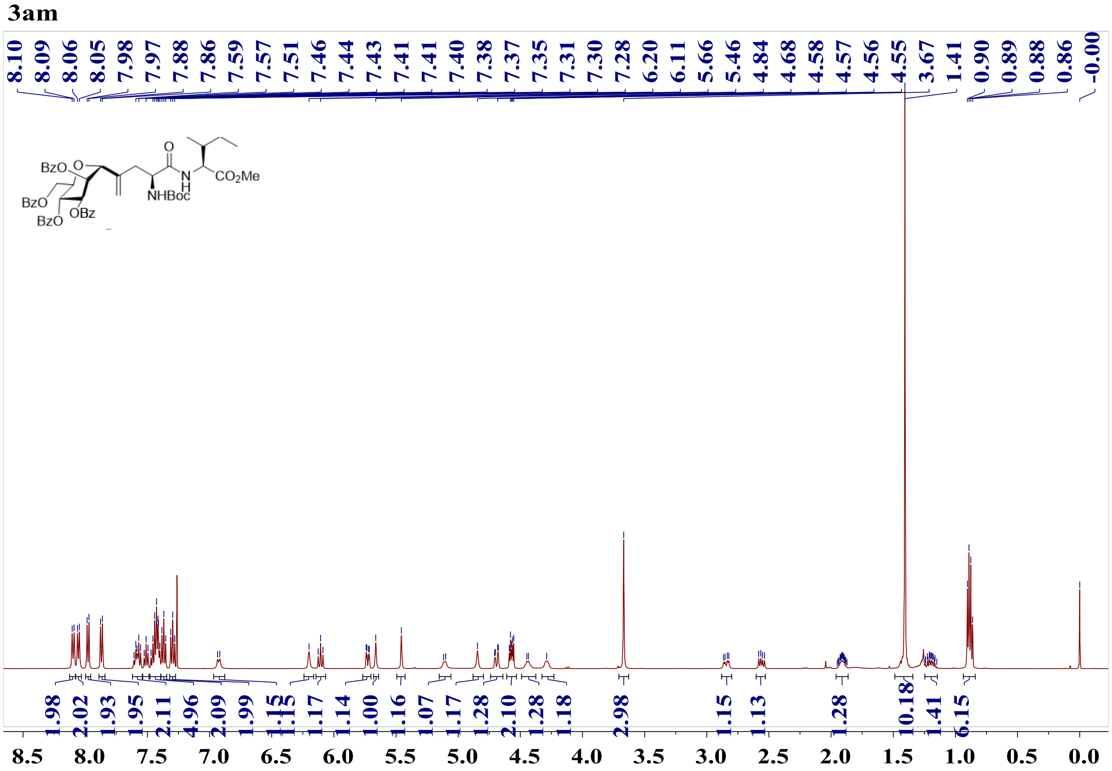

Supplementary Figure 171. ^{13}C NMR spectrum of compound **3am** (125 MHz, $CDCl_3$, 25 °C)

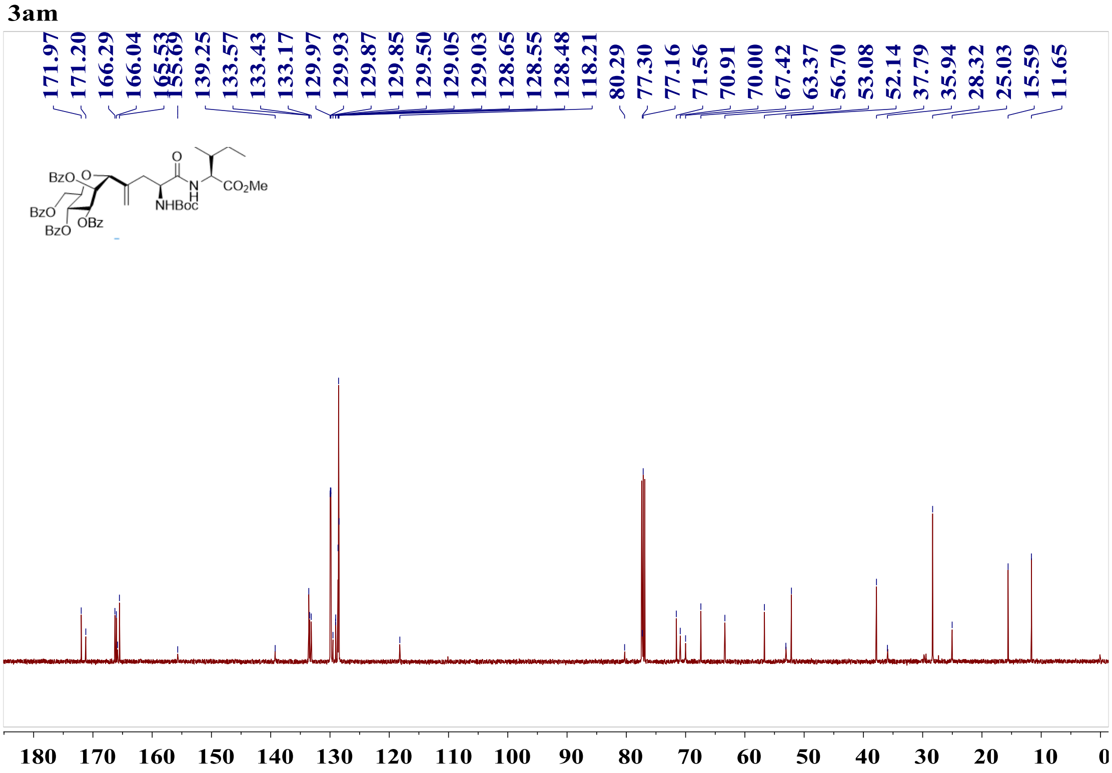

Supplementary Figure 172. ^{1}H NMR spectrum of compound **3an** (500 MHz, $CDCl_3$, 25 ^{o}C)

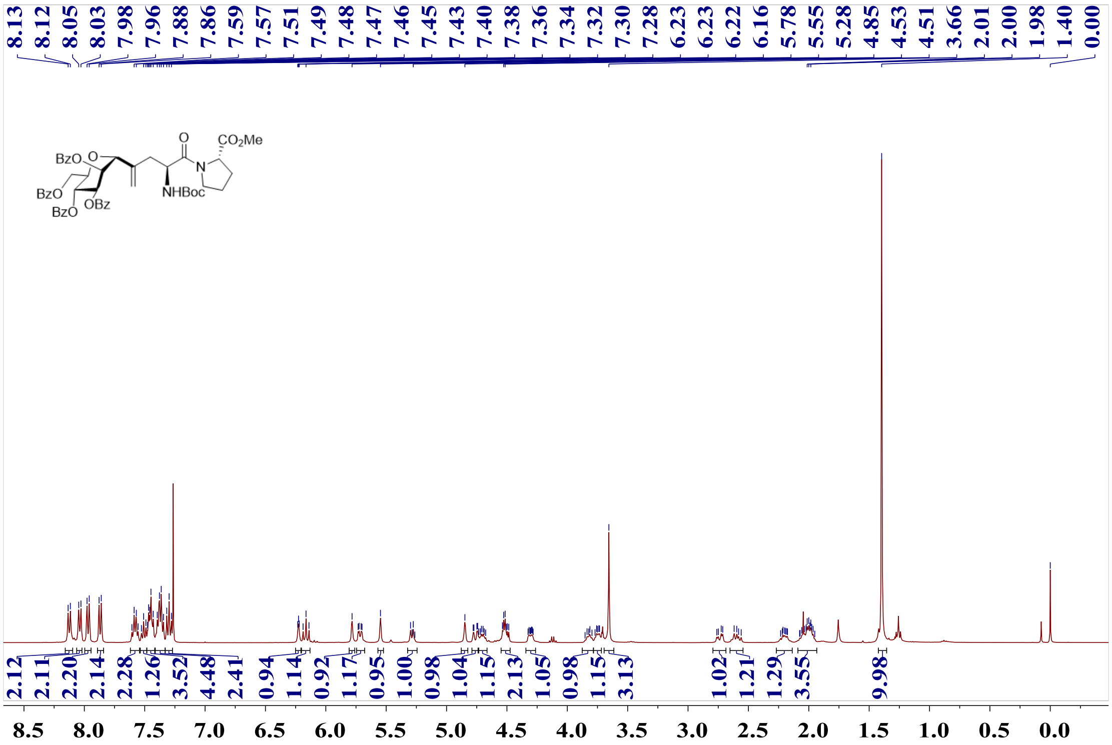

Supplementary Figure 173. ^{13}C NMR spectrum of compound **3an** (125 MHz, $CDCl_3$, 25 ^{o}C)

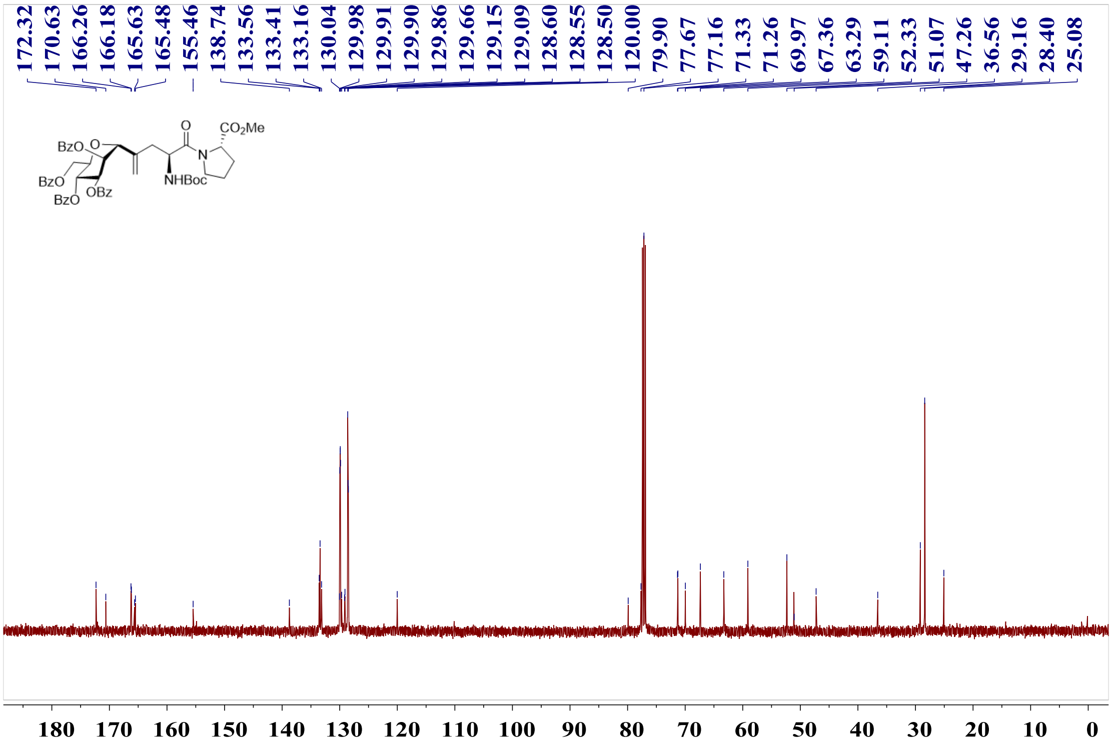

Supplementary Figure 174. ^{1}H NMR spectrum of compound **3ao** (500 MHz, $CDCl_3$, 25 °C)

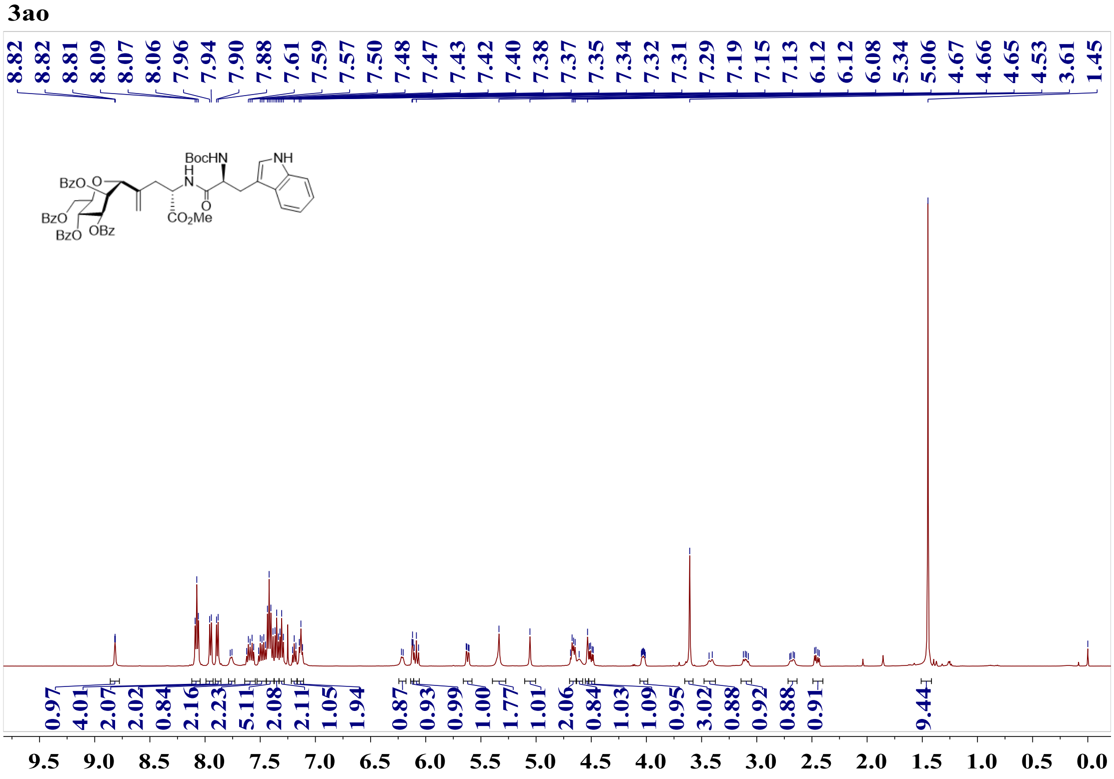

Supplementary Figure 175. ^{13}C NMR spectrum of compound **3ao** (125 MHz, $CDCl_3$, 25 °C)

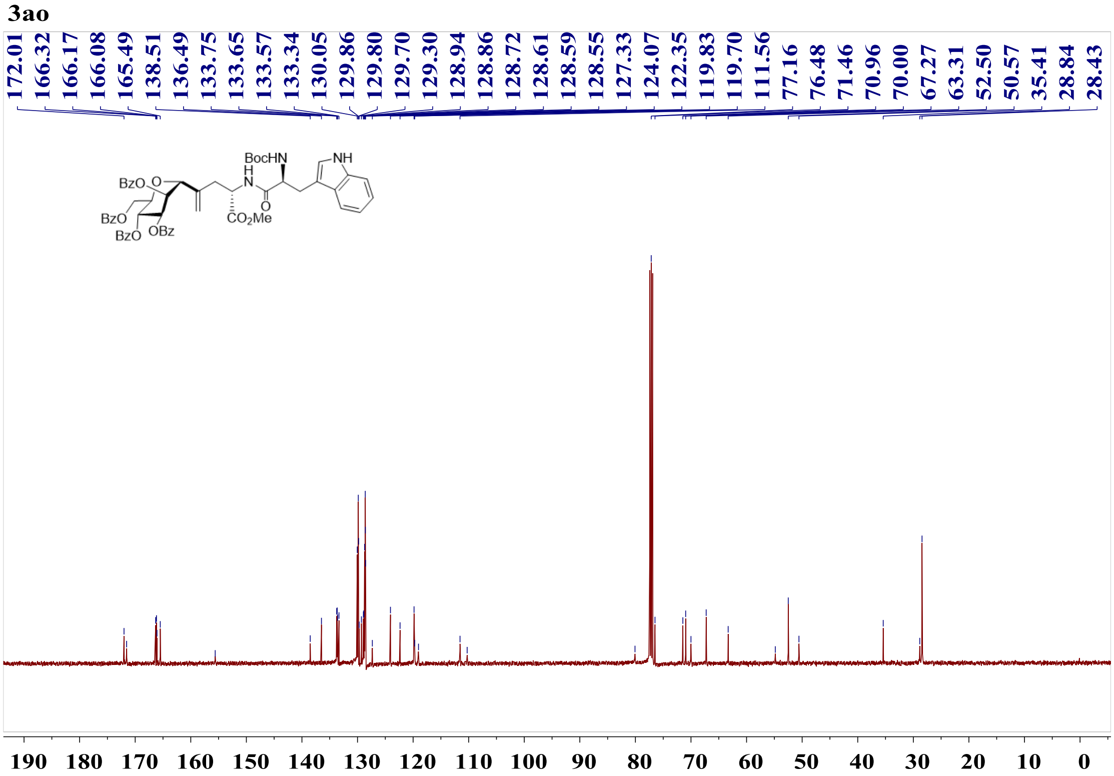

Supplementary Figure 176. ^{1}H NMR spectrum of compound **3ap** (500 MHz, $CDCl_3$, 25 °C)

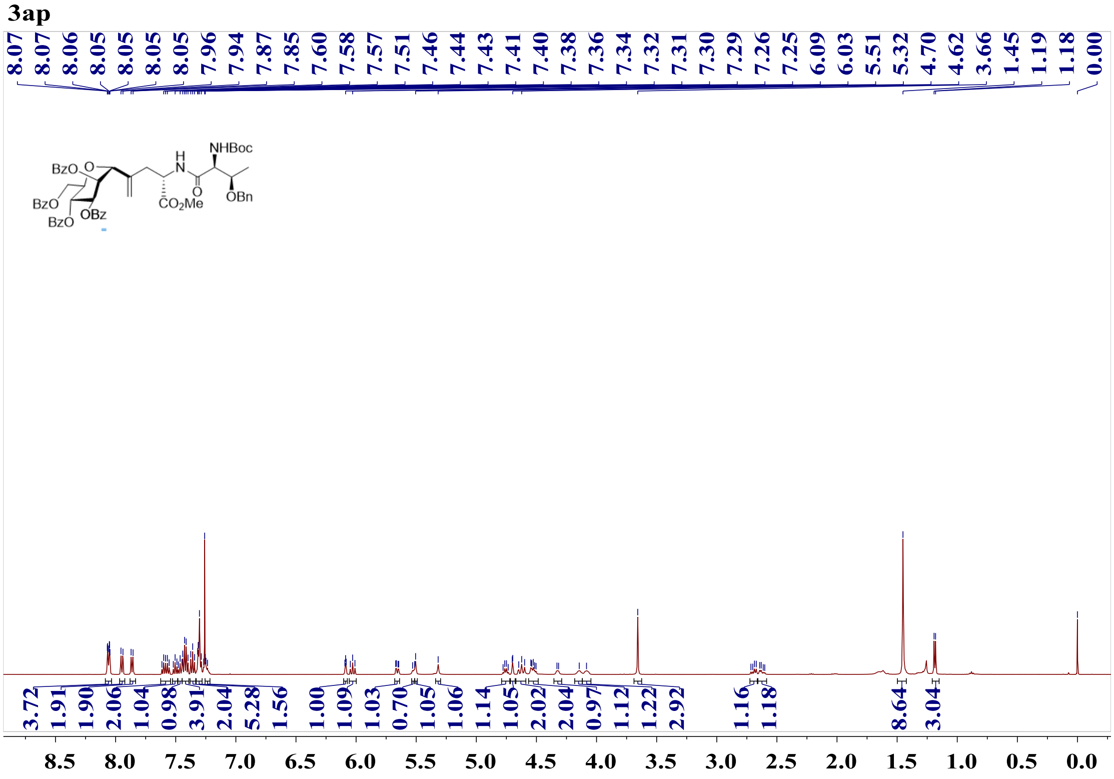

Supplementary Figure 177. ^{13}C NMR spectrum of compound **3ap** (125 MHz, $CDCl_3$, 25 °C)

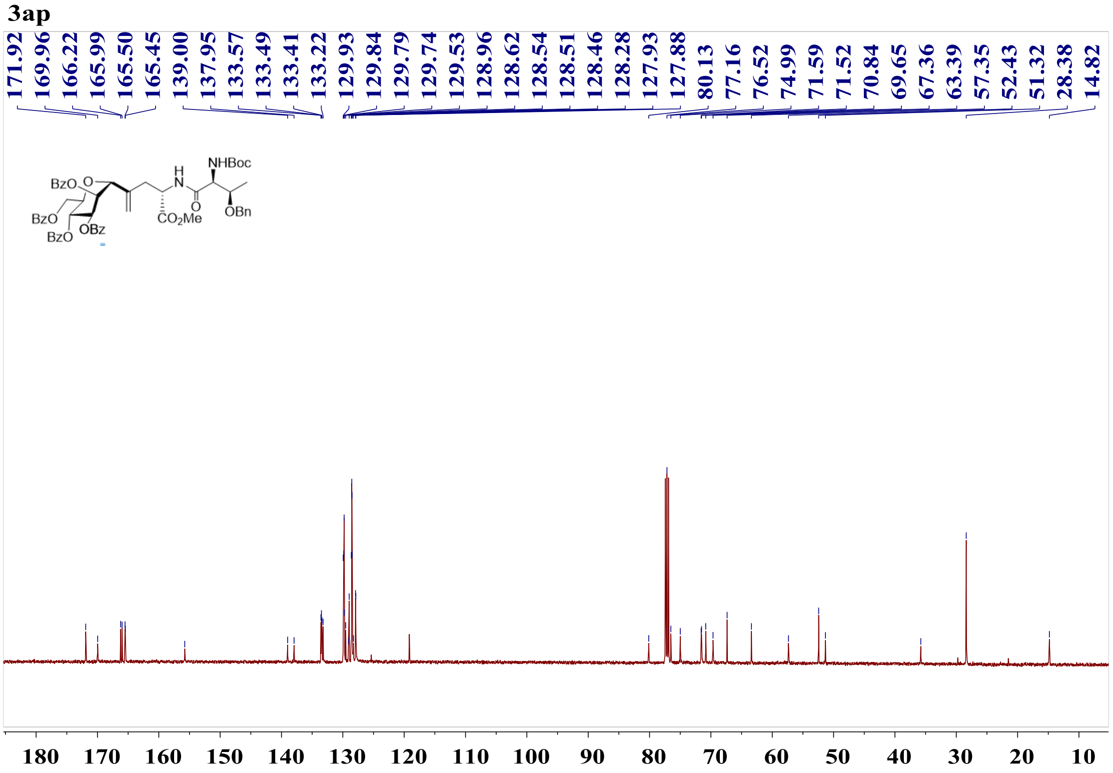

Supplementary Figure 178. ^{1}H NMR spectrum of compound (500 MHz, $CDCl_3$, 25 ^{o}C)

3aq

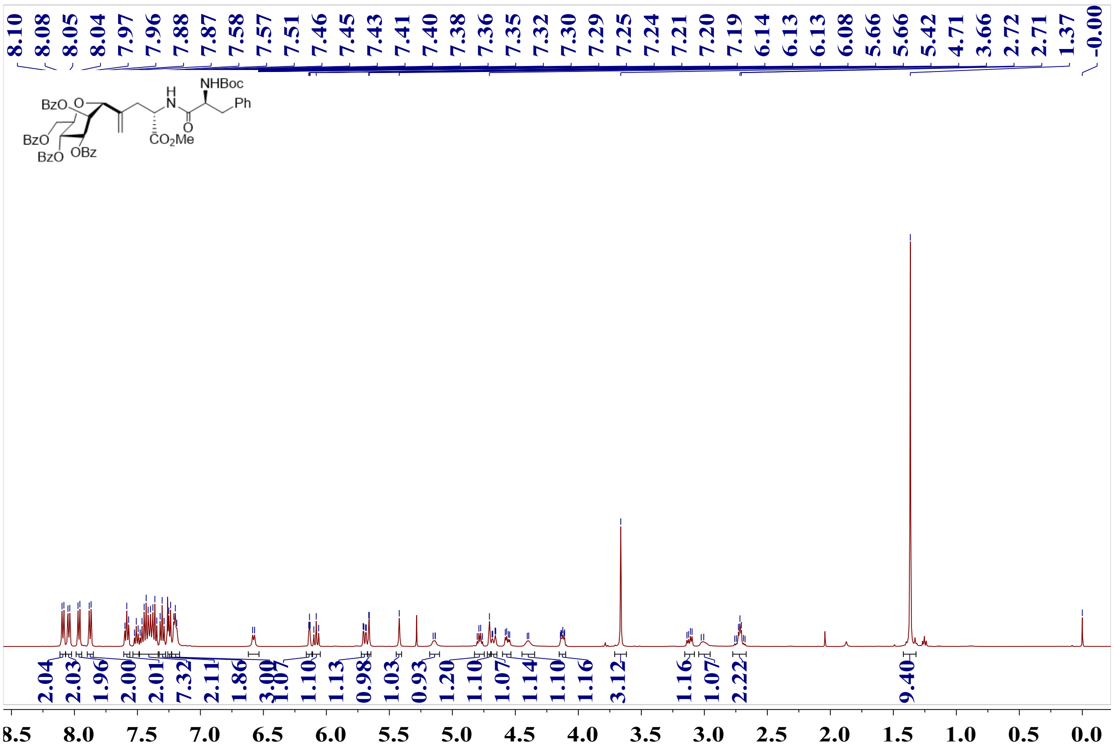

Supplementary Figure 179. ^{13}C NMR spectrum of compound **3aq** (125 MHz, $CDCl_3$, 25 ^{o}C)

3aq

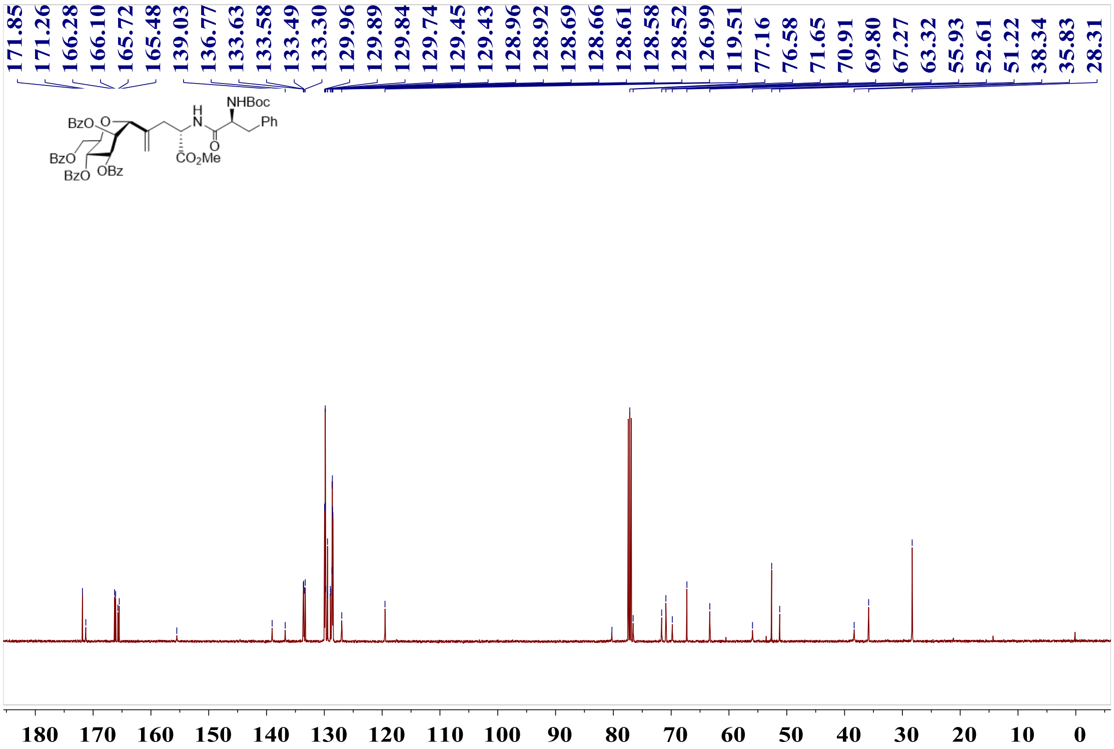

Supplementary Figure 180. ^{1}H NMR spectrum of compound **3ar** (500 MHz, $CDCl_3$, 25 °C)

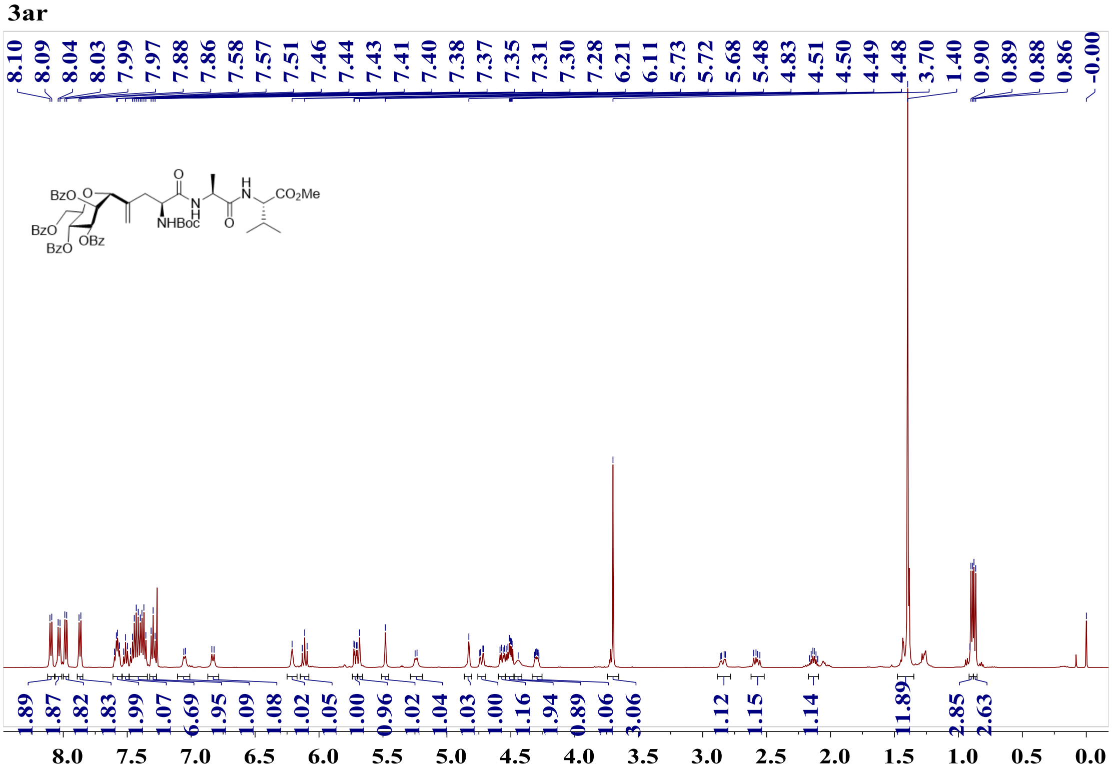

Supplementary Figure 181. ^{13}C NMR spectrum of compound **3ar** (125 MHz, $CDCl_3$, 25 °C)

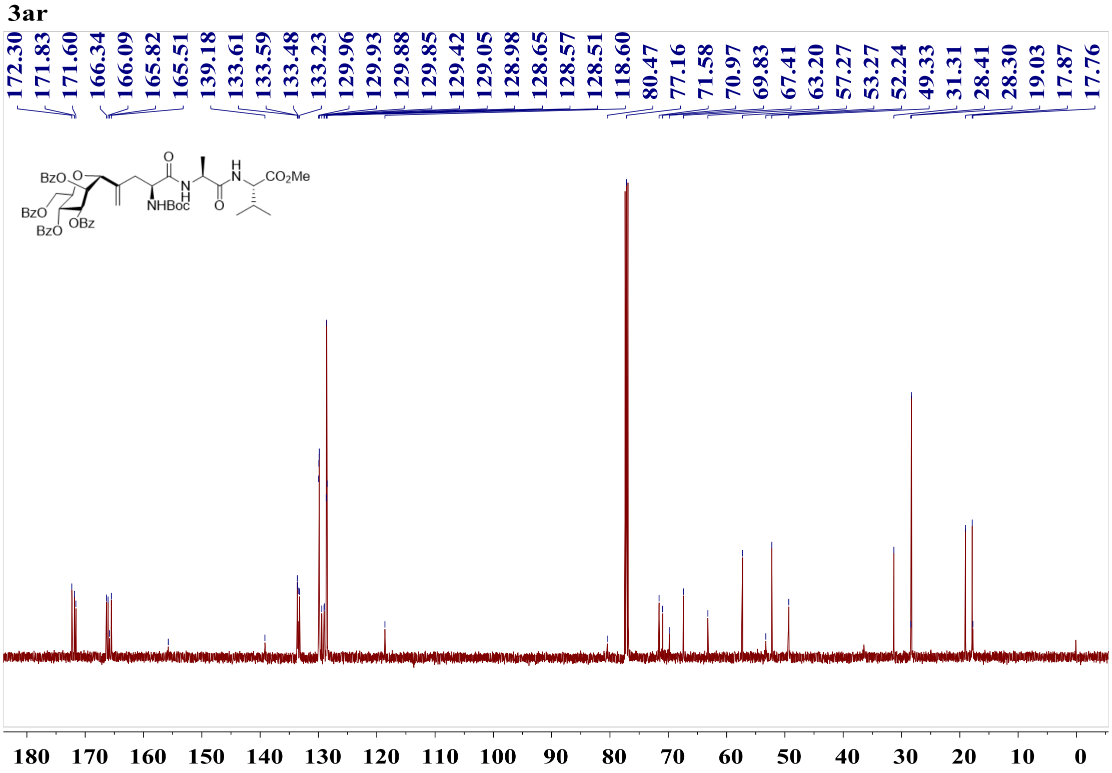

Supplementary Figure 182. ^{1}H NMR spectrum of compound **3as** (500 MHz, $CDCl_3$, 25 °C)

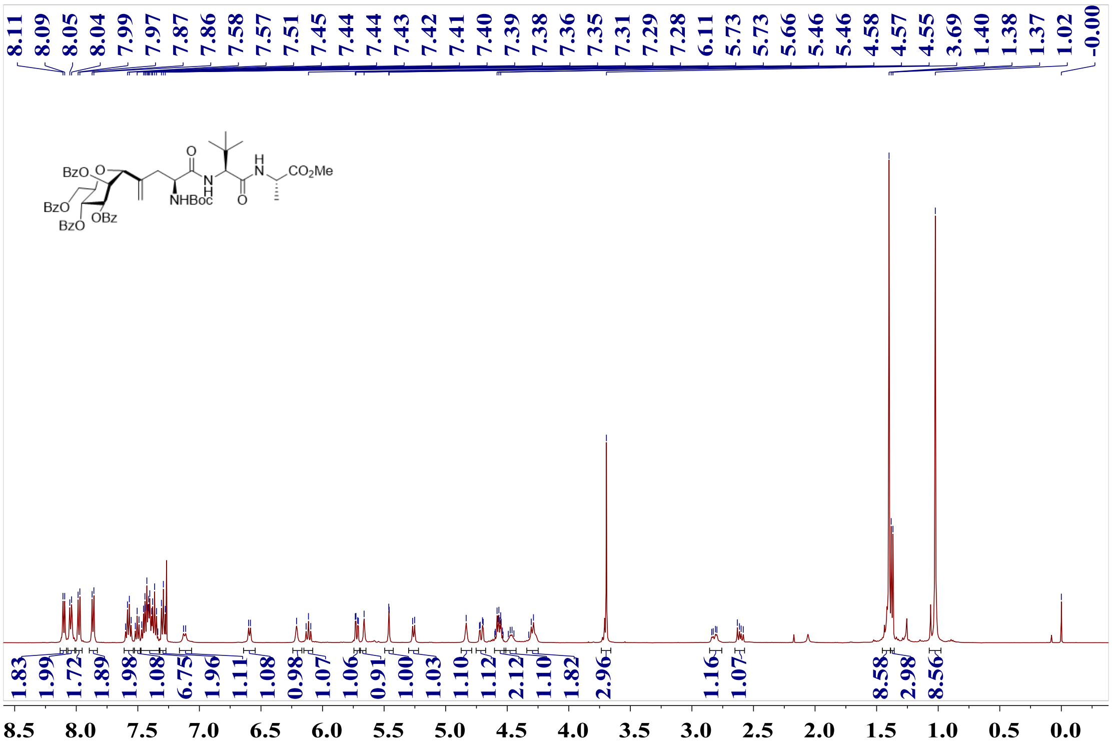

Supplementary Figure 183. ^{13}C NMR spectrum of compound **3as** (125 MHz, $CDCl_3$, 25 °C)

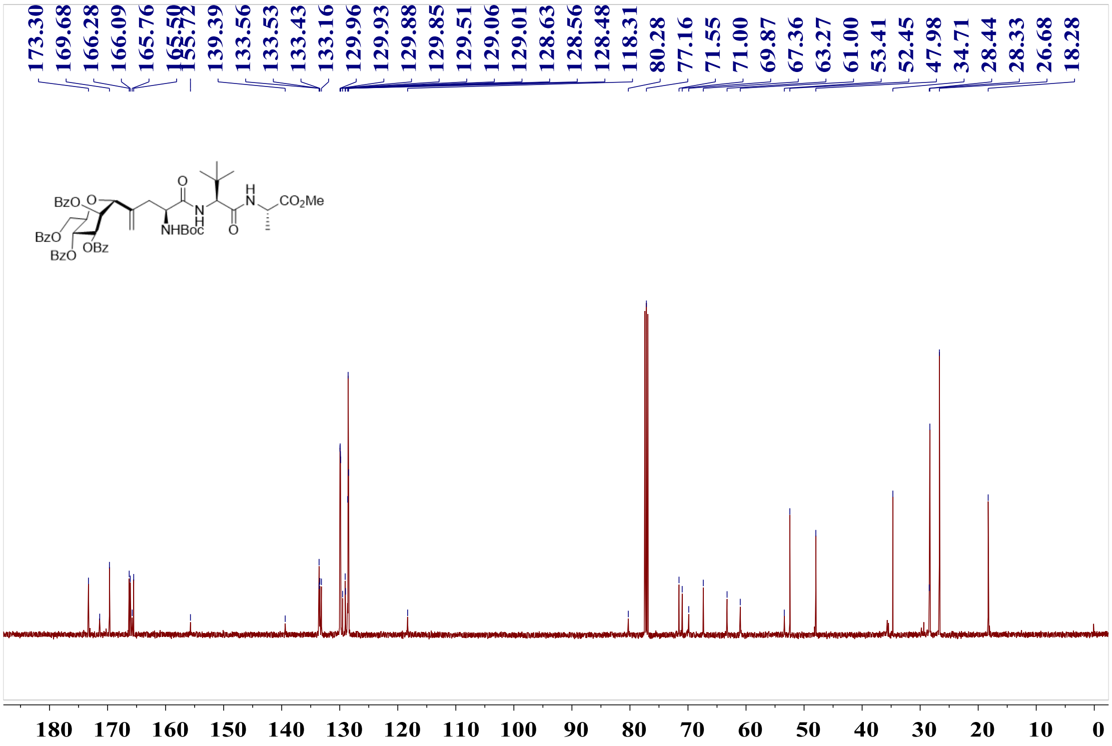

Supplementary Figure 184. ^{1}H NMR spectrum of compound **3at** (500 MHz, $CDCl_3$, 25 ^{o}C)

3at

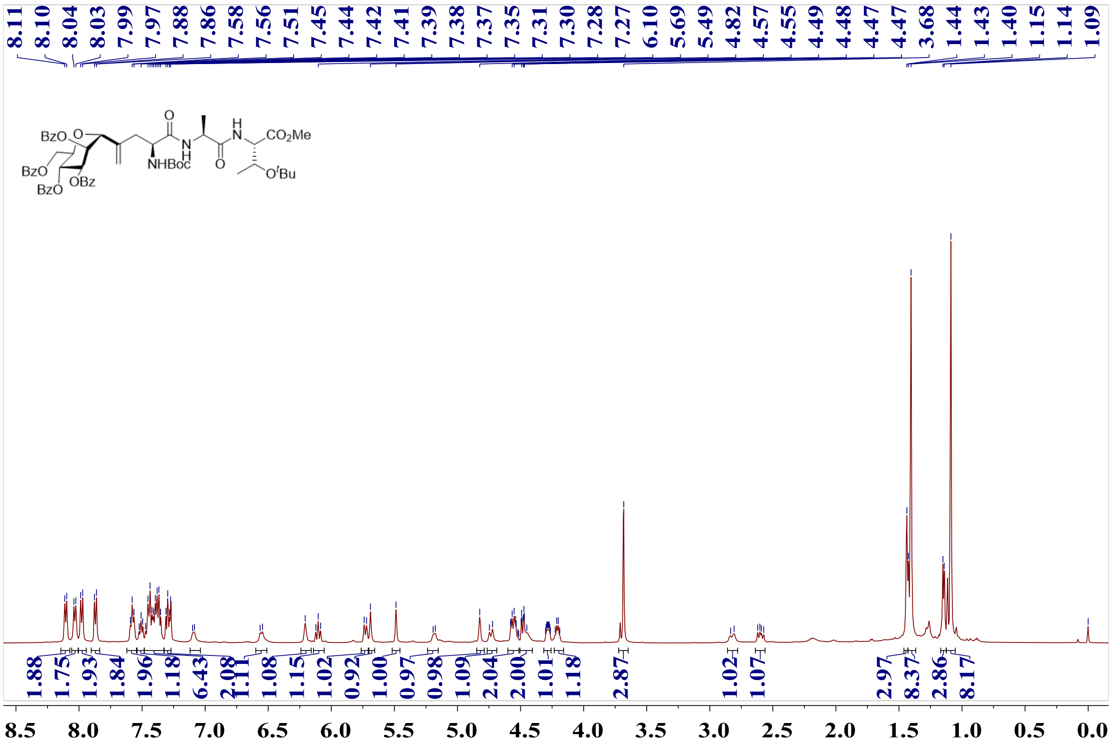

Supplementary Figure 185. ^{13}C NMR spectrum of compound **3at** (125 MHz, $CDCl_3$, 25 ^{o}C)

3at

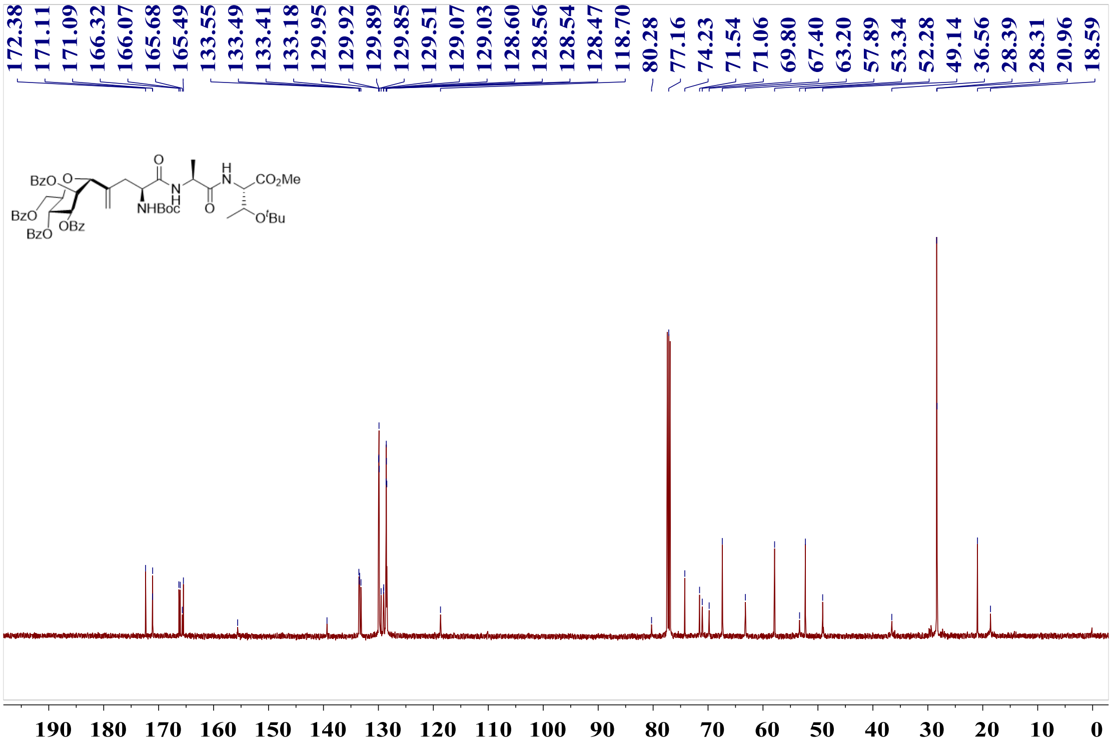

Supplementary Figure 186. ^{1}H NMR spectrum of compound **3ba** (500 MHz, $CDCl_3$, 25 °C)

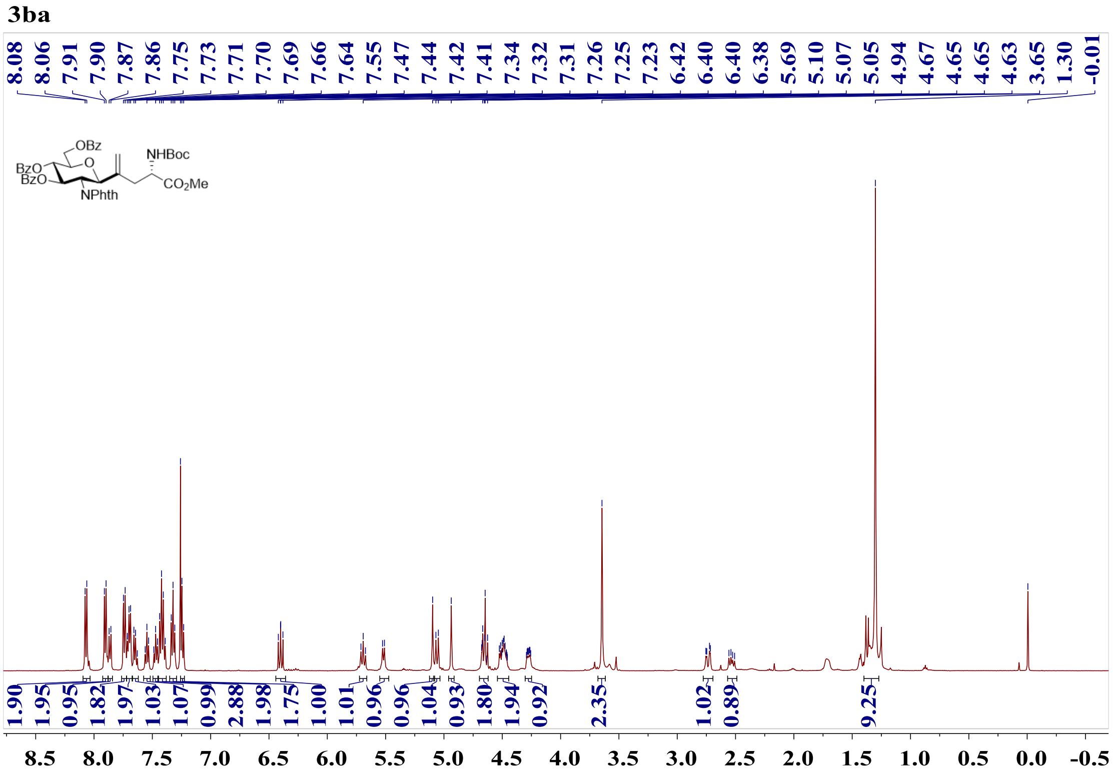

Supplementary Figure 187. ^{13}C NMR spectrum of compound **3ba** (125 MHz, $CDCl_3$, 25 °C)

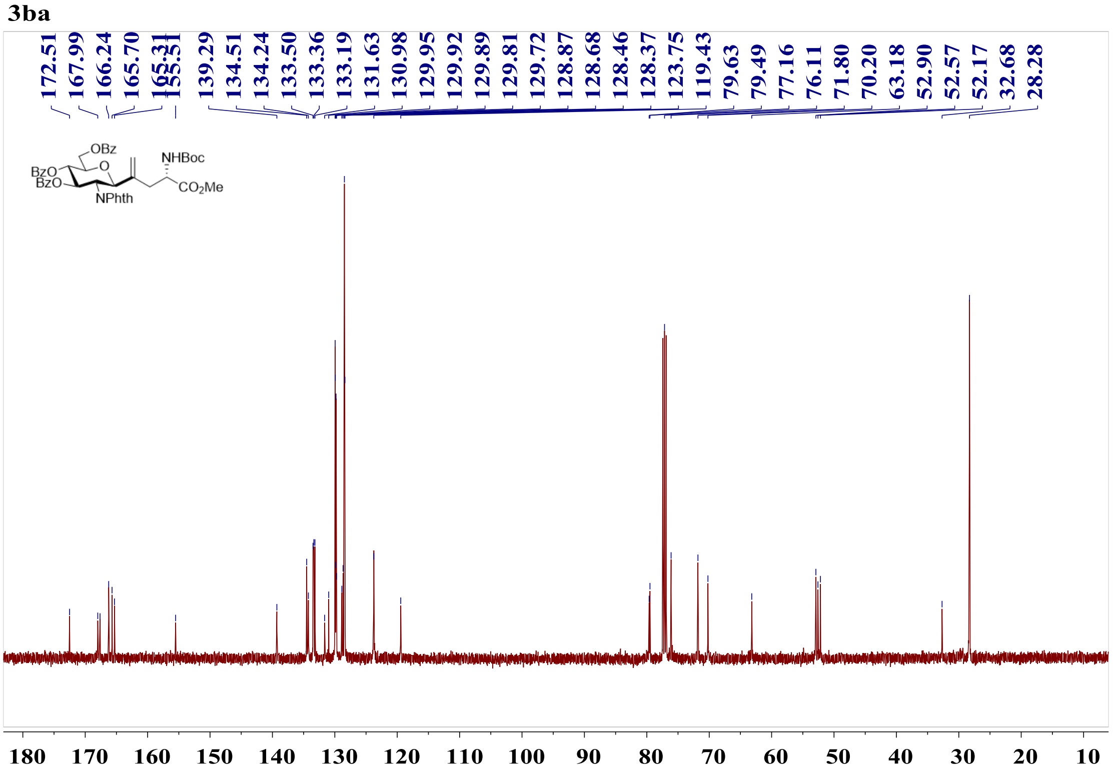

Supplementary Figure 188. ^{1}H NMR spectrum of compound **3bl** (500 MHz, $CDCl_3$, 25 °C)

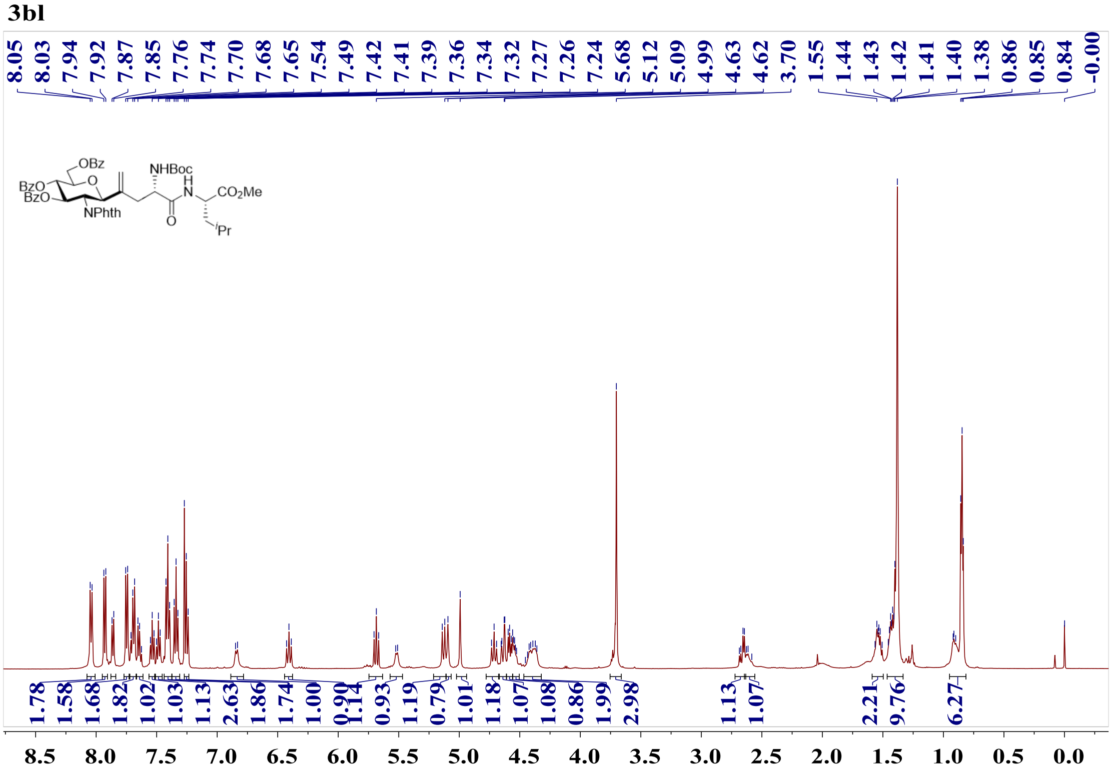

Supplementary Figure 189. ^{13}C NMR spectrum of compound **3bl** (125 MHz, $CDCl_3$, 25 °C)

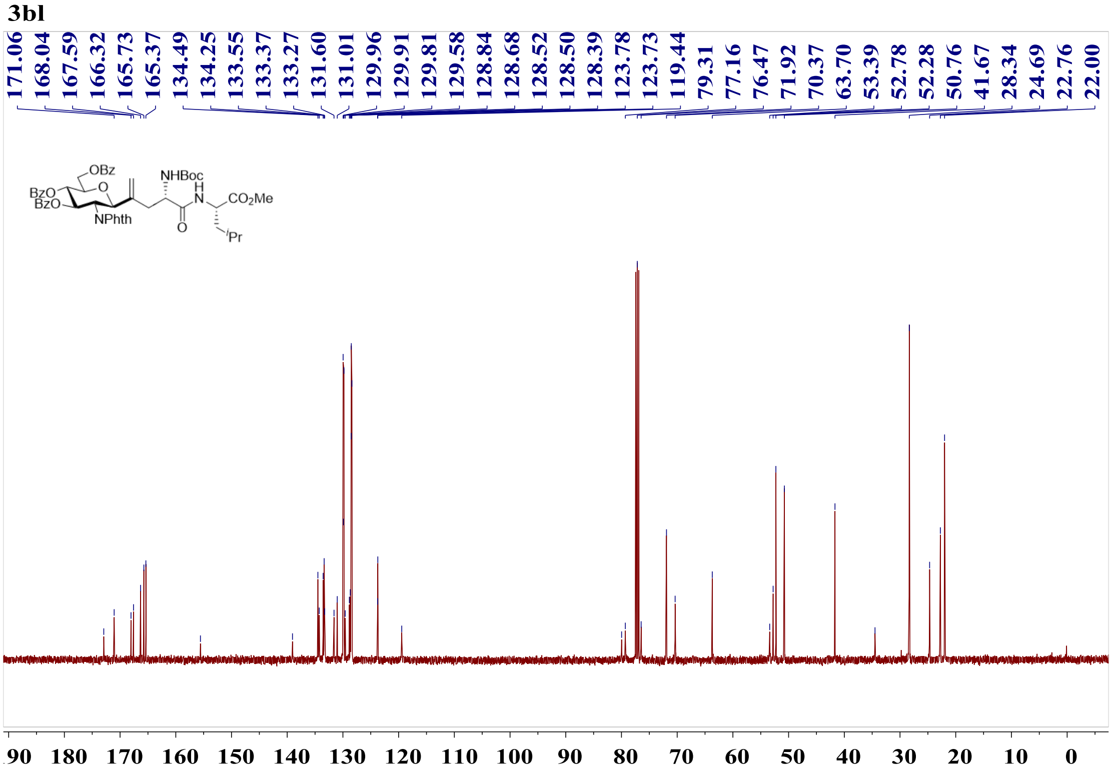

Supplementary Figure 190. ^{1}H NMR spectrum of compound **3bt** (500 MHz, $CDCl_3$, 25 °C)

3bt

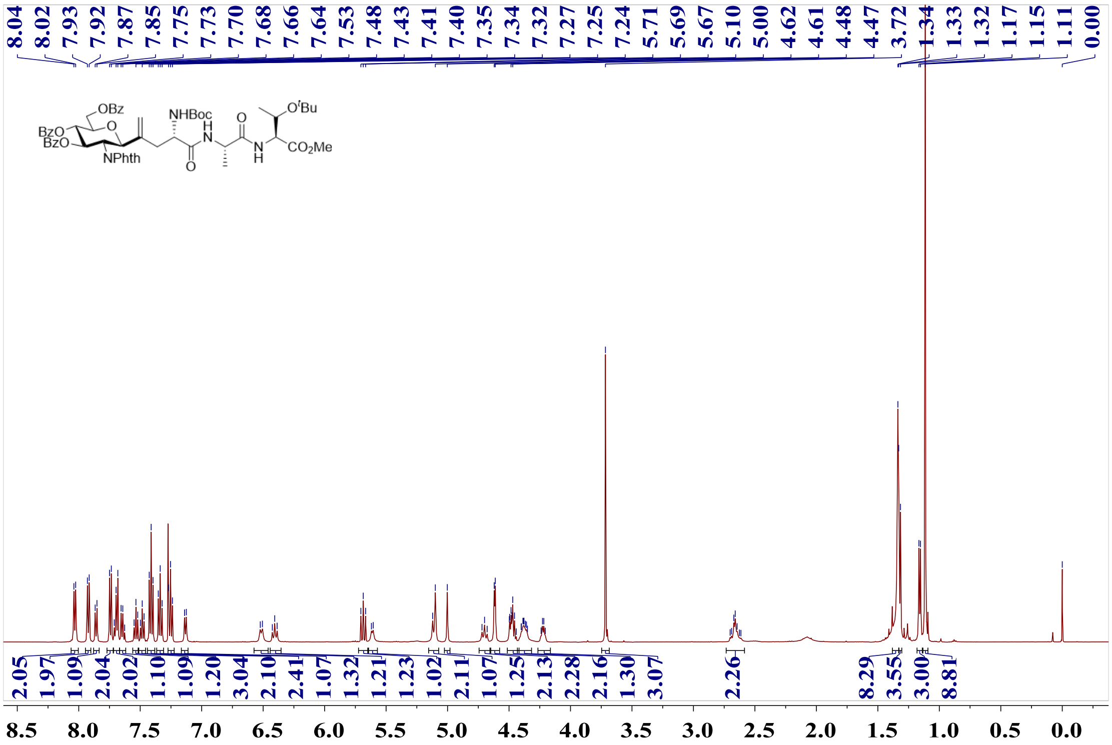

Supplementary Figure 191. ^{13}C NMR spectrum of compound **3bt** (125 MHz, $CDCl_3$, 25 °C)

3bt

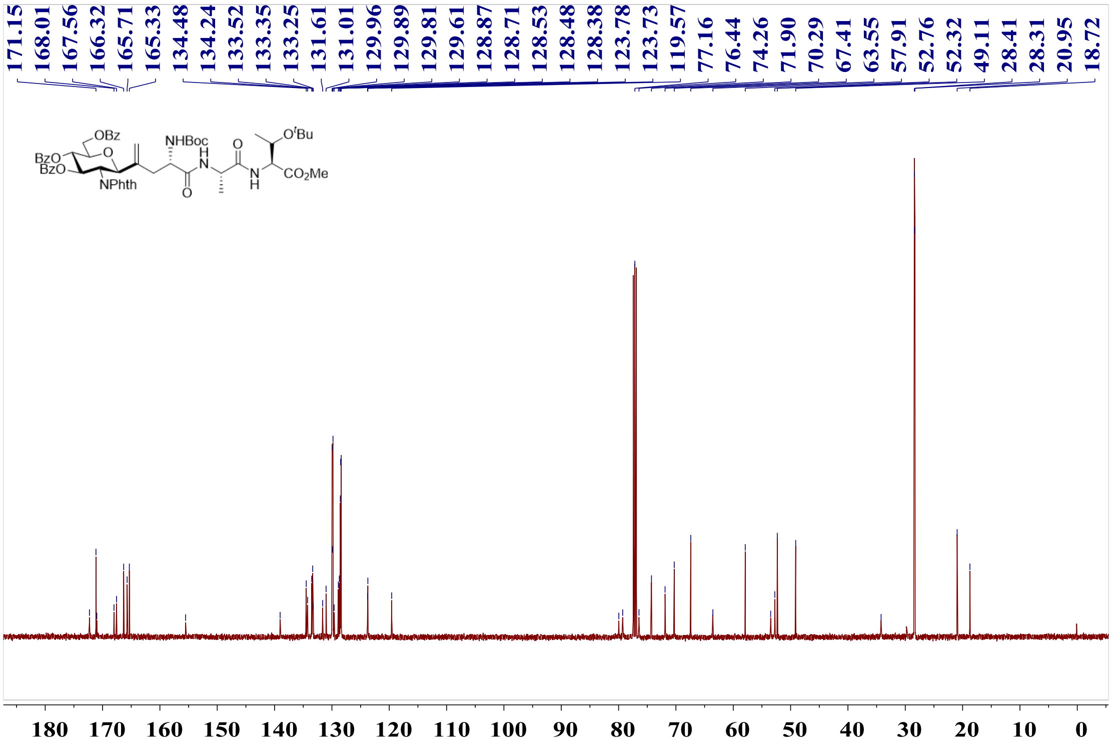

Supplementary Figure 192. ^{1}H NMR spectrum of compound **3bu** (500 MHz, $CDCl_3$, 25 °C)

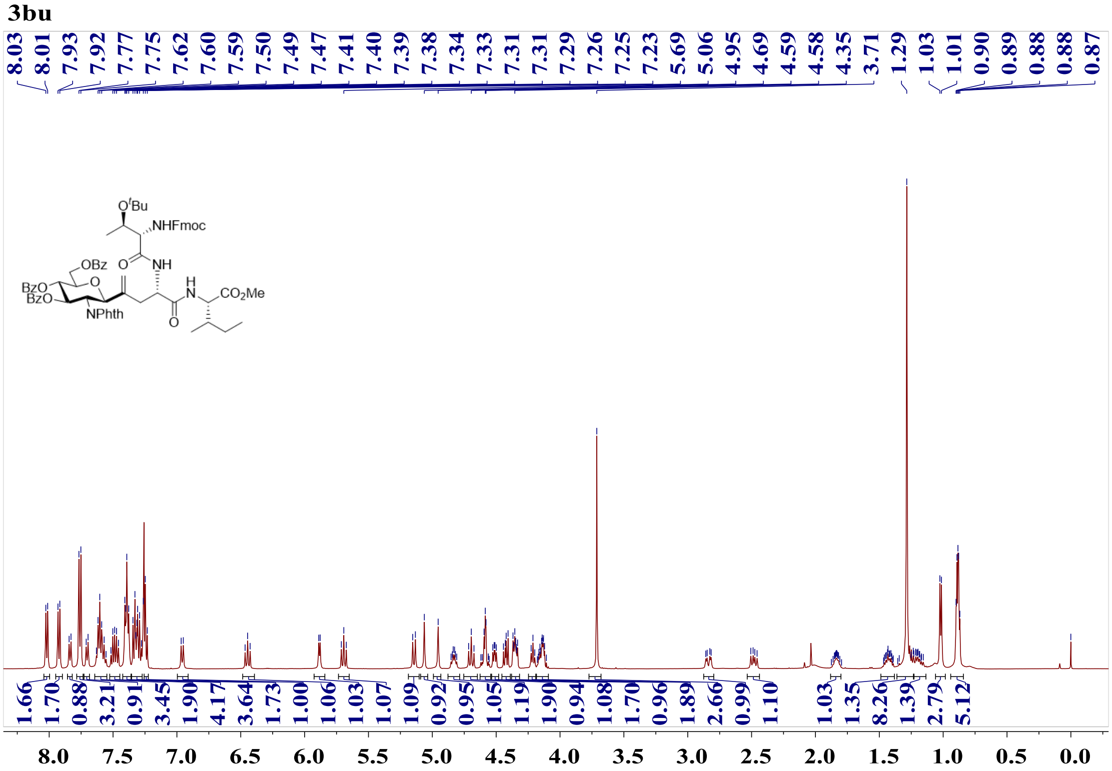

Supplementary Figure 193. ^{13}C NMR spectrum of compound **3bu** (125 MHz, $CDCl_3$, 25 °C)

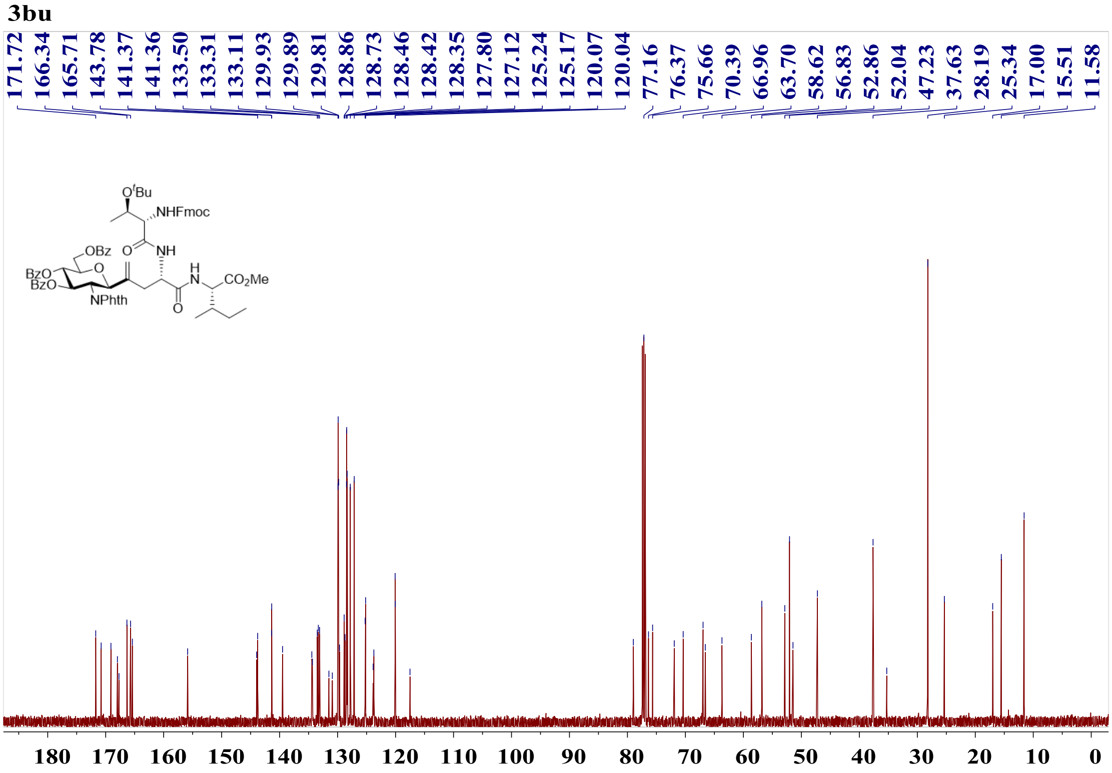

Supplementary Figure 194. ^{1}H NMR spectrum of compound **3bp** (500 MHz, $CDCl_3$, 25 °C)

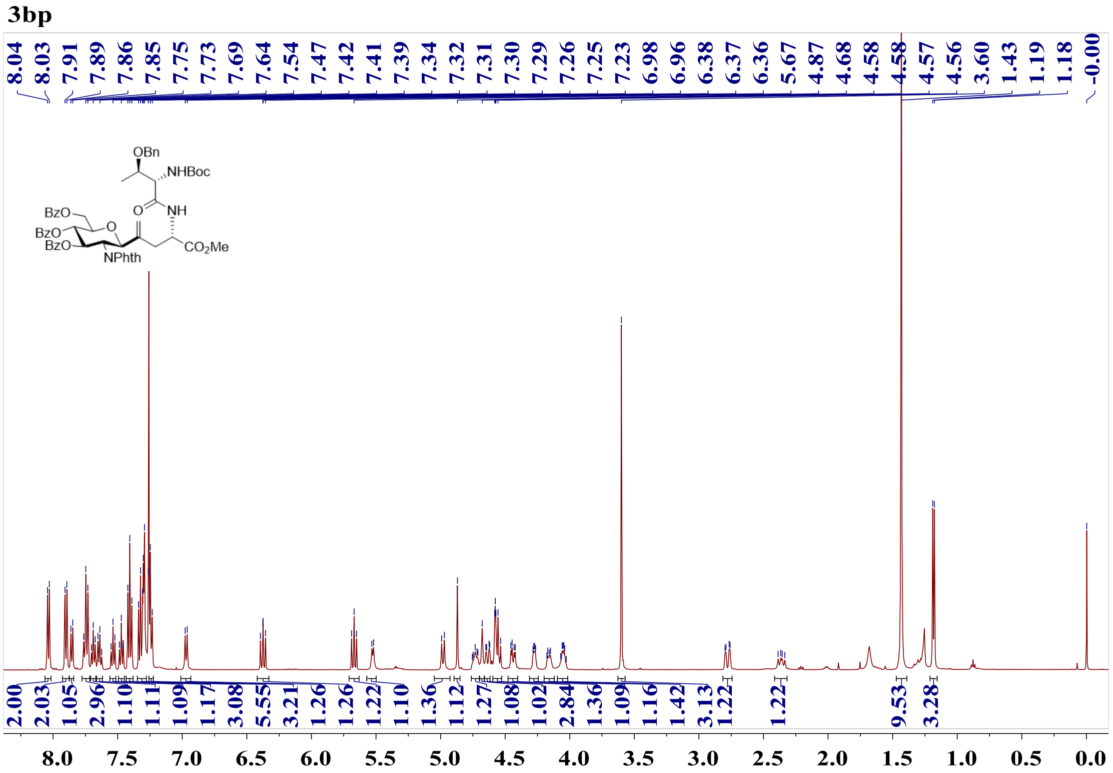

Supplementary Figure 195. ^{13}C NMR spectrum of compound **3bp** (125 MHz, $CDCl_3$, 25 °C)

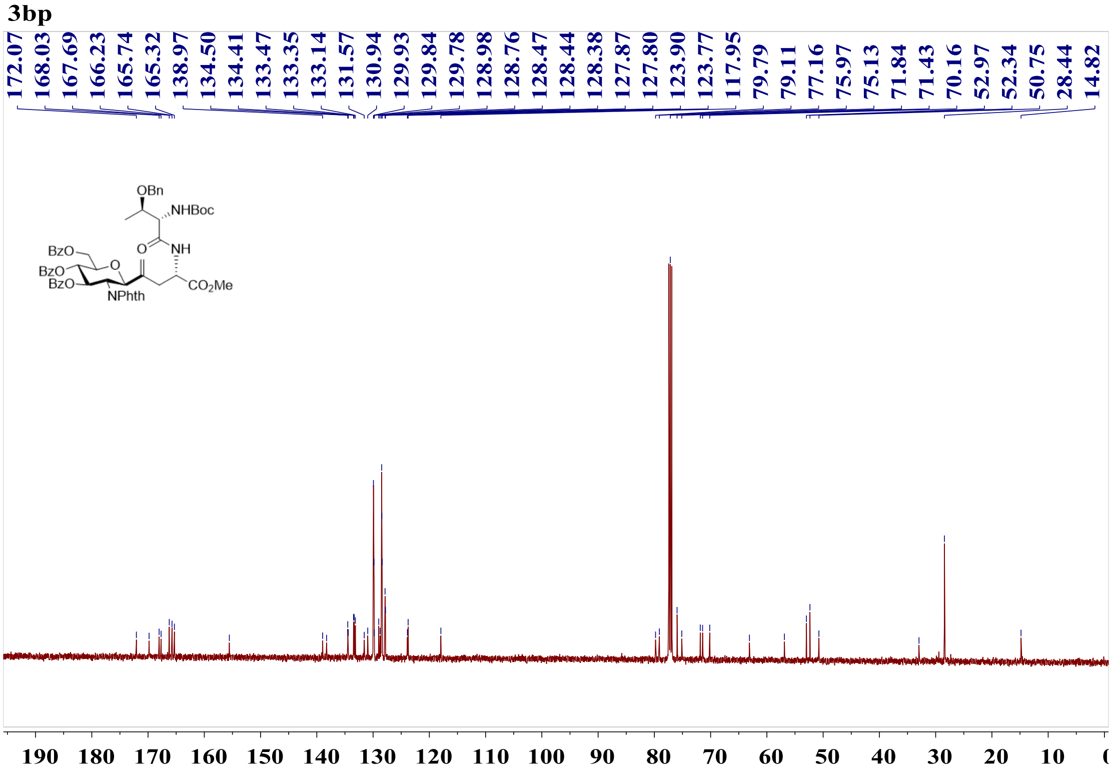

Supplementary Figure 196. ^{1}H NMR spectrum of compound **3ca** (500 MHz, $CDCl_3$, 25 ^{o}C)

3ca

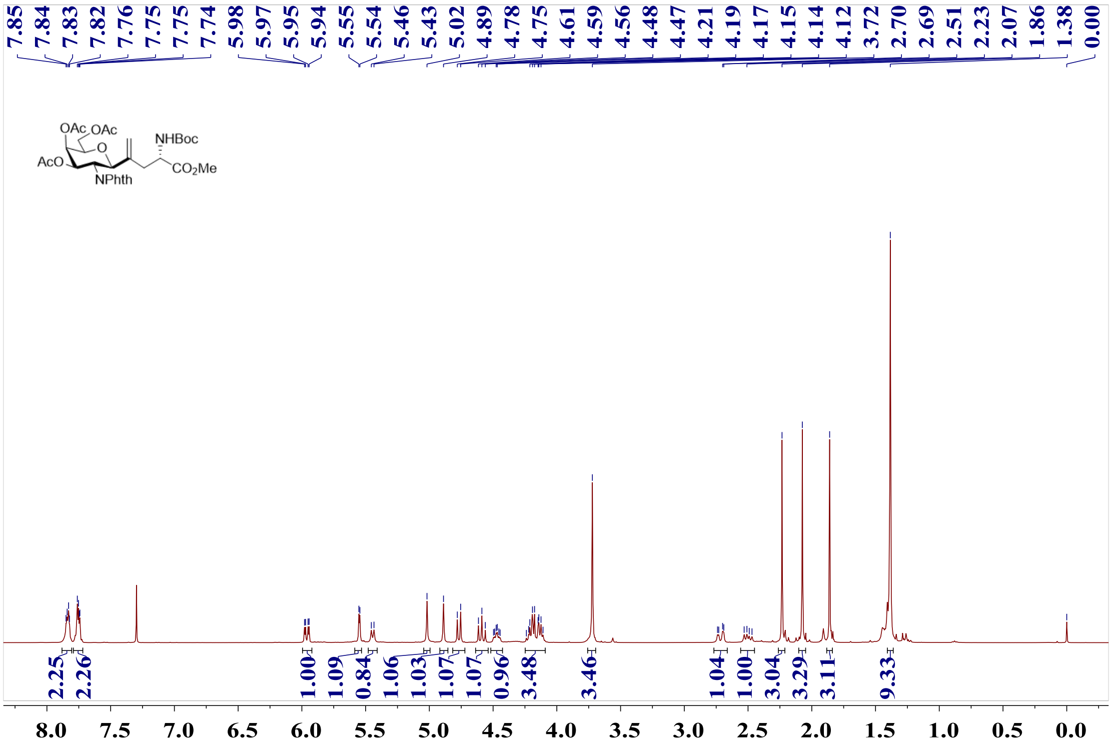

Supplementary Figure 197. ^{13}C NMR spectrum of compound **3ca** (125 MHz, $CDCl_3$, 25 ^{o}C)

3ca

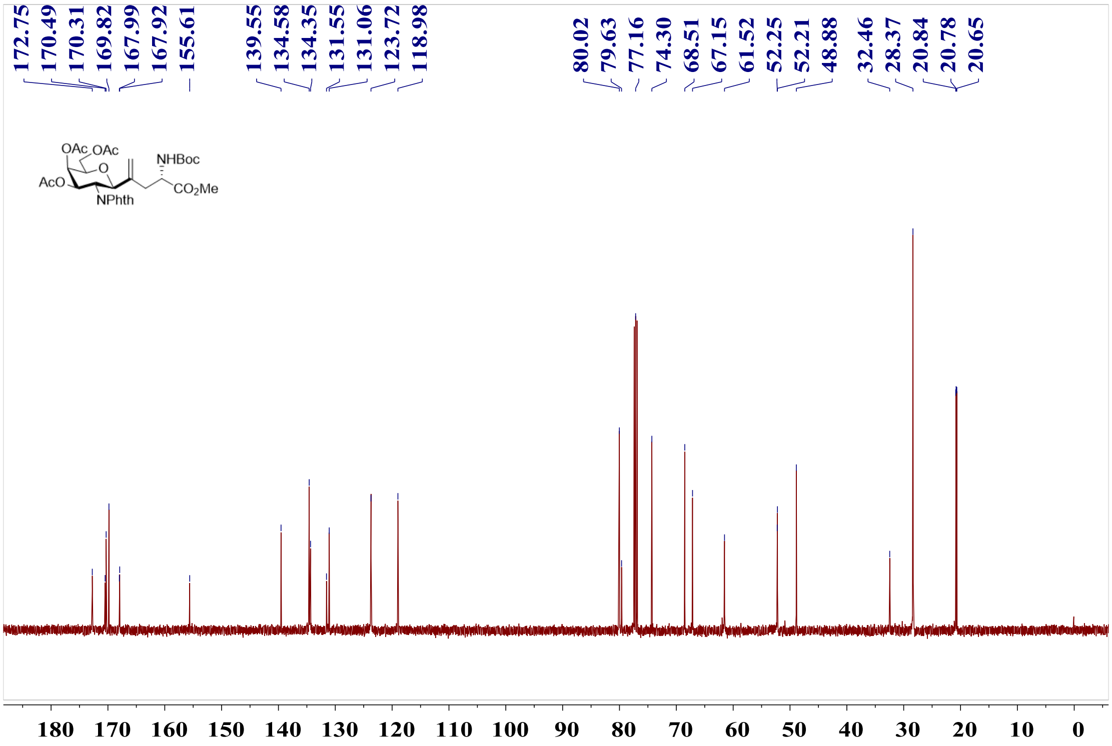

Supplementary Figure 198. ^{1}H NMR spectrum of compound **3da-β** (500 MHz, $CDCl_3$, 25 °C)

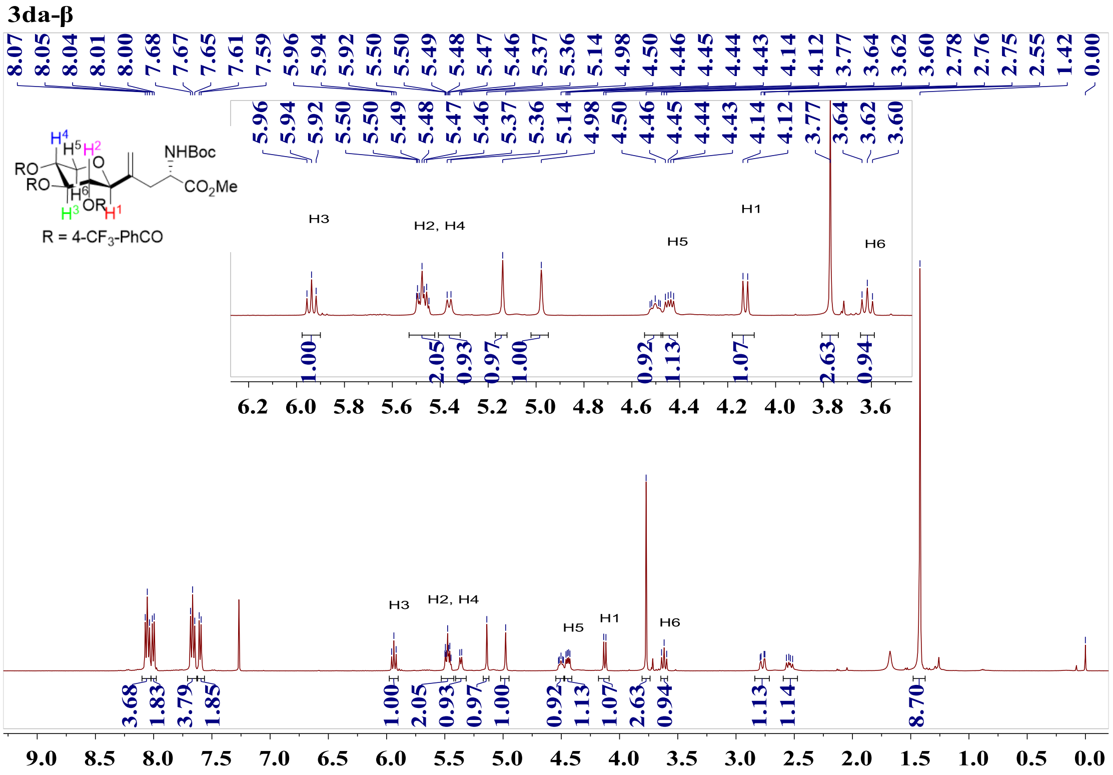

Supplementary Figure 199. ^{13}C NMR spectrum of compound **3da-β** (125 MHz, $CDCl_3$, 25 °C)

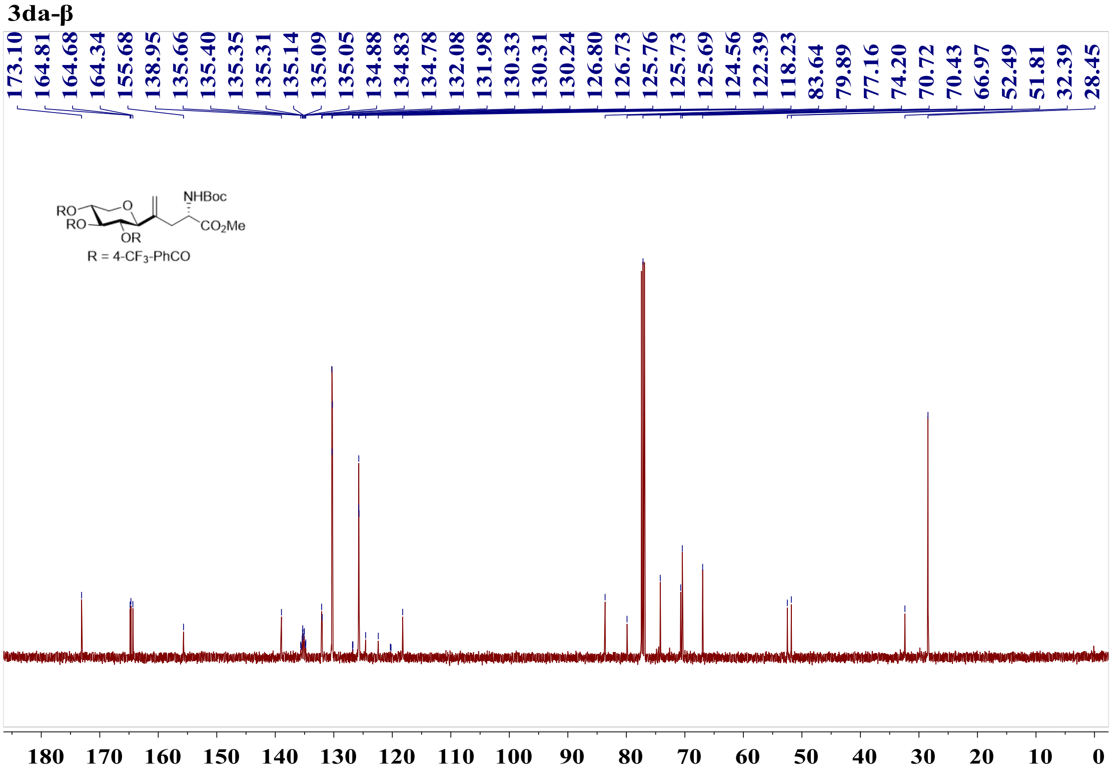

Supplementary Figure 200. ^{1}H-COSY spectrum of compound **3da-β** (500 MHz, $CDCl_3$, 25 °C)

Key COSY correlations

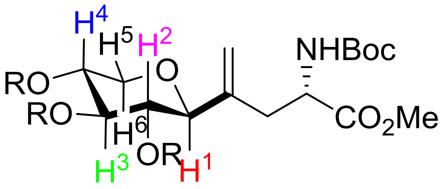

3da-β, $^4C_1(\beta)$, R = 4-CF_3-PhCO

3da-β-COSY

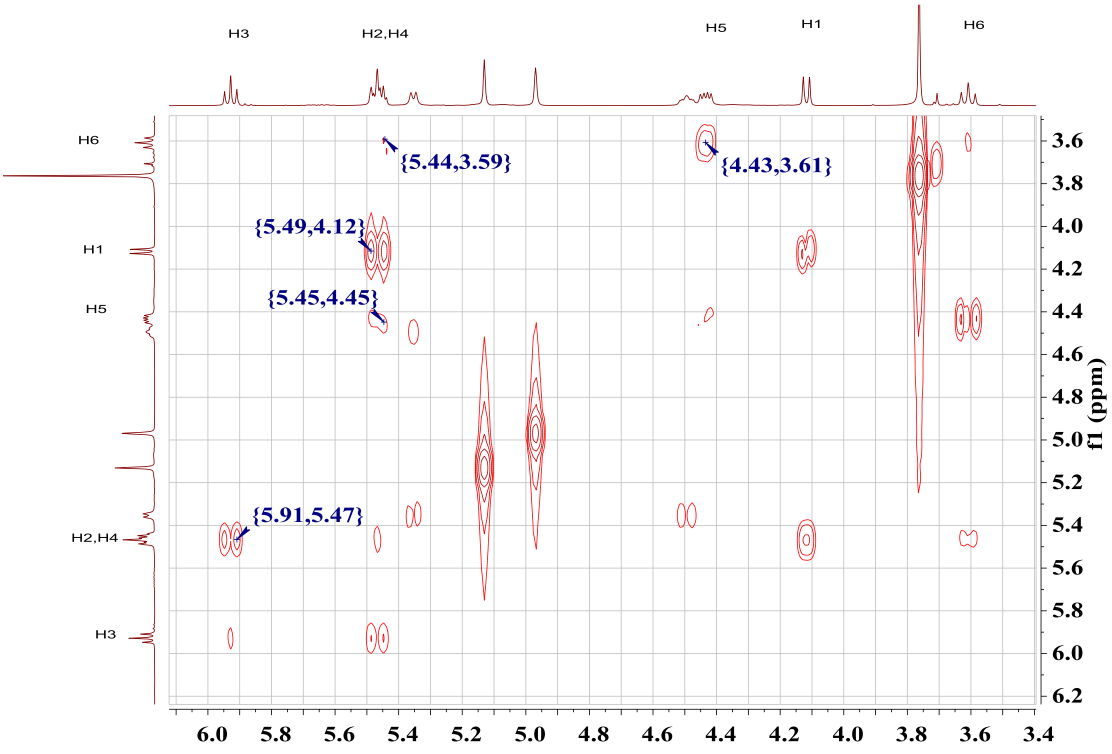

Supplementary Figure 201. HSQC NMR spectrum of compound **3da-β** ($CDCl_3$)

Key HSQC correlations

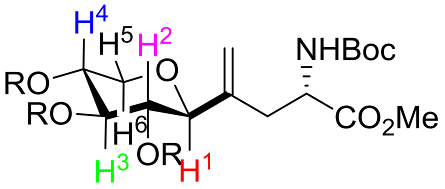

3da-β, $^4C_1(\beta)$, R = 4-CF_3-PhCO

3da-β-HSQC

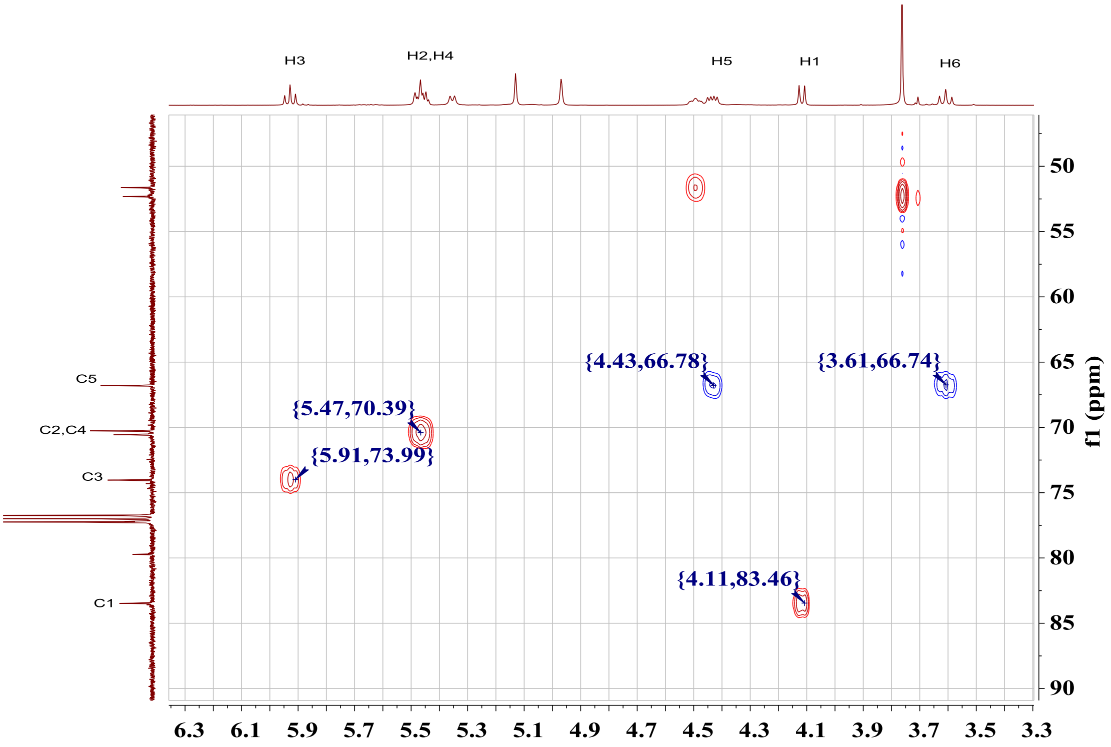

Supplementary Figure 202. ^{1}H NMR spectrum of compound **3da-α** (600 MHz, $CDCl_3$, 25 °C)

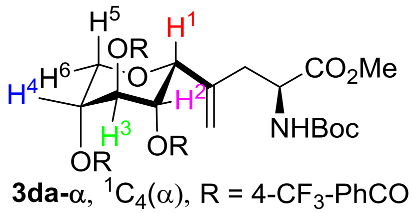

3da-α, $^{1}C_4$(α), R = 4-CF_3-PhCO

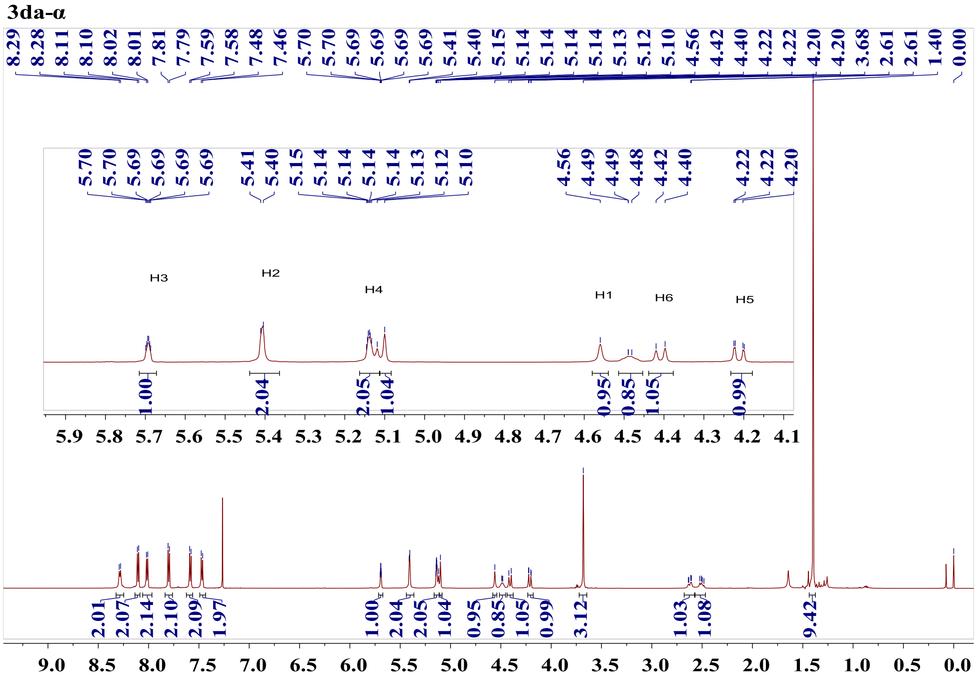

Supplementary Figure 203. ^{13}C NMR spectrum of compound **3da-α** (150 MHz, $CDCl_3$, 25 °C)

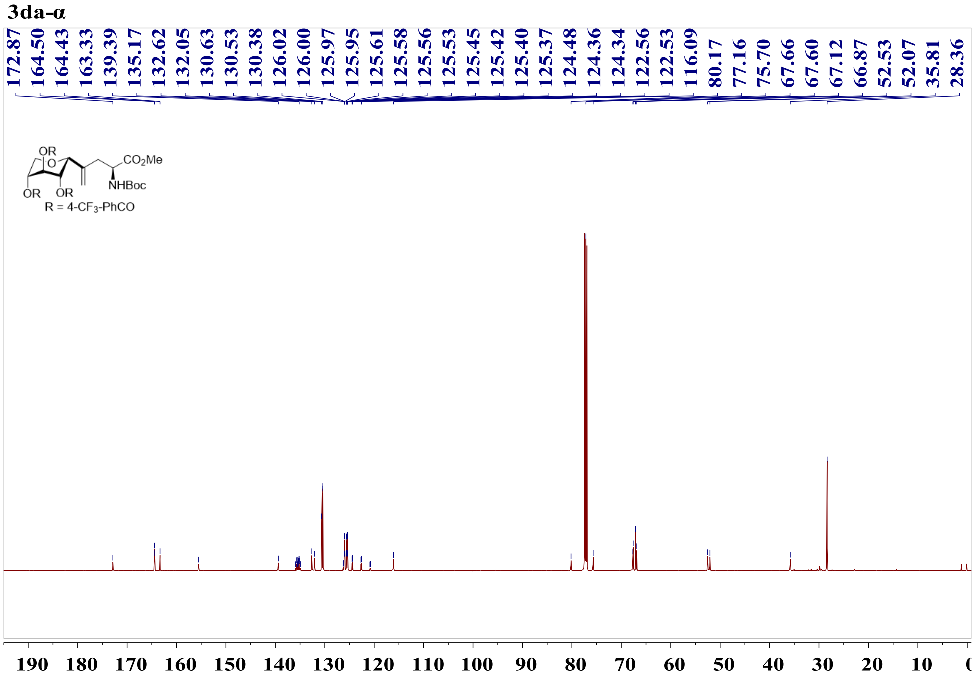

Supplementary Figure 204. ^{1}H-COSY spectrum of compound **3da-α** (500 MHz, $CDCl_3$, 25 ^oC)

Key COSY correlations

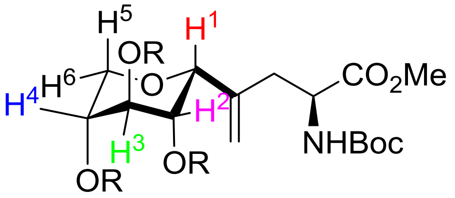

3da-α, $^1C_4(\alpha)$, R = 4-CF$_3$-PhCO

3da-α-COSY

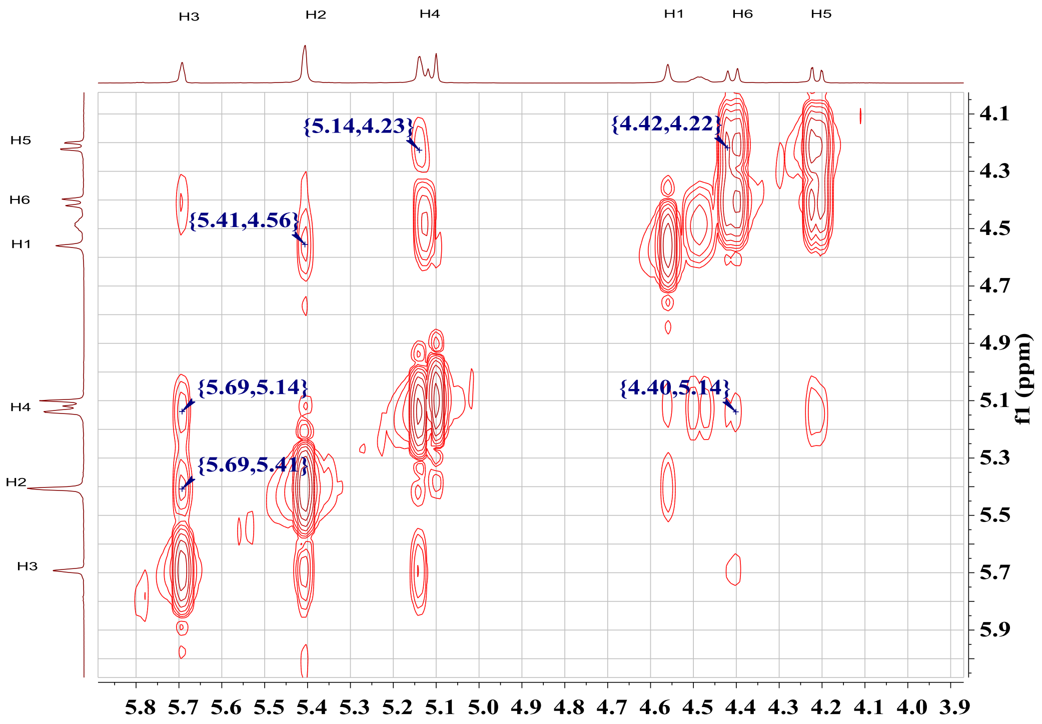

Supplementary Figure 205. HSQC NMR spectrum of compound **3da-α** ($CDCl_3$)

Key HSQC correlations

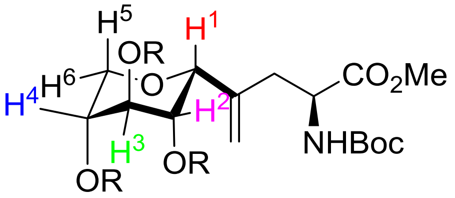

3da-α, $^{1}C_4$(α), R = 4-CF_3-PhCO

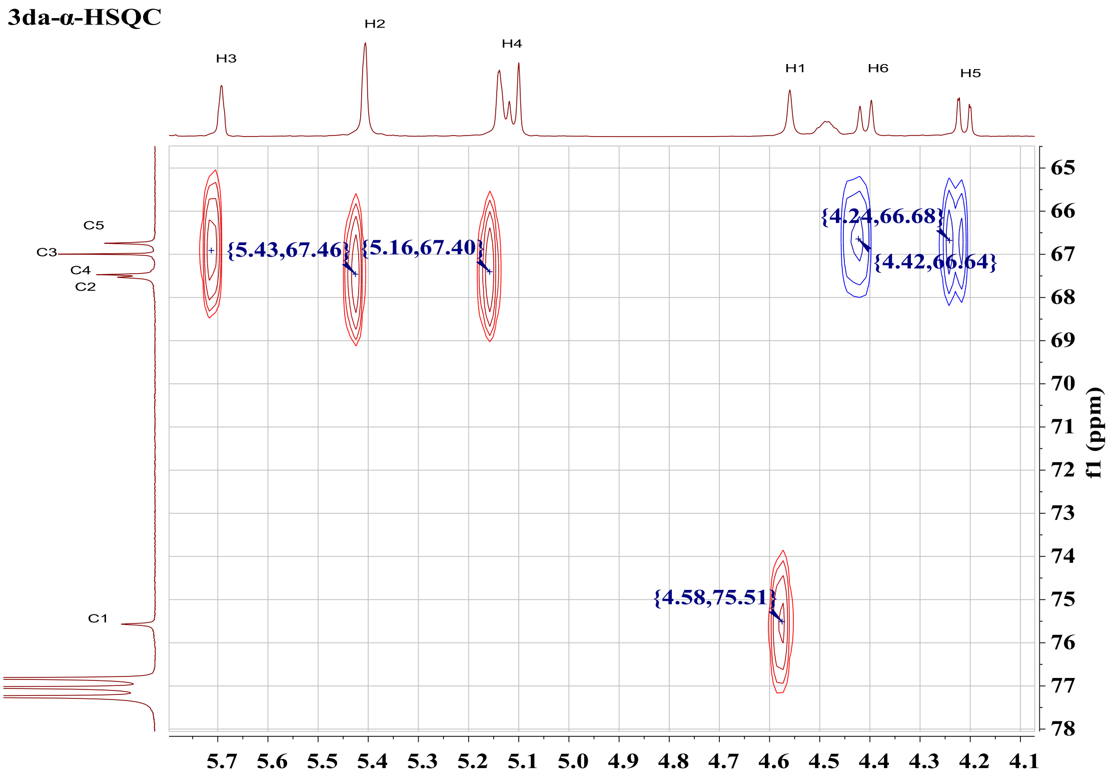

Supplementary Figure 206. NOE NMR spectrum of compound **3da-α** ($CDCl_3$)

Key NOE correlations

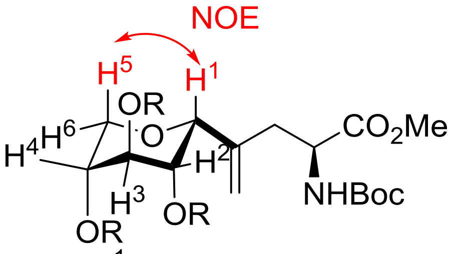

3da-α, $^{1}C_4(\alpha)$, R = 4-CF$_3$-PhCO

3da-α-NOE

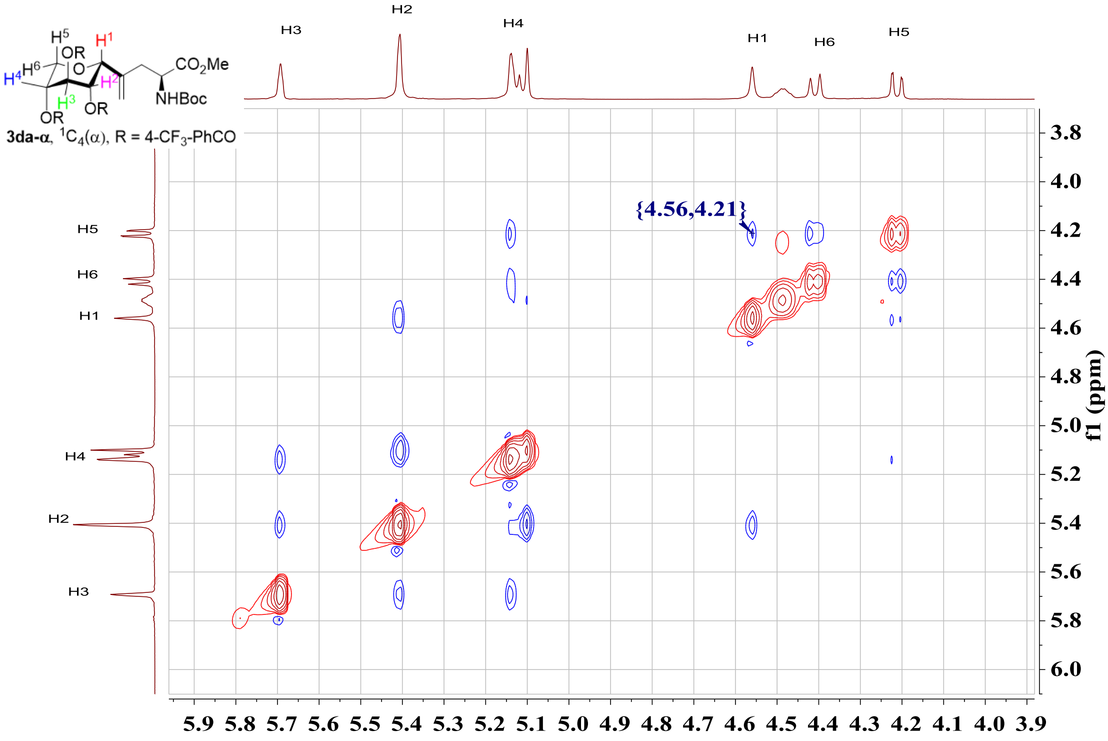

Supplementary Figure 207. ^{1}H NMR spectrum of compound **3ea** (500 MHz, $CDCl_3$, 25 °C)

3ea

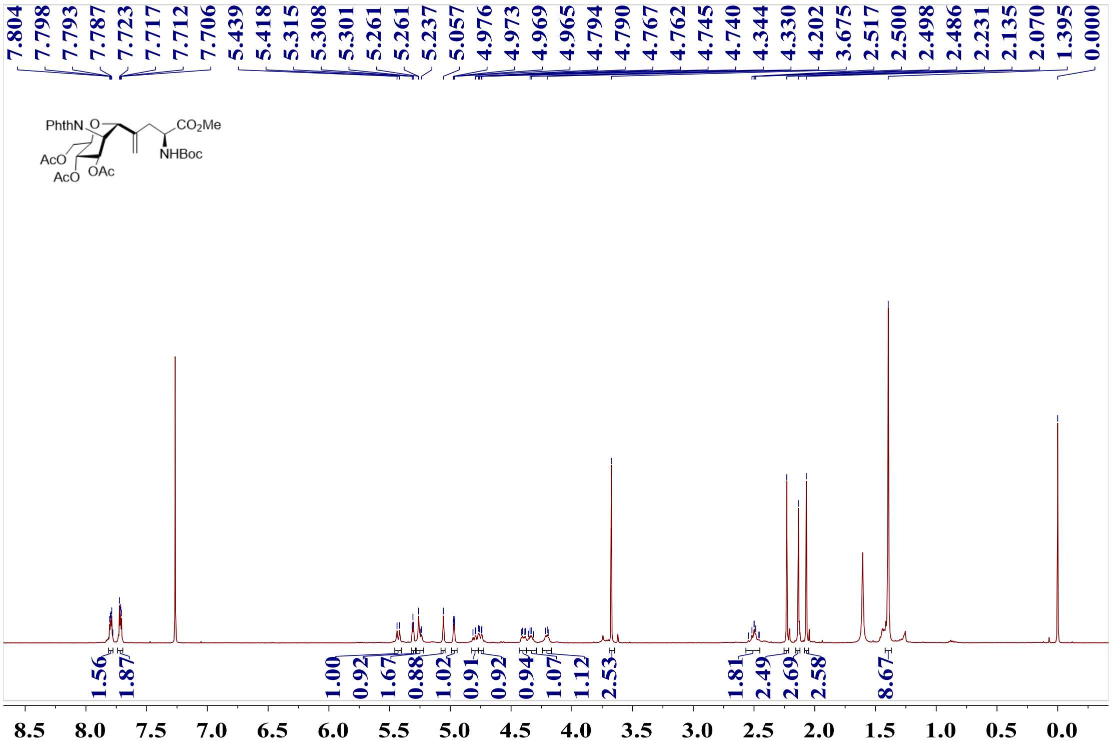

Supplementary Figure 208. ^{13}C NMR spectrum of compound **3ea** (125 MHz, $CDCl_3$, 25 °C)

3ea

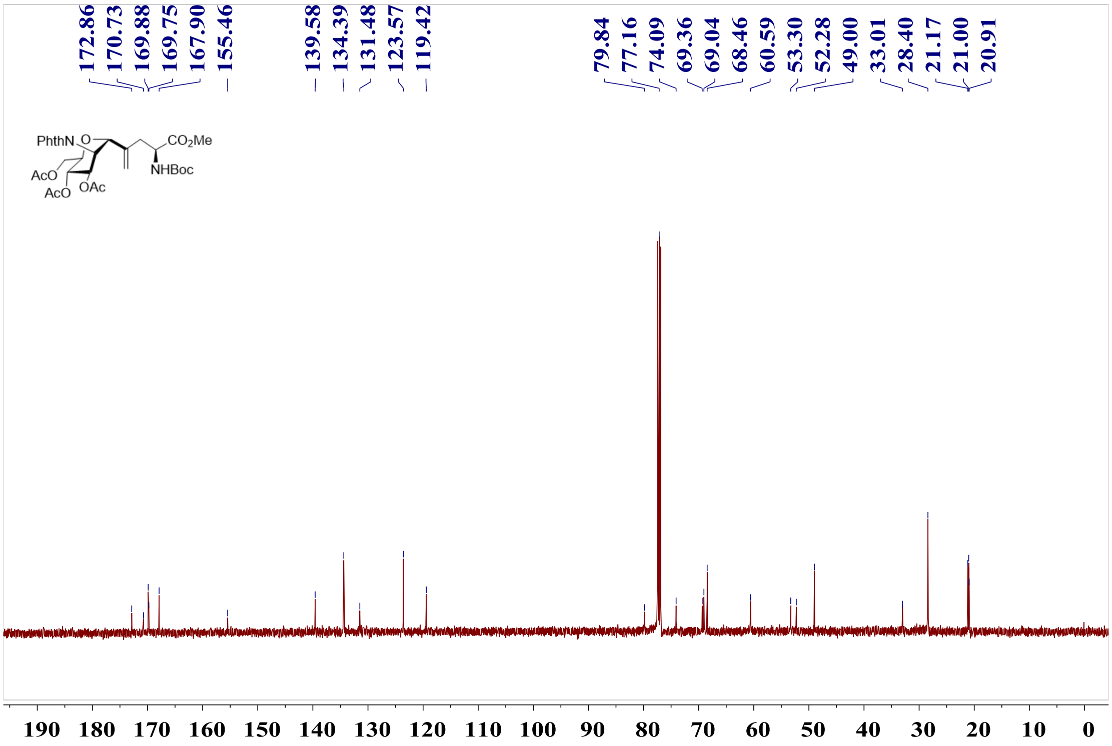

Supplementary Figure 209. ^{1}H NMR spectrum of compound **3fa** (500 MHz, $CDCl_3$, 25 °C)

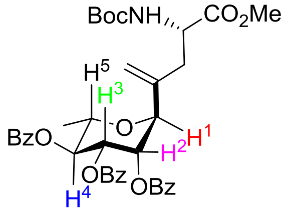

3fa

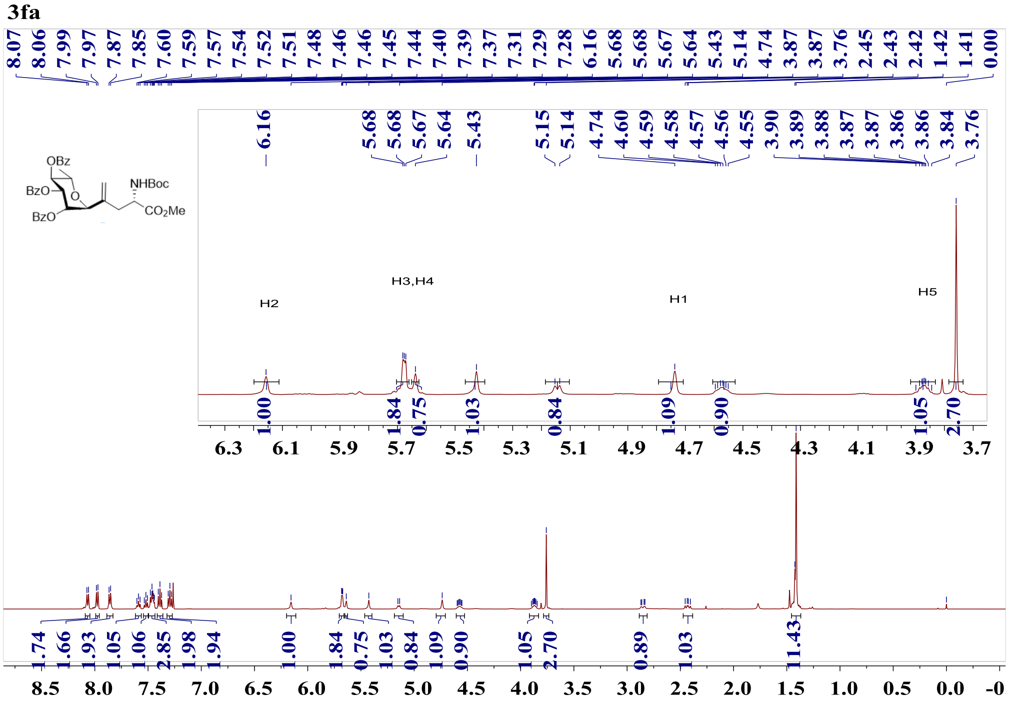

Supplementary Figure 210. ^{13}C NMR spectrum of compound **3fa** (125 MHz, $CDCl_3$, 25 °C)

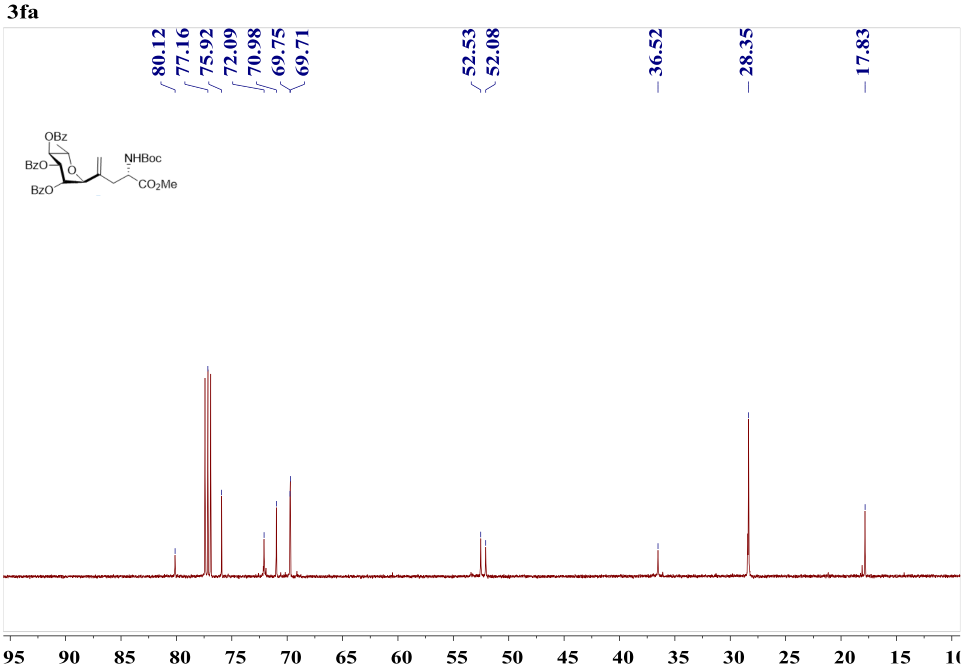

Supplementary Figure 211. ^{1}H COSY NMR spectrum of compound **3fa** (500 MHz, $CDCl_3$, 25 °C)

Key COSY correlations

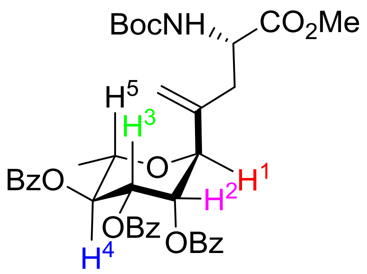

3fa

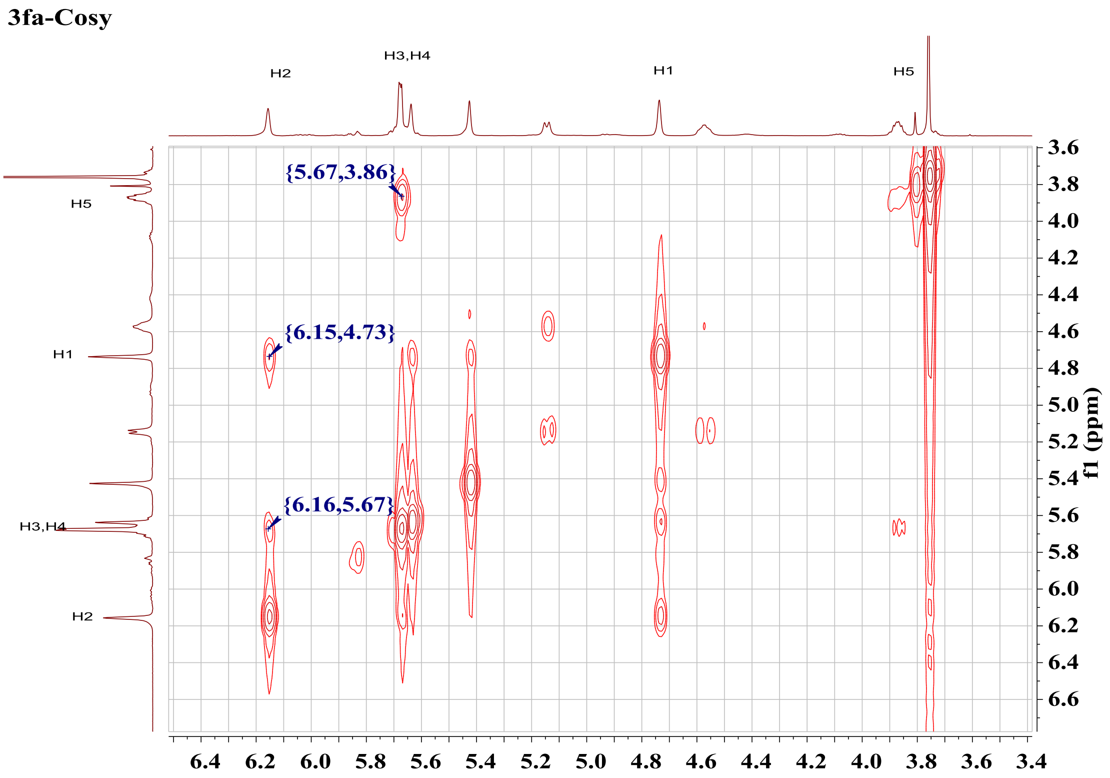

Supplementary Figure 212. HSQC NMR spectrum of compound **3fa** ($CDCl_3$)

Key HSQC correlations

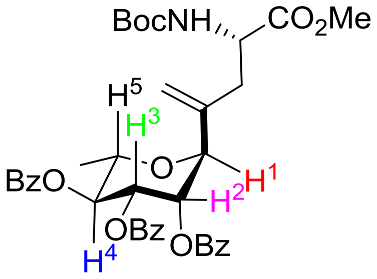

3fa

3fa-HSQC

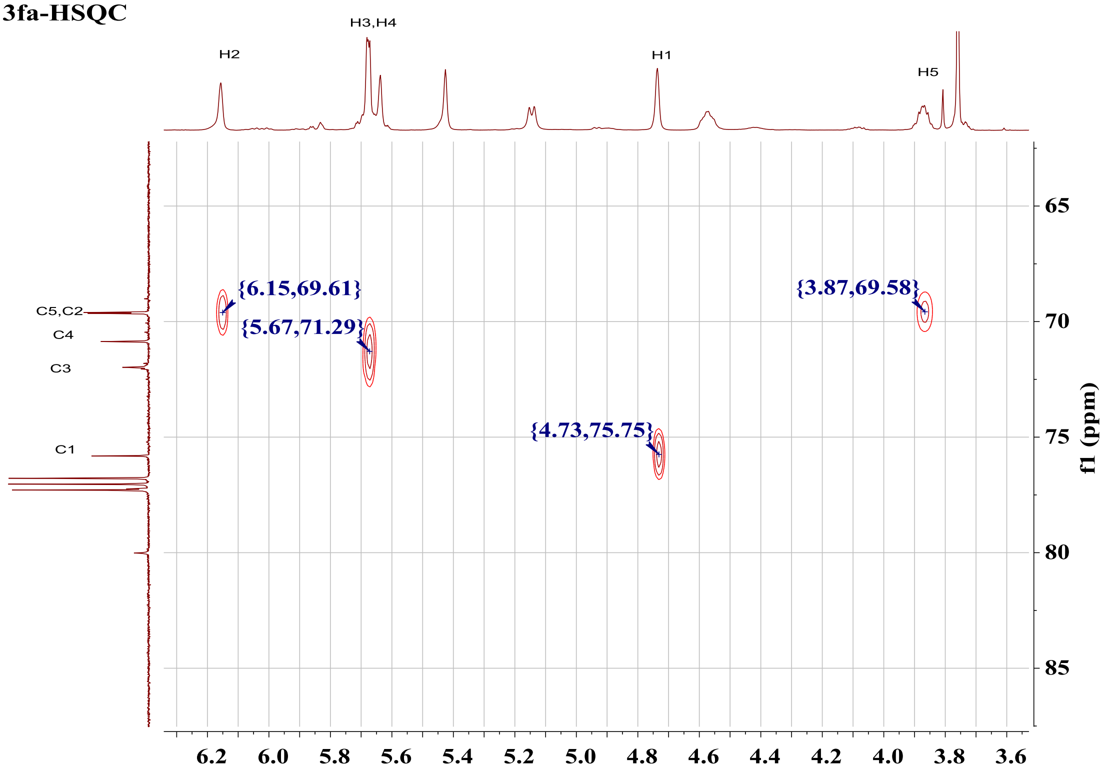

Supplementary Figure 213. ^{1}H NMR spectrum of compound **3ga** (500 MHz, $CDCl_3$, 25 °C)

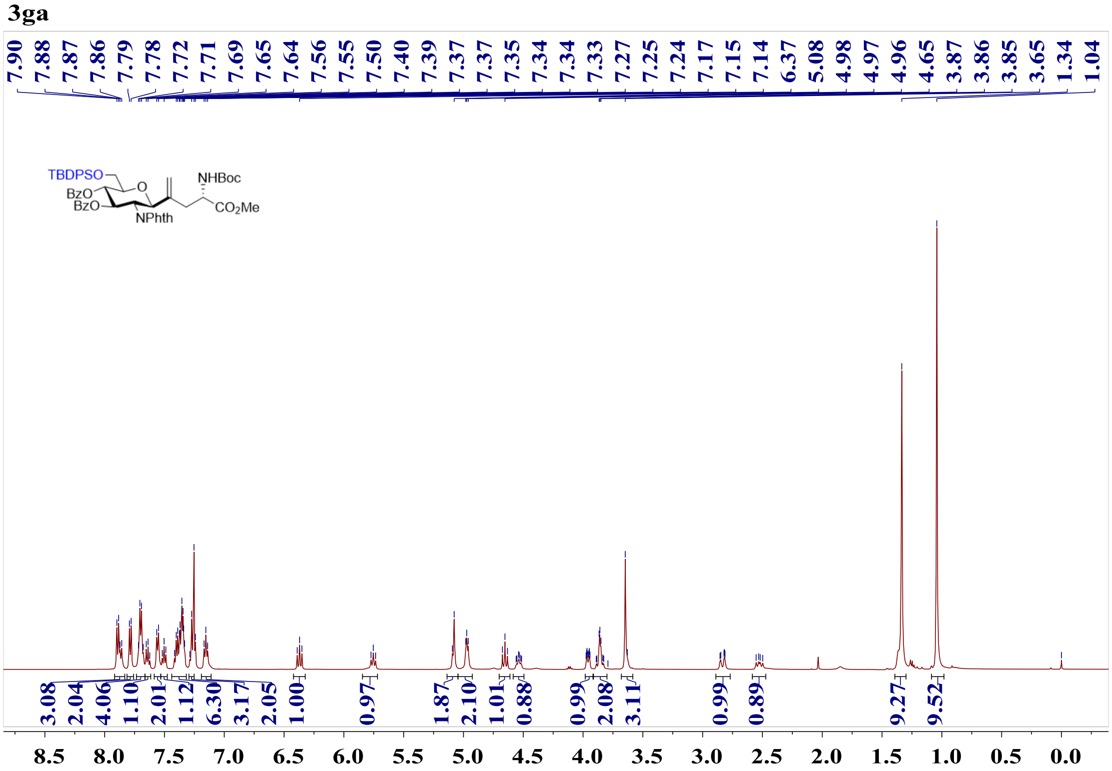

Supplementary Figure 214. ^{13}C NMR spectrum of compound **3ga** (125 MHz, $CDCl_3$, 25 °C)

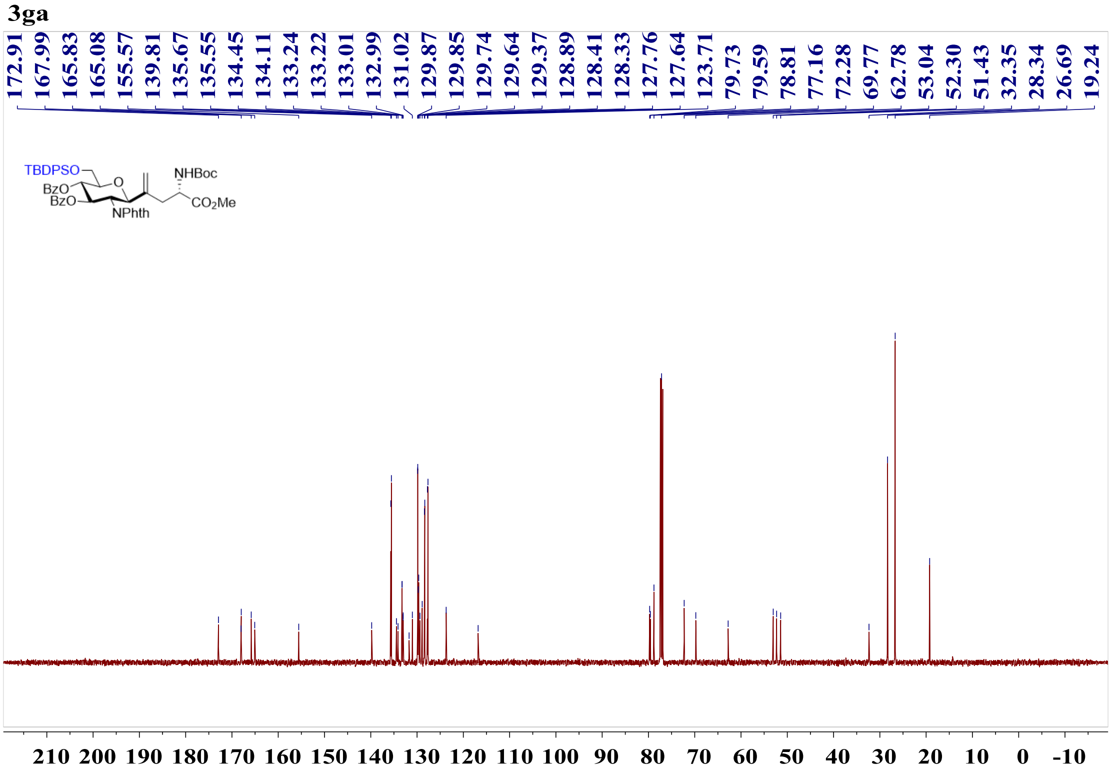

Supplementary Figure 215. ^{1}H NMR spectrum of compound **3ha** (500 MHz, $CDCl_3$, 25 °C)

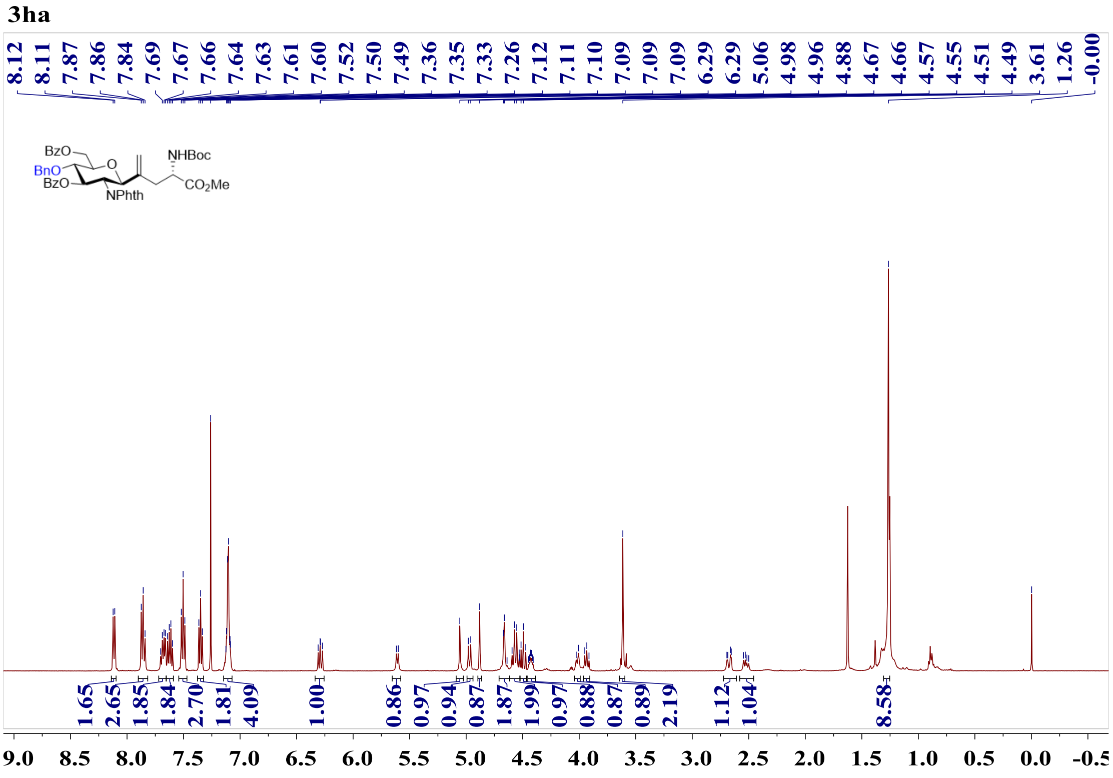

Supplementary Figure 216. ^{13}C NMR spectrum of compound **3ha** (125 MHz, $CDCl_3$, 25 °C)

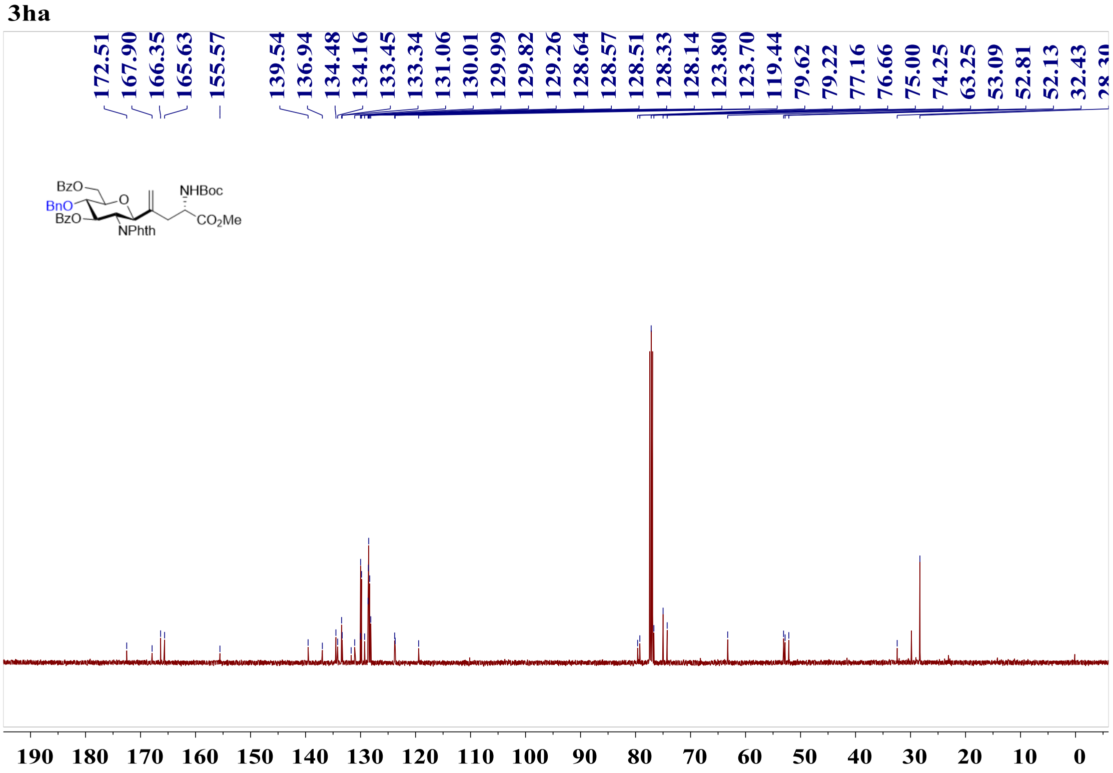

Supplementary Figure 217. ^{1}H NMR spectrum of compound **3iaβ** (500 MHz, $CDCl_3$, 25 °C)

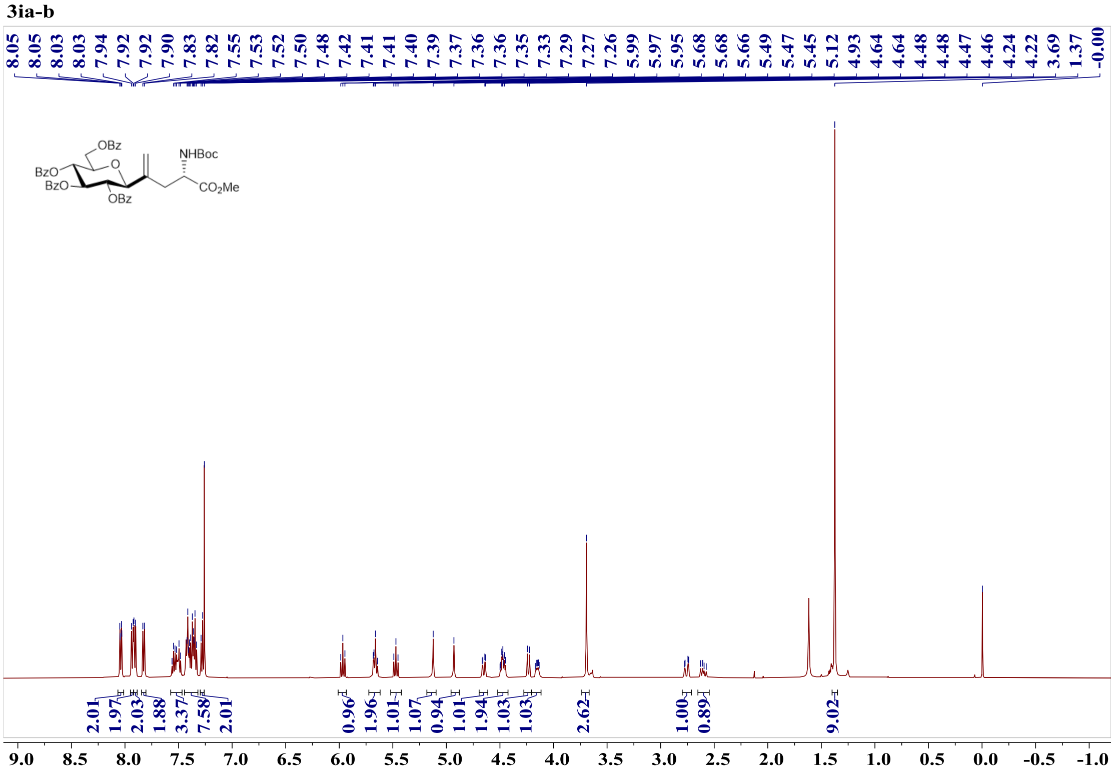

Supplementary Figure 218. ^{13}C NMR spectrum of compound **3iaβ** (125 MHz, $CDCl_3$, 25 °C)

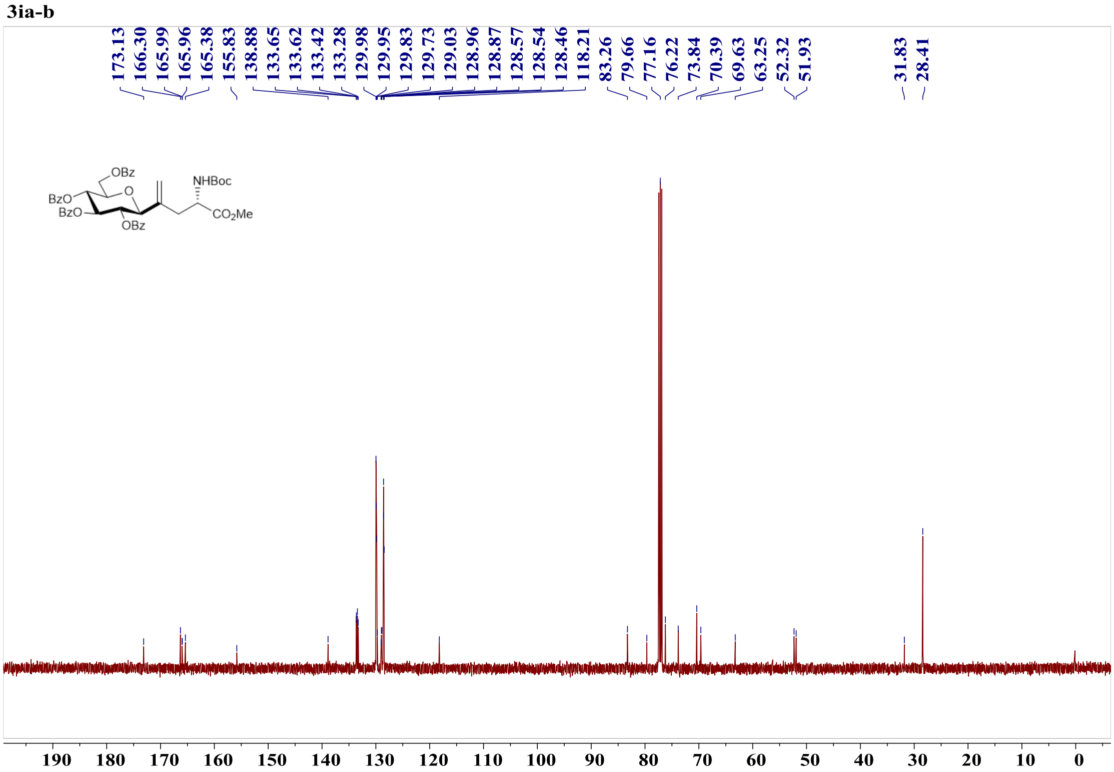

Supplementary Figure 219. ^{1}H NMR spectrum of compound **3iaα** (500 MHz, $CDCl_3$, 25 °C)

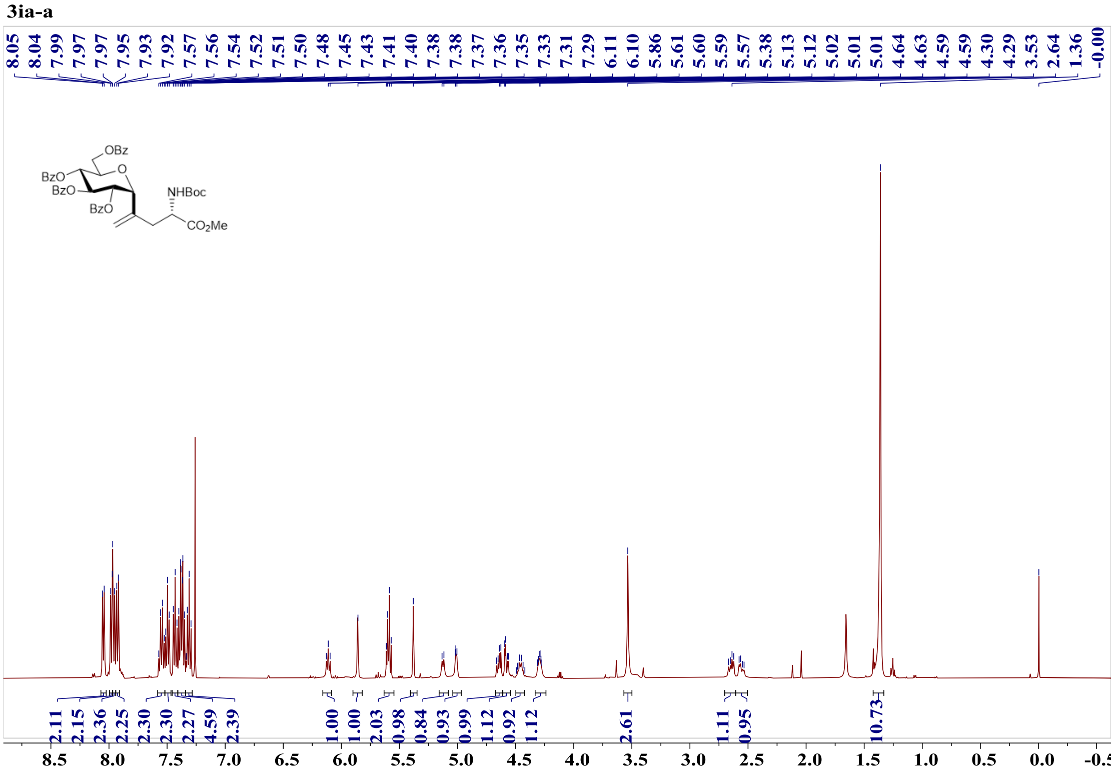

Supplementary Figure 220. ^{13}C NMR spectrum of compound **3iaα** (125 MHz, $CDCl_3$, 25 °C)

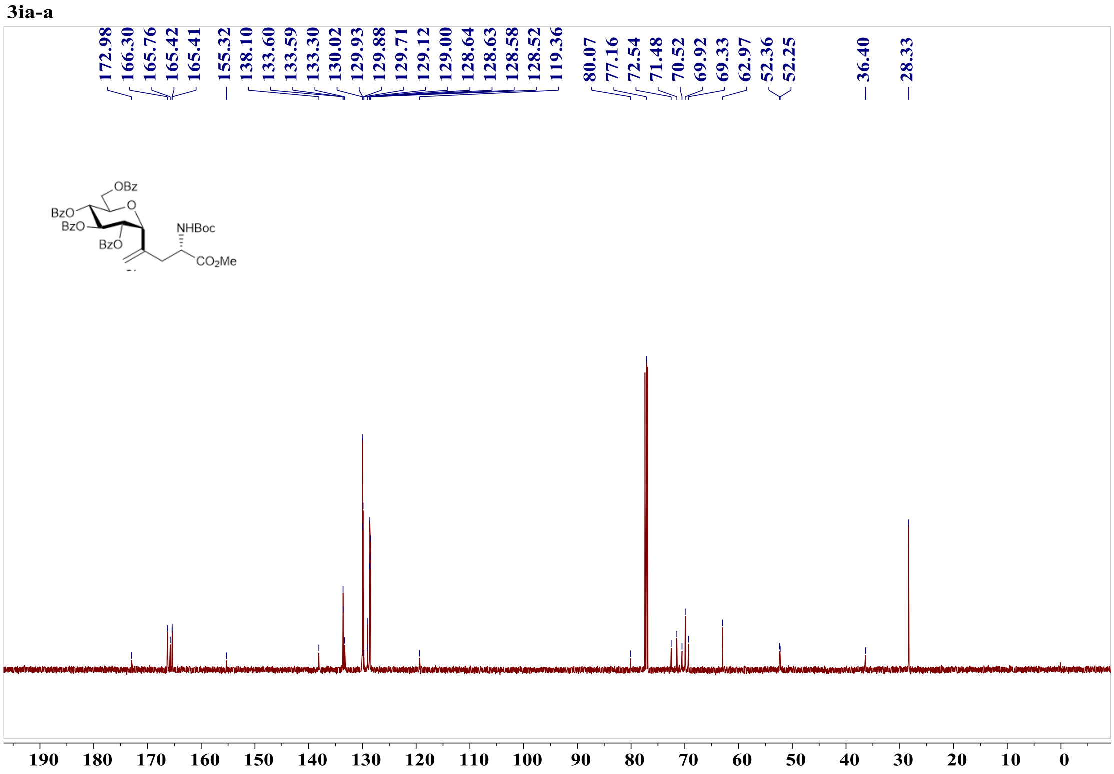

Supplementary Figure 221. ^{1}H NMR spectrum of compound **3ja** (500 MHz, $CDCl_3$, 25 °C)

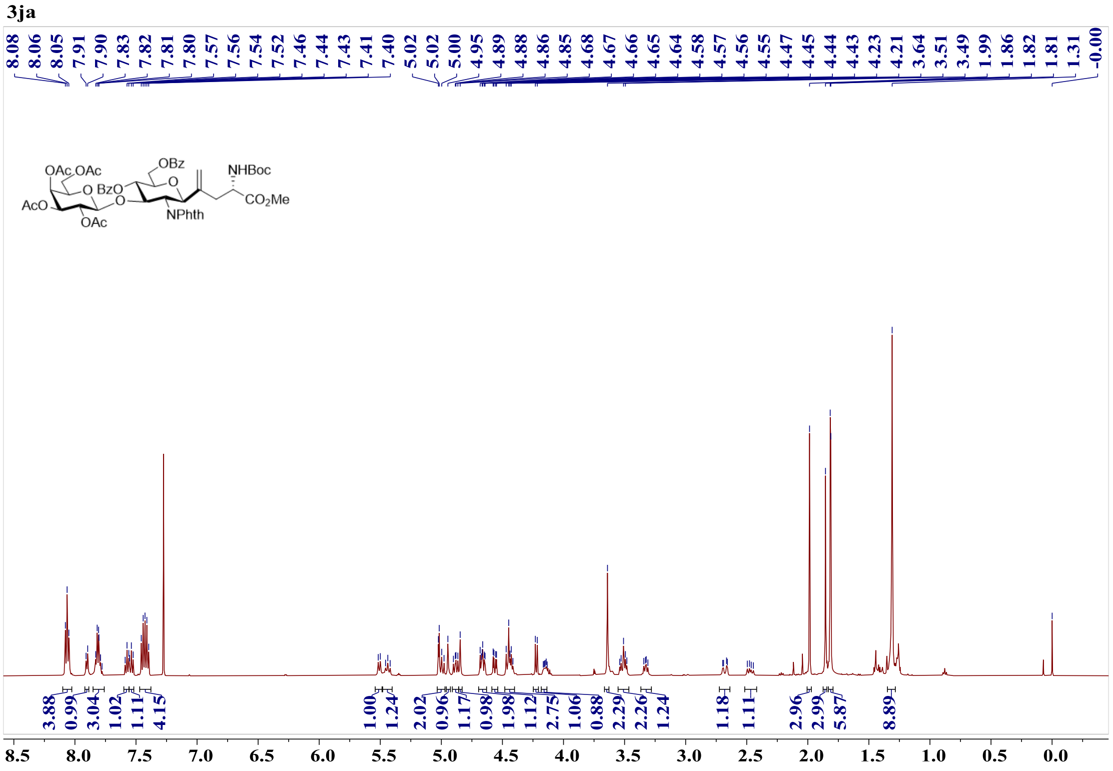

Supplementary Figure 222. ^{13}C NMR spectrum of compound **3ja** (125 MHz, $CDCl_3$, 25 °C)

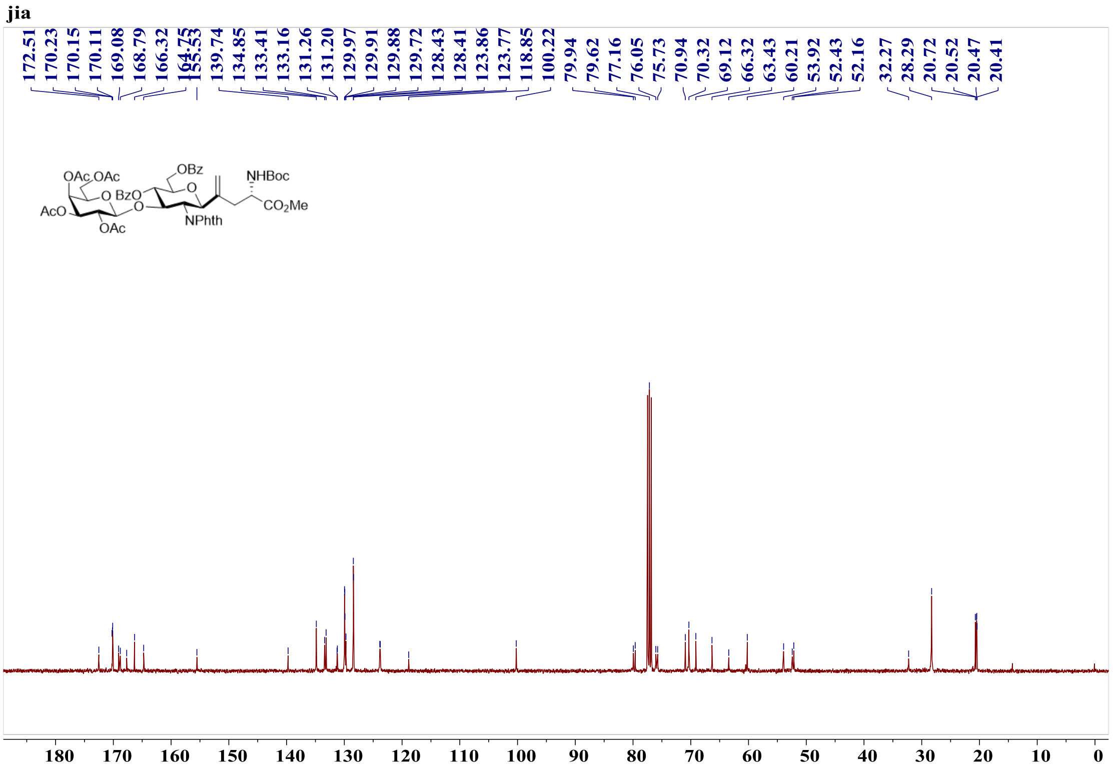

Supplementary Figure 223. ^{1}H NMR spectrum of compound **3ka** (500 MHz, $CDCl_3$, 25 ^{o}C)

3ka

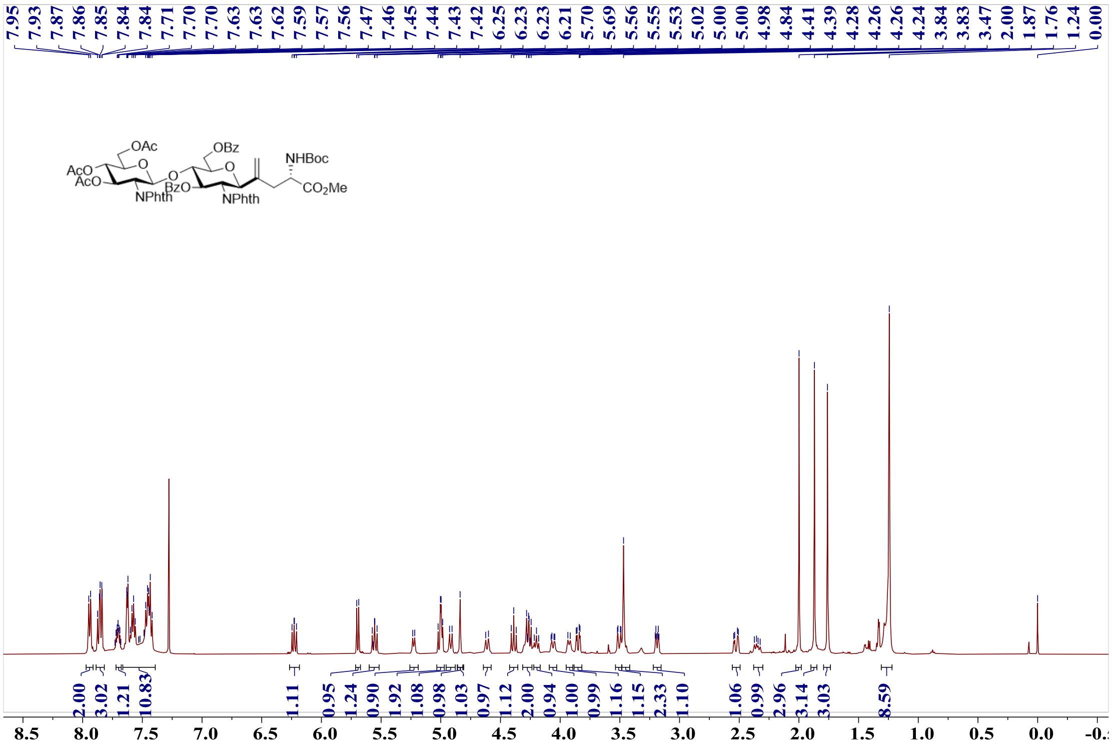

Supplementary Figure 224. ^{13}C NMR spectrum of compound **3ka** (125 MHz, $CDCl_3$, 25 ^{o}C)

3ka

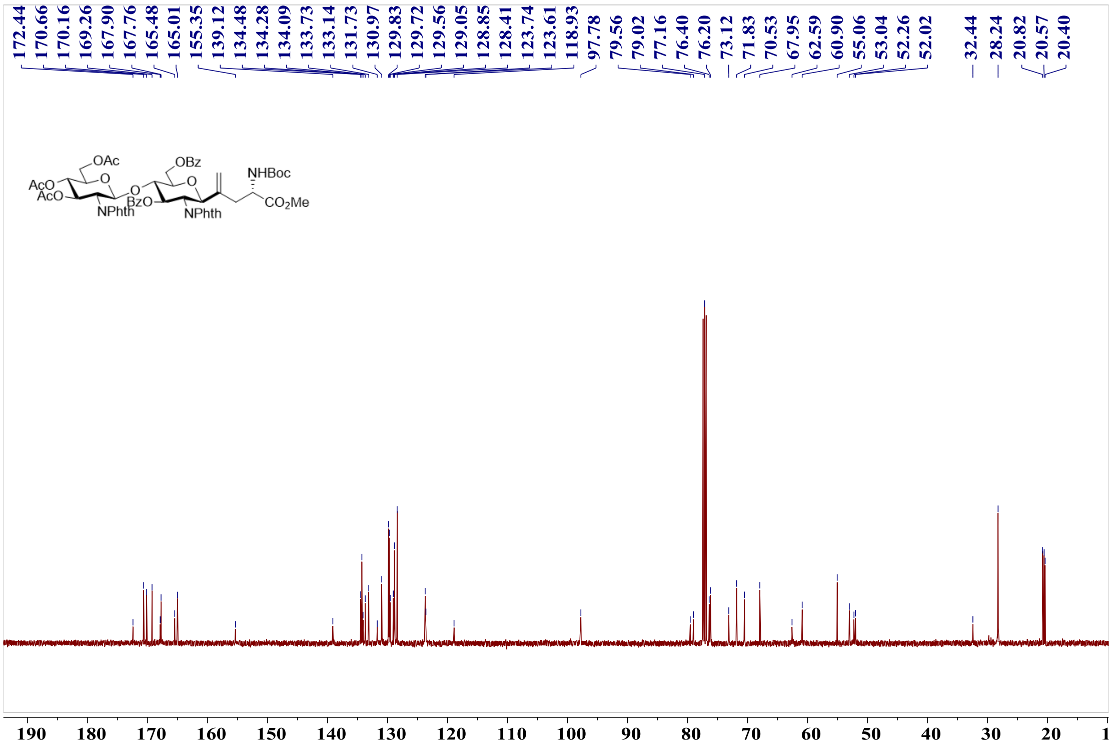

Supplementary Figure 225. ^{1}H NMR spectrum of compound **3la** (500 MHz, $CDCl_3$, 25 ^oC)

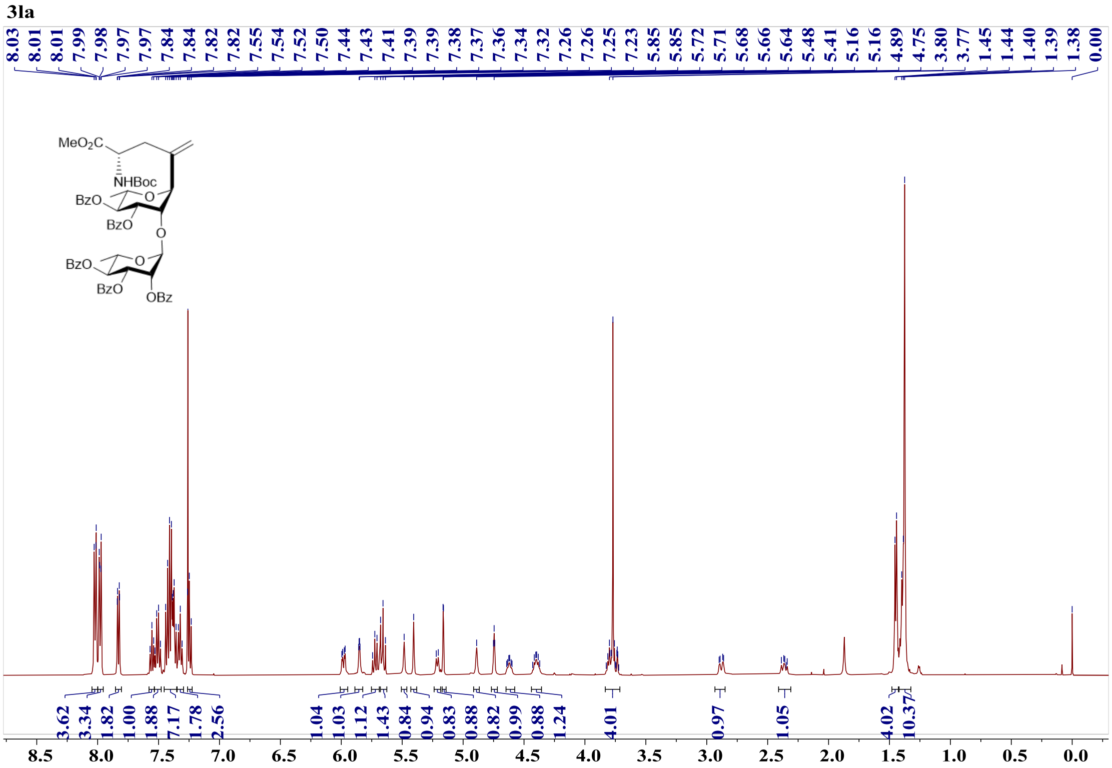

Supplementary Figure 226. ^{13}C NMR spectrum of compound **3la** (125 MHz, $CDCl_3$, 25 ^oC)

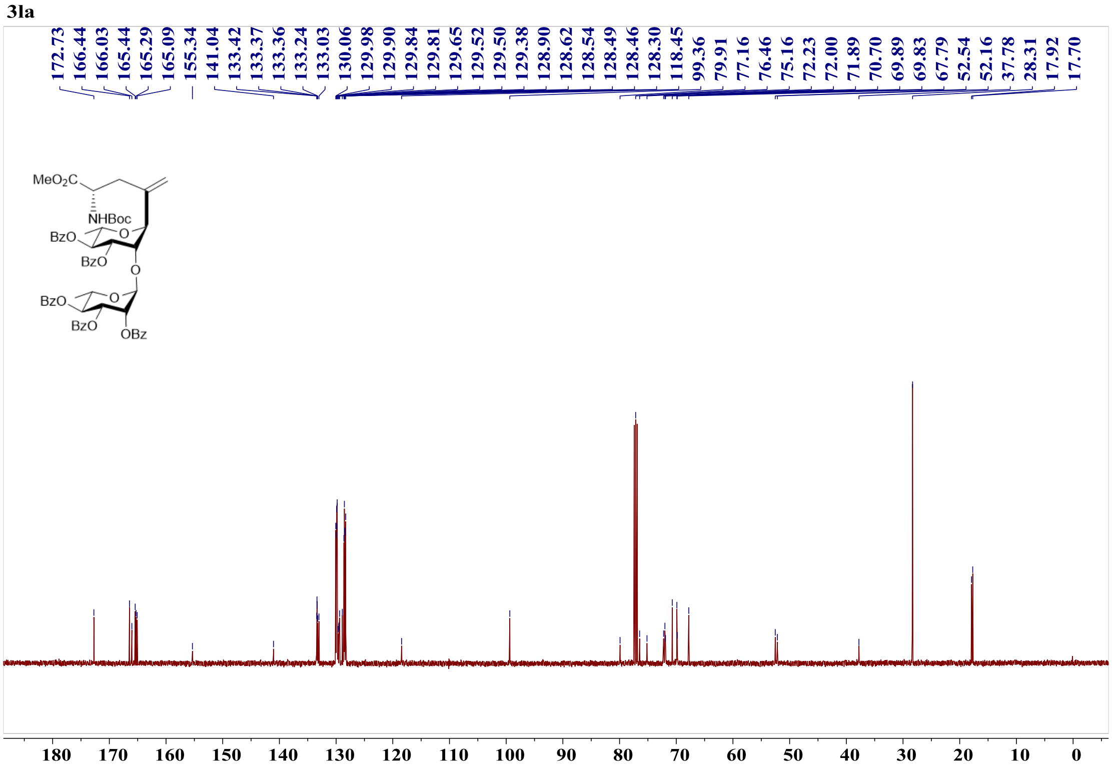

Supplementary Figure 227. ^{1}H NMR spectrum of compound **3ma** (500 MHz, $CDCl_3$, 25 °C)

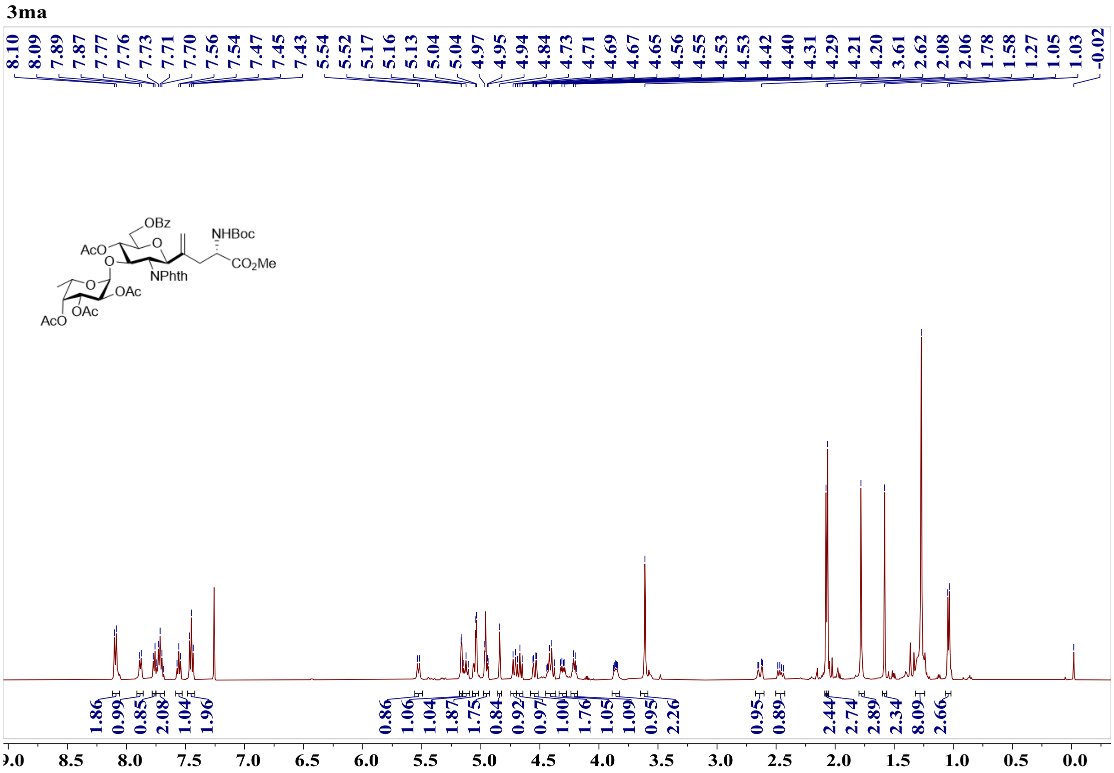

Supplementary Figure 228. ^{13}C NMR spectrum of compound **3ma** (125 MHz, $CDCl_3$, 25 °C)

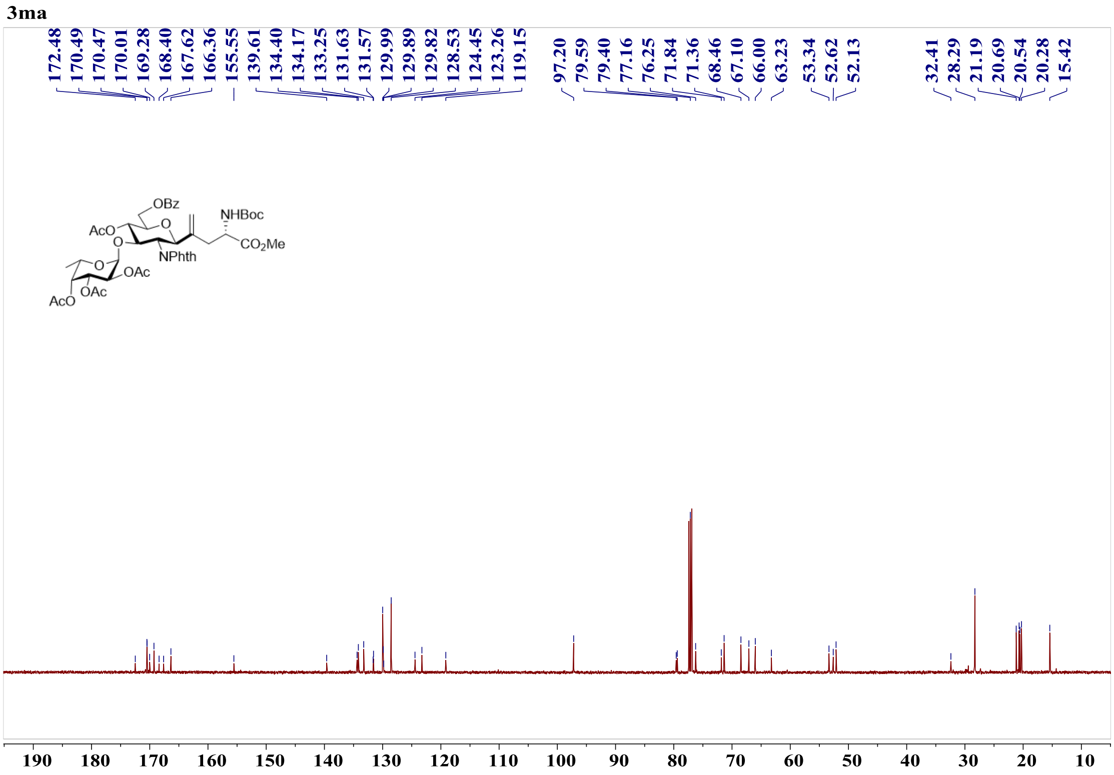

Supplementary Figure 229. ^{1}H NMR spectrum of compound **3av1** (500 MHz, $CDCl_3$, 25 °C)

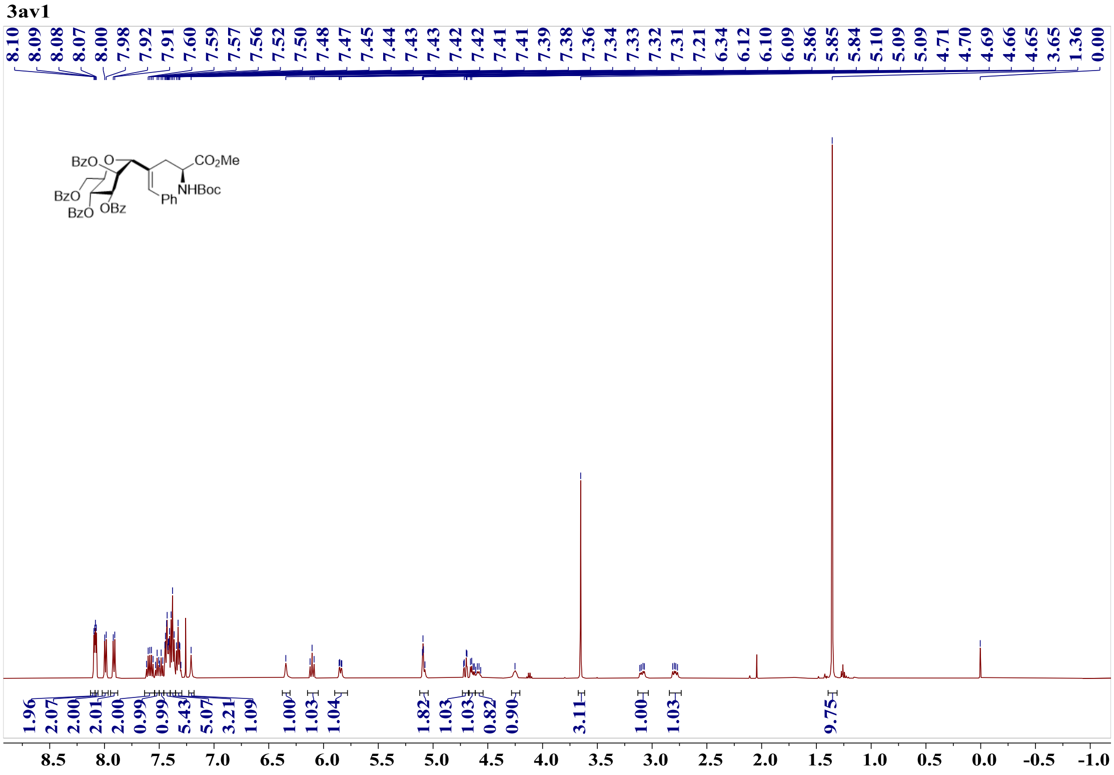

Supplementary Figure 230. ^{13}C NMR spectrum of compound **3av1** (125 MHz, $CDCl_3$, 25 °C)

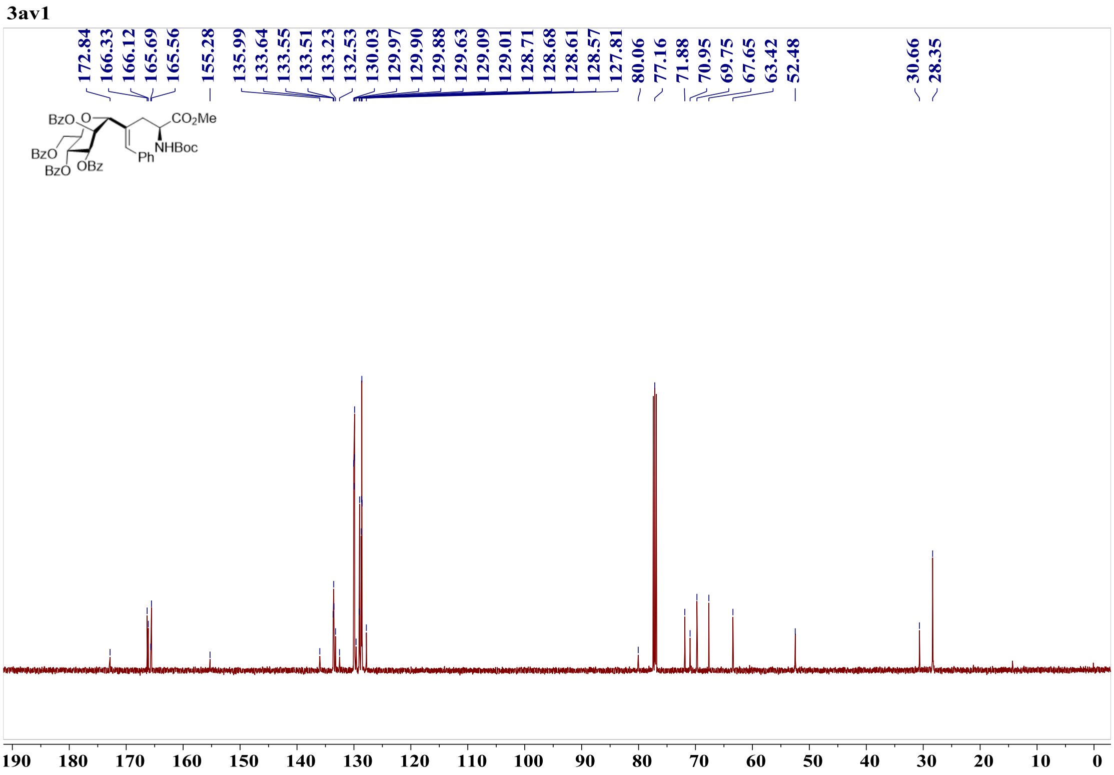

Supplementary Figure 231. ^{1}H NMR spectrum of compound **3av2** (500 MHz, $CDCl_3$, 25 °C)

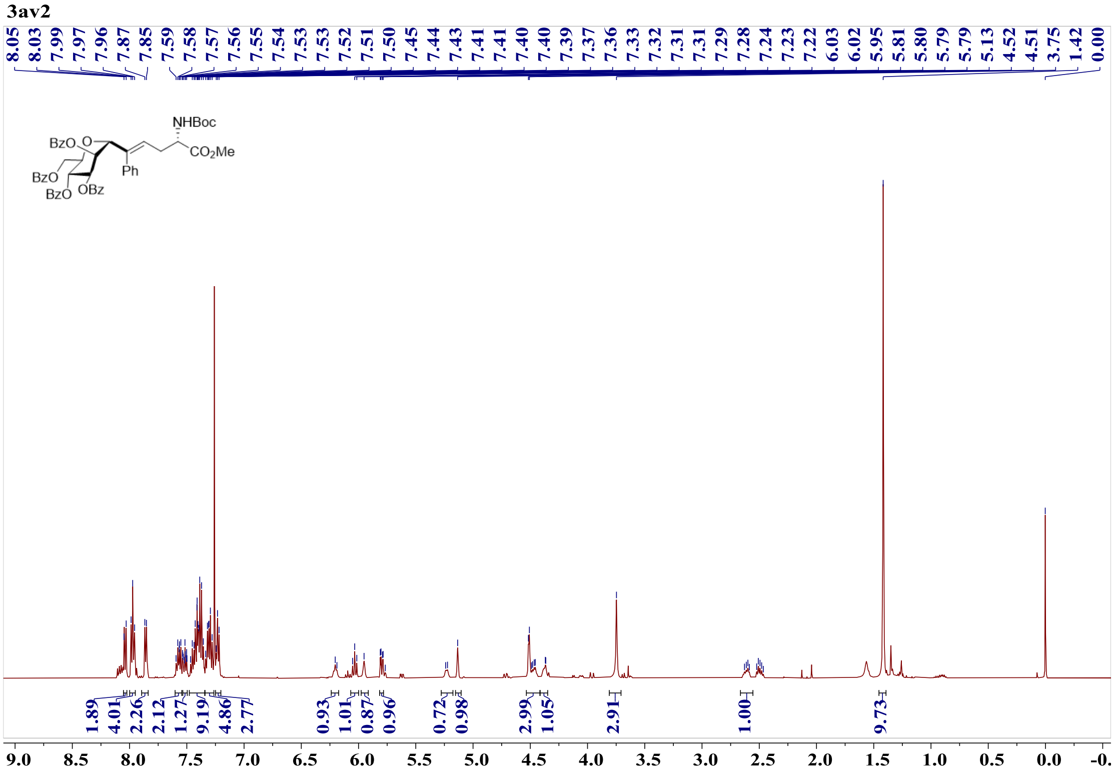

Supplementary Figure 232. ^{13}C NMR spectrum of compound **3av2** (125 MHz, $CDCl_3$, 25 °C)

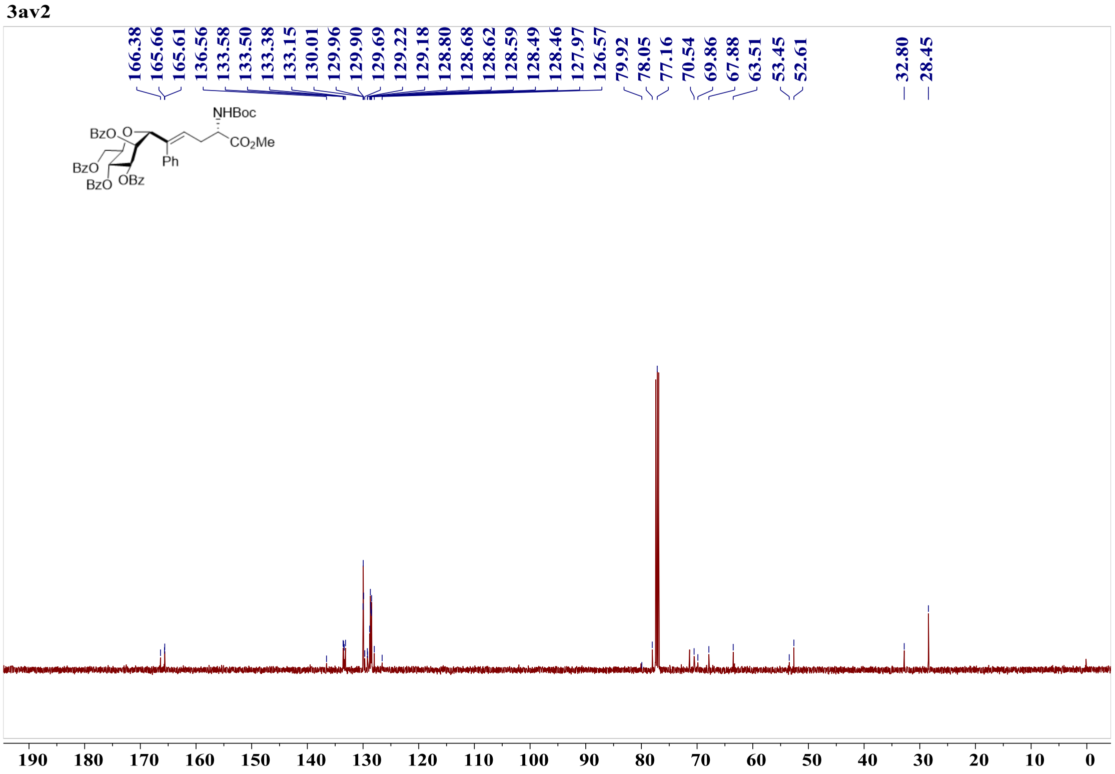

Supplementary Figure 233. ^{1}H NMR spectrum of compound **3bv1** (500 MHz, $CDCl_3$, 25 °C)

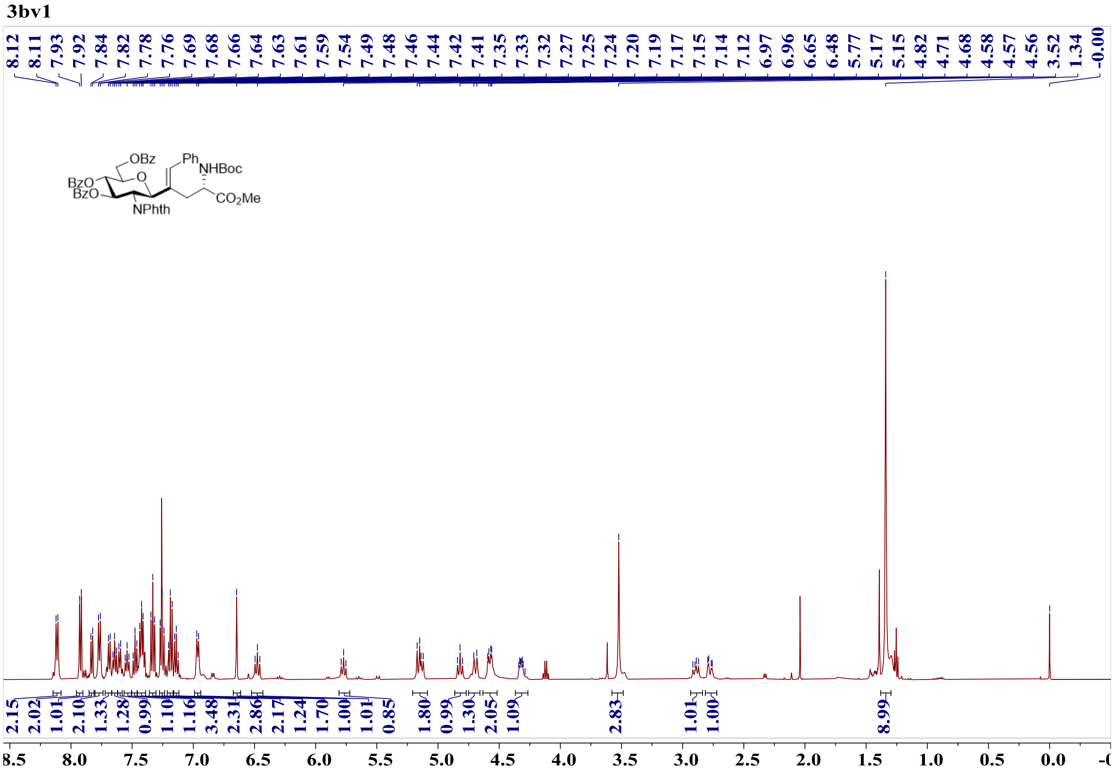

Supplementary Figure 234. ^{13}C NMR spectrum of compound **3bv1** (125 MHz, $CDCl_3$, 25 °C)

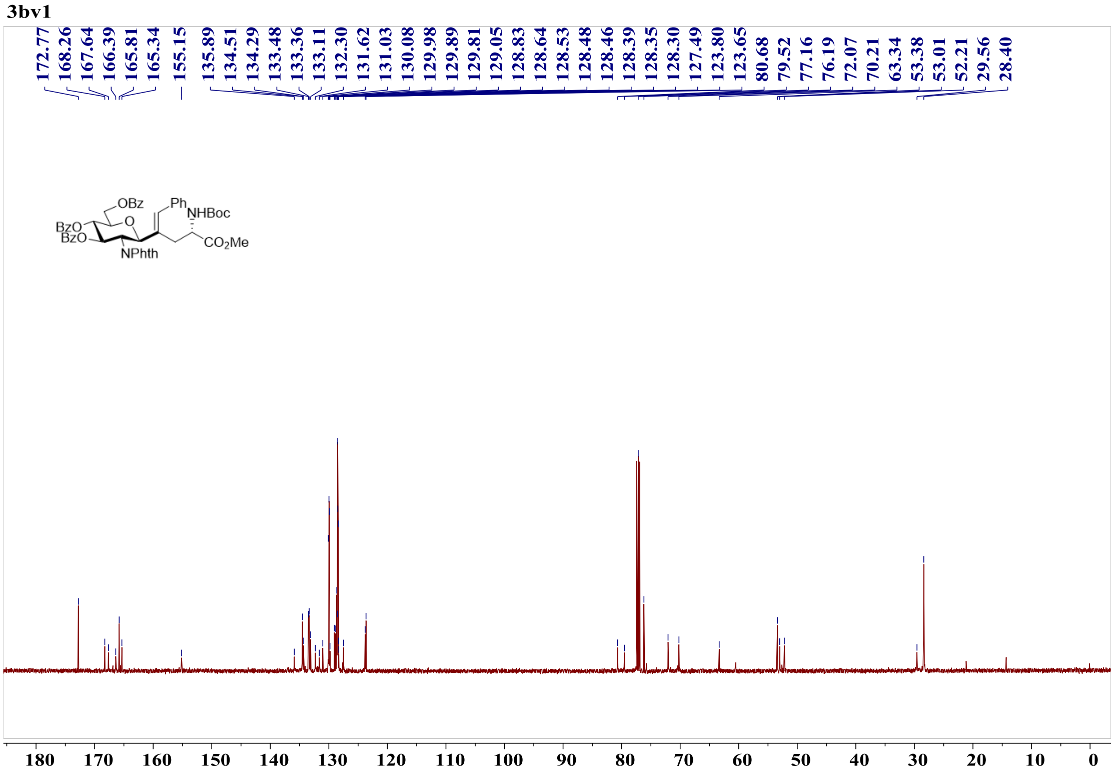

Supplementary Figure 235. ^{1}H NMR spectrum of compound **3bv2** (500 MHz, $CDCl_3$, 25 °C)

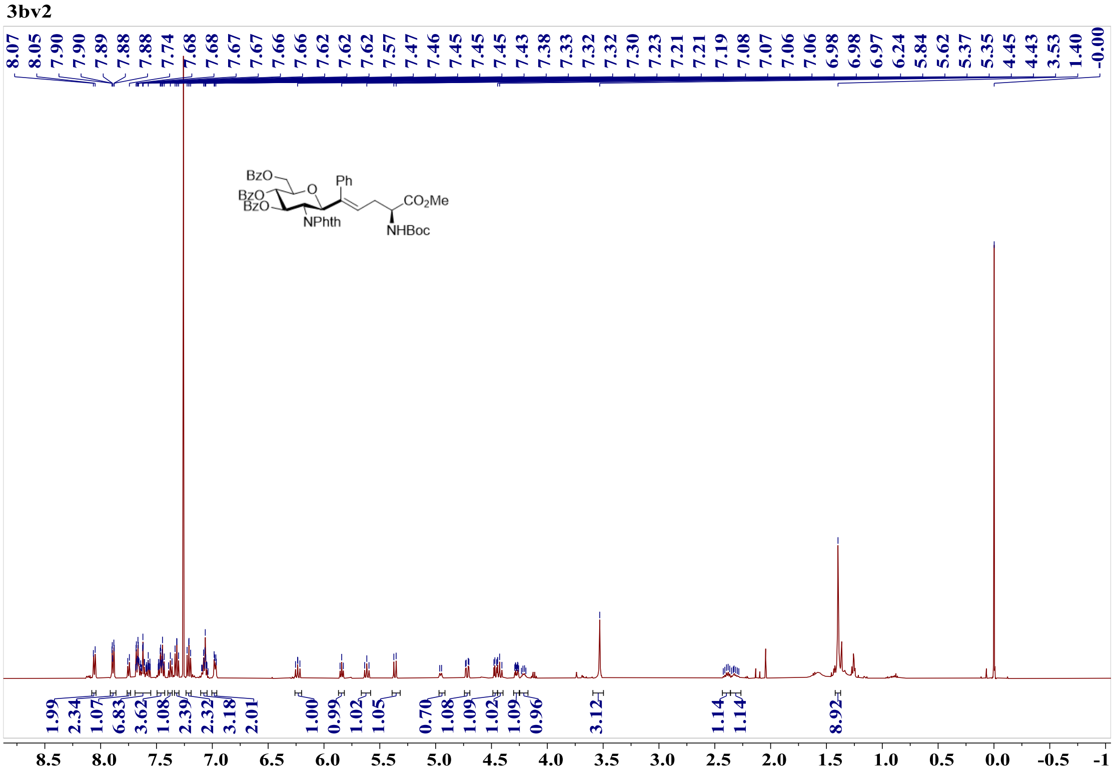

Supplementary Figure 236. ^{13}C NMR spectrum of compound **3bv2** (125 MHz, $CDCl_3$, 25 °C)

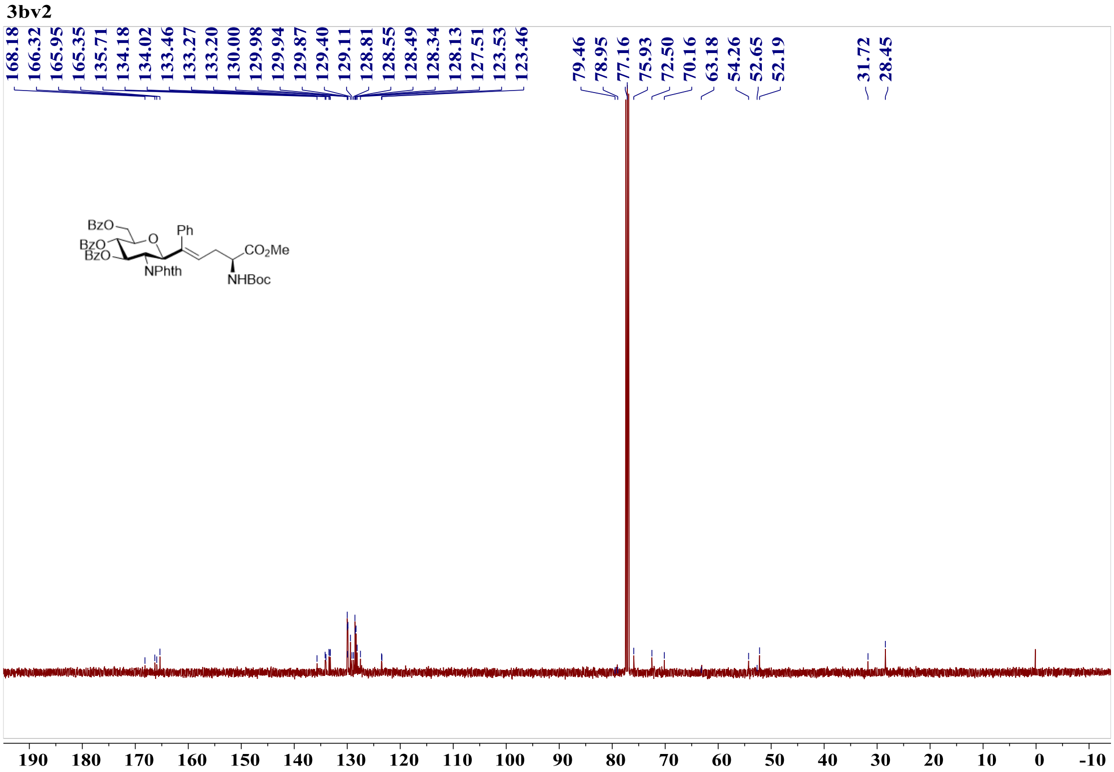

Supplementary Figure 237. ^{1}H NMR spectrum of compound **S41** (500 MHz, $CDCl_3$, 25 ^{o}C)

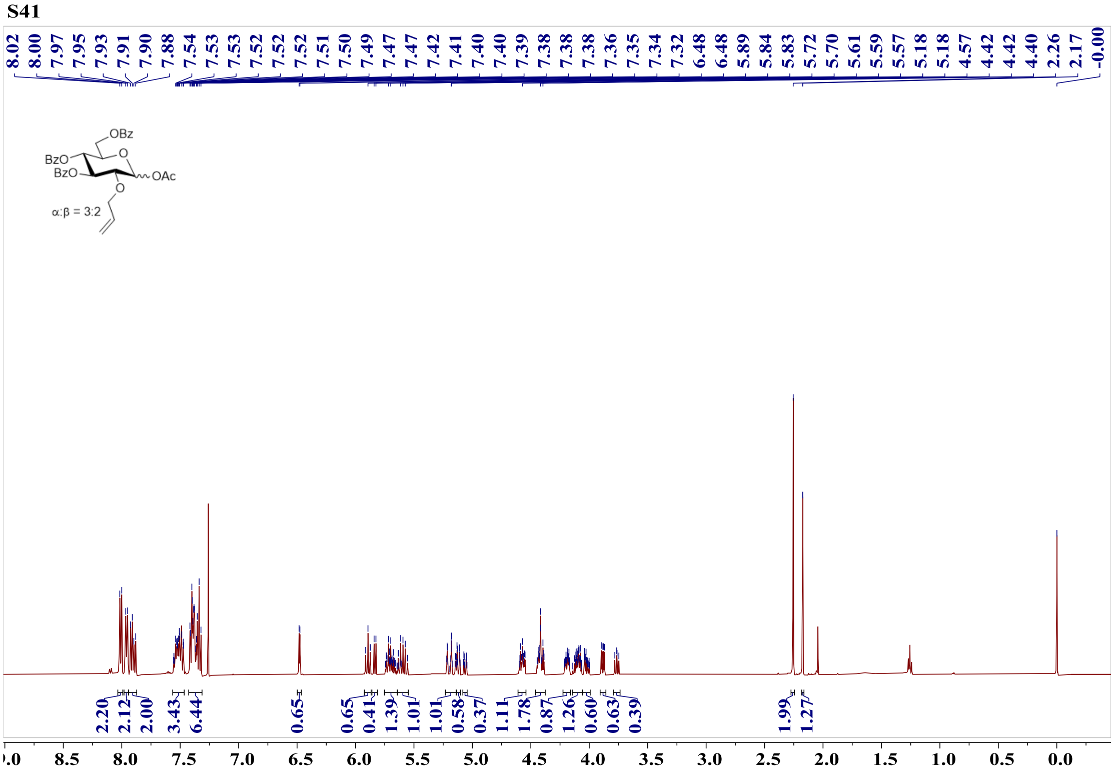

Supplementary Figure 238. ^{13}C NMR spectrum of compound **S41** (125 MHz, $CDCl_3$, 25 ^{o}C)

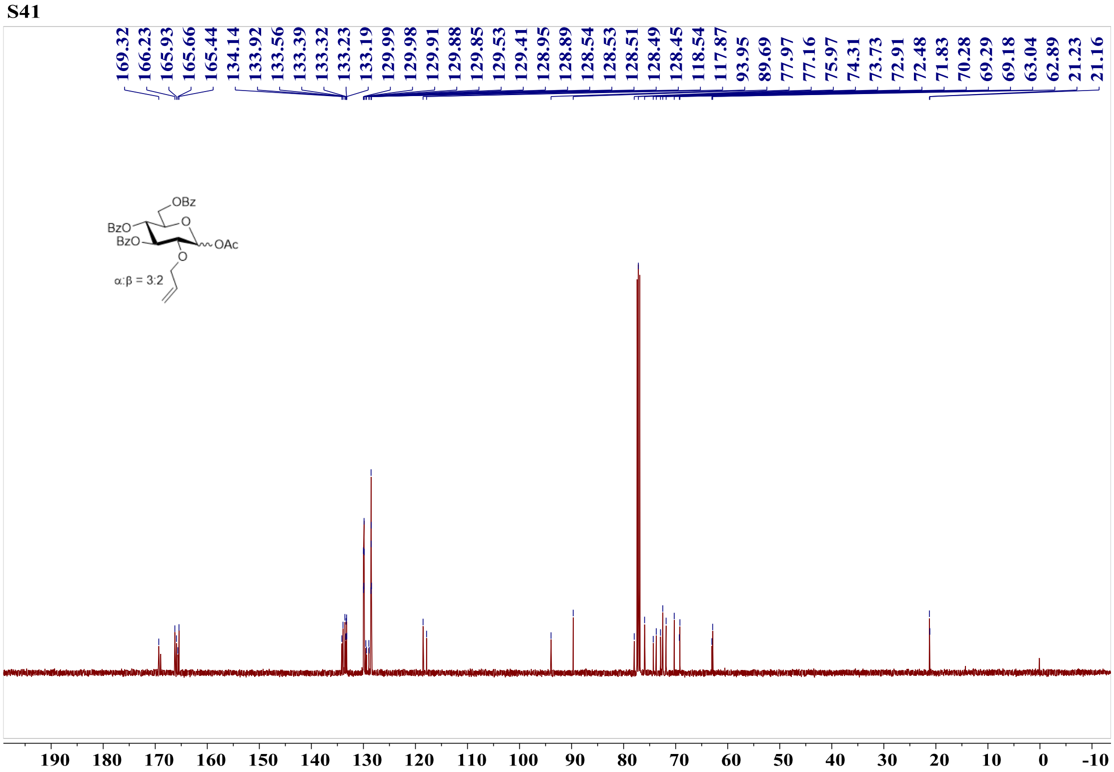

Supplementary Figure 239. ^{1}H NMR spectrum of compound **6** (500 MHz, $CDCl_3$, 25 °C)

6

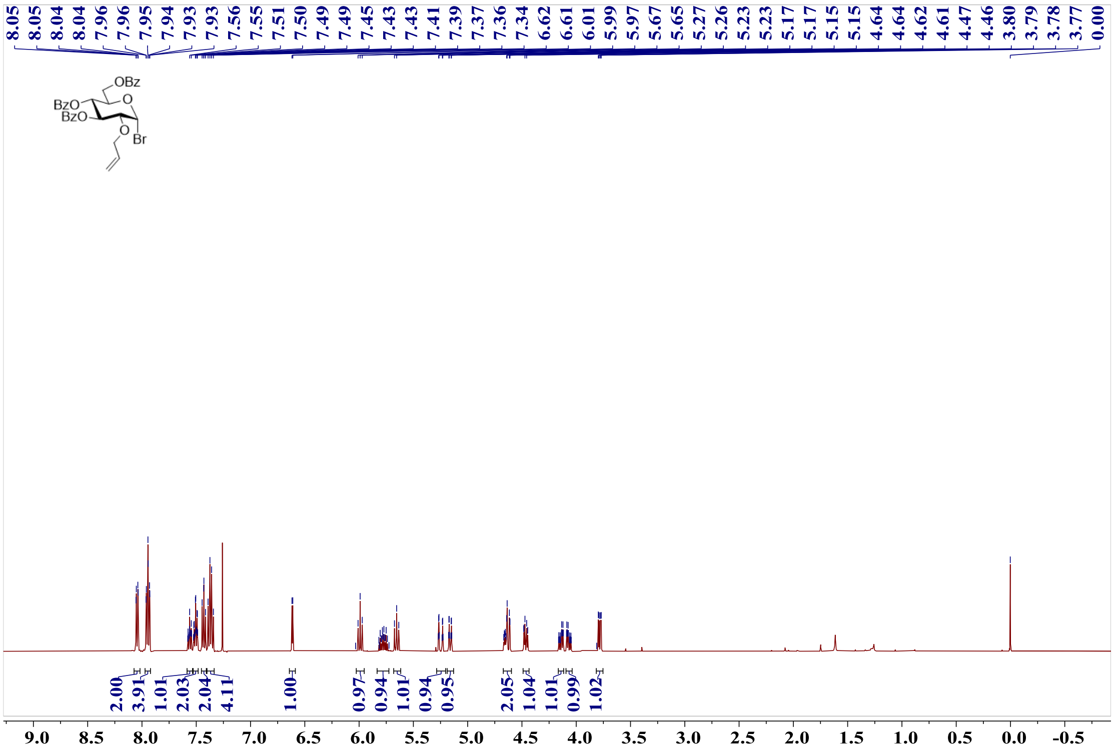

Supplementary Figure 240. ^{13}C NMR spectrum of compound **6** (125 MHz, $CDCl_3$, 25 °C)

6

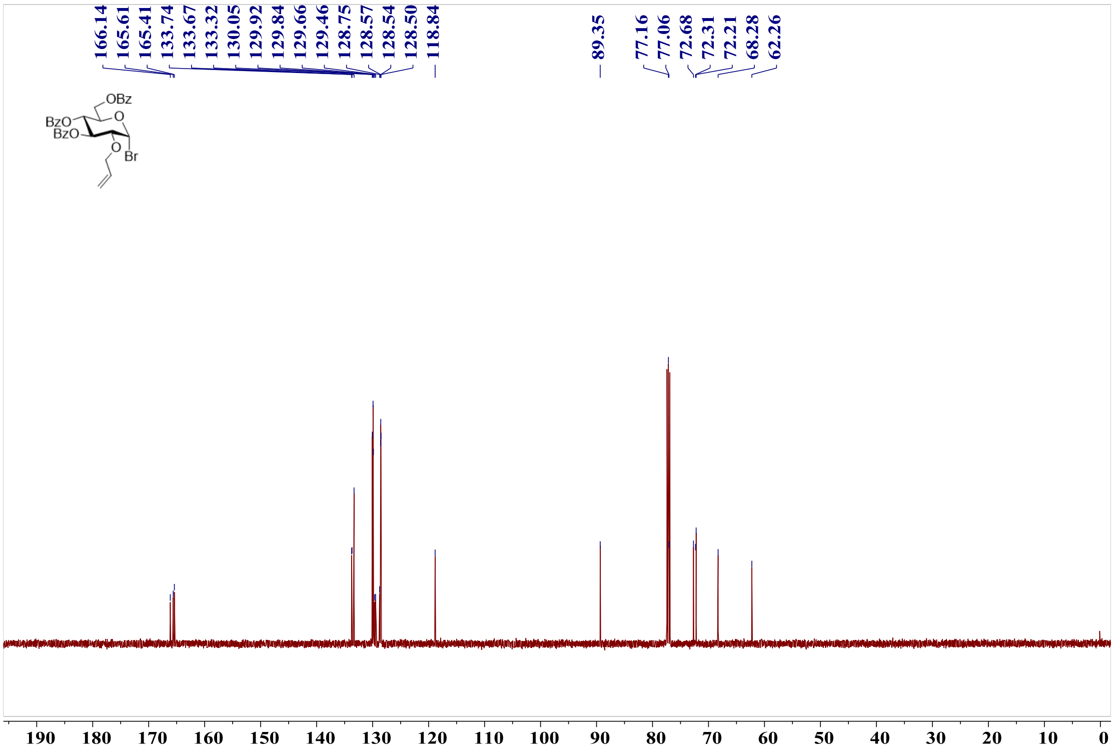

Supplementary Figure 241. ^{1}H NMR spectrum of compound **7** (500 MHz, $CDCl_3$, 25 °C)

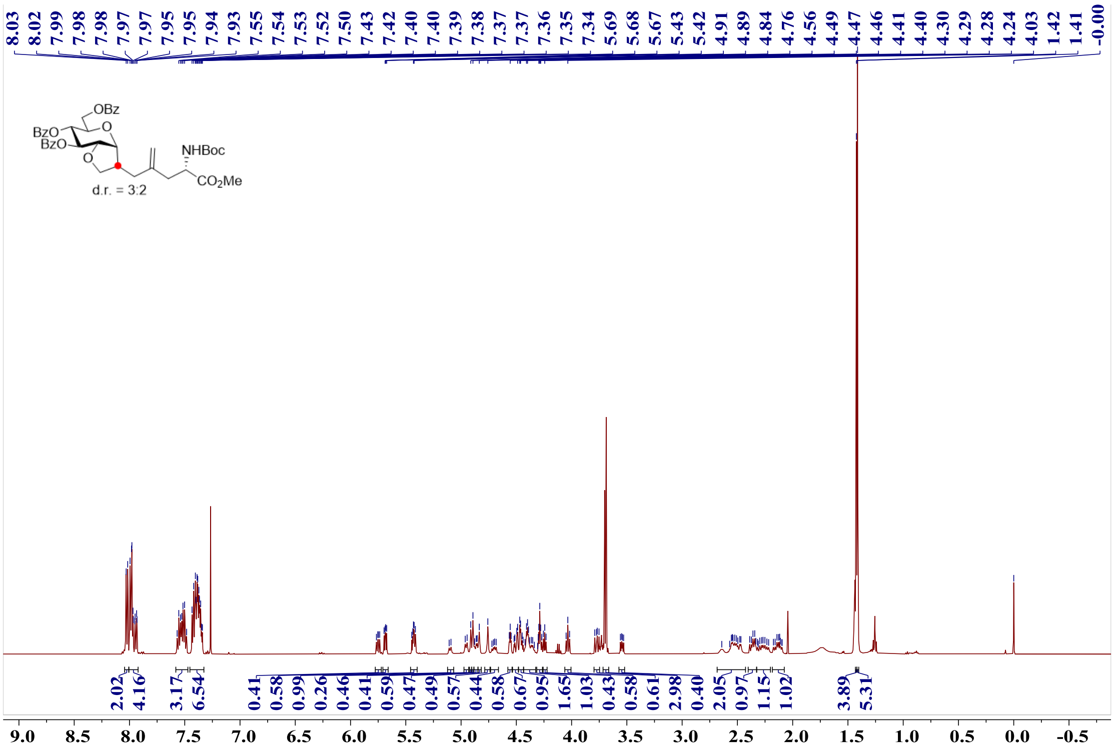

Supplementary Figure 242. ^{13}C NMR spectrum of compound **7** (125 MHz, $CDCl_3$, 25 °C)

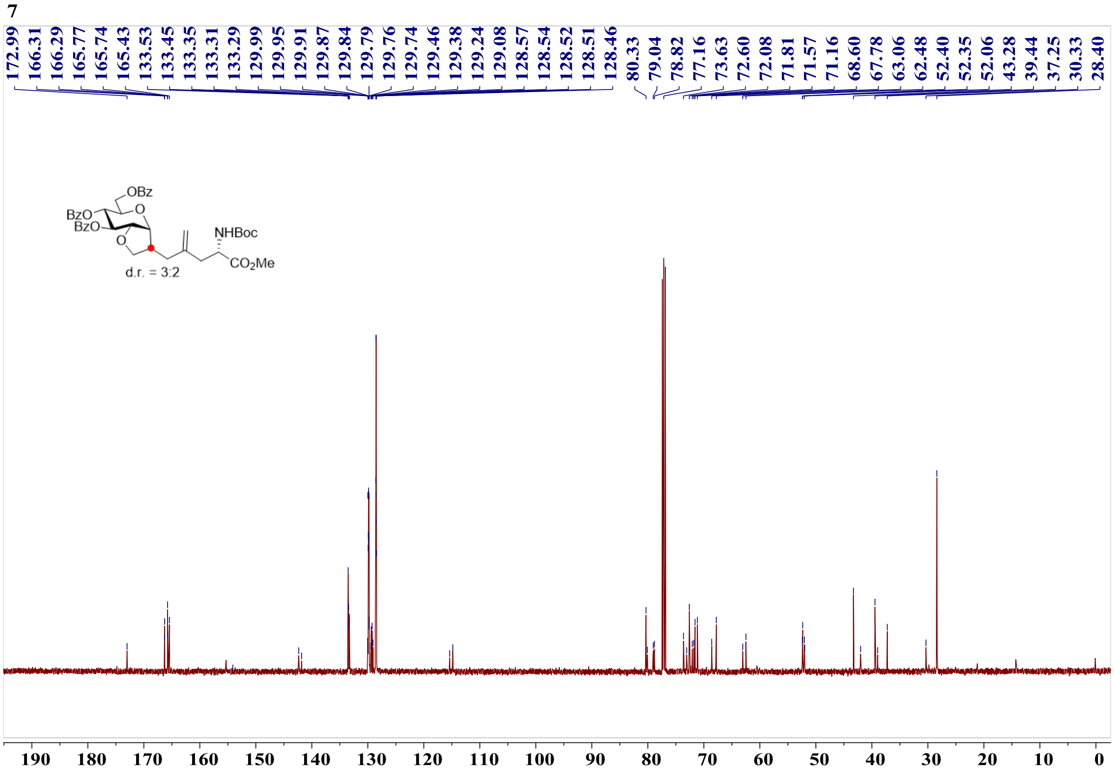

Supplementary Figure 243. ^{1}H NMR spectrum of compound **8** (500 MHz, $CDCl_3$, 25 ^{o}C)

8

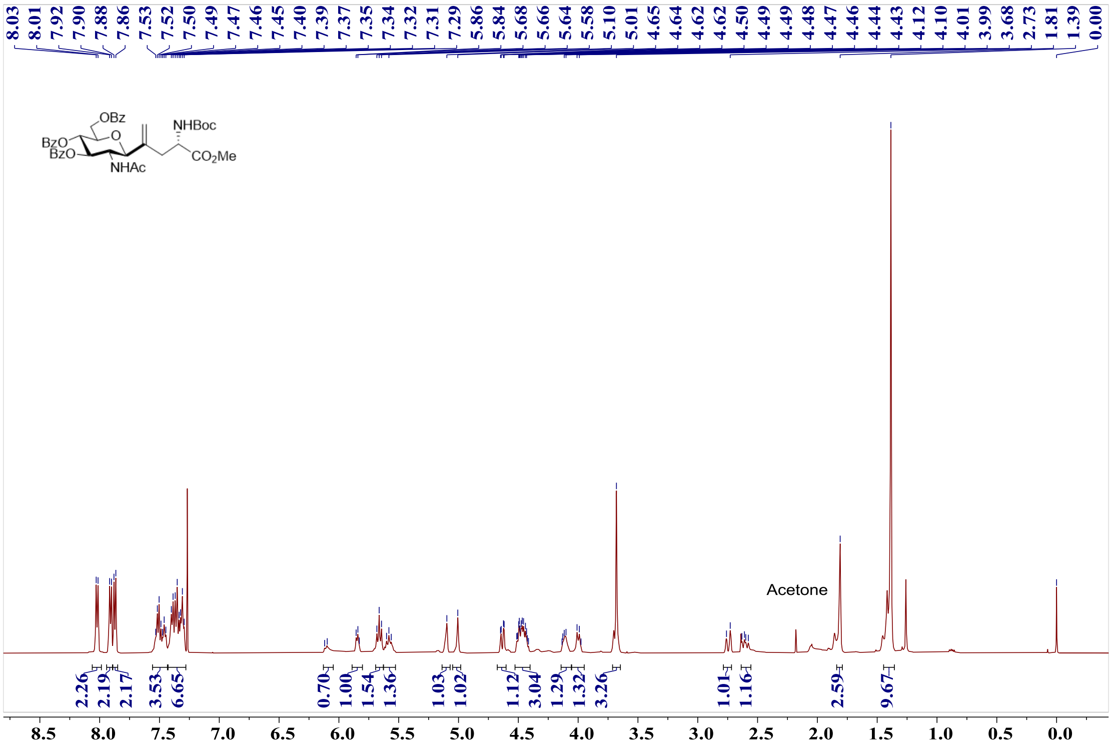

Supplementary Figure 244. ^{13}C NMR spectrum of compound **8** (125 MHz, $CDCl_3$, 25 ^{o}C)

8

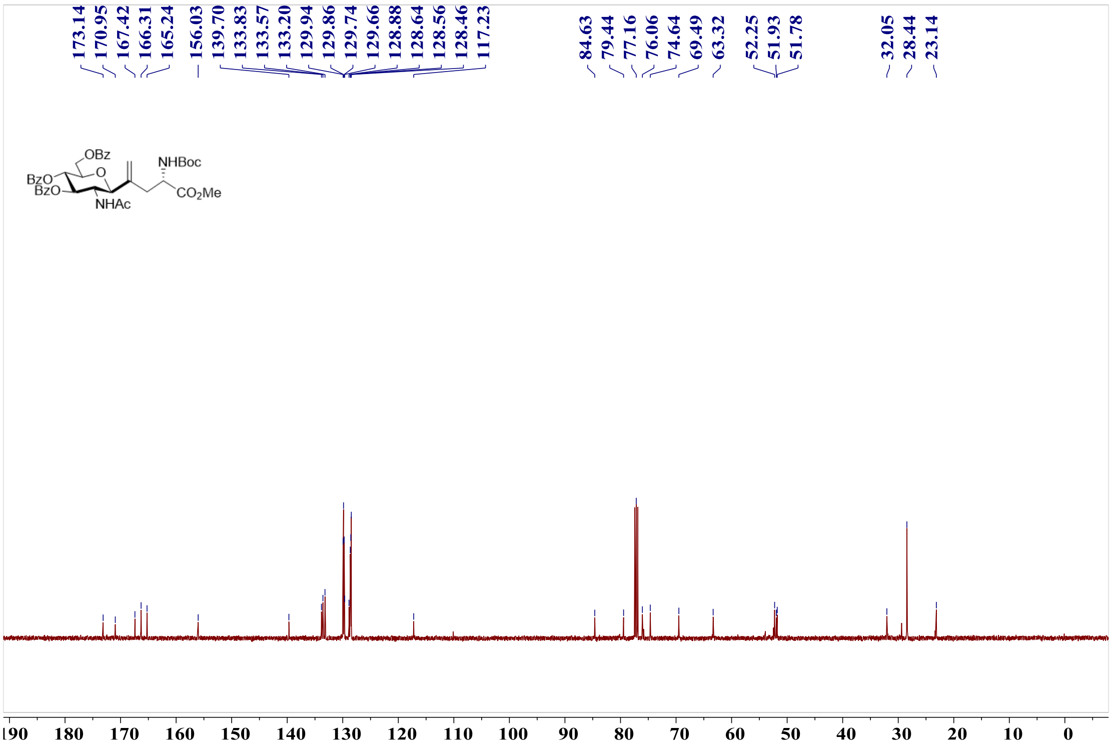

Supplementary Figure 245. ^{1}H NMR spectrum of compound **8-*R*** (500 MHz, $CDCl_3$, 25 °C)

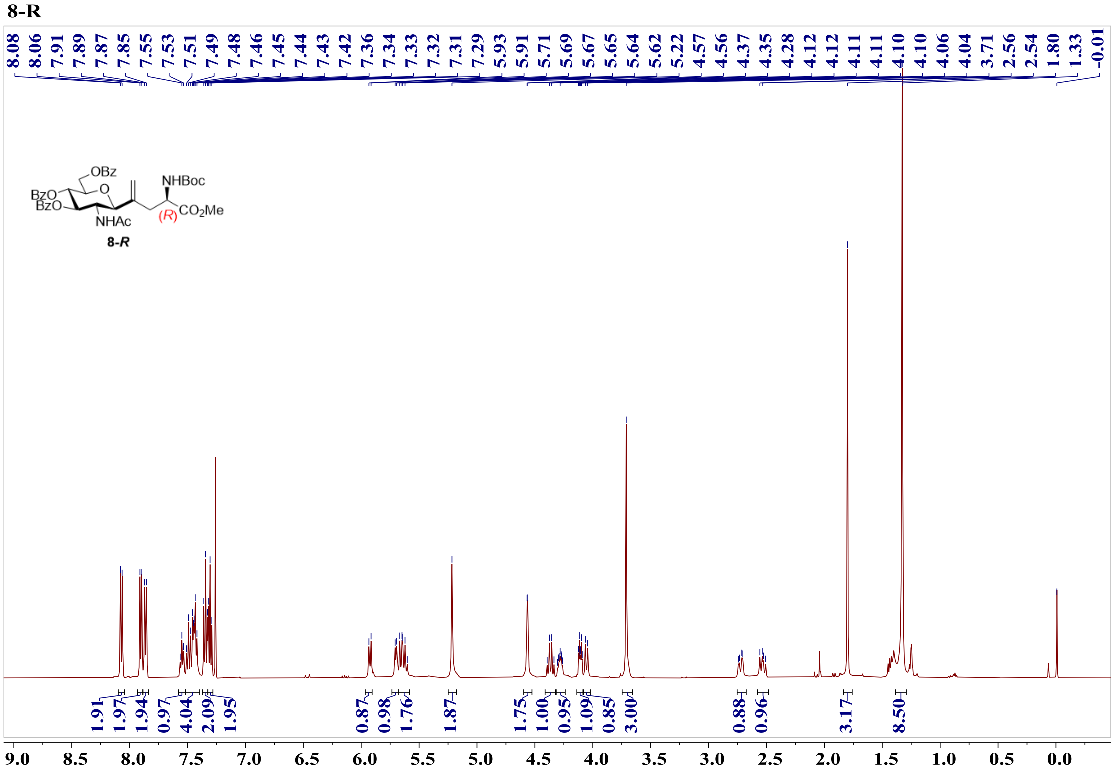

Supplementary Figure 246. ^{13}C NMR spectrum of compound **8-*R*** (125 MHz, $CDCl_3$, 25 °C)

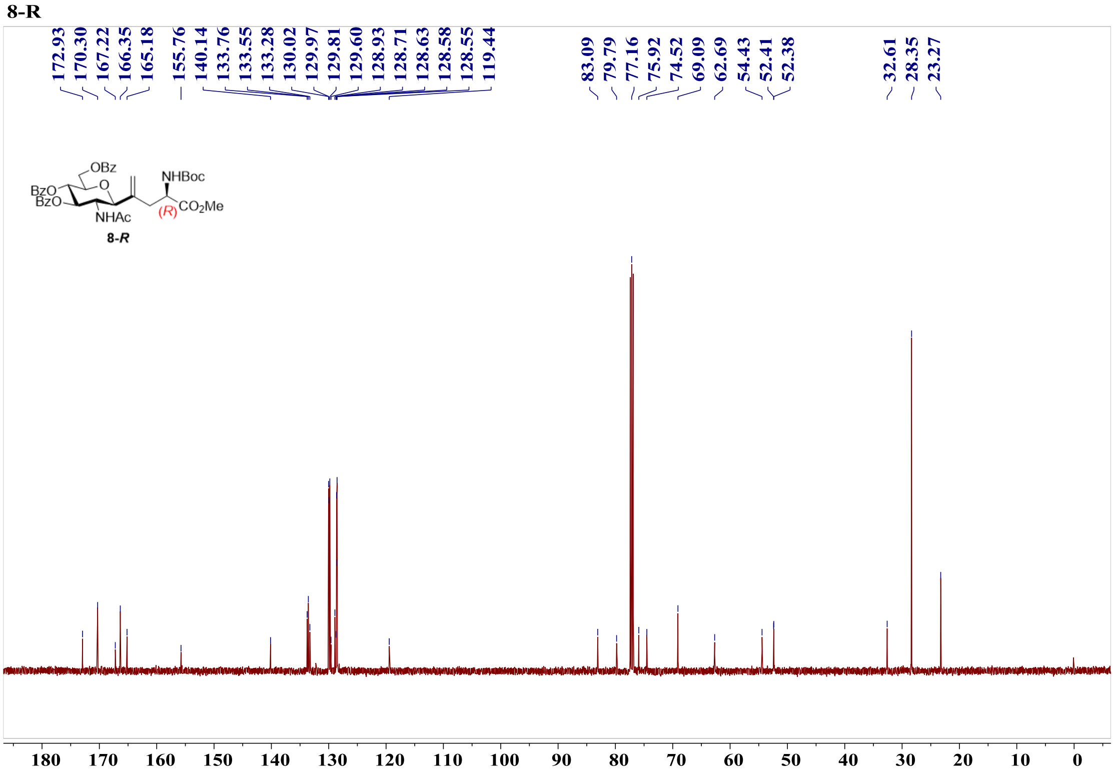

Supplementary Figure 247. ^{1}H NMR spectrum of compound **S39** (500 MHz, $CDCl_3$, 25 °C)

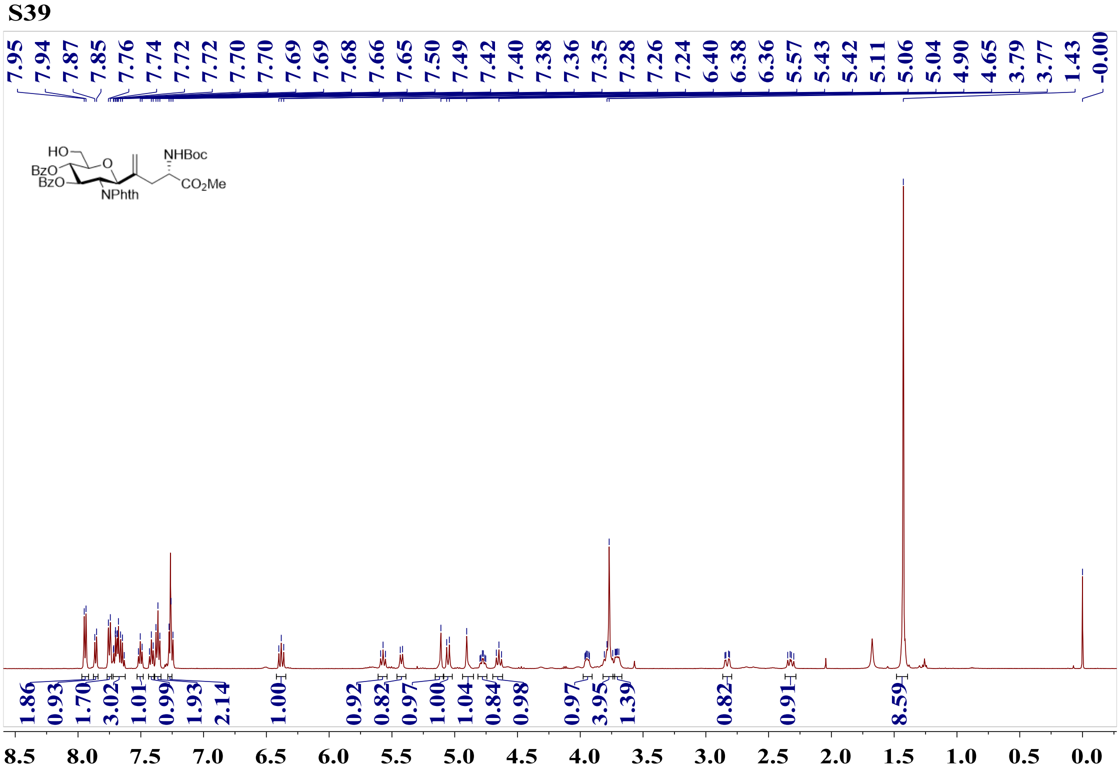

Supplementary Figure 248. ^{13}C NMR spectrum of compound **S39** (125 MHz, $CDCl_3$, 25 °C)

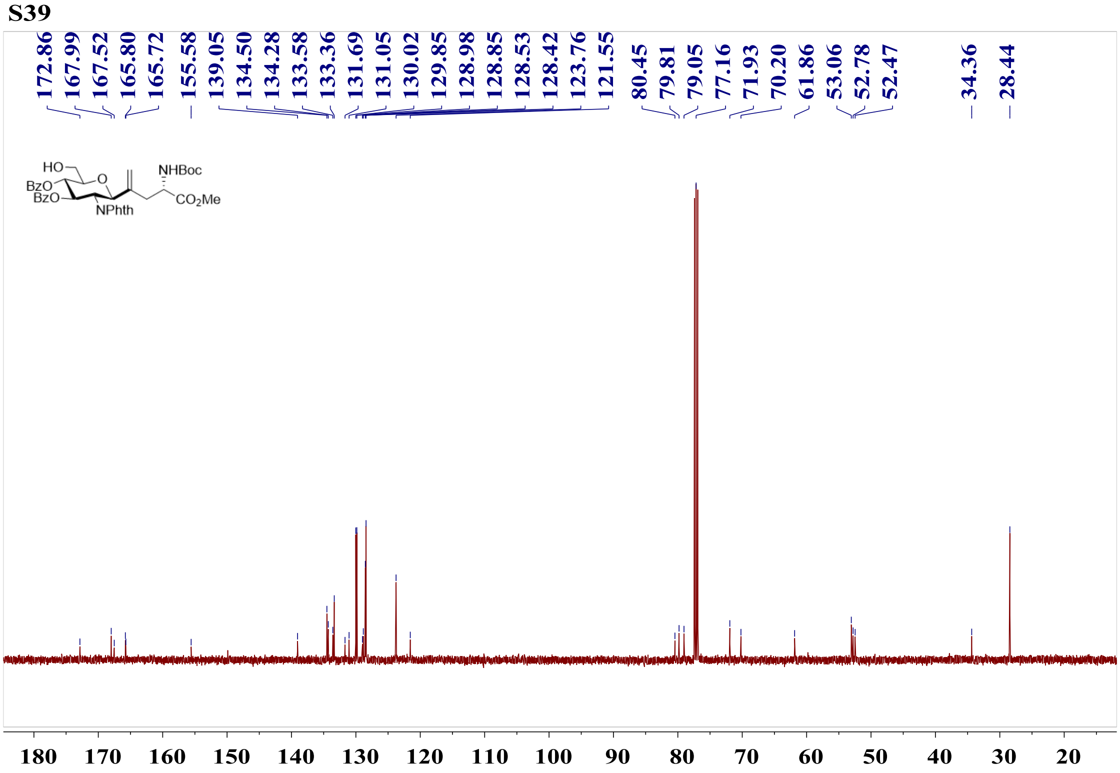

Supplementary Figure 249. ^{1}H NMR spectrum of compound **9** (500 MHz, $CDCl_3$, 25 °C)

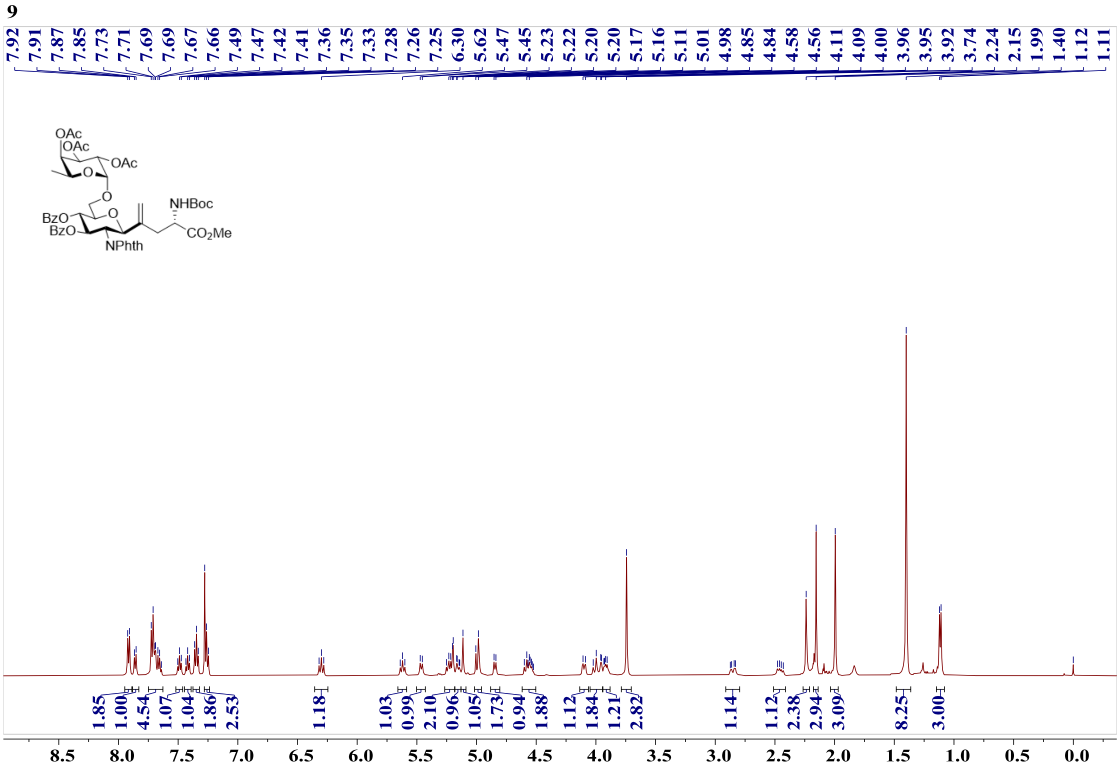

Supplementary Figure 250. ^{13}C NMR spectrum of compound **9** (125 MHz, $CDCl_3$, 25 °C)

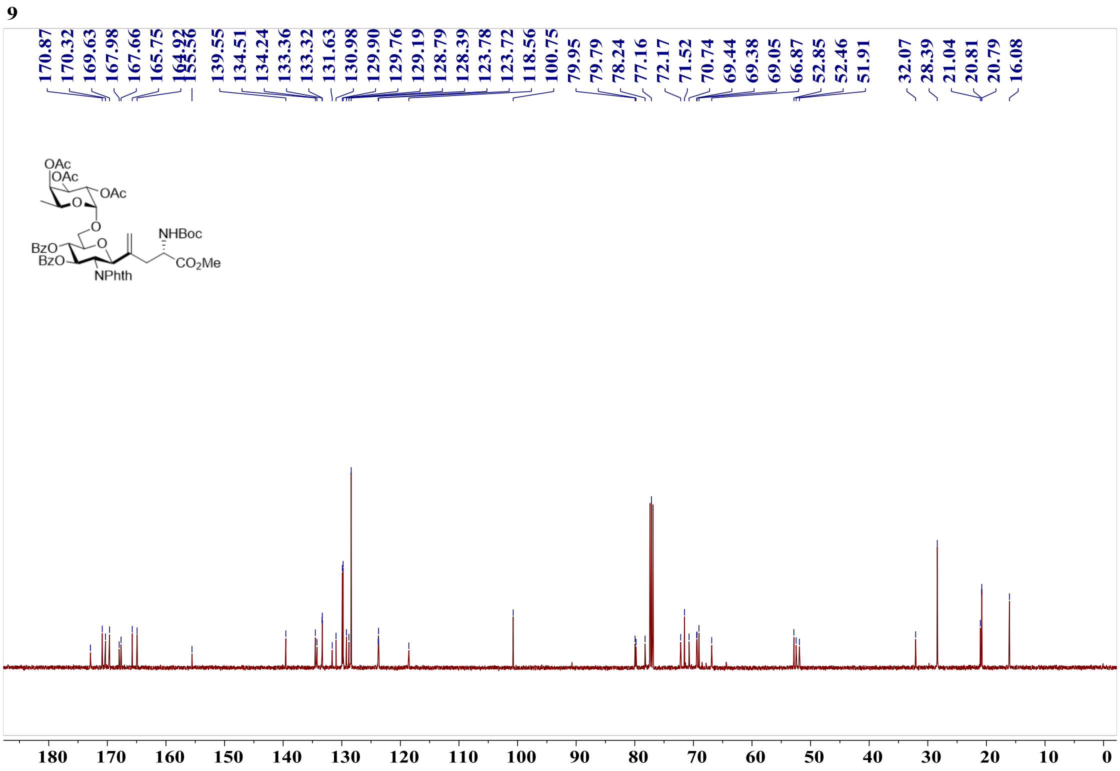

Supplementary Figure 251. ^1H NMR spectrum of compound **10** (500 MHz, $CDCl_3$, 25 °C)

10

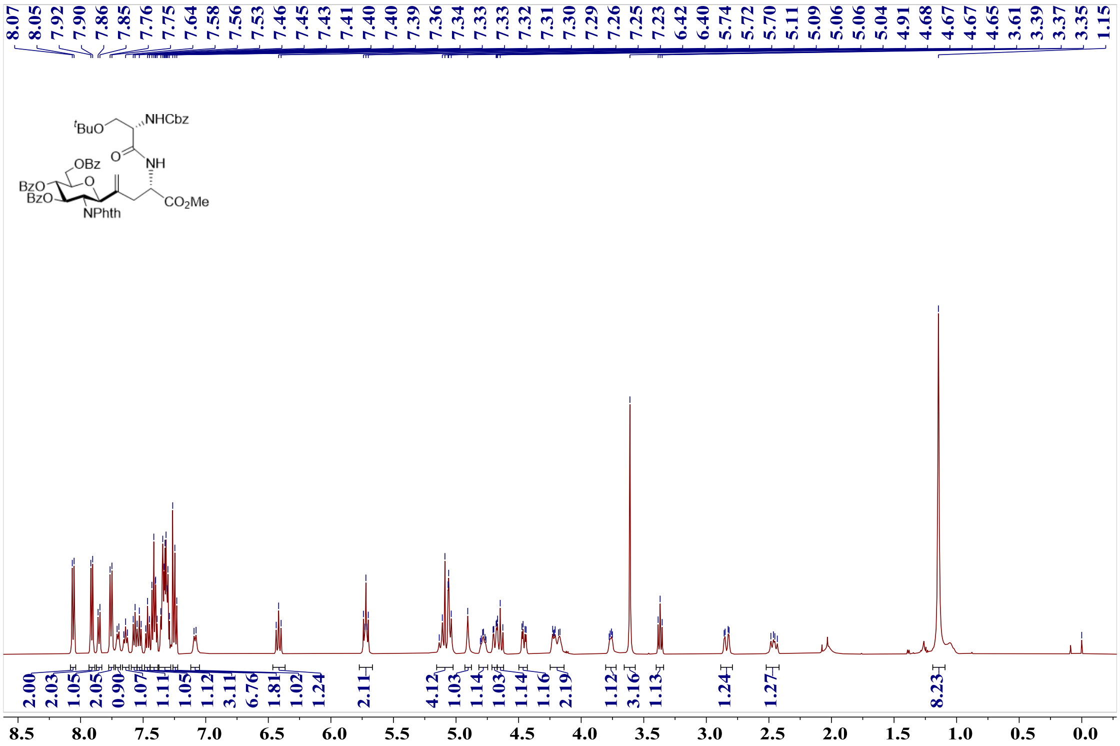

Supplementary Figure 252. ^{13}C NMR spectrum of compound **10** (125 MHz, $CDCl_3$, 25 °C)

10

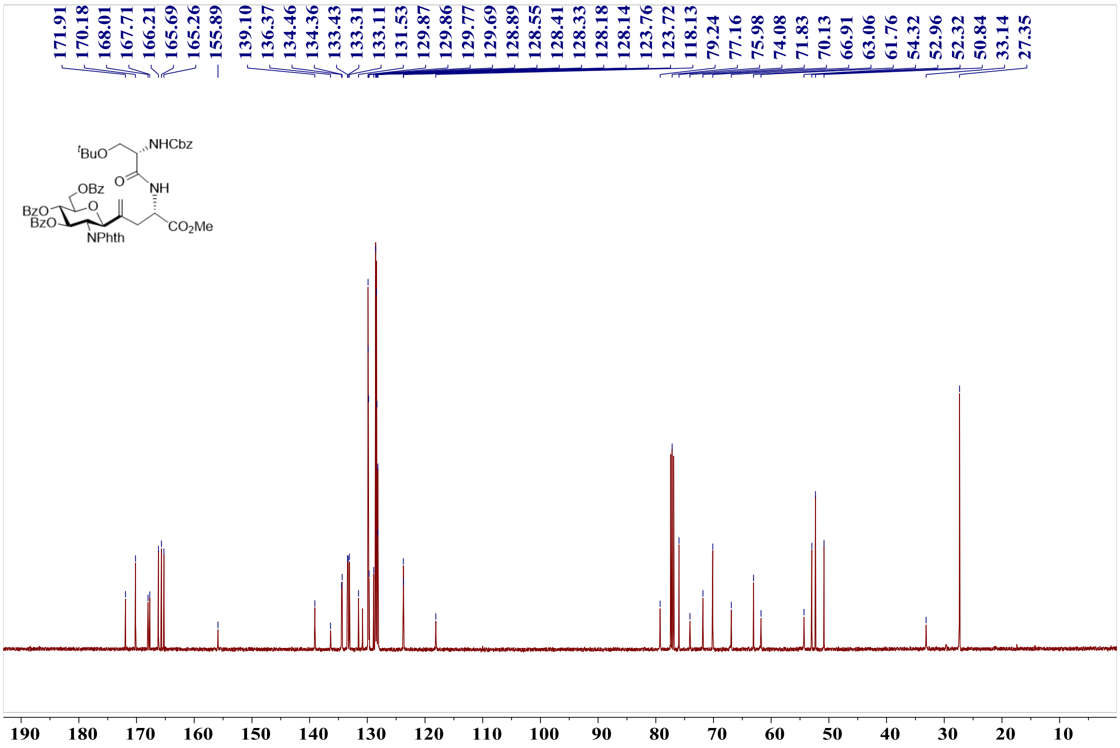

Supplementary Figure 253. ^{1}H NMR spectrum of compound **11** (500 MHz, D_2O, 25 °C)

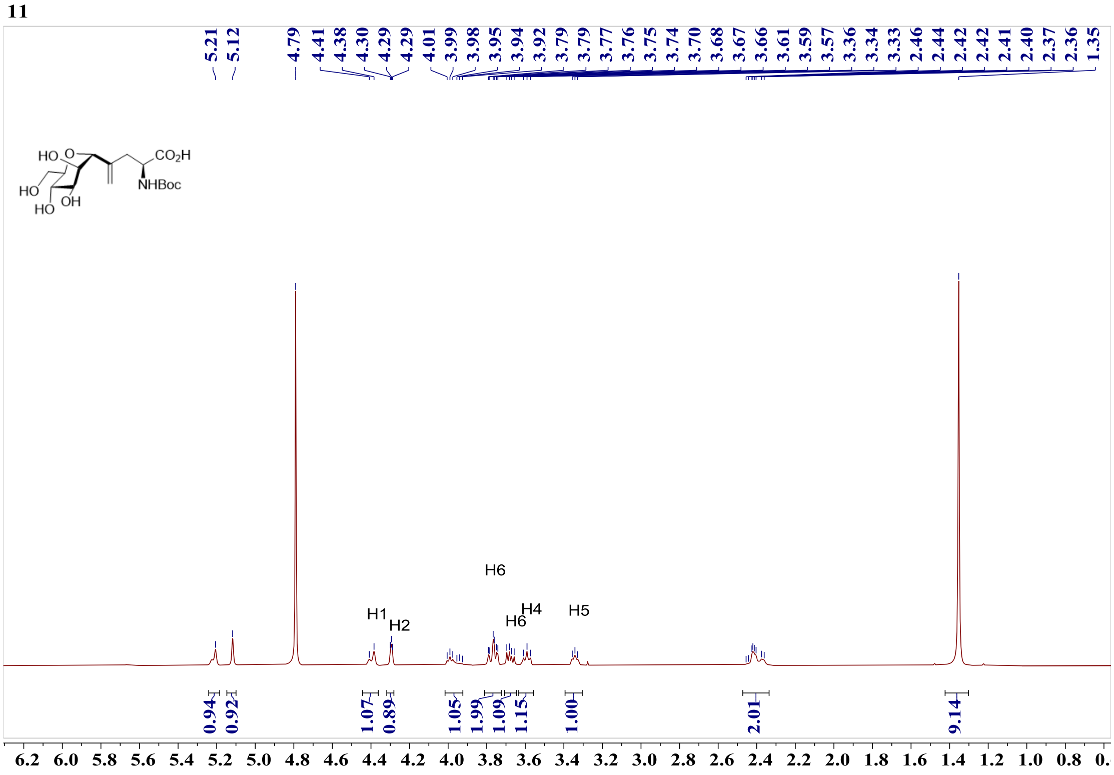

Supplementary Figure 254. ^{13}C NMR spectrum of compound **11** (125 MHz, D_2O, 25 °C)

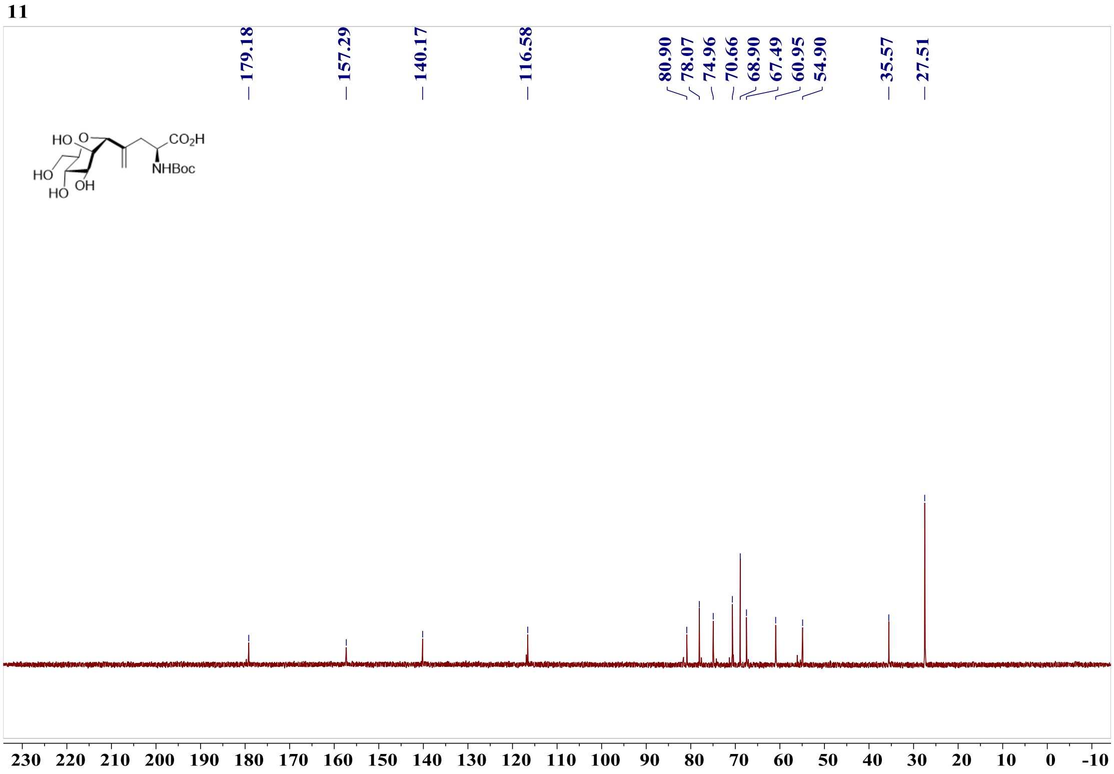

Supplementary Figure 255. ^{1}H NMR spectrum of compound **12** (500 MHz, CD_3OD, 25 °C)

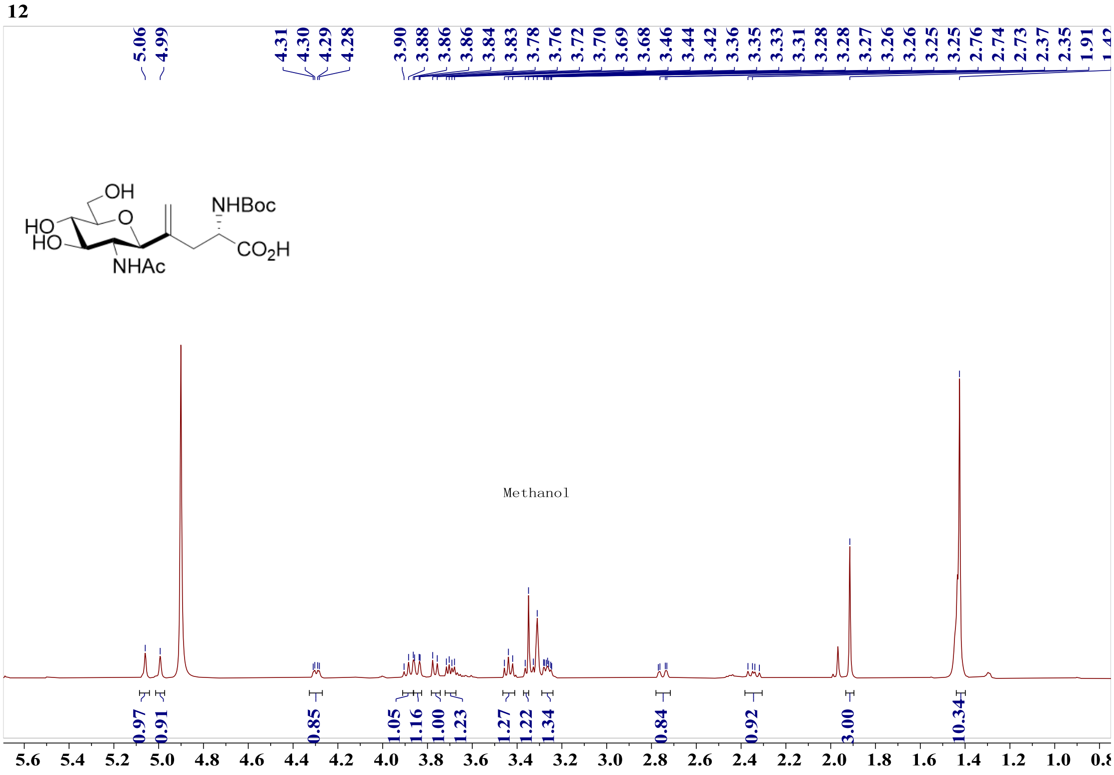

Supplementary Figure 256. ^{13}C NMR spectrum of compound **12** (125 MHz, CD_3OD, 25 °C)

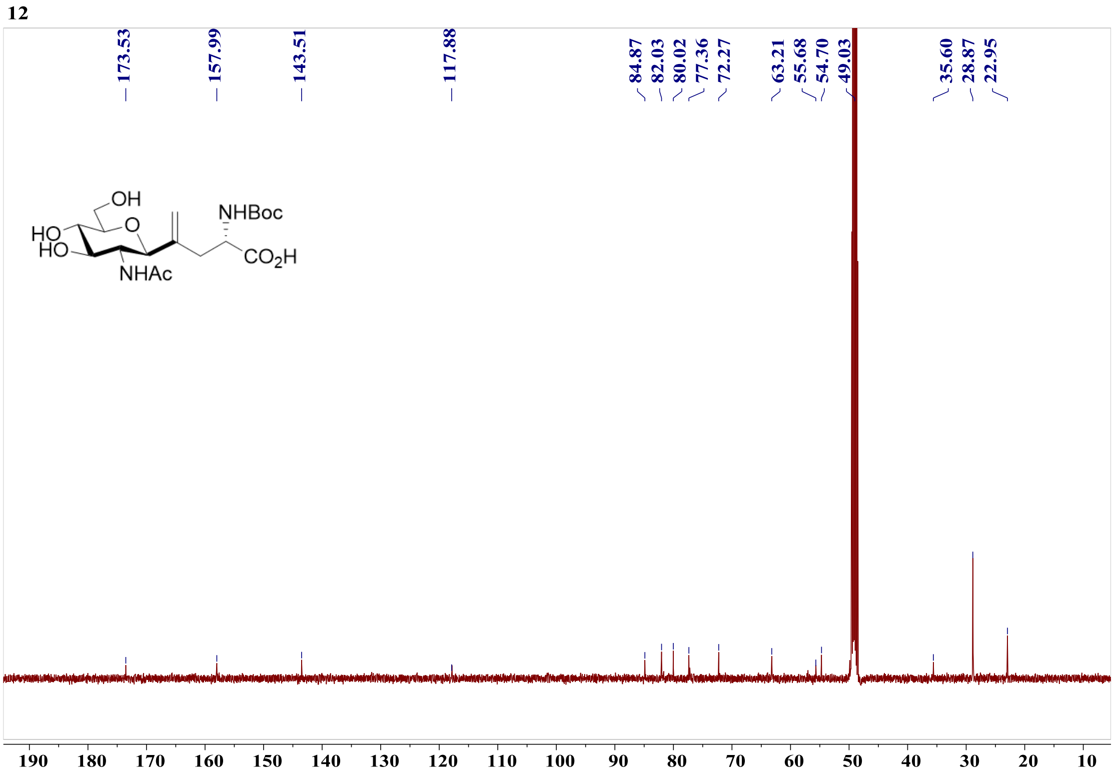

Supplementary Figure 257. ^{1}H NMR spectrum of compound **13** (500 MHz, CD_3OD, 25 °C)

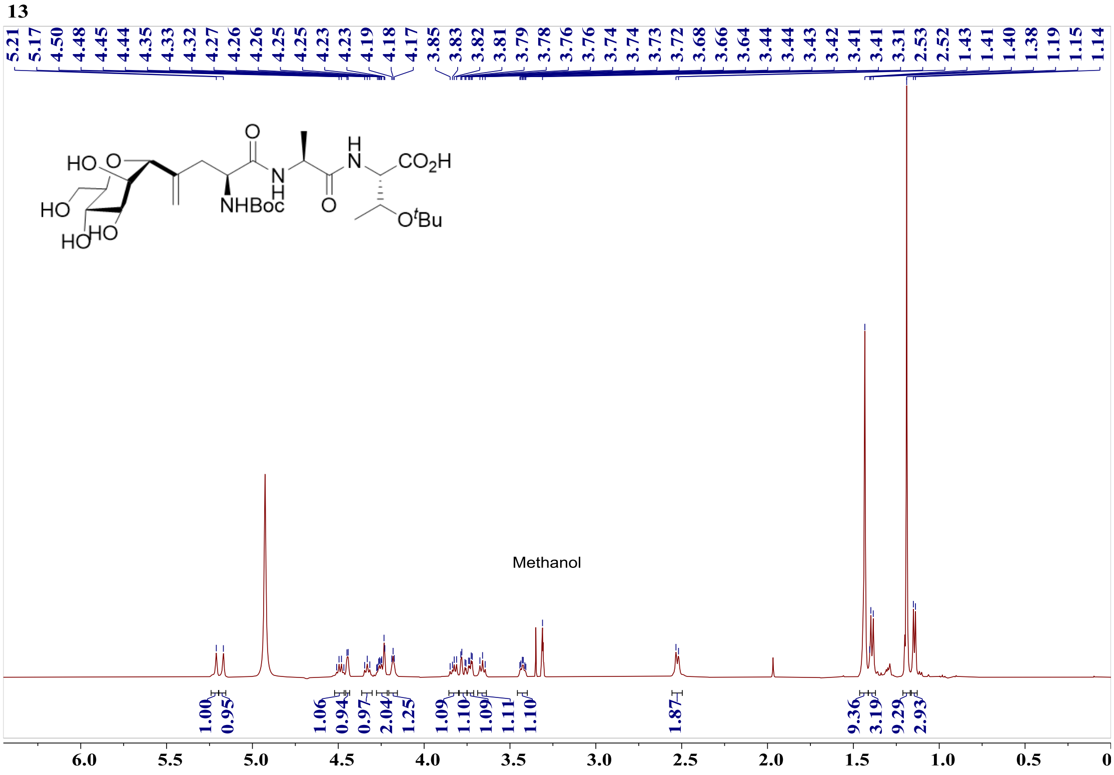

Supplementary Figure 258. ^{13}C NMR spectrum of compound **13** (125 MHz, CD_3OD, 25 °C)

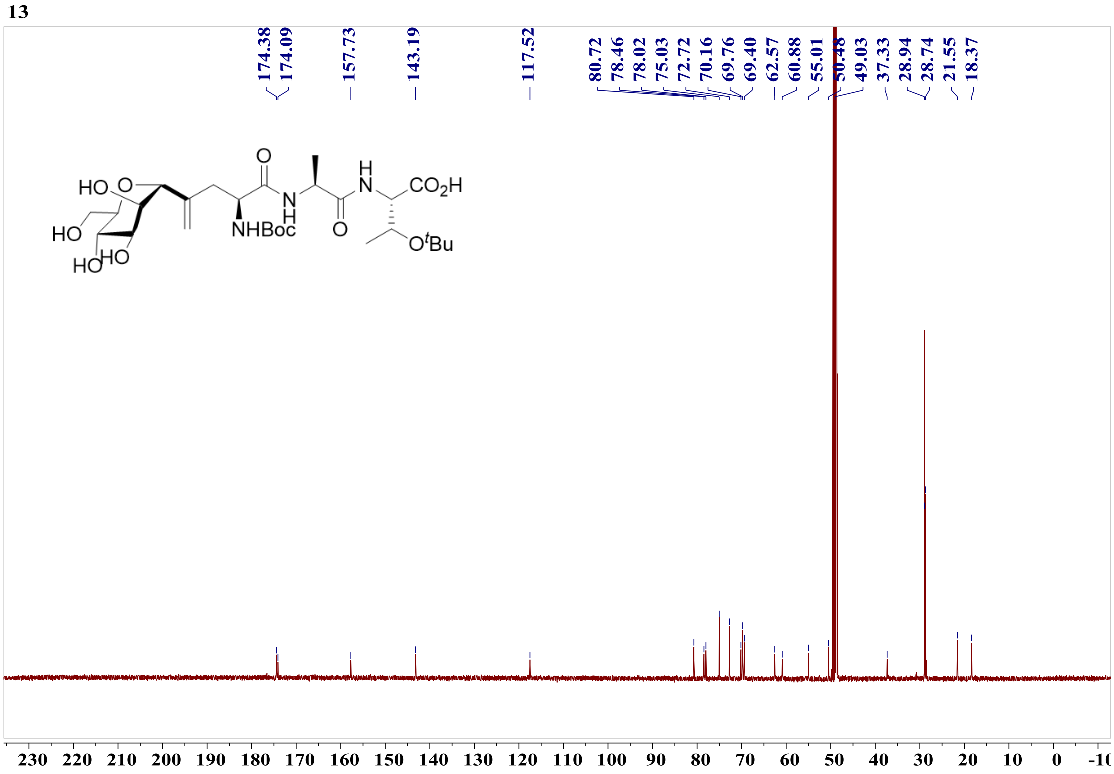

Supplementary Figure 259. ^{1}H NMR spectrum of compound **14** (600 MHz, $CDCl_3$, 25 °C)

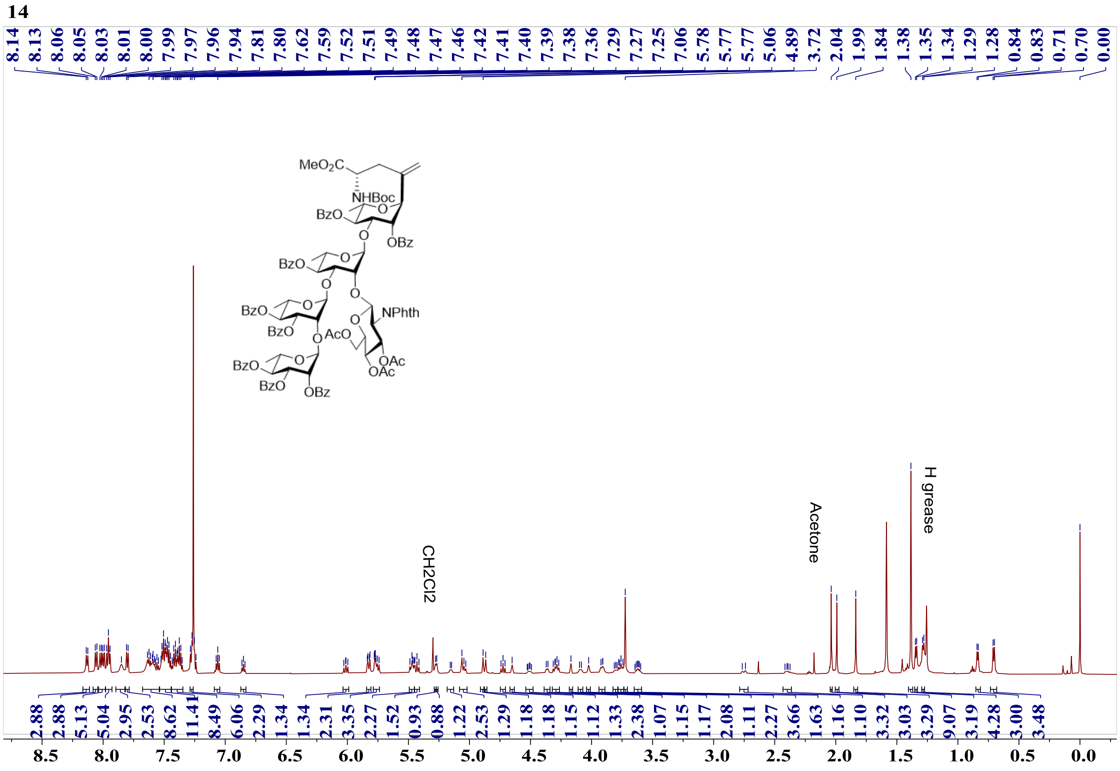

Supplementary Figure 260. ^{13}C NMR spectrum of compound **14** (150 MHz, $CDCl_3$, 25 °C)

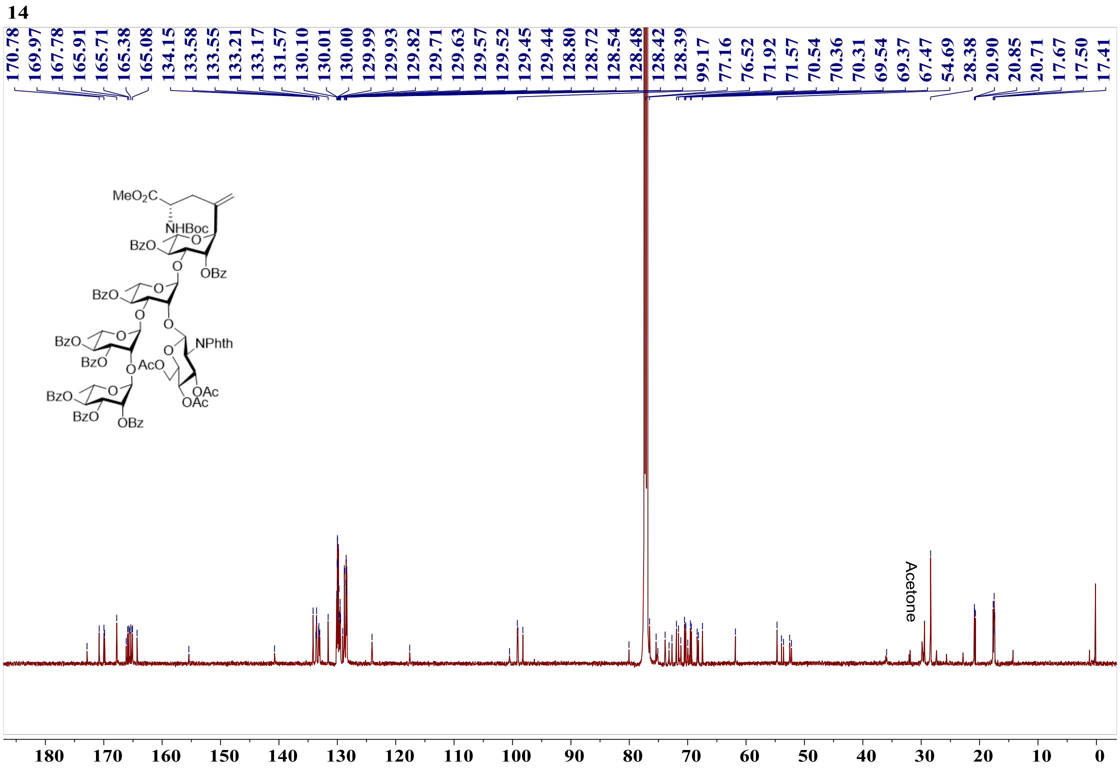

Supplementary Figure 261. ^{1}H NMR spectrum of compound **15** (600 MHz, $CDCl_3$, 25 ^{o}C)

15

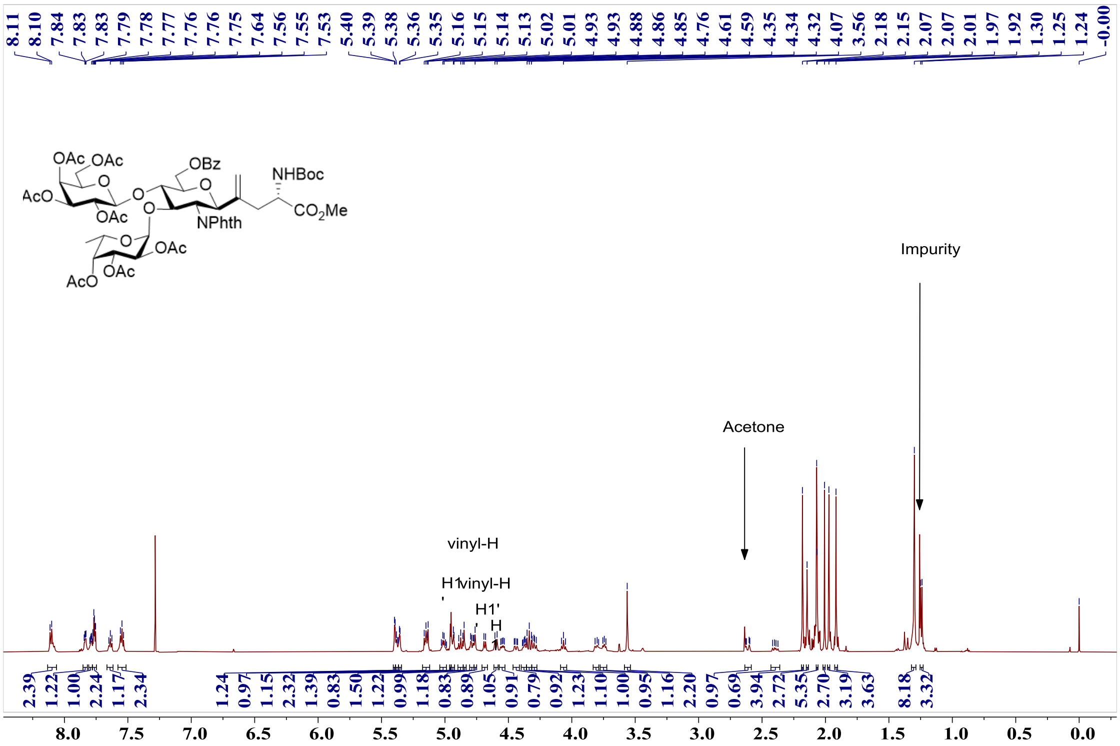

Supplementary Figure 262. ^{13}C NMR spectrum of compound **15** (150 MHz, $CDCl_3$, 25 ^{o}C)

15

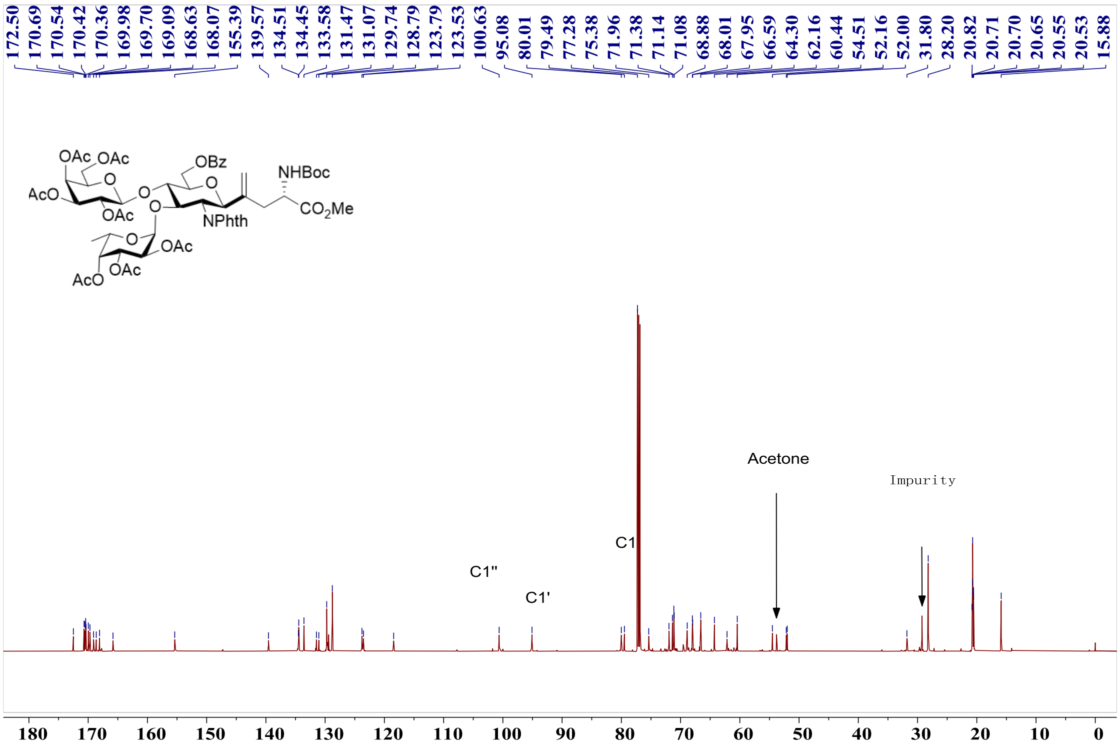

4. Supplementary References

1. Doyle, L. M. et al. Stereoselective epimerizations of glycosyl thiols. *Org. Lett.* **19**, 5802−5805 (2017).
2. Reimer, K. B., Harris, S. L., Varma, V. & Pinto, B. M. Convergent synthesis of higher-order oligosaccharides corresponding to the cell-wall polysaccharide of the β-hemolytic *streptococci* group A, a branched hexasaccharide hapten. *Carbohydr. Res.* **228**, 399−414 (1992).
3. Walczewska, A. et al. *N*-Alkyl derivatives of diosgenyl 2-amino-2-deoxy-β-D-glucopyranoside; synthesis and antimicrobial activity. *Beilstein J. Org. Chem.* **11**, 869–874 (2015).
4. Vesely, J., Ledvina, M., Jindrich, J., Saman, D. & Trnka, T. Improved synthesis of 1,2-*trans*-acetates and 1,2-*trans* ethyl 1-thioglycosides derived from 3,4,6-tri-*O*-acetyl-2-deoxy-2-phthalimido-D-hexopyranosides. *Collect. Czech. Chem. Commun.* **68**, 1264−1274 (2003).
5. Kaeothip, S., Yasomanee, J. P. & Demchenko, A. V. Glycosidation of thioglycosides in the presence of bromine: mechanism, reactivity, and stereoselectivity. *J. Org. Chem.* **77**, 291–299 (2012).
6. Hederos, M. & Konradsson, P. J. Efficient routes to ethyl-2-deoxy-2-phthalimido-1-β-D-thio-galactosamine derivatives via epimerization of the corresponding glucosamine compounds. *J. Carbohydr. Chem.* **24**, 297−320 (2005).
7. Reddy, G. V. R., Jain, R. K., Locke, R. D. & Matta, K. L. Synthesis of precursors for the dimeric 3-*O*-SO_3Na Lewis X and Lewis A structures. *Carbohydr. Res.* **280**, 261−276 (1996).
8. Zhu, T. & Boons, G.-J. Intermolecular aglycon transfer of ethyl thioglycosides can be prevented by judicious choice of protecting group. *Carbohydr. Res.* **329**, 709−715 (2000).
9. Wang, P., Wang, J., Guo, T. T. & Li, Y. Synthesis and cytotoxic activity of the N-acetylglucosamine-bearing triterpenoid saponins. *Carbohydr. Res.* **345**, 607−620 (2010).
10. Mao, Z., Zhang, J. & Kong, F. A concise synthesis of two isomeric pentasaccharides, the O repeat units from the lipopolysaccharides of *P. syringae* pv. porri NCPPB 3364^{T} and NCPPB 3365. *Carbohydr. Res.* **339**, 43−49 (2004).
11. de la Fuente, J. M. & Penadés, S. Synthesis of Le^{x}-neoglycoconjugate to study carbohydrate–carbohydrate associations and its intramolecular interaction. *Tetrahedron: Asymmetry* **13**, 1879−1888 (2002).
12. Mindt, T. L. & Schibli. R. Cu(I)-Catalyzed Intramolecular Cyclization of Alkynoic Acids in Aqueous Media: A “Click Side Reaction”. *J. Org. Chem.* **72**, 10247–10250 (2007).
13. Groothuys, S. et al. Chemoenzymatic synthesis of triazole-linked glycopeptides. *Synthesis* **18**, 3146−3152 (2006).

14. Roldan, R. J. et al. Propargylglycine-based antimicrobial compounds are targets of TolC-dependent efflux systems in *Escherichia coli. Bioorg. Med. Chem. Lett.* **30**, 126875 (2020).
15. Yamada, H., Hayashi, T. & Usuki, T. Total synthesis of the COPD biomarker desmosine via stepwise Sonogashira cross-coupling reactions. *Bull. Chem. Soc. Jpn.* **88**, 673−683 (2015).
16. Murakami, Y., Yanuma, H. & Usuki, T. Total synthesis of the elastin crosslinker (+)-desmopyridine. *Tetrahedron: Asymmetry* **23**, 1557–1563 (2012).
17. Ye, X. et al. Silver-free palladium-catalyzed sp^3 and sp^2 C–H alkynylation promoted by a 1,2,3-triazole amine directing group. *Org. Lett.* **18**, 2970–2973 (2016).
18. Sminia, T. J. & Pedersen, D. S. Azide- and alkyne-functionalised α- and β^3-amino acids. *Synlett* **23**, 2643–2646 (2012).
19. Lutteroth, K. R. et al. Incorporation of 'click' chemistry glycomimetics dramatically alters triple-helix stability in an adiponectin model peptide. *Org. Biomol. Chem.* **15**, 5602−5608 (2017).
20. Maurits, E. et al. Immunoproteasome inhibitor–doxorubicin conjugates target multiple myeloma cells and release doxorubicin upon low-dose photon irradiation. *J. Am. Chem. Soc.* **142**, 7250–7253 (2020).
21. Icik, E. et al. Synthesis and biological evaluation of a library of AGE-related amino acid triazole crosslinkers. *Eur. J. Org. Chem.* **33**, 5368–5379 (2020).
22. van Esseveldt, B. C. J. et al. Novel approach to 5-substituted proline derivatives using a silver-catalyzed cyclization as the key step. *J. Org. Chem.* **70**, 1791–1795 (2005).
23. Brehm, M., Göckel, V. H., Jarglis, P. & Lichtenthaler, F. W. Expedient conversion of D-glucose into 1,5-anhydro-D-fructose and into single stereogenic-center dihydropyranones, suitable six-carbon scaffolds for concise syntheses of the soft-coral constituents (−)-bissetone and (−)-palythazine. *Tetrahedron: Asymmetry* **19**, 358–373 (2008).
24. Cui, J. & Horton, D. Stereocontrolled allylation of 2-amino-2-deoxy sugar derivatives by a free-radical procedure. *Carbohydr. Res.* **309**, 319–330 (1998).
25. Takaya, H., Mashima, K. & Koyano, K. Practical synthesis of (R)- or (S)-2,2'-bis(diarylphosphino)-1,1'-binaphthyls (BINAPs). *J. Org. Chem.* **51**, 629–635 (1986).
26. Jiao, Y. et al. Clostridium difficile PSI polysaccharide: synthesis of pentasaccharide repeating block, conjugation to exotoxin B subunit, and detection of natural anti-PSI IgG antibodies in horse serum. *Carbohydr. Res.* **378**, 15–25 (2013).
